# New insights into the proteins interacting with the promoters of silkworm fibroin genes

**DOI:** 10.1038/s41598-021-95400-0

**Published:** 2021-08-05

**Authors:** Yan Ma, Qin Luo, Yao Ou, Yiyun Tang, Wenhui Zeng, Haomiao Wang, Jie Hu, Hanfu Xu

**Affiliations:** State Key Laboratory of Silkworm Genome Biology, College of Sericulture, Textile and Biomass Sciences, Southwest University, Chongqing, 400715 China

**Keywords:** Molecular biology, Zoology

## Abstract

The silkworm, *Bombyx mori*, is a silk-producing insect that has contributed greatly to human society. The silk gland of *B. mori* is a specialized organ responsible for synthesizing silk fibroin and sericin proteins under control of numerous factors. However, which factors are involved in direct silk protein synthesis regulation remains largely unknown. We report the identification of promoter-interacting proteins (PIPs) necessary for the regulation of genes encoding fibroin proteins, including the *fibroin heavy chain* (*fibH*), *fibroin light chain* (*fibL*), and a 25-kD polypeptide protein (*P25*). In the fourth larval molting stage (M4) or day 5 fifth-instar larvae (L5D5), a total of 198, 292, and 247 or 330, 305, and 460 proteins interacting with the promoter region of *fibH*, *fibL* and *P25*, respectively, were identified from the posterior silk gland by DNA pull-down combined with mass spectrometry. Many PIPs were particularly involved in ribosome- and metabolism-related pathways. Additionally, 135 and 212 proteins were identified as common PIPs of *fibH*, *fibL* and *P25* in M4 and L5D5, respectively. Among all PIPs, we identified 31 potential transcription factors, such as Y-box and poly A-binding proteins, which play roles in nucleotide binding, ATP binding, or protein folding. This study provides the first in-depth profile of proteins interacting with fibroin gene promoters and contributes to a better understanding of silk protein synthesis regulation.

## Introduction

The temporal and spatial control of gene expression, which is mainly achieved through the precise regulation of gene promoters, is indispensable for many biological processes^1^. The regulation of gene promoters is a rather complex process due to the involvement of various regulatory factors that interact directly with promoters and activate/inhibit their activities. For each target gene, regulatory factors are ubiquitous or tissue-specific proteins that associate with promoter DNA and regulate gene expression through a variety of mechanisms, leading to alternative cell fates and behaviors and controlling organ development^2–4^. Therefore, elucidating the regulatory factors that bind directly to the promoter regions of target genes is a key step toward ascertaining their functions as well as regulatory mechanisms.

The silkworm, *Bombyx mori*, is a lepidopteran insect of great importance because of its use in silk production and its research value for understanding the biology of insects^5^. The silk gland (SG) of *B. mori* is a specialized organ in which silk proteins are efficiently synthesized and spun out to build cocoons. Generally, the SG is divided into three morphologically and functionally distinct parts: the anterior silk gland (ASG), the middle silk gland (MSG), and the posterior silk gland (PSG)^6^. PSG is the only part that synthesizes fibroin protein, a major component that accounts for approximately 75% of silk fibers^7^. The regulation of fibroin protein synthesis is particularly interesting and rather complex. In brief, the expression of genes encoding fibroin proteins, including the *fibroin heavy chain* (*fibH*), *fibroin light chain* (*fibL*), and a 25-kD polypeptide protein (*P25*), is synchronously switched on during intermolt larval stages and switched off during molt stages. Remarkably, massive amounts of fibroin protein are rapidly synthesized in PSG cells beginning on the 3^rd^ day of the fifth instar in larvae^8,9^. Previous studies have suggested that fibroin gene expression is regulated by many regulatory factors at the transcriptional level in a concerted manner^10,11^. It is therefore of profound interest to elucidate the factors involved to better understand the regulatory mechanisms of both SG development and silk protein synthesis.

In recent years, great efforts have been made to identify the factors regulating fibroin genes. In particular, transcription factors (TFs) have attracted the most attention due to their vital roles in the transcriptional regulation of gene expression^12^. In *B. mori*, only a few TFs, including FMBP-1, SGF-2, Bmdimm, POU-M2, and BmFTZ-F1^13–19^, have been shown to regulate the expression of fibroin genes via direct binding to their promoter regions. A comprehensive understanding of the regulatory factors determining fibroin gene expression is still lacking, especially for those factors that directly interact with fibroin genes.

In this study, we aimed to elucidate the promoter-interacting proteins (PIPs) that regulate fibroin gene transcription and protein synthesis. By using the DNA pull-down strategy combined with mass spectrometry, we identified a large number of PIPs of *fibH*, *fibL*, and *P25* from the PSG in two representative developmental stages, the fourth larval molting stage (M4) and day 5 fifth-instar larvae (L5D5). Further analyses revealed common and unique PIPs between M4 and L5D5 as well as their functional characteristics and interaction relationships. Overall, these results provide preliminary information and new insights for understanding the regulatory factors associated with fibroin genes.

## Results

### Enrichment of PIPs using 5’-biotinylated DNA probes

**Figure 1:**
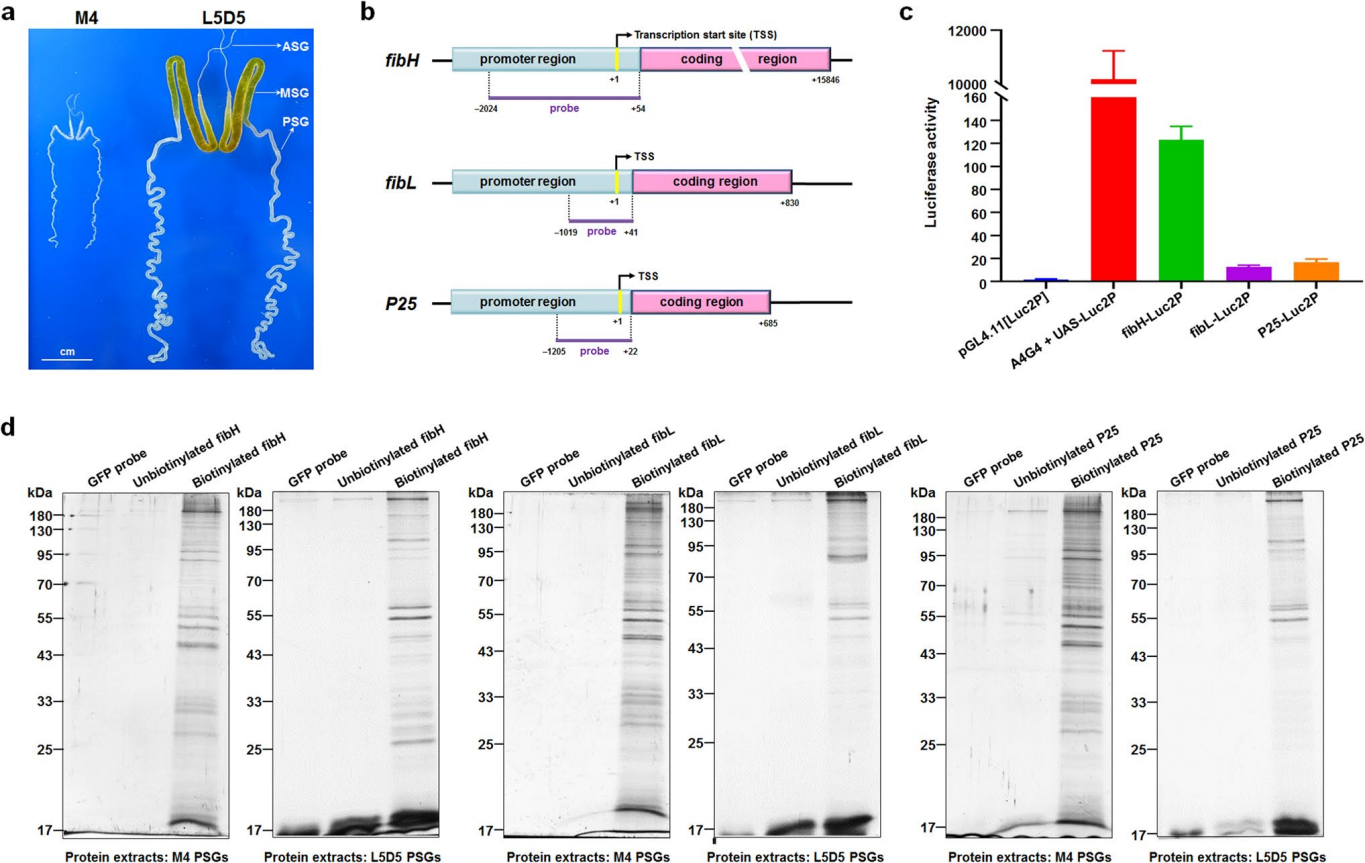
Probe design and DNA pull-down assay. (**a**) SGs dissected from silkworm larvae developed to M4 and L5D5. (**b**) Diagram of the fibroin genes *fibH*, *fibL*, and *P25* and the locations of DNA probes. (**c**) Promoter activities of the *fibH*, *fibL*, and *P25* genes measured by luciferase reporter assays. (**d**) Potential PIPs in PSG nuclear protein extracts were pulled down using the probes (full-length gels are presented in Supplementary Fig. S2). After SDS-PAGE and silver staining, the bound proteins were significantly enriched only in those groups in which 5’-biotinylated probes were used.

To identify the PIPs of each fibroin gene in M4 and L5D5 PSGs (Fig. 1a), we first examined the promoter activities of *fibH*, *fibL*, and *P25* in cultured BmE cells. Significantly, the luciferase activities driven by the promoters of *fibH*, *fibL*, and *P25* were approximately 133-, 13-, and 20-fold higher than that of the negative control, demonstrating that the promoter region of each fibroin gene contains regulatory sequences related to its expression (Fig. 1b, c). Furthermore, we generated 5’-biotinylated DNA probes based on sequences of the *fibH*, *fibL*, and *P25* promoters shown in Fig. 1c as well as two control probes (Supplementary Fig. S1), and DNA pull-down assays were subsequently performed. As shown in Fig. 1d and Supplementary Fig. S2, multiple proteins from either the M4 or L5D5 PSG showed obvious binding to each of the biotin-labeled DNA probes specific for the *fibH*, *fibL*, and *P25* promoter regions in comparison with the control group results, demonstrating that the pull-down assays were effective and successful.

### Summary of PIPs determined by HPLC–MS

HPLC–MS was employed to determine the candidate PIPs of each fibroin gene, which are summarized in Table 1 (detailed see Supplementary Table S1). After removing the proteins captured by both negative control DNA probes, we finally identified 198 and 330 PIPs of *fibH*, 292 and 305 PIPs of *fibL*, and 247 and 460 PIPs of *P25* from the M4 and L5D5 PSGs, respectively. Notably, the PIPs identified from the L5D5 PSG were much more abundant than those from the M4 PSG, implying that more proteins are recruited to regulate the expression of fibroin genes in L5D5, a key period of the efficient synthesis of massive amounts of fibroin protein in the PSG. Moreover, the number of *P25* PIPs identified from the L5D5 PSG was much greater than the numbers of *fibH* and *fibL* PIPs, which was a surprising finding and deserves further study. Preliminary Gene Ontology (GO) and Kyoto Encyclopedia of Genes and Genomes (KEGG) pathway annotation revealed that the specific PIPs of each group showed some degree of similarity in terms of functional classification (Supplementary Figs. S3 and S4), suggesting that these PIPs may play important roles in the cooperative regulation of fibroin gene expression. Next, we will focus on these common, interesting PIPs to explore their similarities and differences.

**Table 1:** Summary of candidate PIPs identified by HPLC–MS.

Gene promoters	Developmental stages	Control group (GFP probe)	Control group (unbiotinylated probe)	Experimental group (biotinylated probe)	Specific PIPs
*fibH*	M4	34	18	232	198
	L5D5	39	23	373	330
*fibL*	M4	34	23	325	292
	L5D5	39	77	387	305
*P25*	M4	34	40	291	247
	L5D5	39	28	504	460

### Comparison of PIPs between the M4 and L5D5 PSGs

**Figure 2:**
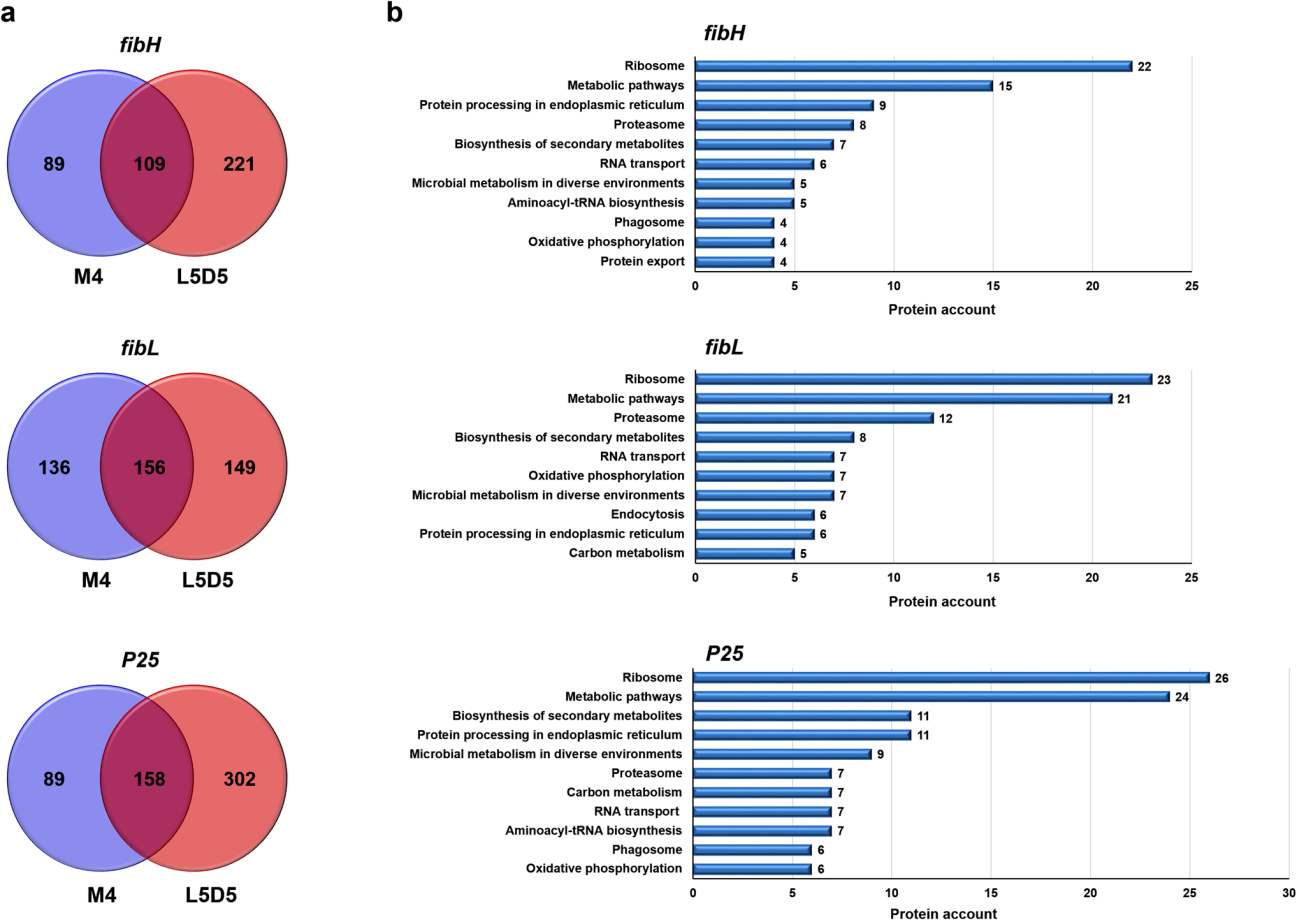
Overlap between the PIPs of each fibroin gene in M4 and L5D5. (**a**) Venn diagrams showing the numbers of common and unique PIPs of the *fibH*, *fibL*, and *P25* genes. (**b**) KEGG pathway enrichment of common PIPs. Top 10 KEGG pathways that excludes those related to human diseases are displayed.

To better understand the characteristics of these PIPs, we first analyzed the common and unique PIPs identified in the M4 and L5D5 PSGs corresponding to each fibroin gene promoter. The results showed that the numbers of common *fibH*, *fibL*, and *P25* PIPs in M4 and L5D5 were 109, 156, and 158, respectively (Fig. 2a and Supplementary Table S2). More than 90% of these PIPs could be annotated using the BlastKOALA web tool of the KEGG database. Intriguingly, a large proportion of the common PIPs were found to be enriched in two primary pathway categories (*i.e.*, metabolism, particularly metabolic pathways, biosynthesis of secondary metabolites, and microbial metabolism in diverse environments; genetic information processing, especially ribosome, proteasome, and protein processing in the endoplasmic reticulum) (Fig. 2b).

**Figure 3:**
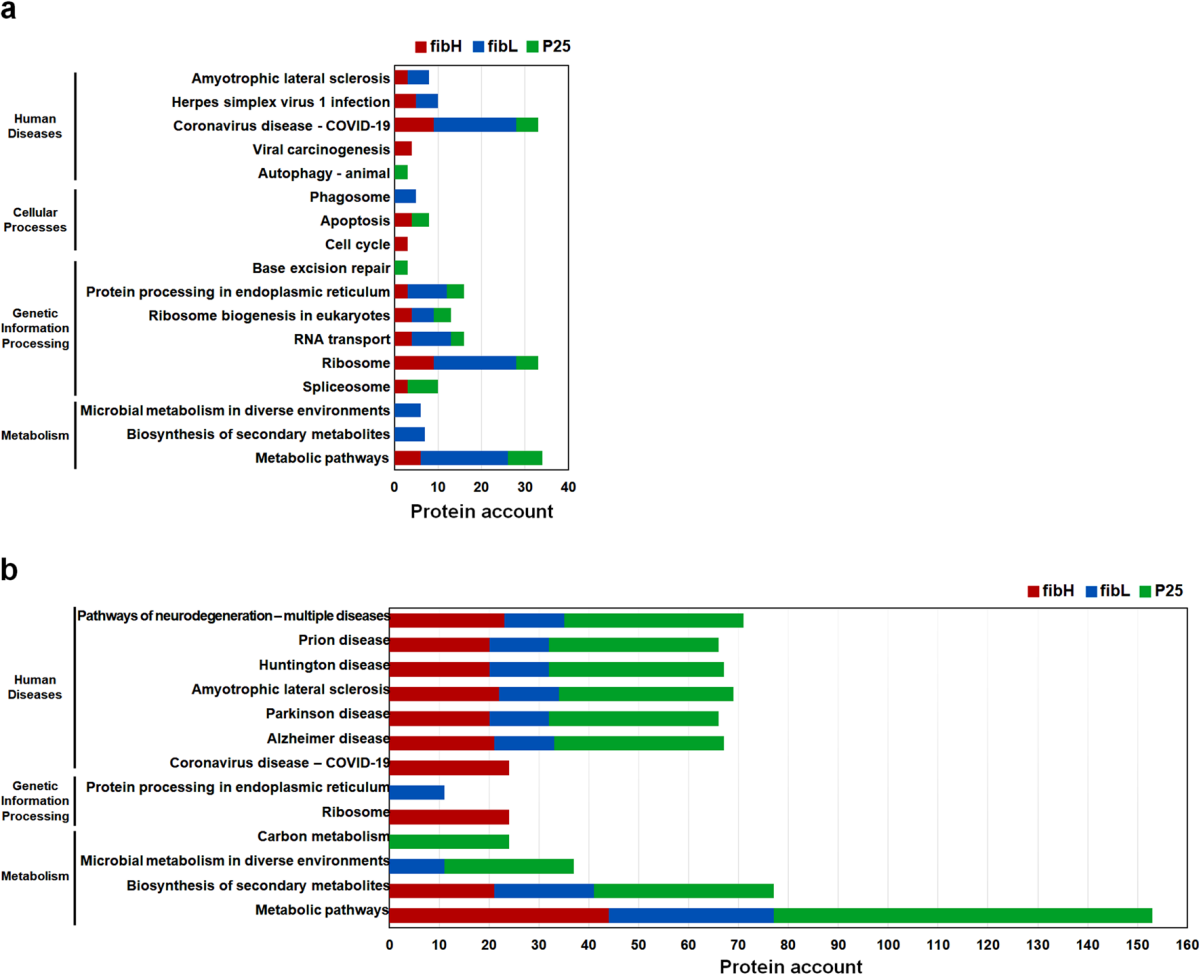
Top 10 KEGG pathways enriched in unique PIPs of each fibroin gene in M4 (**a**) and L5D5 (**b**). Red, blue, and green represent the PIPs of *fibH*, *fibL*, and *P25*, respectively.

Among the unique PIPs (Supplementary Table S3), there were some obvious differences between the fibroin gene promoters and between the two developmental stages. For example, in the M4 PSG, PIPs of *fibH* and *fibL* were enriched in human disease and genetic information processing pathways, especially those related to the ribosome and RNA transport. PIPs of *fibL* were also abundant in metabolism pathways, particularly metabolic pathways, while PIPs of *P25* were abundant only in pathways involved in transcription, translation, folding, sorting and degradation and replication and repair (Fig. 3a). In the L5D5 PSG, the PIPs of the three fibroin genes were most abundant in disease-related pathways, particularly those related to neurodegenerative disease, followed by metabolism-related categories, such as metabolic pathways and the biosynthesis of secondary metabolites. Some PIPs of *fibH* and *fibL* were also enriched in the ribosome and protein processing in the endoplasmic reticulum categories, but no PIPs of *P25* were enriched in these two pathways (Fig. 3b). In addition, there were many more PIPs identified in the L5D5 PSG than in the M4 PSG, which suggests that more proteins are recruited to ensure the efficient transcription of fibroin genes in the L5 stage, leading to the large-scale synthesis of fibroin proteins.

### Identification of common PIPs shared by the three fibroin genes

**Figure 4:**
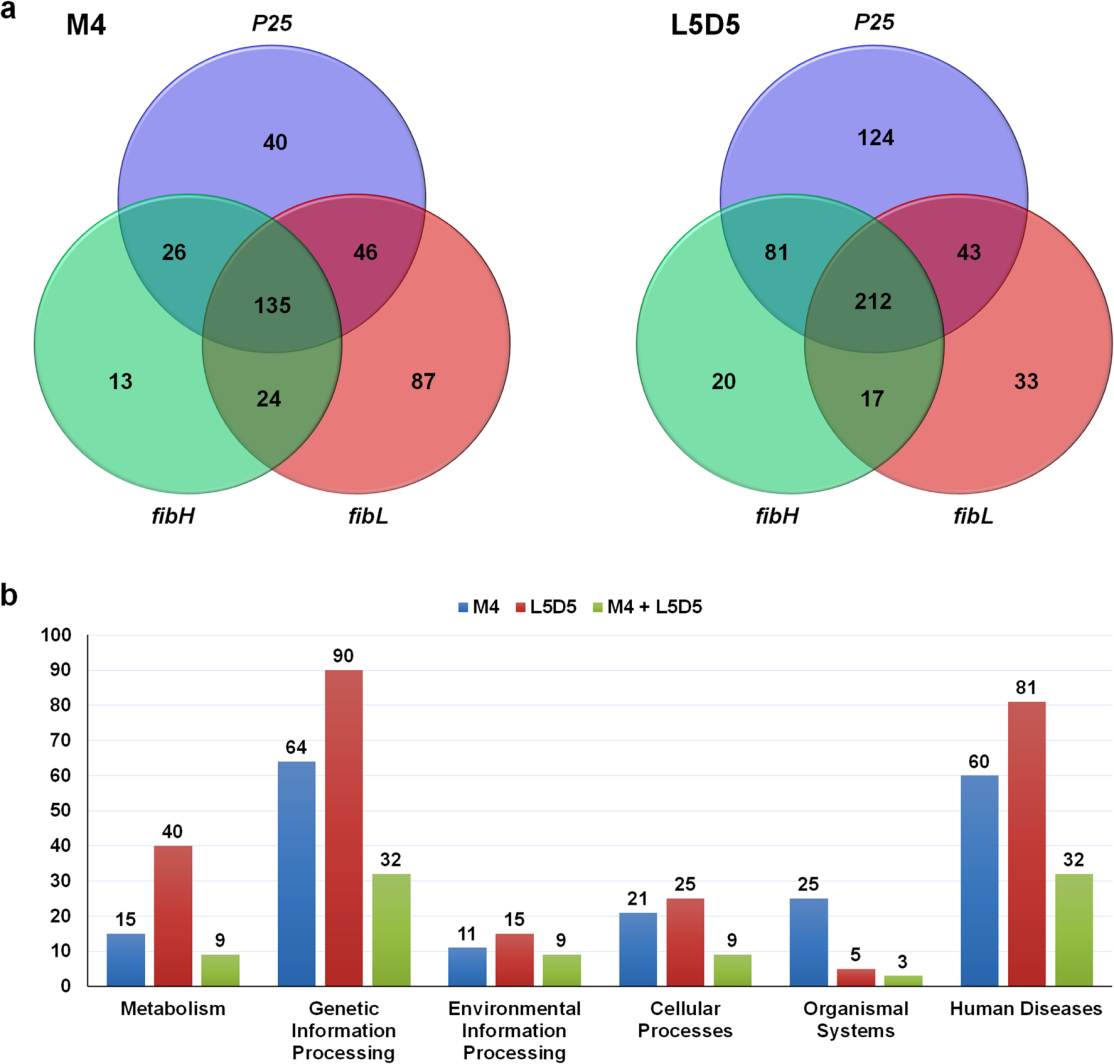
Common PIPs shared by the three fibroin genes. (**a**) Venn diagrams showing the numbers of PIPs interacting with the promoters of *fibH*, *fibL*, and *P25* in either the M4 or L5D5 PSG. (**b**) Distribution of common PIPs in KEGG systems. The numbers on the top of each column are the PIP counts. The green columns represent the PIPs found in both M4 and L5D5 PSGs.

**Figure 5:**
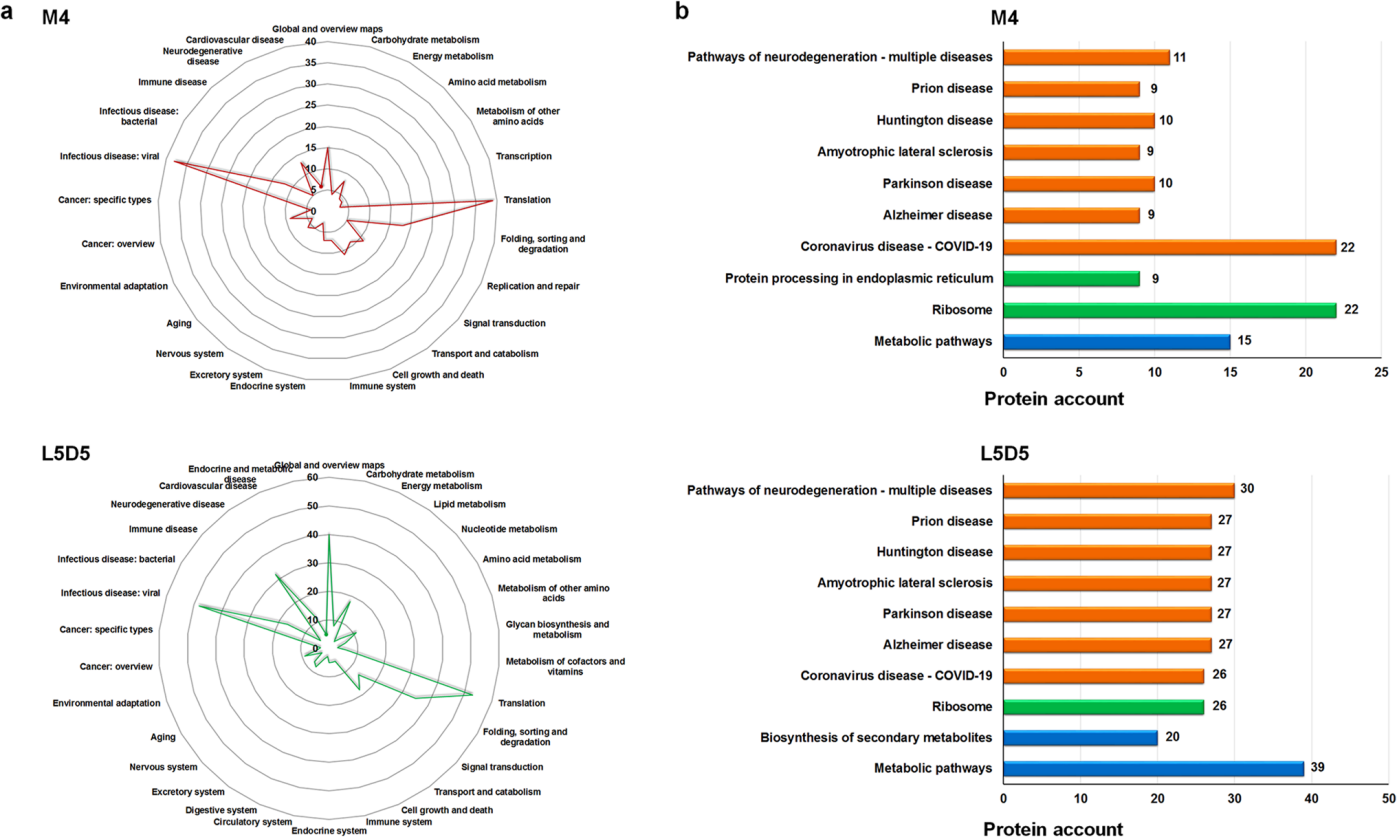
Functional characterization of common PIPs shared by the three fibroin genes. (**a**) KO pathways. (**b**) KO Brite Top 10.

The expression of fibroin genes is regulated mainly at the transcriptional level in a concerted manner^10,11^. To explore the proteins that may be involved in such coregulation, we analyzed the PIPs that interact with the promoters of all three fibroin genes. The results showed that a large number of PIPs (135 in the M4 PSG and 212 in the L5D5 PSG) were shared by *fibH*, *fibL* and *P25* (Fig. 4a and Supplementary Table S4). Functional prediction based on the BlastKOALA web tool of the KEGG database revealed that 90.4% (122/135) and 90.1% (191/212) of the common PIPs received some annotation. These PIPs were annotated to all six KEGG systems, especially genetic information processing, human diseases and metabolism, and some of these PIPs were found in both the M4 and L5D4 PSGs. In addition to organismal systems, there were many more common PIPs in the other five KEGG systems in the L5D5 PSG than in the M4 PSG (Fig. 4b). Further analysis revealed the detailed pathway annotations of those common PIPs identified in M4 and L5D5 (Fig. 5a). The most highly enriched system was human diseases related to neurodegeneration pathways, including the multiple disease, Alzheimer’s disease, and Parkinson’s disease categories, among others. The second most enriched systems were those of common PIPs involved in the ribosome, protein processing in the endoplasmic reticulum, and metabolic pathways (Fig. 5b).

### Functional interaction network of the common PIPs shared by the three fibroin genes

**Figure 6:**
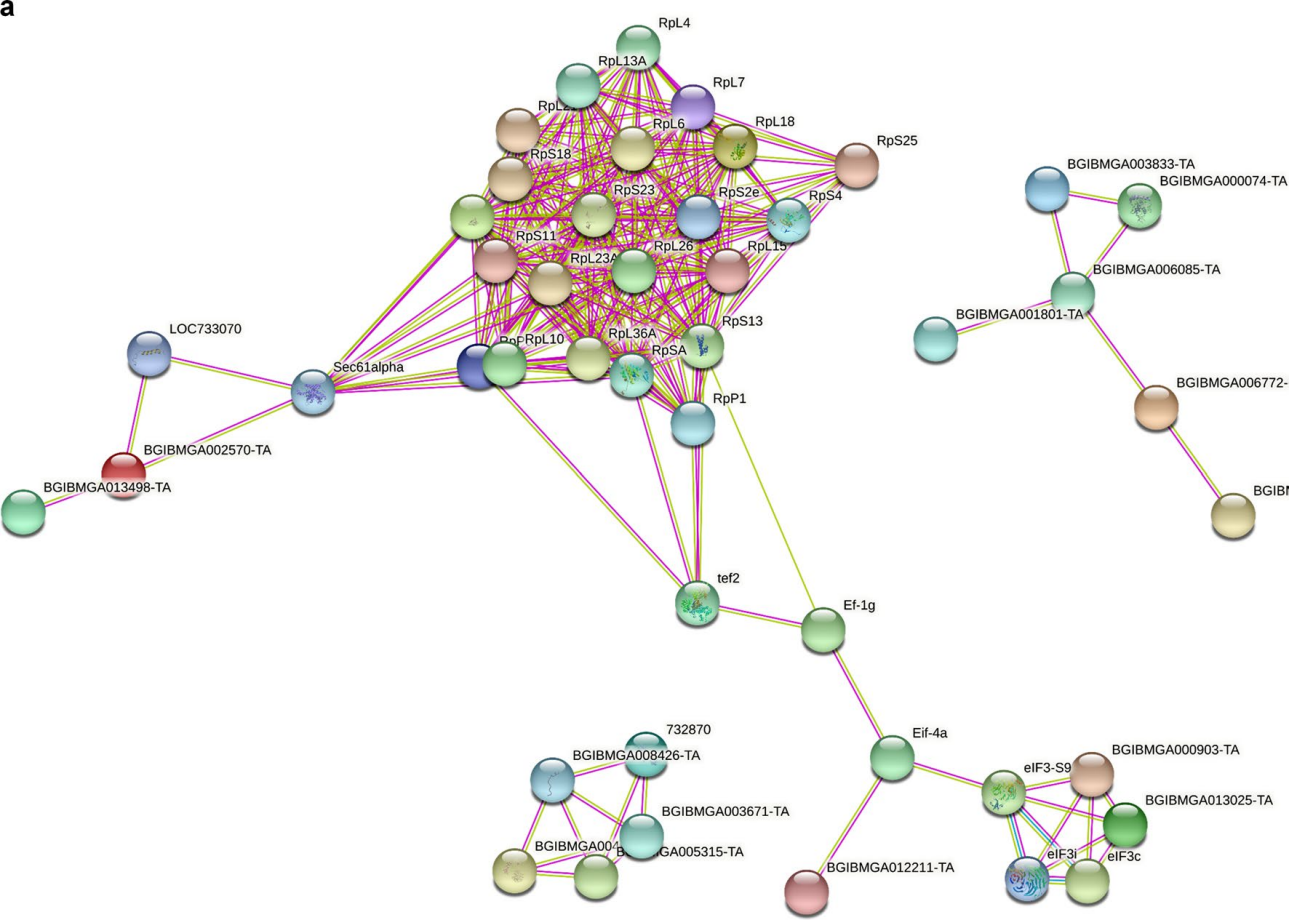

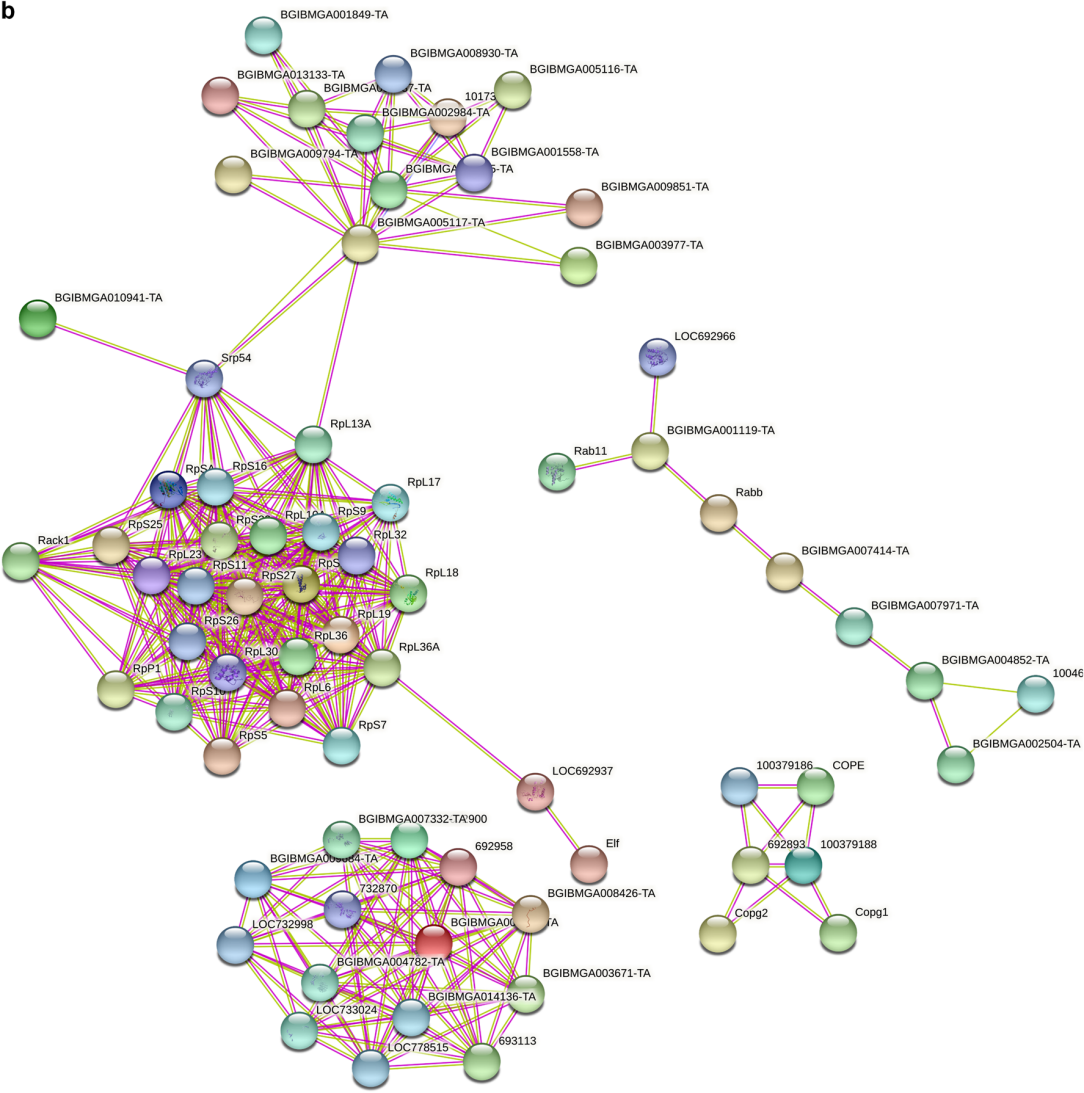
Protein–protein interaction networks among the common PIPs shared by the three fibroin genes. (**a**) The top three significant modules selected from the PPI network generated based on common PIPs from M4. (**b**) The top four significant modules selected from the PPI network generated based on common PIPs from L5D5. Pink or pale green lines represent interaction relationships between the nodes generated based on the available evidence in the STRING database (https://string-db.org/). Minimum required interaction score: highest confidence (0.900); PPI enrichment *p*-value: < 1.0e-16.

To obtain a greater understanding of the possible functional relationships among the common PIPs identified in the M4 and L5D5 PSGs, we further constructed protein–protein interaction (PPI) networks using the online Search Tool for the Retrieval of Interacting Genes/Proteins (STRING, https://string-db.org/). The initial network of the common PIPs in M4 consists of 132 nodes and 254 edges (Fig. 6a and Supplementary Table S5). The vast majority of the nodes were related to 35 PIPs and were mainly involved in ribosome (22 PIPs), RNA transport (5 PIPs), and protein processing in the endoplasmic reticulum (4 PIPs) pathways. The second most significant module (6 PIPs) was involved in ribosome biogenesis in the eukaryotic pathway, and the third most significant module (5 PIPs) was involved in the proteasome pathway. The other modules, which consisted of two or three PIPs, were involved in mismatch repair, aminoacyl-tRNA biosynthesis, DNA replication, nucleotide excision repair, spliceosome, and protein processing in endoplasmic reticulum pathways.

In terms of the common PIPs identified in L5D5, 209 nodes and 444 interactions were found in the initial network (Fig. 6b and Supplementary Table S5). The vast majority of the nodes were related to 44 PIPs and were mainly involved in ribosome (25 PIPs) and aminoacyl-tRNA biosynthesis (12 PIPs) pathways, followed by protein export (2 PIPs) and mRNA surveillance (2 PIPs) pathways. Two significant modules consisting of 14 and 9 PIPs were involved in the proteasome and protein processing in the endoplasmic reticulum, respectively. Another significant module consisted of 6 PIPs that were annotated as subunits of the coatomer complex, a cytosolic protein complex that binds to dilysine motifs and is essential for the retrograde Golgi-to-ER transport of dilysine-tagged proteins. The other modules, consisting of two, three, or four PIPs, were mainly involved in pathways such as protein processing in the endoplasmic reticulum, protein export, and ribosome biogenesis in eukaryotes. Taken together, these results indicate that the common PIPs identified in M4 and L5D5 PSGs show many interactions among themselves and are at least partially biologically connected as a group. Further studies could lead to a better understanding of their roles in the direct regulation of fibroin gene expression during larval molting and feeding stages.

### Description of TFs among the PIPs of fibroin genes

TFs are indispensable for the regulation of fibroin gene transcription and protein synthesis^12–19^. However, only a few TFs from the PSG have been isolated and validated using in vivo/*vitro* methods thus far. Therefore, we further identified the TFs among the PIPs of fibroin genes. As summarized in Table 2, 31 potential TFs were identified, which were distributed on 18 chromosomes. The functions of most of these TFs in regulating fibroin gene expression have not been reported. Notably, 5 TFs that could interact with all fibroin gene promoters were identified in both M4 and L5D5, while the numbers of TFs that could interact with all fibroin gene promoters in either M4 or L5D5 were 11 and 10, respectively. Five TFs were found only in M4, while 9 TFs were found only in L5D5. Surprisingly, 20, 23, and 29 TFs could interact with the promoters of *fibH*, *fibL*, and *P25*, respectively, in either M4 or L5D5. Without considering other factors, these results may reflect the similarities and differences of TFs regulating the expression of the three fibroin genes and are worthy of further study.

**Table 2:** Potential TFs identified from the PIPs of fibroin genes.

Protein ID	Annotation in silkbase	Chromosome	Family/domain	Interacting promoters in M4			Interacting promoters in L5D5		
P_KWMTBOMO00339	Y-box_protein_[Bombyx_mori]	Chr1	CSD	*fibH*		*P25*	*fibH*		*P25*
P_KWMTBOMO00366	PREDICTED:_insulin-like_growth_factor_2_mRNA-binding_protein_1_isoform_X1_[Bombyx_mori]	Chr1	TF_others	*fibH*	*fibL*	*P25*		*fibL*	*P25*
P_KWMTBOMO00497	hypothetical_protein_KGM_14041_[Danaus_plexippus]	Chr1	THAP	*fibH*	*fibL*	*P25*	*fibH*		*P25*
P_KWMTBOMO01796	prohibitin_protein_WPH_[Bombyx_mori]	Chr4	TF_others		*fibL*		*fibH*	*fibL*	*P25*
P_KWMTBOMO03626	PREDICTED:_T-complex_protein_1_subunit_alpha_[Amyelois_transitella]	Chr6	NCU-G1	*fibH*	*fibL*	*P25*	*fibH*	*fibL*	*P25*
P_KWMTBOMO04742	ribosomal_protein_L27_[Bombyx_mori]	Chr8	ETS	*fibH*		*P25*			
P_KWMTBOMO04674	mobility_group_protein_1B_[Bombyx_mori]	Chr8	HMG					*fibL*	*P25*
P_KWMTBOMO04757	PREDICTED:_myosin-IB_isoform_X1_[Papilio_xuthus]	Chr8	MBD						*P25*
P_KWMTBOMO04877	PREDICTED:_myosin_heavy_chain_95F_isoform_X2_[Papilio_polytes]	Chr9	MBD						*P25*
P_KWMTBOMO04966	inorganic_pyrophosphatase_[Bombyx_mori]	Chr9	zf-C2H2					*fibL*	
P_KWMTBOMO05228	cyclophilin-like_protein_[Bombyx_mori]	Chr9	zf-C2H2						*P25*
P_KWMTBOMO05675	ribosomal_protein_L23A_[Bombyx_mori]	Chr10	zf-C2H2	*fibH*	*fibL*	*P25*			
P_KWMTBOMO05699	PREDICTED:_tRNA-splicing_ligase_RtcB_homolog_[Amyelois_transitella]	Chr10	zf-C2H2					*fibL*	
P_KWMTBOMO05810	PREDICTED:_adenylate_kinase_9_isoform_X2_[Amyelois_transitella]	Chr10	Nrf1				*fibH*	*fibL*	*P25*
P_KWMTBOMO07323	chaperonin_subunit_4_delta_[Bombyx_mori]	Chr12	NCU-G1		*fibL*	*P25*	*fibH*	*fibL*	*P25*
P_KWMTBOMO08022	PREDICTED:_HMGB_protein_isoform_X1_[Bombyx_mori]	Chr13	HMG		*fibL*	*P25*			
P_KWMTBOMO08820	abnormal_wing_disc-like_protein_[Bombyx_mori]	Chr15	TF_others	*fibH*	*fibL*	*P25*	*fibH*	*fibL*	*P25*
P_KWMTBOMO09050	Methylcrotonoyl-CoA_carboxylase_subunit_alpha_mitochondrial_[Papilio_xuthus]	Chr15	zf-C2H2	*fibH*	*fibL*		*fibH*		*P25*
P_KWMTBOMO09808	chaperonin_subunit_6a_zeta_[Bombyx_mori]	Chr16	NCU-G1	*fibH*	*fibL*	*P25*	*fibH*	*fibL*	*P25*
P_KWMTBOMO10147	Sr_protein_[Bombyx_mori]	Chr17	TF_others						*P25*
P_KWMTBOMO11061	mitochondrial_prohibitin_complex_protein_2_[Bombyx_mori]	Chr18	TF_others	*fibH*	*fibL*	*P25*	*fibH*	*fibL*	*P25*
P_KWMTBOMO11731	translation_elongation_factor_2_isoform_2_[Bombyx_mori]	Chr19	HMG	*fibH*	*fibL*	*P25*			
P_KWMTBOMO12659	PREDICTED:_T-complex_protein_1_subunit_gamma_[Amyelois_transitella]	Chr21	NCU-G1	*fibH*	*fibL*	*P25*	*fibH*		*P25*
P_KWMTBOMO14887	PREDICTED:_T-complex_protein_1_subunit_eta_isoform_X2_[Papilio_xuthus]	Chr24	NCU-G1		*fibL*	*P25*	*fibH*	*fibL*	*P25*
P_KWMTBOMO15035	PREDICTED:_chlorophyllide_A_binding_protein_isoform_X1_[Bombyx_mori]	Chr25	THAP						*P25*
P_KWMTBOMO15757	uncharacterized_protein_LOC100169462_[Acyrthosiphon_pisum]	Chr26	Homeobox				*fibH*		*P25*
P_KWMTBOMO15758	vacuolar_ATP_synthase_subunit_B_[Bombyx_mori]	Chr26	Homeobox	*fibH*	*fibL*	*P25*	*fibH*	*fibL*	*P25*
P_KWMTBOMO15781	PREDICTED:_C-terminal-binding_protein_isoform_X1_[Bombyx_mori]	Chr26	THAP		*fibL*		*fibH*	*fibL*	*P25*
P_KWMTBOMO15928	poly_A_binding_protein_[Bombyx_mori]	Chr27	bHLH		*fibL*				*P25*
P_KWMTBOMO16040	PREDICTED:_transcriptional_activator_protein_Pur-alpha_isoform_X3_[Bombyx_mori]	Chr27	TF_others		*fibL*	*P25*			*P25*
P_KWMTBOMO16524	Sr_protein_[Bombyx_mori]	Scaf007	TF_others	*fibH*	*fibL*	*P25*		*fibL*	

Combinatorial TF interactions are critical for gene regulation and are important determinants of different cellular functions. To determine possible interactions among the 31 TFs, we constructed corresponding PPI network models using the STRING database. As shown in Supplementary Fig. S5 and Supplementary Table S6, two interaction networks were identified, which consisted of 28 nodes and 16 edges. One module was composed of 2 TFs annotated as prohibitin, which is a highly conserved protein that can inhibit the proliferation and apoptosis of tumor cells by regulating gene transcription and maintaining the stability of mitochondrial proteins. The other module was composed of 9 TFs. Interestingly, two ribosomal proteins (P_KWMTBOMO04742 and P_KWMTBOMO05675) and one translation elongation factor (P_KWMTBOMO11731) that form a subnetwork and interact with each other were found only in the M4 PSG, while adenylate kinase 9 (P_KWMTBOMO05810) was found only in the L5D5 PSG. In addition, another subnetwork was composed of 5 TFs (P_KWMTBOMO07323, P_KWMTBOMO03626, P_KWMTBOMO14887, P_KWMTBOMO09808, and P_KWMTBOMO12659) that interact with each other and are all molecular chaperones with similar functions assisting the folding of proteins upon ATP hydrolysis. Taken together, these results suggest that these TFs are crucial for the correct transcription and folding of silk fibroin proteins and deserve further study.

## Discussion

As a fully domesticated insect, the silkworm, *Bombyx mori*, has attracted much attention from researchers, not only because it can produce silk for human uses but also because of the fascinating mechanisms underlying the precise, concerted regulation of silk protein synthesis at the transcriptional level during larval development. Previous studies have provided some regulatory evidence related to silk protein synthesis, particularly regarding the discovery of several factors, such as TFs, that directly regulate silk protein gene expression. However, it is still difficult to reveal the detailed mechanisms of silk protein synthesis on the basis of the reported regulatory factors.

In an attempt to discover the factors regulating silk protein genes, we mainly focused on the proteins interacting with the promoters of the fibroin genes *fibH*, *fibL*, and *P25*, which encode one of the two component proteins of silk fibers. Candidate factors interacting with the promoters of the three fibroin genes, or PIPs, were obtained from M4 and L5D5 PSGs by DNA pull-down assays and identified using the HPLC–MS method. Our results revealed hundreds of PIPs for each fibroin gene in either the M4 or L5D5 PSG, which preliminarily demonstrates the complexity of the regulation of fibroin gene expression. It is of course possible that some PIPs may have been overlooked in our experiment, partially due to the limitations of the applied technique itself and the use of only one biotin-labeled DNA probe for each fibroin gene. Nevertheless, the obtained data are of great importance for better understanding the regulatory factors that interact with fibroin protein genes.

Whether there is any difference between the proteins interacting with fibroin gene promoters during larval molting and feeding stages is an interesting question. In this work, we selected two representative stages showing significant differences in fibroin protein synthesis in the PSG. M4 is one of the stages during which fibroin protein synthesis is inhibited, while L5D5 is a critical period in which abundant fibroin proteins are synthesized. As a result, 198, 292, and 247 proteins obtained from the M4 PSG and 330, 305, and 460 proteins obtained from the L5D5 PSG were identified as PIPs of *fibH*, *fibL*, and *P25*, respectively. From these data and subsequent KEGG pathway annotation, we found that many of these PIPs had similar functional annotation classifications and were annotated to several different KEGG systems, particularly metabolism, genetic information processing, and human diseases. Interestingly, the number of PIPs in the L5D5 PSG was greater than that observed in the M4 PSG, suggesting that more regulatory proteins may be needed to ensure efficient silk protein synthesis. In addition, there were some obvious differences in the PIPs identified between different fibroin genes, which implies that the cooperative regulation mechanisms of the three fibroin genes may be far more complex than we predicted.

The TFs among the fibroin gene PIPs were key factors upon which we focused because many TFs play vital roles as ‘‘master regulators’’ and ‘‘selector genes’’ of downstream target genes^20^. In *B. mori*, TFs are particularly critical for the spatiotemporal regulation of silk protein synthesis; however, only a few TFs have been identified and confirmed by in vivo or in vitro experiments. In this study, we identified 31 TFs from the M4 and/or L5D5 PSG, which contained conserved domains such as zf-C2H2, bHLH, MYB and THAP domains. Surprisingly, no TFs previously proven to bind to the promoter regions of fibroin genes, including FMBP-1, SGF-2, Bmdimm, POU-M2, and BmFTZ-F1^13–19^, were found among the PIPs of fibroin genes. We speculate that in addition to the limitations of the experimental techniques mentioned above, there is another possibility that is worthy of discussion. TFs do not seem to like to act alone. Most eukaryotic TFs are thought to act by recruiting cofactors and forming protein complexes with them^21^. However, some studies have suggested that the interactions between a TF and its cofactors are weak/transient^12,22^. Therefore, it is possible that some cofactors, rather than TFs, that play master regulatory roles in the PSG of *B. mori*, may have been captured in the present study. In addition, only two periods of PSG development were examined in this study, and it is also possible that some TFs functioning outside these two periods cannot be captured.

In summary, the identification and functional analysis of regulatory factors involved in the regulation of fibroin genes is a complex and arduous task. This study describes the large-scale identification of proteins interacting with the promoter regions of fibroin genes for the first time and provides insights into the regulatory network of fibroin genes. To our knowledge, there is little information on the roles of these DNA-interacting proteins in regulating fibroin genes. Therefore, it would be interesting to investigate the function of these proteins in the PSG, which will improve our better understanding of the regulatory roles of them and elaborating the intricate regulatory network of fibroin protein synthesis.

## Materials and methods

### Animals and sample preparation

The silkworm strain *Nistari* was maintained in our laboratory. The hatched larvae were normally reared on fresh mulberry leaves at 25–26 °C. Fresh mulberry leaves were picked from mulberry trees of the Jialing strain in the campus of Southwest University (Chongqing, China; All procedures were carried out according to the State Key Laboratory of Silkworm Genome Biology Guide). The *Nistari* PSGs used in the DNA pull-down assay were dissected from the M4 and L5D5 larvae. All PSG samples were washed in phosphate-buffered saline (PBS, pH 7.4) and stored at − 80 °C until required.

### Cell culture and luciferase reporter assay

Cultured *B. mori* embryonic cells (BmE) maintained at 27 °C in Grace’s insect medium containing 10% fetal bovine serum (HyClone, China) were used to examine the promoter activities of fibroin genes. In brief, the promoter sequences of the fibroin genes *fibH* (AF226688.1)*, fibL* (AAA27840.1) and *P25* (X04226.1), with lengths of 2096 bp, 1060 bp and 1227 bp, respectively, were commercially synthesized (GenScript, China) and inserted into the empty pGL4.11[*Luc2P*] vector (Promega, USA). The abbreviations for the final constructs were fibH-Luc2P, fibL-Luc2P, and P25-Luc2P, respectively. Then, a mixture of 3.5 μL of transfection reagents (Roche, USA) with 1.5 μg of the fibH-Luc2P, fibL-Luc2P, or P25-Luc2P plasmid DNA was transfected into BmE cells. After 36 h of culture at 27 °C, the cells were harvested, and luciferase activity was determined using a Dual Luciferase Reporter Gene Assay Kit (Yeasen, China). pGL4.11[*Luc2P*] was used as a negative control. The plasmids p57S[hrA4-*Gal4*] (A4G4) and pGL4.11[UAS-*Luc2P*] (UAS-Luc2P), which we constructed based on a previously modified Gal4/UAS binary system^23,24^, were used as positive controls.

### DNA pull-down assay

First, the plasmids fibH-Luc2P, fibL-Luc2P, and P25-Luc2P were used as templates, and the promoter regions of *fibH, fibL* and *P25* were amplified by PCR using 5’-biotin-labeled forward primers (Supplementary Table S7). Each of the 5’-biotinylated DNA sequences was immobilized on streptavidin beads following the manufacturer’s protocol (Dynabeads MyOne Streptavidin C1, Invitrogen, USA). Then, PSG proteins in the nuclear fraction were incubated with 5’-biotinylated DNA beads on a rotating shaker at 4 °C overnight. Following this incubation, the supernatant was removed, and the beads were washed three times with RIP wash buffer (Fitgene, China). After the last wash, the pull-down mixture was resuspended in elution buffer on a rotating shaker at room temperature for 15 min to break the bonds between streptavidin and biotin. Finally, the proteins eluted from the beads were subjected to polyacrylamide gel electrophoresis (PAGE) followed by silver nitrate staining and MS analysis. The proteins eluted from the beads with the unbiotinylated DNA probe and GFP probe were used as controls.

### HPLC–MS analysis

Peptides were dissolved in 0.1% formic acid (FA, Sigma, USA) and 2% acetonitrile (ACN, Fisher, Germany) and directly loaded onto a reversed-phase analytical column (75 μm i.d. × 150 mm, packed with Acclaim PepMap RSLC C18, 2 μm, 100 Å, nanoViper). The applied gradient was as follows: increase from 5 to 50% solvent B (0.1% FA in 80% ACN) over 40 min, followed by an increase to 90% B over 5 min, and then holding at 90% B for 5 min, all at a constant flow rate of 300 nL/min.

MS analysis was performed on a Q Exactive hybrid quadrupole-Orbitrap mass spectrometer (Thermo Fisher Scientific). The peptides were subjected to a nanospray ionization (NSI) source, followed by tandem mass spectrometry (MS/MS) in a Q Exactive system (Thermo Fisher Scientific) coupled online to an ultraperformance liquid chromatography (UPLC) system. Intact peptides were detected in the Orbitrap at a resolution of 70,000. Peptides were selected for MS/MS using a normalized collision energy (NCE) setting of 27; ion fragments were detected in the Orbitrap at a resolution of 17,500. A data-dependent procedure that alternated between one MS scan and 20 subsequent MS/MS scans was applied for the top 20 precursor ions above a threshold ion count of 1E4 in the MS survey scan with 30.0 s dynamic exclusion. The applied electrospray voltage was 2.0 kV. Automatic gain control (AGC) was used to prevent the overfilling of the ion trap; 1E5 ions were accumulated for the generation of MS/MS spectra. For MS scans, the m/z scan range was 350 to 1800 m/z. The fixed first mass was set as 100 m/z.

### Data analysis

Peptide and protein identification were performed with ProteinPilot Software 4.5 (version 1656, AB SCIEX, USA; https://sciex.com/products/software/proteinpilot-software) by searching SilkBase (http://silkbase.ab.a.u-tokyo.ac.jp). The functional annotation of the PIPs was conducted with KEGG BlastKoala^25^ by selecting “Taxonomy group: Eukaryotes”, setting the KEGG database search parameters to “family_eukaryotes + genus_prokaryotes” and setting other parameters to the default values. The proteins identified by the pull-down assays were aligned in the AnimalTFDB3.0 website (http://bioinfo.life.hust.edu.cn/AnimalTFDB2/index.shtml) using the following filtration parameters to predict the candidate TFs: identity (%) ≥ 30 and coverage (%) ≥ 50. The STRING database (https://string-db.org/) and web tool^26^ were used to predict the protein–protein interaction networks.

## Supplementary Information

Supplementary Information.
