# Supplementary material for: New insights into the proteins interacting with the promoters of silkworm fibroin genes
Source: Sci Rep. 2021 Aug 5;11:15880. doi: 10.1038/s41598-021-95400-0 (PMC8342599; doi:10.1038/s41598-021-95400-0)
Supplement: Supplementary file 1 — Supplementary Information. [file 41598_2021_95400_MOESM1_ESM.pdf]

Supplementary information

New insights into the proteins interacting with the promoters of silkworm fibroin genes

Yan Ma[1†], Qin Luo[1†], Yao Ou[1], Yiyun Tang[1], Wenhui Zeng[1], Haomiao Wang[1], Jie Hu[1], Hanfu Xu[1*]

[1]State Key Laboratory of Silkworm Genome Biology, College of Sericulture, Textile and Biomass Sciences, Southwest University, Chongqing, 400715, China

*Corresponding. xuhf@swu.edu.cn

[†]These authors contributed equally to this work.

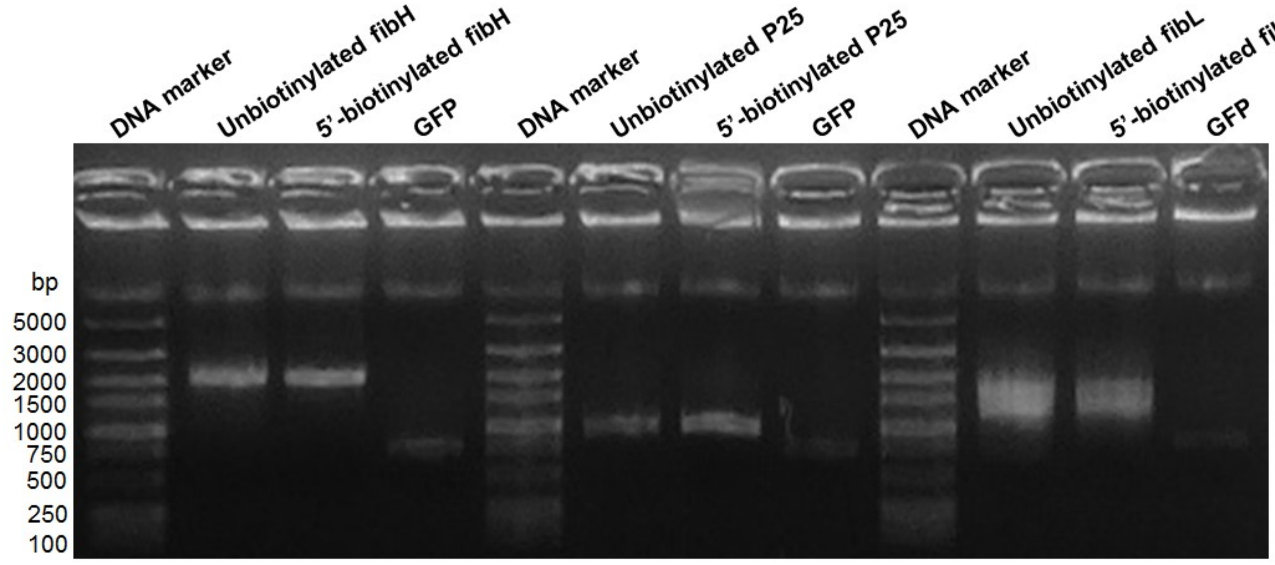

Supplementary Figure S1. Agarose gel electrophoresis of 5'-biotinylated and unbiotinylated DNA probes.

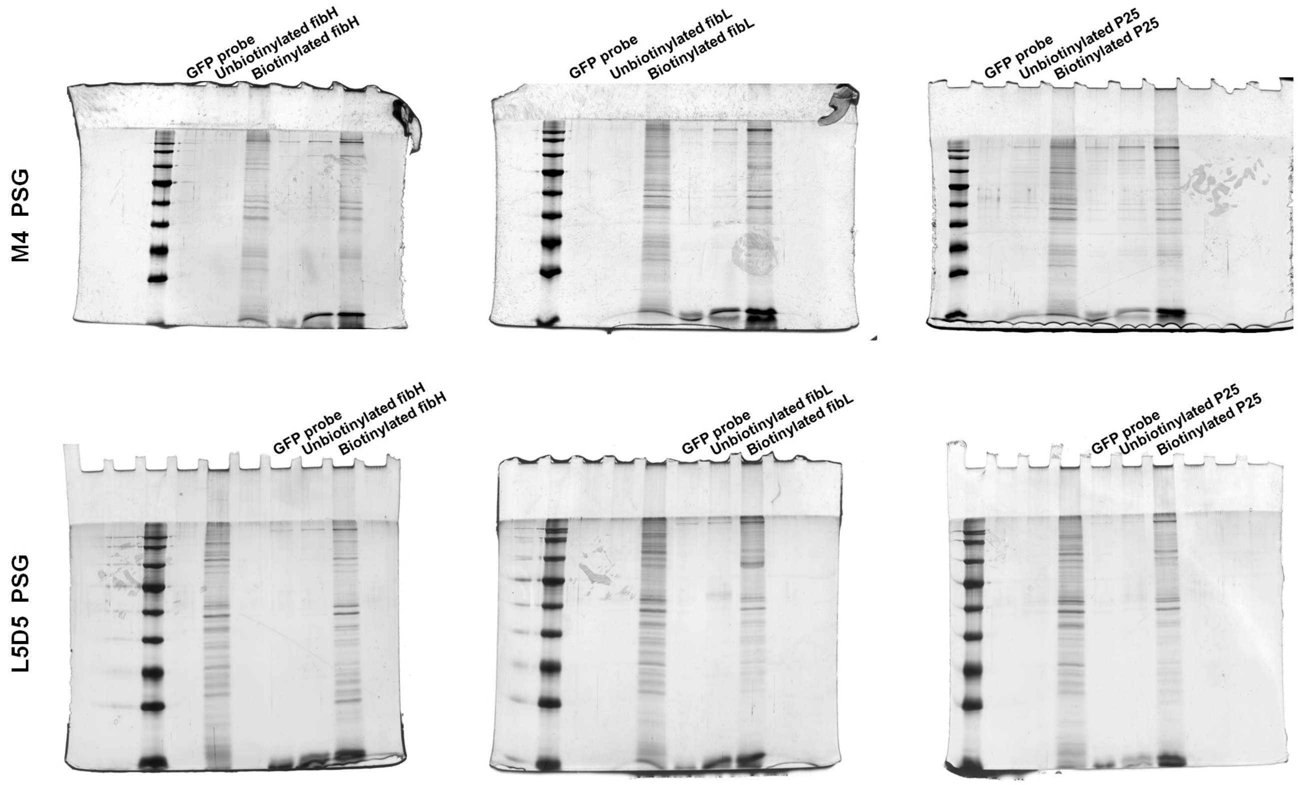

Supplementary Figure S2. SDS-PAGE analysis of the potential PIPs in PSG nuclear protein extracts pulled down using the probes.

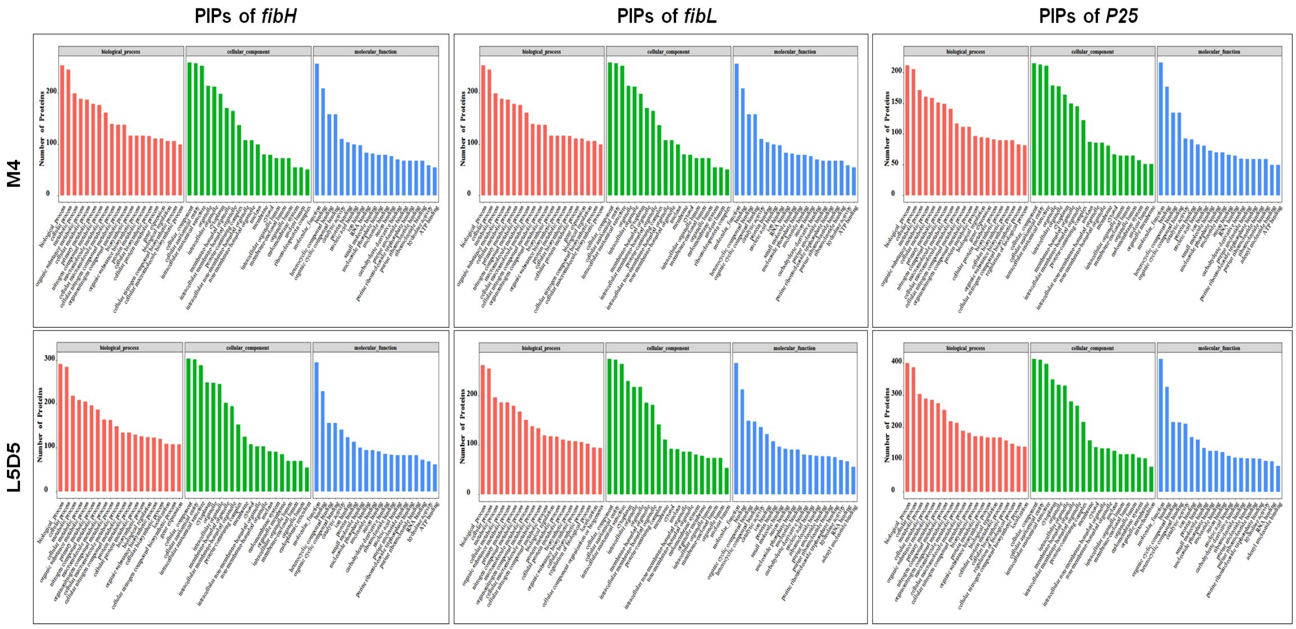

Supplementary Figure S3. GO annotation of candidate PIPs identified by HPLC-MS.

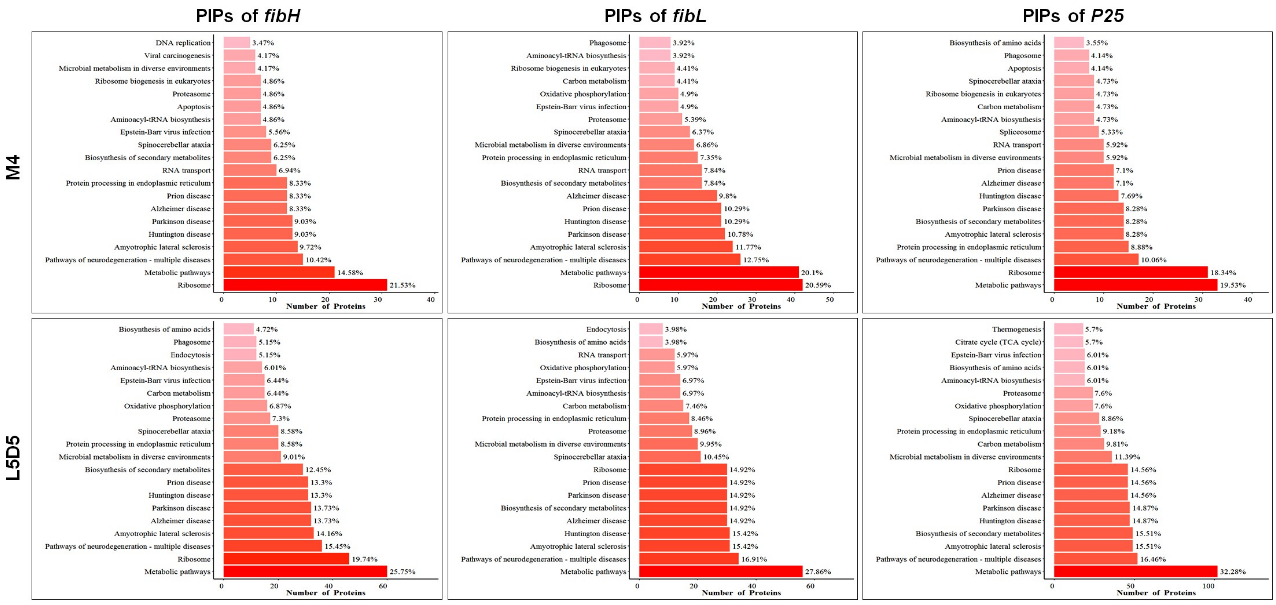

Supplementary Figure S4. KEGG pathway annotation of candidate PIPs identified by HPLC-MS.

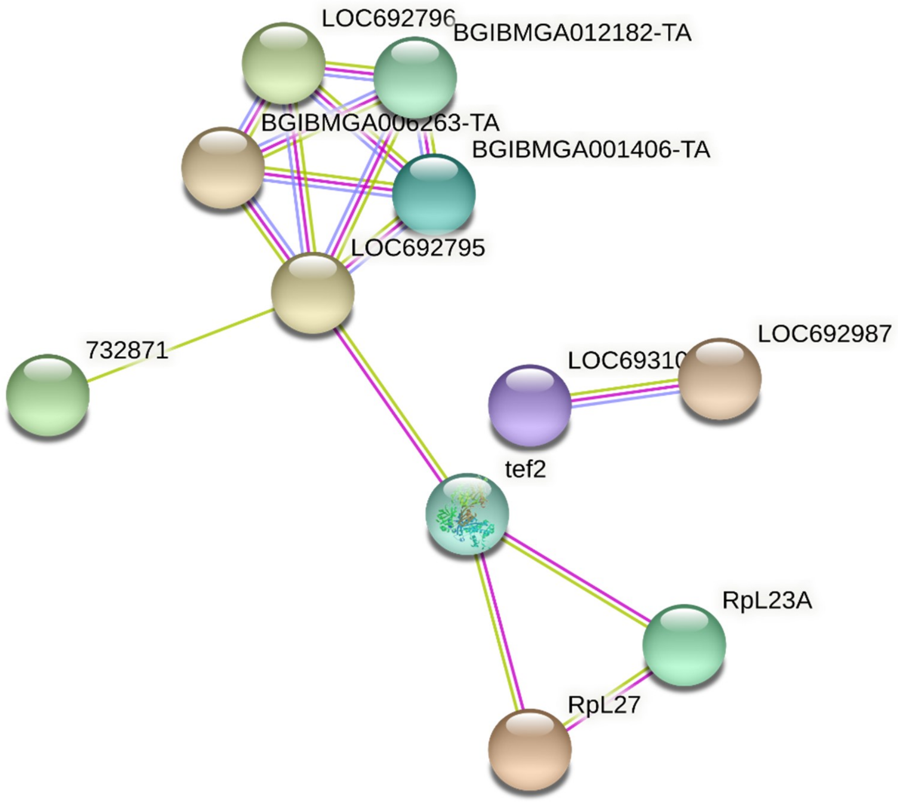

Supplementary Figure S5. Protein-protein interaction networks among the TFs interacting with fibroin gene promoters. Colored lines represent interaction relationships between the nodes generated based on the available evidence in the STRING database (https://string-db.org/). Minimum required interaction score: high confidence (0.700); PPI enrichment *p*-value: 1.71e-08.

Supplementary Table S1. Summary of the identified PIPs of fibroin genes.

Supplementary Table S2. Common PIPs of each fibroin gene in the M4 and L5D5 PSGs.

Supplementary Table S3. Unique PIPs of each fibroin gene in the M4 and L5D5 PSGs.

Supplementary Table S4. Common PIPs shared by the three fibroin genes.

Supplementary Table S5. List of PIPs associated with Figure 6.

Supplementary Table S6. List of TFs associated with Figure S4.

Supplementary Table S7. Primer sequences used in this study.

Supplementary Table S1. Summary of the identified PIPs of fibroin genes.

Summary of the identified PIPs of fibH_M4

N	Unused	Total	%Cov	%Cov(50)	%Cov(95)	Accession	Citable Accession	Name	Peptides(95%)
5	20.56	20.56	10.2	6.3550003	5.0170001	P_KWMTBOMO08648	P_KWMTBOMO08648	P_KWMTBOMO08648	9
6	19.37	19.37	25.34	22.750001	18.170001	P_KWMTBOMO08642	P_KWMTBOMO08642	P_KWMTBOMO08642	12
7	16.7	16.7	18.13	13.98	9.0049997	P_KWMTBOMO11731	P_KWMTBOMO11731	P_KWMTBOMO11731	7
8	16.55	16.55	34.259999	26.530001	24.75	P_KWMTBOMO07374	P_KWMTBOMO07374	P_KWMTBOMO07374	12
13	13.69	13.69	8.4770001	6.4970002	3.4010001	P_KWMTBOMO10056	P_KWMTBOMO10056	P_KWMTBOMO10056	5
16	12.04	12.04	36.649999	25.569999	22.73	P_KWMTBOMO05675	P_KWMTBOMO05675	P_KWMTBOMO05675	7
17	11.3	11.3	8.5430004	8.5430004	8.5430004	P_KWMTBOMO15159	P_KWMTBOMO15159	P_KWMTBOMO15159	6
18	11.26	11.26	14.41	9.0089999	6.7570001	P_KWMTBOMO00462	P_KWMTBOMO00462	P_KWMTBOMO00462	5
19	11.03	11.03	4.786	3.5209998	3.2049999	P_KWMTBOMO06632	P_KWMTBOMO06632	P_KWMTBOMO06632	6
21	10.28	10.28	29.74	21.19	10.78	P_KWMTBOMO11985	P_KWMTBOMO11985	P_KWMTBOMO11985	6
22	10.27	10.27	11.5	7.9000004	6.8000004	P_KWMTBOMO13293	P_KWMTBOMO13293	P_KWMTBOMO13293	5
24	10.13	10.13	15.48	13.3	11.48	P_KWMTBOMO00927	P_KWMTBOMO00927	P_KWMTBOMO00927	5
26	9.71	9.71	10.71	10.71	8.6130001	P_KWMTBOMO12895	P_KWMTBOMO12895	P_KWMTBOMO12895	6
28	9.58	9.58	8.997	6.1009999	6.1009999	P_KWMTBOMO01927	P_KWMTBOMO01927	P_KWMTBOMO01927	7
29	9.08	9.08	20.909999	16.35	16.35	P_KWMTBOMO13455	P_KWMTBOMO13455	P_KWMTBOMO13455	5
31	8.92	8.92	10.87	10.87	10.87	P_KWMTBOMO04502	P_KWMTBOMO04502	P_KWMTBOMO04502	5
32	8.68	8.68	40.849999	35.92	26.76	P_KWMTBOMO07373	P_KWMTBOMO07373	P_KWMTBOMO07373	6
36	8.32	8.32	6.5640002	6.5640002	5.6949999	P_KWMTBOMO11517	P_KWMTBOMO11517	P_KWMTBOMO11517	4
37	8.3	8.3	23.74	17.900001	17.51	P_KWMTBOMO08983	P_KWMTBOMO08983	P_KWMTBOMO08983	6
38	8.07	8.07	16.56	11.18	8.3870001	P_KWMTBOMO05564	P_KWMTBOMO05564	P_KWMTBOMO05564	4
41	7.48	7.48	10.69	6.5009996	2.908	P_KWMTBOMO04505	P_KWMTBOMO04505	P_KWMTBOMO04505	3
43	7.34	7.34	12.48	8.5900001	7.2930001	P_KWMTBOMO11096	P_KWMTBOMO11096	P_KWMTBOMO11096	4
44	6.88	6.88	23.62	15.6	11.7	P_KWMTBOMO09591	P_KWMTBOMO09591	P_KWMTBOMO09591	4
45	6.78	6.78	20.88	19.78	11.72	P_KWMTBOMO09442	P_KWMTBOMO09442	P_KWMTBOMO09442	3
46	6.7	6.7	18.92	11.5	10.02	P_KWMTBOMO01075	P_KWMTBOMO01075	P_KWMTBOMO01075	3
48	6.51	6.51	7.7990003	7.7990003	6.6849999	P_KWMTBOMO10358	P_KWMTBOMO10358	P_KWMTBOMO10358	3
49	6.38	6.38	9.9079996	8.2570001	6.972	P_KWMTBOMO13721	P_KWMTBOMO13721	P_KWMTBOMO13721	3
50	6.24	6.24	50	50	35.29	P_KWMTBOMO13569	P_KWMTBOMO13569	P_KWMTBOMO13569	3
51	6.2	6.2	19	15.67	10.67	P_KWMTBOMO14732	P_KWMTBOMO14732	P_KWMTBOMO14732	3
52	6.05	6.05	25.690001	15.019999	9.8810002	P_KWMTBOMO12497	P_KWMTBOMO12497	P_KWMTBOMO12497	3
53	6.01	6.01	22.47	13.92	13.92	P_KWMTBOMO09300	P_KWMTBOMO09300	P_KWMTBOMO09300	4
54	6	6	7.5029999	4.5019999	4.5019999	P_KWMTBOMO03177	P_KWMTBOMO03177	P_KWMTBOMO03177	3
55	6	6	23.810001	23.810001	23.810001	P_KWMTBOMO10509	P_KWMTBOMO10509	P_KWMTBOMO10509	5
56	6	6	11.31	11.31	11.31	P_KWMTBOMO00497	P_KWMTBOMO00497	P_KWMTBOMO00497	3
57	5.92	5.92	24.2	20.550001	9.589	P_KWMTBOMO05979	P_KWMTBOMO05979	P_KWMTBOMO05979	2
58	5.9	5.9	8.2879998	7.3870003	7.3870003	P_KWMTBOMO03626	P_KWMTBOMO03626	P_KWMTBOMO03626	4
59	5.89	5.89	12.42	5.28	5.28	P_KWMTBOMO00687	P_KWMTBOMO00687	P_KWMTBOMO00687	3
60	5.84	5.84	20	12.890001	8.9469999	P_KWMTBOMO01720	P_KWMTBOMO01720	P_KWMTBOMO01720	3
62	5.74	5.74	15.019999	15.019999	15.019999	P_KWMTBOMO05566	P_KWMTBOMO05566	P_KWMTBOMO05566	3
63	5.64	5.64	9.2859998	9.2859998	9.2859998	P_KWMTBOMO02081	P_KWMTBOMO02081	P_KWMTBOMO02081	3
64	5.54	5.54	5.6030001	4.7499999	4.7499999	P_KWMTBOMO00638	P_KWMTBOMO00638	P_KWMTBOMO00638	3
65	5.43	5.43	20.62	13.4	7.7320002	P_KWMTBOMO11255	P_KWMTBOMO11255	P_KWMTBOMO11255	2
66	5.3	5.3	6.8240002	6.8240002	6.8240002	P_KWMTBOMO09415	P_KWMTBOMO09415	P_KWMTBOMO09415	3
67	5.19	5.19	27.320001	24.590001	11.48	P_KWMTBOMO06480	P_KWMTBOMO06480	P_KWMTBOMO06480	2

68	5.16	5.16	19.39	8.5110001	8.5110001	P_KWMTBOMO11083	P_KWMTBOMO11083	P_KWMTBOMO11083	3
69	5.12	5.12	3.444	2.4970001	1.636	P_KWMTBOMO11045	P_KWMTBOMO11045	P_KWMTBOMO11045	3
70	5.09	5.09	9.6510001	9.6510001	9.6510001	P_KWMTBOMO06231	P_KWMTBOMO06231	P_KWMTBOMO06231	3
71	4.97	4.97	13.13	7.5049996	5.6290001	P_KWMTBOMO06770	P_KWMTBOMO06770	P_KWMTBOMO06770	3
72	4.65	4.65	20.280001	8.986	4.1469999	P_KWMTBOMO06229	P_KWMTBOMO06229	P_KWMTBOMO06229	2
73	4.57	4.57	34.529999	28.780001	15.83	P_KWMTBOMO08399	P_KWMTBOMO08399	P_KWMTBOMO08399	2
74	4.48	4.48	15.629999	8.8509999	3.5780001	P_KWMTBOMO09808	P_KWMTBOMO09808	P_KWMTBOMO09808	2
75	4.29	4.29	4.623	3.015	2.211	P_KWMTBOMO10780	P_KWMTBOMO10780	P_KWMTBOMO10780	2
76	4.19	4.19	9.646	6.4309999	4.3409999	P_KWMTBOMO03748	P_KWMTBOMO03748	P_KWMTBOMO03748	2
77	4.07	4.07	8.6630002	5.2409999	2.781	P_KWMTBOMO14420	P_KWMTBOMO14420	P_KWMTBOMO14420	2
79	4.02	4.38	13.74	11.83	8.7789997	P_KWMTBOMO09980	P_KWMTBOMO09980	P_KWMTBOMO09980	2
80	4.02	4.02	6.786	2.595	2.595	P_KWMTBOMO05541	P_KWMTBOMO05541	P_KWMTBOMO05541	2
81	4.02	4.02	4.972	3.125	3.125	P_KWMTBOMO08889	P_KWMTBOMO08889	P_KWMTBOMO08889	3
82	4.01	4.01	6.3490003	4.7619998	4.7619998	P_KWMTBOMO02748	P_KWMTBOMO02748	P_KWMTBOMO02748	2
83	4	4	3.046	1.668	1.668	P_KWMTBOMO14858	P_KWMTBOMO14858	P_KWMTBOMO14858	2
84	4	4	21.55	15.47	15.47	P_KWMTBOMO16637	P_KWMTBOMO16637	P_KWMTBOMO16637	2
85	4	4	7.7100001	4.9070001	4.9070001	P_KWMTBOMO14984	P_KWMTBOMO14984	P_KWMTBOMO14984	3
86	4	4	10.56	7.9180002	7.9180002	P_KWMTBOMO13997	P_KWMTBOMO13997	P_KWMTBOMO13997	2
87	4	4	8.0399998	5.779	5.779	P_KWMTBOMO12166	P_KWMTBOMO12166	P_KWMTBOMO12166	2
88	4	4	10.63	6.5399997	6.5399997	P_KWMTBOMO04370	P_KWMTBOMO04370	P_KWMTBOMO04370	2
89	4	4	10.49	7.6920003	7.6920003	P_KWMTBOMO03860	P_KWMTBOMO03860	P_KWMTBOMO03860	2
90	4	4	6.6239998	5.556	5.556	P_KWMTBOMO02470	P_KWMTBOMO02470	P_KWMTBOMO02470	2
91	4	4	7.1560003	5.6499999	5.6499999	P_KWMTBOMO00366	P_KWMTBOMO00366	P_KWMTBOMO00366	2
92	4	4	7.6920003	7.6920003	7.6920003	P_KWMTBOMO11061	P_KWMTBOMO11061	P_KWMTBOMO11061	2
93	4	4	9.4769999	9.4769999	9.4769999	P_KWMTBOMO10248	P_KWMTBOMO10248	P_KWMTBOMO10248	2
95	3.85	3.85	2.228	1.733	1.188	P_KWMTBOMO11868	P_KWMTBOMO11868	P_KWMTBOMO11868	2
96	3.85	3.85	5.2510001	3.1959999	3.1959999	P_KWMTBOMO09621	P_KWMTBOMO09621	P_KWMTBOMO09621	2
97	3.85	3.85	3.2600001	2.397	2.397	P_KWMTBOMO04806	P_KWMTBOMO04806	P_KWMTBOMO04806	2
98	3.77	3.77	12.05	7.4699998	6.024	P_KWMTBOMO13979	P_KWMTBOMO13979	P_KWMTBOMO13979	2
99	3.74	3.74	4.7460001	1.78	1.78	P_KWMTBOMO09542	P_KWMTBOMO09542	P_KWMTBOMO09542	2
100	3.72	3.72	25.130001	12.31	12.31	P_KWMTBOMO01320	P_KWMTBOMO01320	P_KWMTBOMO01320	2
101	3.68	3.68	21.65	21.65	21.65	P_KWMTBOMO01918	P_KWMTBOMO01918	P_KWMTBOMO01918	2
102	3.66	3.66	10.19	7.9620004	7.9620004	P_KWMTBOMO15758	P_KWMTBOMO15758	P_KWMTBOMO15758	2
103	3.52	3.52	7.7940002	4.5589998	2.9410001	P_KWMTBOMO08652	P_KWMTBOMO08652	P_KWMTBOMO08652	2
104	3.51	3.52	10.8	6.103	6.103	P_KWMTBOMO00437	P_KWMTBOMO00437	P_KWMTBOMO00437	2
105	3.51	3.51	5.847	3.2219999	3.2219999	P_KWMTBOMO08213	P_KWMTBOMO08213	P_KWMTBOMO08213	2
106	3.5	3.5	6.329	3.376	2.743	P_KWMTBOMO08343	P_KWMTBOMO08343	P_KWMTBOMO08343	2
107	3.4	3.4	10.23	5.8460001	5.8460001	P_KWMTBOMO08956	P_KWMTBOMO08956	P_KWMTBOMO08956	2
108	3.35	3.55	10.26	10.26	7.051	P_KWMTBOMO16357	P_KWMTBOMO16357	P_KWMTBOMO16357	2
109	3.3	3.3	4.834	3.7289999	3.7289999	P_KWMTBOMO15359	P_KWMTBOMO15359	P_KWMTBOMO15359	2
110	3.29	3.29	6.3560002	4.0959999	4.0959999	P_KWMTBOMO09050	P_KWMTBOMO09050	P_KWMTBOMO09050	2
111	3.19	3.19	29.269999	19.509999	19.509999	P_KWMTBOMO12287	P_KWMTBOMO12287	P_KWMTBOMO12287	2
112	3.18	3.18	8.8409998	3.768	3.768	P_KWMTBOMO14549	P_KWMTBOMO14549	P_KWMTBOMO14549	2
114	3.14	3.14	11.15	3.7810002	3.7810002	P_KWMTBOMO01606	P_KWMTBOMO01606	P_KWMTBOMO01606	2
115	3.14	3.14	2.4390001	1.368	1.368	P_KWMTBOMO09747	P_KWMTBOMO09747	P_KWMTBOMO09747	3
116	3.07	3.07	4.2640001	4.2640001	4.2640001	P_KWMTBOMO14752	P_KWMTBOMO14752	P_KWMTBOMO14752	2
117	2.96	2.96	11.99	4.6130002	1.8449999	P_KWMTBOMO00793	P_KWMTBOMO00793	P_KWMTBOMO00793	1
118	2.94	2.94	14.02	7.9269998	7.9269998	P_KWMTBOMO06082	P_KWMTBOMO06082	P_KWMTBOMO06082	2

119	2.94	2.94	20.900001	12.69	5.9700001	P_KWMTBOMO04742	P_KWMTBOMO04742	P_KWMTBOMO04742	1
120	2.89	2.89	7.4819997	2.734	1.439	P_KWMTBOMO10493	P_KWMTBOMO10493	P_KWMTBOMO10493	1
121	2.86	2.86	7.6480001	6.061	2.3089999	P_KWMTBOMO13567	P_KWMTBOMO13567	P_KWMTBOMO13567	1
122	2.83	3.62	4.9619999	4.9619999	1.718	P_KWMTBOMO12560	P_KWMTBOMO12560	P_KWMTBOMO12560	1
123	2.79	2.79	4.5699999	4.205	4.205	P_KWMTBOMO07294	P_KWMTBOMO07294	P_KWMTBOMO07294	2
124	2.76	2.76	22.55	13.240001	3.9220002	P_KWMTBOMO01273	P_KWMTBOMO01273	P_KWMTBOMO01273	1
126	2.63	2.64	9.7929999	5.6499999	2.448	P_KWMTBOMO02994	P_KWMTBOMO02994	P_KWMTBOMO02994	1
127	2.59	2.59	24.05	15.82	8.2280003	P_KWMTBOMO14479	P_KWMTBOMO14479	P_KWMTBOMO14479	1
128	2.45	2.45	6.3189998	3.3179998	1.7379999	P_KWMTBOMO06803	P_KWMTBOMO06803	P_KWMTBOMO06803	1
129	2.42	3.15	6.9140002	3.827	1.481	P_KWMTBOMO12844	P_KWMTBOMO12844	P_KWMTBOMO12844	1
130	2.42	2.42	8.4420003	8.4420003	8.4420003	P_KWMTBOMO16524	P_KWMTBOMO16524	P_KWMTBOMO16524	2
132	2.31	2.31	17.62	13.410001	4.2149998	P_KWMTBOMO07688	P_KWMTBOMO07688	P_KWMTBOMO07688	1
133	2.27	2.27	16.779999	12.59	12.59	P_KWMTBOMO08666	P_KWMTBOMO08666	P_KWMTBOMO08666	2
134	2.23	2.24	36.970001	19.329999	8.4030002	P_KWMTBOMO15114	P_KWMTBOMO15114	P_KWMTBOMO15114	1
135	2.21	2.21	2.128	0.3676	0.2515	P_KWMTBOMO08740	P_KWMTBOMO08740	P_KWMTBOMO08740	1
136	2.2	2.2	21.94	13.55	8.3870001	P_KWMTBOMO05068	P_KWMTBOMO05068	P_KWMTBOMO05068	2
137	2.19	2.19	5.7470001	5.7470001	3.218	P_KWMTBOMO09054	P_KWMTBOMO09054	P_KWMTBOMO09054	1
138	2.18	2.18	5.6090001	3.5599999	1.079	P_KWMTBOMO04610	P_KWMTBOMO04610	P_KWMTBOMO04610	1
139	2.17	2.18	12.69	7.3080003	7.3080003	P_KWMTBOMO13405	P_KWMTBOMO13405	P_KWMTBOMO13405	1
140	2.17	2.17	2.595	0.8949	0.5369	P_KWMTBOMO05728	P_KWMTBOMO05728	P_KWMTBOMO05728	1
142	2.11	2.12	6.6869996	3.647	3.647	P_KWMTBOMO08776	P_KWMTBOMO08776	P_KWMTBOMO08776	1
143	2.11	2.11	8.1990004	2.251	2.251	P_KWMTBOMO02432	P_KWMTBOMO02432	P_KWMTBOMO02432	1
144	2.11	2.11	4.2180002	2.4809999	2.4809999	P_KWMTBOMO11098	P_KWMTBOMO11098	P_KWMTBOMO11098	1
145	2.07	2.07	5.3160001	2.5319999	2.5319999	P_KWMTBOMO08310	P_KWMTBOMO08310	P_KWMTBOMO08310	1
146	2.04	2.04	3.4729999	0.9471	0.9471	P_KWMTBOMO03067	P_KWMTBOMO03067	P_KWMTBOMO03067	1
147	2.04	2.04	2.152	1.133	1.133	P_KWMTBOMO08045	P_KWMTBOMO08045	P_KWMTBOMO08045	1
148	2.03	2.03	5.3800002	3.3229999	3.3229999	P_KWMTBOMO08044	P_KWMTBOMO08044	P_KWMTBOMO08044	1
149	2.02	2.02	4.9789999	1.245	1.245	P_KWMTBOMO05741	P_KWMTBOMO05741	P_KWMTBOMO05741	1
151	2.02	2.02	1.8920001	0.9461	0.9461	P_KWMTBOMO01790	P_KWMTBOMO01790	P_KWMTBOMO01790	1
152	2.01	2.01	13.31	2.823	2.823	P_KWMTBOMO12037	P_KWMTBOMO12037	P_KWMTBOMO12037	1
153	2.01	2.01	21.33	7.333	7.333	P_KWMTBOMO04504	P_KWMTBOMO04504	P_KWMTBOMO04504	1
154	2.01	2.01	3.743	2.273	2.273	P_KWMTBOMO16130	P_KWMTBOMO16130	P_KWMTBOMO16130	1
155	2	4	5.4919999	5.4919999	5.4919999	P_KWMTBOMO15123	P_KWMTBOMO15123	P_KWMTBOMO15123	2
156	2	2	5.1849999	2.407	2.407	P_KWMTBOMO12659	P_KWMTBOMO12659	P_KWMTBOMO12659	1
157	2	2	7.2920002	1.7859999	1.7859999	P_KWMTBOMO08865	P_KWMTBOMO08865	P_KWMTBOMO08865	1
158	2	2	3.0719999	1.097	1.097	P_KWMTBOMO02211	P_KWMTBOMO02211	P_KWMTBOMO02211	1
159	2	2	5.658	2.079	2.079	P_KWMTBOMO01030	P_KWMTBOMO01030	P_KWMTBOMO01030	1
160	2	2	11.03	5.8970001	5.8970001	P_KWMTBOMO16309	P_KWMTBOMO16309	P_KWMTBOMO16309	1
161	2	2	2.22	0.793	0.793	P_KWMTBOMO14773	P_KWMTBOMO14773	P_KWMTBOMO14773	1
162	2	2	3.4120001	1.386	1.386	P_KWMTBOMO12667	P_KWMTBOMO12667	P_KWMTBOMO12667	1
163	2	2	5.7360001	1.912	1.912	P_KWMTBOMO12264	P_KWMTBOMO12264	P_KWMTBOMO12264	1
165	2	2	4.0690001	1.322	1.322	P_KWMTBOMO08404	P_KWMTBOMO08404	P_KWMTBOMO08404	1
166	2	2	14.98	6.763	6.763	P_KWMTBOMO08106	P_KWMTBOMO08106	P_KWMTBOMO08106	1
167	2	2	5.9209999	1.809	1.809	P_KWMTBOMO06787	P_KWMTBOMO06787	P_KWMTBOMO06787	1
168	2	2	3.7930001	1.77	1.77	P_KWMTBOMO05612	P_KWMTBOMO05612	P_KWMTBOMO05612	1
169	2	2	7.1429998	5.7599999	2.995	P_KWMTBOMO01114	P_KWMTBOMO01114	P_KWMTBOMO01114	1
170	2	2	6.5339997	3.977	3.977	P_KWMTBOMO16530	P_KWMTBOMO16530	P_KWMTBOMO16530	1
171	2	2	3.9329998	1.545	1.545	P_KWMTBOMO13226	P_KWMTBOMO13226	P_KWMTBOMO13226	1

172	2	2	2.282	1.091	1.091	P_KWMTBOMO12350	P_KWMTBOMO12350	P_KWMTBOMO12350	1
173	2	2	5.5300001	2.7650001	2.7650001	P_KWMTBOMO12165	P_KWMTBOMO12165	P_KWMTBOMO12165	1
174	2	2	6.6270001	3.6139999	3.6139999	P_KWMTBOMO12057	P_KWMTBOMO12057	P_KWMTBOMO12057	1
175	2	2	12.620001	6.5420002	6.5420002	P_KWMTBOMO11448	P_KWMTBOMO11448	P_KWMTBOMO11448	1
176	2	2	6.4439997	3.3330001	3.3330001	P_KWMTBOMO10283	P_KWMTBOMO10283	P_KWMTBOMO10283	1
177	2	2	5.1029999	1.8230001	1.8230001	P_KWMTBOMO09057	P_KWMTBOMO09057	P_KWMTBOMO09057	1
178	2	2	3.3610001	2.101	2.101	P_KWMTBOMO09034	P_KWMTBOMO09034	P_KWMTBOMO09034	1
179	2	2	19.61	11.11	11.11	P_KWMTBOMO08820	P_KWMTBOMO08820	P_KWMTBOMO08820	1
180	2	2	3.52	2.3469999	2.3469999	P_KWMTBOMO07380	P_KWMTBOMO07380	P_KWMTBOMO07380	1
181	2	2	3.3369999	1.891	1.891	P_KWMTBOMO07274	P_KWMTBOMO07274	P_KWMTBOMO07274	1
182	2	2	19.230001	12.5	12.5	P_KWMTBOMO06974	P_KWMTBOMO06974	P_KWMTBOMO06974	1
183	2	2	5.1759999	2.4839999	2.4839999	P_KWMTBOMO06731	P_KWMTBOMO06731	P_KWMTBOMO06731	1
184	2	2	2.382	0.8777	0.8777	P_KWMTBOMO06482	P_KWMTBOMO06482	P_KWMTBOMO06482	1
185	2	2	6.005	3.695	3.695	P_KWMTBOMO04605	P_KWMTBOMO04605	P_KWMTBOMO04605	1
186	2	2	5.4579999	3.5089999	3.5089999	P_KWMTBOMO00881	P_KWMTBOMO00881	P_KWMTBOMO00881	1
187	2	2	19.310001	9.6529998	9.6529998	P_KWMTBOMO00339	P_KWMTBOMO00339	P_KWMTBOMO00339	1
188	2	2	4.8149999	4.8149999	4.8149999	P_KWMTBOMO15867	P_KWMTBOMO15867	P_KWMTBOMO15867	1
190	2	2	2.935	2.935	2.935	P_KWMTBOMO14804	P_KWMTBOMO14804	P_KWMTBOMO14804	1
191	2	2	12.16	12.16	12.16	P_KWMTBOMO14440	P_KWMTBOMO14440	P_KWMTBOMO14440	1
193	2	2	5.2919999	5.2919999	5.2919999	P_KWMTBOMO08607	P_KWMTBOMO08607	P_KWMTBOMO08607	1
194	2	2	2.4940001	2.4940001	2.4940001	P_KWMTBOMO08194	P_KWMTBOMO08194	P_KWMTBOMO08194	1
195	2	2	17.389999	17.389999	17.389999	P_KWMTBOMO08193	P_KWMTBOMO08193	P_KWMTBOMO08193	1
196	2	2	6.7570001	6.7570001	6.7570001	P_KWMTBOMO07930	P_KWMTBOMO07930	P_KWMTBOMO07930	1
197	2	2	3.8860001	3.8860001	3.8860001	P_KWMTBOMO07780	P_KWMTBOMO07780	P_KWMTBOMO07780	1
198	2	2	2.111	2.111	2.111	P_KWMTBOMO07076	P_KWMTBOMO07076	P_KWMTBOMO07076	1
199	2	2	4.8930001	4.8930001	4.8930001	P_KWMTBOMO06056	P_KWMTBOMO06056	P_KWMTBOMO06056	1
200	2	2	5.0719999	5.0719999	5.0719999	P_KWMTBOMO05975	P_KWMTBOMO05975	P_KWMTBOMO05975	1
201	2	2	2.369	2.369	2.369	P_KWMTBOMO05205	P_KWMTBOMO05205	P_KWMTBOMO05205	1
202	2	2	0.3514	0.3514	0.3514	P_KWMTBOMO05179	P_KWMTBOMO05179	P_KWMTBOMO05179	1
203	2	2	9.5239997	9.5239997	9.5239997	P_KWMTBOMO03601	P_KWMTBOMO03601	P_KWMTBOMO03601	1
204	2	2	3.686	3.686	3.686	P_KWMTBOMO02835	P_KWMTBOMO02835	P_KWMTBOMO02835	1
205	2	2	7.8950003	7.8950003	7.8950003	P_KWMTBOMO02059	P_KWMTBOMO02059	P_KWMTBOMO02059	1
207	2	2	11.72	11.72	11.72	P_KWMTBOMO00786	P_KWMTBOMO00786	P_KWMTBOMO00786	1
208	1.92	2	7.6920003	7.6920003	7.6920003	P_KWMTBOMO13859	P_KWMTBOMO13859	P_KWMTBOMO13859	1
209	1.89	1.89	4.651	4.651	4.651	P_KWMTBOMO09390	P_KWMTBOMO09390	P_KWMTBOMO09390	1
210	1.82	1.82	5.074	1.9029999	1.9029999	P_KWMTBOMO12205	P_KWMTBOMO12205	P_KWMTBOMO12205	1
211	1.81	2.17	41.960001	41.960001	36.610001	P_KWMTBOMO09230	P_KWMTBOMO09230	P_KWMTBOMO09230	2
212	1.8	1.8	27.149999	6.6229999	6.6229999	P_KWMTBOMO13960	P_KWMTBOMO13960	P_KWMTBOMO13960	1
213	1.77	1.77	3.96	3.96	2.7720001	P_KWMTBOMO11276	P_KWMTBOMO11276	P_KWMTBOMO11276	1
214	1.74	1.74	16.93	3.4480002	3.4480002	P_KWMTBOMO01148	P_KWMTBOMO01148	P_KWMTBOMO01148	1
215	1.73	1.73	12.4	6.9770001	6.9770001	P_KWMTBOMO03173	P_KWMTBOMO03173	P_KWMTBOMO03173	1
216	1.72	1.72	0.6018	0.3484	0.3484	P_KWMTBOMO11519	P_KWMTBOMO11519	P_KWMTBOMO11519	1
217	1.7	1.7	2.5830001	0.9298	0.9298	P_KWMTBOMO07589	P_KWMTBOMO07589	P_KWMTBOMO07589	1
218	1.7	1.7	27.270001	11.11	11.11	P_KWMTBOMO02431	P_KWMTBOMO02431	P_KWMTBOMO02431	2
219	1.68	1.68	2.9999999	2.9999999	2.9999999	P_KWMTBOMO08926	P_KWMTBOMO08926	P_KWMTBOMO08926	1
220	1.68	1.68	0.3481	0.3481	0.3481	P_KWMTBOMO05680	P_KWMTBOMO05680	P_KWMTBOMO05680	1
221	1.64	1.64	12.58	6.9540001	3.311	P_KWMTBOMO12389	P_KWMTBOMO12389	P_KWMTBOMO12389	1
222	1.64	1.64	3.9179999	3.9179999	1.493	P_KWMTBOMO07855	P_KWMTBOMO07855	P_KWMTBOMO07855	1

223	1.62	1.62	7.463	7.463	7.463	P_KWMTBOMO04691	P_KWMTBOMO04691	P_KWMTBOMO04691	1
224	1.47	1.47	19.61	4.4119999	4.4119999	P_KWMTBOMO03314	P_KWMTBOMO03314	P_KWMTBOMO03314	1
225	1.47	1.47	8.9780003	2.7860001	2.7860001	P_KWMTBOMO03986	P_KWMTBOMO03986	P_KWMTBOMO03986	1
226	1.46	1.47	2.4320001	2.4320001	2.4320001	P_KWMTBOMO15584	P_KWMTBOMO15584	P_KWMTBOMO15584	1
227	1.45	1.45	23.270001	12.58	7.5470001	P_KWMTBOMO08100	P_KWMTBOMO08100	P_KWMTBOMO08100	1
228	1.44	1.44	3.647	3.647	1.403	P_KWMTBOMO11336	P_KWMTBOMO11336	P_KWMTBOMO11336	1
229	1.43	1.43	2.61	1.461	1.461	P_KWMTBOMO14568	P_KWMTBOMO14568	P_KWMTBOMO14568	1
230	1.43	1.43	5.5709999	5.5709999	3.6210001	P_KWMTBOMO02894	P_KWMTBOMO02894	P_KWMTBOMO02894	1
231	1.37	1.37	2.722	1.815	1.815	P_KWMTBOMO14936	P_KWMTBOMO14936	P_KWMTBOMO14936	1
232	1.35	1.35	4.18	4.18	4.18	P_KWMTBOMO01312	P_KWMTBOMO01312	P_KWMTBOMO01312	1

Summary of the identified PIPs of fibH_L5D5

N	Unused	Total	%Cov	%Cov(50)	%Cov(95)	Accession	Citable Accession	Name	Peptides(95%)
2	41.03	41.03	38.47	34.05	32.519999	P_KWMTBOMO08642	P_KWMTBOMO08642	P_KWMTBOMO08642	24
3	39.75	39.75	50.459999	44.7	39.399999	P_KWMTBOMO06229	P_KWMTBOMO06229	P_KWMTBOMO06229	22
4	38.76	38.76	31.23	25.34	21.23	P_KWMTBOMO05687	P_KWMTBOMO05687	P_KWMTBOMO05687	21
5	38.25	38.25	31.529999	28.27	25.68	P_KWMTBOMO00462	P_KWMTBOMO00462	P_KWMTBOMO00462	22
10	32.69	32.69	26.499999	23.3	20	P_KWMTBOMO13293	P_KWMTBOMO13293	P_KWMTBOMO13293	17
12	30.69	30.69	26.13	25.27	17.49	P_KWMTBOMO09597	P_KWMTBOMO09597	P_KWMTBOMO09597	13
17	25.71	25.71	48.74	48.74	44.029999	P_KWMTBOMO05178	P_KWMTBOMO05178	P_KWMTBOMO05178	15
20	24.32	24.32	43.869999	39.410001	33.090001	P_KWMTBOMO11985	P_KWMTBOMO11985	P_KWMTBOMO11985	16
23	20.85	20.85	18.440001	14.97	10.83	P_KWMTBOMO10816	P_KWMTBOMO10816	P_KWMTBOMO10816	9
25	20.28	20.28	48.969999	38.139999	33.51	P_KWMTBOMO11255	P_KWMTBOMO11255	P_KWMTBOMO11255	10
26	20	20	15.989999	13.58	12.05	P_KWMTBOMO15159	P_KWMTBOMO15159	P_KWMTBOMO15159	11
27	19.94	19.94	24.510001	15.38	10.29	P_KWMTBOMO06595	P_KWMTBOMO06595	P_KWMTBOMO06595	8
28	19.87	19.87	49.05	42.590001	34.979999	P_KWMTBOMO13455	P_KWMTBOMO13455	P_KWMTBOMO13455	11
29	19.83	19.83	25.45	23.989999	19.45	P_KWMTBOMO11096	P_KWMTBOMO11096	P_KWMTBOMO11096	10
31	18.92	18.92	60.930002	55.629998	55.629998	P_KWMTBOMO08440	P_KWMTBOMO08440	P_KWMTBOMO08440	9
34	17.92	17.92	42.199999	39.140001	36.390001	P_KWMTBOMO06763	P_KWMTBOMO06763	P_KWMTBOMO06763	11
38	17.18	17.18	28.819999	25.72	22.62	P_KWMTBOMO03092	P_KWMTBOMO03092	P_KWMTBOMO03092	9
40	16.81	16.81	25.709999	21.520001	17.71	P_KWMTBOMO15198	P_KWMTBOMO15198	P_KWMTBOMO15198	13
41	16.75	16.75	74.110001	59.820002	59.820002	P_KWMTBOMO09876	P_KWMTBOMO09876	P_KWMTBOMO09876	10
44	16.23	16.23	22.17	14.37	10.96	P_KWMTBOMO00638	P_KWMTBOMO00638	P_KWMTBOMO00638	10
46	16.05	16.05	31.68	28.369999	19.859999	P_KWMTBOMO11083	P_KWMTBOMO11083	P_KWMTBOMO11083	9
47	15.9	15.9	38.550001	37.79	33.590001	P_KWMTBOMO08464	P_KWMTBOMO08464	P_KWMTBOMO08464	13
48	15.31	15.31	39.17	39.17	39.17	P_KWMTBOMO09232	P_KWMTBOMO09232	P_KWMTBOMO09232	10
50	15.17	15.17	24.54	20.88	15.019999	P_KWMTBOMO09442	P_KWMTBOMO09442	P_KWMTBOMO09442	8
52	14.93	14.93	18.24	18.24	16.54	P_KWMTBOMO13370	P_KWMTBOMO13370	P_KWMTBOMO13370	11
53	14.45	14.45	14.229999	11.27	8.7870002	P_KWMTBOMO09055	P_KWMTBOMO09055	P_KWMTBOMO09055	7
54	14	14	32.969999	29.4	27.200001	P_KWMTBOMO09553	P_KWMTBOMO09553	P_KWMTBOMO09553	7
55	13.97	13.97	34.77	32.780001	29.139999	P_KWMTBOMO12389	P_KWMTBOMO12389	P_KWMTBOMO12389	8
57	13.82	13.82	34.869999	34.869999	34.869999	P_KWMTBOMO07688	P_KWMTBOMO07688	P_KWMTBOMO07688	8
58	13.79	13.79	20.53	18.269999	13.75	P_KWMTBOMO09808	P_KWMTBOMO09808	P_KWMTBOMO09808	6
59	13.51	13.51	24.950001	18.58	18.58	P_KWMTBOMO00927	P_KWMTBOMO00927	P_KWMTBOMO00927	7
61	13.02	13.02	21.19	17.86	17.86	P_KWMTBOMO02081	P_KWMTBOMO02081	P_KWMTBOMO02081	11
62	12.77	12.77	22.18	14.27	12.35	P_KWMTBOMO15357	P_KWMTBOMO15357	P_KWMTBOMO15357	7
63	12.71	12.71	31.330001	28.330001	23.999999	P_KWMTBOMO14732	P_KWMTBOMO14732	P_KWMTBOMO14732	7
66	12.03	12.03	15.76	13.03	13.03	P_KWMTBOMO12895	P_KWMTBOMO12895	P_KWMTBOMO12895	11
67	12	12	12.5	12.5	12.5	P_KWMTBOMO07855	P_KWMTBOMO07855	P_KWMTBOMO07855	6

68	11.96	11.96	14.17	8.4830001	8.4830001	P_KWMTBOMO05541	P_KWMTBOMO05541	P_KWMTBOMO05541	7
69	11.9	11.9	46.329999	38.420001	33.329999	P_KWMTBOMO13800	P_KWMTBOMO13800	P_KWMTBOMO13800	6
70	11.72	12.75	26.910001	16.410001	15.270001	P_KWMTBOMO12560	P_KWMTBOMO12560	P_KWMTBOMO12560	6
71	11.7	11.7	31.079999	31.079999	25.34	P_KWMTBOMO16197	P_KWMTBOMO16197	P_KWMTBOMO16197	6
72	11.38	11.38	25.999999	23.4	21.510001	P_KWMTBOMO00375	P_KWMTBOMO00375	P_KWMTBOMO00375	6
73	11.37	11.37	7.6090001	4.0550001	3.6789998	P_KWMTBOMO08648	P_KWMTBOMO08648	P_KWMTBOMO08648	7
74	11.23	11.23	16.39	16.39	14.380001	P_KWMTBOMO04502	P_KWMTBOMO04502	P_KWMTBOMO04502	6
76	11.05	11.05	6.4669997	5.1630002	3.4779999	P_KWMTBOMO12285	P_KWMTBOMO12285	P_KWMTBOMO12285	6
77	10.82	10.82	45.950001	37.39	37.39	P_KWMTBOMO01424	P_KWMTBOMO01424	P_KWMTBOMO01424	10
78	10.79	10.79	24.699999	24.699999	19.88	P_KWMTBOMO01182	P_KWMTBOMO01182	P_KWMTBOMO01182	5
79	10.62	10.62	17.52	16.45	14.32	P_KWMTBOMO02470	P_KWMTBOMO02470	P_KWMTBOMO02470	5
80	10.21	10.21	22.61	15.83	15.83	P_KWMTBOMO12166	P_KWMTBOMO12166	P_KWMTBOMO12166	5
81	10.19	10.19	22.88	22.88	22.88	P_KWMTBOMO10248	P_KWMTBOMO10248	P_KWMTBOMO10248	8
82	10.14	10.14	40.000001	40.000001	34.290001	P_KWMTBOMO00838	P_KWMTBOMO00838	P_KWMTBOMO00838	8
83	10.09	10.09	23.08	23.08	23.08	P_KWMTBOMO08666	P_KWMTBOMO08666	P_KWMTBOMO08666	5
84	10.05	10.05	55.35	40.880001	40.880001	P_KWMTBOMO08100	P_KWMTBOMO08100	P_KWMTBOMO08100	6
85	10.04	10.05	25.709999	23.51	16.3	P_KWMTBOMO01148	P_KWMTBOMO01148	P_KWMTBOMO01148	6
87	9.85	9.85	35.47	35.47	30.809999	P_KWMTBOMO02278	P_KWMTBOMO02278	P_KWMTBOMO02278	5
88	9.85	9.85	28.18	28.18	28.18	P_KWMTBOMO16637	P_KWMTBOMO16637	P_KWMTBOMO16637	5
89	9.84	9.84	9.578	6.0679998	3.926	P_KWMTBOMO09747	P_KWMTBOMO09747	P_KWMTBOMO09747	5
90	9.8	9.8	32.42	26.480001	20.09	P_KWMTBOMO05979	P_KWMTBOMO05979	P_KWMTBOMO05979	5
91	9.77	9.77	8.9220002	8.9220002	7.8790002	P_KWMTBOMO14157	P_KWMTBOMO14157	P_KWMTBOMO14157	6
92	9.57	9.57	16.17	16.17	16.17	P_KWMTBOMO12461	P_KWMTBOMO12461	P_KWMTBOMO12461	6
93	9.52	9.52	26.050001	15.530001	15.530001	P_KWMTBOMO01720	P_KWMTBOMO01720	P_KWMTBOMO01720	6
94	9.49	9.49	42.570001	34.650001	34.650001	P_KWMTBOMO15361	P_KWMTBOMO15361	P_KWMTBOMO15361	6
95	9.38	9.38	36.880001	23.13	23.13	P_KWMTBOMO07772	P_KWMTBOMO07772	P_KWMTBOMO07772	5
96	9.35	9.35	14.129999	1.281	1.281	P_KWMTBOMO15365	P_KWMTBOMO15365	P_KWMTBOMO15365	5
97	9.25	9.25	22.849999	17.19	12.58	P_KWMTBOMO15675	P_KWMTBOMO15675	P_KWMTBOMO15675	6
98	9.2	9.2	15.31	15.31	9.5940001	P_KWMTBOMO14887	P_KWMTBOMO14887	P_KWMTBOMO14887	5
99	9.17	9.17	23.22	18.85	15.86	P_KWMTBOMO09054	P_KWMTBOMO09054	P_KWMTBOMO09054	5
100	9.08	9.08	21.1	18.350001	18.350001	P_KWMTBOMO00497	P_KWMTBOMO00497	P_KWMTBOMO00497	5
101	9.03	9.03	36.590001	36.590001	36.590001	P_KWMTBOMO12287	P_KWMTBOMO12287	P_KWMTBOMO12287	6
103	8.52	8.52	9.313	6.3139997	5.446	P_KWMTBOMO03067	P_KWMTBOMO03067	P_KWMTBOMO03067	5
104	8.44	8.44	11.16	7.5410001	6.5080002	P_KWMTBOMO07589	P_KWMTBOMO07589	P_KWMTBOMO07589	4
105	8.41	8.41	21.66	21.66	17.97	P_KWMTBOMO08596	P_KWMTBOMO08596	P_KWMTBOMO08596	4
106	8.22	8.22	17.51	11.16	7.9099998	P_KWMTBOMO09050	P_KWMTBOMO09050	P_KWMTBOMO09050	4
107	8.14	8.14	20.79	11.92	11.92	P_KWMTBOMO14984	P_KWMTBOMO14984	P_KWMTBOMO14984	6
108	8.13	8.13	16.57	12.29	12.29	P_KWMTBOMO07323	P_KWMTBOMO07323	P_KWMTBOMO07323	4
109	8.08	8.08	17.41	13.160001	13.160001	P_KWMTBOMO11680	P_KWMTBOMO11680	P_KWMTBOMO11680	5
110	8.06	8.06	7.587	5.119	5.119	P_KWMTBOMO09183	P_KWMTBOMO09183	P_KWMTBOMO09183	4
111	8.05	8.05	23.78	23.78	23.78	P_KWMTBOMO11678	P_KWMTBOMO11678	P_KWMTBOMO11678	4
112	8	8.22	53.780001	37.819999	32.769999	P_KWMTBOMO15114	P_KWMTBOMO15114	P_KWMTBOMO15114	7
113	8	8	9.025	4.8760001	4.8760001	P_KWMTBOMO05741	P_KWMTBOMO05741	P_KWMTBOMO05741	4
114	8	8	20.71	14.17	14.17	P_KWMTBOMO04370	P_KWMTBOMO04370	P_KWMTBOMO04370	4
116	7.88	7.88	12.620001	9.8329999	9.8329999	P_KWMTBOMO01075	P_KWMTBOMO01075	P_KWMTBOMO01075	5
117	7.67	7.67	20.550001	20.550001	20.09	P_KWMTBOMO08894	P_KWMTBOMO08894	P_KWMTBOMO08894	5
118	7.64	7.64	14.59	13.689999	10.09	P_KWMTBOMO03626	P_KWMTBOMO03626	P_KWMTBOMO03626	4
119	7.62	7.62	30.599999	30.599999	24.04	P_KWMTBOMO06480	P_KWMTBOMO06480	P_KWMTBOMO06480	4

120	7.58	7.58	12.05	8.0750003	5.342	P_KWMTBOMO11666	P_KWMTBOMO11666	P_KWMTBOMO11666	4
121	7.51	7.51	33.109999	32.449999	27.810001	P_KWMTBOMO13960	P_KWMTBOMO13960	P_KWMTBOMO13960	4
122	7.47	7.47	6.3529998	5.528	3.7129998	P_KWMTBOMO12628	P_KWMTBOMO12628	P_KWMTBOMO12628	4
123	7.19	7.19	25.38	21.15	12.69	P_KWMTBOMO07103	P_KWMTBOMO07103	P_KWMTBOMO07103	4
124	7.14	7.14	14.210001	9.7790003	6.6419996	P_KWMTBOMO00793	P_KWMTBOMO00793	P_KWMTBOMO00793	4
125	7.03	7.03	4.4130001	1.981	1.32	P_KWMTBOMO07761	P_KWMTBOMO07761	P_KWMTBOMO07761	4
126	6.88	6.88	44.119999	44.119999	29.409999	P_KWMTBOMO13569	P_KWMTBOMO13569	P_KWMTBOMO13569	5
127	6.87	6.87	7.0349999	4.9249999	3.92	P_KWMTBOMO10780	P_KWMTBOMO10780	P_KWMTBOMO10780	3
128	6.84	6.84	16.57	16.57	16.57	P_KWMTBOMO10129	P_KWMTBOMO10129	P_KWMTBOMO10129	4
129	6.74	6.74	35.249999	28.060001	21.58	P_KWMTBOMO08399	P_KWMTBOMO08399	P_KWMTBOMO08399	4
130	6.66	6.66	10.25	10.25	6.205	P_KWMTBOMO16000	P_KWMTBOMO16000	P_KWMTBOMO16000	3
131	6.54	6.54	19.12	19.12	18.629999	P_KWMTBOMO03314	P_KWMTBOMO03314	P_KWMTBOMO03314	5
132	6.46	6.46	32.620001	22.99	18.719999	P_KWMTBOMO12428	P_KWMTBOMO12428	P_KWMTBOMO12428	3
133	6.39	6.39	38.420001	38.420001	32.63	P_KWMTBOMO03113	P_KWMTBOMO03113	P_KWMTBOMO03113	4
134	6.25	6.25	9.2770003	5.2859999	3.2359999	P_KWMTBOMO04610	P_KWMTBOMO04610	P_KWMTBOMO04610	3
135	6.22	6.22	25.18	25.18	18.610001	P_KWMTBOMO01796	P_KWMTBOMO01796	P_KWMTBOMO01796	3
136	6.08	6.08	27.000001	26.499999	17.5	P_KWMTBOMO02477	P_KWMTBOMO02477	P_KWMTBOMO02477	5
137	6.02	6.02	31.09	23.32	23.32	P_KWMTBOMO05281	P_KWMTBOMO05281	P_KWMTBOMO05281	4
138	6.02	6.02	32.620001	29.409999	29.409999	P_KWMTBOMO07857	P_KWMTBOMO07857	P_KWMTBOMO07857	3
139	6	6	7.931	4.0520001	4.0520001	P_KWMTBOMO01806	P_KWMTBOMO01806	P_KWMTBOMO01806	3
140	6	6	9.0070002	6.6969998	6.6969998	P_KWMTBOMO04605	P_KWMTBOMO04605	P_KWMTBOMO04605	3
141	6	6	24.84	24.84	24.84	P_KWMTBOMO08820	P_KWMTBOMO08820	P_KWMTBOMO08820	4
142	6	6	8.946	8.946	8.946	P_KWMTBOMO08204	P_KWMTBOMO08204	P_KWMTBOMO08204	3
143	5.95	5.95	10.07	10.07	8.0090001	P_KWMTBOMO06660	P_KWMTBOMO06660	P_KWMTBOMO06660	4
144	5.91	5.91	13.98	13.98	13.98	P_KWMTBOMO10965	P_KWMTBOMO10965	P_KWMTBOMO10965	4
145	5.9	8.31	13.82	10.57	6.7359999	P_KWMTBOMO00427	P_KWMTBOMO00427	P_KWMTBOMO00427	5
146	5.89	5.9	9.9150002	3.559	2.458	P_KWMTBOMO09542	P_KWMTBOMO09542	P_KWMTBOMO09542	3
147	5.8	5.8	19.63	19.63	19.63	P_KWMTBOMO11448	P_KWMTBOMO11448	P_KWMTBOMO11448	3
148	5.74	5.74	43.040001	16.46	16.46	P_KWMTBOMO14479	P_KWMTBOMO14479	P_KWMTBOMO14479	3
149	5.62	5.62	28.95	20	14.740001	P_KWMTBOMO12018	P_KWMTBOMO12018	P_KWMTBOMO12018	3
150	5.61	5.76	12.04	12.04	10.54	P_KWMTBOMO14761	P_KWMTBOMO14761	P_KWMTBOMO14761	5
151	5.51	5.51	53.100002	32.74	32.74	P_KWMTBOMO08961	P_KWMTBOMO08961	P_KWMTBOMO08961	4
152	5.4	5.4	12.74	12.74	10.51	P_KWMTBOMO15758	P_KWMTBOMO15758	P_KWMTBOMO15758	4
153	5.39	5.39	7.5290002	7.5290002	7.5290002	P_KWMTBOMO09415	P_KWMTBOMO09415	P_KWMTBOMO09415	4
154	5.32	5.32	13.66	13.66	13.66	P_KWMTBOMO01431	P_KWMTBOMO01431	P_KWMTBOMO01431	3
155	5.28	5.28	18.59	18.59	18.59	P_KWMTBOMO16357	P_KWMTBOMO16357	P_KWMTBOMO16357	4
156	5.22	5.22	23	23	15.019999	P_KWMTBOMO05566	P_KWMTBOMO05566	P_KWMTBOMO05566	3
157	5.12	5.23	30.520001	24.41	24.41	P_KWMTBOMO16481	P_KWMTBOMO16481	P_KWMTBOMO16481	6
158	5.11	5.12	31.690001	18.11	10.7	P_KWMTBOMO04168	P_KWMTBOMO04168	P_KWMTBOMO04168	2
159	5.11	5.11	23.53	22.69	17.649999	P_KWMTBOMO12706	P_KWMTBOMO12706	P_KWMTBOMO12706	3
160	5.06	5.06	37.5	21.15	14.42	P_KWMTBOMO06974	P_KWMTBOMO06974	P_KWMTBOMO06974	3
161	5.04	5.04	13.330001	13.330001	13.330001	P_KWMTBOMO08655	P_KWMTBOMO08655	P_KWMTBOMO08655	5
162	5.02	5.02	14.77	6.9770001	6.9770001	P_KWMTBOMO01423	P_KWMTBOMO01423	P_KWMTBOMO01423	3
163	4.99	4.99	66.960001	66.960001	43.75	P_KWMTBOMO09230	P_KWMTBOMO09230	P_KWMTBOMO09230	2
164	4.99	4.99	10.13	7.8390002	7.8390002	P_KWMTBOMO08041	P_KWMTBOMO08041	P_KWMTBOMO08041	3
165	4.95	4.95	31.369999	26.47	15.2	P_KWMTBOMO02734	P_KWMTBOMO02734	P_KWMTBOMO02734	2
166	4.84	4.85	12.630001	9.0910003	9.0910003	P_KWMTBOMO15518	P_KWMTBOMO15518	P_KWMTBOMO15518	3
167	4.84	4.84	8.2220003	8.2220003	8.2220003	P_KWMTBOMO10283	P_KWMTBOMO10283	P_KWMTBOMO10283	3

168	4.81	4.81	7.0780002	7.0780002	7.0780002	P_KWMTBOMO14936	P_KWMTBOMO14936	P_KWMTBOMO14936	3
169	4.73	4.73	42.42	39.390001	26.26	P_KWMTBOMO02431	P_KWMTBOMO02431	P_KWMTBOMO02431	3
170	4.72	4.72	12.22	6.1110001	4.0739998	P_KWMTBOMO12659	P_KWMTBOMO12659	P_KWMTBOMO12659	2
171	4.69	4.69	7.4330002	7.4330002	5.61	P_KWMTBOMO11336	P_KWMTBOMO11336	P_KWMTBOMO11336	3
174	4.58	4.58	13.950001	4.834	3.7289999	P_KWMTBOMO15359	P_KWMTBOMO15359	P_KWMTBOMO15359	2
175	4.5	4.5	8.2520001	6.7129999	5.5939998	P_KWMTBOMO15060	P_KWMTBOMO15060	P_KWMTBOMO15060	3
176	4.45	4.45	9.6110001	9.6110001	8.0090001	P_KWMTBOMO14530	P_KWMTBOMO14530	P_KWMTBOMO14530	3
177	4.45	4.45	22.88	22.88	16.339999	P_KWMTBOMO11021	P_KWMTBOMO11021	P_KWMTBOMO11021	3
178	4.43	4.45	13.46	13.46	10.38	P_KWMTBOMO13405	P_KWMTBOMO13405	P_KWMTBOMO13405	3
179	4.39	4.39	38.81	18.66	18.66	P_KWMTBOMO14639	P_KWMTBOMO14639	P_KWMTBOMO14639	3
180	4.34	4.34	18.69	18.69	12.15	P_KWMTBOMO10128	P_KWMTBOMO10128	P_KWMTBOMO10128	2
181	4.29	4.29	21.080001	16.670001	9.804	P_KWMTBOMO01273	P_KWMTBOMO01273	P_KWMTBOMO01273	2
182	4.23	4.23	27.700001	19.589999	16.22	P_KWMTBOMO08064	P_KWMTBOMO08064	P_KWMTBOMO08064	3
183	4.19	4.19	17.33	12.94	6.8889998	P_KWMTBOMO08956	P_KWMTBOMO08956	P_KWMTBOMO08956	2
184	4.17	4.17	13.46	9.6150003	5.7689998	P_KWMTBOMO13729	P_KWMTBOMO13729	P_KWMTBOMO13729	2
185	4.16	4.16	6.1170001	4.7869999	4.1219998	P_KWMTBOMO06291	P_KWMTBOMO06291	P_KWMTBOMO06291	2
186	4.16	4.16	5.1460002	5.1460002	3.774	P_KWMTBOMO03301	P_KWMTBOMO03301	P_KWMTBOMO03301	2
187	4.15	4.16	4.5019999	4.5019999	4.5019999	P_KWMTBOMO03177	P_KWMTBOMO03177	P_KWMTBOMO03177	3
188	4.13	4.13	26.359999	26.359999	20.93	P_KWMTBOMO05723	P_KWMTBOMO05723	P_KWMTBOMO05723	4
189	4.08	4.08	4.4380002	1.241	1.241	P_KWMTBOMO06045	P_KWMTBOMO06045	P_KWMTBOMO06045	2
190	4.08	4.08	12.41	12.41	8.5110001	P_KWMTBOMO00362	P_KWMTBOMO00362	P_KWMTBOMO00362	2
192	4	5.84	10.37	5.556	3.4570001	P_KWMTBOMO12844	P_KWMTBOMO12844	P_KWMTBOMO12844	3
193	4	4	9.9569999	6.9260001	6.9260001	P_KWMTBOMO15547	P_KWMTBOMO15547	P_KWMTBOMO15547	2
194	4	4	10.98	10.98	10.98	P_KWMTBOMO10916	P_KWMTBOMO10916	P_KWMTBOMO10916	2
195	4	4	2.183	1.523	1.523	P_KWMTBOMO10056	P_KWMTBOMO10056	P_KWMTBOMO10056	2
196	4	4	8.4959999	6.3720003	6.3720003	P_KWMTBOMO08578	P_KWMTBOMO08578	P_KWMTBOMO08578	2
197	4	4	6.4029999	4.2229999	4.2229999	P_KWMTBOMO03430	P_KWMTBOMO03430	P_KWMTBOMO03430	2
198	4	4	5.421	4.383	4.383	P_KWMTBOMO02535	P_KWMTBOMO02535	P_KWMTBOMO02535	2
199	4	4	35.620001	35.620001	35.620001	P_KWMTBOMO08098	P_KWMTBOMO08098	P_KWMTBOMO08098	2
200	4	4	16.850001	16.850001	16.850001	P_KWMTBOMO00860	P_KWMTBOMO00860	P_KWMTBOMO00860	2
201	3.9	3.9	12.59	6.8970002	3.9659999	P_KWMTBOMO02088	P_KWMTBOMO02088	P_KWMTBOMO02088	2
202	3.87	3.87	8.9780003	8.9780003	8.9780003	P_KWMTBOMO03986	P_KWMTBOMO03986	P_KWMTBOMO03986	3
203	3.82	3.82	24.73	24.73	24.73	P_KWMTBOMO06427	P_KWMTBOMO06427	P_KWMTBOMO06427	4
204	3.77	3.77	5.3270001	2.5420001	1.7109999	P_KWMTBOMO12878	P_KWMTBOMO12878	P_KWMTBOMO12878	3
205	3.76	3.76	8.4749997	3.7889998	2.592	P_KWMTBOMO04051	P_KWMTBOMO04051	P_KWMTBOMO04051	2
206	3.72	3.72	3.2680001	3.2680001	3.2680001	P_KWMTBOMO10779	P_KWMTBOMO10779	P_KWMTBOMO10779	2
207	3.7	3.7	5.618	5.618	5.618	P_KWMTBOMO02840	P_KWMTBOMO02840	P_KWMTBOMO02840	2
208	3.69	3.69	9.2749998	3.3330001	3.3330001	P_KWMTBOMO14549	P_KWMTBOMO14549	P_KWMTBOMO14549	2
209	3.67	3.67	44.299999	30.87	30.87	P_KWMTBOMO13520	P_KWMTBOMO13520	P_KWMTBOMO13520	3
210	3.66	3.67	3.1649999	3.1649999	2.2150001	P_KWMTBOMO08343	P_KWMTBOMO08343	P_KWMTBOMO08343	2
211	3.62	3.62	20.33	20.33	11.54	P_KWMTBOMO04529	P_KWMTBOMO04529	P_KWMTBOMO04529	2
212	3.59	3.59	8.7130003	8.7130003	6.1390001	P_KWMTBOMO00982	P_KWMTBOMO00982	P_KWMTBOMO00982	2
213	3.54	3.54	34.619999	34.619999	34.619999	P_KWMTBOMO12193	P_KWMTBOMO12193	P_KWMTBOMO12193	2
214	3.51	3.51	7.2870001	3.2600001	2.2050001	P_KWMTBOMO04806	P_KWMTBOMO04806	P_KWMTBOMO04806	2
215	3.47	3.47	6.2339999	6.2339999	6.2339999	P_KWMTBOMO14041	P_KWMTBOMO14041	P_KWMTBOMO14041	2
216	3.45	3.45	21.430001	12.5	12.5	P_KWMTBOMO04740	P_KWMTBOMO04740	P_KWMTBOMO04740	2
217	3.44	3.44	6.8800002	6.8800002	6.8800002	P_KWMTBOMO02835	P_KWMTBOMO02835	P_KWMTBOMO02835	2
218	3.42	3.42	2.74	2.283	2.283	P_KWMTBOMO04906	P_KWMTBOMO04906	P_KWMTBOMO04906	2

219	3.41	3.41	4.1579999	4.1579999	4.1579999	P_KWMTBOMO07374	P_KWMTBOMO07374	P_KWMTBOMO07374	2
220	3.38	3.38	17.44	12.31	12.31	P_KWMTBOMO09355	P_KWMTBOMO09355	P_KWMTBOMO09355	2
221	3.36	3.36	9.4700001	9.4700001	9.4700001	P_KWMTBOMO05651	P_KWMTBOMO05651	P_KWMTBOMO05651	2
222	3.28	3.28	9.2869997	3.624	2.605	P_KWMTBOMO08045	P_KWMTBOMO08045	P_KWMTBOMO08045	2
223	3.12	3.12	18.179999	18.179999	18.179999	P_KWMTBOMO01055	P_KWMTBOMO01055	P_KWMTBOMO01055	3
224	3.09	3.09	8.9309998	6.442	3.8070001	P_KWMTBOMO01452	P_KWMTBOMO01452	P_KWMTBOMO01452	2
225	3.05	3.05	7.3310003	5.263	5.263	P_KWMTBOMO03722	P_KWMTBOMO03722	P_KWMTBOMO03722	2
226	3.02	3.02	6.8149999	4.5960002	4.5960002	P_KWMTBOMO12094	P_KWMTBOMO12094	P_KWMTBOMO12094	2
227	3.02	3.02	39.289999	39.289999	17.86	P_KWMTBOMO03951	P_KWMTBOMO03951	P_KWMTBOMO03951	1
228	3.01	3.01	12.32	8.6400002	5.147	P_KWMTBOMO10337	P_KWMTBOMO10337	P_KWMTBOMO10337	2
229	2.99	2.99	18.009999	12.32	12.32	P_KWMTBOMO15757	P_KWMTBOMO15757	P_KWMTBOMO15757	2
230	2.97	2.97	9.6969999	9.6969999	9.6969999	P_KWMTBOMO02914	P_KWMTBOMO02914	P_KWMTBOMO02914	2
231	2.96	2.96	11.16	7.3629998	5.463	P_KWMTBOMO04330	P_KWMTBOMO04330	P_KWMTBOMO04330	2
232	2.95	2.95	28.459999	21.950001	10.57	P_KWMTBOMO01941	P_KWMTBOMO01941	P_KWMTBOMO01941	1
233	2.94	2.94	6.9360003	2.1190001	2.1190001	P_KWMTBOMO04768	P_KWMTBOMO04768	P_KWMTBOMO04768	2
235	2.88	2.88	6.735	5.4790001	1.484	P_KWMTBOMO09621	P_KWMTBOMO09621	P_KWMTBOMO09621	1
236	2.86	2.86	8.3779998	4.3880001	4.3880001	P_KWMTBOMO14942	P_KWMTBOMO14942	P_KWMTBOMO14942	2
237	2.83	2.83	6.4839996	4.115	4.115	P_KWMTBOMO01072	P_KWMTBOMO01072	P_KWMTBOMO01072	2
238	2.81	2.81	8.4250003	8.4250003	8.4250003	P_KWMTBOMO04789	P_KWMTBOMO04789	P_KWMTBOMO04789	2
239	2.76	2.76	8.1330001	5.3599998	5.3599998	P_KWMTBOMO06999	P_KWMTBOMO06999	P_KWMTBOMO06999	2
240	2.75	5.05	16.27	16.27	16.27	P_KWMTBOMO03027	P_KWMTBOMO03027	P_KWMTBOMO03027	3
241	2.74	2.74	5.0140001	5.0140001	2.7860001	P_KWMTBOMO10358	P_KWMTBOMO10358	P_KWMTBOMO10358	1
242	2.71	2.71	12.29	9.7769998	3.3519998	P_KWMTBOMO14715	P_KWMTBOMO14715	P_KWMTBOMO14715	2
243	2.69	2.69	8.0920003	4.8950002	3.9960001	P_KWMTBOMO00722	P_KWMTBOMO00722	P_KWMTBOMO00722	1
244	2.68	2.68	8.1550002	6.4379998	4.5060001	P_KWMTBOMO02500	P_KWMTBOMO02500	P_KWMTBOMO02500	2
245	2.66	2.66	16.35	14.1	9.2950001	P_KWMTBOMO15665	P_KWMTBOMO15665	P_KWMTBOMO15665	2
246	2.64	2.64	17.16	17.16	14.219999	P_KWMTBOMO09631	P_KWMTBOMO09631	P_KWMTBOMO09631	2
247	2.63	2.63	18.799999	9.6000001	4.8	P_KWMTBOMO11583	P_KWMTBOMO11583	P_KWMTBOMO11583	1
248	2.61	2.61	9.4520003	7.5609997	3.4030002	P_KWMTBOMO01606	P_KWMTBOMO01606	P_KWMTBOMO01606	1
249	2.59	2.59	11.06	6.5459996	2.935	P_KWMTBOMO14804	P_KWMTBOMO14804	P_KWMTBOMO14804	1
250	2.58	2.58	8.6960003	8.6960003	5.3509999	P_KWMTBOMO11061	P_KWMTBOMO11061	P_KWMTBOMO11061	1
251	2.53	2.53	5.8479998	3.5089999	3.5089999	P_KWMTBOMO04170	P_KWMTBOMO04170	P_KWMTBOMO04170	2
252	2.5	2.5	5.184	5.184	5.184	P_KWMTBOMO12208	P_KWMTBOMO12208	P_KWMTBOMO12208	2
253	2.48	2.48	13.4	5.412	3.6079999	P_KWMTBOMO14160	P_KWMTBOMO14160	P_KWMTBOMO14160	1
254	2.48	2.48	34.740001	34.740001	14.740001	P_KWMTBOMO14478	P_KWMTBOMO14478	P_KWMTBOMO14478	1
255	2.46	2.46	2.6140001	1.542	0.6702	P_KWMTBOMO07214	P_KWMTBOMO07214	P_KWMTBOMO07214	1
256	2.45	2.45	5.8649998	2.933	2.933	P_KWMTBOMO05321	P_KWMTBOMO05321	P_KWMTBOMO05321	2
257	2.4	2.4	11.89	8.6489998	4.324	P_KWMTBOMO15584	P_KWMTBOMO15584	P_KWMTBOMO15584	1
258	2.4	2.4	8.602	6.2369999	3.441	P_KWMTBOMO05564	P_KWMTBOMO05564	P_KWMTBOMO05564	1
259	2.4	2.4	13.14	13.14	8.5709997	P_KWMTBOMO16319	P_KWMTBOMO16319	P_KWMTBOMO16319	1
260	2.36	2.36	16.45	6.9410004	3.0850001	P_KWMTBOMO05464	P_KWMTBOMO05464	P_KWMTBOMO05464	1
261	2.31	2.31	8.845	5.235	3.249	P_KWMTBOMO09838	P_KWMTBOMO09838	P_KWMTBOMO09838	1
262	2.29	2.29	31.54	24.62	24.62	P_KWMTBOMO07368	P_KWMTBOMO07368	P_KWMTBOMO07368	2
263	2.25	2.25	12.92	12.92	8.427	P_KWMTBOMO05078	P_KWMTBOMO05078	P_KWMTBOMO05078	1
264	2.24	2.24	12.78	4.0449999	2.589	P_KWMTBOMO03630	P_KWMTBOMO03630	P_KWMTBOMO03630	1
265	2.24	2.24	4.2550001	4.2550001	2.5529999	P_KWMTBOMO15125	P_KWMTBOMO15125	P_KWMTBOMO15125	1
266	2.23	2.23	32.429999	12.84	6.7570001	P_KWMTBOMO07930	P_KWMTBOMO07930	P_KWMTBOMO07930	1
267	2.22	2.22	25.709999	25.709999	11.43	P_KWMTBOMO10049	P_KWMTBOMO10049	P_KWMTBOMO10049	1

268	2.18	2.18	4.5170002	0.7652	0.3456	P_KWMTBOMO12602	P_KWMTBOMO12602	P_KWMTBOMO12602	1
271	2.13	2.13	26.620001	17.27	6.4750001	P_KWMTBOMO11987	P_KWMTBOMO11987	P_KWMTBOMO11987	1
272	2.11	2.11	4.7619998	4.7619998	2.692	P_KWMTBOMO00473	P_KWMTBOMO00473	P_KWMTBOMO00473	1
273	2.1	2.1	18.359999	8.213	4.831	P_KWMTBOMO08106	P_KWMTBOMO08106	P_KWMTBOMO08106	1
274	2.1	2.1	19.69	8.2680002	4.3310001	P_KWMTBOMO04594	P_KWMTBOMO04594	P_KWMTBOMO04594	1
275	2.1	2.1	6.7160003	2.564	1.099	P_KWMTBOMO00303	P_KWMTBOMO00303	P_KWMTBOMO00303	1
276	2.08	2.08	12.56	8.7180004	5.8970001	P_KWMTBOMO16309	P_KWMTBOMO16309	P_KWMTBOMO16309	1
277	2.07	2.07	4.1359998	2.6760001	2.6760001	P_KWMTBOMO00390	P_KWMTBOMO00390	P_KWMTBOMO00390	1
278	2.06	2.06	8.6669996	5.9999999	5.9999999	P_KWMTBOMO02775	P_KWMTBOMO02775	P_KWMTBOMO02775	1
279	2.04	2.04	20.100001	12.06	6.03	P_KWMTBOMO00200	P_KWMTBOMO00200	P_KWMTBOMO00200	1
280	2.03	2.03	6.3429996	6.3429996	3.7310001	P_KWMTBOMO11161	P_KWMTBOMO11161	P_KWMTBOMO11161	1
281	2.03	2.03	18.46	9.2309996	9.2309996	P_KWMTBOMO06373	P_KWMTBOMO06373	P_KWMTBOMO06373	1
282	2.03	2.03	11.6	11.6	7.9999998	P_KWMTBOMO04427	P_KWMTBOMO04427	P_KWMTBOMO04427	1
283	2.03	2.03	5.3879999	2.802	2.802	P_KWMTBOMO00373	P_KWMTBOMO00373	P_KWMTBOMO00373	1
284	2.02	2.02	9.4959997	6.202	3.2949999	P_KWMTBOMO06354	P_KWMTBOMO06354	P_KWMTBOMO06354	1
285	2.02	2.02	18.92	18.92	12.16	P_KWMTBOMO14440	P_KWMTBOMO14440	P_KWMTBOMO14440	1
286	2.02	2.02	3.7930001	2.069	2.069	P_KWMTBOMO11590	P_KWMTBOMO11590	P_KWMTBOMO11590	1
287	2.02	2.02	6.061	4.408	4.408	P_KWMTBOMO01802	P_KWMTBOMO01802	P_KWMTBOMO01802	1
289	2.01	2.01	5.556	1.778	1.778	P_KWMTBOMO06822	P_KWMTBOMO06822	P_KWMTBOMO06822	1
290	2.01	2.01	8.1840001	2.7939999	2.7939999	P_KWMTBOMO15121	P_KWMTBOMO15121	P_KWMTBOMO15121	1
291	2.01	2.01	6.4309999	4.18	4.18	P_KWMTBOMO01312	P_KWMTBOMO01312	P_KWMTBOMO01312	1
292	2	4	5.4919999	5.4919999	5.4919999	P_KWMTBOMO15123	P_KWMTBOMO15123	P_KWMTBOMO15123	2
293	2	4	10.28	10.28	10.28	P_KWMTBOMO13774	P_KWMTBOMO13774	P_KWMTBOMO13774	2
294	2	2.01	4.473	2.8750001	2.8750001	P_KWMTBOMO05299	P_KWMTBOMO05299	P_KWMTBOMO05299	1
295	2	2.01	10.49	7.4069999	7.4069999	P_KWMTBOMO03622	P_KWMTBOMO03622	P_KWMTBOMO03622	1
296	2	2	3.858	0.5735	0.5735	P_KWMTBOMO15243	P_KWMTBOMO15243	P_KWMTBOMO15243	1
297	2	2	3.7500001	1.6070001	1.6070001	P_KWMTBOMO10243	P_KWMTBOMO10243	P_KWMTBOMO10243	1
298	2	2	17.18	4.58	4.58	P_KWMTBOMO00837	P_KWMTBOMO00837	P_KWMTBOMO00837	1
299	2	2	15.32	7.2070003	7.2070003	P_KWMTBOMO16375	P_KWMTBOMO16375	P_KWMTBOMO16375	1
300	2	2	10.37	10.37	4.521	P_KWMTBOMO14845	P_KWMTBOMO14845	P_KWMTBOMO14845	1
301	2	2	40.23	22.99	22.99	P_KWMTBOMO13722	P_KWMTBOMO13722	P_KWMTBOMO13722	1
302	2	2	9.6770003	5.0689999	5.0689999	P_KWMTBOMO13597	P_KWMTBOMO13597	P_KWMTBOMO13597	1
303	2	2	24.240001	11.36	11.36	P_KWMTBOMO10591	P_KWMTBOMO10591	P_KWMTBOMO10591	1
304	2	2	8.0779999	5.2919999	5.2919999	P_KWMTBOMO08607	P_KWMTBOMO08607	P_KWMTBOMO08607	1
305	2	2	6.8630002	2.696	2.696	P_KWMTBOMO07554	P_KWMTBOMO07554	P_KWMTBOMO07554	1
306	2	2	10.03	4.425	4.425	P_KWMTBOMO07278	P_KWMTBOMO07278	P_KWMTBOMO07278	1
307	2	2	11.59	5.0719999	5.0719999	P_KWMTBOMO05975	P_KWMTBOMO05975	P_KWMTBOMO05975	1
309	2	2	5.1720001	3.0169999	3.0169999	P_KWMTBOMO01708	P_KWMTBOMO01708	P_KWMTBOMO01708	1
310	2	2	1.8440001	0.922	0.922	P_KWMTBOMO01579	P_KWMTBOMO01579	P_KWMTBOMO01579	1
311	2	2	3.8740002	3.8740002	3.8740002	P_KWMTBOMO16261	P_KWMTBOMO16261	P_KWMTBOMO16261	1
312	2	2	3.077	3.077	3.077	P_KWMTBOMO15781	P_KWMTBOMO15781	P_KWMTBOMO15781	1
313	2	2	7.6920003	7.6920003	7.6920003	P_KWMTBOMO13859	P_KWMTBOMO13859	P_KWMTBOMO13859	1
314	2	2	5.263	5.263	5.263	P_KWMTBOMO13723	P_KWMTBOMO13723	P_KWMTBOMO13723	1
315	2	2	4.3650001	4.3650001	4.3650001	P_KWMTBOMO12448	P_KWMTBOMO12448	P_KWMTBOMO12448	1
316	2	2	16.92	16.92	16.92	P_KWMTBOMO12400	P_KWMTBOMO12400	P_KWMTBOMO12400	1
317	2	2	4.5139998	4.5139998	4.5139998	P_KWMTBOMO11202	P_KWMTBOMO11202	P_KWMTBOMO11202	1
318	2	2	2.376	2.376	2.376	P_KWMTBOMO11110	P_KWMTBOMO11110	P_KWMTBOMO11110	1
319	2	2	4.183	4.183	4.183	P_KWMTBOMO09709	P_KWMTBOMO09709	P_KWMTBOMO09709	1

320	2	2	1.9640001	1.9640001	1.9640001	P_KWMTBOMO08707	P_KWMTBOMO08707	P_KWMTBOMO08707	1
321	2	2	5.263	5.263	5.263	P_KWMTBOMO08323	P_KWMTBOMO08323	P_KWMTBOMO08323	1
322	2	2	2.709	2.709	2.709	P_KWMTBOMO08056	P_KWMTBOMO08056	P_KWMTBOMO08056	1
323	2	2	14.71	14.71	14.71	P_KWMTBOMO07600	P_KWMTBOMO07600	P_KWMTBOMO07600	1
324	2	2	2.8759999	2.8759999	2.8759999	P_KWMTBOMO05205	P_KWMTBOMO05205	P_KWMTBOMO05205	1
325	2	2	5.0190002	5.0190002	5.0190002	P_KWMTBOMO04992	P_KWMTBOMO04992	P_KWMTBOMO04992	1
326	2	2	2.994	2.994	2.994	P_KWMTBOMO04966	P_KWMTBOMO04966	P_KWMTBOMO04966	1
327	2	2	6.0109999	6.0109999	6.0109999	P_KWMTBOMO04584	P_KWMTBOMO04584	P_KWMTBOMO04584	1
328	2	2	2.936	2.936	2.936	P_KWMTBOMO03631	P_KWMTBOMO03631	P_KWMTBOMO03631	1
329	2	2	8.5709997	8.5709997	8.5709997	P_KWMTBOMO03003	P_KWMTBOMO03003	P_KWMTBOMO03003	1
330	2	2	13.89	13.89	13.89	P_KWMTBOMO02542	P_KWMTBOMO02542	P_KWMTBOMO02542	1
331	2	2	12.58	12.58	12.58	P_KWMTBOMO02450	P_KWMTBOMO02450	P_KWMTBOMO02450	1
332	2	2	4.8719998	4.8719998	4.8719998	P_KWMTBOMO02223	P_KWMTBOMO02223	P_KWMTBOMO02223	1
333	2	2	1.5939999	1.5939999	1.5939999	P_KWMTBOMO01817	P_KWMTBOMO01817	P_KWMTBOMO01817	1
334	2	2	7.2729997	7.2729997	7.2729997	P_KWMTBOMO01799	P_KWMTBOMO01799	P_KWMTBOMO01799	1
335	2	2	6.5729998	6.5729998	6.5729998	P_KWMTBOMO01662	P_KWMTBOMO01662	P_KWMTBOMO01662	1
336	2	2	3.9999999	3.9999999	3.9999999	P_KWMTBOMO01315	P_KWMTBOMO01315	P_KWMTBOMO01315	1
337	2	2	7.2729997	7.2729997	7.2729997	P_KWMTBOMO01129	P_KWMTBOMO01129	P_KWMTBOMO01129	1
338	2	2	5.2129999	5.2129999	5.2129999	P_KWMTBOMO00794	P_KWMTBOMO00794	P_KWMTBOMO00794	1
339	1.92	1.92	9.2050001	9.2050001	5.858	P_KWMTBOMO05784	P_KWMTBOMO05784	P_KWMTBOMO05784	1
340	1.92	1.92	9.3170002	9.3170002	9.3170002	P_KWMTBOMO07061	P_KWMTBOMO07061	P_KWMTBOMO07061	1
341	1.92	1.92	6.3639998	6.3639998	6.3639998	P_KWMTBOMO06639	P_KWMTBOMO06639	P_KWMTBOMO06639	1
342	1.92	1.92	3.836	3.836	3.836	P_KWMTBOMO05806	P_KWMTBOMO05806	P_KWMTBOMO05806	1
343	1.84	1.84	7.124	7.124	4.4849999	P_KWMTBOMO05258	P_KWMTBOMO05258	P_KWMTBOMO05258	1
344	1.82	1.82	4.8050001	4.8050001	4.8050001	P_KWMTBOMO09250	P_KWMTBOMO09250	P_KWMTBOMO09250	1
345	1.8	1.8	14.97	14.97	8.8440001	P_KWMTBOMO12483	P_KWMTBOMO12483	P_KWMTBOMO12483	2
346	1.8	1.8	6.1159998	3.1399999	3.1399999	P_KWMTBOMO04132	P_KWMTBOMO04132	P_KWMTBOMO04132	1
347	1.74	1.74	19.509999	9.7560003	4.3900002	P_KWMTBOMO15407	P_KWMTBOMO15407	P_KWMTBOMO15407	1
348	1.72	1.72	2.0020001	1.144	1.144	P_KWMTBOMO12965	P_KWMTBOMO12965	P_KWMTBOMO12965	1
349	1.7	1.7	4.5200001	4.5200001	4.5200001	P_KWMTBOMO01380	P_KWMTBOMO01380	P_KWMTBOMO01380	1
350	1.66	1.66	15.5	6.6869996	3.647	P_KWMTBOMO08776	P_KWMTBOMO08776	P_KWMTBOMO08776	1
351	1.62	1.62	4.6300001	4.6300001	4.6300001	P_KWMTBOMO16600	P_KWMTBOMO16600	P_KWMTBOMO16600	1
352	1.6	1.6	2.132	1.066	1.066	P_KWMTBOMO12667	P_KWMTBOMO12667	P_KWMTBOMO12667	1
353	1.58	1.58	14.29	8.5249998	4.3779999	P_KWMTBOMO01114	P_KWMTBOMO01114	P_KWMTBOMO01114	2
354	1.55	1.55	2.2390001	2.2390001	2.2390001	P_KWMTBOMO11689	P_KWMTBOMO11689	P_KWMTBOMO11689	1
355	1.52	1.52	11.9	9.5239997	1.905	P_KWMTBOMO16204	P_KWMTBOMO16204	P_KWMTBOMO16204	1
356	1.52	1.52	10.09	10.09	10.09	P_KWMTBOMO06299	P_KWMTBOMO06299	P_KWMTBOMO06299	1
357	1.52	1.52	3.4090001	3.4090001	3.4090001	P_KWMTBOMO03431	P_KWMTBOMO03431	P_KWMTBOMO03431	1
358	1.46	1.46	3.5089999	3.5089999	3.5089999	P_KWMTBOMO03721	P_KWMTBOMO03721	P_KWMTBOMO03721	1
359	1.43	1.43	11.34	11.34	11.34	P_KWMTBOMO01918	P_KWMTBOMO01918	P_KWMTBOMO01918	1
360	1.42	5.77	16.75	16.75	16.75	P_KWMTBOMO06927	P_KWMTBOMO06927	P_KWMTBOMO06927	3
361	1.41	1.41	7.463	7.463	7.463	P_KWMTBOMO11416	P_KWMTBOMO11416	P_KWMTBOMO11416	2
362	1.41	1.41	4.5570001	2.5319999	2.5319999	P_KWMTBOMO08310	P_KWMTBOMO08310	P_KWMTBOMO08310	1
363	1.4	1.4	8.427	1.685	1.685	P_KWMTBOMO13226	P_KWMTBOMO13226	P_KWMTBOMO13226	1
364	1.36	1.36	3.9329998	3.9329998	3.9329998	P_KWMTBOMO15419	P_KWMTBOMO15419	P_KWMTBOMO15419	1
365	1.35	1.35	3.1520002	3.1520002	3.1520002	P_KWMTBOMO01076	P_KWMTBOMO01076	P_KWMTBOMO01076	1
366	1.34	1.34	2.9410001	0.8913	0.4902	P_KWMTBOMO05810	P_KWMTBOMO05810	P_KWMTBOMO05810	1
367	1.34	1.34	6.0679998	1.138	1.138	P_KWMTBOMO05612	P_KWMTBOMO05612	P_KWMTBOMO05612	1

368	1.33	1.33	6.25	6.25	6.25	P_KWMTBOMO00265	P_KWMTBOMO00265	P_KWMTBOMO00265	1
369	1.32	1.32	3.743	3.743	3.743	P_KWMTBOMO15712	P_KWMTBOMO15712	P_KWMTBOMO15712	1
370	1.31	1.31	7.2800003	2.874	2.874	P_KWMTBOMO15179	P_KWMTBOMO15179	P_KWMTBOMO15179	1
371	1.31	1.31	9.9260002	6.6179998	6.6179998	P_KWMTBOMO09672	P_KWMTBOMO09672	P_KWMTBOMO09672	1
372	1.3	1.31	13.11	13.11	13.11	P_KWMTBOMO12189	P_KWMTBOMO12189	P_KWMTBOMO12189	1
373	1.3	1.3	6.5640002	6.5640002	6.5640002	P_KWMTBOMO00339	P_KWMTBOMO00339	P_KWMTBOMO00339	1

Summary of the identified PIPs of fibL_M4

N	Unused	Total	%Cov	%Cov(50)	%Cov(95)	Accession	Citable Accession	Name	Peptides(95%)
3	34.52	34.52	14.300001	11.37	10.12	P_KWMTBOMO08648	P_KWMTBOMO08648	P_KWMTBOMO08648	18
10	21.55	21.55	22.99	19.08	15.4	P_KWMTBOMO11731	P_KWMTBOMO11731	P_KWMTBOMO11731	12
12	19.05	19.05	19.140001	13.510001	13.510001	P_KWMTBOMO00462	P_KWMTBOMO00462	P_KWMTBOMO00462	11
14	18.14	26.2	24.12	22.6	22.44	P_KWMTBOMO08642	P_KWMTBOMO08642	P_KWMTBOMO08642	16
17	15.96	15.96	46.14	34.259999	19.410001	P_KWMTBOMO07374	P_KWMTBOMO07374	P_KWMTBOMO07374	9
18	15.69	15.69	8.7739997	7.1070001	7.1070001	P_KWMTBOMO14858	P_KWMTBOMO14858	P_KWMTBOMO14858	8
19	15.34	15.34	28.439999	23.35	19.769999	P_KWMTBOMO09808	P_KWMTBOMO09808	P_KWMTBOMO09808	8
21	15.09	15.09	22.99	20.58	19.13	P_KWMTBOMO03748	P_KWMTBOMO03748	P_KWMTBOMO03748	8
22	15.03	15.03	31.600001	29.370001	19.7	P_KWMTBOMO11985	P_KWMTBOMO11985	P_KWMTBOMO11985	9
24	14.5	14.5	25.27	25.27	20.51	P_KWMTBOMO09442	P_KWMTBOMO09442	P_KWMTBOMO09442	8
25	13.86	13.86	10.96	9.6170001	7.446	P_KWMTBOMO01927	P_KWMTBOMO01927	P_KWMTBOMO01927	7
27	13.03	13.03	8.9850001	5.7360001	4.315	P_KWMTBOMO10056	P_KWMTBOMO10056	P_KWMTBOMO10056	7
28	12.75	12.75	12.49	10.3	8.3240002	P_KWMTBOMO15159	P_KWMTBOMO15159	P_KWMTBOMO15159	6
29	12.57	12.57	31.05	19.74	19.74	P_KWMTBOMO01720	P_KWMTBOMO01720	P_KWMTBOMO01720	7
30	12.48	12.48	40.059999	31.819999	24.429999	P_KWMTBOMO05675	P_KWMTBOMO05675	P_KWMTBOMO05675	8
31	12.36	12.36	8.0499999	5.424	2.884	P_KWMTBOMO11045	P_KWMTBOMO11045	P_KWMTBOMO11045	5
33	12.11	12.11	22.579999	16.72	15.719999	P_KWMTBOMO04502	P_KWMTBOMO04502	P_KWMTBOMO04502	8
34	11.99	11.99	20.42	14.749999	10.7	P_KWMTBOMO11096	P_KWMTBOMO11096	P_KWMTBOMO11096	6
36	11.28	11.28	10.7	7.914	7.914	P_KWMTBOMO14420	P_KWMTBOMO14420	P_KWMTBOMO14420	6
37	11.22	11.22	33.64	17.28	13.36	P_KWMTBOMO06229	P_KWMTBOMO06229	P_KWMTBOMO06229	5
38	10.84	10.84	29.159999	24.62	20.09	P_KWMTBOMO03005	P_KWMTBOMO03005	P_KWMTBOMO03005	7
40	10.64	10.64	41.240001	35.04	28.470001	P_KWMTBOMO01796	P_KWMTBOMO01796	P_KWMTBOMO01796	6
41	10.37	10.37	33.840001	29.280001	17.110001	P_KWMTBOMO13455	P_KWMTBOMO13455	P_KWMTBOMO13455	4
42	10.27	10.27	15.2	10.6	7.8000002	P_KWMTBOMO13293	P_KWMTBOMO13293	P_KWMTBOMO13293	6
44	9.99	9.99	18.179999	16.509999	15.03	P_KWMTBOMO01075	P_KWMTBOMO01075	P_KWMTBOMO01075	5
46	9.55	9.55	16.670001	11.16	7.203	P_KWMTBOMO09050	P_KWMTBOMO09050	P_KWMTBOMO09050	4
48	9.4	9.4	10.7	3.6120001	1.6249999	P_KWMTBOMO06632	P_KWMTBOMO06632	P_KWMTBOMO06632	3
49	9.31	9.31	39.340001	33.329999	29.51	P_KWMTBOMO06480	P_KWMTBOMO06480	P_KWMTBOMO06480	6
51	9.14	9.14	18.350001	18.350001	18.350001	P_KWMTBOMO00497	P_KWMTBOMO00497	P_KWMTBOMO00497	5
52	9.06	9.06	34.020001	29.899999	20.62	P_KWMTBOMO11255	P_KWMTBOMO11255	P_KWMTBOMO11255	4
54	8.76	8.76	32.949999	24.14	21.84	P_KWMTBOMO07688	P_KWMTBOMO07688	P_KWMTBOMO07688	5
55	8.73	8.73	11.88	7.2849996	5.5890001	P_KWMTBOMO05541	P_KWMTBOMO05541	P_KWMTBOMO05541	4
56	8.68	8.68	17.309999	14.32	12.18	P_KWMTBOMO02470	P_KWMTBOMO02470	P_KWMTBOMO02470	4
57	8.38	8.38	9.6660003	6.5009996	3.678	P_KWMTBOMO04505	P_KWMTBOMO04505	P_KWMTBOMO04505	4
58	8.18	8.18	15.189999	10.6	7.5949997	P_KWMTBOMO08044	P_KWMTBOMO08044	P_KWMTBOMO08044	4
59	8	8	35.769999	35.769999	35.769999	P_KWMTBOMO12287	P_KWMTBOMO12287	P_KWMTBOMO12287	4
60	8	8	24.49	24.49	24.49	P_KWMTBOMO10509	P_KWMTBOMO10509	P_KWMTBOMO10509	6
62	7.88	7.88	9.2569999	6.0899999	6.0899999	P_KWMTBOMO00638	P_KWMTBOMO00638	P_KWMTBOMO00638	4
63	7.86	7.86	9.685	6.9410004	3.067	P_KWMTBOMO05687	P_KWMTBOMO05687	P_KWMTBOMO05687	4
64	7.79	7.79	14.63	9.9440001	8.0679998	P_KWMTBOMO06770	P_KWMTBOMO06770	P_KWMTBOMO06770	4

65	7.78	7.89	8.4880002	7.9070002	6.3950002	P_KWMTBOMO15583	P_KWMTBOMO15583	P_KWMTBOMO15583	5
66	7.72	7.72	22.18	19.46	19.46	P_KWMTBOMO08983	P_KWMTBOMO08983	P_KWMTBOMO08983	7
67	7.69	7.69	21.04	16.159999	16.159999	P_KWMTBOMO06082	P_KWMTBOMO06082	P_KWMTBOMO06082	4
68	7.56	7.56	16.760001	11.89	10.09	P_KWMTBOMO03626	P_KWMTBOMO03626	P_KWMTBOMO03626	4
69	7.52	7.52	18.67	18.67	15.82	P_KWMTBOMO09300	P_KWMTBOMO09300	P_KWMTBOMO09300	4
70	7.47	7.47	14.41	13.150001	8.1419997	P_KWMTBOMO08956	P_KWMTBOMO08956	P_KWMTBOMO08956	3
71	7.44	7.44	17.86	13.1	13.1	P_KWMTBOMO02081	P_KWMTBOMO02081	P_KWMTBOMO02081	5
72	7.4	7.4	11.29	11.29	7.2860003	P_KWMTBOMO00927	P_KWMTBOMO00927	P_KWMTBOMO00927	3
74	7.24	7.24	14.390001	14.390001	14.390001	P_KWMTBOMO11192	P_KWMTBOMO11192	P_KWMTBOMO11192	5
75	7.17	7.17	35.100001	29.800001	29.800001	P_KWMTBOMO08440	P_KWMTBOMO08440	P_KWMTBOMO08440	4
76	7.1	7.1	11.93	10.28	10.28	P_KWMTBOMO13721	P_KWMTBOMO13721	P_KWMTBOMO13721	5
77	7.04	7.04	13.99	10.45	10.45	P_KWMTBOMO07855	P_KWMTBOMO07855	P_KWMTBOMO07855	4
78	7.02	7.02	11.81	11.81	7.0110001	P_KWMTBOMO00793	P_KWMTBOMO00793	P_KWMTBOMO00793	4
80	6.75	6.75	37.850001	25.99	20.900001	P_KWMTBOMO13800	P_KWMTBOMO13800	P_KWMTBOMO13800	3
81	6.73	6.73	16.9	11.97	11.97	P_KWMTBOMO00437	P_KWMTBOMO00437	P_KWMTBOMO00437	4
83	6.46	6.46	22.69	19.62	19.62	P_KWMTBOMO13405	P_KWMTBOMO13405	P_KWMTBOMO13405	4
84	6.39	6.39	21.67	16.329999	10.33	P_KWMTBOMO14732	P_KWMTBOMO14732	P_KWMTBOMO14732	4
85	6.11	6.11	17.06	17.06	10.37	P_KWMTBOMO11061	P_KWMTBOMO11061	P_KWMTBOMO11061	4
86	6.06	6.06	10.55	9.0400003	9.0400003	P_KWMTBOMO00366	P_KWMTBOMO00366	P_KWMTBOMO00366	3
87	6.05	6.05	14.820001	11.29	7.5290002	P_KWMTBOMO09415	P_KWMTBOMO09415	P_KWMTBOMO09415	5
88	6.03	6.03	30.379999	16.46	16.46	P_KWMTBOMO14479	P_KWMTBOMO14479	P_KWMTBOMO14479	3
89	6.01	6.01	10.68	3.9420001	3.9420001	P_KWMTBOMO05741	P_KWMTBOMO05741	P_KWMTBOMO05741	3
90	6	6	19.35	9.2739999	9.2739999	P_KWMTBOMO12037	P_KWMTBOMO12037	P_KWMTBOMO12037	3
91	6	6	21.55	21.55	21.55	P_KWMTBOMO16637	P_KWMTBOMO16637	P_KWMTBOMO16637	3
92	6	6	22.07	22.07	22.07	P_KWMTBOMO01424	P_KWMTBOMO01424	P_KWMTBOMO01424	4
93	5.83	5.83	12.899999	10.97	9.0319999	P_KWMTBOMO05564	P_KWMTBOMO05564	P_KWMTBOMO05564	3
94	5.8	5.8	4.7359999	2.6839999	2.6839999	P_KWMTBOMO03067	P_KWMTBOMO03067	P_KWMTBOMO03067	3
95	5.73	5.73	21.68	21.68	16.08	P_KWMTBOMO08666	P_KWMTBOMO08666	P_KWMTBOMO08666	3
96	5.72	5.72	5.4749999	4.3389998	4.3389998	P_KWMTBOMO07589	P_KWMTBOMO07589	P_KWMTBOMO07589	3
97	5.64	5.64	5.226	4.724	3.92	P_KWMTBOMO10780	P_KWMTBOMO10780	P_KWMTBOMO10780	3
98	5.59	5.59	47.799999	16.98	16.98	P_KWMTBOMO08100	P_KWMTBOMO08100	P_KWMTBOMO08100	4
99	5.55	5.55	16.339999	16.339999	13.070001	P_KWMTBOMO10248	P_KWMTBOMO10248	P_KWMTBOMO10248	4
100	5.54	5.54	11.89	10.84	10.84	P_KWMTBOMO03860	P_KWMTBOMO03860	P_KWMTBOMO03860	3
101	5.52	5.52	28.95	14.740001	14.740001	P_KWMTBOMO12018	P_KWMTBOMO12018	P_KWMTBOMO12018	3
102	5.44	5.44	18.57	18.57	18.57	P_KWMTBOMO00838	P_KWMTBOMO00838	P_KWMTBOMO00838	5
103	5.23	5.23	24.66	20.550001	9.589	P_KWMTBOMO05979	P_KWMTBOMO05979	P_KWMTBOMO05979	2
104	5.23	5.23	18.59	18.59	18.59	P_KWMTBOMO16357	P_KWMTBOMO16357	P_KWMTBOMO16357	4
105	5.11	5.11	12.85	8.4109999	8.4109999	P_KWMTBOMO14984	P_KWMTBOMO14984	P_KWMTBOMO14984	3
106	5.02	5.02	6.6179998	5.147	3.5289999	P_KWMTBOMO08652	P_KWMTBOMO08652	P_KWMTBOMO08652	4
107	5.02	5.02	19.63	19.63	19.63	P_KWMTBOMO11448	P_KWMTBOMO11448	P_KWMTBOMO11448	3
108	5	5	16.410001	16.410001	16.410001	P_KWMTBOMO01320	P_KWMTBOMO01320	P_KWMTBOMO01320	3
110	4.96	4.96	3.8509998	3.8509998	3.8509998	P_KWMTBOMO01817	P_KWMTBOMO01817	P_KWMTBOMO01817	3
111	4.9	4.9	38.240001	31.369999	29.409999	P_KWMTBOMO13569	P_KWMTBOMO13569	P_KWMTBOMO13569	5
112	4.77	4.77	4.834	4.834	3.7289999	P_KWMTBOMO15359	P_KWMTBOMO15359	P_KWMTBOMO15359	2
114	4.68	5.71	10.25	6.7900002	2.716	P_KWMTBOMO12844	P_KWMTBOMO12844	P_KWMTBOMO12844	2
115	4.68	4.68	11.78	8.2220003	6.222	P_KWMTBOMO10283	P_KWMTBOMO10283	P_KWMTBOMO10283	2
116	4.66	4.66	9.8710001	6.3110001	6.3110001	P_KWMTBOMO03630	P_KWMTBOMO03630	P_KWMTBOMO03630	3
117	4.6	4.6	4.9940001	3.0200001	3.0200001	P_KWMTBOMO00427	P_KWMTBOMO00427	P_KWMTBOMO00427	3

118	4.59	4.59	7.1029998	5.7100002	4.7350001	P_KWMTBOMO10358	P_KWMTBOMO10358	P_KWMTBOMO10358	2
119	4.55	4.55	17.829999	10.6	6.9880001	P_KWMTBOMO13979	P_KWMTBOMO13979	P_KWMTBOMO13979	2
120	4.42	4.42	5.5750001	1.832	1.287	P_KWMTBOMO12304	P_KWMTBOMO12304	P_KWMTBOMO12304	2
121	4.36	4.36	19.87	19.87	19.87	P_KWMTBOMO16040	P_KWMTBOMO16040	P_KWMTBOMO16040	3
122	4.34	4.34	9.4520003	6.8049997	4.9150001	P_KWMTBOMO01606	P_KWMTBOMO01606	P_KWMTBOMO01606	2
123	4.33	4.33	11.31	9.296	5.779	P_KWMTBOMO12166	P_KWMTBOMO12166	P_KWMTBOMO12166	2
124	4.3	4.3	13.249999	13.249999	9.6029997	P_KWMTBOMO12389	P_KWMTBOMO12389	P_KWMTBOMO12389	2
125	4.21	4.21	28.479999	22.52	14.569999	P_KWMTBOMO13960	P_KWMTBOMO13960	P_KWMTBOMO13960	2
126	4.18	4.18	13.72	7.6739997	6.2789999	P_KWMTBOMO02061	P_KWMTBOMO02061	P_KWMTBOMO02061	2
127	4.17	4.17	5.5020001	2.9920001	2.9920001	P_KWMTBOMO11517	P_KWMTBOMO11517	P_KWMTBOMO11517	2
128	4.17	4.17	10.09	10.09	8.0119997	P_KWMTBOMO10376	P_KWMTBOMO10376	P_KWMTBOMO10376	3
129	4.16	4.16	14.54	5.229	5.229	P_KWMTBOMO15928	P_KWMTBOMO15928	P_KWMTBOMO15928	2
130	4.15	4.15	32.929999	23.17	23.17	P_KWMTBOMO11678	P_KWMTBOMO11678	P_KWMTBOMO11678	3
131	4.13	4.13	15.49	9.155	9.155	P_KWMTBOMO07373	P_KWMTBOMO07373	P_KWMTBOMO07373	3
132	4.09	5.11	23.66	19.08	8.7789997	P_KWMTBOMO09980	P_KWMTBOMO09980	P_KWMTBOMO09980	3
133	4.08	4.08	17.910001	10.14	10.14	P_KWMTBOMO16197	P_KWMTBOMO16197	P_KWMTBOMO16197	2
134	4.08	4.08	22.28	10.89	10.89	P_KWMTBOMO15361	P_KWMTBOMO15361	P_KWMTBOMO15361	2
135	4.08	4.08	10.56	7.9180002	7.9180002	P_KWMTBOMO13997	P_KWMTBOMO13997	P_KWMTBOMO13997	2
136	4.07	4.07	14.42	4.3889999	4.3889999	P_KWMTBOMO01148	P_KWMTBOMO01148	P_KWMTBOMO01148	2
137	4.07	4.07	11.46	7.9620004	7.9620004	P_KWMTBOMO15758	P_KWMTBOMO15758	P_KWMTBOMO15758	2
138	4.06	4.06	7.1429998	6.5130003	4.4119999	P_KWMTBOMO12895	P_KWMTBOMO12895	P_KWMTBOMO12895	2
139	4.04	4.04	21.05	15.13	15.13	P_KWMTBOMO02059	P_KWMTBOMO02059	P_KWMTBOMO02059	2
140	4.03	4.03	33.590001	19.310001	19.310001	P_KWMTBOMO00339	P_KWMTBOMO00339	P_KWMTBOMO00339	2
141	4.01	4.01	5.6090001	3.2219999	3.2219999	P_KWMTBOMO08213	P_KWMTBOMO08213	P_KWMTBOMO08213	2
142	4	4	39.500001	19.329999	19.329999	P_KWMTBOMO15114	P_KWMTBOMO15114	P_KWMTBOMO15114	2
143	4	4	7.8829996	4.9460001	4.9460001	P_KWMTBOMO13370	P_KWMTBOMO13370	P_KWMTBOMO13370	2
144	4	4	5.8219999	3.1959999	3.1959999	P_KWMTBOMO09621	P_KWMTBOMO09621	P_KWMTBOMO09621	2
145	4	4	14.669999	11.33	11.33	P_KWMTBOMO02775	P_KWMTBOMO02775	P_KWMTBOMO02775	2
146	4	4	13.64	8.4420003	8.4420003	P_KWMTBOMO16524	P_KWMTBOMO16524	P_KWMTBOMO16524	2
147	4	4	5.9700001	4.2640001	4.2640001	P_KWMTBOMO14752	P_KWMTBOMO14752	P_KWMTBOMO14752	2
148	4	4	4.2130001	4.2130001	4.2130001	P_KWMTBOMO15419	P_KWMTBOMO15419	P_KWMTBOMO15419	2
149	4	4	6.3210003	6.3210003	6.3210003	P_KWMTBOMO14804	P_KWMTBOMO14804	P_KWMTBOMO14804	2
150	4	4	10.7	10.7	10.7	P_KWMTBOMO04168	P_KWMTBOMO04168	P_KWMTBOMO04168	2
151	4	4	4.7619998	4.7619998	4.7619998	P_KWMTBOMO02748	P_KWMTBOMO02748	P_KWMTBOMO02748	2
152	4	4	16.850001	16.850001	16.850001	P_KWMTBOMO00860	P_KWMTBOMO00860	P_KWMTBOMO00860	2
153	3.92	3.92	4.2640001	2.452	2.452	P_KWMTBOMO12667	P_KWMTBOMO12667	P_KWMTBOMO12667	2
155	3.88	3.88	14.34	11.92	6.3309997	P_KWMTBOMO07323	P_KWMTBOMO07323	P_KWMTBOMO07323	2
156	3.83	3.83	15.279999	9.6510001	9.6510001	P_KWMTBOMO06231	P_KWMTBOMO06231	P_KWMTBOMO06231	3
157	3.82	3.82	6.0029998	4.366	4.366	P_KWMTBOMO03177	P_KWMTBOMO03177	P_KWMTBOMO03177	3
158	3.82	3.82	9.8590001	9.8590001	9.8590001	P_KWMTBOMO05566	P_KWMTBOMO05566	P_KWMTBOMO05566	2
159	3.8	3.8	18.92	10.81	10.81	P_KWMTBOMO08064	P_KWMTBOMO08064	P_KWMTBOMO08064	2
160	3.77	3.77	4.4440001	4.4440001	4.4440001	P_KWMTBOMO12659	P_KWMTBOMO12659	P_KWMTBOMO12659	2
161	3.74	3.74	4.93	2.908	2.908	P_KWMTBOMO05612	P_KWMTBOMO05612	P_KWMTBOMO05612	2
162	3.74	3.74	3.122	1.987	1.987	P_KWMTBOMO01790	P_KWMTBOMO01790	P_KWMTBOMO01790	2
163	3.7	3.7	8.7760001	6.4669997	6.4669997	P_KWMTBOMO04605	P_KWMTBOMO04605	P_KWMTBOMO04605	2
164	3.66	3.66	6.3490003	4.7619998	4.7619998	P_KWMTBOMO13567	P_KWMTBOMO13567	P_KWMTBOMO13567	2
165	3.62	3.62	10.19	6.2350001	4.0770002	P_KWMTBOMO15357	P_KWMTBOMO15357	P_KWMTBOMO15357	2
166	3.54	3.54	12.549999	6.9569997	1.863	P_KWMTBOMO11666	P_KWMTBOMO11666	P_KWMTBOMO11666	1

167	3.51	3.51	11.82	9.4559997	4.9649999	P_KWMTBOMO11083	P_KWMTBOMO11083	P_KWMTBOMO11083	2
168	3.5	3.5	7.7169999	6.752	3.537	P_KWMTBOMO02432	P_KWMTBOMO02432	P_KWMTBOMO02432	2
169	3.43	4.67	26.789999	26.789999	26.789999	P_KWMTBOMO09230	P_KWMTBOMO09230	P_KWMTBOMO09230	3
170	3.36	3.36	10.09	3.2609999	3.2609999	P_KWMTBOMO00687	P_KWMTBOMO00687	P_KWMTBOMO00687	2
171	3.36	3.36	8.845	6.3879997	6.3879997	P_KWMTBOMO02835	P_KWMTBOMO02835	P_KWMTBOMO02835	2
172	3.31	3.31	13.73	10.59	10.59	P_KWMTBOMO10916	P_KWMTBOMO10916	P_KWMTBOMO10916	2
173	3.27	3.27	1.668	1.668	1.668	P_KWMTBOMO04676	P_KWMTBOMO04676	P_KWMTBOMO04676	2
174	3.22	3.22	20.93	20.93	14.73	P_KWMTBOMO05723	P_KWMTBOMO05723	P_KWMTBOMO05723	3
175	3.21	3.21	21.65	21.65	21.65	P_KWMTBOMO01918	P_KWMTBOMO01918	P_KWMTBOMO01918	2
176	3.19	3.19	24.06	14.97	10.7	P_KWMTBOMO12428	P_KWMTBOMO12428	P_KWMTBOMO12428	3
177	3.17	3.17	7.9570003	5.5909999	5.5909999	P_KWMTBOMO07811	P_KWMTBOMO07811	P_KWMTBOMO07811	2
178	3.15	3.15	14.71	9.6550003	5.7470001	P_KWMTBOMO09054	P_KWMTBOMO09054	P_KWMTBOMO09054	2
179	3.13	3.13	22.059999	13.73	5.8820002	P_KWMTBOMO01273	P_KWMTBOMO01273	P_KWMTBOMO01273	1
180	3.12	3.12	20.32	17.110001	17.110001	P_KWMTBOMO07857	P_KWMTBOMO07857	P_KWMTBOMO07857	2
181	3.11	3.11	10.14	6.2320001	2.029	P_KWMTBOMO14549	P_KWMTBOMO14549	P_KWMTBOMO14549	1
182	3.1	3.1	8.4320001	5.565	3.204	P_KWMTBOMO00834	P_KWMTBOMO00834	P_KWMTBOMO00834	2
183	3.08	3.08	5.618	5.618	5.618	P_KWMTBOMO02840	P_KWMTBOMO02840	P_KWMTBOMO02840	2
184	3.06	3.06	14.69	3.9549999	3.9549999	P_KWMTBOMO02994	P_KWMTBOMO02994	P_KWMTBOMO02994	2
185	3.04	3.04	38.56	24.84	18.95	P_KWMTBOMO08820	P_KWMTBOMO08820	P_KWMTBOMO08820	2
187	2.95	2.95	10.24	10.24	10.24	P_KWMTBOMO08535	P_KWMTBOMO08535	P_KWMTBOMO08535	2
188	2.93	2.93	4.7460001	3.6010001	3.6010001	P_KWMTBOMO05572	P_KWMTBOMO05572	P_KWMTBOMO05572	2
189	2.87	2.87	5.466	5.466	5.466	P_KWMTBOMO11680	P_KWMTBOMO11680	P_KWMTBOMO11680	2
190	2.86	2.86	45.24	29.76	29.76	P_KWMTBOMO03951	P_KWMTBOMO03951	P_KWMTBOMO03951	3
191	2.79	2.79	6.4130001	6.4130001	6.4130001	P_KWMTBOMO08893	P_KWMTBOMO08893	P_KWMTBOMO08893	2
192	2.77	2.77	16.72	8.9780003	5.263	P_KWMTBOMO03986	P_KWMTBOMO03986	P_KWMTBOMO03986	2
193	2.73	2.73	19.679999	12.13	12.13	P_KWMTBOMO06660	P_KWMTBOMO06660	P_KWMTBOMO06660	3
194	2.72	2.73	11.04	7.3569998	3.1339999	P_KWMTBOMO03430	P_KWMTBOMO03430	P_KWMTBOMO03430	2
195	2.72	2.72	7.3940001	5.7300001	5.7300001	P_KWMTBOMO06760	P_KWMTBOMO06760	P_KWMTBOMO06760	2
196	2.65	2.65	7.3540002	4.2020001	1.719	P_KWMTBOMO09055	P_KWMTBOMO09055	P_KWMTBOMO09055	1
198	2.6	2.6	18.38	8.0779999	8.0779999	P_KWMTBOMO08607	P_KWMTBOMO08607	P_KWMTBOMO08607	2
199	2.57	2.57	5.7799999	3.6990002	1.503	P_KWMTBOMO06595	P_KWMTBOMO06595	P_KWMTBOMO06595	1
200	2.55	2.55	20.190001	13.46	12.5	P_KWMTBOMO06974	P_KWMTBOMO06974	P_KWMTBOMO06974	1
201	2.54	2.54	15.440001	10.14	7.3729999	P_KWMTBOMO01114	P_KWMTBOMO01114	P_KWMTBOMO01114	2
202	2.5	2.5	13.330001	13.330001	13.330001	P_KWMTBOMO09355	P_KWMTBOMO09355	P_KWMTBOMO09355	2
203	2.48	2.48	3.8940001	3.8940001	2.6550001	P_KWMTBOMO08578	P_KWMTBOMO08578	P_KWMTBOMO08578	1
204	2.44	2.44	11.81	6.4580001	2.9519999	P_KWMTBOMO14887	P_KWMTBOMO14887	P_KWMTBOMO14887	1
205	2.41	2.41	9.3249999	6.4309999	4.18	P_KWMTBOMO01312	P_KWMTBOMO01312	P_KWMTBOMO01312	1
207	2.33	2.33	12.4	12.4	6.9770001	P_KWMTBOMO03173	P_KWMTBOMO03173	P_KWMTBOMO03173	1
208	2.29	2.29	3.7409998	2.734	1.439	P_KWMTBOMO10493	P_KWMTBOMO10493	P_KWMTBOMO10493	1
209	2.2	2.2	2.517	2.517	1.818	P_KWMTBOMO15060	P_KWMTBOMO15060	P_KWMTBOMO15060	1
210	2.19	2.19	9.2270002	4.5060001	2.7899999	P_KWMTBOMO02500	P_KWMTBOMO02500	P_KWMTBOMO02500	1
211	2.18	4.09	8.2379997	8.2379997	5.4919999	P_KWMTBOMO15123	P_KWMTBOMO15123	P_KWMTBOMO15123	2
212	2.16	2.16	11.49	11.49	6.7570001	P_KWMTBOMO07930	P_KWMTBOMO07930	P_KWMTBOMO07930	1
213	2.13	2.13	8.4749997	0.9322	0.9322	P_KWMTBOMO09542	P_KWMTBOMO09542	P_KWMTBOMO09542	1
214	2.13	2.13	20.290001	7.8259997	7.8259997	P_KWMTBOMO12903	P_KWMTBOMO12903	P_KWMTBOMO12903	2
215	2.13	2.13	7.5910002	7.5910002	5.2809998	P_KWMTBOMO03169	P_KWMTBOMO03169	P_KWMTBOMO03169	1
216	2.12	2.12	4.8489999	4.8489999	3.0099999	P_KWMTBOMO12208	P_KWMTBOMO12208	P_KWMTBOMO12208	1
217	2.1	2.1	2.892	1.891	1.891	P_KWMTBOMO07274	P_KWMTBOMO07274	P_KWMTBOMO07274	1

218	2.09	2.09	39.35	13.55	8.3870001	P_KWMTBOMO05068	P_KWMTBOMO05068	P_KWMTBOMO05068	2
219	2.07	2.07	6.6869996	3.647	3.647	P_KWMTBOMO08776	P_KWMTBOMO08776	P_KWMTBOMO08776	1
220	2.06	2.06	4.078	2.33	2.33	P_KWMTBOMO07952	P_KWMTBOMO07952	P_KWMTBOMO07952	1
221	2.06	2.06	11.54	6.044	6.044	P_KWMTBOMO04529	P_KWMTBOMO04529	P_KWMTBOMO04529	1
222	2.03	2.03	7.3229998	2.778	2.778	P_KWMTBOMO15518	P_KWMTBOMO15518	P_KWMTBOMO15518	1
223	2.03	2.03	7.265	2.3499999	2.3499999	P_KWMTBOMO13729	P_KWMTBOMO13729	P_KWMTBOMO13729	1
224	2.03	2.03	4.9710002	1.912	1.912	P_KWMTBOMO12264	P_KWMTBOMO12264	P_KWMTBOMO12264	1
225	2.03	2.03	19.37	8.7499999	8.7499999	P_KWMTBOMO07772	P_KWMTBOMO07772	P_KWMTBOMO07772	1
226	2.03	2.03	15.2	6.3730001	6.3730001	P_KWMTBOMO02734	P_KWMTBOMO02734	P_KWMTBOMO02734	1
227	2.02	3.06	2.09	0.4837	0.3676	P_KWMTBOMO08740	P_KWMTBOMO08740	P_KWMTBOMO08740	2
228	2.02	2.02	10.29	4.4849999	4.4849999	P_KWMTBOMO05258	P_KWMTBOMO05258	P_KWMTBOMO05258	1
229	2.02	2.02	1.492	0.8032	0.8032	P_KWMTBOMO06334	P_KWMTBOMO06334	P_KWMTBOMO06334	1
230	2.01	2.01	26.519999	8.3329998	8.3329998	P_KWMTBOMO09788	P_KWMTBOMO09788	P_KWMTBOMO09788	1
231	2.01	2.01	4.1510001	1.099	1.099	P_KWMTBOMO00303	P_KWMTBOMO00303	P_KWMTBOMO00303	1
232	2.01	2.01	1.6869999	1.6869999	1.091	P_KWMTBOMO12350	P_KWMTBOMO12350	P_KWMTBOMO12350	1
233	2	3.7	3.641	3.641	3.641	P_KWMTBOMO09034	P_KWMTBOMO09034	P_KWMTBOMO09034	2
234	2	2	5.9489999	0.6544	0.6544	P_KWMTBOMO09747	P_KWMTBOMO09747	P_KWMTBOMO09747	2
235	2	2	4.8300002	0.8669	0.8669	P_KWMTBOMO15715	P_KWMTBOMO15715	P_KWMTBOMO15715	1
236	2	2	16.74	6.4219996	2.523	P_KWMTBOMO09591	P_KWMTBOMO09591	P_KWMTBOMO09591	1
237	2	2	2.3089999	0.8722	0.8722	P_KWMTBOMO12968	P_KWMTBOMO12968	P_KWMTBOMO12968	1
238	2	2	8.5249998	2.7650001	2.7650001	P_KWMTBOMO12165	P_KWMTBOMO12165	P_KWMTBOMO12165	1
239	2	2	8.7350003	3.6139999	3.6139999	P_KWMTBOMO12057	P_KWMTBOMO12057	P_KWMTBOMO12057	1
240	2	2	7.0689999	2.069	2.069	P_KWMTBOMO11590	P_KWMTBOMO11590	P_KWMTBOMO11590	1
241	2	2	9.7939998	4.1239999	4.1239999	P_KWMTBOMO10299	P_KWMTBOMO10299	P_KWMTBOMO10299	1
242	2	2	13.04	6.763	6.763	P_KWMTBOMO08106	P_KWMTBOMO08106	P_KWMTBOMO08106	1
243	2	2	7.209	2.957	2.957	P_KWMTBOMO06999	P_KWMTBOMO06999	P_KWMTBOMO06999	1
244	2	2	13.349999	3.8150001	3.8150001	P_KWMTBOMO04370	P_KWMTBOMO04370	P_KWMTBOMO04370	1
245	2	2	8.8420004	3.9999999	3.9999999	P_KWMTBOMO01315	P_KWMTBOMO01315	P_KWMTBOMO01315	1
246	2	2	4.6130002	1.746	1.746	P_KWMTBOMO01072	P_KWMTBOMO01072	P_KWMTBOMO01072	1
247	2	2	25	6.8180002	6.8180002	P_KWMTBOMO01055	P_KWMTBOMO01055	P_KWMTBOMO01055	1
248	2	2	3.4839999	1.9160001	1.9160001	P_KWMTBOMO14633	P_KWMTBOMO14633	P_KWMTBOMO14633	1
249	2	2	4.8909999	2.1740001	2.1740001	P_KWMTBOMO14483	P_KWMTBOMO14483	P_KWMTBOMO14483	1
250	2	2	9.0279996	4.5139998	4.5139998	P_KWMTBOMO11202	P_KWMTBOMO11202	P_KWMTBOMO11202	1
251	2	2	4.9380001	3.0859999	3.0859999	P_KWMTBOMO09385	P_KWMTBOMO09385	P_KWMTBOMO09385	1
252	2	2	9.4080001	5.923	5.923	P_KWMTBOMO08930	P_KWMTBOMO08930	P_KWMTBOMO08930	1
253	2	2	6.4769998	3.8860001	3.8860001	P_KWMTBOMO07780	P_KWMTBOMO07780	P_KWMTBOMO07780	1
254	2	2	3.1860001	2.696	2.696	P_KWMTBOMO07554	P_KWMTBOMO07554	P_KWMTBOMO07554	1
255	2	2	2.0129999	2.0129999	1.151	P_KWMTBOMO04806	P_KWMTBOMO04806	P_KWMTBOMO04806	1
256	2	2	10.62	4.3960001	4.3960001	P_KWMTBOMO04789	P_KWMTBOMO04789	P_KWMTBOMO04789	1
257	2	2	4.8870001	2.8200001	2.8200001	P_KWMTBOMO03722	P_KWMTBOMO03722	P_KWMTBOMO03722	1
258	2	2	5.8479998	3.5089999	3.5089999	P_KWMTBOMO00881	P_KWMTBOMO00881	P_KWMTBOMO00881	1
259	2	2	12.32	12.32	5.2129999	P_KWMTBOMO00794	P_KWMTBOMO00794	P_KWMTBOMO00794	1
260	2	2	3.8740002	3.8740002	3.8740002	P_KWMTBOMO16261	P_KWMTBOMO16261	P_KWMTBOMO16261	1
261	2	2	3.077	3.077	3.077	P_KWMTBOMO15781	P_KWMTBOMO15781	P_KWMTBOMO15781	1
262	2	2	3.743	3.743	3.743	P_KWMTBOMO15712	P_KWMTBOMO15712	P_KWMTBOMO15712	1
263	2	2	12.16	12.16	12.16	P_KWMTBOMO14440	P_KWMTBOMO14440	P_KWMTBOMO14440	1
264	2	2	6.1900001	6.1900001	6.1900001	P_KWMTBOMO13641	P_KWMTBOMO13641	P_KWMTBOMO13641	1
265	2	2	1.674	1.674	1.674	P_KWMTBOMO13230	P_KWMTBOMO13230	P_KWMTBOMO13230	1

266	2	2	3.015	3.015	3.015	P_KWMTBOMO12902	P_KWMTBOMO12902	P_KWMTBOMO12902	1
267	2	2	16.92	16.92	16.92	P_KWMTBOMO12400	P_KWMTBOMO12400	P_KWMTBOMO12400	1
268	2	2	8.6209998	8.6209998	8.6209998	P_KWMTBOMO11771	P_KWMTBOMO11771	P_KWMTBOMO11771	1
269	2	2	2.424	2.424	2.424	P_KWMTBOMO09954	P_KWMTBOMO09954	P_KWMTBOMO09954	1
270	2	2	4.6689998	4.6689998	4.6689998	P_KWMTBOMO08542	P_KWMTBOMO08542	P_KWMTBOMO08542	1
271	2	2	5.263	5.263	5.263	P_KWMTBOMO08323	P_KWMTBOMO08323	P_KWMTBOMO08323	1
272	2	2	2.4940001	2.4940001	2.4940001	P_KWMTBOMO08194	P_KWMTBOMO08194	P_KWMTBOMO08194	1
273	2	2	17.389999	17.389999	17.389999	P_KWMTBOMO08193	P_KWMTBOMO08193	P_KWMTBOMO08193	1
274	2	2	3.125	3.125	3.125	P_KWMTBOMO08022	P_KWMTBOMO08022	P_KWMTBOMO08022	1
275	2	2	3.3640001	3.3640001	3.3640001	P_KWMTBOMO06763	P_KWMTBOMO06763	P_KWMTBOMO06763	1
276	2	2	2.4839999	2.4839999	2.4839999	P_KWMTBOMO06731	P_KWMTBOMO06731	P_KWMTBOMO06731	1
277	2	2	4.71	4.71	4.71	P_KWMTBOMO06621	P_KWMTBOMO06621	P_KWMTBOMO06621	1
278	2	2	9.2309996	9.2309996	9.2309996	P_KWMTBOMO06373	P_KWMTBOMO06373	P_KWMTBOMO06373	1
279	2	2	5.0719999	5.0719999	5.0719999	P_KWMTBOMO05975	P_KWMTBOMO05975	P_KWMTBOMO05975	1
280	2	2	2.107	2.107	2.107	P_KWMTBOMO05631	P_KWMTBOMO05631	P_KWMTBOMO05631	1
281	2	2	2.936	2.936	2.936	P_KWMTBOMO03631	P_KWMTBOMO03631	P_KWMTBOMO03631	1
282	2	2	9.5239997	9.5239997	9.5239997	P_KWMTBOMO03601	P_KWMTBOMO03601	P_KWMTBOMO03601	1
283	2	2	5.7250001	5.7250001	5.7250001	P_KWMTBOMO03550	P_KWMTBOMO03550	P_KWMTBOMO03550	1
284	2	2	7.6609999	7.6609999	7.6609999	P_KWMTBOMO02460	P_KWMTBOMO02460	P_KWMTBOMO02460	2
285	2	2	11.11	11.11	11.11	P_KWMTBOMO02431	P_KWMTBOMO02431	P_KWMTBOMO02431	1
286	2	2	6.024	6.024	6.024	P_KWMTBOMO02185	P_KWMTBOMO02185	P_KWMTBOMO02185	1
287	1.92	2	7.48	5.5119999	5.5119999	P_KWMTBOMO11558	P_KWMTBOMO11558	P_KWMTBOMO11558	1
288	1.92	1.92	1.7689999	1.7689999	1.7689999	P_KWMTBOMO03122	P_KWMTBOMO03122	P_KWMTBOMO03122	1
289	1.89	1.89	1.567	1.567	1.567	P_KWMTBOMO13235	P_KWMTBOMO13235	P_KWMTBOMO13235	1
290	1.88	1.88	20.59	8.3329998	8.3329998	P_KWMTBOMO03314	P_KWMTBOMO03314	P_KWMTBOMO03314	2
291	1.85	1.85	2.844	1.7379999	1.7379999	P_KWMTBOMO06803	P_KWMTBOMO06803	P_KWMTBOMO06803	1
292	1.85	1.85	2.111	2.111	2.111	P_KWMTBOMO07076	P_KWMTBOMO07076	P_KWMTBOMO07076	1
293	1.8	1.8	4.0460002	4.0460002	4.0460002	P_KWMTBOMO00545	P_KWMTBOMO00545	P_KWMTBOMO00545	1
294	1.77	1.77	12.26	8.602	3.6559999	P_KWMTBOMO14761	P_KWMTBOMO14761	P_KWMTBOMO14761	2
295	1.74	1.74	8.2570001	8.2570001	8.2570001	P_KWMTBOMO15592	P_KWMTBOMO15592	P_KWMTBOMO15592	1
296	1.7	1.7	8.2149997	4.8160002	4.8160002	P_KWMTBOMO14877	P_KWMTBOMO14877	P_KWMTBOMO14877	1
297	1.7	1.7	4.228	2.114	2.114	P_KWMTBOMO10169	P_KWMTBOMO10169	P_KWMTBOMO10169	1
298	1.68	1.68	10.63	2.5319999	2.5319999	P_KWMTBOMO08310	P_KWMTBOMO08310	P_KWMTBOMO08310	1
299	1.66	1.66	8.3719999	8.3719999	8.3719999	P_KWMTBOMO06076	P_KWMTBOMO06076	P_KWMTBOMO06076	1
300	1.62	1.62	6.3600004	2.4119999	2.4119999	P_KWMTBOMO13287	P_KWMTBOMO13287	P_KWMTBOMO13287	1
301	1.62	1.62	6.6229999	6.6229999	6.6229999	P_KWMTBOMO09000	P_KWMTBOMO09000	P_KWMTBOMO09000	1
302	1.6	1.6	1.475	0.9587	0.9587	P_KWMTBOMO09433	P_KWMTBOMO09433	P_KWMTBOMO09433	1
303	1.59	1.59	14.71	14.71	14.71	P_KWMTBOMO07600	P_KWMTBOMO07600	P_KWMTBOMO07600	1
304	1.57	1.57	15.04	9.7350001	9.7350001	P_KWMTBOMO08961	P_KWMTBOMO08961	P_KWMTBOMO08961	1
305	1.57	1.57	7.0370004	4.4440001	4.4440001	P_KWMTBOMO08201	P_KWMTBOMO08201	P_KWMTBOMO08201	1
306	1.55	1.57	5.7890002	5.7890002	5.7890002	P_KWMTBOMO14024	P_KWMTBOMO14024	P_KWMTBOMO14024	1
307	1.51	1.51	8.9110002	3.96	3.96	P_KWMTBOMO02857	P_KWMTBOMO02857	P_KWMTBOMO02857	1
308	1.51	1.51	4.8950002	4.8950002	4.8950002	P_KWMTBOMO05700	P_KWMTBOMO05700	P_KWMTBOMO05700	1
309	1.49	1.49	16.82	16.82	10.28	P_KWMTBOMO10128	P_KWMTBOMO10128	P_KWMTBOMO10128	1
310	1.48	1.48	14.71	10.78	5.3920001	P_KWMTBOMO01502	P_KWMTBOMO01502	P_KWMTBOMO01502	1
311	1.48	1.48	1.252	1.252	1.252	P_KWMTBOMO04768	P_KWMTBOMO04768	P_KWMTBOMO04768	1
312	1.46	1.46	11.72	11.72	11.72	P_KWMTBOMO00786	P_KWMTBOMO00786	P_KWMTBOMO00786	1
313	1.45	1.45	17.89	7.8950003	7.8950003	P_KWMTBOMO03113	P_KWMTBOMO03113	P_KWMTBOMO03113	1

314	1.45	1.45	12.61	12.61	6.7230001	P_KWMTBOMO01068	P_KWMTBOMO01068	P_KWMTBOMO01068	1
315	1.44	1.44	2.2390001	2.2390001	2.2390001	P_KWMTBOMO11689	P_KWMTBOMO11689	P_KWMTBOMO11689	1
316	1.43	1.43	25.170001	14.29	14.29	P_KWMTBOMO12483	P_KWMTBOMO12483	P_KWMTBOMO12483	2
317	1.42	1.42	4.101	4.101	4.101	P_KWMTBOMO03337	P_KWMTBOMO03337	P_KWMTBOMO03337	1
318	1.4	1.4	14	9.0000004	5.0000001	P_KWMTBOMO02477	P_KWMTBOMO02477	P_KWMTBOMO02477	1
319	1.4	1.4	1.472	1.472	1.472	P_KWMTBOMO14536	P_KWMTBOMO14536	P_KWMTBOMO14536	1
320	1.39	1.4	11.94	7.463	7.463	P_KWMTBOMO04691	P_KWMTBOMO04691	P_KWMTBOMO04691	1
321	1.37	1.37	3.3440001	1.726	1.079	P_KWMTBOMO04610	P_KWMTBOMO04610	P_KWMTBOMO04610	1
322	1.36	1.36	4.651	4.651	4.651	P_KWMTBOMO09390	P_KWMTBOMO09390	P_KWMTBOMO09390	1
323	1.33	1.33	3.0200001	1.6179999	1.6179999	P_KWMTBOMO15176	P_KWMTBOMO15176	P_KWMTBOMO15176	1
324	1.33	1.33	1.747	1.747	1.747	P_KWMTBOMO07679	P_KWMTBOMO07679	P_KWMTBOMO07679	1
325	1.32	1.32	1.203	0.3481	0.3481	P_KWMTBOMO05680	P_KWMTBOMO05680	P_KWMTBOMO05680	1

Summary of the identified PIPs of fibL_L5D5

N	Unused	Total	%Cov	%Cov(50)	%Cov(95)	Accession	Citable Accession	Name	Peptides(95%)
4	34.89	34.89	30.180001	25.9	24.1	P_KWMTBOMO00462	P_KWMTBOMO00462	P_KWMTBOMO00462	23
8	32.35	32.35	49.540001	37.329999	34.099999	P_KWMTBOMO06229	P_KWMTBOMO06229	P_KWMTBOMO06229	21
21	23.47	23.47	24.3	18.9	14.1	P_KWMTBOMO13293	P_KWMTBOMO13293	P_KWMTBOMO13293	14
22	22.93	22.93	50.629997	50.629997	48.74	P_KWMTBOMO05178	P_KWMTBOMO05178	P_KWMTBOMO05178	14
26	19.17	19.17	34.43	29.67	25.639999	P_KWMTBOMO09442	P_KWMTBOMO09442	P_KWMTBOMO09442	9
30	17.82	17.82	22.220001	18.03	18.03	P_KWMTBOMO00927	P_KWMTBOMO00927	P_KWMTBOMO00927	9
32	17.24	17.24	26.76	24.41	20.900001	P_KWMTBOMO04502	P_KWMTBOMO04502	P_KWMTBOMO04502	9
35	16.31	16.31	28.749999	27.520001	22.63	P_KWMTBOMO06763	P_KWMTBOMO06763	P_KWMTBOMO06763	10
36	15.87	15.87	36.809999	36.809999	25	P_KWMTBOMO09553	P_KWMTBOMO09553	P_KWMTBOMO09553	10
42	14.99	14.99	26.33	24.14	21.32	P_KWMTBOMO01148	P_KWMTBOMO01148	P_KWMTBOMO01148	11
43	14.93	14.93	14.569999	12.67	9.6809998	P_KWMTBOMO05541	P_KWMTBOMO05541	P_KWMTBOMO05541	8
44	14.85	14.85	6.9399998	4.8909999	3.303	P_KWMTBOMO08648	P_KWMTBOMO08648	P_KWMTBOMO08648	6
45	14.76	14.76	18.94	16.670001	12.95	P_KWMTBOMO15357	P_KWMTBOMO15357	P_KWMTBOMO15357	7
48	14.43	14.43	23.540001	16.949999	16.949999	P_KWMTBOMO09808	P_KWMTBOMO09808	P_KWMTBOMO09808	9
49	14.37	14.37	32.780001	32.780001	29.139999	P_KWMTBOMO12389	P_KWMTBOMO12389	P_KWMTBOMO12389	7
50	14.27	14.27	9.9009998	8.828	7.5910002	P_KWMTBOMO12628	P_KWMTBOMO12628	P_KWMTBOMO12628	8
53	14.09	14.09	17.569999	11.56	8.7860003	P_KWMTBOMO06595	P_KWMTBOMO06595	P_KWMTBOMO06595	7
54	13.77	13.77	23.34	18.64	11.83	P_KWMTBOMO11096	P_KWMTBOMO11096	P_KWMTBOMO11096	7
55	13.54	13.54	20.94	19.87	18.38	P_KWMTBOMO02470	P_KWMTBOMO02470	P_KWMTBOMO02470	7
59	12.85	12.85	59.600002	47.679999	43.05	P_KWMTBOMO08440	P_KWMTBOMO08440	P_KWMTBOMO08440	6
60	12.85	12.85	19.850001	19.850001	16.7	P_KWMTBOMO01075	P_KWMTBOMO01075	P_KWMTBOMO01075	7
61	12.74	12.74	17.16	14.219999	12.36	P_KWMTBOMO13370	P_KWMTBOMO13370	P_KWMTBOMO13370	9
62	12.7	12.7	13.940001	8.405	6.8769999	P_KWMTBOMO09055	P_KWMTBOMO09055	P_KWMTBOMO09055	7
65	12.2	12.2	35.67	31.999999	23.67	P_KWMTBOMO14732	P_KWMTBOMO14732	P_KWMTBOMO14732	7
70	11.95	11.95	25.229999	16.760001	12.61	P_KWMTBOMO03626	P_KWMTBOMO03626	P_KWMTBOMO03626	7
76	11.07	11.07	37.630001	37.630001	25.260001	P_KWMTBOMO11255	P_KWMTBOMO11255	P_KWMTBOMO11255	6
78	10.88	10.88	11.47	9.4010003	7.9549998	P_KWMTBOMO07589	P_KWMTBOMO07589	P_KWMTBOMO07589	6
80	10.72	10.72	36.070001	33.329999	33.329999	P_KWMTBOMO06480	P_KWMTBOMO06480	P_KWMTBOMO06480	7
82	10.5	10.5	45.950001	37.39	37.39	P_KWMTBOMO01424	P_KWMTBOMO01424	P_KWMTBOMO01424	9
83	10.45	10.45	8.1979997	7.4429996	5.9330001	P_KWMTBOMO04610	P_KWMTBOMO04610	P_KWMTBOMO04610	5
84	10.36	10.36	14.29	9.7560003	6.7359999	P_KWMTBOMO00427	P_KWMTBOMO00427	P_KWMTBOMO00427	5
85	10.3	10.3	26.320001	19.74	19.74	P_KWMTBOMO01720	P_KWMTBOMO01720	P_KWMTBOMO01720	6
86	10.1	10.1	13.689999	7.158	7.158	P_KWMTBOMO05741	P_KWMTBOMO05741	P_KWMTBOMO05741	5
88	10.07	10.07	20.34	13.99	13.99	P_KWMTBOMO07855	P_KWMTBOMO07855	P_KWMTBOMO07855	6

90	9.69	9.69	23.100001	16.670001	13.81	P_KWMTBOMO02081	P_KWMTBOMO02081	P_KWMTBOMO02081	6
91	9.44	9.44	18.170001	16.509999	12.29	P_KWMTBOMO13721	P_KWMTBOMO13721	P_KWMTBOMO13721	5
92	9.34	9.34	27.419999	21.510001	17.49	P_KWMTBOMO00375	P_KWMTBOMO00375	P_KWMTBOMO00375	4
93	9.08	9.08	27.790001	18.73	16.01	P_KWMTBOMO07103	P_KWMTBOMO07103	P_KWMTBOMO07103	5
95	9.03	9.03	6.5750003	5.8449998	5.8449998	P_KWMTBOMO04906	P_KWMTBOMO04906	P_KWMTBOMO04906	5
96	9	9	10.58	5.13	4.4199999	P_KWMTBOMO03067	P_KWMTBOMO03067	P_KWMTBOMO03067	5
97	8.87	8.87	41.580001	38.609999	30.199999	P_KWMTBOMO15361	P_KWMTBOMO15361	P_KWMTBOMO15361	6
99	8.8	8.8	13.22	9.5370002	7.9020001	P_KWMTBOMO03430	P_KWMTBOMO03430	P_KWMTBOMO03430	4
100	8.63	8.63	29.409999	23.53	23.53	P_KWMTBOMO10248	P_KWMTBOMO10248	P_KWMTBOMO10248	7
101	8.61	8.61	8.5919999	5.8499999	4.8450001	P_KWMTBOMO09183	P_KWMTBOMO09183	P_KWMTBOMO09183	4
102	8.42	8.42	6.5760002	4.1299999	2.6629999	P_KWMTBOMO12285	P_KWMTBOMO12285	P_KWMTBOMO12285	6
103	8.39	8.39	26.570001	21.68	21.68	P_KWMTBOMO08666	P_KWMTBOMO08666	P_KWMTBOMO08666	5
104	8.37	8.37	53.100002	53.100002	53.100002	P_KWMTBOMO08961	P_KWMTBOMO08961	P_KWMTBOMO08961	5
105	8.33	8.83	19.35	16.13	12.69	P_KWMTBOMO14761	P_KWMTBOMO14761	P_KWMTBOMO14761	5
106	8.29	8.29	35.02	31.34	24.420001	P_KWMTBOMO09232	P_KWMTBOMO09232	P_KWMTBOMO09232	5
110	8.02	8.02	8.1890002	6.0690001	4.6239998	P_KWMTBOMO04768	P_KWMTBOMO04768	P_KWMTBOMO04768	4
111	8.01	8.01	11.09	7.2930001	7.2930001	P_KWMTBOMO00722	P_KWMTBOMO00722	P_KWMTBOMO00722	6
112	8	8	10.57	10.57	10.57	P_KWMTBOMO02835	P_KWMTBOMO02835	P_KWMTBOMO02835	4
113	7.98	7.98	10.31	9.1729999	7.1350001	P_KWMTBOMO08045	P_KWMTBOMO08045	P_KWMTBOMO08045	4
114	7.78	7.78	9.482	3.128	2.493	P_KWMTBOMO12878	P_KWMTBOMO12878	P_KWMTBOMO12878	4
115	7.74	7.74	15.69	15.69	15.69	P_KWMTBOMO10916	P_KWMTBOMO10916	P_KWMTBOMO10916	4
117	7.43	8	28.240001	13.93	10.69	P_KWMTBOMO12560	P_KWMTBOMO12560	P_KWMTBOMO12560	4
118	7.4	7.4	65.179998	65.179998	65.179998	P_KWMTBOMO09230	P_KWMTBOMO09230	P_KWMTBOMO09230	4
119	7.38	7.38	24.41	24.41	20.66	P_KWMTBOMO16481	P_KWMTBOMO16481	P_KWMTBOMO16481	4
120	7.35	7.35	17.1	13.97	10.29	P_KWMTBOMO10337	P_KWMTBOMO10337	P_KWMTBOMO10337	3
121	7.32	7.32	16.11	12.39	10.27	P_KWMTBOMO08578	P_KWMTBOMO08578	P_KWMTBOMO08578	4
122	7.23	7.23	33.109999	33.109999	22.52	P_KWMTBOMO13960	P_KWMTBOMO13960	P_KWMTBOMO13960	3
123	7.13	7.13	7.8730002	6.0770001	6.0770001	P_KWMTBOMO15359	P_KWMTBOMO15359	P_KWMTBOMO15359	4
124	7.04	7.04	20.94	17.88	14.35	P_KWMTBOMO09415	P_KWMTBOMO09415	P_KWMTBOMO09415	6
125	7.03	7.03	18.82	17.53	7.0110001	P_KWMTBOMO14887	P_KWMTBOMO14887	P_KWMTBOMO14887	4
126	6.98	6.98	12.729999	11.25	7.0110001	P_KWMTBOMO00793	P_KWMTBOMO00793	P_KWMTBOMO00793	4
127	6.93	6.93	18.6	18.6	18.6	P_KWMTBOMO02278	P_KWMTBOMO02278	P_KWMTBOMO02278	4
128	6.73	6.73	3.8740002	2.407	1.5799999	P_KWMTBOMO06045	P_KWMTBOMO06045	P_KWMTBOMO06045	3
129	6.65	6.65	3.6830001	1.7720001	1.39	P_KWMTBOMO07761	P_KWMTBOMO07761	P_KWMTBOMO07761	3
131	6.42	6.42	17.98	13.9	9.5370002	P_KWMTBOMO04370	P_KWMTBOMO04370	P_KWMTBOMO04370	3
133	6.22	6.22	18.59	18.59	12.18	P_KWMTBOMO16357	P_KWMTBOMO16357	P_KWMTBOMO16357	3
134	6.21	6.82	11.36	7.4069999	5.3089999	P_KWMTBOMO12844	P_KWMTBOMO12844	P_KWMTBOMO12844	3
135	6.14	6.14	9.0230003	3.8600001	2.3560001	P_KWMTBOMO08584	P_KWMTBOMO08584	P_KWMTBOMO08584	3
136	6.13	6.14	8.2220003	8.2220003	6.3100003	P_KWMTBOMO12264	P_KWMTBOMO12264	P_KWMTBOMO12264	3
137	6.08	6.08	14.61	12.94	9.1859996	P_KWMTBOMO08956	P_KWMTBOMO08956	P_KWMTBOMO08956	3
138	6.04	6.04	12.75	8.5019998	8.5019998	P_KWMTBOMO11680	P_KWMTBOMO11680	P_KWMTBOMO11680	4
139	6.03	6.03	12.13	10.07	10.07	P_KWMTBOMO15123	P_KWMTBOMO15123	P_KWMTBOMO15123	3
141	6.02	6.02	29.390001	24.73	24.73	P_KWMTBOMO06427	P_KWMTBOMO06427	P_KWMTBOMO06427	4
142	6	6.14	48.74	36.129999	31.09	P_KWMTBOMO15114	P_KWMTBOMO15114	P_KWMTBOMO15114	4
144	5.93	5.93	13.04	7.5779997	6.0869999	P_KWMTBOMO11666	P_KWMTBOMO11666	P_KWMTBOMO11666	4
145	5.84	5.84	21.72	11.62	11.62	P_KWMTBOMO15518	P_KWMTBOMO15518	P_KWMTBOMO15518	4
146	5.82	5.82	37.86	18.57	18.57	P_KWMTBOMO00838	P_KWMTBOMO00838	P_KWMTBOMO00838	4
148	5.7	5.7	5.9239998	1.382	0.6665	P_KWMTBOMO12602	P_KWMTBOMO12602	P_KWMTBOMO12602	2

150	5.63	5.63	27.880001	21.15	14.42	P_KWMTBOMO06974	P_KWMTBOMO06974	P_KWMTBOMO06974	3
151	5.51	5.51	12.96	12.96	12.96	P_KWMTBOMO08655	P_KWMTBOMO08655	P_KWMTBOMO08655	4
153	5.44	5.44	5.9319999	4.2369999	2.881	P_KWMTBOMO09542	P_KWMTBOMO09542	P_KWMTBOMO09542	3
154	5.41	5.41	14.07	9.9440001	5.6290001	P_KWMTBOMO06770	P_KWMTBOMO06770	P_KWMTBOMO06770	3
155	5.4	5.4	21.05	16.410001	11.46	P_KWMTBOMO03986	P_KWMTBOMO03986	P_KWMTBOMO03986	3
156	5.4	5.4	10.09	8.3690003	8.3690003	P_KWMTBOMO02500	P_KWMTBOMO02500	P_KWMTBOMO02500	3
157	5.36	5.36	26.47	25.979999	20.100001	P_KWMTBOMO02734	P_KWMTBOMO02734	P_KWMTBOMO02734	3
158	5.19	5.19	10.14	6.0430001	6.0430001	P_KWMTBOMO04170	P_KWMTBOMO04170	P_KWMTBOMO04170	3
159	5.19	5.19	11.72	9.4520003	7.5609997	P_KWMTBOMO01606	P_KWMTBOMO01606	P_KWMTBOMO01606	3
160	5.16	5.16	20.209999	16.580001	16.580001	P_KWMTBOMO05281	P_KWMTBOMO05281	P_KWMTBOMO05281	4
161	5.11	5.11	33.329999	33.329999	27.450001	P_KWMTBOMO08820	P_KWMTBOMO08820	P_KWMTBOMO08820	4
162	5.08	5.08	12.74	12.74	10.51	P_KWMTBOMO15758	P_KWMTBOMO15758	P_KWMTBOMO15758	3
163	5.04	5.04	9.2390001	8.0430001	8.0430001	P_KWMTBOMO07723	P_KWMTBOMO07723	P_KWMTBOMO07723	4
164	4.99	4.99	16.09	12.6	5.2329998	P_KWMTBOMO06354	P_KWMTBOMO06354	P_KWMTBOMO06354	2
165	4.97	4.97	17.51	17.51	17.51	P_KWMTBOMO08596	P_KWMTBOMO08596	P_KWMTBOMO08596	3
166	4.91	4.91	29.949999	22.99	18.719999	P_KWMTBOMO12428	P_KWMTBOMO12428	P_KWMTBOMO12428	5
168	4.89	4.89	15.3	8.0219999	8.0219999	P_KWMTBOMO03311	P_KWMTBOMO03311	P_KWMTBOMO03311	3
169	4.76	4.76	8.4579997	5.3210001	4.6379998	P_KWMTBOMO03177	P_KWMTBOMO03177	P_KWMTBOMO03177	3
170	4.75	4.75	16.46	16.46	16.46	P_KWMTBOMO14479	P_KWMTBOMO14479	P_KWMTBOMO14479	2
171	4.64	4.64	8.2220003	8.2220003	6.222	P_KWMTBOMO10283	P_KWMTBOMO10283	P_KWMTBOMO10283	2
172	4.58	4.58	38.420001	38.420001	26.840001	P_KWMTBOMO03113	P_KWMTBOMO03113	P_KWMTBOMO03113	3
173	4.58	4.58	32.679999	22.88	22.88	P_KWMTBOMO11021	P_KWMTBOMO11021	P_KWMTBOMO11021	3
174	4.52	4.52	20.33	14.839999	11.54	P_KWMTBOMO04529	P_KWMTBOMO04529	P_KWMTBOMO04529	3
175	4.46	4.46	17.28	17.28	9.877	P_KWMTBOMO04168	P_KWMTBOMO04168	P_KWMTBOMO04168	2
176	4.45	4.45	19.63	19.63	19.63	P_KWMTBOMO11448	P_KWMTBOMO11448	P_KWMTBOMO11448	3
177	4.44	4.44	8.6709999	7.2729997	7.2729997	P_KWMTBOMO15060	P_KWMTBOMO15060	P_KWMTBOMO15060	3
178	4.41	4.41	13.73	8.0090001	5.7209998	P_KWMTBOMO06660	P_KWMTBOMO06660	P_KWMTBOMO06660	2
179	4.4	4.4	9.0729997	5.085	2.991	P_KWMTBOMO04051	P_KWMTBOMO04051	P_KWMTBOMO04051	3
180	4.4	4.4	13.72	10.03	7.3880002	P_KWMTBOMO05258	P_KWMTBOMO05258	P_KWMTBOMO05258	2
182	4.3	4.3	30.500001	17.5	13.500001	P_KWMTBOMO02477	P_KWMTBOMO02477	P_KWMTBOMO02477	2
183	4.28	4.28	8.647	8.647	6.2029999	P_KWMTBOMO03722	P_KWMTBOMO03722	P_KWMTBOMO03722	2
185	4.2	4.2	10.75	8.602	6.2369999	P_KWMTBOMO05564	P_KWMTBOMO05564	P_KWMTBOMO05564	2
188	4.12	4.12	14.049999	8.5029997	5.3599998	P_KWMTBOMO06999	P_KWMTBOMO06999	P_KWMTBOMO06999	2
189	4.12	4.12	17.18	17.18	11.45	P_KWMTBOMO00837	P_KWMTBOMO00837	P_KWMTBOMO00837	2
191	4.04	4.04	8.7480001	8.7480001	5.9640002	P_KWMTBOMO08204	P_KWMTBOMO08204	P_KWMTBOMO08204	2
192	4.03	4.03	15.539999	15.539999	8.7839998	P_KWMTBOMO16197	P_KWMTBOMO16197	P_KWMTBOMO16197	2
193	4.03	4.03	13.850001	11.03	11.03	P_KWMTBOMO16309	P_KWMTBOMO16309	P_KWMTBOMO16309	2
194	4.03	4.03	14.62	12.31	12.31	P_KWMTBOMO13405	P_KWMTBOMO13405	P_KWMTBOMO13405	2
195	4.02	4.02	8.4890001	5.3830002	5.3830002	P_KWMTBOMO00473	P_KWMTBOMO00473	P_KWMTBOMO00473	2
196	4	4	7.3530003	3.743	3.743	P_KWMTBOMO16130	P_KWMTBOMO16130	P_KWMTBOMO16130	2
197	4	4	6.3349999	3.6200002	3.6200002	P_KWMTBOMO11316	P_KWMTBOMO11316	P_KWMTBOMO11316	2
198	4	4	8.4969997	5.7190001	5.7190001	P_KWMTBOMO10779	P_KWMTBOMO10779	P_KWMTBOMO10779	2
200	4	4	5.5070002	5.5070002	3.3330001	P_KWMTBOMO14549	P_KWMTBOMO14549	P_KWMTBOMO14549	2
201	4	4	13.53	11.59	11.59	P_KWMTBOMO08106	P_KWMTBOMO08106	P_KWMTBOMO08106	2
202	4	4	7.3789999	7.3789999	7.3789999	P_KWMTBOMO02392	P_KWMTBOMO02392	P_KWMTBOMO02392	2
203	4	4	41.38	41.38	41.38	P_KWMTBOMO13722	P_KWMTBOMO13722	P_KWMTBOMO13722	2
204	4	4	35.620001	35.620001	35.620001	P_KWMTBOMO08098	P_KWMTBOMO08098	P_KWMTBOMO08098	2
205	4	4	4.383	4.383	4.383	P_KWMTBOMO02535	P_KWMTBOMO02535	P_KWMTBOMO02535	2

206	4	4	16.850001	16.850001	16.850001	P_KWMTBOMO00860	P_KWMTBOMO00860	P_KWMTBOMO00860	2
207	3.96	3.96	9.3099996	6.3790001	4.3099999	P_KWMTBOMO02088	P_KWMTBOMO02088	P_KWMTBOMO02088	2
208	3.96	3.96	6.4939998	6.4939998	6.4939998	P_KWMTBOMO14041	P_KWMTBOMO14041	P_KWMTBOMO14041	2
209	3.92	3.92	5.8820002	4.2410001	4.2410001	P_KWMTBOMO01423	P_KWMTBOMO01423	P_KWMTBOMO01423	2
210	3.91	3.91	18.51	6.9410004	4.8840001	P_KWMTBOMO05464	P_KWMTBOMO05464	P_KWMTBOMO05464	2
211	3.87	3.87	28.95	20.53	10.53	P_KWMTBOMO12018	P_KWMTBOMO12018	P_KWMTBOMO12018	2
212	3.85	3.85	17.839999	17.839999	9.8590001	P_KWMTBOMO05566	P_KWMTBOMO05566	P_KWMTBOMO05566	2
213	3.82	3.82	39.289999	39.289999	29.76	P_KWMTBOMO03951	P_KWMTBOMO03951	P_KWMTBOMO03951	3
214	3.75	3.75	10.81	6.7570001	6.7570001	P_KWMTBOMO15584	P_KWMTBOMO15584	P_KWMTBOMO15584	2
215	3.75	3.75	31.54	31.54	24.62	P_KWMTBOMO07368	P_KWMTBOMO07368	P_KWMTBOMO07368	2
216	3.73	3.73	26.04	10.14	7.3729999	P_KWMTBOMO01114	P_KWMTBOMO01114	P_KWMTBOMO01114	2
217	3.7	3.7	12.32	12.32	12.32	P_KWMTBOMO00794	P_KWMTBOMO00794	P_KWMTBOMO00794	2
218	3.68	3.68	2.734	2.734	2.734	P_KWMTBOMO10493	P_KWMTBOMO10493	P_KWMTBOMO10493	2
219	3.62	3.62	14.650001	5.4919999	5.4919999	P_KWMTBOMO14530	P_KWMTBOMO14530	P_KWMTBOMO14530	2
220	3.62	3.62	2.8139999	2.8139999	2.8139999	P_KWMTBOMO10780	P_KWMTBOMO10780	P_KWMTBOMO10780	2
221	3.6	3.6	31.079999	22.97	16.22	P_KWMTBOMO08064	P_KWMTBOMO08064	P_KWMTBOMO08064	2
222	3.6	3.6	10.22	10.22	10.22	P_KWMTBOMO10129	P_KWMTBOMO10129	P_KWMTBOMO10129	2
223	3.57	3.57	8.7760001	6.4669997	6.4669997	P_KWMTBOMO04605	P_KWMTBOMO04605	P_KWMTBOMO04605	2
224	3.56	3.56	6.3259996	4.8659999	2.6760001	P_KWMTBOMO00390	P_KWMTBOMO00390	P_KWMTBOMO00390	2
225	3.52	3.52	2.729	1.287	1.287	P_KWMTBOMO12304	P_KWMTBOMO12304	P_KWMTBOMO12304	2
226	3.51	3.51	12.5	12.5	12.5	P_KWMTBOMO09672	P_KWMTBOMO09672	P_KWMTBOMO09672	2
227	3.46	3.46	10.06	8.1009999	5.587	P_KWMTBOMO14715	P_KWMTBOMO14715	P_KWMTBOMO14715	3
228	3.43	3.43	11.44	6.7450002	6.7450002	P_KWMTBOMO09474	P_KWMTBOMO09474	P_KWMTBOMO09474	2
229	3.39	3.39	18.92	18.92	18.92	P_KWMTBOMO14440	P_KWMTBOMO14440	P_KWMTBOMO14440	2
230	3.39	3.39	5.1460002	5.1460002	5.1460002	P_KWMTBOMO03301	P_KWMTBOMO03301	P_KWMTBOMO03301	2
231	3.36	3.36	5.2700002	1.7340001	1.7340001	P_KWMTBOMO04676	P_KWMTBOMO04676	P_KWMTBOMO04676	2
232	3.36	3.36	3.6249999	2.452	2.452	P_KWMTBOMO12667	P_KWMTBOMO12667	P_KWMTBOMO12667	2
233	3.31	3.31	10.45	7.463	7.463	P_KWMTBOMO11416	P_KWMTBOMO11416	P_KWMTBOMO11416	2
234	3.22	3.22	12.61	12.61	12.61	P_KWMTBOMO01068	P_KWMTBOMO01068	P_KWMTBOMO01068	2
235	3.19	3.21	12.86	11.19	7.1429998	P_KWMTBOMO16204	P_KWMTBOMO16204	P_KWMTBOMO16204	2
236	3.13	6.64	11.82	7.4160002	6.4889997	P_KWMTBOMO14157	P_KWMTBOMO14157	P_KWMTBOMO14157	4
237	3.1	3.1	10.14	7.6920003	7.6920003	P_KWMTBOMO03860	P_KWMTBOMO03860	P_KWMTBOMO03860	2
238	3.05	3.05	6.7469999	6.7469999	6.7469999	P_KWMTBOMO10965	P_KWMTBOMO10965	P_KWMTBOMO10965	2
239	2.98	2.98	19.599999	16	12.4	P_KWMTBOMO04427	P_KWMTBOMO04427	P_KWMTBOMO04427	2
240	2.97	2.97	13.150001	10.36	10.36	P_KWMTBOMO11741	P_KWMTBOMO11741	P_KWMTBOMO11741	2
241	2.96	2.96	3.974	2.0810001	2.0810001	P_KWMTBOMO01790	P_KWMTBOMO01790	P_KWMTBOMO01790	2
242	2.95	2.95	20.51	20.51	3.8460001	P_KWMTBOMO15665	P_KWMTBOMO15665	P_KWMTBOMO15665	1
243	2.86	2.86	9.8180003	5.61	3.647	P_KWMTBOMO11336	P_KWMTBOMO11336	P_KWMTBOMO11336	2
244	2.85	2.85	13.75	13.75	13.75	P_KWMTBOMO07772	P_KWMTBOMO07772	P_KWMTBOMO07772	2
245	2.82	2.82	28.79	18.179999	18.179999	P_KWMTBOMO01055	P_KWMTBOMO01055	P_KWMTBOMO01055	3
246	2.81	2.81	5.0140001	4.1779999	4.1779999	P_KWMTBOMO10358	P_KWMTBOMO10358	P_KWMTBOMO10358	2
247	2.81	2.81	15.530001	15.530001	15.530001	P_KWMTBOMO07061	P_KWMTBOMO07061	P_KWMTBOMO07061	2
248	2.78	2.78	9.6510001	6.7019999	6.7019999	P_KWMTBOMO06231	P_KWMTBOMO06231	P_KWMTBOMO06231	2
249	2.76	2.76	17.28	17.28	17.28	P_KWMTBOMO03622	P_KWMTBOMO03622	P_KWMTBOMO03622	2
250	2.74	2.74	20.09	13.240001	9.1320001	P_KWMTBOMO08894	P_KWMTBOMO08894	P_KWMTBOMO08894	2
251	2.73	2.74	10.1	4.1579999	4.1579999	P_KWMTBOMO07374	P_KWMTBOMO07374	P_KWMTBOMO07374	2
252	2.7	2.7	1.276	1.276	1.276	P_KWMTBOMO10397	P_KWMTBOMO10397	P_KWMTBOMO10397	1
253	2.58	2.58	26.87	17.910001	10.45	P_KWMTBOMO14639	P_KWMTBOMO14639	P_KWMTBOMO14639	1

254	2.53	2.53	18.15	6.9499999	4.2470001	P_KWMTBOMO04469	P_KWMTBOMO04469	P_KWMTBOMO04469	1
255	2.51	2.51	5.3879999	5.3879999	2.802	P_KWMTBOMO00373	P_KWMTBOMO00373	P_KWMTBOMO00373	1
256	2.49	2.49	9.307	9.307	2.8139999	P_KWMTBOMO15547	P_KWMTBOMO15547	P_KWMTBOMO15547	1
257	2.48	2.49	19.69	8.2680002	8.2680002	P_KWMTBOMO04594	P_KWMTBOMO04594	P_KWMTBOMO04594	2
258	2.46	2.46	3.2760002	2.5	1.207	P_KWMTBOMO01806	P_KWMTBOMO01806	P_KWMTBOMO01806	1
259	2.44	2.44	3.2299999	3.2299999	1.545	P_KWMTBOMO13226	P_KWMTBOMO13226	P_KWMTBOMO13226	1
260	2.42	2.42	18.970001	18.970001	13.330001	P_KWMTBOMO09355	P_KWMTBOMO09355	P_KWMTBOMO09355	2
261	2.41	2.41	9.0400003	4.896	2.448	P_KWMTBOMO02994	P_KWMTBOMO02994	P_KWMTBOMO02994	1
262	2.4	2.4	3.272	2.4390001	1.368	P_KWMTBOMO09747	P_KWMTBOMO09747	P_KWMTBOMO09747	3
263	2.36	2.36	21.85	21.01	15.970001	P_KWMTBOMO12706	P_KWMTBOMO12706	P_KWMTBOMO12706	2
265	2.35	2.35	8.0389999	7.0739999	2.251	P_KWMTBOMO02432	P_KWMTBOMO02432	P_KWMTBOMO02432	1
266	2.34	2.34	5.8510002	3.59	2.3940001	P_KWMTBOMO14942	P_KWMTBOMO14942	P_KWMTBOMO14942	1
267	2.24	2.24	18.84	6.5329999	3.015	P_KWMTBOMO12166	P_KWMTBOMO12166	P_KWMTBOMO12166	1
268	2.24	2.24	11.85	9.9529997	7.3459998	P_KWMTBOMO08503	P_KWMTBOMO08503	P_KWMTBOMO08503	2
269	2.2	2.2	4.016	2.547	1.567	P_KWMTBOMO13235	P_KWMTBOMO13235	P_KWMTBOMO13235	1
270	2.14	2.14	7.0869997	2.6769999	1.89	P_KWMTBOMO08773	P_KWMTBOMO08773	P_KWMTBOMO08773	1
271	2.13	2.13	15.710001	3.3330001	2.3809999	P_KWMTBOMO07205	P_KWMTBOMO07205	P_KWMTBOMO07205	1
272	2.13	2.13	16.060001	10.95	6.9339998	P_KWMTBOMO01796	P_KWMTBOMO01796	P_KWMTBOMO01796	1
273	2.11	2.11	8.2570001	3.8989998	2.523	P_KWMTBOMO05987	P_KWMTBOMO05987	P_KWMTBOMO05987	1
274	2.11	2.11	14.040001	14.040001	8.427	P_KWMTBOMO05078	P_KWMTBOMO05078	P_KWMTBOMO05078	1
275	2.09	2.09	18.440001	9.8360002	4.9180001	P_KWMTBOMO10310	P_KWMTBOMO10310	P_KWMTBOMO10310	1
276	2.09	2.09	5.263	5.263	1.815	P_KWMTBOMO14936	P_KWMTBOMO14936	P_KWMTBOMO14936	1
277	2.09	2.09	19.419999	19.419999	11.65	P_KWMTBOMO11742	P_KWMTBOMO11742	P_KWMTBOMO11742	1
278	2.09	2.09	23.53	23.53	14.29	P_KWMTBOMO04674	P_KWMTBOMO04674	P_KWMTBOMO04674	1
279	2.07	2.07	28.920001	5.8820002	5.8820002	P_KWMTBOMO01273	P_KWMTBOMO01273	P_KWMTBOMO01273	1
280	2.06	2.06	3.641	1.56	1.56	P_KWMTBOMO15101	P_KWMTBOMO15101	P_KWMTBOMO15101	1
281	2.05	2.05	1.528	0.3354	0.3354	P_KWMTBOMO11570	P_KWMTBOMO11570	P_KWMTBOMO11570	1
282	2.04	2.04	7.0689999	2.069	2.069	P_KWMTBOMO11590	P_KWMTBOMO11590	P_KWMTBOMO11590	1
283	2.04	2.04	12.59	3.7999999	3.7999999	P_KWMTBOMO08893	P_KWMTBOMO08893	P_KWMTBOMO08893	1
284	2.04	2.04	16.339999	11.39	5.9409998	P_KWMTBOMO07722	P_KWMTBOMO07722	P_KWMTBOMO07722	1
285	2.02	2.02	15.24	4.5729998	4.5729998	P_KWMTBOMO06082	P_KWMTBOMO06082	P_KWMTBOMO06082	1
286	2.02	2.02	7.265	2.3499999	2.3499999	P_KWMTBOMO13729	P_KWMTBOMO13729	P_KWMTBOMO13729	1
287	2.02	2.02	13.14	8.5709997	8.5709997	P_KWMTBOMO16319	P_KWMTBOMO16319	P_KWMTBOMO16319	1
288	2.02	2.02	34.740001	34.740001	20	P_KWMTBOMO14478	P_KWMTBOMO14478	P_KWMTBOMO14478	1
289	2.01	2.01	11.38	1.8209999	1.8209999	P_KWMTBOMO14240	P_KWMTBOMO14240	P_KWMTBOMO14240	1
290	2.01	2.01	11.17	6.1450001	2.421	P_KWMTBOMO07323	P_KWMTBOMO07323	P_KWMTBOMO07323	1
291	2.01	2.01	4.259	1.508	1.508	P_KWMTBOMO01821	P_KWMTBOMO01821	P_KWMTBOMO01821	1
292	2.01	2.01	6.5930001	3.077	3.077	P_KWMTBOMO15781	P_KWMTBOMO15781	P_KWMTBOMO15781	1
293	2.01	2.01	18.48	5.0719999	5.0719999	P_KWMTBOMO05975	P_KWMTBOMO05975	P_KWMTBOMO05975	1
294	2.01	2.01	8.6960003	8.6960003	5.3509999	P_KWMTBOMO11061	P_KWMTBOMO11061	P_KWMTBOMO11061	1
295	2.01	2.01	2.0129999	1.151	1.151	P_KWMTBOMO04806	P_KWMTBOMO04806	P_KWMTBOMO04806	1
296	2	4	4.3820001	2.39	2.39	P_KWMTBOMO01817	P_KWMTBOMO01817	P_KWMTBOMO01817	2
297	2	2.02	4.4149999	3.091	3.091	P_KWMTBOMO04737	P_KWMTBOMO04737	P_KWMTBOMO04737	1
298	2	2	8.072	1.682	1.682	P_KWMTBOMO09657	P_KWMTBOMO09657	P_KWMTBOMO09657	1
299	2	2	11.99	1.712	1.712	P_KWMTBOMO09621	P_KWMTBOMO09621	P_KWMTBOMO09621	1
300	2	2	2.454	0.6888	0.6888	P_KWMTBOMO11045	P_KWMTBOMO11045	P_KWMTBOMO11045	1
301	2	2	7.271	2.503	2.503	P_KWMTBOMO10661	P_KWMTBOMO10661	P_KWMTBOMO10661	1
302	2	2	5.486	1.746	1.746	P_KWMTBOMO01072	P_KWMTBOMO01072	P_KWMTBOMO01072	1

303	2	2	6.1050002	1.099	1.099	P_KWMTBOMO00303	P_KWMTBOMO00303	P_KWMTBOMO00303	1
304	2	2	9.2009999	3.8740002	3.8740002	P_KWMTBOMO16261	P_KWMTBOMO16261	P_KWMTBOMO16261	1
305	2	2	2.5389999	0.8669	0.8669	P_KWMTBOMO15715	P_KWMTBOMO15715	P_KWMTBOMO15715	1
306	2	2	4.0550001	1.385	1.385	P_KWMTBOMO14962	P_KWMTBOMO14962	P_KWMTBOMO14962	1
307	2	2	7.2899997	2.853	2.853	P_KWMTBOMO01438	P_KWMTBOMO01438	P_KWMTBOMO01438	1
308	2	2	7.9099998	3.5780001	3.5780001	P_KWMTBOMO00366	P_KWMTBOMO00366	P_KWMTBOMO00366	1
309	2	2	8.7959997	4.6300001	4.6300001	P_KWMTBOMO16600	P_KWMTBOMO16600	P_KWMTBOMO16600	1
310	2	2	2.22	1.544	1.544	P_KWMTBOMO11517	P_KWMTBOMO11517	P_KWMTBOMO11517	1
311	2	2	3.672	2.376	2.376	P_KWMTBOMO11110	P_KWMTBOMO11110	P_KWMTBOMO11110	1
312	2	2	3.506	2.0029999	2.0029999	P_KWMTBOMO10212	P_KWMTBOMO10212	P_KWMTBOMO10212	1
313	2	2	4.6130002	2.1709999	2.1709999	P_KWMTBOMO09811	P_KWMTBOMO09811	P_KWMTBOMO09811	1
314	2	2	6.095	2.709	2.709	P_KWMTBOMO08056	P_KWMTBOMO08056	P_KWMTBOMO08056	1
315	2	2	3.1860001	2.696	2.696	P_KWMTBOMO07554	P_KWMTBOMO07554	P_KWMTBOMO07554	1
316	2	2	1.743	1.072	1.072	P_KWMTBOMO07214	P_KWMTBOMO07214	P_KWMTBOMO07214	1
317	2	2	9.0910003	5.6279998	5.6279998	P_KWMTBOMO06762	P_KWMTBOMO06762	P_KWMTBOMO06762	1
318	2	2	12.729999	6.8180002	6.8180002	P_KWMTBOMO06639	P_KWMTBOMO06639	P_KWMTBOMO06639	1
319	2	2	6.0699999	2.8750001	2.8750001	P_KWMTBOMO05299	P_KWMTBOMO05299	P_KWMTBOMO05299	1
320	2	2	5.415	2.8759999	2.8759999	P_KWMTBOMO05205	P_KWMTBOMO05205	P_KWMTBOMO05205	1
321	2	2	16.670001	5.2850001	5.2850001	P_KWMTBOMO04424	P_KWMTBOMO04424	P_KWMTBOMO04424	1
322	2	2	4.0380001	2.115	2.115	P_KWMTBOMO04057	P_KWMTBOMO04057	P_KWMTBOMO04057	1
324	2	2	8.6669996	5.9999999	5.9999999	P_KWMTBOMO02775	P_KWMTBOMO02775	P_KWMTBOMO02775	1
325	2	2	8.3530001	4.8719998	4.8719998	P_KWMTBOMO02223	P_KWMTBOMO02223	P_KWMTBOMO02223	1
326	2	2	4.7120001	2.88	2.88	P_KWMTBOMO02013	P_KWMTBOMO02013	P_KWMTBOMO02013	1
327	2	2	4.5159999	2.366	2.366	P_KWMTBOMO00279	P_KWMTBOMO00279	P_KWMTBOMO00279	1
328	2	2	7.6920003	7.6920003	7.6920003	P_KWMTBOMO13859	P_KWMTBOMO13859	P_KWMTBOMO13859	1
329	2	2	4.3650001	4.3650001	4.3650001	P_KWMTBOMO12448	P_KWMTBOMO12448	P_KWMTBOMO12448	1
330	2	2	4.5139998	4.5139998	4.5139998	P_KWMTBOMO11202	P_KWMTBOMO11202	P_KWMTBOMO11202	1
331	2	2	1.9640001	1.9640001	1.9640001	P_KWMTBOMO08707	P_KWMTBOMO08707	P_KWMTBOMO08707	1
332	2	2	5.2919999	5.2919999	5.2919999	P_KWMTBOMO08607	P_KWMTBOMO08607	P_KWMTBOMO08607	1
333	2	2	5.263	5.263	5.263	P_KWMTBOMO08323	P_KWMTBOMO08323	P_KWMTBOMO08323	1
334	2	2	17.389999	17.389999	17.389999	P_KWMTBOMO08193	P_KWMTBOMO08193	P_KWMTBOMO08193	1
335	2	2	9.6259996	9.6259996	9.6259996	P_KWMTBOMO07857	P_KWMTBOMO07857	P_KWMTBOMO07857	1
336	2	2	13.850001	13.850001	13.850001	P_KWMTBOMO06654	P_KWMTBOMO06654	P_KWMTBOMO06654	1
337	2	2	4.3960001	4.3960001	4.3960001	P_KWMTBOMO04789	P_KWMTBOMO04789	P_KWMTBOMO04789	1
338	2	2	6.0109999	6.0109999	6.0109999	P_KWMTBOMO04584	P_KWMTBOMO04584	P_KWMTBOMO04584	1
340	2	2	5.914	5.914	5.914	P_KWMTBOMO02836	P_KWMTBOMO02836	P_KWMTBOMO02836	1
341	2	2	13.89	13.89	13.89	P_KWMTBOMO02542	P_KWMTBOMO02542	P_KWMTBOMO02542	1
342	2	2	7.6609999	7.6609999	7.6609999	P_KWMTBOMO02460	P_KWMTBOMO02460	P_KWMTBOMO02460	1
343	2	2	2.08	2.08	2.08	P_KWMTBOMO02354	P_KWMTBOMO02354	P_KWMTBOMO02354	1
344	2	2	6.024	6.024	6.024	P_KWMTBOMO02185	P_KWMTBOMO02185	P_KWMTBOMO02185	1
346	2	2	3.0169999	3.0169999	3.0169999	P_KWMTBOMO01708	P_KWMTBOMO01708	P_KWMTBOMO01708	1
347	2	2	8.901	8.901	8.901	P_KWMTBOMO01658	P_KWMTBOMO01658	P_KWMTBOMO01658	1
348	2	2	4.18	4.18	4.18	P_KWMTBOMO01312	P_KWMTBOMO01312	P_KWMTBOMO01312	1
349	2	2	6.5839998	6.5839998	6.5839998	P_KWMTBOMO00973	P_KWMTBOMO00973	P_KWMTBOMO00973	1
350	1.96	2	2.767	2.767	2.767	P_KWMTBOMO05699	P_KWMTBOMO05699	P_KWMTBOMO05699	1
351	1.96	2	8.5709997	8.5709997	8.5709997	P_KWMTBOMO03003	P_KWMTBOMO03003	P_KWMTBOMO03003	1
352	1.96	2	6.3160002	6.3160002	6.3160002	P_KWMTBOMO01420	P_KWMTBOMO01420	P_KWMTBOMO01420	1
353	1.92	1.92	5.0190002	5.0190002	5.0190002	P_KWMTBOMO04992	P_KWMTBOMO04992	P_KWMTBOMO04992	1

354	1.83	1.83	9.5169999	2.6350001	1.6109999	P_KWMTBOMO01452	P_KWMTBOMO01452	P_KWMTBOMO01452	1
355	1.82	4.13	17.06	8.4109999	6.3079998	P_KWMTBOMO14984	P_KWMTBOMO14984	P_KWMTBOMO14984	2
357	1.8	1.8	4.1269999	3.81	3.81	P_KWMTBOMO05113	P_KWMTBOMO05113	P_KWMTBOMO05113	1
358	1.77	1.77	7.158	3.9999999	3.9999999	P_KWMTBOMO01315	P_KWMTBOMO01315	P_KWMTBOMO01315	1
359	1.74	1.74	13.66	13.66	7.6499999	P_KWMTBOMO01431	P_KWMTBOMO01431	P_KWMTBOMO01431	1
360	1.7	1.7	6.9279999	3.6139999	3.6139999	P_KWMTBOMO07821	P_KWMTBOMO07821	P_KWMTBOMO07821	1
361	1.68	1.68	4.7899999	2.7939999	2.7939999	P_KWMTBOMO15121	P_KWMTBOMO15121	P_KWMTBOMO15121	1
362	1.66	3.92	14.95	14.95	10.28	P_KWMTBOMO13774	P_KWMTBOMO13774	P_KWMTBOMO13774	2
363	1.66	1.66	5.689	1.138	1.138	P_KWMTBOMO05612	P_KWMTBOMO05612	P_KWMTBOMO05612	1
364	1.66	1.66	11.72	11.72	11.72	P_KWMTBOMO00786	P_KWMTBOMO00786	P_KWMTBOMO00786	1
365	1.62	1.62	7.863	2.823	2.823	P_KWMTBOMO13041	P_KWMTBOMO13041	P_KWMTBOMO13041	1
366	1.6	1.6	13.699999	13.699999	13.699999	P_KWMTBOMO10211	P_KWMTBOMO10211	P_KWMTBOMO10211	1
367	1.6	1.6	1.16	1.16	1.16	P_KWMTBOMO08343	P_KWMTBOMO08343	P_KWMTBOMO08343	1
368	1.57	1.57	7.1429998	7.1429998	7.1429998	P_KWMTBOMO11772	P_KWMTBOMO11772	P_KWMTBOMO11772	1
369	1.53	1.53	17.219999	17.219999	7.7780001	P_KWMTBOMO06228	P_KWMTBOMO06228	P_KWMTBOMO06228	1
370	1.51	1.51	1.98	1.98	1.98	P_KWMTBOMO00982	P_KWMTBOMO00982	P_KWMTBOMO00982	1
371	1.49	1.49	2.674	0.8913	0.4902	P_KWMTBOMO05810	P_KWMTBOMO05810	P_KWMTBOMO05810	1
372	1.49	1.49	7.3260002	3.297	3.297	P_KWMTBOMO09641	P_KWMTBOMO09641	P_KWMTBOMO09641	1
373	1.48	3.7	16.27	10.53	10.53	P_KWMTBOMO03027	P_KWMTBOMO03027	P_KWMTBOMO03027	2
374	1.48	1.49	6.6409998	4.2970002	2.148	P_KWMTBOMO08040	P_KWMTBOMO08040	P_KWMTBOMO08040	2
375	1.48	1.48	8.6209998	8.6209998	8.6209998	P_KWMTBOMO11771	P_KWMTBOMO11771	P_KWMTBOMO11771	1
376	1.47	1.48	11.16	5.463	3.088	P_KWMTBOMO04330	P_KWMTBOMO04330	P_KWMTBOMO04330	1
377	1.44	1.53	6.9250003	3.4619998	2.2399999	P_KWMTBOMO03002	P_KWMTBOMO03002	P_KWMTBOMO03002	1
378	1.43	1.43	8.6120002	5.263	5.263	P_KWMTBOMO13723	P_KWMTBOMO13723	P_KWMTBOMO13723	1
379	1.42	1.42	7.3420003	4.5570001	2.5319999	P_KWMTBOMO08310	P_KWMTBOMO08310	P_KWMTBOMO08310	2
380	1.41	1.41	6.318	1.9859999	1.9859999	P_KWMTBOMO09838	P_KWMTBOMO09838	P_KWMTBOMO09838	1
382	1.36	1.36	12.34	8.4420003	8.4420003	P_KWMTBOMO16524	P_KWMTBOMO16524	P_KWMTBOMO16524	1
384	1.34	1.34	17.16	17.16	8.3329998	P_KWMTBOMO09631	P_KWMTBOMO09631	P_KWMTBOMO09631	1
385	1.34	1.34	3.743	3.743	3.743	P_KWMTBOMO15712	P_KWMTBOMO15712	P_KWMTBOMO15712	1
386	1.3	1.3	3.036	3.036	3.036	P_KWMTBOMO05993	P_KWMTBOMO05993	P_KWMTBOMO05993	1
387	1.3	1.3	2.994	2.994	2.994	P_KWMTBOMO04966	P_KWMTBOMO04966	P_KWMTBOMO04966	1

Summary of the identified PIPs of P25_M4

N	Unused	Total	%Cov	%Cov(50)	%Cov(95)	Accession	Citable Accession	Name	Peptides(95%)
3	27.84	27.84	12.67	8.946	6.7309998	P_KWMTBOMO08648	P_KWMTBOMO08648	P_KWMTBOMO08648	13
8	19.9	19.9	19.82	16.44	13.29	P_KWMTBOMO00462	P_KWMTBOMO00462	P_KWMTBOMO00462	9
10	16.33	16.33	9.1880001	6.6500001	4.518	P_KWMTBOMO10056	P_KWMTBOMO10056	P_KWMTBOMO10056	9
12	15.22	15.22	39.6	36.24	28.119999	P_KWMTBOMO07374	P_KWMTBOMO07374	P_KWMTBOMO07374	10
13	15.13	15.13	17.309999	15.12	12.27	P_KWMTBOMO15159	P_KWMTBOMO15159	P_KWMTBOMO15159	7
16	14.21	14.21	16	13.98	10.19	P_KWMTBOMO11731	P_KWMTBOMO11731	P_KWMTBOMO11731	8
17	14.03	14.03	21.4	13.88	13.88	P_KWMTBOMO04502	P_KWMTBOMO04502	P_KWMTBOMO04502	7
19	13.23	13.23	10.2	4.9660001	3.07	P_KWMTBOMO06632	P_KWMTBOMO06632	P_KWMTBOMO06632	6
20	13.13	20.06	31.299999	27.630001	17.1	P_KWMTBOMO08642	P_KWMTBOMO08642	P_KWMTBOMO08642	12
21	12.35	12.35	21.520001	21.359999	18.2	P_KWMTBOMO08044	P_KWMTBOMO08044	P_KWMTBOMO08044	6
24	11.94	11.94	11.07	7.2389998	7.2389998	P_KWMTBOMO01927	P_KWMTBOMO01927	P_KWMTBOMO01927	8
25	11.4	11.4	13.11	10.93	8.5610002	P_KWMTBOMO00927	P_KWMTBOMO00927	P_KWMTBOMO00927	5
27	11.02	11.02	12.92	6.0740001	6.0740001	P_KWMTBOMO04505	P_KWMTBOMO04505	P_KWMTBOMO04505	6
29	10.89	10.89	28.850001	28.850001	25.690001	P_KWMTBOMO12497	P_KWMTBOMO12497	P_KWMTBOMO12497	7
31	9.97	9.97	17.07	15.4	13.91	P_KWMTBOMO01075	P_KWMTBOMO01075	P_KWMTBOMO01075	5

32	9.94	9.94	29.83	24.429999	18.47	P_KWMTBOMO05675	P_KWMTBOMO05675	P_KWMTBOMO05675	8
33	9.88	9.88	20.34	17.89	10.73	P_KWMTBOMO09808	P_KWMTBOMO09808	P_KWMTBOMO09808	5
34	9.76	9.76	9.5559999	8.687	6.6600002	P_KWMTBOMO11517	P_KWMTBOMO11517	P_KWMTBOMO11517	5
35	9.62	9.62	11.18	11.18	11.18	P_KWMTBOMO05564	P_KWMTBOMO05564	P_KWMTBOMO05564	5
37	9.39	9.39	24.71	18.629999	16.35	P_KWMTBOMO13455	P_KWMTBOMO13455	P_KWMTBOMO13455	5
38	9.26	9.26	20.819999	15.24	15.24	P_KWMTBOMO11985	P_KWMTBOMO11985	P_KWMTBOMO11985	5
41	8.76	8.76	11.44	7.0589997	5.2409999	P_KWMTBOMO14420	P_KWMTBOMO14420	P_KWMTBOMO14420	4
43	8.39	8.39	8.5510001	8.5510001	8.5510001	P_KWMTBOMO14549	P_KWMTBOMO14549	P_KWMTBOMO14549	5
45	8.06	8.06	18.77	15.82	15.82	P_KWMTBOMO06231	P_KWMTBOMO06231	P_KWMTBOMO06231	5
46	8.03	8.03	7.3969997	3.263	3.263	P_KWMTBOMO14858	P_KWMTBOMO14858	P_KWMTBOMO14858	4
47	7.81	7.81	17.01	14.37	14.37	P_KWMTBOMO13997	P_KWMTBOMO13997	P_KWMTBOMO13997	5
49	7.49	7.49	11.09	11.09	7.5560004	P_KWMTBOMO03748	P_KWMTBOMO03748	P_KWMTBOMO03748	4
52	6.83	6.83	10.7	9.2249997	9.2249997	P_KWMTBOMO14887	P_KWMTBOMO14887	P_KWMTBOMO14887	5
55	6.73	6.73	23.720001	19.87	19.87	P_KWMTBOMO16040	P_KWMTBOMO16040	P_KWMTBOMO16040	7
56	6.41	6.41	9.3800001	9.3800001	5.7720002	P_KWMTBOMO13567	P_KWMTBOMO13567	P_KWMTBOMO13567	3
57	6.39	6.39	6.03	4.8239999	4.0199999	P_KWMTBOMO10780	P_KWMTBOMO10780	P_KWMTBOMO10780	3
58	6.32	6.32	10.53	9.076	6.3210003	P_KWMTBOMO11096	P_KWMTBOMO11096	P_KWMTBOMO11096	4
59	6.19	6.19	11.21	6.8209998	4.8719998	P_KWMTBOMO00638	P_KWMTBOMO00638	P_KWMTBOMO00638	3
60	6.09	6.09	6.9310002	4.4769999	1.291	P_KWMTBOMO11045	P_KWMTBOMO11045	P_KWMTBOMO11045	2
61	5.98	5.98	12.890001	12.890001	8.9469999	P_KWMTBOMO01720	P_KWMTBOMO01720	P_KWMTBOMO01720	4
63	5.95	5.95	13.31	9.2739999	9.2739999	P_KWMTBOMO12037	P_KWMTBOMO12037	P_KWMTBOMO12037	3
64	5.9	5.9	27.320001	20.77	16.940001	P_KWMTBOMO06480	P_KWMTBOMO06480	P_KWMTBOMO06480	4
65	5.79	5.79	21.789999	18.59	12.18	P_KWMTBOMO16357	P_KWMTBOMO16357	P_KWMTBOMO16357	3
66	5.74	5.74	11.72	8.6960003	8.6960003	P_KWMTBOMO01606	P_KWMTBOMO01606	P_KWMTBOMO01606	4
67	5.66	5.66	2.998	1.7899999	1.7899999	P_KWMTBOMO05728	P_KWMTBOMO05728	P_KWMTBOMO05728	3
69	5.56	5.56	9.4279997	7.5730003	7.5730003	P_KWMTBOMO13370	P_KWMTBOMO13370	P_KWMTBOMO13370	3
70	5.55	5.55	40.849999	30.989999	15.49	P_KWMTBOMO07373	P_KWMTBOMO07373	P_KWMTBOMO07373	5
71	5.45	5.45	8.6999997	6.8999998	4.3000001	P_KWMTBOMO13293	P_KWMTBOMO13293	P_KWMTBOMO13293	3
72	5.29	5.29	9.5619999	4.648	4.648	P_KWMTBOMO01817	P_KWMTBOMO01817	P_KWMTBOMO01817	3
73	5.28	5.28	6.1020002	3.136	1.6100001	P_KWMTBOMO09542	P_KWMTBOMO09542	P_KWMTBOMO09542	2
74	5.14	5.14	9.9100001	5.5860002	5.5860002	P_KWMTBOMO03626	P_KWMTBOMO03626	P_KWMTBOMO03626	3
75	5.11	5.11	3.0200001	3.0200001	3.0200001	P_KWMTBOMO00427	P_KWMTBOMO00427	P_KWMTBOMO00427	3
76	4.88	4.88	17.69	14.62	12.31	P_KWMTBOMO13405	P_KWMTBOMO13405	P_KWMTBOMO13405	2
77	4.82	5.75	7.9010002	5.0620001	2.716	P_KWMTBOMO12844	P_KWMTBOMO12844	P_KWMTBOMO12844	2
78	4.82	4.82	6.0770001	6.0770001	4.972	P_KWMTBOMO15359	P_KWMTBOMO15359	P_KWMTBOMO15359	3
79	4.75	4.75	9.7829998	5.28	4.0369999	P_KWMTBOMO00687	P_KWMTBOMO00687	P_KWMTBOMO00687	2
82	4.59	4.59	24.2	17.35	9.589	P_KWMTBOMO05979	P_KWMTBOMO05979	P_KWMTBOMO05979	3
84	4.56	4.56	13.65	12	10.12	P_KWMTBOMO09415	P_KWMTBOMO09415	P_KWMTBOMO09415	7
85	4.5	4.5	27.469999	15.38	7.3260002	P_KWMTBOMO09442	P_KWMTBOMO09442	P_KWMTBOMO09442	2
86	4.45	4.45	8.6300001	7.5049996	4.1280001	P_KWMTBOMO06770	P_KWMTBOMO06770	P_KWMTBOMO06770	2
87	4.45	4.45	10.95	9.2859998	6.9049999	P_KWMTBOMO02081	P_KWMTBOMO02081	P_KWMTBOMO02081	2
88	4.43	4.43	11.31	7.7890001	5.779	P_KWMTBOMO12166	P_KWMTBOMO12166	P_KWMTBOMO12166	2
89	4.32	4.32	5.4570001	3.9560001	3.9560001	P_KWMTBOMO03177	P_KWMTBOMO03177	P_KWMTBOMO03177	3
90	4.3	4.3	9.3850002	4.0989999	2.6969999	P_KWMTBOMO04610	P_KWMTBOMO04610	P_KWMTBOMO04610	2
91	4.29	4.29	10.44	5.8820002	2.9410001	P_KWMTBOMO08652	P_KWMTBOMO08652	P_KWMTBOMO08652	3
92	4.24	4.24	45.71	25.99	8.8919997	P_KWMTBOMO12607	P_KWMTBOMO12607	P_KWMTBOMO12607	2
93	4.23	4.23	7.1630001	4.394	2.674	P_KWMTBOMO09055	P_KWMTBOMO09055	P_KWMTBOMO09055	2
94	4.23	4.23	11.68	8.286	8.286	P_KWMTBOMO00366	P_KWMTBOMO00366	P_KWMTBOMO00366	3

95	4.12	4.12	4.1280001	1.223	1.223	P_KWMTBOMO15617	P_KWMTBOMO15617	P_KWMTBOMO15617	2
96	4.1	4.1	27.970001	14.94	8.4289998	P_KWMTBOMO07688	P_KWMTBOMO07688	P_KWMTBOMO07688	2
97	4.08	4.08	29.339999	19.310001	19.310001	P_KWMTBOMO00339	P_KWMTBOMO00339	P_KWMTBOMO00339	4
98	4.07	4.07	7.66	6.6849999	6.6849999	P_KWMTBOMO10358	P_KWMTBOMO10358	P_KWMTBOMO10358	3
99	4.07	4.07	5.9870001	4.5310002	4.5310002	P_KWMTBOMO03630	P_KWMTBOMO03630	P_KWMTBOMO03630	2
100	4.05	4.05	23	15.019999	15.019999	P_KWMTBOMO05566	P_KWMTBOMO05566	P_KWMTBOMO05566	3
101	4.04	4.04	17.53	8.4420003	8.4420003	P_KWMTBOMO16524	P_KWMTBOMO16524	P_KWMTBOMO16524	2
102	4.04	4.04	12.74	7.9620004	7.9620004	P_KWMTBOMO15758	P_KWMTBOMO15758	P_KWMTBOMO15758	3
104	4	4	27.070001	15.47	15.47	P_KWMTBOMO16637	P_KWMTBOMO16637	P_KWMTBOMO16637	2
105	4	4	20	8.7499999	8.7499999	P_KWMTBOMO07772	P_KWMTBOMO07772	P_KWMTBOMO07772	2
106	4	4	8.2379997	5.4919999	5.4919999	P_KWMTBOMO15123	P_KWMTBOMO15123	P_KWMTBOMO15123	2
108	4	4	14.489999	10.87	10.87	P_KWMTBOMO06621	P_KWMTBOMO06621	P_KWMTBOMO06621	2
109	4	4	7.1429998	4.7619998	4.7619998	P_KWMTBOMO02748	P_KWMTBOMO02748	P_KWMTBOMO02748	3
110	4	4	9.4769999	9.4769999	9.4769999	P_KWMTBOMO10248	P_KWMTBOMO10248	P_KWMTBOMO10248	2
111	4	4	7.6920003	7.6920003	7.6920003	P_KWMTBOMO03860	P_KWMTBOMO03860	P_KWMTBOMO03860	2
112	3.92	3.92	11.54	4.487	4.487	P_KWMTBOMO02470	P_KWMTBOMO02470	P_KWMTBOMO02470	2
113	3.92	3.92	3.1959999	3.1959999	3.1959999	P_KWMTBOMO09621	P_KWMTBOMO09621	P_KWMTBOMO09621	2
114	3.89	4.44	8.5879996	6.2980004	5.153	P_KWMTBOMO12560	P_KWMTBOMO12560	P_KWMTBOMO12560	2
115	3.8	3.8	21.68	13.29	13.29	P_KWMTBOMO08666	P_KWMTBOMO08666	P_KWMTBOMO08666	3
117	3.74	3.74	10.63	6.5399997	6.5399997	P_KWMTBOMO04370	P_KWMTBOMO04370	P_KWMTBOMO04370	2
118	3.72	3.72	10.56	6.2109999	2.981	P_KWMTBOMO11666	P_KWMTBOMO11666	P_KWMTBOMO11666	2
119	3.67	3.67	3.5519999	2.6839999	1.736	P_KWMTBOMO03067	P_KWMTBOMO03067	P_KWMTBOMO03067	2
120	3.64	3.64	4.2640001	4.2640001	4.2640001	P_KWMTBOMO14752	P_KWMTBOMO14752	P_KWMTBOMO14752	2
121	3.59	3.59	6.295	1.6140001	1.6140001	P_KWMTBOMO05687	P_KWMTBOMO05687	P_KWMTBOMO05687	2
122	3.56	3.56	39.219999	27.450001	27.450001	P_KWMTBOMO13569	P_KWMTBOMO13569	P_KWMTBOMO13569	2
123	3.55	3.56	3.5399999	3.5399999	3.413	P_KWMTBOMO05612	P_KWMTBOMO05612	P_KWMTBOMO05612	2
124	3.54	3.54	6.5130003	6.5130003	4.4119999	P_KWMTBOMO12895	P_KWMTBOMO12895	P_KWMTBOMO12895	3
126	3.51	3.51	12.32	12.32	12.32	P_KWMTBOMO00794	P_KWMTBOMO00794	P_KWMTBOMO00794	3
127	3.49	3.49	39.500001	19.329999	19.329999	P_KWMTBOMO15114	P_KWMTBOMO15114	P_KWMTBOMO15114	2
128	3.47	3.47	4.4909999	2.3949999	2.3949999	P_KWMTBOMO05541	P_KWMTBOMO05541	P_KWMTBOMO05541	2
129	3.47	3.47	9.7259998	9.7259998	6.6869996	P_KWMTBOMO08776	P_KWMTBOMO08776	P_KWMTBOMO08776	2
130	3.39	3.39	26.800001	12.890001	9.2780001	P_KWMTBOMO11255	P_KWMTBOMO11255	P_KWMTBOMO11255	2
131	3.36	3.36	13.950001	6.1469998	6.1469998	P_KWMTBOMO11083	P_KWMTBOMO11083	P_KWMTBOMO11083	2
132	3.36	3.36	2.734	2.734	2.734	P_KWMTBOMO10493	P_KWMTBOMO10493	P_KWMTBOMO10493	2
133	3.27	3.27	38.850001	20.14	14.390001	P_KWMTBOMO08399	P_KWMTBOMO08399	P_KWMTBOMO08399	2
134	3.26	3.27	12.21	12.21	12.21	P_KWMTBOMO03550	P_KWMTBOMO03550	P_KWMTBOMO03550	2
135	3.26	3.26	8.1950001	3.5270002	2.386	P_KWMTBOMO05741	P_KWMTBOMO05741	P_KWMTBOMO05741	2
136	3.22	3.22	9.8119996	4.5929998	4.5929998	P_KWMTBOMO08956	P_KWMTBOMO08956	P_KWMTBOMO08956	3
137	3.21	3.21	21.65	21.65	21.65	P_KWMTBOMO01918	P_KWMTBOMO01918	P_KWMTBOMO01918	2
138	3.18	3.18	8.168	4.4550002	4.4550002	P_KWMTBOMO07331	P_KWMTBOMO07331	P_KWMTBOMO07331	2
139	3.13	3.16	8.2560003	3.3720002	2.2089999	P_KWMTBOMO15583	P_KWMTBOMO15583	P_KWMTBOMO15583	2
140	3.12	3.12	7.9510003	7.9510003	7.9510003	P_KWMTBOMO00497	P_KWMTBOMO00497	P_KWMTBOMO00497	2
141	3.06	3.06	5.006	3.576	3.576	P_KWMTBOMO10661	P_KWMTBOMO10661	P_KWMTBOMO10661	2
142	3.04	3.04	6.921	3.2219999	3.2219999	P_KWMTBOMO08213	P_KWMTBOMO08213	P_KWMTBOMO08213	2
143	3.01	3.01	10.68	7.7150002	3.858	P_KWMTBOMO10376	P_KWMTBOMO10376	P_KWMTBOMO10376	1
144	2.98	2.98	4.775	3.2299999	3.2299999	P_KWMTBOMO13226	P_KWMTBOMO13226	P_KWMTBOMO13226	2
146	2.95	2.95	9.6029997	9.6029997	9.6029997	P_KWMTBOMO12389	P_KWMTBOMO12389	P_KWMTBOMO12389	2
147	2.93	2.93	18.99	12.03	6.013	P_KWMTBOMO09300	P_KWMTBOMO09300	P_KWMTBOMO09300	1

148	2.9	2.9	4.2640001	2.452	2.452	P_KWMTBOMO12667	P_KWMTBOMO12667	P_KWMTBOMO12667	2
149	2.9	2.9	19.499999	12.58	12.58	P_KWMTBOMO08100	P_KWMTBOMO08100	P_KWMTBOMO08100	2
150	2.77	2.77	16.08	4.7279999	1.891	P_KWMTBOMO00375	P_KWMTBOMO00375	P_KWMTBOMO00375	1
151	2.76	2.76	25.670001	7.9999998	7.9999998	P_KWMTBOMO14732	P_KWMTBOMO14732	P_KWMTBOMO14732	2
153	2.7	2.7	7.8780003	5.466	2.251	P_KWMTBOMO02432	P_KWMTBOMO02432	P_KWMTBOMO02432	1
154	2.68	2.68	14.35	14.35	5.0930001	P_KWMTBOMO14417	P_KWMTBOMO14417	P_KWMTBOMO14417	1
155	2.68	2.68	11.04	11.04	5.3509999	P_KWMTBOMO11061	P_KWMTBOMO11061	P_KWMTBOMO11061	2
156	2.66	2.66	15.82	15.82	8.2280003	P_KWMTBOMO14479	P_KWMTBOMO14479	P_KWMTBOMO14479	1
157	2.61	2.68	24.65	6.2210001	2.5350001	P_KWMTBOMO06229	P_KWMTBOMO06229	P_KWMTBOMO06229	1
158	2.58	2.58	31.34	20.900001	5.9700001	P_KWMTBOMO04742	P_KWMTBOMO04742	P_KWMTBOMO04742	1
160	2.55	2.55	9.3659997	6.0419999	6.0419999	P_KWMTBOMO07506	P_KWMTBOMO07506	P_KWMTBOMO07506	4
161	2.54	2.54	29.800001	24.5	11.92	P_KWMTBOMO08440	P_KWMTBOMO08440	P_KWMTBOMO08440	2
162	2.48	2.48	5.6340002	5.6340002	3.052	P_KWMTBOMO00437	P_KWMTBOMO00437	P_KWMTBOMO00437	1
163	2.46	2.46	27.149999	14.569999	14.569999	P_KWMTBOMO13960	P_KWMTBOMO13960	P_KWMTBOMO13960	2
164	2.4	2.4	3.6430001	3.6430001	2.186	P_KWMTBOMO10208	P_KWMTBOMO10208	P_KWMTBOMO10208	1
165	2.37	2.37	13.73	9.804	5.8820002	P_KWMTBOMO01273	P_KWMTBOMO01273	P_KWMTBOMO01273	1
166	2.34	2.34	12.800001	7.0119999	4.5729998	P_KWMTBOMO06082	P_KWMTBOMO06082	P_KWMTBOMO06082	1
167	2.34	2.34	1.6100001	1.6100001	0.8669	P_KWMTBOMO15715	P_KWMTBOMO15715	P_KWMTBOMO15715	1
168	2.32	2.32	14.839999	8.6000003	3.204	P_KWMTBOMO00834	P_KWMTBOMO00834	P_KWMTBOMO00834	2
169	2.23	2.23	12.09	4.1859999	2.791	P_KWMTBOMO02061	P_KWMTBOMO02061	P_KWMTBOMO02061	1
170	2.18	2.18	3.743	3.743	2.273	P_KWMTBOMO16130	P_KWMTBOMO16130	P_KWMTBOMO16130	1
171	2.15	2.15	20.93	13.18	6.9770001	P_KWMTBOMO05723	P_KWMTBOMO05723	P_KWMTBOMO05723	2
172	2.13	2.13	14.309999	4.3310001	2.448	P_KWMTBOMO02994	P_KWMTBOMO02994	P_KWMTBOMO02994	1
174	2.12	2.12	7.4929997	3.678	2.18	P_KWMTBOMO03430	P_KWMTBOMO03430	P_KWMTBOMO03430	1
176	2.1	2.1	16.18	4.4119999	4.4119999	P_KWMTBOMO03314	P_KWMTBOMO03314	P_KWMTBOMO03314	1
177	2.1	2.1	14.41	6.7570001	6.7570001	P_KWMTBOMO01424	P_KWMTBOMO01424	P_KWMTBOMO01424	1
178	2.08	2.08	4.228	4.228	1.9029999	P_KWMTBOMO12205	P_KWMTBOMO12205	P_KWMTBOMO12205	1
179	2.08	2.08	14.63	9.1459997	9.1459997	P_KWMTBOMO11678	P_KWMTBOMO11678	P_KWMTBOMO11678	1
180	2.06	2.06	13.37	5.446	5.446	P_KWMTBOMO15361	P_KWMTBOMO15361	P_KWMTBOMO15361	1
181	2.05	2.05	5.9209999	3.9469998	1.809	P_KWMTBOMO06787	P_KWMTBOMO06787	P_KWMTBOMO06787	1
182	2.04	2.04	1.904	0.6544	0.6544	P_KWMTBOMO09747	P_KWMTBOMO09747	P_KWMTBOMO09747	2
183	2.03	2.03	5.3160001	2.5319999	2.5319999	P_KWMTBOMO08310	P_KWMTBOMO08310	P_KWMTBOMO08310	1
184	2.02	2.02	2	0.3209	0.3209	P_KWMTBOMO12602	P_KWMTBOMO12602	P_KWMTBOMO12602	1
185	2.02	2.02	8.0069996	4.2830002	2.421	P_KWMTBOMO07323	P_KWMTBOMO07323	P_KWMTBOMO07323	1
186	2.02	2.02	10.57	6.4149998	6.4149998	P_KWMTBOMO11780	P_KWMTBOMO11780	P_KWMTBOMO11780	1
188	2.01	2.01	25.960001	12.5	12.5	P_KWMTBOMO06974	P_KWMTBOMO06974	P_KWMTBOMO06974	1
190	2	4	7.7100001	4.9070001	4.9070001	P_KWMTBOMO14984	P_KWMTBOMO14984	P_KWMTBOMO14984	2
191	2	2	6.2029999	2.4809999	2.4809999	P_KWMTBOMO11098	P_KWMTBOMO11098	P_KWMTBOMO11098	1
192	2	2	5.2730002	1.151	1.151	P_KWMTBOMO04806	P_KWMTBOMO04806	P_KWMTBOMO04806	1
193	2	2	6.5420002	2.4529999	2.4529999	P_KWMTBOMO03016	P_KWMTBOMO03016	P_KWMTBOMO03016	1
194	2	2	10.14	3.5089999	3.5089999	P_KWMTBOMO00881	P_KWMTBOMO00881	P_KWMTBOMO00881	1
195	2	2	8.467	2.517	2.517	P_KWMTBOMO14530	P_KWMTBOMO14530	P_KWMTBOMO14530	1
196	2	2	5.1720001	2.069	2.069	P_KWMTBOMO11590	P_KWMTBOMO11590	P_KWMTBOMO11590	1
197	2	2	2.5599999	1.28	1.28	P_KWMTBOMO08509	P_KWMTBOMO08509	P_KWMTBOMO08509	1
198	2	2	3.7640002	1.322	1.322	P_KWMTBOMO08404	P_KWMTBOMO08404	P_KWMTBOMO08404	1
199	2	2	4.1159999	1.891	1.891	P_KWMTBOMO07274	P_KWMTBOMO07274	P_KWMTBOMO07274	1
200	2	2	4.7389999	1.7379999	1.7379999	P_KWMTBOMO06803	P_KWMTBOMO06803	P_KWMTBOMO06803	1
201	2	2	2.874	1.149	1.149	P_KWMTBOMO06766	P_KWMTBOMO06766	P_KWMTBOMO06766	1

202	2	2	2.2949999	0.8032	0.8032	P_KWMTBOMO06334	P_KWMTBOMO06334	P_KWMTBOMO06334	1
203	2	2	2.32	1.198	1.198	P_KWMTBOMO05688	P_KWMTBOMO05688	P_KWMTBOMO05688	1
204	2	2	5.7100002	2.3429999	2.3429999	P_KWMTBOMO01452	P_KWMTBOMO01452	P_KWMTBOMO01452	1
205	2	2	1.767	0.8346	0.8346	P_KWMTBOMO00574	P_KWMTBOMO00574	P_KWMTBOMO00574	1
206	2	2	6.5339997	3.977	3.977	P_KWMTBOMO16530	P_KWMTBOMO16530	P_KWMTBOMO16530	1
207	2	2	8.7180004	5.8970001	5.8970001	P_KWMTBOMO16309	P_KWMTBOMO16309	P_KWMTBOMO16309	1
208	2	2	8.8890001	4.8149999	4.8149999	P_KWMTBOMO15867	P_KWMTBOMO15867	P_KWMTBOMO15867	1
209	2	2	7.8429997	4.902	4.902	P_KWMTBOMO14346	P_KWMTBOMO14346	P_KWMTBOMO14346	1
210	2	2	6.4939998	3.3769999	3.3769999	P_KWMTBOMO14041	P_KWMTBOMO14041	P_KWMTBOMO14041	1
211	2	2	2.84	1.567	1.567	P_KWMTBOMO13235	P_KWMTBOMO13235	P_KWMTBOMO13235	1
212	2	2	8.0760002	4.276	4.276	P_KWMTBOMO08893	P_KWMTBOMO08893	P_KWMTBOMO08893	1
213	2	2	17.389999	17.389999	17.389999	P_KWMTBOMO08193	P_KWMTBOMO08193	P_KWMTBOMO08193	1
214	2	2	8.6960003	6.763	6.763	P_KWMTBOMO08106	P_KWMTBOMO08106	P_KWMTBOMO08106	1
215	2	2	4.7800001	3.2499999	3.2499999	P_KWMTBOMO08041	P_KWMTBOMO08041	P_KWMTBOMO08041	1
216	2	2	3.9110001	2.3469999	2.3469999	P_KWMTBOMO07380	P_KWMTBOMO07380	P_KWMTBOMO07380	1
217	2	2	4.5699999	2.3770001	2.3770001	P_KWMTBOMO07294	P_KWMTBOMO07294	P_KWMTBOMO07294	1
218	2	2	4.5079999	2.671	2.671	P_KWMTBOMO05673	P_KWMTBOMO05673	P_KWMTBOMO05673	1
219	2	2	11.47	3.7409998	3.7409998	P_KWMTBOMO05056	P_KWMTBOMO05056	P_KWMTBOMO05056	1
220	2	2	5.0810002	3.695	3.695	P_KWMTBOMO04605	P_KWMTBOMO04605	P_KWMTBOMO04605	1
221	2	2	6.8800002	3.686	3.686	P_KWMTBOMO02835	P_KWMTBOMO02835	P_KWMTBOMO02835	1
222	2	2	12.1	7.6609999	7.6609999	P_KWMTBOMO02460	P_KWMTBOMO02460	P_KWMTBOMO02460	2
223	2	2	2.121	1.097	1.097	P_KWMTBOMO02211	P_KWMTBOMO02211	P_KWMTBOMO02211	1
224	2	2	6.25	3.2329999	3.2329999	P_KWMTBOMO01708	P_KWMTBOMO01708	P_KWMTBOMO01708	1
225	2	2	4.324	4.324	4.324	P_KWMTBOMO15584	P_KWMTBOMO15584	P_KWMTBOMO15584	1
226	2	2	0.892	0.892	0.892	P_KWMTBOMO15314	P_KWMTBOMO15314	P_KWMTBOMO15314	1
228	2	2	1.818	1.818	1.818	P_KWMTBOMO15060	P_KWMTBOMO15060	P_KWMTBOMO15060	1
229	2	2	2.935	2.935	2.935	P_KWMTBOMO14804	P_KWMTBOMO14804	P_KWMTBOMO14804	1
230	2	2	1.461	1.461	1.461	P_KWMTBOMO14568	P_KWMTBOMO14568	P_KWMTBOMO14568	1
231	2	2	12.16	12.16	12.16	P_KWMTBOMO14440	P_KWMTBOMO14440	P_KWMTBOMO14440	1
232	2	2	6.1900001	6.1900001	6.1900001	P_KWMTBOMO13641	P_KWMTBOMO13641	P_KWMTBOMO13641	1
233	2	2	1.912	1.912	1.912	P_KWMTBOMO12264	P_KWMTBOMO12264	P_KWMTBOMO12264	1
234	2	2	2.7650001	2.7650001	2.7650001	P_KWMTBOMO12165	P_KWMTBOMO12165	P_KWMTBOMO12165	1
235	2	2	6.5420002	6.5420002	6.5420002	P_KWMTBOMO11448	P_KWMTBOMO11448	P_KWMTBOMO11448	1
236	2	2	4.5139998	4.5139998	4.5139998	P_KWMTBOMO11202	P_KWMTBOMO11202	P_KWMTBOMO11202	1
237	2	2	3.218	3.218	3.218	P_KWMTBOMO09054	P_KWMTBOMO09054	P_KWMTBOMO09054	1
238	2	2	6.6229999	6.6229999	6.6229999	P_KWMTBOMO09000	P_KWMTBOMO09000	P_KWMTBOMO09000	1
239	2	2	5.5319998	5.5319998	5.5319998	P_KWMTBOMO08862	P_KWMTBOMO08862	P_KWMTBOMO08862	1
240	2	2	11.11	11.11	11.11	P_KWMTBOMO08820	P_KWMTBOMO08820	P_KWMTBOMO08820	1
241	2	2	5.2919999	5.2919999	5.2919999	P_KWMTBOMO08607	P_KWMTBOMO08607	P_KWMTBOMO08607	1
242	2	2	3.937	3.937	3.937	P_KWMTBOMO08535	P_KWMTBOMO08535	P_KWMTBOMO08535	1
243	2	2	5.263	5.263	5.263	P_KWMTBOMO08323	P_KWMTBOMO08323	P_KWMTBOMO08323	1
244	2	2	2.4940001	2.4940001	2.4940001	P_KWMTBOMO08194	P_KWMTBOMO08194	P_KWMTBOMO08194	1
245	2	2	3.8860001	3.8860001	3.8860001	P_KWMTBOMO07780	P_KWMTBOMO07780	P_KWMTBOMO07780	1
246	2	2	0.9298	0.9298	0.9298	P_KWMTBOMO07589	P_KWMTBOMO07589	P_KWMTBOMO07589	1
247	2	2	2.111	2.111	2.111	P_KWMTBOMO07076	P_KWMTBOMO07076	P_KWMTBOMO07076	1
248	2	2	2.4839999	2.4839999	2.4839999	P_KWMTBOMO06731	P_KWMTBOMO06731	P_KWMTBOMO06731	1
249	2	2	5.0719999	5.0719999	5.0719999	P_KWMTBOMO05975	P_KWMTBOMO05975	P_KWMTBOMO05975	1
250	2	2	2.369	2.369	2.369	P_KWMTBOMO05205	P_KWMTBOMO05205	P_KWMTBOMO05205	1

251	2	2	7.333	7.333	7.333	P_KWMTBOMO04504	P_KWMTBOMO04504	P_KWMTBOMO04504	1
252	2	2	4.527	4.527	4.527	P_KWMTBOMO04168	P_KWMTBOMO04168	P_KWMTBOMO04168	1
253	2	2	6.9770001	6.9770001	6.9770001	P_KWMTBOMO03173	P_KWMTBOMO03173	P_KWMTBOMO03173	1
254	2	2	3.7140001	3.7140001	3.7140001	P_KWMTBOMO03146	P_KWMTBOMO03146	P_KWMTBOMO03146	1
255	2	2	11.11	11.11	11.11	P_KWMTBOMO02431	P_KWMTBOMO02431	P_KWMTBOMO02431	2
256	2	2	6.024	6.024	6.024	P_KWMTBOMO02185	P_KWMTBOMO02185	P_KWMTBOMO02185	1
257	2	2	7.8950003	7.8950003	7.8950003	P_KWMTBOMO02059	P_KWMTBOMO02059	P_KWMTBOMO02059	1
258	2	2	1.746	1.746	1.746	P_KWMTBOMO01072	P_KWMTBOMO01072	P_KWMTBOMO01072	2
259	2	2	11.72	11.72	11.72	P_KWMTBOMO00786	P_KWMTBOMO00786	P_KWMTBOMO00786	1
260	1.92	2	4.4440001	4.4440001	4.4440001	P_KWMTBOMO08201	P_KWMTBOMO08201	P_KWMTBOMO08201	1
261	1.89	1.89	17.110001	9.6259996	9.6259996	P_KWMTBOMO07857	P_KWMTBOMO07857	P_KWMTBOMO07857	1
262	1.87	1.87	11.49	6.7570001	6.7570001	P_KWMTBOMO07930	P_KWMTBOMO07930	P_KWMTBOMO07930	1
263	1.85	1.85	3.7039999	3.7039999	2.407	P_KWMTBOMO12659	P_KWMTBOMO12659	P_KWMTBOMO12659	1
264	1.85	1.85	8.2570001	4.8930001	4.8930001	P_KWMTBOMO06056	P_KWMTBOMO06056	P_KWMTBOMO06056	1
265	1.82	1.82	6.3409999	6.3409999	2.1740001	P_KWMTBOMO14483	P_KWMTBOMO14483	P_KWMTBOMO14483	1
266	1.8	1.8	1.9889999	0.4232	0.4232	P_KWMTBOMO05013	P_KWMTBOMO05013	P_KWMTBOMO05013	1
267	1.77	1.77	3.221	1.273	1.273	P_KWMTBOMO14080	P_KWMTBOMO14080	P_KWMTBOMO14080	1
268	1.74	1.74	4.9380001	3.0859999	3.0859999	P_KWMTBOMO09385	P_KWMTBOMO09385	P_KWMTBOMO09385	1
269	1.72	1.74	6.8180002	6.8180002	6.8180002	P_KWMTBOMO06639	P_KWMTBOMO06639	P_KWMTBOMO06639	1
270	1.69	1.69	25	16.429999	5.714	P_KWMTBOMO00838	P_KWMTBOMO00838	P_KWMTBOMO00838	1
271	1.68	1.68	3.647	3.647	1.403	P_KWMTBOMO11336	P_KWMTBOMO11336	P_KWMTBOMO11336	1
272	1.66	1.66	7.4189998	3.8710002	3.8710002	P_KWMTBOMO13369	P_KWMTBOMO13369	P_KWMTBOMO13369	1
273	1.63	1.63	5.2060001	3.7530001	2.0579999	P_KWMTBOMO11160	P_KWMTBOMO11160	P_KWMTBOMO11160	1
274	1.62	1.62	7.1099997	4.1280001	2.523	P_KWMTBOMO05987	P_KWMTBOMO05987	P_KWMTBOMO05987	1
275	1.62	1.62	3.0270001	0.9461	0.9461	P_KWMTBOMO01790	P_KWMTBOMO01790	P_KWMTBOMO01790	1
276	1.61	1.61	13.070001	5.5050001	2.523	P_KWMTBOMO09591	P_KWMTBOMO09591	P_KWMTBOMO09591	1
277	1.6	1.6	3.125	3.125	3.125	P_KWMTBOMO08022	P_KWMTBOMO08022	P_KWMTBOMO08022	1
278	1.57	3.45	4.287	3.3599999	3.3599999	P_KWMTBOMO14157	P_KWMTBOMO14157	P_KWMTBOMO14157	2
279	1.56	2.96	58.039999	41.960001	41.960001	P_KWMTBOMO09230	P_KWMTBOMO09230	P_KWMTBOMO09230	3
280	1.54	1.54	6.9590002	1.7130001	1.7130001	P_KWMTBOMO06603	P_KWMTBOMO06603	P_KWMTBOMO06603	1
281	1.51	1.51	6.3320003	3.9579999	3.9579999	P_KWMTBOMO04413	P_KWMTBOMO04413	P_KWMTBOMO04413	1
282	1.46	1.46	4.9109999	3.2740001	1.488	P_KWMTBOMO08865	P_KWMTBOMO08865	P_KWMTBOMO08865	1
283	1.46	1.46	2.012	2.012	2.012	P_KWMTBOMO02096	P_KWMTBOMO02096	P_KWMTBOMO02096	1
284	1.43	1.43	4.6149999	1.41	1.41	P_KWMTBOMO07874	P_KWMTBOMO07874	P_KWMTBOMO07874	1
285	1.43	1.43	6.3380003	2.8170001	2.8170001	P_KWMTBOMO04769	P_KWMTBOMO04769	P_KWMTBOMO04769	1
286	1.43	1.43	6.2650003	2.5520001	2.5520001	P_KWMTBOMO02223	P_KWMTBOMO02223	P_KWMTBOMO02223	1
288	1.41	1.41	6.202	3.359	3.359	P_KWMTBOMO11394	P_KWMTBOMO11394	P_KWMTBOMO11394	1
289	1.36	1.36	10.58	3.6210001	3.6210001	P_KWMTBOMO02894	P_KWMTBOMO02894	P_KWMTBOMO02894	1
290	1.35	1.35	3.7689999	0.3168	0.3168	P_KWMTBOMO11519	P_KWMTBOMO11519	P_KWMTBOMO11519	1
291	1.3	1.3	12.630001	5.263	5.263	P_KWMTBOMO12018	P_KWMTBOMO12018	P_KWMTBOMO12018	1

Summary of the identified PIPs of P25_L5D5

N	Unused	Total	%Cov	%Cov(50)	%Cov(95)	Accession	Citable Accession	Name	Peptides(95%)
1	51.46	51.46	38.88	35.370001	31.54	P_KWMTBOMO01927	P_KWMTBOMO01927	P_KWMTBOMO01927	35
3	43.93	43.93	31.209999	29.159999	25.7	P_KWMTBOMO09597	P_KWMTBOMO09597	P_KWMTBOMO09597	27
4	42.52	42.52	30.590001	23.810001	21.070001	P_KWMTBOMO05687	P_KWMTBOMO05687	P_KWMTBOMO05687	26
6	39.25	39.25	35.699999	28.600001	26.460001	P_KWMTBOMO00462	P_KWMTBOMO00462	P_KWMTBOMO00462	25
14	29.68	29.72	51.889998	51.889998	46.540001	P_KWMTBOMO05178	P_KWMTBOMO05178	P_KWMTBOMO05178	18

15	28.76	36.56	34.810001	31.299999	25.799999	P_KWMTBOMO08642	P_KWMTBOMO08642	P_KWMTBOMO08642	24
18	27.72	27.72	25.780001	18.15	17.34	P_KWMTBOMO06595	P_KWMTBOMO06595	P_KWMTBOMO06595	14
19	26.03	26.03	31.77	29.170001	23.819999	P_KWMTBOMO11096	P_KWMTBOMO11096	P_KWMTBOMO11096	17
20	24.71	24.71	28.7	22.1	16.1	P_KWMTBOMO13293	P_KWMTBOMO13293	P_KWMTBOMO13293	16
21	24.56	24.56	63.499999	47.150001	42.590001	P_KWMTBOMO13455	P_KWMTBOMO13455	P_KWMTBOMO13455	13
24	22.57	22.57	36.809999	36.809999	34.619999	P_KWMTBOMO09553	P_KWMTBOMO09553	P_KWMTBOMO09553	14
25	22.09	22.09	19.499999	15.12	14.129999	P_KWMTBOMO15159	P_KWMTBOMO15159	P_KWMTBOMO15159	12
26	21.82	21.82	50.319999	44.620001	44.299999	P_KWMTBOMO09300	P_KWMTBOMO09300	P_KWMTBOMO09300	15
27	21.44	21.44	38.139999	35.030001	32.589999	P_KWMTBOMO03092	P_KWMTBOMO03092	P_KWMTBOMO03092	14
28	21.21	21.21	34.279999	34.040001	26.24	P_KWMTBOMO11083	P_KWMTBOMO11083	P_KWMTBOMO11083	11
30	20.69	20.69	22.669999	19.239999	17.900001	P_KWMTBOMO15198	P_KWMTBOMO15198	P_KWMTBOMO15198	16
31	20.55	22.35	29.58	29.58	24.05	P_KWMTBOMO12560	P_KWMTBOMO12560	P_KWMTBOMO12560	11
32	20.52	20.52	15.85	12.800001	10.6	P_KWMTBOMO09055	P_KWMTBOMO09055	P_KWMTBOMO09055	11
34	19.85	19.85	42.269999	38.139999	34.020001	P_KWMTBOMO11255	P_KWMTBOMO11255	P_KWMTBOMO11255	11
35	19.47	19.47	40.149999	34.57	23.79	P_KWMTBOMO11985	P_KWMTBOMO11985	P_KWMTBOMO11985	12
37	19.2	19.2	60.259998	54.970002	54.970002	P_KWMTBOMO08440	P_KWMTBOMO08440	P_KWMTBOMO08440	11
39	18.55	18.55	24.46	23.74	17.990001	P_KWMTBOMO15357	P_KWMTBOMO15357	P_KWMTBOMO15357	11
40	18.54	18.54	16.37	14.77	11.58	P_KWMTBOMO05541	P_KWMTBOMO05541	P_KWMTBOMO05541	10
41	18.24	18.24	36.629999	25.150001	25.150001	P_KWMTBOMO07374	P_KWMTBOMO07374	P_KWMTBOMO07374	12
42	18.16	18.16	29.38	24.67	21.66	P_KWMTBOMO09808	P_KWMTBOMO09808	P_KWMTBOMO09808	10
44	17.65	17.65	28.760001	27.090001	24.680001	P_KWMTBOMO01075	P_KWMTBOMO01075	P_KWMTBOMO01075	11
47	17.29	17.29	14.86	12.67	11.81	P_KWMTBOMO00638	P_KWMTBOMO00638	P_KWMTBOMO00638	9
48	17.04	17.04	8.1309997	3.8570002	3.4049999	P_KWMTBOMO07761	P_KWMTBOMO07761	P_KWMTBOMO07761	8
49	16.85	16.85	24.250001	21.46	21.46	P_KWMTBOMO07855	P_KWMTBOMO07855	P_KWMTBOMO07855	10
51	16.23	16.23	10.87	6.6469997	4.5150001	P_KWMTBOMO08648	P_KWMTBOMO08648	P_KWMTBOMO08648	8
52	16.12	16.12	14.72	9.9830002	9.2220001	P_KWMTBOMO10816	P_KWMTBOMO10816	P_KWMTBOMO10816	8
54	16.02	16.02	26.49	22.01	22.01	P_KWMTBOMO08699	P_KWMTBOMO08699	P_KWMTBOMO08699	11
57	15.47	15.47	46.740001	37.16	34.869999	P_KWMTBOMO07688	P_KWMTBOMO07688	P_KWMTBOMO07688	10
58	15.37	15.37	30.509999	27.790001	25.68	P_KWMTBOMO07103	P_KWMTBOMO07103	P_KWMTBOMO07103	9
59	15.3	15.3	15.65	13.789999	9.9380001	P_KWMTBOMO11666	P_KWMTBOMO11666	P_KWMTBOMO11666	7
61	15.19	15.19	33.78	31.420001	21.28	P_KWMTBOMO16197	P_KWMTBOMO16197	P_KWMTBOMO16197	6
63	15.02	15.02	17.17	15.15	14.139999	P_KWMTBOMO16000	P_KWMTBOMO16000	P_KWMTBOMO16000	7
64	14.98	14.98	8.2110003	5.9629999	4.3499999	P_KWMTBOMO12878	P_KWMTBOMO12878	P_KWMTBOMO12878	8
65	14.84	14.84	12.13	9.3230002	6.436	P_KWMTBOMO12628	P_KWMTBOMO12628	P_KWMTBOMO12628	7
66	14.8	14.8	34.040001	26.480001	24.590001	P_KWMTBOMO00375	P_KWMTBOMO00375	P_KWMTBOMO00375	7
67	14.54	14.54	24.950001	24.950001	18.66	P_KWMTBOMO15675	P_KWMTBOMO15675	P_KWMTBOMO15675	9
68	14.52	14.52	15.530001	14.71	10.76	P_KWMTBOMO03430	P_KWMTBOMO03430	P_KWMTBOMO03430	8
69	14.38	14.38	36.320001	32.409999	21.84	P_KWMTBOMO09054	P_KWMTBOMO09054	P_KWMTBOMO09054	8
70	13.97	13.97	20	20	18.02	P_KWMTBOMO03626	P_KWMTBOMO03626	P_KWMTBOMO03626	9
71	13.86	13.86	27.14	20.22	15.3	P_KWMTBOMO00927	P_KWMTBOMO00927	P_KWMTBOMO00927	6
72	13.85	13.85	43.020001	34.88	30.809999	P_KWMTBOMO02278	P_KWMTBOMO02278	P_KWMTBOMO02278	8
73	13.62	13.62	62.120003	62.120003	50	P_KWMTBOMO09788	P_KWMTBOMO09788	P_KWMTBOMO09788	7
75	13.11	13.11	30.399999	30.039999	19.050001	P_KWMTBOMO09442	P_KWMTBOMO09442	P_KWMTBOMO09442	8
76	13.03	13.03	12.710001	12.710001	9.1159999	P_KWMTBOMO15359	P_KWMTBOMO15359	P_KWMTBOMO15359	6
77	12.99	12.99	25.99	25.99	18.65	P_KWMTBOMO00497	P_KWMTBOMO00497	P_KWMTBOMO00497	6
78	12.97	12.97	38.240001	31.029999	20.379999	P_KWMTBOMO01148	P_KWMTBOMO01148	P_KWMTBOMO01148	10
79	12.89	12.89	25.459999	23.800001	12.18	P_KWMTBOMO14887	P_KWMTBOMO14887	P_KWMTBOMO14887	6
80	12.64	12.64	47.139999	40.000001	40.000001	P_KWMTBOMO00838	P_KWMTBOMO00838	P_KWMTBOMO00838	10

82	12.55	12.55	20.71	20.71	20.71	P_KWMTBOMO02081	P_KWMTBOMO02081	P_KWMTBOMO02081	10
84	12.45	12.45	30.809999	24.49	21.21	P_KWMTBOMO15518	P_KWMTBOMO15518	P_KWMTBOMO15518	7
85	12.31	12.31	21.33	14.37	12.83	P_KWMTBOMO13370	P_KWMTBOMO13370	P_KWMTBOMO13370	9
86	12.18	12.18	27.12	27.12	26.800001	P_KWMTBOMO10248	P_KWMTBOMO10248	P_KWMTBOMO10248	8
87	12.03	12.03	24.160001	21.41	16.82	P_KWMTBOMO06763	P_KWMTBOMO06763	P_KWMTBOMO06763	6
89	12	12	37.39	37.39	37.39	P_KWMTBOMO01424	P_KWMTBOMO01424	P_KWMTBOMO01424	11
90	11.92	11.92	12.43	9.0559997	7.418	P_KWMTBOMO04768	P_KWMTBOMO04768	P_KWMTBOMO04768	6
91	11.87	11.87	33.590001	33.590001	30.53	P_KWMTBOMO08464	P_KWMTBOMO08464	P_KWMTBOMO08464	11
92	11.8	11.8	31.459999	29.47	25.830001	P_KWMTBOMO12389	P_KWMTBOMO12389	P_KWMTBOMO12389	8
93	11.68	11.68	28.259999	20.370001	10.28	P_KWMTBOMO13721	P_KWMTBOMO13721	P_KWMTBOMO13721	5
94	11.59	11.59	13.45	10.71	10.71	P_KWMTBOMO12895	P_KWMTBOMO12895	P_KWMTBOMO12895	9
95	11.53	11.53	16.8	9.3359999	8.1950001	P_KWMTBOMO05741	P_KWMTBOMO05741	P_KWMTBOMO05741	6
96	11.41	11.41	17.89	15.530001	15.530001	P_KWMTBOMO01720	P_KWMTBOMO01720	P_KWMTBOMO01720	7
97	11.35	11.35	20.59	17.81	15.85	P_KWMTBOMO15928	P_KWMTBOMO15928	P_KWMTBOMO15928	6
98	11.34	11.35	42.399999	42.399999	35.94	P_KWMTBOMO09232	P_KWMTBOMO09232	P_KWMTBOMO09232	9
100	11.16	11.16	39.89	37.16	37.16	P_KWMTBOMO06480	P_KWMTBOMO06480	P_KWMTBOMO06480	8
101	11.04	11.04	19.02	17.95	16.45	P_KWMTBOMO02470	P_KWMTBOMO02470	P_KWMTBOMO02470	6
102	10.99	10.99	30.410001	26.269999	26.269999	P_KWMTBOMO08596	P_KWMTBOMO08596	P_KWMTBOMO08596	6
103	10.93	10.93	46.529999	41.580001	34.650001	P_KWMTBOMO15361	P_KWMTBOMO15361	P_KWMTBOMO15361	6
104	10.9	10.9	58.19	46.89	31.639999	P_KWMTBOMO13800	P_KWMTBOMO13800	P_KWMTBOMO13800	6
105	10.86	10.86	28.95	25.79	20.53	P_KWMTBOMO12018	P_KWMTBOMO12018	P_KWMTBOMO12018	5
106	10.85	10.85	13.88	12.48	11.08	P_KWMTBOMO11336	P_KWMTBOMO11336	P_KWMTBOMO11336	6
107	10.71	12.76	26.809999	26.809999	24.699999	P_KWMTBOMO01182	P_KWMTBOMO01182	P_KWMTBOMO01182	7
108	10.65	10.65	10.24	8.867	7.4040003	P_KWMTBOMO09183	P_KWMTBOMO09183	P_KWMTBOMO09183	6
110	10.52	10.52	25.63	17.839999	15.83	P_KWMTBOMO12166	P_KWMTBOMO12166	P_KWMTBOMO12166	5
111	10.51	10.52	24.49	23.109999	18.99	P_KWMTBOMO06660	P_KWMTBOMO06660	P_KWMTBOMO06660	6
112	10.39	10.39	35.780001	31.09	20.53	P_KWMTBOMO09474	P_KWMTBOMO09474	P_KWMTBOMO09474	5
113	10.16	10.16	44.119999	44.119999	38.240001	P_KWMTBOMO13569	P_KWMTBOMO13569	P_KWMTBOMO13569	6
114	10.1	10.1	47.799999	40.880001	40.880001	P_KWMTBOMO08100	P_KWMTBOMO08100	P_KWMTBOMO08100	6
115	9.96	9.96	20.88	18.16	13.99	P_KWMTBOMO08956	P_KWMTBOMO08956	P_KWMTBOMO08956	5
116	9.89	9.89	42.860001	40.93	37.84	P_KWMTBOMO00339	P_KWMTBOMO00339	P_KWMTBOMO00339	6
117	9.86	9.86	32.2	23.84	19.2	P_KWMTBOMO03986	P_KWMTBOMO03986	P_KWMTBOMO03986	5
118	9.75	9.75	11.38	11.38	6.0389999	P_KWMTBOMO00427	P_KWMTBOMO00427	P_KWMTBOMO00427	6
119	9.72	9.72	7.0110001	4.3480001	3.424	P_KWMTBOMO12285	P_KWMTBOMO12285	P_KWMTBOMO12285	8
120	9.68	9.68	31.470001	31.470001	22.38	P_KWMTBOMO08666	P_KWMTBOMO08666	P_KWMTBOMO08666	6
121	9.58	9.58	19.069999	15.369999	8.704	P_KWMTBOMO12659	P_KWMTBOMO12659	P_KWMTBOMO12659	4
122	9.55	9.55	17.659999	17.659999	14.929999	P_KWMTBOMO12461	P_KWMTBOMO12461	P_KWMTBOMO12461	6
123	9.45	9.45	26.47	26.47	26.47	P_KWMTBOMO02734	P_KWMTBOMO02734	P_KWMTBOMO02734	6
125	9.35	9.35	10.43	8.574	6.5080002	P_KWMTBOMO07589	P_KWMTBOMO07589	P_KWMTBOMO07589	4
126	9.35	9.35	13.82	10.81	10.81	P_KWMTBOMO01423	P_KWMTBOMO01423	P_KWMTBOMO01423	5
127	9.18	9.18	36.129999	31.389999	22.63	P_KWMTBOMO01796	P_KWMTBOMO01796	P_KWMTBOMO01796	4
128	9.14	9.14	27.57	27.57	23.459999	P_KWMTBOMO04168	P_KWMTBOMO04168	P_KWMTBOMO04168	5
129	8.96	8.96	8.0909997	5.2859999	3.6680002	P_KWMTBOMO04610	P_KWMTBOMO04610	P_KWMTBOMO04610	4
130	8.91	8.91	36.489999	36.489999	36.489999	P_KWMTBOMO14440	P_KWMTBOMO14440	P_KWMTBOMO14440	6
131	8.89	8.89	12.890001	12.890001	12.890001	P_KWMTBOMO08040	P_KWMTBOMO08040	P_KWMTBOMO08040	7
132	8.84	8.84	58.410001	58.410001	41.589999	P_KWMTBOMO08961	P_KWMTBOMO08961	P_KWMTBOMO08961	5
133	8.78	8.78	12.69	10.12	8.7480001	P_KWMTBOMO03301	P_KWMTBOMO03301	P_KWMTBOMO03301	4
134	8.76	8.76	17.209999	14.15	8.6039998	P_KWMTBOMO08041	P_KWMTBOMO08041	P_KWMTBOMO08041	5

135	8.68	8.68	17.209999	14.569999	13.160001	P_KWMTBOMO11680	P_KWMTBOMO11680	P_KWMTBOMO11680	8
136	8.61	8.61	13.99	9.1909997	8.0920003	P_KWMTBOMO00722	P_KWMTBOMO00722	P_KWMTBOMO00722	6
137	8.57	8.57	19.78	13.76	13.76	P_KWMTBOMO00279	P_KWMTBOMO00279	P_KWMTBOMO00279	5
139	8.5	8.51	5.6620002	5.6620002	4.7490001	P_KWMTBOMO04906	P_KWMTBOMO04906	P_KWMTBOMO04906	4
141	8.37	8.37	30.520001	24.41	20.66	P_KWMTBOMO16481	P_KWMTBOMO16481	P_KWMTBOMO16481	4
142	8.17	8.17	40.400001	33.770001	23.18	P_KWMTBOMO13960	P_KWMTBOMO13960	P_KWMTBOMO13960	4
143	8.15	8.15	15.719999	15.719999	9.5320001	P_KWMTBOMO04502	P_KWMTBOMO04502	P_KWMTBOMO04502	4
144	8.1	8.1	7.7189997	3.108	3.108	P_KWMTBOMO08584	P_KWMTBOMO08584	P_KWMTBOMO08584	4
145	8.07	8.07	15.04	12.970001	10.71	P_KWMTBOMO03722	P_KWMTBOMO03722	P_KWMTBOMO03722	4
146	8.05	8.05	37.43	24.600001	24.600001	P_KWMTBOMO12428	P_KWMTBOMO12428	P_KWMTBOMO12428	6
147	8.03	8.03	36.590001	35.769999	35.769999	P_KWMTBOMO12287	P_KWMTBOMO12287	P_KWMTBOMO12287	5
148	8	8	1.223	1.071	1.071	P_KWMTBOMO15365	P_KWMTBOMO15365	P_KWMTBOMO15365	5
149	8	8	11.06	11.06	11.06	P_KWMTBOMO09415	P_KWMTBOMO09415	P_KWMTBOMO09415	6
150	8	8	13.23	13.23	13.23	P_KWMTBOMO02223	P_KWMTBOMO02223	P_KWMTBOMO02223	4
151	7.92	7.92	10.93	10.93	10.93	P_KWMTBOMO08204	P_KWMTBOMO08204	P_KWMTBOMO08204	4
152	7.9	7.9	19.159999	11.92	11.92	P_KWMTBOMO14984	P_KWMTBOMO14984	P_KWMTBOMO14984	6
153	7.88	7.88	18.48	10.85	10.85	P_KWMTBOMO04605	P_KWMTBOMO04605	P_KWMTBOMO04605	4
154	7.88	7.88	38.420001	38.420001	34.209999	P_KWMTBOMO03113	P_KWMTBOMO03113	P_KWMTBOMO03113	5
157	7.7	7.7	7.6269999	5.508	3.644	P_KWMTBOMO09542	P_KWMTBOMO09542	P_KWMTBOMO09542	4
158	7.64	8.78	18.92	16.13	15.48	P_KWMTBOMO14761	P_KWMTBOMO14761	P_KWMTBOMO14761	6
159	7.49	7.49	15.73	10.97	10.97	P_KWMTBOMO00473	P_KWMTBOMO00473	P_KWMTBOMO00473	4
160	7.47	7.47	29.409999	29.409999	29.409999	P_KWMTBOMO11021	P_KWMTBOMO11021	P_KWMTBOMO11021	5
161	7.46	7.46	17.69	13.97	12.1	P_KWMTBOMO07323	P_KWMTBOMO07323	P_KWMTBOMO07323	4
162	7.44	7.44	33.82	24.510001	15.2	P_KWMTBOMO03314	P_KWMTBOMO03314	P_KWMTBOMO03314	4
163	7.37	7.37	15.270001	11.41	9.369	P_KWMTBOMO03002	P_KWMTBOMO03002	P_KWMTBOMO03002	4
165	7.31	7.31	6.5329999	5.7289999	4.9249999	P_KWMTBOMO10780	P_KWMTBOMO10780	P_KWMTBOMO10780	4
166	7.23	7.23	17.640001	15.700001	11.05	P_KWMTBOMO06354	P_KWMTBOMO06354	P_KWMTBOMO06354	5
167	7.22	7.23	13.45	10.69	7.0689999	P_KWMTBOMO02088	P_KWMTBOMO02088	P_KWMTBOMO02088	3
168	7.13	7.13	9.8229997	5.8660001	5.8660001	P_KWMTBOMO03177	P_KWMTBOMO03177	P_KWMTBOMO03177	4
169	7.1	7.1	14.579999	11.81	7.1960002	P_KWMTBOMO00793	P_KWMTBOMO00793	P_KWMTBOMO00793	4
170	7.08	7.08	10.1	8.3460003	5.4170001	P_KWMTBOMO01452	P_KWMTBOMO01452	P_KWMTBOMO01452	3
171	7.06	7.06	35.249999	29.499999	28.780001	P_KWMTBOMO08399	P_KWMTBOMO08399	P_KWMTBOMO08399	4
172	7.05	7.06	11.33	10.63	7.5520001	P_KWMTBOMO15060	P_KWMTBOMO15060	P_KWMTBOMO15060	5
173	6.99	6.99	14.55	9.8870002	6.3560002	P_KWMTBOMO09050	P_KWMTBOMO09050	P_KWMTBOMO09050	3
174	6.97	6.97	8.1019998	6.944	4.5139998	P_KWMTBOMO08815	P_KWMTBOMO08815	P_KWMTBOMO08815	4
175	6.96	6.96	28.850001	18.59	18.59	P_KWMTBOMO16357	P_KWMTBOMO16357	P_KWMTBOMO16357	4
176	6.93	6.93	23.13	19.37	19.37	P_KWMTBOMO07772	P_KWMTBOMO07772	P_KWMTBOMO07772	4
177	6.81	6.81	20.739999	20.739999	17.06	P_KWMTBOMO11061	P_KWMTBOMO11061	P_KWMTBOMO11061	5
178	6.7	6.7	27.669999	23.33	10.33	P_KWMTBOMO14732	P_KWMTBOMO14732	P_KWMTBOMO14732	3
180	6.68	6.68	5.762	3.6309998	3.6309998	P_KWMTBOMO03067	P_KWMTBOMO03067	P_KWMTBOMO03067	4
181	6.65	6.65	29.679999	23.74	17.35	P_KWMTBOMO05979	P_KWMTBOMO05979	P_KWMTBOMO05979	5
182	6.65	6.65	4.3430001	2.7960001	2.32	P_KWMTBOMO09747	P_KWMTBOMO09747	P_KWMTBOMO09747	5
183	6.56	6.56	32.640001	23.32	19.17	P_KWMTBOMO05281	P_KWMTBOMO05281	P_KWMTBOMO05281	4
184	6.55	6.55	20.88	20.88	15.260001	P_KWMTBOMO02185	P_KWMTBOMO02185	P_KWMTBOMO02185	3
185	6.39	8.45	8.4590003	7.8790002	6.4889997	P_KWMTBOMO14157	P_KWMTBOMO14157	P_KWMTBOMO14157	4
186	6.36	6.36	19.04	10.91	10.91	P_KWMTBOMO06999	P_KWMTBOMO06999	P_KWMTBOMO06999	4
187	6.34	6.34	9.8530002	7.4749999	6.4549997	P_KWMTBOMO08045	P_KWMTBOMO08045	P_KWMTBOMO08045	4
188	6.34	6.34	48.480001	41.409999	41.409999	P_KWMTBOMO02431	P_KWMTBOMO02431	P_KWMTBOMO02431	5

189	6.23	6.23	29.229999	24.1	18.46	P_KWMTBOMO09355	P_KWMTBOMO09355	P_KWMTBOMO09355	3
190	6.14	6.14	13.81	10.62	9.0269998	P_KWMTBOMO08578	P_KWMTBOMO08578	P_KWMTBOMO08578	3
191	6.06	6.06	18.430001	15.69	15.69	P_KWMTBOMO10916	P_KWMTBOMO10916	P_KWMTBOMO10916	5
192	6.02	6.02	9.8920003	8.3870001	8.3870001	P_KWMTBOMO05564	P_KWMTBOMO05564	P_KWMTBOMO05564	3
193	6.01	6.01	25	20.739999	20.739999	P_KWMTBOMO10453	P_KWMTBOMO10453	P_KWMTBOMO10453	3
194	6	6	17.14	9.6100003	9.6100003	P_KWMTBOMO14041	P_KWMTBOMO14041	P_KWMTBOMO14041	3
195	6	6	9.967	6.0460001	6.0460001	P_KWMTBOMO10779	P_KWMTBOMO10779	P_KWMTBOMO10779	4
196	6	6	31.18	18.82	18.82	P_KWMTBOMO02836	P_KWMTBOMO02836	P_KWMTBOMO02836	3
197	6	6	16.46	10.57	10.57	P_KWMTBOMO02835	P_KWMTBOMO02835	P_KWMTBOMO02835	3
198	6	6	12.06	9.9200003	9.9200003	P_KWMTBOMO06231	P_KWMTBOMO06231	P_KWMTBOMO06231	3
199	6	6	17.54	17.54	17.54	P_KWMTBOMO15757	P_KWMTBOMO15757	P_KWMTBOMO15757	3
200	6	6	8.1940003	8.1940003	8.1940003	P_KWMTBOMO12208	P_KWMTBOMO12208	P_KWMTBOMO12208	3
201	6	6	13.66	13.66	13.66	P_KWMTBOMO01431	P_KWMTBOMO01431	P_KWMTBOMO01431	3
202	6	6	16.83	16.83	16.83	P_KWMTBOMO00637	P_KWMTBOMO00637	P_KWMTBOMO00637	3
203	5.96	5.96	10.38	10.38	8.354	P_KWMTBOMO08310	P_KWMTBOMO08310	P_KWMTBOMO08310	3
204	5.95	5.95	5.2579999	2.0500001	1.6489999	P_KWMTBOMO05810	P_KWMTBOMO05810	P_KWMTBOMO05810	4
205	5.92	5.92	4.747	4.747	3.7969999	P_KWMTBOMO08343	P_KWMTBOMO08343	P_KWMTBOMO08343	3
206	5.88	5.88	14.560001	8.6960003	7.1829997	P_KWMTBOMO01606	P_KWMTBOMO01606	P_KWMTBOMO01606	3
207	5.84	5.84	5.3619999	5.3619999	5.3619999	P_KWMTBOMO14549	P_KWMTBOMO14549	P_KWMTBOMO14549	4
208	5.82	5.82	15.2	10.69	10.69	P_KWMTBOMO08893	P_KWMTBOMO08893	P_KWMTBOMO08893	3
209	5.82	5.82	20.54	17.86	17.86	P_KWMTBOMO04740	P_KWMTBOMO04740	P_KWMTBOMO04740	3
210	5.8	5.8	8.2290001	5.8600001	5.8600001	P_KWMTBOMO01072	P_KWMTBOMO01072	P_KWMTBOMO01072	3
211	5.7	5.7	33.539999	28.479999	28.479999	P_KWMTBOMO14479	P_KWMTBOMO14479	P_KWMTBOMO14479	3
212	5.69	5.69	32.210001	26.85	26.85	P_KWMTBOMO13520	P_KWMTBOMO13520	P_KWMTBOMO13520	3
213	5.68	5.68	43.14	30.720001	24.84	P_KWMTBOMO08820	P_KWMTBOMO08820	P_KWMTBOMO08820	6
214	5.64	5.64	20.09	19.63	15.530001	P_KWMTBOMO08894	P_KWMTBOMO08894	P_KWMTBOMO08894	4
215	5.62	5.62	13.62	9.5370002	9.5370002	P_KWMTBOMO04370	P_KWMTBOMO04370	P_KWMTBOMO04370	3
216	5.62	5.62	22.130001	15.979999	15.979999	P_KWMTBOMO10310	P_KWMTBOMO10310	P_KWMTBOMO10310	3
217	5.6	5.6	17.94	13.72	10.03	P_KWMTBOMO05258	P_KWMTBOMO05258	P_KWMTBOMO05258	3
218	5.53	5.53	9.3960002	4.6020001	3.5470001	P_KWMTBOMO04806	P_KWMTBOMO04806	P_KWMTBOMO04806	3
219	5.49	5.49	6.769	2.557	1.542	P_KWMTBOMO06045	P_KWMTBOMO06045	P_KWMTBOMO06045	3
220	5.48	5.48	12.43	12.43	12.43	P_KWMTBOMO10129	P_KWMTBOMO10129	P_KWMTBOMO10129	3
221	5.44	5.44	7.581	5.415	5.415	P_KWMTBOMO09838	P_KWMTBOMO09838	P_KWMTBOMO09838	3
222	5.38	5.38	15.49	10.63	6.9030002	P_KWMTBOMO03311	P_KWMTBOMO03311	P_KWMTBOMO03311	3
223	5.35	5.35	11.09	7.9240002	6.656	P_KWMTBOMO12094	P_KWMTBOMO12094	P_KWMTBOMO12094	3
224	5.31	5.31	48.480001	48.480001	19.39	P_KWMTBOMO05228	P_KWMTBOMO05228	P_KWMTBOMO05228	2
225	5.28	5.28	10.63	4.2860001	4.2860001	P_KWMTBOMO10243	P_KWMTBOMO10243	P_KWMTBOMO10243	3
226	5.28	5.28	19.63	19.63	19.63	P_KWMTBOMO11448	P_KWMTBOMO11448	P_KWMTBOMO11448	3
227	5.24	5.24	45.629999	45.629999	35.92	P_KWMTBOMO06772	P_KWMTBOMO06772	P_KWMTBOMO06772	3
228	5.21	5.21	16	16	16	P_KWMTBOMO09236	P_KWMTBOMO09236	P_KWMTBOMO09236	3
229	5.15	5.15	13.500001	13.500001	11.25	P_KWMTBOMO01312	P_KWMTBOMO01312	P_KWMTBOMO01312	3
230	5.14	5.14	20.42	20.42	9.155	P_KWMTBOMO07373	P_KWMTBOMO07373	P_KWMTBOMO07373	2
231	5.1	5.1	13.38	10.33	4.3979999	P_KWMTBOMO12264	P_KWMTBOMO12264	P_KWMTBOMO12264	2
232	5.06	5.06	17.219999	11.48	11.48	P_KWMTBOMO04003	P_KWMTBOMO04003	P_KWMTBOMO04003	3
233	5.05	5.05	12.74	12.74	7.9620004	P_KWMTBOMO15758	P_KWMTBOMO15758	P_KWMTBOMO15758	3
234	5.04	5.04	36.570001	18.66	18.66	P_KWMTBOMO14639	P_KWMTBOMO14639	P_KWMTBOMO14639	3
235	5.02	5.02	14.52	12.899999	8.7559998	P_KWMTBOMO01114	P_KWMTBOMO01114	P_KWMTBOMO01114	4
237	4.98	4.98	10.65	4.5650002	4.5650002	P_KWMTBOMO07723	P_KWMTBOMO07723	P_KWMTBOMO07723	3

238	4.89	4.89	10.37	5.683	3.5890002	P_KWMTBOMO04051	P_KWMTBOMO04051	P_KWMTBOMO04051	4
239	4.86	4.86	34.150001	33.539999	24.39	P_KWMTBOMO11678	P_KWMTBOMO11678	P_KWMTBOMO11678	3
240	4.83	4.83	25	15.94	10.14	P_KWMTBOMO08046	P_KWMTBOMO08046	P_KWMTBOMO08046	2
241	4.79	4.79	34.799999	18.14	9.804	P_KWMTBOMO01273	P_KWMTBOMO01273	P_KWMTBOMO01273	2
242	4.77	4.77	10.24	10.24	10.24	P_KWMTBOMO07821	P_KWMTBOMO07821	P_KWMTBOMO07821	3
243	4.76	4.76	27.700001	24.32	16.22	P_KWMTBOMO08064	P_KWMTBOMO08064	P_KWMTBOMO08064	3
244	4.74	4.74	6.9229998	6.9229998	4.487	P_KWMTBOMO07874	P_KWMTBOMO07874	P_KWMTBOMO07874	2
245	4.67	4.67	28.02	17.479999	8.997	P_KWMTBOMO05464	P_KWMTBOMO05464	P_KWMTBOMO05464	3
246	4.65	4.65	9.375	6.9849998	6.9849998	P_KWMTBOMO10337	P_KWMTBOMO10337	P_KWMTBOMO10337	3
247	4.63	4.63	18.31	7.0940003	7.0940003	P_KWMTBOMO14530	P_KWMTBOMO14530	P_KWMTBOMO14530	3
248	4.6	4.6	11.29	7.7229999	7.7229999	P_KWMTBOMO00982	P_KWMTBOMO00982	P_KWMTBOMO00982	3
249	4.59	6.82	34.93	25.36	16.27	P_KWMTBOMO03027	P_KWMTBOMO03027	P_KWMTBOMO03027	3
250	4.52	4.52	6.8049997	4.383	4.383	P_KWMTBOMO02535	P_KWMTBOMO02535	P_KWMTBOMO02535	2
251	4.51	4.51	5.8639999	4.6909999	2.452	P_KWMTBOMO12667	P_KWMTBOMO12667	P_KWMTBOMO12667	2
252	4.49	4.49	14.07	7.88	4.5030002	P_KWMTBOMO06770	P_KWMTBOMO06770	P_KWMTBOMO06770	2
253	4.45	4.45	18.49	14.34	11.32	P_KWMTBOMO11780	P_KWMTBOMO11780	P_KWMTBOMO11780	2
254	4.42	4.42	9.2380002	4.9849998	3.9590001	P_KWMTBOMO05321	P_KWMTBOMO05321	P_KWMTBOMO05321	2
255	4.39	4.39	32.859999	28.569999	28.569999	P_KWMTBOMO10049	P_KWMTBOMO10049	P_KWMTBOMO10049	2
256	4.36	4.36	39.500001	26.890001	19.329999	P_KWMTBOMO15114	P_KWMTBOMO15114	P_KWMTBOMO15114	3
257	4.35	4.35	2.641	1.086	0.6171	P_KWMTBOMO12602	P_KWMTBOMO12602	P_KWMTBOMO12602	2
258	4.31	4.31	14.98	14.98	11.59	P_KWMTBOMO08106	P_KWMTBOMO08106	P_KWMTBOMO08106	2
259	4.3	4.3	13.45	13.45	10.31	P_KWMTBOMO09900	P_KWMTBOMO09900	P_KWMTBOMO09900	2
260	4.29	4.29	4.7150001	2.758	2.224	P_KWMTBOMO05295	P_KWMTBOMO05295	P_KWMTBOMO05295	2
262	4.25	4.25	3.8199998	3.8199998	2.84	P_KWMTBOMO13235	P_KWMTBOMO13235	P_KWMTBOMO13235	2
263	4.24	4.24	13.950001	10.3	6.6519998	P_KWMTBOMO02500	P_KWMTBOMO02500	P_KWMTBOMO02500	2
264	4.22	4.22	7.277	7.277	5.6340002	P_KWMTBOMO11413	P_KWMTBOMO11413	P_KWMTBOMO11413	2
265	4.21	4.21	14.76	7.6190002	4.603	P_KWMTBOMO07205	P_KWMTBOMO07205	P_KWMTBOMO07205	2
266	4.07	4.07	14.29	6.9260001	6.9260001	P_KWMTBOMO15547	P_KWMTBOMO15547	P_KWMTBOMO15547	2
267	4.06	4.06	14.920001	9.1530003	4.7460001	P_KWMTBOMO15769	P_KWMTBOMO15769	P_KWMTBOMO15769	2
268	4.06	4.06	23.05	12.41	8.1560001	P_KWMTBOMO00362	P_KWMTBOMO00362	P_KWMTBOMO00362	2
269	4.06	4.06	8.2220003	6.222	6.222	P_KWMTBOMO10283	P_KWMTBOMO10283	P_KWMTBOMO10283	2
270	4.05	4.05	15.95	7.1429998	7.1429998	P_KWMTBOMO16204	P_KWMTBOMO16204	P_KWMTBOMO16204	2
271	4.04	4.04	7.8539997	1.9640001	1.9640001	P_KWMTBOMO08707	P_KWMTBOMO08707	P_KWMTBOMO08707	2
272	4.02	4.02	23.199999	8.247	6.4429998	P_KWMTBOMO14160	P_KWMTBOMO14160	P_KWMTBOMO14160	2
273	4.02	4.02	40.64	29.409999	17.110001	P_KWMTBOMO07857	P_KWMTBOMO07857	P_KWMTBOMO07857	2
274	4.02	4.02	14.219999	6.3210003	6.3210003	P_KWMTBOMO14804	P_KWMTBOMO14804	P_KWMTBOMO14804	2
275	4.02	4.02	12.28	8.0200002	8.0200002	P_KWMTBOMO08241	P_KWMTBOMO08241	P_KWMTBOMO08241	2
276	4.02	4.02	8.14	6.2789999	6.2789999	P_KWMTBOMO02061	P_KWMTBOMO02061	P_KWMTBOMO02061	2
277	4.01	4.01	4.5820002	1.6100001	1.6100001	P_KWMTBOMO15715	P_KWMTBOMO15715	P_KWMTBOMO15715	2
278	4.01	4.01	13.680001	6.1969999	6.1969999	P_KWMTBOMO13729	P_KWMTBOMO13729	P_KWMTBOMO13729	2
279	4	4	9.3120001	6.4779997	6.4779997	P_KWMTBOMO05993	P_KWMTBOMO05993	P_KWMTBOMO05993	2
280	4	4	14.55	9.6969999	9.6969999	P_KWMTBOMO02914	P_KWMTBOMO02914	P_KWMTBOMO02914	2
282	4	4	9.685	9.685	9.685	P_KWMTBOMO16261	P_KWMTBOMO16261	P_KWMTBOMO16261	2
283	4	4	4.3880001	4.3880001	4.3880001	P_KWMTBOMO14942	P_KWMTBOMO14942	P_KWMTBOMO14942	2
284	4	4	14.68	14.68	14.68	P_KWMTBOMO12448	P_KWMTBOMO12448	P_KWMTBOMO12448	2
285	4	4	35.620001	35.620001	35.620001	P_KWMTBOMO08098	P_KWMTBOMO08098	P_KWMTBOMO08098	3
286	4	4	8.2319997	8.2319997	8.2319997	P_KWMTBOMO06082	P_KWMTBOMO06082	P_KWMTBOMO06082	2
287	4	4	21.65	21.65	21.65	P_KWMTBOMO01918	P_KWMTBOMO01918	P_KWMTBOMO01918	2

288	4	4	16.850001	16.850001	16.850001	P_KWMTBOMO00860	P_KWMTBOMO00860	P_KWMTBOMO00860	2
289	4	4	11.45	11.45	11.45	P_KWMTBOMO00837	P_KWMTBOMO00837	P_KWMTBOMO00837	2
290	3.96	3.96	12.93	12.93	12.93	P_KWMTBOMO11771	P_KWMTBOMO11771	P_KWMTBOMO11771	2
291	3.96	3.96	18.46	18.46	18.46	P_KWMTBOMO06373	P_KWMTBOMO06373	P_KWMTBOMO06373	2
292	3.93	3.93	22.499999	17.5	12.5	P_KWMTBOMO02477	P_KWMTBOMO02477	P_KWMTBOMO02477	4
293	3.92	3.92	4.9589999	4.9589999	4.9589999	P_KWMTBOMO04132	P_KWMTBOMO04132	P_KWMTBOMO04132	2
294	3.89	3.89	20.45	20.45	20.45	P_KWMTBOMO10591	P_KWMTBOMO10591	P_KWMTBOMO10591	2
295	3.86	3.86	5.7739999	4.0539999	2.826	P_KWMTBOMO07820	P_KWMTBOMO07820	P_KWMTBOMO07820	2
296	3.84	3.84	56.840003	56.840003	34.740001	P_KWMTBOMO14478	P_KWMTBOMO14478	P_KWMTBOMO14478	2
297	3.82	3.82	5.192	1.354	1.084	P_KWMTBOMO06632	P_KWMTBOMO06632	P_KWMTBOMO06632	2
298	3.81	3.82	20.93	20.93	20.93	P_KWMTBOMO05723	P_KWMTBOMO05723	P_KWMTBOMO05723	5
299	3.73	6.08	14.2	8.1479996	5.6790002	P_KWMTBOMO12844	P_KWMTBOMO12844	P_KWMTBOMO12844	5
300	3.67	3.67	15.13	15.13	9.2440002	P_KWMTBOMO01068	P_KWMTBOMO01068	P_KWMTBOMO01068	2
301	3.66	3.66	27.239999	24.73	24.73	P_KWMTBOMO06427	P_KWMTBOMO06427	P_KWMTBOMO06427	3
302	3.65	3.65	18.6	7.9070002	7.9070002	P_KWMTBOMO09390	P_KWMTBOMO09390	P_KWMTBOMO09390	2
303	3.64	3.64	4.589	4.589	4.589	P_KWMTBOMO03473	P_KWMTBOMO03473	P_KWMTBOMO03473	2
304	3.6	3.6	3.8090002	1.549	1.549	P_KWMTBOMO06932	P_KWMTBOMO06932	P_KWMTBOMO06932	2
305	3.6	3.6	4.5150001	3.7179999	3.7179999	P_KWMTBOMO04568	P_KWMTBOMO04568	P_KWMTBOMO04568	2
306	3.57	3.57	13.19	8.4250003	8.4250003	P_KWMTBOMO04789	P_KWMTBOMO04789	P_KWMTBOMO04789	2
307	3.57	3.57	8.5600004	8.5600004	8.5600004	P_KWMTBOMO11486	P_KWMTBOMO11486	P_KWMTBOMO11486	2
308	3.51	3.51	52.560002	52.560002	34.619999	P_KWMTBOMO12193	P_KWMTBOMO12193	P_KWMTBOMO12193	2
309	3.51	3.51	5.294	5.294	5.294	P_KWMTBOMO03271	P_KWMTBOMO03271	P_KWMTBOMO03271	2
310	3.44	3.44	34.850001	18.179999	18.179999	P_KWMTBOMO01055	P_KWMTBOMO01055	P_KWMTBOMO01055	3
311	3.35	3.35	22.750001	18.82	14.51	P_KWMTBOMO09934	P_KWMTBOMO09934	P_KWMTBOMO09934	3
312	3.34	3.35	5.7100002	5.7100002	2.7860001	P_KWMTBOMO10358	P_KWMTBOMO10358	P_KWMTBOMO10358	1
313	3.33	3.33	6.9660001	6.9660001	5.618	P_KWMTBOMO02840	P_KWMTBOMO02840	P_KWMTBOMO02840	2
314	3.3	3.3	15.019999	10.28	7.5099997	P_KWMTBOMO15066	P_KWMTBOMO15066	P_KWMTBOMO15066	2
315	3.28	3.28	6.8020001	1.291	1.291	P_KWMTBOMO11045	P_KWMTBOMO11045	P_KWMTBOMO11045	2
316	3.25	3.25	18.07	13.45	10.08	P_KWMTBOMO09387	P_KWMTBOMO09387	P_KWMTBOMO09387	2
317	3.25	3.25	23.53	23.53	23.53	P_KWMTBOMO04674	P_KWMTBOMO04674	P_KWMTBOMO04674	2
318	3.22	3.22	15.350001	15.350001	11.2	P_KWMTBOMO11710	P_KWMTBOMO11710	P_KWMTBOMO11710	2
319	3.22	3.22	16.249999	11.87	11.87	P_KWMTBOMO01131	P_KWMTBOMO01131	P_KWMTBOMO01131	2
320	3.14	3.14	13.62	13.62	9.8590001	P_KWMTBOMO05566	P_KWMTBOMO05566	P_KWMTBOMO05566	2
321	3.14	3.14	6.1730001	6.1730001	6.1730001	P_KWMTBOMO11514	P_KWMTBOMO11514	P_KWMTBOMO11514	2
322	3.13	3.13	45.24	29.76	29.76	P_KWMTBOMO03951	P_KWMTBOMO03951	P_KWMTBOMO03951	3
323	3.1	3.1	24.510001	21.57	21.57	P_KWMTBOMO09631	P_KWMTBOMO09631	P_KWMTBOMO09631	3
324	3.07	3.07	8.9160003	8.9160003	6.7469999	P_KWMTBOMO10965	P_KWMTBOMO10965	P_KWMTBOMO10965	2
325	3.03	3.03	4.177	4.177	2.864	P_KWMTBOMO05357	P_KWMTBOMO05357	P_KWMTBOMO05357	2
326	3.01	5.17	11.66	6.7000002	6.7000002	P_KWMTBOMO12199	P_KWMTBOMO12199	P_KWMTBOMO12199	3
327	2.99	2.99	8.1050001	3.1369999	3.1369999	P_KWMTBOMO13712	P_KWMTBOMO13712	P_KWMTBOMO13712	2
328	2.98	2.98	8.8040002	8.8040002	6.095	P_KWMTBOMO08056	P_KWMTBOMO08056	P_KWMTBOMO08056	2
329	2.95	2.95	21.3	21.3	21.3	P_KWMTBOMO02542	P_KWMTBOMO02542	P_KWMTBOMO02542	2
330	2.92	2.92	21.85	21.85	9.2440002	P_KWMTBOMO12706	P_KWMTBOMO12706	P_KWMTBOMO12706	1
331	2.91	2.91	6.7419998	3.2299999	3.2299999	P_KWMTBOMO13226	P_KWMTBOMO13226	P_KWMTBOMO13226	2
332	2.91	2.91	8.382	6.0430001	2.534	P_KWMTBOMO04170	P_KWMTBOMO04170	P_KWMTBOMO04170	1
333	2.91	2.91	10.48	10.48	10.48	P_KWMTBOMO00254	P_KWMTBOMO00254	P_KWMTBOMO00254	2
334	2.9	2.91	26.980001	20.630001	20.630001	P_KWMTBOMO02082	P_KWMTBOMO02082	P_KWMTBOMO02082	2
335	2.9	2.91	5.6960002	5.6960002	5.6960002	P_KWMTBOMO12442	P_KWMTBOMO12442	P_KWMTBOMO12442	2

336	2.84	2.84	10.5	6.2410001	2.5529999	P_KWMTBOMO15125	P_KWMTBOMO15125	P_KWMTBOMO15125	1
337	2.84	2.84	2.908	2.908	2.908	P_KWMTBOMO05612	P_KWMTBOMO05612	P_KWMTBOMO05612	2
338	2.75	2.75	8.0750003	6.2109999	6.2109999	P_KWMTBOMO10558	P_KWMTBOMO10558	P_KWMTBOMO10558	2
339	2.75	2.75	10.87	10.87	10.87	P_KWMTBOMO05975	P_KWMTBOMO05975	P_KWMTBOMO05975	2
340	2.69	2.69	7.9910003	3.1959999	3.1959999	P_KWMTBOMO09621	P_KWMTBOMO09621	P_KWMTBOMO09621	2
341	2.69	2.69	6.1170001	4.7869999	4.1219998	P_KWMTBOMO06291	P_KWMTBOMO06291	P_KWMTBOMO06291	2
342	2.69	2.69	14.6	14.6	5.7519998	P_KWMTBOMO03975	P_KWMTBOMO03975	P_KWMTBOMO03975	1
343	2.67	2.67	25.59	18.48	7.1089998	P_KWMTBOMO00794	P_KWMTBOMO00794	P_KWMTBOMO00794	1
344	2.66	2.66	7.0119999	3.8400002	2.0029999	P_KWMTBOMO10212	P_KWMTBOMO10212	P_KWMTBOMO10212	1
345	2.65	2.65	6.6869996	6.6869996	3.647	P_KWMTBOMO08776	P_KWMTBOMO08776	P_KWMTBOMO08776	1
346	2.64	4.8	16.93	10.76	5.4919999	P_KWMTBOMO15123	P_KWMTBOMO15123	P_KWMTBOMO15123	3
347	2.63	2.63	21.54	20.77	13.850001	P_KWMTBOMO07368	P_KWMTBOMO07368	P_KWMTBOMO07368	2
348	2.62	2.62	14.73	4.7510002	2.375	P_KWMTBOMO04330	P_KWMTBOMO04330	P_KWMTBOMO04330	1
349	2.61	2.62	9.725	6.9770001	3.805	P_KWMTBOMO10169	P_KWMTBOMO10169	P_KWMTBOMO10169	2
350	2.58	2.7	20.100001	10.05	3.4309998	P_KWMTBOMO05820	P_KWMTBOMO05820	P_KWMTBOMO05820	2
351	2.56	2.56	5.0760001	5.0760001	2.8759999	P_KWMTBOMO05205	P_KWMTBOMO05205	P_KWMTBOMO05205	1
352	2.56	2.56	7.3679999	7.3679999	3.5089999	P_KWMTBOMO03721	P_KWMTBOMO03721	P_KWMTBOMO03721	1
353	2.55	2.55	27.64	21.950001	10.57	P_KWMTBOMO01941	P_KWMTBOMO01941	P_KWMTBOMO01941	2
354	2.52	2.52	14.71	8.8239998	6.6179998	P_KWMTBOMO09672	P_KWMTBOMO09672	P_KWMTBOMO09672	1
355	2.49	2.49	26.550001	15.82	11.3	P_KWMTBOMO01380	P_KWMTBOMO01380	P_KWMTBOMO01380	1
356	2.49	2.49	4.9740002	4.9740002	2.88	P_KWMTBOMO02013	P_KWMTBOMO02013	P_KWMTBOMO02013	1
357	2.48	2.48	11.62	8.8380001	4.5449998	P_KWMTBOMO11192	P_KWMTBOMO11192	P_KWMTBOMO11192	1
358	2.42	2.42	15.090001	7.0749998	7.0749998	P_KWMTBOMO02468	P_KWMTBOMO02468	P_KWMTBOMO02468	2
359	2.42	2.42	2.815	1.542	0.6702	P_KWMTBOMO07214	P_KWMTBOMO07214	P_KWMTBOMO07214	1
360	2.4	2.4	15.700001	4.0449999	2.589	P_KWMTBOMO03630	P_KWMTBOMO03630	P_KWMTBOMO03630	1
361	2.35	2.35	10.67	10.67	5.618	P_KWMTBOMO11812	P_KWMTBOMO11812	P_KWMTBOMO11812	1
362	2.34	2.34	13.850001	13.850001	5.8970001	P_KWMTBOMO16309	P_KWMTBOMO16309	P_KWMTBOMO16309	1
363	2.33	2.33	5.9590001	3.576	2.503	P_KWMTBOMO10661	P_KWMTBOMO10661	P_KWMTBOMO10661	1
364	2.32	2.32	16.28	4.1280001	2.523	P_KWMTBOMO05987	P_KWMTBOMO05987	P_KWMTBOMO05987	1
365	2.3	2.32	22.63	13.680001	13.680001	P_KWMTBOMO14024	P_KWMTBOMO14024	P_KWMTBOMO14024	2
366	2.3	2.3	12.01	5.587	3.3519998	P_KWMTBOMO14715	P_KWMTBOMO14715	P_KWMTBOMO14715	2
367	2.29	2.29	12.88	6.301	3.836	P_KWMTBOMO05806	P_KWMTBOMO05806	P_KWMTBOMO05806	1
368	2.25	2.25	6.3259996	6.3259996	2.6760001	P_KWMTBOMO00390	P_KWMTBOMO00390	P_KWMTBOMO00390	1
369	2.22	2.22	9.0449996	6.03	4.5230001	P_KWMTBOMO12902	P_KWMTBOMO12902	P_KWMTBOMO12902	1
370	2.2	2.2	14.55	6.3429996	6.3429996	P_KWMTBOMO11161	P_KWMTBOMO11161	P_KWMTBOMO11161	2
371	2.19	2.19	10.48	5.6449998	2.823	P_KWMTBOMO12037	P_KWMTBOMO12037	P_KWMTBOMO12037	1
372	2.18	2.18	27.880001	13.46	13.46	P_KWMTBOMO06974	P_KWMTBOMO06974	P_KWMTBOMO06974	1
373	2.18	2.18	8.3329998	8.3329998	5.556	P_KWMTBOMO05719	P_KWMTBOMO05719	P_KWMTBOMO05719	1
374	2.17	2.17	50.889999	43.75	43.75	P_KWMTBOMO09230	P_KWMTBOMO09230	P_KWMTBOMO09230	2
375	2.16	2.17	17.07	8.0140002	4.1809998	P_KWMTBOMO08930	P_KWMTBOMO08930	P_KWMTBOMO08930	1
376	2.16	2.16	13.249999	13.249999	13.249999	P_KWMTBOMO00974	P_KWMTBOMO00974	P_KWMTBOMO00974	2
377	2.15	2.15	9.0910003	6.6430002	3.497	P_KWMTBOMO03860	P_KWMTBOMO03860	P_KWMTBOMO03860	1
378	2.12	2.6	11.06	4.941	3.5289999	P_KWMTBOMO08672	P_KWMTBOMO08672	P_KWMTBOMO08672	1
379	2.08	2.08	4.8730001	1.287	1.287	P_KWMTBOMO12304	P_KWMTBOMO12304	P_KWMTBOMO12304	2
380	2.08	2.08	13.779999	11.54	3.8460001	P_KWMTBOMO15665	P_KWMTBOMO15665	P_KWMTBOMO15665	1
381	2.08	2.08	7.4680001	7.4680001	5.195	P_KWMTBOMO06474	P_KWMTBOMO06474	P_KWMTBOMO06474	1
382	2.07	2.07	14.36	10.37	10.37	P_KWMTBOMO14845	P_KWMTBOMO14845	P_KWMTBOMO14845	2
383	2.07	2.07	2.9039999	2.9039999	1.815	P_KWMTBOMO14936	P_KWMTBOMO14936	P_KWMTBOMO14936	1

384	2.04	2.04	10.58	2.7939999	2.7939999	P_KWMTBOMO15121	P_KWMTBOMO15121	P_KWMTBOMO15121	1
385	2.04	2.04	4.1850001	1.6519999	1.6519999	P_KWMTBOMO11064	P_KWMTBOMO11064	P_KWMTBOMO11064	2
386	2.02	2.02	9.6770003	3.8710002	3.8710002	P_KWMTBOMO13369	P_KWMTBOMO13369	P_KWMTBOMO13369	1
387	2.02	2.02	2.7240001	1.362	1.362	P_KWMTBOMO10489	P_KWMTBOMO10489	P_KWMTBOMO10489	1
388	2.02	2.02	5.3029999	5.3029999	2.424	P_KWMTBOMO09954	P_KWMTBOMO09954	P_KWMTBOMO09954	2
389	2.02	2.02	11.6	11.6	7.9999998	P_KWMTBOMO04427	P_KWMTBOMO04427	P_KWMTBOMO04427	1
390	2.01	2.01	5.0080001	0.9852	0.9852	P_KWMTBOMO04877	P_KWMTBOMO04877	P_KWMTBOMO04877	1
391	2.01	2.01	7.542	2.396	1.508	P_KWMTBOMO01821	P_KWMTBOMO01821	P_KWMTBOMO01821	1
392	2.01	2.01	7.0689999	2.069	2.069	P_KWMTBOMO11590	P_KWMTBOMO11590	P_KWMTBOMO11590	1
393	2.01	2.01	11.72	3.297	3.297	P_KWMTBOMO09641	P_KWMTBOMO09641	P_KWMTBOMO09641	1
394	2.01	2.01	12.41	5.4740001	5.4740001	P_KWMTBOMO05976	P_KWMTBOMO05976	P_KWMTBOMO05976	1
395	2.01	2.01	4.4149999	3.091	3.091	P_KWMTBOMO04737	P_KWMTBOMO04737	P_KWMTBOMO04737	1
396	2	6	16.75	16.75	16.75	P_KWMTBOMO06927	P_KWMTBOMO06927	P_KWMTBOMO06927	3
397	2	2.01	6.278	1.682	1.682	P_KWMTBOMO06100	P_KWMTBOMO06100	P_KWMTBOMO06100	1
398	2	2.01	7.3480003	2.8750001	2.8750001	P_KWMTBOMO05299	P_KWMTBOMO05299	P_KWMTBOMO05299	1
399	2	2.01	4.1760001	3.077	3.077	P_KWMTBOMO15781	P_KWMTBOMO15781	P_KWMTBOMO15781	1
400	2	2	4.1390002	0.8467	0.8467	P_KWMTBOMO06227	P_KWMTBOMO06227	P_KWMTBOMO06227	1
401	2	2	39.19	6.7570001	6.7570001	P_KWMTBOMO07930	P_KWMTBOMO07930	P_KWMTBOMO07930	1
402	2	2	50.569999	22.99	22.99	P_KWMTBOMO13722	P_KWMTBOMO13722	P_KWMTBOMO13722	1
403	2	2	26.620001	6.4750001	6.4750001	P_KWMTBOMO11987	P_KWMTBOMO11987	P_KWMTBOMO11987	1
404	2	2	12.809999	5.2919999	5.2919999	P_KWMTBOMO08607	P_KWMTBOMO08607	P_KWMTBOMO08607	1
405	2	2	3.3909999	1.647	1.647	P_KWMTBOMO04757	P_KWMTBOMO04757	P_KWMTBOMO04757	1
406	2	2	6.4000003	2.08	2.08	P_KWMTBOMO02354	P_KWMTBOMO02354	P_KWMTBOMO02354	1
407	2	2	3.7930001	1.293	1.293	P_KWMTBOMO01806	P_KWMTBOMO01806	P_KWMTBOMO01806	1
408	2	2	4.2739999	1.099	1.099	P_KWMTBOMO00303	P_KWMTBOMO00303	P_KWMTBOMO00303	1
409	2	2	1.454	0.3436	0.3436	P_KWMTBOMO15035	P_KWMTBOMO15035	P_KWMTBOMO15035	1
410	2	2	11.3	11.3	11.3	P_KWMTBOMO12903	P_KWMTBOMO12903	P_KWMTBOMO12903	2
411	2	2	7.2920002	4.5139998	4.5139998	P_KWMTBOMO11202	P_KWMTBOMO11202	P_KWMTBOMO11202	1
412	2	2	15.82	6.78	6.78	P_KWMTBOMO10147	P_KWMTBOMO10147	P_KWMTBOMO10147	1
413	2	2	2.9850001	2.1709999	2.1709999	P_KWMTBOMO09811	P_KWMTBOMO09811	P_KWMTBOMO09811	1
414	2	2	1.589	1.059	1.059	P_KWMTBOMO08734	P_KWMTBOMO08734	P_KWMTBOMO08734	1
415	2	2	9.1229998	5.263	5.263	P_KWMTBOMO08323	P_KWMTBOMO08323	P_KWMTBOMO08323	1
417	2	2	9.1449998	4.425	4.425	P_KWMTBOMO07278	P_KWMTBOMO07278	P_KWMTBOMO07278	1
418	2	2	9.2050001	5.858	5.858	P_KWMTBOMO05784	P_KWMTBOMO05784	P_KWMTBOMO05784	1
419	2	2	9.7560003	5.2850001	5.2850001	P_KWMTBOMO04424	P_KWMTBOMO04424	P_KWMTBOMO04424	1
421	2	2	8.202	4.101	4.101	P_KWMTBOMO03337	P_KWMTBOMO03337	P_KWMTBOMO03337	1
422	2	2	8.6669996	5.9999999	5.9999999	P_KWMTBOMO02775	P_KWMTBOMO02775	P_KWMTBOMO02775	1
423	2	2	10.91	7.2729997	7.2729997	P_KWMTBOMO01129	P_KWMTBOMO01129	P_KWMTBOMO01129	1
424	2	2	6.2150002	3.5780001	3.5780001	P_KWMTBOMO00366	P_KWMTBOMO00366	P_KWMTBOMO00366	1
425	2	2	8.5709997	8.5709997	8.5709997	P_KWMTBOMO16319	P_KWMTBOMO16319	P_KWMTBOMO16319	1
426	2	2	4.487	4.487	4.487	P_KWMTBOMO16040	P_KWMTBOMO16040	P_KWMTBOMO16040	1
427	2	2	3.743	3.743	3.743	P_KWMTBOMO15712	P_KWMTBOMO15712	P_KWMTBOMO15712	1
428	2	2	10.92	10.92	10.92	P_KWMTBOMO15520	P_KWMTBOMO15520	P_KWMTBOMO15520	1
429	2	2	3.9329998	3.9329998	3.9329998	P_KWMTBOMO15419	P_KWMTBOMO15419	P_KWMTBOMO15419	1
430	2	2	3.9999999	3.9999999	3.9999999	P_KWMTBOMO15341	P_KWMTBOMO15341	P_KWMTBOMO15341	1
431	2	2	2.874	2.874	2.874	P_KWMTBOMO15179	P_KWMTBOMO15179	P_KWMTBOMO15179	1
432	2	2	1.385	1.385	1.385	P_KWMTBOMO14962	P_KWMTBOMO14962	P_KWMTBOMO14962	1
433	2	2	4.8160002	4.8160002	4.8160002	P_KWMTBOMO14877	P_KWMTBOMO14877	P_KWMTBOMO14877	1

434	2	2	3.6249999	3.6249999	3.6249999	P_KWMTBOMO14752	P_KWMTBOMO14752	P_KWMTBOMO14752	1
435	2	2	5.4219998	5.4219998	5.4219998	P_KWMTBOMO13923	P_KWMTBOMO13923	P_KWMTBOMO13923	1
436	2	2	11.21	11.21	11.21	P_KWMTBOMO13899	P_KWMTBOMO13899	P_KWMTBOMO13899	1
437	2	2	5.263	5.263	5.263	P_KWMTBOMO13723	P_KWMTBOMO13723	P_KWMTBOMO13723	1
438	2	2	11.69	11.69	11.69	P_KWMTBOMO13530	P_KWMTBOMO13530	P_KWMTBOMO13530	1
439	2	2	4.18	4.18	4.18	P_KWMTBOMO13301	P_KWMTBOMO13301	P_KWMTBOMO13301	1
440	2	2	9.1499999	9.1499999	9.1499999	P_KWMTBOMO12682	P_KWMTBOMO12682	P_KWMTBOMO12682	1
441	2	2	4.5940001	4.5940001	4.5940001	P_KWMTBOMO11977	P_KWMTBOMO11977	P_KWMTBOMO11977	1
442	2	2	7.5580001	7.5580001	7.5580001	P_KWMTBOMO10354	P_KWMTBOMO10354	P_KWMTBOMO10354	1
443	2	2	4.8050001	4.8050001	4.8050001	P_KWMTBOMO09250	P_KWMTBOMO09250	P_KWMTBOMO09250	1
444	2	2	3.07	3.07	3.07	P_KWMTBOMO08991	P_KWMTBOMO08991	P_KWMTBOMO08991	1
445	2	2	17.389999	17.389999	17.389999	P_KWMTBOMO08193	P_KWMTBOMO08193	P_KWMTBOMO08193	1
446	2	2	1.891	1.891	1.891	P_KWMTBOMO07274	P_KWMTBOMO07274	P_KWMTBOMO07274	1
447	2	2	7.2219998	7.2219998	7.2219998	P_KWMTBOMO06558	P_KWMTBOMO06558	P_KWMTBOMO06558	1
448	2	2	10.09	10.09	10.09	P_KWMTBOMO06299	P_KWMTBOMO06299	P_KWMTBOMO06299	1
449	2	2	2.293	2.293	2.293	P_KWMTBOMO05364	P_KWMTBOMO05364	P_KWMTBOMO05364	1
450	2	2	6.3029997	6.3029997	6.3029997	P_KWMTBOMO05254	P_KWMTBOMO05254	P_KWMTBOMO05254	1
451	2	2	12.16	12.16	12.16	P_KWMTBOMO05032	P_KWMTBOMO05032	P_KWMTBOMO05032	2
452	2	2	5.0190002	5.0190002	5.0190002	P_KWMTBOMO04992	P_KWMTBOMO04992	P_KWMTBOMO04992	1
453	2	2	6.0109999	6.0109999	6.0109999	P_KWMTBOMO04584	P_KWMTBOMO04584	P_KWMTBOMO04584	1
454	2	2	5.9999999	5.9999999	5.9999999	P_KWMTBOMO04167	P_KWMTBOMO04167	P_KWMTBOMO04167	1
455	2	2	5.2809998	5.2809998	5.2809998	P_KWMTBOMO03169	P_KWMTBOMO03169	P_KWMTBOMO03169	1
456	2	2	8.5709997	8.5709997	8.5709997	P_KWMTBOMO03003	P_KWMTBOMO03003	P_KWMTBOMO03003	1
457	2	2	2.699	2.699	2.699	P_KWMTBOMO02577	P_KWMTBOMO02577	P_KWMTBOMO02577	1
458	2	2	4.58	4.58	4.58	P_KWMTBOMO02392	P_KWMTBOMO02392	P_KWMTBOMO02392	1
459	2	2	2.012	2.012	2.012	P_KWMTBOMO02096	P_KWMTBOMO02096	P_KWMTBOMO02096	1
460	2	2	4.408	4.408	4.408	P_KWMTBOMO01802	P_KWMTBOMO01802	P_KWMTBOMO01802	1
461	2	2	3.0169999	3.0169999	3.0169999	P_KWMTBOMO01708	P_KWMTBOMO01708	P_KWMTBOMO01708	1
462	2	2	8.9199997	8.9199997	8.9199997	P_KWMTBOMO01662	P_KWMTBOMO01662	P_KWMTBOMO01662	1
463	2	2	3.9999999	3.9999999	3.9999999	P_KWMTBOMO01315	P_KWMTBOMO01315	P_KWMTBOMO01315	1
464	2	2	4.222	4.222	4.222	P_KWMTBOMO00223	P_KWMTBOMO00223	P_KWMTBOMO00223	1
465	2	2	1.542	1.542	1.542	P_KWMTBOMO00106	P_KWMTBOMO00106	P_KWMTBOMO00106	1
466	1.92	1.92	7.4699998	3.1330001	3.1330001	P_KWMTBOMO08760	P_KWMTBOMO08760	P_KWMTBOMO08760	1
467	1.92	1.92	12.14	12.14	12.14	P_KWMTBOMO15875	P_KWMTBOMO15875	P_KWMTBOMO15875	1
468	1.92	1.92	9.9380001	9.9380001	9.9380001	P_KWMTBOMO07247	P_KWMTBOMO07247	P_KWMTBOMO07247	1
469	1.89	1.92	7.8210004	7.8210004	7.8210004	P_KWMTBOMO06401	P_KWMTBOMO06401	P_KWMTBOMO06401	1
470	1.89	1.89	15.14	8.1079997	4.324	P_KWMTBOMO15584	P_KWMTBOMO15584	P_KWMTBOMO15584	1
471	1.85	1.85	4.9240001	4.9240001	4.9240001	P_KWMTBOMO05651	P_KWMTBOMO05651	P_KWMTBOMO05651	1
472	1.82	1.82	1.904	1.904	1.904	P_KWMTBOMO10508	P_KWMTBOMO10508	P_KWMTBOMO10508	1
473	1.81	1.81	10.98	5.3410001	3.2639999	P_KWMTBOMO01757	P_KWMTBOMO01757	P_KWMTBOMO01757	1
474	1.78	1.78	7.4069999	2.9100001	2.9100001	P_KWMTBOMO05509	P_KWMTBOMO05509	P_KWMTBOMO05509	1
475	1.77	1.77	4.4920001	1.176	1.176	P_KWMTBOMO14420	P_KWMTBOMO14420	P_KWMTBOMO14420	1
476	1.77	1.77	5.0689999	5.0689999	5.0689999	P_KWMTBOMO13597	P_KWMTBOMO13597	P_KWMTBOMO13597	1
477	1.73	1.73	10.49	6.2139999	2.524	P_KWMTBOMO07952	P_KWMTBOMO07952	P_KWMTBOMO07952	1
478	1.72	1.72	2.2390001	2.2390001	2.2390001	P_KWMTBOMO11689	P_KWMTBOMO11689	P_KWMTBOMO11689	1
479	1.68	1.68	6.6380002	1.5869999	1.5869999	P_KWMTBOMO13567	P_KWMTBOMO13567	P_KWMTBOMO13567	1
480	1.68	1.68	1.184	1.184	1.184	P_KWMTBOMO04543	P_KWMTBOMO04543	P_KWMTBOMO04543	1
481	1.66	1.66	7.2729997	7.2729997	7.2729997	P_KWMTBOMO01799	P_KWMTBOMO01799	P_KWMTBOMO01799	1

482	1.64	1.64	1.173	0.4399	0.4399	P_KWMTBOMO13925	P_KWMTBOMO13925	P_KWMTBOMO13925	1
483	1.64	1.64	6.1700001	2.5529999	2.5529999	P_KWMTBOMO04762	P_KWMTBOMO04762	P_KWMTBOMO04762	1
484	1.62	1.62	0.8989	0.8989	0.8989	P_KWMTBOMO14080	P_KWMTBOMO14080	P_KWMTBOMO14080	1
485	1.6	1.6	3.8389999	1.8279999	1.8279999	P_KWMTBOMO07294	P_KWMTBOMO07294	P_KWMTBOMO07294	1
486	1.59	1.59	11.93	2.7519999	2.7519999	P_KWMTBOMO01300	P_KWMTBOMO01300	P_KWMTBOMO01300	1
487	1.55	1.55	3.5209998	0.922	0.922	P_KWMTBOMO01579	P_KWMTBOMO01579	P_KWMTBOMO01579	1
488	1.55	1.55	5.9599999	5.9599999	5.9599999	P_KWMTBOMO03120	P_KWMTBOMO03120	P_KWMTBOMO03120	1
489	1.54	1.54	6.9519997	6.9519997	6.9519997	P_KWMTBOMO13713	P_KWMTBOMO13713	P_KWMTBOMO13713	1
490	1.49	1.49	3.9159998	3.9159998	3.9159998	P_KWMTBOMO07617	P_KWMTBOMO07617	P_KWMTBOMO07617	1
491	1.48	1.48	15.73	15.73	15.73	P_KWMTBOMO07685	P_KWMTBOMO07685	P_KWMTBOMO07685	1
492	1.42	1.42	18.69	12.15	12.15	P_KWMTBOMO10128	P_KWMTBOMO10128	P_KWMTBOMO10128	1
493	1.41	1.41	11.11	11.11	11.11	P_KWMTBOMO14850	P_KWMTBOMO14850	P_KWMTBOMO14850	1
494	1.4	1.4	6.5839998	6.5839998	6.5839998	P_KWMTBOMO00973	P_KWMTBOMO00973	P_KWMTBOMO00973	1
495	1.39	1.81	28.650001	13.48	7.3030002	P_KWMTBOMO16126	P_KWMTBOMO16126	P_KWMTBOMO16126	1
496	1.37	1.37	0.8228	0.484	0.484	P_KWMTBOMO08552	P_KWMTBOMO08552	P_KWMTBOMO08552	1
497	1.36	1.36	20.14	20.14	11.26	P_KWMTBOMO01379	P_KWMTBOMO01379	P_KWMTBOMO01379	2
498	1.36	1.36	14.71	4.4119999	4.4119999	P_KWMTBOMO03656	P_KWMTBOMO03656	P_KWMTBOMO03656	1
499	1.35	1.35	1.518	0.308	0.308	P_KWMTBOMO06355	P_KWMTBOMO06355	P_KWMTBOMO06355	1
500	1.35	1.35	7.8469999	6.0219999	2.372	P_KWMTBOMO15629	P_KWMTBOMO15629	P_KWMTBOMO15629	1
501	1.35	1.35	9.4439998	9.4439998	9.4439998	P_KWMTBOMO06228	P_KWMTBOMO06228	P_KWMTBOMO06228	1
502	1.34	1.34	2.276	1.6690001	0.8346	P_KWMTBOMO15485	P_KWMTBOMO15485	P_KWMTBOMO15485	1
503	1.32	1.32	14.839999	11.54	6.044	P_KWMTBOMO04529	P_KWMTBOMO04529	P_KWMTBOMO04529	1
504	1.31	1.31	12.4	12.4	6.9770001	P_KWMTBOMO03173	P_KWMTBOMO03173	P_KWMTBOMO03173	1

Supplementary Table S2. Common PIPs of each fibroin gene in the M4 and L5D5 PSGs.

common PIPs of fibH

Name	Bombyx-mori	Subject ID	Description	e-value	identity(%)	coverage(%)	Metazoa	Subject ID	Description	e-value	identity(%)	coverage(%)
P_KWMTBOMO00339		tr|H9ITE6|H9ITE6_BOMMO	CSD_1 domain-containing protein OS=Bombyx mori OX=7091 PE=4 SV=1	0	100	100		sp|P41824|YBOXH_APLCA	Y-box factor homolog OS=Aplysia californica OX=6500 PE=2 SV=1	1.24E-50	69.106	47.49034749
P_KWMTBOMO00462		tr|D9N4J4|D9N4J4_BOMMO	Staphylococcal nuclease domain-containing protein OS=Bombyx mori OX=7091 GN=Tudor-SN PE=2 SV=1	0	99.887	100		sp|Q9W0S7|SND1_DROME	Staphylococcal nuclease domain-containing protein 1 OS=Drosophila melanogaster OX=7227 GN=Tudor-SN PE=1 SV=1	0	52.412	100
P_KWMTBOMO00497		tr|H9JRZ9|H9JRZ9_BOMMO	Uncharacterized protein OS=Bombyx mori OX=7091 PE=3 SV=1	0	100	100		sp|Q60HD7|SERA_MACFA	D-3-phosphoglycerate dehydrogenase OS=Macaca fascicularis OX=9541 GN=PHGDH PE=2 SV=4	2.42E-109	50.814	93.88379205
P_KWMTBOMO00638		tr|D2Y4R4|D2Y4R4_BOMMO	Coatomer subunit beta' OS=Bombyx mori OX=7091 GN=COPB2 PE=2 SV=1	0	99.877	99.26918392		sp|O62621|COPB2_DROME	Coatomer subunit beta' OS=Drosophila melanogaster OX=7227 GN=beta'COP PE=2 SV=2	0	71.059	98.90377588
P_KWMTBOMO00793		tr|H9JJJ8|H9JJJ8_BOMMO	Uncharacterized protein OS=Bombyx mori OX=7091 GN=101743807 PE=3 SV=1	0	100	100		sp|Q68FQ0|TCPE_RAT	T-complex protein 1 subunit epsilon OS=Rattus norvegicus OX=10116 GN=Cct5 PE=1 SV=1	0	70.391	99.07749077
P_KWMTBOMO00927		tr|H9JJU3|H9JJU3_BOMMO	Aspartate--tRNA ligase, cytoplasmic OS=Bombyx mori OX=7091 PE=3 SV=1	0	100	71.22040073		sp|Q3SYZ4|SYDC_BOVIN	Aspartate--tRNA ligase, cytoplasmic OS=Bos taurus OX=9913 GN=DARS1 PE=2 SV=1	0	64.329	90.89253188
P_KWMTBOMO01075		tr|H9JCV2|H9JCV2_BOMMO	Uncharacterized protein OS=Bombyx mori OX=7091 GN=101741707 PE=3 SV=1	0	99.629	100		sp|O02649|CH60A_DROME	Heat shock protein 60A OS=Drosophila melanogaster OX=7227 GN=Hsp60A PE=1 SV=3	0	84.72	95.91836735
P_KWMTBOMO01114		tr|H9JCT5|H9JCT5_BOMMO	26S proteasome regulatory subunit 7 OS=Bombyx mori OX=7091 GN=101744256 PE=3 SV=1	0	100	100		sp|P35998|PRS7_HUMAN	26S proteasome regulatory subunit 7 OS=Homo sapiens OX=9606 GN=PSMC2 PE=1 SV=3	0	91.014	100
P_KWMTBOMO01148		tr|Q2F5M4|Q2F5M4_BOMMO	Receptor for activated protein kinase C RACK isoform 1 OS=Bombyx mori OX=7091 PE=2 SV=1	0	100	100		sp|O18640|GBLP_DROME	Guanine nucleotide-binding protein subunit beta-like protein OS=Drosophila melanogaster OX=7227 GN=Rack1 PE=1 SV=2	0	86.52	100
P_KWMTBOMO01273		tr|Q5UAS5|Q5UAS5_BOMMO	60S ribosomal protein L13a OS=Bombyx mori OX=7091 GN=RpL13A PE=2 SV=1	3.08E-151	100	100		sp|Q8MUR4|RL13A_CHOPR	60S ribosomal protein L13a OS=Choristoneura parallela OX=106495 GN=RpL13A PE=2 SV=1	3.13E-140	91.667	100
P_KWMTBOMO01312		tr|Q2F5N7|Q2F5N7_BOMMO	26S proteasome regulatory subunit RPN11 OS=Bombyx mori OX=7091 PE=2 SV=1	0	100	100		sp|Q9V3H2|PSDE_DROME	26S proteasome non-ATPase regulatory subunit 14 OS=Drosophila melanogaster OX=7227 GN=Rpn11 PE=1 SV=1	0	92.926	100
P_KWMTBOMO01606		tr|H9J993|H9J993_BOMMO	Nop domain-containing protein OS=Bombyx mori OX=7091 GN=101742458 PE=3 SV=1	0	99.436	100		sp|Q9Y2X3|NOP58_HUMAN	Nucleolar protein 58 OS=Homo sapiens OX=9606 GN=NOP58 PE=1 SV=1	0	63.377	86.20037807
P_KWMTBOMO01720		tr|H9J930|H9J930_BOMMO	Uncharacterized protein OS=Bombyx mori OX=7091 PE=4 SV=1	0	98.191	100		sp|Q26457|LA_AEDAL	La protein homolog OS=Aedes albopictus OX=7160 PE=1 SV=1	2.92E-91	44.225	93.42105263
P_KWMTBOMO01918		tr|Q2F5L5|Q2F5L5_BOMMO	Protein transport protein Sec61 subunit beta OS=Bombyx mori OX=7091 GN=733070 PE=2 SV=1	1.66E-65	100	100		sp|P60467|SC61B_CANLF	Protein transport protein Sec61 subunit beta OS=Canis lupus familiaris OX=9615 GN=SEC61B PE=1 SV=2	2.67E-41	71.277	96.90721649
P_KWMTBOMO02081		tr|Q1HPW2|Q1HPW2_BOMMO	RNA helicase OS=Bombyx mori OX=7091 GN=692781 PE=2 SV=1	0	100	100		sp|Q02748|IF4A_DROME	Eukaryotic initiation factor 4A OS=Drosophila melanogaster OX=7227 GN=eIF4A PE=1 SV=3	0	79.949	93.80952381
P_KWMTBOMO02431		tr|Q1HQ25|Q1HQ25_BOMMO	ATP synthase subunit OS=Bombyx mori OX=7091 GN=732934 PE=2 SV=1	2.04E-70	100	100		sp|Q5RFH0|ATP5L_PONAB	ATP synthase subunit g, mitochondrial OS=Pongo abelii OX=9601 GN=ATP5MG PE=3 SV=1	1.02E-32	52.632	95.95959596
P_KWMTBOMO02470		tr|H9IZ85|H9IZ85_BOMMO	Dolichyl-diphosphooligosaccharide--protein glycosyltransferase subunit 1 OS=Bombyx mori OX=7091 PE=3 SV=1	0	100	98.93162393		sp|E2RQ08|RPN1_CANLF	Dolichyl-diphosphooligosaccharide--protein glycosyltransferase subunit 1 OS=Canis lupus familiaris OX=9615 GN=RPN1 PE=1 SV=1	6.16E-166	53.811	92.52136752

P_KWMTBO MO02835	tr|H9J2D4|H9J2 D4_BOMMO	RPN13_C domain-containing protein OS=Bombyx mori OX=7091 PE=3 SV=1	0	97.136	100	sp|Q6NZ09|ADR M1_DANRE	Proteasomal ubiquitin receptor ADRM1 OS=Danio rerio OX=7955 GN=adrm1b PE=1 SV=1	1.84E-92	45.524	96.06879607
P_KWMTBO MO03067	tr|H9J8B0|H9J8 B0_BOMMO	Tripeptidyl-peptidase 2 OS=Bombyx mori OX=7091 GN=101742969 PE=4 SV=1	0	100	100	sp|P29144|TPP2 _HUMAN	Tripeptidyl-peptidase 2 OS=Homo sapiens OX=9606 GN=TPP2 PE=1 SV=4	0	41.24	100
P_KWMTBO MO03177	tr|H9JV99|H9JV 99_BOMMO	BRO1 domain-containing protein OS=Bombyx mori OX=7091 PE=4 SV=1	0	97.718	100	sp|Q9QZA2|PDC 6I_RAT	Programmed cell death 6-interacting protein OS=Rattus norvegicus OX=10116 GN=Pdcd6ip PE=1 SV=2	0	43.273	98.36289222
P_KWMTBO MO03314	tr|Q5UAS3|Q5U AS3_BOMMO	Ribosomal protein L15 OS=Bombyx mori OX=7091 GN=RpL15 PE=2 SV=1	2.87E-148	100	100	sp|P30736|RL15 _CHITE	60S ribosomal protein L15 OS=Chironomus tentans OX=7153 GN=RpL15 PE=3 SV=3	3.13E-122	80.392	100
P_KWMTBO MO03626	tr|H9J9S0|H9J9S 0_BOMMO	CCT-alpha OS=Bombyx mori OX=7091 GN=101742382 PE=3 SV=1	0	100	100	sp|P12613|TCPA _DROME	T-complex protein 1 subunit alpha OS=Drosophila melanogaster OX=7227 GN=CCT1 PE=2 SV=2	0	80.576	100
P_KWMTBO MO03986	tr|Q9NL60|Q9N L60_BOMMO	Annexin OS=Bombyx mori OX=7091 PE=2 SV=1	0	99.69	100	sp|P22464|ANX B9_DROME	Annexin B9 OS=Drosophila melanogaster OX=7227 GN=AnxB9 PE=2 SV=2	9.67E-180	76.012	99.38080495
P_KWMTBO MO04370	tr|H9J6V7|H9J6 V7_BOMMO	OBG-type G domain-containing protein OS=Bombyx mori OX=7091 PE=4 SV=1	2.07E-172	99.156	64.57765668	sp|P32234|128U P_DROME	GTP-binding protein 128up OS=Drosophila melanogaster OX=7227 GN=128up PE=2 SV=2	0	89.946	100
P_KWMTBO MO04502	tr|Q5TLD3|Q5T LD3_BOMMO	Replication protein A subunit OS=Bombyx mori OX=7091 GN=BmRPA1 PE=2 SV=1	0	99.498	100	sp|Q24492|RFA1 _DROME	Replication protein A 70 kDa DNA-binding subunit OS=Drosophila melanogaster OX=7227 GN=RpA-70 PE=1 SV=1	0	47.421	100
P_KWMTBO MO04605	tr|H9J7K1|H9J7 K1_BOMMO	2-phospho-D-glycerate hydro-lyase OS=Bombyx mori OX=7091 PE=3 SV=1	0	100	100	sp|P15007|ENO_ DROME	Enolase OS=Drosophila melanogaster OX=7227 GN=Eno PE=1 SV=2	0	81.481	99.76905312
P_KWMTBO MO04610	tr|H9J723|H9J72 3_BOMMO	26S proteasome non-ATPase regulatory subunit 2 OS=Bombyx mori OX=7091 PE=3 SV=1	0	97.303	100	sp|Q5R9I6|PSM D2_PONAB	26S proteasome non-ATPase regulatory subunit 2 OS=Pongo abelii OX=9601 GN=PSMD2 PE=2 SV=1	0	55.556	98.05825243
P_KWMTBO MO04806	tr|H9JK60|H9JK 60_BOMMO	Ubiquitin-activating enzyme E1 OS=Bombyx mori OX=7091 GN=101739389 PE=3 SV=1	0	99.904	100	sp|Q5U300|UBA 1_RAT	Ubiquitin-like modifier-activating enzyme 1 OS=Rattus norvegicus OX=10116 GN=Uba1 PE=1 SV=1	0	65.034	97.8906999
P_KWMTBO MO05205	tr|H9IYX7|H9IY X7_BOMMO	5-aminoimidazole-4-carboxamide ribonucleotide formyltransferase OS=Bombyx mori OX=7091 PE=3 SV=1	0	100	98.81556684	sp|P31335|PUR9 _CHICK	Bifunctional purine biosynthesis protein ATIC OS=Gallus gallus OX=9031 GN=ATIC PE=1 SV=1	0	68.855	100
P_KWMTBO MO05541	tr|C7AQP4|C7A QP4_BOMMO	Calcium-transporting ATPase OS=Bombyx mori OX=7091 PE=2 SV=1	0	99.301	100	sp|Q7PPA5|ATC 1_ANOGA	Calcium-transporting ATPase sarcoplasmic/endoplasmic reticulum type OS=Anopheles gambiae OX=7165 GN=SERCA PE=3 SV=5	0	89.279	99.6007984
P_KWMTBO MO05564	tr|Q2F6A6|Q2F6 A6_BOMMO	Elongation factor Tu OS=Bombyx mori OX=7091 PE=2 SV=1	0	100	100	sp|P49411|EFTU _HUMAN	Elongation factor Tu, mitochondrial OS=Homo sapiens OX=9606 GN=TUFM PE=1 SV=2	0	65.672	86.4516129
P_KWMTBO MO05566	tr|Q2F5M1|Q2F5 M1_BOMMO	GTP-binding nuclear protein OS=Bombyx mori OX=7091 GN=692970 PE=2 SV=1	1.62E-162	100	100	sp|Q9VZ23|RAN _DROME	GTP-binding nuclear protein Ran OS=Drosophila melanogaster OX=7227 GN=Ran PE=1 SV=1	2.67E-147	91.429	98.5915493
P_KWMTBO MO05612	tr|H9JB77|H9JB 77_BOMMO	SAM_MT_RSMB_NOP domain-containing protein OS=Bombyx mori OX=7091 GN=101741314 PE=3 SV=1	0	99.874	100	sp|Q922K7|NOP 2_MOUSE	Probable 28S rRNA (cytosine-C(5))-methyltransferase OS=Mus musculus OX=10090 GN=Nop2 PE=1 SV=1	0	53.565	72.69279393
P_KWMTBO MO05741	tr|A1E129|A1E1 29_BOMMO	Kinesin-like protein OS=Bombyx mori OX=7091 PE=3 SV=2	0	99.17	100	sp|P17210|KINH _DROME	Kinesin heavy chain OS=Drosophila melanogaster OX=7227 GN=Khc PE=1 SV=2	0	74.871	100
P_KWMTBO MO05975	tr|H9J059|H9J05 9_BOMMO	Tr-type G domain-containing protein OS=Bombyx mori OX=7091 PE=4 SV=1	0	100	100	sp|Q5R8Q7|GTP B1_PONAB	GTP-binding protein 1 (Fragment) OS=Pongo abelii OX=9601 GN=GTPBP1 PE=2 SV=2	2.41E-105	74.641	75.72463768
P_KWMTBO MO05979	tr|Q5UAT0|Q5U AT0_BOMMO	Ribosomal protein L10 OS=Bombyx mori OX=7091 GN=RpL10 PE=2 SV=1	7.89E-166	100	100	sp|O96647|RL10 _BOMMA	60S ribosomal protein L10 OS=Bombyx mandarina OX=7092 GN=RpL10 PE=2 SV=1	2.91E-164	99.543	100
P_KWMTBO MO06229	tr|H9IX17|H9IX 17_BOMMO	Ribos_L4_asso_C domain-containing protein OS=Bombyx mori OX=7091 GN=692659 PE=3 SV=1	0	100	100	sp|P09180|RL4_ DROME	60S ribosomal protein L4 OS=Drosophila melanogaster OX=7227 GN=RpL4 PE=1 SV=2	0	68.407	88.24884793

P_KWMTBO MO06480	sp|Q5UAS1|RL1 8_BOMMO	60S ribosomal protein L18 OS=Bombyx mori OX=7091 GN=RpL18 PE=2 SV=1	1.41E-132	100	100	sp|Q5UAS1|RL1 8_BOMMO	60S ribosomal protein L18 OS=Bombyx mori OX=7091 GN=RpL18 PE=2 SV=1	9.16E-132	100	100
P_KWMTBO MO06974	tr|Q5UAQ1|Q5U AQ1_BOMMO	Ribosomal protein L36A OS=Bombyx mori OX=7091 GN=RpL36A PE=2 SV=1	2.04E-71	100	100	sp|Q9NB33|RL4 4_OCHTR	60S ribosomal protein L44 OS=Ochlerotatus triseriatus OX=7162 GN=RpL44 PE=3 SV=3	5.04E-61	85.577	100
P_KWMTBO MO07374	tr|Q0ZAL3|Q0Z AL3_BOMMO	Splicing factor proline-and glutamine-rich OS=Bombyx mori OX=7091 GN=SFPQ PE=2 SV=1	0	99.802	100	sp|Q9GRW7|NO NA_DROVI	Protein no-on-transient A OS=Drosophila virilis OX=7244 GN=nonA PE=2 SV=1	5.73E-114	61.132	52.47524752
P_KWMTBO MO07589	tr|A0A023SG69|A0A023SG69_B OMMO	Coatomer subunit beta OS=Bombyx mori OX=7091 GN=beta-COP PE=4 SV=1	0	93.705	100	sp|Q5ZIA5|COP B_CHICK	Coatomer subunit beta OS=Gallus gallus OX=9031 GN=COPB1 PE=2 SV=1	0	57.39	100
P_KWMTBO MO07688	tr|Q5UAP3|Q5U AP3_BOMMO	40S ribosomal protein S2 OS=Bombyx mori OX=7091 GN=RpS2 PE=2 SV=1	0	100	100	sp|P31009|RS2_ DROME	40S ribosomal protein S2 OS=Drosophila melanogaster OX=7227 GN=RpS2 PE=1 SV=2	4.18E-144	90.278	82.75862069
P_KWMTBO MO07855	tr|Q2F6C2|Q2F6 C2_BOMMO	CCT-beta OS=Bombyx mori OX=7091 GN=692797 PE=2 SV=1	0	100	100	sp|Q3ZBH0|TCP B_BOVIN	T-complex protein 1 subunit beta OS=Bos taurus OX=9913 GN=CCT2 PE=1 SV=3	0	72.15	99.81343284
P_KWMTBO MO07930	tr|Q1HQ48|Q1H Q48_BOMMO	Mitochondrial single-stranded DNA-binding protein OS=Bombyx mori OX=7091 PE=2 SV=1	1.39E-108	99.324	100	sp|P54622|SSBP _DROME	Single-stranded DNA-binding protein, mitochondrial OS=Drosophila melanogaster OX=7227 GN=mtSSB PE=1 SV=2	2.56E-50	64.545	74.32432432
P_KWMTBO MO08045	tr|H9IUV7|H9IU V7_BOMMO	Leucyl-tRNA synthetase OS=Bombyx mori OX=7091 PE=3 SV=1	0	99.658	99.20724802	sp|Q9P2J5|SYLC _HUMAN	Leucine--tRNA ligase, cytoplasmic OS=Homo sapiens OX=9606 GN=LARS1 PE=1 SV=2	0	65.984	99.54699887
P_KWMTBO MO08100	tr|Q5UAR8|Q5U AR8_BOMMO	60S ribosomal protein L21 OS=Bombyx mori OX=7091 GN=RpL21 PE=2 SV=1	1.42E-115	100	100	sp|P46778|RL21 _HUMAN	60S ribosomal protein L21 OS=Homo sapiens OX=9606 GN=RPL21 PE=1 SV=2	2.69E-77	66.875	100
P_KWMTBO MO08106	tr|H9IUY0|H9IU Y0_BOMMO	Clathrin light chain OS=Bombyx mori OX=7091 PE=3 SV=1	2.92E-149	98.095	100	sp|Q9VWA1|CL C_DROME	Clathrin light chain OS=Drosophila melanogaster OX=7227 GN=Clc PE=2 SV=1	5.76E-40	46.948	100
P_KWMTBO MO08310	tr|Q1HQB9|Q1H QB9_BOMMO	Nucleosome assembly protein isoform 1 OS=Bombyx mori OX=7091 PE=2 SV=1	0	100	100	sp|Q5U2Z3|NP1 L4_RAT	Nucleosome assembly protein 1-like 4 OS=Rattus norvegicus OX=10116 GN=Nap1l4 PE=1 SV=1	4.23E-97	49.462	94.17721519
P_KWMTBO MO08343	tr|H9JI86|H9JI86 _BOMMO	Uncharacterized protein OS=Bombyx mori OX=7091 PE=4 SV=1	0	100	71.51898734	sp|P54802|ANA G_HUMAN	Alpha-N-acetylglucosaminidase OS=Homo sapiens OX=9606 GN=NAGLU PE=1 SV=2	2.63E-165	41.81	67.61603376
P_KWMTBO MO08399	tr|Q5UAR0|Q5U AR0_BOMMO	60S ribosomal protein L28 OS=Bombyx mori OX=7091 GN=RpL28 PE=2 SV=1	1.61E-98	100	100	sp|Q962T2|RL28 _SPOFR	60S ribosomal protein L28 OS=Spodoptera frugiperda OX=7108 GN=RpL28 PE=2 SV=1	7.15E-81	92.857	90.64748201
P_KWMTBO MO08607	tr|H9JEL8|H9JE L8_BOMMO	Uncharacterized protein OS=Bombyx mori OX=7091 PE=4 SV=1	2.44E-142	98.974	54.31754875	sp|Q5M9F1|RB M34_RAT	RNA-binding protein 34 OS=Rattus norvegicus OX=10116 GN=Rbm34 PE=1 SV=1	8.14E-07	26.087	64.06685237
P_KWMTBO MO08642	tr|F8UN44|F8UN 44_BOMMO	Heat shock protein 70-3 OS=Bombyx mori OX=7091 PE=2 SV=1	0	100	100	sp|P29844|BIP_ DROME	Endoplasmic reticulum chaperone BiP OS=Drosophila melanogaster OX=7227 GN=Hsc70-3 PE=1 SV=2	0	89.708	99.38931298
P_KWMTBO MO08648	tr|H9JEK1|H9JE K1_BOMMO	Uncharacterized protein OS=Bombyx mori OX=7091 PE=3 SV=1	0	91.125	100	sp|P13395|SPTC A_DROME	Spectrin alpha chain OS=Drosophila melanogaster OX=7227 GN=alpha-Spec PE=1 SV=2	0	76.124	100
P_KWMTBO MO08666	tr|Q66SV4|Q66S V4_BOMMO	40S ribosomal protein S23 OS=Bombyx mori OX=7091 GN=S23 PE=2 SV=1	5.48E-103	100	100	sp|Q6EV23|RS2 3_PAPDA	40S ribosomal protein S23 OS=Papilio dardanus OX=77259 GN=RpS23 PE=2 SV=1	6.27E-101	97.902	100
P_KWMTBO MO08776	sp|Q1HPW4|EIF 3I_BOMMO	Eukaryotic translation initiation factor 3 subunit I OS=Bombyx mori OX=7091 PE=2 SV=1	0	100	100	sp|Q1HPW4|EIF 3I_BOMMO	Eukaryotic translation initiation factor 3 subunit I OS=Bombyx mori OX=7091 PE=2 SV=1	0	100	100
P_KWMTBO MO08820	tr|Q1HPI5|Q1HP I5_BOMMO	Nucleoside diphosphate kinase OS=Bombyx mori OX=7091 PE=2 SV=1	4.74E-113	100	100	sp|P08879|NDK A_DROME	Nucleoside diphosphate kinase OS=Drosophila melanogaster OX=7227 GN=awd PE=1 SV=3	5.17E-94	81.046	100
P_KWMTBO MO08956	tr|H9JDY1|H9JD Y1_BOMMO	M20_dimer domain-containing protein OS=Bombyx mori OX=7091 PE=3 SV=1	0	99.784	96.86847599	sp|Q3ZC84|CND P2_BOVIN	Cytosolic non-specific dipeptidase OS=Bos taurus OX=9913 GN=CNDP2 PE=2 SV=1	0	58.65	98.95615866

P_KWMTBOMO09050	tr|H9JDA0|H9JDA0_BOMMO	Uncharacterized protein OS=Bombyx mori OX=7091 PE=4 SV=1	0	100	92.79661017	sp|Q96RQ3|MCCA_HUMAN	Methylcrotonoyl-CoA carboxylase subunit alpha, mitochondrial OS=Homo sapiens OX=9606 GN=MCCC1 PE=1 SV=3	0	56.278	95.62146893
P_KWMTBOMO09054	tr|Q1HQ47|Q1HQ47_BOMMO	Isocitrate dehydrogenase [NADP] OS=Bombyx mori OX=7091 GN=100101164 PE=2 SV=1	0	100	100	sp|P33198|IDHP_PIG	Isocitrate dehydrogenase [NADP], mitochondrial (Fragment) OS=Sus scrofa OX=9823 GN=IDH2 PE=1 SV=1	0	71.325	95.40229885
P_KWMTBOMO09230	tr|H9J1M5|H9J1M5_BOMMO	Uncharacterized protein OS=Bombyx mori OX=7091 PE=3 SV=1	3.45E-75	100	100	sp|P08570|RLA1_DROME	60S acidic ribosomal protein P1 OS=Drosophila melanogaster OX=7227 GN=RpLP1 PE=1 SV=2	1.01E-42	81.25	100
P_KWMTBOMO09415	tr|H9JTB0|H9JTB0_BOMMO	RNA helicase OS=Bombyx mori OX=7091 GN=101741115 PE=4 SV=1	0	99.765	100	sp|Q27268|DX39B_DROME	ATP-dependent RNA helicase WM6 OS=Drosophila melanogaster OX=7227 GN=Hel25E PE=1 SV=1	0	88.498	100
P_KWMTBOMO09442	tr|Q5UAT5|Q5UAT5_BOMMO	60S ribosomal protein L6 OS=Bombyx mori OX=7091 GN=RpL6 PE=2 SV=1	0	99.634	100	sp|Q6QMZ4|RL6_CHILA	60S ribosomal protein L6 OS=Chinchilla lanigera OX=34839 GN=RPL6 PE=2 SV=3	4.24E-65	48.8	91.57509158
P_KWMTBOMO09542	tr|H9JU13|H9JU13_BOMMO	Eukaryotic translation initiation factor 3 subunit A OS=Bombyx mori OX=7091 PE=3 SV=1	0	96.154	90.33898305	sp|Q173M7|EIF3A_AEDAE	Eukaryotic translation initiation factor 3 subunit A OS=Aedes aegypti OX=7159 GN=eIF3-S10 PE=3 SV=1	0	66.215	68.72881356
P_KWMTBOMO09621	tr|H9JTI9|H9JTI9_BOMMO	Eukaryotic translation initiation factor 3 subunit C OS=Bombyx mori OX=7091 GN=eIF3c PE=3 SV=1	0	100	100	sp|Q0ZB76|EIF3C_BOMMO	Eukaryotic translation initiation factor 3 subunit C OS=Bombyx mori OX=7091 GN=eIF3-S8 PE=2 SV=1	0	99.886	100
P_KWMTBOMO09747	tr|B7XFU6|B7XFU6_BOMMO	Clathrin heavy chain OS=Bombyx mori OX=7091 GN=100233163 PE=2 SV=1	0	99.941	100	sp|P29742|CLH_DROME	Clathrin heavy chain OS=Drosophila melanogaster OX=7227 GN=Chc PE=1 SV=1	0	85.196	100
P_KWMTBOMO09808	tr|Q2F6C3|Q2F6C3_BOMMO	Chaperonin subunit 6a zeta OS=Bombyx mori OX=7091 GN=692796 PE=2 SV=1	0	100	100	sp|P80317|TCPZ_MOUSE	T-complex protein 1 subunit zeta OS=Mus musculus OX=10090 GN=Cct6a PE=1 SV=3	0	72.505	100
P_KWMTBOMO10056	tr|B5M9A0|B5M9A0_BOMMO	Myosin heavy chain (Fragment) OS=Bombyx mori OX=7091 PE=2 SV=1	0	55.179	26.95431472	sp|Q99323|MYSN_DROME	Myosin heavy chain, non-muscle OS=Drosophila melanogaster OX=7227 GN=zip PE=1 SV=2	0	74.987	100
P_KWMTBOMO10248	tr|H9JCR4|H9JCR4_BOMMO	40S ribosomal protein SA OS=Bombyx mori OX=7091 PE=3 SV=1	0	100	100	sp|Q5UAP4|RSSA_BOMMO	40S ribosomal protein SA OS=Bombyx mori OX=7091 PE=2 SV=1	0	99.673	100
P_KWMTBOMO10283	tr|H9JCM2|H9JCM2_BOMMO	PCI domain-containing protein OS=Bombyx mori OX=7091 GN=692900 PE=3 SV=1	0	100	100	sp|Q9D8W5|PSD12_MOUSE	26S proteasome non-ATPase regulatory subunit 12 OS=Mus musculus OX=10090 GN=Psmd12 PE=1 SV=4	0	59.071	100
P_KWMTBOMO10358	tr|H9JX26|H9JX26_BOMMO	DUF4781 domain-containing protein OS=Bombyx mori OX=7091 PE=4 SV=1	0	79.827	48.32869081	--	--	--	--	--
P_KWMTBOMO10780	tr|H9JFX9|H9JFX9_BOMMO	Uncharacterized protein OS=Bombyx mori OX=7091 PE=4 SV=1	0	90.138	43.81909548	sp|Q9V3P6|PSMD1_DROME	26S proteasome non-ATPase regulatory subunit 1 OS=Drosophila melanogaster OX=7227 GN=Rpn2 PE=1 SV=1	0	71.218	100
P_KWMTBOMO11061	tr|Q2F5N1|Q2F5N1_BOMMO	Prohibitin OS=Bombyx mori OX=7091 GN=693108 PE=2 SV=1	0	100	100	sp|Q5XIH7|PHB2_RAT	Prohibitin-2 OS=Rattus norvegicus OX=10116 GN=Phb2 PE=1 SV=1	5.66E-158	72.667	100
P_KWMTBOMO11083	tr|Q9BPS3|Q9BPS3_BOMMO	Elongation factor 1 gamma OS=Bombyx mori OX=7091 GN=ef-1g PE=2 SV=1	0	100	100	sp|P12261|EF1G_ARTSA	Elongation factor 1-gamma OS=Artemia salina OX=85549 PE=1 SV=3	0	65.741	100
P_KWMTBOMO11096	tr|A1E9B3|A1E9B3_BOMMO	H(+)-transporting two-sector ATPase OS=Bombyx mori OX=7091 PE=2 SV=1	0	99.676	100	sp|P31400|VATA_MANSE	V-type proton ATPase catalytic subunit A OS=Manduca sexta OX=7130 GN=VHAA PE=2 SV=1	0	98.217	100
P_KWMTBOMO11255	tr|Q5UAN5|Q5UAN5_BOMMO	40S ribosomal protein S9 OS=Bombyx mori OX=7091 GN=RpS9 PE=2 SV=1	5.62E-140	100	100	sp|B4PEU8|RS9_DROYA	40S ribosomal protein S9 OS=Drosophila yakuba OX=7245 GN=RpS9 PE=2 SV=1	2.83E-117	89.231	100
P_KWMTBOMO11336	tr|H9IX66|H9IX66_BOMMO	Threonyl-tRNA synthetase OS=Bombyx mori OX=7091 PE=3 SV=1	0	98.151	98.59747546	sp|Q3ZBV8|SYTC_BOVIN	Threonine--tRNA ligase 1, cytoplasmic OS=Bos taurus OX=9913 GN=TARS1 PE=2 SV=1	0	69.371	100
P_KWMTBOMO11448	tr|H9IXE5|H9IXE5_BOMMO	Uncharacterized protein OS=Bombyx mori OX=7091 GN=733057 PE=4 SV=1	1.36E-160	100	100	sp|P53994|RAB2A_MOUSE	Ras-related protein Rab-2A OS=Mus musculus OX=10090 GN=Rab2a PE=1 SV=1	2.20E-141	90.654	100

P_KWMTBOMO11985	tr|Q5UAT4|Q5UAT4_BOMMO	Ribosomal protein L7 OS=Bombyx mori OX=7091 GN=RpL7 PE=2 SV=1	0	100	100	sp|P32100|RL7_DROME	60S ribosomal protein L7 OS=Drosophila melanogaster OX=7227 GN=RpL7 PE=1 SV=2	4.63E-117	66.667	92.56505576
P_KWMTBOMO12166	tr|H9JWZ4|H9JWZ4_BOMMO	NADH dehydrogenase [ubiquinone] 1 alpha subcomplex subunit 10, mitochondrial OS=Bombyx mori OX=7091 GN=101745480 PE=3 SV=1	0	100	100	sp|P91929|NDUAA_DROME	NADH dehydrogenase [ubiquinone] 1 alpha subcomplex subunit 10, mitochondrial OS=Drosophila melanogaster OX=7227 GN=ND-42 PE=1 SV=2	1.80E-134	49.485	97.48743719
P_KWMTBOMO12287	tr|Q5UAM2|Q5UAM2_BOMMO	40S ribosomal protein S20 OS=Bombyx mori OX=7091 GN=RpS20 PE=2 SV=1	3.74E-87	100	100	sp|P55828|RS20_DROME	40S ribosomal protein S20 OS=Drosophila melanogaster OX=7227 GN=RpS20 PE=1 SV=1	1.92E-68	83.74	100
P_KWMTBOMO12389	tr|H9JSC6|H9JSC6_BOMMO	Coatomer subunit epsilon OS=Bombyx mori OX=7091 PE=3 SV=1	0	100	100	sp|Q5ZIK9|COPE_CHICK	Coatomer subunit epsilon OS=Gallus gallus OX=9031 GN=COPE PE=2 SV=1	6.42E-96	48.475	97.68211921
P_KWMTBOMO12560	tr|A0A1Q1NKL1|A0A1Q1NKL1_BOMMO	Tyrosine--tRNA ligase OS=Bombyx mori OX=7091 PE=2 SV=1	0	99.809	100	sp|Q5ZJ08|SYYC_CHICK	Tyrosine--tRNA ligase, cytoplasmic OS=Gallus gallus OX=9031 GN=YARS1 PE=2 SV=1	0	71.374	100
P_KWMTBOMO12659	tr|H9IVX5|H9IVX5_BOMMO	T-complex protein 1 subunit gamma OS=Bombyx mori OX=7091 GN=101744569 PE=3 SV=1	0	100	100	sp|P48605|TCPG_DROME	T-complex protein 1 subunit gamma OS=Drosophila melanogaster OX=7227 GN=CCT3 PE=2 SV=2	0	81.041	99.62962963
P_KWMTBOMO12667	tr|Q1HPM7|Q1HPM7_BOMMO	Mitochondrial aldehyde dehydrogenase OS=Bombyx mori OX=7091 PE=2 SV=1	0	97.291	47.22814499	sp|P81178|ALDH2_MESAU	Aldehyde dehydrogenase, mitochondrial OS=Mesocricetus auratus OX=10036 GN=ALDH2 PE=1 SV=1	0	70	45.84221748
P_KWMTBOMO12844	tr|H9U396|H9U396_BOMMO	Endoplasmin-like protein OS=Bombyx mori OX=7091 PE=2 SV=1	0	100	100	sp|Q66HD0|ENPL_RAT	Endoplasmin OS=Rattus norvegicus OX=10116 GN=Hsp90b1 PE=1 SV=2	0	59.264	93.95061728
P_KWMTBOMO12895	tr|Q19AA9|Q19AA9_BOMMO	Transport protein Sec61 alpha subunit OS=Bombyx mori OX=7091 GN=733068 PE=2 SV=1	0	100	100	sp|Q9JLR1|S61A2_MOUSE	Protein transport protein Sec61 subunit alpha isoform 2 OS=Mus musculus OX=10090 GN=Sec61a2 PE=2 SV=3	0	91.597	100
P_KWMTBOMO13226	tr|H6VTQ9|H6VTQ9_BOMMO	DnaJ-19 OS=Bombyx mori OX=7091 PE=2 SV=1	0	100	99.29775281	sp|Q9UGP8|SEC63_HUMAN	Translocation protein SEC63 homolog OS=Homo sapiens OX=9606 GN=SEC63 PE=1 SV=2	0	44.537	100
P_KWMTBOMO13293	tr|E9JEI6|E9JEI6_BOMMO	Poly [ADP-ribose] polymerase (Fragment) OS=Bombyx mori OX=7091 PE=2 SV=1	0	99.894	94.5	sp|Q11208|PARP_SARPE	Poly [ADP-ribose] polymerase OS=Sarcophaga peregrina OX=7386 PE=1 SV=1	0	48.473	100
P_KWMTBOMO13405	tr|H9JN03|H9JN03_BOMMO	Proliferating cell nuclear antigen OS=Bombyx mori OX=7091 GN=692356 PE=3 SV=1	0	100	100	sp|O01377|PCNA_BOMMO	Proliferating cell nuclear antigen OS=Bombyx mori OX=7091 GN=PCNA PE=2 SV=1	0	98.846	100
P_KWMTBOMO13455	sp|Q5UAP0|RS4_BOMMO	40S ribosomal protein S4 OS=Bombyx mori OX=7091 GN=RpS4 PE=2 SV=1	0	100	100	sp|Q5UAP0|RS4_BOMMO	40S ribosomal protein S4 OS=Bombyx mori OX=7091 GN=RpS4 PE=2 SV=1	0	100	100
P_KWMTBOMO13569	tr|Q5UAR3|Q5UAR3_BOMMO	Ribosomal protein L26 OS=Bombyx mori OX=7091 GN=RpL26 PE=2 SV=1	7.52E-72	100	100	sp|Q95WA0|RL26_LITLI	60S ribosomal protein L26 OS=Littorina littorea OX=31216 GN=RPL26 PE=2 SV=1	1.90E-51	80.392	100
P_KWMTBOMO13859	tr|H9JPJ1|H9JPJ1_BOMMO	40S ribosomal protein S30 OS=Bombyx mori OX=7091 PE=3 SV=1	5.15E-93	100	100	sp|Q9W6Y0|RS30_ORYLA	40S ribosomal protein S30 OS=Oryzias latipes OX=8090 GN=fau PE=3 SV=2	1.58E-23	72.881	45.38461538
P_KWMTBOMO13960	tr|Q5UAN0|Q5UAN0_BOMMO	40S ribosomal protein S13 OS=Bombyx mori OX=7091 GN=RpS13 PE=2 SV=1	8.93E-110	100	100	sp|Q962R6|RS13_SPOFR	40S ribosomal protein S13 OS=Spodoptera frugiperda OX=7108 GN=RpS13 PE=2 SV=3	1.63E-108	99.338	100
P_KWMTBOMO14440	tr|Q1HPP5|Q1HPP5_BOMMO	Actin-depolymerizing factor 1 OS=Bombyx mori OX=7091 PE=2 SV=1	1.70E-108	100	100	sp|P45594|CADF_DROME	Cofilin/actin-depolymerizing factor homolog OS=Drosophila melanogaster OX=7227 GN=tsr PE=1 SV=1	2.22E-100	92.568	100
P_KWMTBOMO14479	tr|H9IS48|H9IS48_BOMMO	Uncharacterized protein OS=Bombyx mori OX=7091 GN=101742289 PE=3 SV=1	7.91E-100	100	100	sp|Q8I1F4|FBRL_DROER	rRNA 2'-O-methyltransferase fibrillarin OS=Drosophila erecta OX=7220 GN=Fib PE=3 SV=1	2.11E-41	82.278	50
P_KWMTBOMO14549	tr|I6XKQ0|I6XKQ0_BOMMO	Heat shock protein 70-5 OS=Bombyx mori OX=7091 PE=2 SV=1	0	99.565	100	sp|P29845|HSP7E_DROME	Heat shock 70 kDa protein cognate 5 OS=Drosophila melanogaster OX=7227 GN=Hsc70-5 PE=1 SV=2	0	81.86	93.47826087
P_KWMTBOMO14732	tr|Q86PG2|Q86PG2_BOMMO	ADP/ATP translocase OS=Bombyx mori OX=7091 GN=ANT PE=2 SV=1	0	100	100	sp|Q26365|ADT_DROME	ADP,ATP carrier protein OS=Drosophila melanogaster OX=7227 GN=sesB PE=2 SV=4	0	87.458	98.33333333

P_KWMTBOMO14804		tr|H9JRQ1|H9JRQ1_BOMMO	Programmed cell death protein 4 OS=Bombyx mori OX=7091 GN=101743674 PE=3 SV=1	0	100	59.14221219		sp|Q98TX3|PDCD4_CHICK	Programmed cell death protein 4 OS=Gallus gallus OX=9031 GN=PDCD4 PE=2 SV=1	3.96E-92	43.371	100
P_KWMTBOMO14936		tr|H9IU37|H9IU37_BOMMO	GB1/RHD3-type G domain-containing protein OS=Bombyx mori OX=7091 PE=3 SV=1	0	97.695	100		sp|Q9VC57|ATLAS_DROME	Atlastin OS=Drosophila melanogaster OX=7227 GN=atl PE=1 SV=1	0	68.275	97.82214156
P_KWMTBOMO14984		tr|H9J5J2|H9J5J2_BOMMO	AAA domain-containing protein OS=Bombyx mori OX=7091 PE=3 SV=1	0	100	100		sp|O42587|PR6AA_XENLA	26S proteasome regulatory subunit 6A-A OS=Xenopus laevis OX=8355 GN=psmc3-a PE=2 SV=2	0	88.124	98.36448598
P_KWMTBOMO15114		tr|Q5UAL8|Q5UAL8_BOMMO	40S ribosomal protein S25 OS=Bombyx mori OX=7091 GN=RpS25 PE=2 SV=1	7.13E-83	100	100		sp|Q962Q5|RS25_SPOFR	40S ribosomal protein S25 OS=Spodoptera frugiperda OX=7108 GN=RpS25 PE=3 SV=1	1.98E-81	99.16	100
P_KWMTBOMO15123		tr|H9J5W8|H9J5W8_BOMMO	AAA domain-containing protein OS=Bombyx mori OX=7091 PE=3 SV=2	0	99.241	90.38901602		sp|P48601|PRS4_DROME	26S proteasome regulatory subunit 4 OS=Drosophila melanogaster OX=7227 GN=Rpt2 PE=1 SV=2	0	96.811	100
P_KWMTBOMO15159		tr|H9J5Z1|H9J5Z1_BOMMO	Uncharacterized protein OS=Bombyx mori OX=7091 PE=4 SV=1	0	97.685	99.34282585		sp|Q99PL5|RRBP1_MOUSE	Ribosome-binding protein 1 OS=Mus musculus OX=10090 GN=Rrbp1 PE=1 SV=2	3.36E-17	27.524	67.25082147
P_KWMTBOMO15359		tr|H9J6H4|H9J6H4_BOMMO	Glutamyl-tRNA synthetase OS=Bombyx mori OX=7091 PE=3 SV=1	0	100	97.09944751		sp|P28668|SYEP_DROME	Bifunctional glutamate/proline--tRNA ligase OS=Drosophila melanogaster OX=7227 GN=GluProRS PE=1 SV=2	0	60.526	99.72375691
P_KWMTBOMO15584		tr|D2Y4R2|D2Y4R2_BOMMO	Coatomer subunit alpha OS=Bombyx mori OX=7091 GN=COPA PE=2 SV=1	0	99.729	99.72972973		sp|P53621|COPA_HUMAN	Coatomer subunit alpha OS=Homo sapiens OX=9606 GN=COPA PE=1 SV=2	0	78.729	97.83783784
P_KWMTBOMO15758		tr|B3VBE3|B3VBE3_BOMMO	Vacuolar proton pump subunit B OS=Bombyx mori OX=7091 PE=2 SV=1	0	99.278	88.21656051		sp|P31401|VATB_MANSE	V-type proton ATPase subunit B OS=Manduca sexta OX=7130 GN=VHA55 PE=2 SV=1	0	97.112	88.21656051
P_KWMTBOMO16309		tr|H9JW60|H9JW60_BOMMO	UBX domain-containing protein OS=Bombyx mori OX=7091 PE=4 SV=1	1.01E-106	92.353	43.58974359		sp|Q3SZC4|NSF1C_BOVIN	NSFL1 cofactor p47 OS=Bos taurus OX=9913 GN=NSFL1C PE=2 SV=1	6.48E-85	40.852	100
P_KWMTBOMO16357		tr|Q5UAN3|Q5UAN3_BOMMO	40S ribosomal protein S11 OS=Bombyx mori OX=7091 GN=RpS11-1 PE=2 SV=1	6.00E-114	100	100		sp|P62280|RS11_HUMAN	40S ribosomal protein S11 OS=Homo sapiens OX=9606 GN=RPS11 PE=1 SV=3	4.63E-88	77.848	100
P_KWMTBOMO16637		tr|Q9GSB6|Q9GSB6_BOMMO	Heat shock protein hsp20.4 OS=Bombyx mori OX=7091 GN=Hsp20.4 PE=2 SV=2	5.98E-133	100	100		sp|P82147|L2EFL_DROME	Protein lethal(2)essential for life OS=Drosophila melanogaster OX=7227 GN=l(2)efl PE=1 SV=1	8.44E-55	50.568	97.23756906

common PIPs of fibL

Name	Bombyx-mori	Subject ID	Description	e-value	identity(%)	coverage(%)	Metazoa	Subject ID	Description	e-value	identity(%)	coverage(%)
P_KWMTBOMO00303		tr|H9ITG0|H9ITG0_BOMMO	Rab-GAP TBC domain-containing protein OS=Bombyx mori OX=7091 PE=4 SV=1	0	98.655	99.87789988		sp|Q9VYY9|EVI5_DROME	Ecotropic viral integration site 5 ortholog OS=Drosophila melanogaster OX=7227 GN=Evi5 PE=1 SV=3	0	59.115	93.77289377
P_KWMTBOMO00366		tr|H9ITD6|H9ITD6_BOMMO	Uncharacterized protein OS=Bombyx mori OX=7091 PE=4 SV=1	0	99.775	83.80414313		sp|O42254|IF2B1_CHICK	Insulin-like growth factor 2 mRNA-binding protein 1 OS=Gallus gallus OX=9031 GN=IGF2BP1 PE=1 SV=1	7.53E-128	44.578	93.78531073
P_KWMTBOMO00427		tr|Q9NKX3|Q9NKX3_BOMMO	Coatomer subunit gamma OS=Bombyx mori OX=7091 GN=copg1 PE=2 SV=1	0	99.884	100		sp|Q8I0G5|COPG_DROME	Coatomer subunit gamma OS=Drosophila melanogaster OX=7227 GN=gammaCOP PE=2 SV=1	0	57.555	100
P_KWMTBOMO00462		tr|D9N4J4|D9N4J4_BOMMO	Staphylococcal nuclease domain-containing protein OS=Bombyx mori OX=7091 GN=Tudor-SN PE=2 SV=1	0	99.887	100		sp|Q9W0S7|SND1_DROME	Staphylococcal nuclease domain-containing protein 1 OS=Drosophila melanogaster OX=7227 GN=Tudor-SN PE=1 SV=1	0	52.412	100
P_KWMTBOMO00786		tr|H9JJK3|H9JJK3_BOMMO	HIT domain-containing protein OS=Bombyx mori OX=7091 GN=693069 PE=4 SV=1	6.16E-93	100	100		sp|P62958|HINT1_BOVIN	Histidine triad nucleotide-binding protein 1 OS=Bos taurus OX=9913 GN=HINT1 PE=1 SV=2	1.81E-62	68.75	100
P_KWMTBOMO00793		tr|H9JJJ8|H9JJJ8_BOMMO	Uncharacterized protein OS=Bombyx mori OX=7091 GN=101743807 PE=3 SV=1	0	100	100		sp|Q68FQ0|TCPE_RAT	T-complex protein 1 subunit epsilon OS=Rattus norvegicus OX=10116 GN=Cct5 PE=1 SV=1	0	70.391	99.07749077

P_KWMTBOMO00794	tr|Q1HQ81|Q1HQ81_BOMMO	Nascent polypeptide associated complex protein alpha subunit OS=Bombyx mori OX=7091 PE=2 SV=1	1.91E-153	100	100	sp|Q94518|NACA_DROME	Nascent polypeptide-associated complex subunit alpha OS=Drosophila melanogaster OX=7227 GN=Nacalpha PE=1 SV=2	1.16E-78	65.455	100
P_KWMTBOMO00838	tr|Q5UAR6|Q5UAR6_BOMMO	60S ribosomal protein L23 OS=Bombyx mori OX=7091 GN=RpL23 PE=2 SV=1	5.38E-99	100	100	sp|P48159|RL23_DROME	60S ribosomal protein L23 OS=Drosophila melanogaster OX=7227 GN=RpL23 PE=1 SV=2	5.37E-94	95	100
P_KWMTBOMO00860	tr|D2Y4R7|D2Y4R7_BOMMO	Coatomer subunit zeta OS=Bombyx mori OX=7091 GN=COPZ PE=2 SV=1	1.18E-132	100	100	sp|P35604|COPZ1_BOVIN	Coatomer subunit zeta-1 OS=Bos taurus OX=9913 GN=COPZ1 PE=1 SV=2	2.88E-83	62.921	100
P_KWMTBOMO00927	tr|H9JJU3|H9JJU3_BOMMO	Aspartate--tRNA ligase, cytoplasmic OS=Bombyx mori OX=7091 PE=3 SV=1	0	100	71.22040073	sp|Q3SYZ4|SYDC_BOVIN	Aspartate--tRNA ligase, cytoplasmic OS=Bos taurus OX=9913 GN=DARS1 PE=2 SV=1	0	64.329	90.89253188
P_KWMTBOMO01055	tr|Q5UAL7|Q5UAL7_BOMMO	40S ribosomal protein S26 OS=Bombyx mori OX=7091 GN=RpS26 PE=2 SV=1	1.02E-81	100	87.12121212	sp|Q9GT45|RS26_ANOGA	40S ribosomal protein S26 OS=Anopheles gambiae OX=7165 GN=RpS26 PE=3 SV=2	5.07E-67	84.348	87.12121212
P_KWMTBOMO01068	tr|H9JD15|H9JD15_BOMMO	60S ribosomal protein L34 OS=Bombyx mori OX=7091 PE=3 SV=1	1.19E-81	100	100	sp|P45842|RL34_AEDAL	60S ribosomal protein L34 OS=Aedes albopictus OX=7160 GN=RpL34 PE=2 SV=2	1.72E-56	74.074	90.75630252
P_KWMTBOMO01072	tr|H9JD17|H9JD17_BOMMO	Uncharacterized protein OS=Bombyx mori OX=7091 PE=4 SV=1	0	99.688	40.02493766	sp|Q9W3N6|USO1_DROME	General vesicular transport factor p115 OS=Drosophila melanogaster OX=7227 GN=p115 PE=1 SV=2	0	50.619	100
P_KWMTBOMO01075	tr|H9JCV2|H9JCV2_BOMMO	Uncharacterized protein OS=Bombyx mori OX=7091 GN=101741707 PE=3 SV=1	0	99.629	100	sp|O02649|CH60A_DROME	Heat shock protein 60A OS=Drosophila melanogaster OX=7227 GN=Hsp60A PE=1 SV=3	0	84.72	95.91836735
P_KWMTBOMO01114	tr|H9JCT5|H9JCT5_BOMMO	26S proteasome regulatory subunit 7 OS=Bombyx mori OX=7091 GN=101744256 PE=3 SV=1	0	100	100	sp|P35998|PRS7_HUMAN	26S proteasome regulatory subunit 7 OS=Homo sapiens OX=9606 GN=PSMC2 PE=1 SV=3	0	91.014	100
P_KWMTBOMO01148	tr|Q2F5M4|Q2F5M4_BOMMO	Receptor for activated protein kinase C RACK isoform 1 OS=Bombyx mori OX=7091 PE=2 SV=1	0	100	100	sp|O18640|GBLP_DROME	Guanine nucleotide-binding protein subunit beta-like protein OS=Drosophila melanogaster OX=7227 GN=Rack1 PE=1 SV=2	0	86.52	100
P_KWMTBOMO01273	tr|Q5UAS5|Q5UAS5_BOMMO	60S ribosomal protein L13a OS=Bombyx mori OX=7091 GN=RpL13A PE=2 SV=1	3.08E-151	100	100	sp|Q8MUR4|RL13A_CHOPR	60S ribosomal protein L13a OS=Choristoneura parallela OX=106495 GN=RpL13A PE=2 SV=1	3.13E-140	91.667	100
P_KWMTBOMO01312	tr|Q2F5N7|Q2F5N7_BOMMO	26S proteasome regulatory subunit RPN11 OS=Bombyx mori OX=7091 PE=2 SV=1	0	100	100	sp|Q9V3H2|PSDE_DROME	26S proteasome non-ATPase regulatory subunit 14 OS=Drosophila melanogaster OX=7227 GN=Rpn11 PE=1 SV=1	0	92.926	100
P_KWMTBOMO01315	tr|Q1HPJ9|Q1HPJ9_BOMMO	V-type proton ATPase subunit H OS=Bombyx mori OX=7091 PE=2 SV=1	0	99.789	100	sp|Q9U5N0|VATH_MANSE	V-type proton ATPase subunit H OS=Manduca sexta OX=7130 PE=2 SV=1	0	96.421	100
P_KWMTBOMO01424	tr|H9JHC4|H9JHC4_BOMMO	Elongation factor 1-beta' OS=Bombyx mori OX=7091 PE=3 SV=1	5.51E-165	100	100	sp|P29522|EF1B2_BOMMO	Elongation factor 1-beta' OS=Bombyx mori OX=7091 PE=1 SV=2	3.22E-163	99.55	100
P_KWMTBOMO01606	tr|H9J993|H9J993_BOMMO	Nop domain-containing protein OS=Bombyx mori OX=7091 GN=101742458 PE=3 SV=1	0	99.436	100	sp|Q9Y2X3|NOP58_HUMAN	Nucleolar protein 58 OS=Homo sapiens OX=9606 GN=NOP58 PE=1 SV=1	0	63.377	86.20037807
P_KWMTBOMO01720	tr|H9J930|H9J930_BOMMO	Uncharacterized protein OS=Bombyx mori OX=7091 PE=4 SV=1	0	98.191	100	sp|Q26457|LA_AEDAL	La protein homolog OS=Aedes albopictus OX=7160 PE=1 SV=1	2.92E-91	44.225	93.42105263
P_KWMTBOMO01790	tr|H9J907|H9J907_BOMMO	TPR_REGION domain-containing protein OS=Bombyx mori OX=7091 PE=4 SV=1	0	99.758	78.05108798	sp|Q9VGU5|TTC14_DROME	Tetratricopeptide repeat protein 14 homolog OS=Drosophila melanogaster OX=7227 GN=CG6621 PE=1 SV=2	1.20E-82	48.657	31.69347209
P_KWMTBOMO01796	tr|Q2F5J2|Q2F5J2_BOMMO	Prohibitin OS=Bombyx mori OX=7091 GN=692987 PE=2 SV=1	0	100	100	sp|P24156|L2CC_DROME	Protein l(2)37Cc OS=Drosophila melanogaster OX=7227 GN=l(2)37Cc PE=2 SV=2	6.28E-169	83.088	99.27007299
P_KWMTBOMO01817	tr|H9IVQ5|H9IVQ5_BOMMO	RNA helicase OS=Bombyx mori OX=7091 PE=3 SV=1	0	100	50.33200531	sp|Q501J6|DDX17_MOUSE	Probable ATP-dependent RNA helicase DDX17 OS=Mus musculus OX=10090 GN=Ddx17 PE=1 SV=1	0	61.331	73.83798141
P_KWMTBOMO02081	tr|Q1HPW2|Q1HPW2_BOMMO	RNA helicase OS=Bombyx mori OX=7091 GN=692781 PE=2 SV=1	0	100	100	sp|Q02748|IF4A_DROME	Eukaryotic initiation factor 4A OS=Drosophila melanogaster OX=7227 GN=eIF4A PE=1 SV=3	0	79.949	93.80952381

P_KWMTBO MO02185	tr|Q2F5X2|Q2F5 X2_BOMMO	Proteasome subunit alpha type OS=Bombyx mori OX=7091 GN=692896 PE=2 SV=1	0	100	100	sp|P22769|PSA7 1_DROME	Proteasome subunit alpha type-7-1 OS=Drosophila melanogaster OX=7227 GN=Prosalpha4 PE=1 SV=2	2.50E-145	81.818	92.77108434
P_KWMTBO MO02432	tr|H9JVD5|H9JV D5_BOMMO	Dolichyl-diphosphooligosaccharide--protein glycosyltransferase subunit 2 OS=Bombyx mori OX=7091 PE=3 SV=1	0	100	100	sp|Q3SZI6|RPN2 _BOVIN	Dolichyl-diphosphooligosaccharide--protein glycosyltransferase subunit 2 OS=Bos taurus OX=9913 GN=RPN2 PE=2 SV=1	1.44E-58	28.155	99.35691318
P_KWMTBO MO02460	tr|H9IZ80|H9IZ8 0_BOMMO	Uncharacterized protein OS=Bombyx mori OX=7091 GN=101746215 PE=4 SV=1	0	100	100	--	--	--	--	--
P_KWMTBO MO02470	tr|H9IZ85|H9IZ8 5_BOMMO	Dolichyl-diphosphooligosaccharide--protein glycosyltransferase subunit 1 OS=Bombyx mori OX=7091 PE=3 SV=1	0	100	98.93162393	sp|E2RQ08|RPN 1_CANLF	Dolichyl-diphosphooligosaccharide--protein glycosyltransferase subunit 1 OS=Canis lupus familiaris OX=9615 GN=RPN1 PE=1 SV=1	6.16E-166	53.811	92.52136752
P_KWMTBO MO02477	tr|Q5UAR9|Q5U AR9_BOMMO	Ribosomal protein L19 OS=Bombyx mori OX=7091 GN=RpL19 PE=2 SV=1	1.82E-145	100	100	sp|P36241|RL19 _DROME	60S ribosomal protein L19 OS=Drosophila melanogaster OX=7227 GN=RpL19 PE=1 SV=2	4.16E-113	84.343	99
P_KWMTBO MO02500	tr|H9IZ97|H9IZ9 7_BOMMO	Uncharacterized protein OS=Bombyx mori OX=7091 PE=4 SV=1	0	90.773	100	sp|O75439|MPP B_HUMAN	Mitochondrial-processing peptidase subunit beta OS=Homo sapiens OX=9606 GN=PMPCB PE=1 SV=2	0	68	96.56652361
P_KWMTBO MO02734	tr|Q9BPS1|Q9BP S1_BOMMO	Elongation factor 1 delta OS=Bombyx mori OX=7091 GN=ef-1d PE=2 SV=1	9.74E-145	100	100	sp|P32192|EF1D _ARTSA	Elongation factor 1-delta OS=Artemia salina OX=85549 PE=1 SV=2	5.90E-72	62.632	93.1372549
P_KWMTBO MO02775	tr|H9J1V6|H9J1 V6_BOMMO	Very-long-chain enoyl-CoA reductase OS=Bombyx mori OX=7091 GN=101742899 PE=3 SV=1	0	100	100	sp|Q9N5Y2|TEC R_CAEEL	Probable very-long-chain enoyl-CoA reductase art-1 OS=Caenorhabditis elegans OX=6239 GN=art-1 PE=3 SV=1	6.15E-119	56.209	100
P_KWMTBO MO02835	tr|H9J2D4|H9J2 D4_BOMMO	RPN13_C domain-containing protein OS=Bombyx mori OX=7091 PE=3 SV=1	0	97.136	100	sp|Q6NZ09|ADR M1_DANRE	Proteasomal ubiquitin receptor ADRM1 OS=Danio rerio OX=7955 GN=adrm1b PE=1 SV=1	1.84E-92	45.524	96.06879607
P_KWMTBO MO02994	tr|H9J281|H9J28 1_BOMMO	DNA photolyase OS=Bombyx mori OX=7091 PE=3 SV=1	0	100	96.04519774	sp|Q28811|PHR_ POTTR	Deoxyribodipyrimidine photo-lyase OS=Potorous tridactylus OX=9310 GN=PHR PE=2 SV=1	0	59.205	90.01883239
P_KWMTBO MO03067	tr|H9J8B0|H9J8 B0_BOMMO	Tripeptidyl-peptidase 2 OS=Bombyx mori OX=7091 GN=101742969 PE=4 SV=1	0	100	100	sp|P29144|TPP2 _HUMAN	Tripeptidyl-peptidase 2 OS=Homo sapiens OX=9606 GN=TPP2 PE=1 SV=4	0	41.24	100
P_KWMTBO MO03113	tr|Q2F5V9|Q2F5 V9_BOMMO	Translocon-associated protein subunit beta OS=Bombyx mori OX=7091 PE=2 SV=1	1.01E-139	100	100	sp|P43308|SSRB _HUMAN	Translocon-associated protein subunit beta OS=Homo sapiens OX=9606 GN=SSR2 PE=1 SV=1	2.35E-60	52.809	93.68421053
P_KWMTBO MO03177	tr|H9JV99|H9JV 99_BOMMO	BRO1 domain-containing protein OS=Bombyx mori OX=7091 PE=4 SV=1	0	97.718	100	sp|Q9QZA2|PDC 6I_RAT	Programmed cell death 6-interacting protein OS=Rattus norvegicus OX=10116 GN=Pdcd6ip PE=1 SV=2	0	43.273	98.36289222
P_KWMTBO MO03430	tr|Q2F5R4|Q2F5 R4_BOMMO	Glutamate dehydrogenase (NAD(P)(+)) OS=Bombyx mori OX=7091 GN=692939 PE=2 SV=1	0	100	74.38692098	sp|P54385|DHE3 _DROME	Glutamate dehydrogenase, mitochondrial OS=Drosophila melanogaster OX=7227 GN=Gdh PE=1 SV=2	0	79.821	76.29427793
P_KWMTBO MO03626	tr|H9J9S0|H9J9S 0_BOMMO	CCT-alpha OS=Bombyx mori OX=7091 GN=101742382 PE=3 SV=1	0	100	100	sp|P12613|TCPA _DROME	T-complex protein 1 subunit alpha OS=Drosophila melanogaster OX=7227 GN=CCT1 PE=2 SV=2	0	80.576	100
P_KWMTBO MO03722	tr|H9JKE1|H9JK E1_BOMMO	Dynamin-type G domain-containing protein OS=Bombyx mori OX=7091 PE=4 SV=1	0	100	76.69172932	sp|Q9H4M9|EH D1_HUMAN	EH domain-containing protein 1 OS=Homo sapiens OX=9606 GN=EHD1 PE=1 SV=2	0	69.101	100
P_KWMTBO MO03860	tr|H9JKX7|H9JK X7_BOMMO	Uncharacterized protein OS=Bombyx mori OX=7091 PE=3 SV=1	0	97.603	100	sp|P26686|SRR5 5_DROME	Serine-arginine protein 55 OS=Drosophila melanogaster OX=7227 GN=B52 PE=1 SV=4	6.44E-97	77.487	66.78321678
P_KWMTBO MO03951	tr|Q5UAL6|Q5U AL6_BOMMO	40S ribosomal protein S27 OS=Bombyx mori OX=7091 GN=RpS27 PE=2 SV=1	1.35E-59	100	100	sp|P55833|RS27 _HOMAM	40S ribosomal protein S27 OS=Homarus americanus OX=6706 GN=RPS27 PE=3 SV=2	1.42E-47	78.571	100
P_KWMTBO MO03986	tr|Q9NL60|Q9N L60_BOMMO	Annexin OS=Bombyx mori OX=7091 PE=2 SV=1	0	99.69	100	sp|P22464|ANX B9_DROME	Annexin B9 OS=Drosophila melanogaster OX=7227 GN=AnxB9 PE=2 SV=2	9.67E-180	76.012	99.38080495

P_KWMTBO MO04168	tr|H9JGM3|H9J GM3_BOMMO	Uncharacterized protein OS=Bombyx mori OX=7091 PE=3 SV=1	0	100	100	sp|Q94516|AT5F 1_DROME	ATP synthase subunit b, mitochondrial OS=Drosophila melanogaster OX=7227 GN=ATPsynB PE=2 SV=2	1.44E-101	69.036	81.06995885
P_KWMTBO MO04370	tr|H9J6V7|H9J6 V7_BOMMO	OBG-type G domain-containing protein OS=Bombyx mori OX=7091 PE=4 SV=1	2.07E-172	99.156	64.57765668	sp|P32234|128U P_DROME	GTP-binding protein 128up OS=Drosophila melanogaster OX=7227 GN=128up PE=2 SV=2	0	89.946	100
P_KWMTBO MO04502	tr|Q5TLD3|Q5T LD3_BOMMO	Replication protein A subunit OS=Bombyx mori OX=7091 GN=BmRPA1 PE=2 SV=1	0	99.498	100	sp|Q24492|RFA1 _DROME	Replication protein A 70 kDa DNA-binding subunit OS=Drosophila melanogaster OX=7227 GN=RpA-70 PE=1 SV=1	0	47.421	100
P_KWMTBO MO04529	tr|Q2F6C7|Q2F6 C7_BOMMO	ADP-ribosylation factor OS=Bombyx mori OX=7091 GN=778456 PE=2 SV=1	9.95E-136	100	100	sp|P61210|ARF1 _LOCMI	ADP-ribosylation factor 1 OS=Locusta migratoria OX=7004 GN=ARF1 PE=2 SV=2	7.09E-134	98.901	100
P_KWMTBO MO04605	tr|H9J7K1|H9J7 K1_BOMMO	2-phospho-D-glycerate hydro-lyase OS=Bombyx mori OX=7091 PE=3 SV=1	0	100	100	sp|P15007|ENO_ DROME	Enolase OS=Drosophila melanogaster OX=7227 GN=Eno PE=1 SV=2	0	81.481	99.76905312
P_KWMTBO MO04610	tr|H9J723|H9J72 3_BOMMO	26S proteasome non-ATPase regulatory subunit 2 OS=Bombyx mori OX=7091 PE=3 SV=1	0	97.303	100	sp|Q5R9I6|PSM D2_PONAB	26S proteasome non-ATPase regulatory subunit 2 OS=Pongo abelii OX=9601 GN=PSMD2 PE=2 SV=1	0	55.556	98.05825243
P_KWMTBO MO04676	tr|H9J7M9|H9J7 M9_BOMMO	Uncharacterized protein OS=Bombyx mori OX=7091 PE=3 SV=1	0	96.91	100	sp|O00291|HIP1 _HUMAN	Huntingtin-interacting protein 1 OS=Homo sapiens OX=9606 GN=HIP1 PE=1 SV=5	4.30E-110	39.219	35.89059373
P_KWMTBO MO04768	tr|H9JK00|H9JK 00_BOMMO	Valyl-tRNA synthetase OS=Bombyx mori OX=7091 PE=3 SV=1	0	93.973	100	sp|Q04462|SYV C_RAT	Valine--tRNA ligase OS=Rattus norvegicus OX=10116 GN=Vars1 PE=2 SV=2	0	59.277	100
P_KWMTBO MO04789	tr|Q2F5P7|Q2F5 P7_BOMMO	Mitochondrial matrix protein p32 OS=Bombyx mori OX=7091 PE=2 SV=1	0	100	100	sp|Q3T0B6|C1Q BP_BOVIN	Complement component 1 Q subcomponent-binding protein, mitochondrial OS=Bos taurus OX=9913 GN=C1QBP PE=2 SV=1	7.30E-40	32.042	100
P_KWMTBO MO04806	tr|H9JK60|H9JK 60_BOMMO	Ubiquitin-activating enzyme E1 OS=Bombyx mori OX=7091 GN=101739389 PE=3 SV=1	0	99.904	100	sp|Q5U300|UBA 1_RAT	Ubiquitin-like modifier-activating enzyme 1 OS=Rattus norvegicus OX=10116 GN=Uba1 PE=1 SV=1	0	65.034	97.8906999
P_KWMTBO MO05258	tr|H9IZ08|H9IZ0 8_BOMMO	Peptidase_M24 domain-containing protein OS=Bombyx mori OX=7091 GN=101746307 PE=3 SV=1	0	100	100	sp|Q6AYD3|PA2 G4_RAT	Proliferation-associated protein 2G4 OS=Rattus norvegicus OX=10116 GN=Pa2g4 PE=1 SV=1	1.26E-149	57.778	94.98680739
P_KWMTBO MO05541	tr|C7AQP4|C7A QP4_BOMMO	Calcium-transporting ATPase OS=Bombyx mori OX=7091 PE=2 SV=1	0	99.301	100	sp|Q7PPA5|ATC 1_ANOGA	Calcium-transporting ATPase sarcoplasmic/endoplasmic reticulum type OS=Anopheles gambiae OX=7165 GN=SERCA PE=3 SV=5	0	89.279	99.6007984
P_KWMTBO MO05564	tr|Q2F6A6|Q2F6 A6_BOMMO	Elongation factor Tu OS=Bombyx mori OX=7091 PE=2 SV=1	0	100	100	sp|P49411|EFTU _HUMAN	Elongation factor Tu, mitochondrial OS=Homo sapiens OX=9606 GN=TUFM PE=1 SV=2	0	65.672	86.4516129
P_KWMTBO MO05566	tr|Q2F5M1|Q2F5 M1_BOMMO	GTP-binding nuclear protein OS=Bombyx mori OX=7091 GN=692970 PE=2 SV=1	1.62E-162	100	100	sp|Q9VZ23|RAN _DROME	GTP-binding nuclear protein Ran OS=Drosophila melanogaster OX=7227 GN=Ran PE=1 SV=1	2.67E-147	91.429	98.5915493
P_KWMTBO MO05612	tr|H9JB77|H9JB 77_BOMMO	SAM_MT_RSMB_NOP domain-containing protein OS=Bombyx mori OX=7091 GN=101741314 PE=3 SV=1	0	99.874	100	sp|Q922K7|NOP 2_MOUSE	Probable 28S rRNA (cytosine-C(5))-methyltransferase OS=Mus musculus OX=10090 GN=Nop2 PE=1 SV=1	0	53.565	72.69279393
P_KWMTBO MO05741	tr|A1E129|A1E1 29_BOMMO	Kinesin-like protein OS=Bombyx mori OX=7091 PE=3 SV=2	0	99.17	100	sp|P17210|KINH _DROME	Kinesin heavy chain OS=Drosophila melanogaster OX=7227 GN=Khc PE=1 SV=2	0	74.871	100
P_KWMTBO MO05975	tr|H9J059|H9J05 9_BOMMO	Tr-type G domain-containing protein OS=Bombyx mori OX=7091 PE=4 SV=1	0	100	100	sp|Q5R8Q7|GTP B1_PONAB	GTP-binding protein 1 (Fragment) OS=Pongo abelii OX=9601 GN=GTPBP1 PE=2 SV=2	2.41E-105	74.641	75.72463768
P_KWMTBO MO06082	tr|H9IWS5|H9I WS5_BOMMO	Uncharacterized protein OS=Bombyx mori OX=7091 GN=101739385 PE=4 SV=1	0	100	100	--	--	--	--	--
P_KWMTBO MO06229	tr|H9IX17|H9IX 17_BOMMO	Ribos_L4_asso_C domain-containing protein OS=Bombyx mori OX=7091 GN=692659 PE=3 SV=1	0	100	100	sp|P09180|RL4_ DROME	60S ribosomal protein L4 OS=Drosophila melanogaster OX=7227 GN=RpL4 PE=1 SV=2	0	68.407	88.24884793
P_KWMTBO MO06231	tr|H9IX18|H9IX 18_BOMMO	RuvB-like helicase OS=Bombyx mori OX=7091 PE=3 SV=1	1.12E-166	98.765	65.14745308	sp|Q9DE27|RUV B2_XENLA	RuvB-like 2 OS=Xenopus laevis OX=8355 GN=ruvbl2 PE=2 SV=1	0	81.501	100

P_KWMTBOMO06480	sp|Q5UAS1|RL18_BOMMO	60S ribosomal protein L18 OS=Bombyx mori OX=7091 GN=RpL18 PE=2 SV=1	1.41E-132	100	100	sp|Q5UAS1|RL18_BOMMO	60S ribosomal protein L18 OS=Bombyx mori OX=7091 GN=RpL18 PE=2 SV=1	9.16E-132	100	100
P_KWMTBOMO06595	tr|I3VR81|I3VR81_BOMMO	Aminopeptidase OS=Bombyx mori OX=7091 PE=2 SV=1	0	100	100	sp|P55786|PSA_HUMAN	Puromycin-sensitive aminopeptidase OS=Homo sapiens OX=9606 GN=NPEPPS PE=1 SV=2	0	57.324	100
P_KWMTBOMO06660	tr|Q2F640|Q2F640_BOMMO	Ubiquinol-cytochrome c reductase core protein II OS=Bombyx mori OX=7091 GN=100127121 PE=2 SV=1	0	100	100	sp|P22695|QCR2_HUMAN	Cytochrome b-c1 complex subunit 2, mitochondrial OS=Homo sapiens OX=9606 GN=UQCRC2 PE=1 SV=3	1.22E-72	34.615	95.19450801
P_KWMTBOMO06763	tr|H9JR70|H9JR70_BOMMO	Uncharacterized protein OS=Bombyx mori OX=7091 PE=4 SV=1	0	99.694	100	--	--	--	--	--
P_KWMTBOMO06770	tr|H9JRG2|H9JRG2_BOMMO	Carboxylic ester hydrolase OS=Bombyx mori OX=7091 PE=3 SV=1	0	100	89.4934334	sp|P35502|ESTF_MYZPE	Esterase FE4 OS=Myzus persicae OX=13164 PE=1 SV=1	8.36E-86	35.577	97.56097561
P_KWMTBOMO06974	tr|Q5UAQ1|Q5UAQ1_BOMMO	Ribosomal protein L36A OS=Bombyx mori OX=7091 GN=RpL36A PE=2 SV=1	2.04E-71	100	100	sp|Q9NB33|RL44_OCHTR	60S ribosomal protein L44 OS=Ochlerotatus triseriatus OX=7162 GN=RpL44 PE=3 SV=3	5.04E-61	85.577	100
P_KWMTBOMO06999	tr|H9JLS3|H9JLS3_BOMMO	TPR_REGION domain-containing protein OS=Bombyx mori OX=7091 PE=3 SV=1	0	100	100	sp|P31948|STIP1_HUMAN	Stress-induced-phosphoprotein 1 OS=Homo sapiens OX=9606 GN=STIP1 PE=1 SV=1	0	52.007	100
P_KWMTBOMO07323	tr|Q2F6C4|Q2F6C4_BOMMO	T-complex protein 1 subunit delta OS=Bombyx mori OX=7091 PE=2 SV=1	0	99.814	100	sp|Q9NB32|TCPD_OCHTR	T-complex protein 1 subunit delta OS=Ochlerotatus triseriatus OX=7162 PE=2 SV=1	0	69.088	100
P_KWMTBOMO07374	tr|Q0ZAL3|Q0ZAL3_BOMMO	Splicing factor proline-and glutamine-rich OS=Bombyx mori OX=7091 GN=SFPQ PE=2 SV=1	0	99.802	100	sp|Q9GRW7|NONA_DROVI	Protein no-on-transient A OS=Drosophila virilis OX=7244 GN=nonA PE=2 SV=1	5.73E-114	61.132	52.47524752
P_KWMTBOMO07554	tr|H9J8L8|H9J8L8_BOMMO	Uncharacterized protein OS=Bombyx mori OX=7091 PE=4 SV=1	8.48E-153	100	53.18627451	sp|Q9NQC3|RTN4_HUMAN	Reticulon-4 OS=Homo sapiens OX=9606 GN=RTN4 PE=1 SV=2	2.84E-61	45.494	57.10784314
P_KWMTBOMO07589	tr|A0A023SG69|A0A023SG69_BOMMO	Coatomer subunit beta OS=Bombyx mori OX=7091 GN=beta-COP PE=4 SV=1	0	93.705	100	sp|Q5ZIA5|COPB_CHICK	Coatomer subunit beta OS=Gallus gallus OX=9031 GN=COPB1 PE=2 SV=1	0	57.39	100
P_KWMTBOMO07772	tr|Q5UAN4|Q5UAN4_BOMMO	Ribosomal protein S10 OS=Bombyx mori OX=7091 GN=RpS10 PE=2 SV=1	4.40E-118	100	100	sp|Q962R9|RS10_SPOFR	40S ribosomal protein S10 OS=Spodoptera frugiperda OX=7108 GN=RpS10 PE=2 SV=1	1.19E-105	92.453	99.375
P_KWMTBOMO07855	tr|Q2F6C2|Q2F6C2_BOMMO	CCT-beta OS=Bombyx mori OX=7091 GN=692797 PE=2 SV=1	0	100	100	sp|Q3ZBH0|TCPB_BOVIN	T-complex protein 1 subunit beta OS=Bos taurus OX=9913 GN=CCT2 PE=1 SV=3	0	72.15	99.81343284
P_KWMTBOMO07857	tr|Q5R1P5|Q5R1P5_BOMMO	Heat shock protein hsp21.4 OS=Bombyx mori OX=7091 GN=Hsp21.4 PE=2 SV=1	4.58E-139	100	100	sp|Q00649|HSPB1_CHICK	Heat shock protein beta-1 OS=Gallus gallus OX=9031 GN=HSPB1 PE=2 SV=1	5.62E-19	47.727	47.05882353
P_KWMTBOMO08064	tr|Q5UAR1|Q5UAR1_BOMMO	60S ribosomal protein L27a OS=Bombyx mori OX=7091 GN=RpL27A PE=2 SV=1	3.15E-107	100	100	sp|P47830|RL27A_XENLA	60S ribosomal protein L27a OS=Xenopus laevis OX=8355 GN=rpl27a PE=2 SV=2	1.19E-82	77.027	100
P_KWMTBOMO08106	tr|H9IUY0|H9IUY0_BOMMO	Clathrin light chain OS=Bombyx mori OX=7091 PE=3 SV=1	2.92E-149	98.095	100	sp|Q9VWA1|CLC_DROME	Clathrin light chain OS=Drosophila melanogaster OX=7227 GN=Clc PE=2 SV=1	5.76E-40	46.948	100
P_KWMTBOMO08193	tr|H9JIX2|H9JIX2_BOMMO	Uncharacterized protein OS=Bombyx mori OX=7091 PE=3 SV=1	2.11E-91	98.571	100	sp|O95831|AIFM1_HUMAN	Apoptosis-inducing factor 1, mitochondrial OS=Homo sapiens OX=9606 GN=AIFM1 PE=1 SV=1	4.02E-30	42.958	100
P_KWMTBOMO08310	tr|Q1HQB9|Q1HQB9_BOMMO	Nucleosome assembly protein isoform 1 OS=Bombyx mori OX=7091 PE=2 SV=1	0	100	100	sp|Q5U2Z3|NP1L4_RAT	Nucleosome assembly protein 1-like 4 OS=Rattus norvegicus OX=10116 GN=Nap1l4 PE=1 SV=1	4.23E-97	49.462	94.17721519
P_KWMTBOMO08323	tr|H9JI91|H9JI91_BOMMO	Uncharacterized protein OS=Bombyx mori OX=7091 PE=4 SV=1	0	99.643	98.24561404	sp|Q5R4J9|NAA15_PONAB	N-alpha-acetyltransferase 15, NatA auxiliary subunit OS=Pongo abelii OX=9601 GN=NAA15 PE=2 SV=1	4.44E-47	41.985	91.92982456
P_KWMTBOMO08440	tr|Q5UAM6|Q5UAM6_BOMMO	Ribosomal protein S16 OS=Bombyx mori OX=7091 GN=RpS16 PE=2 SV=1	8.57E-109	100	100	sp|Q95V31|RS16_SPOFR	40S ribosomal protein S16 OS=Spodoptera frugiperda OX=7108 GN=RpS16 PE=2 SV=1	1.88E-104	98.013	100
P_KWMTBOMO08578	tr|H9JDK4|H9JDK4_BOMMO	Multifunctional fusion protein OS=Bombyx mori OX=7091 GN=101741966 PE=3 SV=1	0	100	100	sp|Q7SY23|AL4A1_DANRE	Delta-1-pyrroline-5-carboxylate dehydrogenase, mitochondrial OS=Danio rerio OX=7955 GN=aldh4a1 PE=2 SV=1	0	55.159	94.33628319

P_KWMTBOMO08607	tr|H9JEL8|H9JEL8_BOMMO	Uncharacterized protein OS=Bombyx mori OX=7091 PE=4 SV=1	2.44E-142	98.974	54.31754875	sp|Q5M9F1|RBM34_RAT	RNA-binding protein 34 OS=Rattus norvegicus OX=10116 GN=Rbm34 PE=1 SV=1	8.14E-07	26.087	64.06685237
P_KWMTBOMO08648	tr|H9JEK1|H9JEK1_BOMMO	Uncharacterized protein OS=Bombyx mori OX=7091 PE=3 SV=1	0	91.125	100	sp|P13395|SPTCA_DROME	Spectrin alpha chain OS=Drosophila melanogaster OX=7227 GN=alpha-Spec PE=1 SV=2	0	76.124	100
P_KWMTBOMO08666	tr|Q66SV4|Q66SV4_BOMMO	40S ribosomal protein S23 OS=Bombyx mori OX=7091 GN=S23 PE=2 SV=1	5.48E-103	100	100	sp|Q6EV23|RS23_PAPDA	40S ribosomal protein S23 OS=Papilio dardanus OX=77259 GN=RpS23 PE=2 SV=1	6.27E-101	97.902	100
P_KWMTBOMO08820	tr|Q1HPI5|Q1HPI5_BOMMO	Nucleoside diphosphate kinase OS=Bombyx mori OX=7091 PE=2 SV=1	4.74E-113	100	100	sp|P08879|NDKA_DROME	Nucleoside diphosphate kinase OS=Drosophila melanogaster OX=7227 GN=awd PE=1 SV=3	5.17E-94	81.046	100
P_KWMTBOMO08893	tr|H9JE77|H9JE77_BOMMO	Succinate--CoA ligase [GDP-forming] subunit beta, mitochondrial OS=Bombyx mori OX=7091 GN=101740061 PE=3 SV=1	0	100	100	sp|Q96I99|SUCB2_HUMAN	Succinate--CoA ligase [GDP-forming] subunit beta, mitochondrial OS=Homo sapiens OX=9606 GN=SUCLG2 PE=1 SV=2	1.11E-159	59.231	92.63657957
P_KWMTBOMO08956	tr|H9JDY1|H9JDY1_BOMMO	M20_dimer domain-containing protein OS=Bombyx mori OX=7091 PE=3 SV=1	0	99.784	96.86847599	sp|Q3ZC84|CNDP2_BOVIN	Cytosolic non-specific dipeptidase OS=Bos taurus OX=9913 GN=CNDP2 PE=2 SV=1	0	58.65	98.95615866
P_KWMTBOMO08961	tr|Q5UAQ8|Q5UAQ8_BOMMO	60S ribosomal protein L30 OS=Bombyx mori OX=7091 GN=RpL30 PE=2 SV=1	2.87E-80	100	100	sp|P58375|RL30_SPOFR	60S ribosomal protein L30 OS=Spodoptera frugiperda OX=7108 GN=RpL30 PE=3 SV=1	9.75E-78	99.099	98.2300885
P_KWMTBOMO09055	tr|H9JDA5|H9JDA5_BOMMO	Proton-translocating NAD(P)(+) transhydrogenase OS=Bombyx mori OX=7091 PE=3 SV=1	0	93.082	100	sp|P11024|NNTM_BOVIN	NAD(P) transhydrogenase, mitochondrial OS=Bos taurus OX=9913 GN=NNT PE=1 SV=3	0	59.925	100
P_KWMTBOMO09230	tr|H9J1M5|H9J1M5_BOMMO	Uncharacterized protein OS=Bombyx mori OX=7091 PE=3 SV=1	3.45E-75	100	100	sp|P08570|RLA1_DROME	60S acidic ribosomal protein P1 OS=Drosophila melanogaster OX=7227 GN=RpLP1 PE=1 SV=2	1.01E-42	81.25	100
P_KWMTBOMO09355	tr|Q6T3A7|Q6T3A7_BOMMO	Thiol peroxiredoxin OS=Bombyx mori OX=7091 GN=692638 PE=2 SV=1	5.60E-148	100	100	sp|Q9V3P0|PRDX1_DROME	Peroxiredoxin 1 OS=Drosophila melanogaster OX=7227 GN=Jafrac1 PE=1 SV=1	8.32E-117	81.152	97.94871795
P_KWMTBOMO09415	tr|H9JTB0|H9JTB0_BOMMO	RNA helicase OS=Bombyx mori OX=7091 GN=101741115 PE=4 SV=1	0	99.765	100	sp|Q27268|DX39B_DROME	ATP-dependent RNA helicase WM6 OS=Drosophila melanogaster OX=7227 GN=Hel25E PE=1 SV=1	0	88.498	100
P_KWMTBOMO09442	tr|Q5UAT5|Q5UAT5_BOMMO	60S ribosomal protein L6 OS=Bombyx mori OX=7091 GN=RpL6 PE=2 SV=1	0	99.634	100	sp|Q6QMZ4|RL6_CHILA	60S ribosomal protein L6 OS=Chinchilla lanigera OX=34839 GN=RPL6 PE=2 SV=3	4.24E-65	48.8	91.57509158
P_KWMTBOMO09542	tr|H9JU13|H9JU13_BOMMO	Eukaryotic translation initiation factor 3 subunit A OS=Bombyx mori OX=7091 PE=3 SV=1	0	96.154	90.33898305	sp|Q173M7|EIF3A_AEDAE	Eukaryotic translation initiation factor 3 subunit A OS=Aedes aegypti OX=7159 GN=eIF3-S10 PE=3 SV=1	0	66.215	68.72881356
P_KWMTBOMO09621	tr|H9JTI9|H9JTI9_BOMMO	Eukaryotic translation initiation factor 3 subunit C OS=Bombyx mori OX=7091 GN=eIF3c PE=3 SV=1	0	100	100	sp|Q0ZB76|EIF3C_BOMMO	Eukaryotic translation initiation factor 3 subunit C OS=Bombyx mori OX=7091 GN=eIF3-S8 PE=2 SV=1	0	99.886	100
P_KWMTBOMO09747	tr|B7XFU6|B7XFU6_BOMMO	Clathrin heavy chain OS=Bombyx mori OX=7091 GN=100233163 PE=2 SV=1	0	99.941	100	sp|P29742|CLH_DROME	Clathrin heavy chain OS=Drosophila melanogaster OX=7227 GN=Chc PE=1 SV=1	0	85.196	100
P_KWMTBOMO09808	tr|Q2F6C3|Q2F6C3_BOMMO	Chaperonin subunit 6a zeta OS=Bombyx mori OX=7091 GN=692796 PE=2 SV=1	0	100	100	sp|P80317|TCPZ_MOUSE	T-complex protein 1 subunit zeta OS=Mus musculus OX=10090 GN=Cct6a PE=1 SV=3	0	72.505	100
P_KWMTBOMO10248	tr|H9JCR4|H9JCR4_BOMMO	40S ribosomal protein SA OS=Bombyx mori OX=7091 PE=3 SV=1	0	100	100	sp|Q5UAP4|RSSA_BOMMO	40S ribosomal protein SA OS=Bombyx mori OX=7091 PE=2 SV=1	0	99.673	100
P_KWMTBOMO10283	tr|H9JCM2|H9JCM2_BOMMO	PCI domain-containing protein OS=Bombyx mori OX=7091 GN=692900 PE=3 SV=1	0	100	100	sp|Q9D8W5|PSD12_MOUSE	26S proteasome non-ATPase regulatory subunit 12 OS=Mus musculus OX=10090 GN=Psmd12 PE=1 SV=4	0	59.071	100
P_KWMTBOMO10358	tr|H9JX26|H9JX26_BOMMO	DUF4781 domain-containing protein OS=Bombyx mori OX=7091 PE=4 SV=1	0	79.827	48.32869081	--	--	--	--	--
P_KWMTBOMO10493	tr|H9JC69|H9JC69_BOMMO	Eukaryotic translation initiation factor 3 subunit B OS=Bombyx mori OX=7091 PE=3 SV=1	0	99.568	100	sp|Q1HDZ5|EIF3B_BOMMO	Eukaryotic translation initiation factor 3 subunit B OS=Bombyx mori OX=7091 GN=eIF3-S9 PE=2 SV=1	0	99.568	100

P_KWMTBO MO10780	tr|H9JFX9|H9JF X9_BOMMO	Uncharacterized protein OS=Bombyx mori OX=7091 PE=4 SV=1	0	90.138	43.81909548	sp|Q9V3P6|PSM D1_DROME	26S proteasome non-ATPase regulatory subunit 1 OS=Drosophila melanogaster OX=7227 GN=Rpn2 PE=1 SV=1	0	71.218	100
P_KWMTBO MO10916	tr|Q1HQ44|Q1H Q44_BOMMO	Proteasome subunit alpha type OS=Bombyx mori OX=7091 PE=2 SV=1	0	100	100	sp|O70435|PSA3 _MOUSE	Proteasome subunit alpha type-3 OS=Mus musculus OX=10090 GN=Psma3 PE=1 SV=3	2.47E-130	65.49	100
P_KWMTBO MO11045	tr|H9JRL1|H9JR L1_BOMMO	Uncharacterized protein OS=Bombyx mori OX=7091 PE=3 SV=1	0	28.363	91.51958674	sp|Q00963|SPTC B_DROME	Spectrin beta chain OS=Drosophila melanogaster OX=7227 GN=beta-Spec PE=1 SV=2	0	73.055	100
P_KWMTBO MO11061	tr|Q2F5N1|Q2F5 N1_BOMMO	Prohibitin OS=Bombyx mori OX=7091 GN=693108 PE=2 SV=1	0	100	100	sp|Q5XIH7|PHB 2_RAT	Prohibitin-2 OS=Rattus norvegicus OX=10116 GN=Phb2 PE=1 SV=1	5.66E-158	72.667	100
P_KWMTBO MO11096	tr|A1E9B3|A1E9 B3_BOMMO	H(+)-transporting two-sector ATPase OS=Bombyx mori OX=7091 PE=2 SV=1	0	99.676	100	sp|P31400|VAT A_MANSE	V-type proton ATPase catalytic subunit A OS=Manduca sexta OX=7130 GN=VHAA PE=2 SV=1	0	98.217	100
P_KWMTBO MO11202	tr|H9JT03|H9JT0 3_BOMMO	F-actin-capping protein subunit alpha OS=Bombyx mori OX=7091 GN=101738938 PE=3 SV=1	0	100	100	sp|Q9W2N0|CA PZA_DROME	F-actin-capping protein subunit alpha OS=Drosophila melanogaster OX=7227 GN=cpa PE=2 SV=1	1.31E-147	68.44	97.91666667
P_KWMTBO MO11255	tr|Q5UAN5|Q5U AN5_BOMMO	40S ribosomal protein S9 OS=Bombyx mori OX=7091 GN=RpS9 PE=2 SV=1	5.62E-140	100	100	sp|B4PEU8|RS9 _DROYA	40S ribosomal protein S9 OS=Drosophila yakuba OX=7245 GN=RpS9 PE=2 SV=1	2.83E-117	89.231	100
P_KWMTBO MO11448	tr|H9IXE5|H9IX E5_BOMMO	Uncharacterized protein OS=Bombyx mori OX=7091 GN=733057 PE=4 SV=1	1.36E-160	100	100	sp|P53994|RAB2 A_MOUSE	Ras-related protein Rab-2A OS=Mus musculus OX=10090 GN=Rab2a PE=1 SV=1	2.20E-141	90.654	100
P_KWMTBO MO11517	tr|H9J3H3|H9J3 H3_BOMMO	Uncharacterized protein OS=Bombyx mori OX=7091 PE=4 SV=1	0	96.387	98.84169884	--	--	--	--	--
P_KWMTBO MO11590	tr|H9J3D2|H9J3 D2_BOMMO	Ku domain-containing protein OS=Bombyx mori OX=7091 GN=101736121 PE=4 SV=1	0	98.077	80.68965517	sp|O93257|XRC C6_CHICK	X-ray repair cross-complementing protein 5 OS=Gallus gallus OX=9031 GN=XRCC6 PE=2 SV=1	5.59E-42	26.126	95.68965517
P_KWMTBO MO11666	tr|Q2V0H5|Q2V 0H5_BOMMO	Vesicle-fusing ATPase OS=Bombyx mori OX=7091 GN=ter94 PE=2 SV=1	0	100	100	sp|Q7KN62|TER A_DROME	Transitional endoplasmic reticulum ATPase TER94 OS=Drosophila melanogaster OX=7227 GN=TER94 PE=1 SV=1	0	88.025	100
P_KWMTBO MO11680	tr|H9J390|H9J39 0_BOMMO	Seryl-tRNA synthetase OS=Bombyx mori OX=7091 GN=101743384 PE=3 SV=1	0	100	100	sp|Q9GMB8|SY SC_BOVIN	Serine--tRNA ligase, cytoplasmic OS=Bos taurus OX=9913 GN=SARS1 PE=2 SV=3	0	69.495	100
P_KWMTBO MO11771	tr|Q1HQ18|Q1H Q18_BOMMO	Proteasome endopeptidase complex OS=Bombyx mori OX=7091 GN=100101166 PE=2 SV=1	4.64E-178	100	100	sp|P40304|PSB1 _DROME	Proteasome subunit beta type-1 OS=Drosophila melanogaster OX=7227 GN=Prosbeta6 PE=2 SV=2	3.74E-105	62.009	98.70689655
P_KWMTBO MO12018	tr|Q5UAN7|Q5U AN7_BOMMO	40S ribosomal protein S7 OS=Bombyx mori OX=7091 GN=RpS7 PE=2 SV=1	1.24E-137	99.474	100	sp|P48155|RS7_ MANSE	40S ribosomal protein S7 OS=Manduca sexta OX=7130 GN=RpS7 PE=2 SV=1	1.42E-134	97.368	100
P_KWMTBO MO12166	tr|H9JWZ4|H9J WZ4_BOMMO	NADH dehydrogenase [ubiquinone] 1 alpha subcomplex subunit 10, mitochondrial OS=Bombyx mori OX=7091 GN=101745480 PE=3 SV=1	0	100	100	sp|P91929|NDU AA_DROME	NADH dehydrogenase [ubiquinone] 1 alpha subcomplex subunit 10, mitochondrial OS=Drosophila melanogaster OX=7227 GN=ND-42 PE=1 SV=2	1.80E-134	49.485	97.48743719
P_KWMTBO MO12264	tr|H9JHZ0|H9JH Z0_BOMMO	CYTOSOL_AP domain-containing protein OS=Bombyx mori OX=7091 PE=3 SV=1	0	93.99	79.54110899	sp|Q5R7G6|PEP L1_PONAB	Probable aminopeptidase NPEPL1 OS=Pongo abelii OX=9601 GN=NPEPL1 PE=3 SV=2	0	54.981	99.80879541
P_KWMTBO MO12304	tr|H9JHX3|H9JH X3_BOMMO	4_1_CTD domain-containing protein OS=Bombyx mori OX=7091 PE=4 SV=1	0	98.443	72.63157895	sp|Q9V8R9|EPB 41_DROME	Protein 4.1 homolog OS=Drosophila melanogaster OX=7227 GN=cora PE=1 SV=1	0	52.778	36.49122807
P_KWMTBO MO12389	tr|H9JSC6|H9JS C6_BOMMO	Coatomer subunit epsilon OS=Bombyx mori OX=7091 PE=3 SV=1	0	100	100	sp|Q5ZIK9|COP E_CHICK	Coatomer subunit epsilon OS=Gallus gallus OX=9031 GN=COPE PE=2 SV=1	6.42E-96	48.475	97.68211921
P_KWMTBO MO12428	sp|Q5UAS2|RL1 7_BOMMO	60S ribosomal protein L17 OS=Bombyx mori OX=7091 GN=RpL17 PE=2 SV=1	5.32E-141	100	100	sp|Q5UAS2|RL1 7_BOMMO	60S ribosomal protein L17 OS=Bombyx mori OX=7091 GN=RpL17 PE=2 SV=1	3.47E-140	100	100
P_KWMTBO MO12667	tr|Q1HPM7|Q1H PM7_BOMMO	Mitochondrial aldehyde dehydrogenase OS=Bombyx mori OX=7091 PE=2 SV=1	0	97.291	47.22814499	sp|P81178|ALD H2_MESAU	Aldehyde dehydrogenase, mitochondrial OS=Mesocricetus	0	70	45.84221748

							auratus OX=10036 GN=ALDH2 PE=1 SV=1			
P_KWMTBOMO12844	tr|H9U396|H9U396_BOMMO	Endoplasmin-like protein OS=Bombyx mori OX=7091 PE=2 SV=1	0	100	100	sp|Q66HD0|ENPL_RAT	Endoplasmin OS=Rattus norvegicus OX=10116 GN=Hsp90b1 PE=1 SV=2	0	59.264	93.95061728
P_KWMTBOMO13235	tr|H9JKC3|H9JKC3_BOMMO	Transket_pyr domain-containing protein OS=Bombyx mori OX=7091 PE=3 SV=1	0	83.749	100	sp|Q60HE2|ODO1_MACFA	2-oxoglutarate dehydrogenase, mitochondrial OS=Macaca fascicularis OX=9541 GN=OGDH PE=2 SV=1	0	62.761	98.6287953
P_KWMTBOMO13293	tr|E9JEI6|E9JEI6_BOMMO	Poly [ADP-ribose] polymerase (Fragment) OS=Bombyx mori OX=7091 PE=2 SV=1	0	99.894	94.5	sp|Q11208|PARP_SARPE	Poly [ADP-ribose] polymerase OS=Sarcophaga peregrina OX=7386 PE=1 SV=1	0	48.473	100
P_KWMTBOMO13370	tr|H9JN38|H9JN38_BOMMO	Signal recognition particle subunit SRP72 OS=Bombyx mori OX=7091 PE=3 SV=1	0	100	66.30602782	sp|O76094|SRP72_HUMAN	Signal recognition particle subunit SRP72 OS=Homo sapiens OX=9606 GN=SRP72 PE=1 SV=3	7.11E-121	37.721	96.29057187
P_KWMTBOMO13405	tr|H9JN03|H9JN03_BOMMO	Proliferating cell nuclear antigen OS=Bombyx mori OX=7091 GN=692356 PE=3 SV=1	0	100	100	sp|O01377|PCNA_BOMMO	Proliferating cell nuclear antigen OS=Bombyx mori OX=7091 GN=PCNA PE=2 SV=1	0	98.846	100
P_KWMTBOMO13721	tr|A1YM11|A1YM11_BOMMO	CCT-theta OS=Bombyx mori OX=7091 PE=2 SV=1	0	99.45	100	sp|Q6EE31|TCPQ_CHICK	T-complex protein 1 subunit theta OS=Gallus gallus OX=9031 GN=CCT8 PE=1 SV=3	0	63.72	99.63302752
P_KWMTBOMO13729	tr|Q684K3|Q684K3_BOMMO	Protein-synthesizing GTPase OS=Bombyx mori OX=7091 GN=eIF2g PE=2 SV=1	0	100	100	sp|Q24208|IF2G_DROME	Eukaryotic translation initiation factor 2 subunit 3 OS=Drosophila melanogaster OX=7227 GN=eIF2gamma PE=2 SV=1	0	84.12	99.57264957
P_KWMTBOMO13960	tr|Q5UAN0|Q5UAN0_BOMMO	40S ribosomal protein S13 OS=Bombyx mori OX=7091 GN=RpS13 PE=2 SV=1	8.93E-110	100	100	sp|Q962R6|RS13_SPOFR	40S ribosomal protein S13 OS=Spodoptera frugiperda OX=7108 GN=RpS13 PE=2 SV=3	1.63E-108	99.338	100
P_KWMTBOMO14440	tr|Q1HPP5|Q1HPP5_BOMMO	Actin-depolymerizing factor 1 OS=Bombyx mori OX=7091 PE=2 SV=1	1.70E-108	100	100	sp|P45594|CADF_DROME	Cofilin/actin-depolymerizing factor homolog OS=Drosophila melanogaster OX=7227 GN=tsr PE=1 SV=1	2.22E-100	92.568	100
P_KWMTBOMO14479	tr|H9IS48|H9IS48_BOMMO	Uncharacterized protein OS=Bombyx mori OX=7091 GN=101742289 PE=3 SV=1	7.91E-100	100	100	sp|Q8I1F4|FBRL_DROER	rRNA 2'-O-methyltransferase fibrillarin OS=Drosophila erecta OX=7220 GN=Fib PE=3 SV=1	2.11E-41	82.278	50
P_KWMTBOMO14549	tr|I6XKQ0|I6XKQ0_BOMMO	Heat shock protein 70-5 OS=Bombyx mori OX=7091 PE=2 SV=1	0	99.565	100	sp|P29845|HSP7E_DROME	Heat shock 70 kDa protein cognate 5 OS=Drosophila melanogaster OX=7227 GN=Hsc70-5 PE=1 SV=2	0	81.86	93.47826087
P_KWMTBOMO14732	tr|Q86PG2|Q86PG2_BOMMO	ADP/ATP translocase OS=Bombyx mori OX=7091 GN=ANT PE=2 SV=1	0	100	100	sp|Q26365|ADT_DROME	ADP,ATP carrier protein OS=Drosophila melanogaster OX=7227 GN=sesB PE=2 SV=4	0	87.458	98.33333333
P_KWMTBOMO14761	tr|H9J2S8|H9J2S8_BOMMO	Dihydrolipoyllysine-residue succinyltransferase component of 2-oxoglutarate dehydrogenase complex, mitochondrial OS=Bombyx mori OX=7091 PE=3 SV=1	0	78.71	100	sp|P11179|ODO2_BOVIN	Dihydrolipoyllysine-residue succinyltransferase component of 2-oxoglutarate dehydrogenase complex, mitochondrial OS=Bos taurus OX=9913 GN=DLST PE=1 SV=2	8.56E-150	56.542	92.04301075
P_KWMTBOMO14887	tr|H9JRM2|H9JRM2_BOMMO	T-complex protein 1 subunit eta OS=Bombyx mori OX=7091 PE=3 SV=1	0	99.631	100	sp|Q5ZJK8|TCPH_CHICK	T-complex protein 1 subunit eta OS=Gallus gallus OX=9031 GN=CCT7 PE=1 SV=1	0	69.376	97.60147601
P_KWMTBOMO14984	tr|H9J5J2|H9J5J2_BOMMO	AAA domain-containing protein OS=Bombyx mori OX=7091 PE=3 SV=1	0	100	100	sp|O42587|PR6AA_XENLA	26S proteasome regulatory subunit 6A-A OS=Xenopus laevis OX=8355 GN=psmc3-a PE=2 SV=2	0	88.124	98.36448598
P_KWMTBOMO15060	tr|H9J5R2|H9J5R2_BOMMO	Protein transport protein SEC23 OS=Bombyx mori OX=7091 PE=3 SV=1	0	100	100	sp|Q01405|SC23A_MOUSE	Protein transport protein Sec23A OS=Mus musculus OX=10090 GN=Sec23a PE=1 SV=2	0	74.583	100
P_KWMTBOMO15114	tr|Q5UAL8|Q5UAL8_BOMMO	40S ribosomal protein S25 OS=Bombyx mori OX=7091 GN=RpS25 PE=2 SV=1	7.13E-83	100	100	sp|Q962Q5|RS25_SPOFR	40S ribosomal protein S25 OS=Spodoptera frugiperda OX=7108 GN=RpS25 PE=3 SV=1	1.98E-81	99.16	100
P_KWMTBOMO15123	tr|H9J5W8|H9J5W8_BOMMO	AAA domain-containing protein OS=Bombyx mori OX=7091 PE=3 SV=2	0	99.241	90.38901602	sp|P48601|PRS4_DROME	26S proteasome regulatory subunit 4 OS=Drosophila melanogaster OX=7227 GN=Rpt2 PE=1 SV=2	0	96.811	100
P_KWMTBOMO15357	tr|H9J6H5|H9J6H5_BOMMO	Prolyl-tRNA synthetase OS=Bombyx mori OX=7091 PE=3 SV=1	0	98.315	99.64028777	sp|P28668|SYEP_DROME	Bifunctional glutamate/proline--tRNA ligase OS=Drosophila melanogaster OX=7227 GN=GluProRS PE=1 SV=2	0	61.306	99.16067146

P_KWMTBOMO15359		tr|H9J6H4|H9J6H4_BOMMO	Glutamyl-tRNA synthetase OS=Bombyx mori OX=7091 PE=3 SV=1	0	100	97.09944751		sp|P28668|SYEP_DROME	Bifunctional glutamate/proline--tRNA ligase OS=Drosophila melanogaster OX=7227 GN=GluProRS PE=1 SV=2	0	60.526	99.72375691
P_KWMTBOMO15361		tr|O16143|O16143_BOMMO	Rab1 protein OS=Bombyx mori OX=7091 GN=rabB PE=2 SV=1	5.31E-153	100	100		sp|Q05974|RAB1A_LYMST	Ras-related protein Rab-1A OS=Lymnaea stagnalis OX=6523 GN=RAB1A PE=2 SV=1	4.74E-124	81.188	100
P_KWMTBOMO15518		tr|Q1HPL0|Q1HPL0_BOMMO	26S proteasome regulatory ATPase subunit 10B OS=Bombyx mori OX=7091 PE=2 SV=1	0	100	100		sp|P62334|PRS10_MOUSE	26S proteasome regulatory subunit 10B OS=Mus musculus OX=10090 GN=Psmc6 PE=1 SV=1	0	88.571	97.22222222
P_KWMTBOMO15712		tr|Q2F5N9|Q2F5N9_BOMMO	Nucleoplasmin isoform 2 OS=Bombyx mori OX=7091 GN=692956 PE=2 SV=1	7.05E-137	100	100		sp|Q27415|NLP_DROME	Nucleoplasmin-like protein OS=Drosophila melanogaster OX=7227 GN=Nlp PE=1 SV=1	2.34E-29	48.148	57.7540107
P_KWMTBOMO15715		tr|H9IYH6|H9IYH6_BOMMO	Uncharacterized protein OS=Bombyx mori OX=7091 PE=4 SV=1	0	86.272	98.32817337		--	--	--	--	--
P_KWMTBOMO15758		tr|B3VBE3|B3VBE3_BOMMO	Vacuolar proton pump subunit B OS=Bombyx mori OX=7091 PE=2 SV=1	0	99.278	88.21656051		sp|P31401|VATB_MANSE	V-type proton ATPase subunit B OS=Manduca sexta OX=7130 GN=VHA55 PE=2 SV=1	0	97.112	88.21656051
P_KWMTBOMO15781		tr|H9IYL2|H9IYL2_BOMMO	Uncharacterized protein OS=Bombyx mori OX=7091 PE=3 SV=2	0	87.5	100		sp|O46036|CTBP_DROME	C-terminal-binding protein OS=Drosophila melanogaster OX=7227 GN=CtBP PE=1 SV=3	0	91.643	78.9010989
P_KWMTBOMO16197		tr|Q1HPX4|Q1HPX4_BOMMO	ATP synthase subunit gamma OS=Bombyx mori OX=7091 GN=732965 PE=2 SV=1	0	100	100		sp|O01666|ATPG_DROME	ATP synthase subunit gamma, mitochondrial OS=Drosophila melanogaster OX=7227 GN=ATPsyngamma PE=2 SV=2	2.51E-151	73.05	95.27027027
P_KWMTBOMO16261		tr|Q2F6A0|Q2F6A0_BOMMO	Exuperantia OS=Bombyx mori OX=7091 PE=2 SV=1	0	100	100		sp|Q24747|EXU_DROVI	Maternal protein exuperantia OS=Drosophila virilis OX=7244 GN=exu PE=3 SV=1	5.40E-82	39.231	94.43099274
P_KWMTBOMO16357		tr|Q5UAN3|Q5UAN3_BOMMO	40S ribosomal protein S11 OS=Bombyx mori OX=7091 GN=RpS11-1 PE=2 SV=1	6.00E-114	100	100		sp|P62280|RS11_HUMAN	40S ribosomal protein S11 OS=Homo sapiens OX=9606 GN=RPS11 PE=1 SV=3	4.63E-88	77.848	100
P_KWMTBOMO16524		tr|H9J7Z3|H9J7Z3_BOMMO	RRM domain-containing protein OS=Bombyx mori OX=7091 PE=3 SV=1	1.00E-90	76.064	100		sp|Q5R1W5|SRSF2_PANTR	Serine/arginine-rich splicing factor 2 OS=Pan troglodytes OX=9598 GN=SRSF2 PE=2 SV=3	1.89E-49	77.083	62.33766234

common PIPs of P25

Name	Bombyx-mori	Subject ID	Description	e-value	identity(%)	coverage(%)	Metazoa	Subject ID	Description	e-value	identity(%)	coverage(%)
P_KWMTBOMO00339		tr|H9ITE6|H9ITE6_BOMMO	CSD_1 domain-containing protein OS=Bombyx mori OX=7091 PE=4 SV=1	0	100	100		sp|P41824|YBOXH_APLCA	Y-box factor homolog OS=Aplysia californica OX=6500 PE=2 SV=1	1.24E-50	69.106	47.49034749
P_KWMTBOMO00366		tr|H9ITD6|H9ITD6_BOMMO	Uncharacterized protein OS=Bombyx mori OX=7091 PE=4 SV=1	0	99.775	83.80414313		sp|O42254|IF2B1_CHICK	Insulin-like growth factor 2 mRNA-binding protein 1 OS=Gallus gallus OX=9031 GN=IGF2BP1 PE=1 SV=1	7.53E-128	44.578	93.78531073
P_KWMTBOMO00375		tr|H9ITY5|H9ITY5_BOMMO	Uncharacterized protein OS=Bombyx mori OX=7091 PE=3 SV=1	0	87.972	100		sp|Q9VSA3|ACADM_DROME	Probable medium-chain specific acyl-CoA dehydrogenase, mitochondrial OS=Drosophila melanogaster OX=7227 GN=CG12262 PE=2 SV=1	0	73.558	98.34515366
P_KWMTBOMO00427		tr|Q9NKX3|Q9NKX3_BOMMO	Coatomer subunit gamma OS=Bombyx mori OX=7091 GN=copg1 PE=2 SV=1	0	99.884	100		sp|Q8I0G5|COPG_DROME	Coatomer subunit gamma OS=Drosophila melanogaster OX=7227 GN=gammaCOP PE=2 SV=1	0	57.555	100
P_KWMTBOMO00462		tr|D9N4J4|D9N4J4_BOMMO	Staphylococcal nuclease domain-containing protein OS=Bombyx mori OX=7091 GN=Tudor-SN PE=2 SV=1	0	99.887	100		sp|Q9W0S7|SND1_DROME	Staphylococcal nuclease domain-containing protein 1 OS=Drosophila melanogaster OX=7227 GN=Tudor-SN PE=1 SV=1	0	52.412	100
P_KWMTBOMO00497		tr|H9JRZ9|H9JRZ9_BOMMO	Uncharacterized protein OS=Bombyx mori OX=7091 PE=3 SV=1	0	100	100		sp|Q60HD7|SERA_MACFA	D-3-phosphoglycerate dehydrogenase OS=Macaca fascicularis OX=9541 GN=PHGDH PE=2 SV=4	2.42E-109	50.814	93.88379205
P_KWMTBOMO00638		tr|D2Y4R4|D2Y4R4_BOMMO	Coatomer subunit beta' OS=Bombyx mori OX=7091 GN=COPB2 PE=2 SV=1	0	99.877	99.26918392		sp|O62621|COPB2_DROME	Coatomer subunit beta' OS=Drosophila melanogaster OX=7227 GN=beta'COP PE=2 SV=2	0	71.059	98.90377588

P_KWMTBOMO00794	tr|Q1HQ81|Q1HQ81_BOMMO	Nascent polypeptide associated complex protein alpha subunit OS=Bombyx mori OX=7091 PE=2 SV=1	1.91E-153	100	100	sp|Q94518|NACA_DROME	Nascent polypeptide-associated complex subunit alpha OS=Drosophila melanogaster OX=7227 GN=Nacalpha PE=1 SV=2	1.16E-78	65.455	100
P_KWMTBOMO00838	tr|Q5UAR6|Q5UAR6_BOMMO	60S ribosomal protein L23 OS=Bombyx mori OX=7091 GN=RpL23 PE=2 SV=1	5.38E-99	100	100	sp|P48159|RL23_DROME	60S ribosomal protein L23 OS=Drosophila melanogaster OX=7227 GN=RpL23 PE=1 SV=2	5.37E-94	95	100
P_KWMTBOMO00927	tr|H9JJU3|H9JJU3_BOMMO	Aspartate--tRNA ligase, cytoplasmic OS=Bombyx mori OX=7091 PE=3 SV=1	0	100	71.22040073	sp|Q3SYZ4|SYDC_BOVIN	Aspartate--tRNA ligase, cytoplasmic OS=Bos taurus OX=9913 GN=DARS1 PE=2 SV=1	0	64.329	90.89253188
P_KWMTBOMO01072	tr|H9JD17|H9JD17_BOMMO	Uncharacterized protein OS=Bombyx mori OX=7091 PE=4 SV=1	0	99.688	40.02493766	sp|Q9W3N6|USO1_DROME	General vesicular transport factor p115 OS=Drosophila melanogaster OX=7227 GN=p115 PE=1 SV=2	0	50.619	100
P_KWMTBOMO01075	tr|H9JCV2|H9JCV2_BOMMO	Uncharacterized protein OS=Bombyx mori OX=7091 GN=101741707 PE=3 SV=1	0	99.629	100	sp|O02649|CH60A_DROME	Heat shock protein 60A OS=Drosophila melanogaster OX=7227 GN=Hsp60A PE=1 SV=3	0	84.72	95.91836735
P_KWMTBOMO01273	tr|Q5UAS5|Q5UAS5_BOMMO	60S ribosomal protein L13a OS=Bombyx mori OX=7091 GN=RpL13A PE=2 SV=1	3.08E-151	100	100	sp|Q8MUR4|RL13A_CHOPR	60S ribosomal protein L13a OS=Choristoneura parallela OX=106495 GN=RpL13A PE=2 SV=1	3.13E-140	91.667	100
P_KWMTBOMO01424	tr|H9JHC4|H9JHC4_BOMMO	Elongation factor 1-beta' OS=Bombyx mori OX=7091 PE=3 SV=1	5.51E-165	100	100	sp|P29522|EF1B2_BOMMO	Elongation factor 1-beta' OS=Bombyx mori OX=7091 PE=1 SV=2	3.22E-163	99.55	100
P_KWMTBOMO01452	tr|H9JHD3|H9JHD3_BOMMO	Arginyl-tRNA synthetase OS=Bombyx mori OX=7091 GN=101737924 PE=3 SV=1	0	100	100	sp|Q6P1S4|SYRC_XENTR	Arginine--tRNA ligase, cytoplasmic OS=Xenopus tropicalis OX=8364 GN=rars1 PE=2 SV=1	0	58.85	99.26793558
P_KWMTBOMO01606	tr|H9J993|H9J993_BOMMO	Nop domain-containing protein OS=Bombyx mori OX=7091 GN=101742458 PE=3 SV=1	0	99.436	100	sp|Q9Y2X3|NOP58_HUMAN	Nucleolar protein 58 OS=Homo sapiens OX=9606 GN=NOP58 PE=1 SV=1	0	63.377	86.20037807
P_KWMTBOMO01708	tr|H9J9C9|H9J9C9_BOMMO	Dynein light intermediate chain OS=Bombyx mori OX=7091 PE=3 SV=1	2.61E-111	99.367	34.05172414	sp|Q6PDL0|DC1L2_MOUSE	Cytoplasmic dynein 1 light intermediate chain 2 OS=Mus musculus OX=10090 GN=Dync1li2 PE=1 SV=2	2.12E-142	50.33	98.06034483
P_KWMTBOMO01720	tr|H9J930|H9J930_BOMMO	Uncharacterized protein OS=Bombyx mori OX=7091 PE=4 SV=1	0	98.191	100	sp|Q26457|LA_AEDAL	La protein homolog OS=Aedes albopictus OX=7160 PE=1 SV=1	2.92E-91	44.225	93.42105263
P_KWMTBOMO01918	tr|Q2F5L5|Q2F5L5_BOMMO	Protein transport protein Sec61 subunit beta OS=Bombyx mori OX=7091 GN=733070 PE=2 SV=1	1.66E-65	100	100	sp|P60467|SC61B_CANLF	Protein transport protein Sec61 subunit beta OS=Canis lupus familiaris OX=9615 GN=SEC61B PE=1 SV=2	2.67E-41	71.277	96.90721649
P_KWMTBOMO01927	sp|P21894|SYAC_BOMMO	Alanine--tRNA ligase, cytoplasmic OS=Bombyx mori OX=7091 PE=1 SV=1	0	99.276	100	sp|P21894|SYAC_BOMMO	Alanine--tRNA ligase, cytoplasmic OS=Bombyx mori OX=7091 PE=1 SV=1	0	99.276	100
P_KWMTBOMO02061	tr|Q1HPU7|Q1HPU7_BOMMO	Adenosylhomocysteinase OS=Bombyx mori OX=7091 PE=2 SV=1	0	100	100	sp|O76757|SAHH_ANOGA	Adenosylhomocysteinase OS=Anopheles gambiae OX=7165 GN=Ahcy13 PE=2 SV=2	0	80.233	100
P_KWMTBOMO02081	tr|Q1HPW2|Q1HPW2_BOMMO	RNA helicase OS=Bombyx mori OX=7091 GN=692781 PE=2 SV=1	0	100	100	sp|Q02748|IF4A_DROME	Eukaryotic initiation factor 4A OS=Drosophila melanogaster OX=7227 GN=eIF4A PE=1 SV=3	0	79.949	93.80952381
P_KWMTBOMO02096	tr|H9J0Z1|H9J0Z1_BOMMO	Uncharacterized protein OS=Bombyx mori OX=7091 GN=101739980 PE=4 SV=1	0	100	100	sp|Q01650|LAT1_HUMAN	Large neutral amino acids transporter small subunit 1 OS=Homo sapiens OX=9606 GN=SLC7A5 PE=1 SV=2	2.52E-159	50.734	95.97585513
P_KWMTBOMO02185	tr|Q2F5X2|Q2F5X2_BOMMO	Proteasome subunit alpha type OS=Bombyx mori OX=7091 GN=692896 PE=2 SV=1	0	100	100	sp|P22769|PSA71_DROME	Proteasome subunit alpha type-7-1 OS=Drosophila melanogaster OX=7227 GN=Prosalpha4 PE=1 SV=2	2.50E-145	81.818	92.77108434
P_KWMTBOMO02223	tr|Q1HQC7|Q1HQC7_BOMMO	Aspartate aminotransferase OS=Bombyx mori OX=7091 PE=2 SV=1	0	99.768	100	sp|P00507|AATM_RAT	Aspartate aminotransferase, mitochondrial OS=Rattus norvegicus OX=10116 GN=Got2 PE=1 SV=2	0	62.06	92.34338747
P_KWMTBOMO02431	tr|Q1HQ25|Q1HQ25_BOMMO	ATP synthase subunit OS=Bombyx mori OX=7091 GN=732934 PE=2 SV=1	2.04E-70	100	100	sp|Q5RFH0|ATP5L_PONAB	ATP synthase subunit g, mitochondrial OS=Pongo abelii OX=9601 GN=ATP5MG PE=3 SV=1	1.02E-32	52.632	95.95959596
P_KWMTBOMO02470	tr|H9IZ85|H9IZ85_BOMMO	Dolichyl-diphosphooligosaccharide--protein glycosyltransferase subunit 1 OS=Bombyx mori OX=7091 PE=3 SV=1	0	100	98.93162393	sp|E2RQ08|RPN1_CANLF	Dolichyl-diphosphooligosaccharide--protein glycosyltransferase subunit 1 OS=Canis lupus familiaris OX=9615 GN=RPN1 PE=1 SV=1	6.16E-166	53.811	92.52136752

P_KWMTBOMO02835	tr|H9J2D4|H9J2D4_BOMMO	RPN13_C domain-containing protein OS=Bombyx mori OX=7091 PE=3 SV=1	0	97.136	100	sp|Q6NZ09|ADRM1_DANRE	Proteasomal ubiquitin receptor ADRM1 OS=Danio rerio OX=7955 GN=adrm1b PE=1 SV=1	1.84E-92	45.524	96.06879607
P_KWMTBOMO03067	tr|H9J8B0|H9J8B0_BOMMO	Tripeptidyl-peptidase 2 OS=Bombyx mori OX=7091 GN=101742969 PE=4 SV=1	0	100	100	sp|P29144|TPP2_HUMAN	Tripeptidyl-peptidase 2 OS=Homo sapiens OX=9606 GN=TPP2 PE=1 SV=4	0	41.24	100
P_KWMTBOMO03173	tr|Q1HPV7|Q1HPV7_BOMMO	Histone H2A OS=Bombyx mori OX=7091 GN=h2a.z PE=2 SV=1	1.20E-88	100	100	sp|Q6Y237|H2AV_PAGMA	Histone H2A.V OS=Pagrus major OX=143350 GN=h2az2 PE=2 SV=3	3.37E-81	97.561	95.34883721
P_KWMTBOMO03177	tr|H9JV99|H9JV99_BOMMO	BRO1 domain-containing protein OS=Bombyx mori OX=7091 PE=4 SV=1	0	97.718	100	sp|Q9QZA2|PDC6I_RAT	Programmed cell death 6-interacting protein OS=Rattus norvegicus OX=10116 GN=Pdcd6ip PE=1 SV=2	0	43.273	98.36289222
P_KWMTBOMO03314	tr|Q5UAS3|Q5UAS3_BOMMO	Ribosomal protein L15 OS=Bombyx mori OX=7091 GN=RpL15 PE=2 SV=1	2.87E-148	100	100	sp|P30736|RL15_CHITE	60S ribosomal protein L15 OS=Chironomus tentans OX=7153 GN=RpL15 PE=3 SV=3	3.13E-122	80.392	100
P_KWMTBOMO03430	tr|Q2F5R4|Q2F5R4_BOMMO	Glutamate dehydrogenase (NAD(P)(+)) OS=Bombyx mori OX=7091 GN=692939 PE=2 SV=1	0	100	74.38692098	sp|P54385|DHE3_DROME	Glutamate dehydrogenase, mitochondrial OS=Drosophila melanogaster OX=7227 GN=Gdh PE=1 SV=2	0	79.821	76.29427793
P_KWMTBOMO03626	tr|H9J9S0|H9J9S0_BOMMO	CCT-alpha OS=Bombyx mori OX=7091 GN=101742382 PE=3 SV=1	0	100	100	sp|P12613|TCPA_DROME	T-complex protein 1 subunit alpha OS=Drosophila melanogaster OX=7227 GN=CCT1 PE=2 SV=2	0	80.576	100
P_KWMTBOMO03630	tr|H9J9R7|H9J9R7_BOMMO	Uncharacterized protein OS=Bombyx mori OX=7091 PE=4 SV=1	2.53E-122	74.359	44.17475728	sp|Q7TMK9|HNRPQ_MOUSE	Heterogeneous nuclear ribonucleoprotein Q OS=Mus musculus OX=10090 GN=Syncrip PE=1 SV=2	1.08E-179	55.031	78.802589
P_KWMTBOMO03860	tr|H9JKX7|H9JKX7_BOMMO	Uncharacterized protein OS=Bombyx mori OX=7091 PE=3 SV=1	0	97.603	100	sp|P26686|SRR55_DROME	Serine-arginine protein 55 OS=Drosophila melanogaster OX=7227 GN=B52 PE=1 SV=4	6.44E-97	77.487	66.78321678
P_KWMTBOMO04168	tr|H9JGM3|H9JGM3_BOMMO	Uncharacterized protein OS=Bombyx mori OX=7091 PE=3 SV=1	0	100	100	sp|Q94516|AT5F1_DROME	ATP synthase subunit b, mitochondrial OS=Drosophila melanogaster OX=7227 GN=ATPsynB PE=2 SV=2	1.44E-101	69.036	81.06995885
P_KWMTBOMO04370	tr|H9J6V7|H9J6V7_BOMMO	OBG-type G domain-containing protein OS=Bombyx mori OX=7091 PE=4 SV=1	2.07E-172	99.156	64.57765668	sp|P32234|128UP_DROME	GTP-binding protein 128up OS=Drosophila melanogaster OX=7227 GN=128up PE=2 SV=2	0	89.946	100
P_KWMTBOMO04502	tr|Q5TLD3|Q5TLD3_BOMMO	Replication protein A subunit OS=Bombyx mori OX=7091 GN=BmRPA1 PE=2 SV=1	0	99.498	100	sp|Q24492|RFA1_DROME	Replication protein A 70 kDa DNA-binding subunit OS=Drosophila melanogaster OX=7227 GN=RpA-70 PE=1 SV=1	0	47.421	100
P_KWMTBOMO04605	tr|H9J7K1|H9J7K1_BOMMO	2-phospho-D-glycerate hydro-lyase OS=Bombyx mori OX=7091 PE=3 SV=1	0	100	100	sp|P15007|ENO_DROME	Enolase OS=Drosophila melanogaster OX=7227 GN=Eno PE=1 SV=2	0	81.481	99.76905312
P_KWMTBOMO04610	tr|H9J723|H9J723_BOMMO	26S proteasome non-ATPase regulatory subunit 2 OS=Bombyx mori OX=7091 PE=3 SV=1	0	97.303	100	sp|Q5R9I6|PSMD2_PONAB	26S proteasome non-ATPase regulatory subunit 2 OS=Pongo abelii OX=9601 GN=PSMD2 PE=2 SV=1	0	55.556	98.05825243
P_KWMTBOMO04806	tr|H9JK60|H9JK60_BOMMO	Ubiquitin-activating enzyme E1 OS=Bombyx mori OX=7091 GN=101739389 PE=3 SV=1	0	99.904	100	sp|Q5U300|UBA1_RAT	Ubiquitin-like modifier-activating enzyme 1 OS=Rattus norvegicus OX=10116 GN=Uba1 PE=1 SV=1	0	65.034	97.8906999
P_KWMTBOMO05205	tr|H9IYX7|H9IYX7_BOMMO	5-aminoimidazole-4-carboxamide ribonucleotide formyltransferase OS=Bombyx mori OX=7091 PE=3 SV=1	0	100	98.81556684	sp|P31335|PUR9_CHICK	Bifunctional purine biosynthesis protein ATIC OS=Gallus gallus OX=9031 GN=ATIC PE=1 SV=1	0	68.855	100
P_KWMTBOMO05541	tr|C7AQP4|C7AQP4_BOMMO	Calcium-transporting ATPase OS=Bombyx mori OX=7091 PE=2 SV=1	0	99.301	100	sp|Q7PPA5|ATC1_ANOGA	Calcium-transporting ATPase sarcoplasmic/endoplasmic reticulum type OS=Anopheles gambiae OX=7165 GN=SERCA PE=3 SV=5	0	89.279	99.6007984
P_KWMTBOMO05564	tr|Q2F6A6|Q2F6A6_BOMMO	Elongation factor Tu OS=Bombyx mori OX=7091 PE=2 SV=1	0	100	100	sp|P49411|EFTU_HUMAN	Elongation factor Tu, mitochondrial OS=Homo sapiens OX=9606 GN=TUFM PE=1 SV=2	0	65.672	86.4516129
P_KWMTBOMO05566	tr|Q2F5M1|Q2F5M1_BOMMO	GTP-binding nuclear protein OS=Bombyx mori OX=7091 GN=692970 PE=2 SV=1	1.62E-162	100	100	sp|Q9VZ23|RAN_DROME	GTP-binding nuclear protein Ran OS=Drosophila melanogaster OX=7227 GN=Ran PE=1 SV=1	2.67E-147	91.429	98.5915493

P_KWMTBO MO05612	tr|H9JB77|H9JB 77_BOMMO	SAM_MT_RSMB_NOP domain-containing protein OS=Bombyx mori OX=7091 GN=101741314 PE=3 SV=1	0	99.874	100	sp|Q922K7|NOP 2_MOUSE	Probable 28S rRNA (cytosine-C(5))-methyltransferase OS=Mus musculus OX=10090 GN=Nop2 PE=1 SV=1	0	53.565	72.69279393
P_KWMTBO MO05687	tr|A8R081|A8R0 81_BOMMO	Bombyx homolog of P-element somatic inhibitor OS=Bombyx mori OX=7091 GN=BmPSI PE=2 SV=1	1.37E-12	23.754	27.52219532	sp|Q00341|VIGL N_HUMAN	Vigilin OS=Homo sapiens OX=9606 GN=HDLBP PE=1 SV=2	0	49.197	100
P_KWMTBO MO05723	tr|Q5UAM7|Q5U AM7_BOMMO	Ribosomal protein S15A OS=Bombyx mori OX=7091 GN=RpS15A PE=2 SV=1	5.26E-94	100	100	sp|Q6XIM8|RS1 5A_DROYA	40S ribosomal protein S15a OS=Drosophila yakuba OX=7245 GN=RpS15Aa PE=2 SV=3	3.84E-87	93.077	100
P_KWMTBO MO05741	tr|A1E129|A1E1 29_BOMMO	Kinesin-like protein OS=Bombyx mori OX=7091 PE=3 SV=2	0	99.17	100	sp|P17210|KINH _DROME	Kinesin heavy chain OS=Drosophila melanogaster OX=7227 GN=Khc PE=1 SV=2	0	74.871	100
P_KWMTBO MO05975	tr|H9J059|H9J05 9_BOMMO	Tr-type G domain-containing protein OS=Bombyx mori OX=7091 PE=4 SV=1	0	100	100	sp|Q5R8Q7|GTP B1_PONAB	GTP-binding protein 1 (Fragment) OS=Pongo abelii OX=9601 GN=GTPBP1 PE=2 SV=2	2.41E-105	74.641	75.72463768
P_KWMTBO MO05979	tr|Q5UAT0|Q5U AT0_BOMMO	Ribosomal protein L10 OS=Bombyx mori OX=7091 GN=RpL10 PE=2 SV=1	7.89E-166	100	100	sp|O96647|RL10 _BOMMA	60S ribosomal protein L10 OS=Bombyx mandarina OX=7092 GN=RpL10 PE=2 SV=1	2.91E-164	99.543	100
P_KWMTBO MO05987	tr|A0FDQ5|A0F DQ5_BOMMO	Dolichyl-diphosphooligosaccharide--protein glycosyltransferase 48 kDa subunit OS=Bombyx mori OX=7091 PE=2 SV=1	0	99.541	100	sp|Q24319|OST4 8_DROME	Dolichyl-diphosphooligosaccharide--protein glycosyltransferase 48 kDa subunit OS=Drosophila melanogaster OX=7227 GN=Ost48 PE=2 SV=2	0	65.707	95.64220183
P_KWMTBO MO06082	tr|H9IWS5|H9I WS5_BOMMO	Uncharacterized protein OS=Bombyx mori OX=7091 GN=101739385 PE=4 SV=1	0	100	100	--	--	--	--	--
P_KWMTBO MO06231	tr|H9IX18|H9IX 18_BOMMO	RuvB-like helicase OS=Bombyx mori OX=7091 PE=3 SV=1	1.12E-166	98.765	65.14745308	sp|Q9DE27|RUV B2_XENLA	RuvB-like 2 OS=Xenopus laevis OX=8355 GN=ruvbl2 PE=2 SV=1	0	81.501	100
P_KWMTBO MO06480	sp|Q5UAS1|RL1 8_BOMMO	60S ribosomal protein L18 OS=Bombyx mori OX=7091 GN=RpL18 PE=2 SV=1	1.41E-132	100	100	sp|Q5UAS1|RL1 8_BOMMO	60S ribosomal protein L18 OS=Bombyx mori OX=7091 GN=RpL18 PE=2 SV=1	9.16E-132	100	100
P_KWMTBO MO06632	tr|H9JQ89|H9JQ 89_BOMMO	Nucleoprotein TPR OS=Bombyx mori OX=7091 PE=3 SV=1	0	87.867	58.41986456	sp|A1Z8P9|TPR_ DROME	Nucleoprotein TPR OS=Drosophila melanogaster OX=7227 GN=Mtor PE=1 SV=1	0	31.858	100
P_KWMTBO MO06770	tr|H9JRG2|H9JR G2_BOMMO	Carboxylic ester hydrolase OS=Bombyx mori OX=7091 PE=3 SV=1	0	100	89.4934334	sp|P35502|ESTF _MYZPE	Esterase FE4 OS=Myzus persicae OX=13164 PE=1 SV=1	8.36E-86	35.577	97.56097561
P_KWMTBO MO06974	tr|Q5UAQ1|Q5U AQ1_BOMMO	Ribosomal protein L36A OS=Bombyx mori OX=7091 GN=RpL36A PE=2 SV=1	2.04E-71	100	100	sp|Q9NB33|RL4 4_OCHTR	60S ribosomal protein L44 OS=Ochlerotatus triseriatus OX=7162 GN=RpL44 PE=3 SV=3	5.04E-61	85.577	100
P_KWMTBO MO07274	tr|A0A4P9D450|A0A4P9D450_B OMMO	Piwi OS=Bombyx mori OX=7091 PE=2 SV=1	0	100	100	sp|A8D8P8|SIWI _BOMMO	Piwi-like protein Siwi OS=Bombyx mori OX=7091 GN=Siwi PE=1 SV=1	0	100	100
P_KWMTBO MO07294	tr|H9JM82|H9JM 82_BOMMO	Uncharacterized protein OS=Bombyx mori OX=7091 GN=101739074 PE=4 SV=1	0	99.634	100	sp|O94826|TOM 70_HUMAN	Mitochondrial import receptor subunit TOM70 OS=Homo sapiens OX=9606 GN=TOMM70 PE=1 SV=1	1.26E-176	44.718	100
P_KWMTBO MO07323	tr|Q2F6C4|Q2F6 C4_BOMMO	T-complex protein 1 subunit delta OS=Bombyx mori OX=7091 PE=2 SV=1	0	99.814	100	sp|Q9NB32|TCP D_OCHTR	T-complex protein 1 subunit delta OS=Ochlerotatus triseriatus OX=7162 PE=2 SV=1	0	69.088	100
P_KWMTBO MO07373	tr|H9J6N5|H9J6 N5_BOMMO	Uncharacterized protein OS=Bombyx mori OX=7091 PE=4 SV=1	4.18E-97	100	100	sp|Q9U1N0|HRP 65_CHITE	Hrp65 protein OS=Chironomus tentans OX=7153 GN=HRP65 PE=1 SV=1	6.32E-24	60.227	61.97183099
P_KWMTBO MO07374	tr|Q0ZAL3|Q0Z AL3_BOMMO	Splicing factor proline-and glutamine-rich OS=Bombyx mori OX=7091 GN=SFPQ PE=2 SV=1	0	99.802	100	sp|Q9GRW7|NO NA_DROVI	Protein no-on-transient A OS=Drosophila virilis OX=7244 GN=nonA PE=2 SV=1	5.73E-114	61.132	52.47524752
P_KWMTBO MO07589	tr|A0A023SG69|A0A023SG69_B OMMO	Coatomer subunit beta OS=Bombyx mori OX=7091 GN=beta-COP PE=4 SV=1	0	93.705	100	sp|Q5ZIA5|COP B_CHICK	Coatomer subunit beta OS=Gallus gallus OX=9031 GN=COPB1 PE=2 SV=1	0	57.39	100
P_KWMTBO MO07688	tr|Q5UAP3|Q5U AP3_BOMMO	40S ribosomal protein S2 OS=Bombyx mori OX=7091 GN=RpS2 PE=2 SV=1	0	100	100	sp|P31009|RS2_ DROME	40S ribosomal protein S2 OS=Drosophila melanogaster OX=7227 GN=RpS2 PE=1 SV=2	4.18E-144	90.278	82.75862069
P_KWMTBO MO07772	tr|Q5UAN4|Q5U AN4_BOMMO	Ribosomal protein S10 OS=Bombyx mori OX=7091 GN=RpS10 PE=2 SV=1	4.40E-118	100	100	sp|Q962R9|RS10 _SPOFR	40S ribosomal protein S10 OS=Spodoptera frugiperda OX=7108 GN=RpS10 PE=2 SV=1	1.19E-105	92.453	99.375

P_KWMTBOMO07857	tr|Q5R1P5|Q5R1P5_BOMMO	Heat shock protein hsp21.4 OS=Bombyx mori OX=7091 GN=Hsp21.4 PE=2 SV=1	4.58E-139	100	100	sp|Q00649|HSPB1_CHICK	Heat shock protein beta-1 OS=Gallus gallus OX=9031 GN=HSPB1 PE=2 SV=1	5.62E-19	47.727	47.05882353
P_KWMTBOMO07874	tr|H9IVB4|H9IVB4_BOMMO	Glutaminyl-tRNA synthetase OS=Bombyx mori OX=7091 PE=3 SV=1	0	99.788	60.51282051	sp|Q9Y105|SYQ_DROME	Probable glutamine--tRNA ligase OS=Drosophila melanogaster OX=7227 GN=GlnRS PE=2 SV=1	0	68.408	100
P_KWMTBOMO07930	tr|Q1HQ48|Q1HQ48_BOMMO	Mitochondrial single-stranded DNA-binding protein OS=Bombyx mori OX=7091 PE=2 SV=1	1.39E-108	99.324	100	sp|P54622|SSBP_DROME	Single-stranded DNA-binding protein, mitochondrial OS=Drosophila melanogaster OX=7227 GN=mtSSB PE=1 SV=2	2.56E-50	64.545	74.32432432
P_KWMTBOMO08041	tr|H9IV46|H9IV46_BOMMO	Rab proteins geranylgeranyltransferase component A OS=Bombyx mori OX=7091 GN=101740100 PE=3 SV=1	0	100	100	sp|Q9V8W3|RABEP_DROME	Rab proteins geranylgeranyltransferase component A OS=Drosophila melanogaster OX=7227 GN=Rep PE=1 SV=1	6.05E-105	36.542	97.32313576
P_KWMTBOMO08100	tr|Q5UAR8|Q5UAR8_BOMMO	60S ribosomal protein L21 OS=Bombyx mori OX=7091 GN=RpL21 PE=2 SV=1	1.42E-115	100	100	sp|P46778|RL21_HUMAN	60S ribosomal protein L21 OS=Homo sapiens OX=9606 GN=RPL21 PE=1 SV=2	2.69E-77	66.875	100
P_KWMTBOMO08106	tr|H9IUY0|H9IUY0_BOMMO	Clathrin light chain OS=Bombyx mori OX=7091 PE=3 SV=1	2.92E-149	98.095	100	sp|Q9VWA1|CLC_DROME	Clathrin light chain OS=Drosophila melanogaster OX=7227 GN=Clc PE=2 SV=1	5.76E-40	46.948	100
P_KWMTBOMO08193	tr|H9JIX2|H9JIX2_BOMMO	Uncharacterized protein OS=Bombyx mori OX=7091 PE=3 SV=1	2.11E-91	98.571	100	sp|O95831|AIFM1_HUMAN	Apoptosis-inducing factor 1, mitochondrial OS=Homo sapiens OX=9606 GN=AIFM1 PE=1 SV=1	4.02E-30	42.958	100
P_KWMTBOMO08310	tr|Q1HQB9|Q1HQB9_BOMMO	Nucleosome assembly protein isoform 1 OS=Bombyx mori OX=7091 PE=2 SV=1	0	100	100	sp|Q5U2Z3|NP1L4_RAT	Nucleosome assembly protein 1-like 4 OS=Rattus norvegicus OX=10116 GN=Nap1l4 PE=1 SV=1	4.23E-97	49.462	94.17721519
P_KWMTBOMO08323	tr|H9JI91|H9JI91_BOMMO	Uncharacterized protein OS=Bombyx mori OX=7091 PE=4 SV=1	0	99.643	98.24561404	sp|Q5R4J9|NAA15_PONAB	N-alpha-acetyltransferase 15, NatA auxiliary subunit OS=Pongo abelii OX=9601 GN=NAA15 PE=2 SV=1	4.44E-47	41.985	91.92982456
P_KWMTBOMO08399	tr|Q5UAR0|Q5UAR0_BOMMO	60S ribosomal protein L28 OS=Bombyx mori OX=7091 GN=RpL28 PE=2 SV=1	1.61E-98	100	100	sp|Q962T2|RL28_SPOFR	60S ribosomal protein L28 OS=Spodoptera frugiperda OX=7108 GN=RpL28 PE=2 SV=1	7.15E-81	92.857	90.64748201
P_KWMTBOMO08440	tr|Q5UAM6|Q5UAM6_BOMMO	Ribosomal protein S16 OS=Bombyx mori OX=7091 GN=RpS16 PE=2 SV=1	8.57E-109	100	100	sp|Q95V31|RS16_SPOFR	40S ribosomal protein S16 OS=Spodoptera frugiperda OX=7108 GN=RpS16 PE=2 SV=1	1.88E-104	98.013	100
P_KWMTBOMO08607	tr|H9JEL8|H9JEL8_BOMMO	Uncharacterized protein OS=Bombyx mori OX=7091 PE=4 SV=1	2.44E-142	98.974	54.31754875	sp|Q5M9F1|RBM34_RAT	RNA-binding protein 34 OS=Rattus norvegicus OX=10116 GN=Rbm34 PE=1 SV=1	8.14E-07	26.087	64.06685237
P_KWMTBOMO08642	tr|F8UN44|F8UN44_BOMMO	Heat shock protein 70-3 OS=Bombyx mori OX=7091 PE=2 SV=1	0	100	100	sp|P29844|BIP_DROME	Endoplasmic reticulum chaperone BiP OS=Drosophila melanogaster OX=7227 GN=Hsc70-3 PE=1 SV=2	0	89.708	99.38931298
P_KWMTBOMO08648	tr|H9JEK1|H9JEK1_BOMMO	Uncharacterized protein OS=Bombyx mori OX=7091 PE=3 SV=1	0	91.125	100	sp|P13395|SPTCA_DROME	Spectrin alpha chain OS=Drosophila melanogaster OX=7227 GN=alpha-Spec PE=1 SV=2	0	76.124	100
P_KWMTBOMO08666	tr|Q66SV4|Q66SV4_BOMMO	40S ribosomal protein S23 OS=Bombyx mori OX=7091 GN=S23 PE=2 SV=1	5.48E-103	100	100	sp|Q6EV23|RS23_PAPDA	40S ribosomal protein S23 OS=Papilio dardanus OX=77259 GN=RpS23 PE=2 SV=1	6.27E-101	97.902	100
P_KWMTBOMO08776	sp|Q1HPW4|EIF3I_BOMMO	Eukaryotic translation initiation factor 3 subunit I OS=Bombyx mori OX=7091 PE=2 SV=1	0	100	100	sp|Q1HPW4|EIF3I_BOMMO	Eukaryotic translation initiation factor 3 subunit I OS=Bombyx mori OX=7091 PE=2 SV=1	0	100	100
P_KWMTBOMO08820	tr|Q1HPI5|Q1HPI5_BOMMO	Nucleoside diphosphate kinase OS=Bombyx mori OX=7091 PE=2 SV=1	4.74E-113	100	100	sp|P08879|NDKA_DROME	Nucleoside diphosphate kinase OS=Drosophila melanogaster OX=7227 GN=awd PE=1 SV=3	5.17E-94	81.046	100
P_KWMTBOMO08893	tr|H9JE77|H9JE77_BOMMO	Succinate--CoA ligase [GDP-forming] subunit beta, mitochondrial OS=Bombyx mori OX=7091 GN=101740061 PE=3 SV=1	0	100	100	sp|Q96I99|SUCB2_HUMAN	Succinate--CoA ligase [GDP-forming] subunit beta, mitochondrial OS=Homo sapiens OX=9606 GN=SUCLG2 PE=1 SV=2	1.11E-159	59.231	92.63657957
P_KWMTBOMO08956	tr|H9JDY1|H9JDY1_BOMMO	M20_dimer domain-containing protein OS=Bombyx mori OX=7091 PE=3 SV=1	0	99.784	96.86847599	sp|Q3ZC84|CNDP2_BOVIN	Cytosolic non-specific dipeptidase OS=Bos taurus OX=9913 GN=CNDP2 PE=2 SV=1	0	58.65	98.95615866

P_KWMTBOMO09054	tr|Q1HQ47|Q1HQ47_BOMMO	Isocitrate dehydrogenase [NADP] OS=Bombyx mori OX=7091 GN=100101164 PE=2 SV=1	0	100	100	sp|P33198|IDHP_PIG	Isocitrate dehydrogenase [NADP], mitochondrial (Fragment) OS=Sus scrofa OX=9823 GN=IDH2 PE=1 SV=1	0	71.325	95.40229885
P_KWMTBOMO09055	tr|H9JDA5|H9JDA5_BOMMO	Proton-translocating NAD(P)(+) transhydrogenase OS=Bombyx mori OX=7091 PE=3 SV=1	0	93.082	100	sp|P11024|NNTM_BOVIN	NAD(P) transhydrogenase, mitochondrial OS=Bos taurus OX=9913 GN=NNT PE=1 SV=3	0	59.925	100
P_KWMTBOMO09230	tr|H9J1M5|H9J1M5_BOMMO	Uncharacterized protein OS=Bombyx mori OX=7091 PE=3 SV=1	3.45E-75	100	100	sp|P08570|RLA1_DROME	60S acidic ribosomal protein P1 OS=Drosophila melanogaster OX=7227 GN=RpLP1 PE=1 SV=2	1.01E-42	81.25	100
P_KWMTBOMO09300	tr|Q5UAU1|Q5UAU1_BOMMO	60S acidic ribosomal protein P0 OS=Bombyx mori OX=7091 GN=RpP0 PE=1 SV=1	0	100	100	sp|Q9U3U0|RLA0_CERCA	60S acidic ribosomal protein P0 OS=Ceratitis capitata OX=7213 GN=RpLP0 PE=3 SV=1	1.73E-177	81.388	100
P_KWMTBOMO09415	tr|H9JTB0|H9JTB0_BOMMO	RNA helicase OS=Bombyx mori OX=7091 GN=101741115 PE=4 SV=1	0	99.765	100	sp|Q27268|DX39B_DROME	ATP-dependent RNA helicase WM6 OS=Drosophila melanogaster OX=7227 GN=Hel25E PE=1 SV=1	0	88.498	100
P_KWMTBOMO09442	tr|Q5UAT5|Q5UAT5_BOMMO	60S ribosomal protein L6 OS=Bombyx mori OX=7091 GN=RpL6 PE=2 SV=1	0	99.634	100	sp|Q6QMZ4|RL6_CHILA	60S ribosomal protein L6 OS=Chinchilla lanigera OX=34839 GN=RPL6 PE=2 SV=3	4.24E-65	48.8	91.57509158
P_KWMTBOMO09542	tr|H9JU13|H9JU13_BOMMO	Eukaryotic translation initiation factor 3 subunit A OS=Bombyx mori OX=7091 PE=3 SV=1	0	96.154	90.33898305	sp|Q173M7|EIF3A_AEDAE	Eukaryotic translation initiation factor 3 subunit A OS=Aedes aegypti OX=7159 GN=eIF3-S10 PE=3 SV=1	0	66.215	68.72881356
P_KWMTBOMO09621	tr|H9JTI9|H9JTI9_BOMMO	Eukaryotic translation initiation factor 3 subunit C OS=Bombyx mori OX=7091 GN=eIF3c PE=3 SV=1	0	100	100	sp|Q0ZB76|EIF3C_BOMMO	Eukaryotic translation initiation factor 3 subunit C OS=Bombyx mori OX=7091 GN=eIF3-S8 PE=2 SV=1	0	99.886	100
P_KWMTBOMO09747	tr|B7XFU6|B7XFU6_BOMMO	Clathrin heavy chain OS=Bombyx mori OX=7091 GN=100233163 PE=2 SV=1	0	99.941	100	sp|P29742|CLH_DROME	Clathrin heavy chain OS=Drosophila melanogaster OX=7227 GN=Chc PE=1 SV=1	0	85.196	100
P_KWMTBOMO09808	tr|Q2F6C3|Q2F6C3_BOMMO	Chaperonin subunit 6a zeta OS=Bombyx mori OX=7091 GN=692796 PE=2 SV=1	0	100	100	sp|P80317|TCPZ_MOUSE	T-complex protein 1 subunit zeta OS=Mus musculus OX=10090 GN=Cct6a PE=1 SV=3	0	72.505	100
P_KWMTBOMO10248	tr|H9JCR4|H9JCR4_BOMMO	40S ribosomal protein SA OS=Bombyx mori OX=7091 PE=3 SV=1	0	100	100	sp|Q5UAP4|RSSA_BOMMO	40S ribosomal protein SA OS=Bombyx mori OX=7091 PE=2 SV=1	0	99.673	100
P_KWMTBOMO10358	tr|H9JX26|H9JX26_BOMMO	DUF4781 domain-containing protein OS=Bombyx mori OX=7091 PE=4 SV=1	0	79.827	48.32869081	--	--	--	--	--
P_KWMTBOMO10661	tr|H9JFF9|H9JFF9_BOMMO	Uncharacterized protein OS=Bombyx mori OX=7091 PE=4 SV=1	0	99.829	69.84505364	sp|B4J9K1|BOP1_DROGR	Ribosome biogenesis protein BOP1 homolog OS=Drosophila grimshawi OX=7222 GN=GH22027 PE=3 SV=1	0	57.698	76.63885578
P_KWMTBOMO10780	tr|H9JFX9|H9JFX9_BOMMO	Uncharacterized protein OS=Bombyx mori OX=7091 PE=4 SV=1	0	90.138	43.81909548	sp|Q9V3P6|PSMD1_DROME	26S proteasome non-ATPase regulatory subunit 1 OS=Drosophila melanogaster OX=7227 GN=Rpn2 PE=1 SV=1	0	71.218	100
P_KWMTBOMO11045	tr|H9JRL1|H9JRL1_BOMMO	Uncharacterized protein OS=Bombyx mori OX=7091 PE=3 SV=1	0	28.363	91.51958674	sp|Q00963|SPTCB_DROME	Spectrin beta chain OS=Drosophila melanogaster OX=7227 GN=beta-Spec PE=1 SV=2	0	73.055	100
P_KWMTBOMO11061	tr|Q2F5N1|Q2F5N1_BOMMO	Prohibitin OS=Bombyx mori OX=7091 GN=693108 PE=2 SV=1	0	100	100	sp|Q5XIH7|PHB2_RAT	Prohibitin-2 OS=Rattus norvegicus OX=10116 GN=Phb2 PE=1 SV=1	5.66E-158	72.667	100
P_KWMTBOMO11083	tr|Q9BPS3|Q9BPS3_BOMMO	Elongation factor 1 gamma OS=Bombyx mori OX=7091 GN=ef-1g PE=2 SV=1	0	100	100	sp|P12261|EF1G_ARTSA	Elongation factor 1-gamma OS=Artemia salina OX=85549 PE=1 SV=3	0	65.741	100
P_KWMTBOMO11096	tr|A1E9B3|A1E9B3_BOMMO	H(+)-transporting two-sector ATPase OS=Bombyx mori OX=7091 PE=2 SV=1	0	99.676	100	sp|P31400|VATA_MANSE	V-type proton ATPase catalytic subunit A OS=Manduca sexta OX=7130 GN=VHAA PE=2 SV=1	0	98.217	100
P_KWMTBOMO11202	tr|H9JT03|H9JT03_BOMMO	F-actin-capping protein subunit alpha OS=Bombyx mori OX=7091 GN=101738938 PE=3 SV=1	0	100	100	sp|Q9W2N0|CAPZA_DROME	F-actin-capping protein subunit alpha OS=Drosophila melanogaster OX=7227 GN=cpa PE=2 SV=1	1.31E-147	68.44	97.91666667
P_KWMTBOMO11255	tr|Q5UAN5|Q5UAN5_BOMMO	40S ribosomal protein S9 OS=Bombyx mori OX=7091 GN=RpS9 PE=2 SV=1	5.62E-140	100	100	sp|B4PEU8|RS9_DROYA	40S ribosomal protein S9 OS=Drosophila yakuba OX=7245 GN=RpS9 PE=2 SV=1	2.83E-117	89.231	100

P_KWMTBOMO11336	tr|H9IX66|H9IX66_BOMMO	Threonyl-tRNA synthetase OS=Bombyx mori OX=7091 PE=3 SV=1	0	98.151	98.59747546	sp|Q3ZBV8|SYTC_BOVIN	Threonine--tRNA ligase 1, cytoplasmic OS=Bos taurus OX=9913 GN=TARS1 PE=2 SV=1	0	69.371	100
P_KWMTBOMO11448	tr|H9IXE5|H9IXE5_BOMMO	Uncharacterized protein OS=Bombyx mori OX=7091 GN=733057 PE=4 SV=1	1.36E-160	100	100	sp|P53994|RAB2A_MOUSE	Ras-related protein Rab-2A OS=Mus musculus OX=10090 GN=Rab2a PE=1 SV=1	2.20E-141	90.654	100
P_KWMTBOMO11590	tr|H9J3D2|H9J3D2_BOMMO	Ku domain-containing protein OS=Bombyx mori OX=7091 GN=101736121 PE=4 SV=1	0	98.077	80.68965517	sp|O93257|XRCC6_CHICK	X-ray repair cross-complementing protein 5 OS=Gallus gallus OX=9031 GN=XRCC6 PE=2 SV=1	5.59E-42	26.126	95.68965517
P_KWMTBOMO11666	tr|Q2V0H5|Q2V0H5_BOMMO	Vesicle-fusing ATPase OS=Bombyx mori OX=7091 GN=ter94 PE=2 SV=1	0	100	100	sp|Q7KN62|TERA_DROME	Transitional endoplasmic reticulum ATPase TER94 OS=Drosophila melanogaster OX=7227 GN=TER94 PE=1 SV=1	0	88.025	100
P_KWMTBOMO11678	tr|Q5UAS7|Q5UAS7_BOMMO	60S ribosomal protein L12 OS=Bombyx mori OX=7091 GN=RpL12 PE=2 SV=1	1.46E-118	100	100	sp|P23358|RL12_RAT	60S ribosomal protein L12 OS=Rattus norvegicus OX=10116 GN=Rpl12 PE=2 SV=1	3.08E-96	81.25	97.56097561
P_KWMTBOMO11780	tr|H9JY65|H9JY65_BOMMO	Complex I-30kD OS=Bombyx mori OX=7091 GN=101738954 PE=3 SV=1	0	100	100	sp|Q9VZU4|NDUS3_DROME	NADH dehydrogenase [ubiquinone] iron-sulfur protein 3, mitochondrial OS=Drosophila melanogaster OX=7227 GN=ND-30 PE=1 SV=1	1.81E-130	74.468	88.67924528
P_KWMTBOMO11985	tr|Q5UAT4|Q5UAT4_BOMMO	Ribosomal protein L7 OS=Bombyx mori OX=7091 GN=RpL7 PE=2 SV=1	0	100	100	sp|P32100|RL7_DROME	60S ribosomal protein L7 OS=Drosophila melanogaster OX=7227 GN=RpL7 PE=1 SV=2	4.63E-117	66.667	92.56505576
P_KWMTBOMO12018	tr|Q5UAN7|Q5UAN7_BOMMO	40S ribosomal protein S7 OS=Bombyx mori OX=7091 GN=RpS7 PE=2 SV=1	1.24E-137	99.474	100	sp|P48155|RS7_MANSE	40S ribosomal protein S7 OS=Manduca sexta OX=7130 GN=RpS7 PE=2 SV=1	1.42E-134	97.368	100
P_KWMTBOMO12037	tr|H9J470|H9J470_BOMMO	DEK_C domain-containing protein OS=Bombyx mori OX=7091 PE=4 SV=1	0	91.221	94.15322581	sp|Q6AXS3|DEK_RAT	Protein DEK OS=Rattus norvegicus OX=10116 GN=Dek PE=1 SV=1	5.05E-20	47.368	19.15322581
P_KWMTBOMO12166	tr|H9JWZ4|H9JWZ4_BOMMO	NADH dehydrogenase [ubiquinone] 1 alpha subcomplex subunit 10, mitochondrial OS=Bombyx mori OX=7091 GN=101745480 PE=3 SV=1	0	100	100	sp|P91929|NDUAA_DROME	NADH dehydrogenase [ubiquinone] 1 alpha subcomplex subunit 10, mitochondrial OS=Drosophila melanogaster OX=7227 GN=ND-42 PE=1 SV=2	1.80E-134	49.485	97.48743719
P_KWMTBOMO12264	tr|H9JHZ0|H9JHZ0_BOMMO	CYTOSOL_AP domain-containing protein OS=Bombyx mori OX=7091 PE=3 SV=1	0	93.99	79.54110899	sp|Q5R7G6|PEPL1_PONAB	Probable aminopeptidase NPEPL1 OS=Pongo abelii OX=9601 GN=NPEPL1 PE=3 SV=2	0	54.981	99.80879541
P_KWMTBOMO12389	tr|H9JSC6|H9JSC6_BOMMO	Coatomer subunit epsilon OS=Bombyx mori OX=7091 PE=3 SV=1	0	100	100	sp|Q5ZIK9|COPE_CHICK	Coatomer subunit epsilon OS=Gallus gallus OX=9031 GN=COPE PE=2 SV=1	6.42E-96	48.475	97.68211921
P_KWMTBOMO12560	tr|A0A1Q1NKL1|A0A1Q1NKL1_BOMMO	Tyrosine--tRNA ligase OS=Bombyx mori OX=7091 PE=2 SV=1	0	99.809	100	sp|Q5ZJ08|SYYC_CHICK	Tyrosine--tRNA ligase, cytoplasmic OS=Gallus gallus OX=9031 GN=YARS1 PE=2 SV=1	0	71.374	100
P_KWMTBOMO12602	tr|H9IWB5|H9IWB5_BOMMO	Uncharacterized protein OS=Bombyx mori OX=7091 PE=3 SV=1	0	95.142	67.58824981	sp|P37276|DYHC_DROME	Dynein heavy chain, cytoplasmic OS=Drosophila melanogaster OX=7227 GN=Dhc64C PE=2 SV=2	0	79.96	99.77783263
P_KWMTBOMO12659	tr|H9IVX5|H9IVX5_BOMMO	T-complex protein 1 subunit gamma OS=Bombyx mori OX=7091 GN=101744569 PE=3 SV=1	0	100	100	sp|P48605|TCPG_DROME	T-complex protein 1 subunit gamma OS=Drosophila melanogaster OX=7227 GN=CCT3 PE=2 SV=2	0	81.041	99.62962963
P_KWMTBOMO12667	tr|Q1HPM7|Q1HPM7_BOMMO	Mitochondrial aldehyde dehydrogenase OS=Bombyx mori OX=7091 PE=2 SV=1	0	97.291	47.22814499	sp|P81178|ALDH2_MESAU	Aldehyde dehydrogenase, mitochondrial OS=Mesocricetus auratus OX=10036 GN=ALDH2 PE=1 SV=1	0	70	45.84221748
P_KWMTBOMO12844	tr|H9U396|H9U396_BOMMO	Endoplasmin-like protein OS=Bombyx mori OX=7091 PE=2 SV=1	0	100	100	sp|Q66HD0|ENPL_RAT	Endoplasmin OS=Rattus norvegicus OX=10116 GN=Hsp90b1 PE=1 SV=2	0	59.264	93.95061728
P_KWMTBOMO12895	tr|Q19AA9|Q19AA9_BOMMO	Transport protein Sec61 alpha subunit OS=Bombyx mori OX=7091 GN=733068 PE=2 SV=1	0	100	100	sp|Q9JLR1|S61A2_MOUSE	Protein transport protein Sec61 subunit alpha isoform 2 OS=Mus musculus OX=10090 GN=Sec61a2 PE=2 SV=3	0	91.597	100
P_KWMTBOMO13226	tr|H6VTQ9|H6VTQ9_BOMMO	DnaJ-19 OS=Bombyx mori OX=7091 PE=2 SV=1	0	100	99.29775281	sp|Q9UGP8|SEC63_HUMAN	Translocation protein SEC63 homolog OS=Homo sapiens OX=9606 GN=SEC63 PE=1 SV=2	0	44.537	100

P_KWMTBOMO13235	tr|H9JKC3|H9JKC3_BOMMO	Transket_pyr domain-containing protein OS=Bombyx mori OX=7091 PE=3 SV=1	0	83.749	100	sp|Q60HE2|ODO1_MACFA	2-oxoglutarate dehydrogenase, mitochondrial OS=Macaca fascicularis OX=9541 GN=OGDH PE=2 SV=1	0	62.761	98.6287953
P_KWMTBOMO13293	tr|E9JEI6|E9JEI6_BOMMO	Poly [ADP-ribose] polymerase (Fragment) OS=Bombyx mori OX=7091 PE=2 SV=1	0	99.894	94.5	sp|Q11208|PARP_SARPE	Poly [ADP-ribose] polymerase OS=Sarcophaga peregrina OX=7386 PE=1 SV=1	0	48.473	100
P_KWMTBOMO13369	tr|Q2F5S0|Q2F5S0_BOMMO	rRNA processing protein Ebp2 OS=Bombyx mori OX=7091 PE=2 SV=1	0	99.032	100	sp|Q9V9Z9|EBP2_DROME	Probable rRNA-processing protein EBP2 homolog OS=Drosophila melanogaster OX=7227 GN=CG1542 PE=2 SV=1	9.55E-77	50.16	100
P_KWMTBOMO13370	tr|H9JN38|H9JN38_BOMMO	Signal recognition particle subunit SRP72 OS=Bombyx mori OX=7091 PE=3 SV=1	0	100	66.30602782	sp|O76094|SRP72_HUMAN	Signal recognition particle subunit SRP72 OS=Homo sapiens OX=9606 GN=SRP72 PE=1 SV=3	7.11E-121	37.721	96.29057187
P_KWMTBOMO13455	sp|Q5UAP0|RS4_BOMMO	40S ribosomal protein S4 OS=Bombyx mori OX=7091 GN=RpS4 PE=2 SV=1	0	100	100	sp|Q5UAP0|RS4_BOMMO	40S ribosomal protein S4 OS=Bombyx mori OX=7091 GN=RpS4 PE=2 SV=1	0	100	100
P_KWMTBOMO13567	tr|H9JN68|H9JN68_BOMMO	Uncharacterized protein OS=Bombyx mori OX=7091 PE=4 SV=1	0	100	61.18326118	--	--	--	--	--
P_KWMTBOMO13569	tr|Q5UAR3|Q5UAR3_BOMMO	Ribosomal protein L26 OS=Bombyx mori OX=7091 GN=RpL26 PE=2 SV=1	7.52E-72	100	100	sp|Q95WA0|RL26_LITLI	60S ribosomal protein L26 OS=Littorina littorea OX=31216 GN=RPL26 PE=2 SV=1	1.90E-51	80.392	100
P_KWMTBOMO13960	tr|Q5UAN0|Q5UAN0_BOMMO	40S ribosomal protein S13 OS=Bombyx mori OX=7091 GN=RpS13 PE=2 SV=1	8.93E-110	100	100	sp|Q962R6|RS13_SPOFR	40S ribosomal protein S13 OS=Spodoptera frugiperda OX=7108 GN=RpS13 PE=2 SV=3	1.63E-108	99.338	100
P_KWMTBOMO14041	tr|Q2F5J1|Q2F5J1_BOMMO	26S proteasome non-ATPase regulatory subunit 13 OS=Bombyx mori OX=7091 PE=2 SV=1	0	100	100	sp|B0BN93|PSD13_RAT	26S proteasome non-ATPase regulatory subunit 13 OS=Rattus norvegicus OX=10116 GN=Psmd13 PE=1 SV=1	1.26E-102	46.921	88.57142857
P_KWMTBOMO14080	tr|H9JNW4|H9JNW4_BOMMO	Uncharacterized protein OS=Bombyx mori OX=7091 PE=4 SV=1	0	100	98.80149813	sp|P35502|ESTF_MYZPE	Esterase FE4 OS=Myzus persicae OX=13164 PE=1 SV=1	4.32E-69	33.142	39.1011236
P_KWMTBOMO14157	tr|H9JNS6|H9JNS6_BOMMO	Coatomer subunit gamma OS=Bombyx mori OX=7091 GN=692402 PE=3 SV=1	0	99.768	100	sp|Q29AE5|COPG_DROPS	Coatomer subunit gamma OS=Drosophila pseudoobscura pseudoobscura OX=46245 GN=gammaCop PE=3 SV=1	0	58.75	100
P_KWMTBOMO14420	tr|H9IS77|H9IS77_BOMMO	Uncharacterized protein OS=Bombyx mori OX=7091 GN=101744691 PE=4 SV=1	0	99.786	99.78609626	sp|Q9BUJ2|HNRL1_HUMAN	Heterogeneous nuclear ribonucleoprotein U-like protein 1 OS=Homo sapiens OX=9606 GN=HNRNPUL1 PE=1 SV=2	7.54E-52	41.87	26.31016043
P_KWMTBOMO14440	tr|Q1HPP5|Q1HPP5_BOMMO	Actin-depolymerizing factor 1 OS=Bombyx mori OX=7091 PE=2 SV=1	1.70E-108	100	100	sp|P45594|CADF_DROME	Cofilin/actin-depolymerizing factor homolog OS=Drosophila melanogaster OX=7227 GN=tsr PE=1 SV=1	2.22E-100	92.568	100
P_KWMTBOMO14479	tr|H9IS48|H9IS48_BOMMO	Uncharacterized protein OS=Bombyx mori OX=7091 GN=101742289 PE=3 SV=1	7.91E-100	100	100	sp|Q8I1F4|FBRL_DROER	rRNA 2'-O-methyltransferase fibrillarin OS=Drosophila erecta OX=7220 GN=Fib PE=3 SV=1	2.11E-41	82.278	50
P_KWMTBOMO14530	tr|Q2F5R7|Q2F5R7_BOMMO	Eukaryotic peptide chain release factor subunit 1 OS=Bombyx mori OX=7091 PE=2 SV=1	0	99.314	100	sp|Q9VPH7|ERF1_DROME	Eukaryotic peptide chain release factor subunit 1 OS=Drosophila melanogaster OX=7227 GN=eRF1 PE=1 SV=2	0	93.822	100
P_KWMTBOMO14549	tr|I6XKQ0|I6XKQ0_BOMMO	Heat shock protein 70-5 OS=Bombyx mori OX=7091 PE=2 SV=1	0	99.565	100	sp|P29845|HSP7E_DROME	Heat shock 70 kDa protein cognate 5 OS=Drosophila melanogaster OX=7227 GN=Hsc70-5 PE=1 SV=2	0	81.86	93.47826087
P_KWMTBOMO14732	tr|Q86PG2|Q86PG2_BOMMO	ADP/ATP translocase OS=Bombyx mori OX=7091 GN=ANT PE=2 SV=1	0	100	100	sp|Q26365|ADT_DROME	ADP,ATP carrier protein OS=Drosophila melanogaster OX=7227 GN=sesB PE=2 SV=4	0	87.458	98.33333333
P_KWMTBOMO14752	tr|H9J2U6|H9J2U6_BOMMO	PUA domain-containing protein OS=Bombyx mori OX=7091 GN=101744752 PE=3 SV=1	0	100	100	sp|O44081|DKC1_DROME	H/ACA ribonucleoprotein complex subunit 4 OS=Drosophila melanogaster OX=7227 GN=Nop60B PE=1 SV=1	0	76.69	91.47121535
P_KWMTBOMO14804	tr|H9JRQ1|H9JRQ1_BOMMO	Programmed cell death protein 4 OS=Bombyx mori OX=7091 GN=101743674 PE=3 SV=1	0	100	59.14221219	sp|Q98TX3|PDCD4_CHICK	Programmed cell death protein 4 OS=Gallus gallus OX=9031 GN=PDCD4 PE=2 SV=1	3.96E-92	43.371	100
P_KWMTBOMO14887	tr|H9JRM2|H9JRM2_BOMMO	T-complex protein 1 subunit eta OS=Bombyx mori OX=7091 PE=3 SV=1	0	99.631	100	sp|Q5ZJK8|TCPH_CHICK	T-complex protein 1 subunit eta OS=Gallus gallus OX=9031 GN=CCT7 PE=1 SV=1	0	69.376	97.60147601

P_KWMTBO MO14984	tr|H9J5J2|H9J5J2 _BOMMO	AAA domain-containing protein OS=Bombyx mori OX=7091 PE=3 SV=1	0	100	100	sp|O42587|PR6A A_XENLA	26S proteasome regulatory subunit 6A-A OS=Xenopus laevis OX=8355 GN=psmc3-a PE=2 SV=2	0	88.124	98.36448598
P_KWMTBO MO15060	tr|H9J5R2|H9J5 R2_BOMMO	Protein transport protein SEC23 OS=Bombyx mori OX=7091 PE=3 SV=1	0	100	100	sp|Q01405|SC23 A_MOUSE	Protein transport protein Sec23A OS=Mus musculus OX=10090 GN=Sec23a PE=1 SV=2	0	74.583	100
P_KWMTBO MO15114	tr|Q5UAL8|Q5U AL8_BOMMO	40S ribosomal protein S25 OS=Bombyx mori OX=7091 GN=RpS25 PE=2 SV=1	7.13E-83	100	100	sp|Q962Q5|RS25 _SPOFR	40S ribosomal protein S25 OS=Spodoptera frugiperda OX=7108 GN=RpS25 PE=3 SV=1	1.98E-81	99.16	100
P_KWMTBO MO15123	tr|H9J5W8|H9J5 W8_BOMMO	AAA domain-containing protein OS=Bombyx mori OX=7091 PE=3 SV=2	0	99.241	90.38901602	sp|P48601|PRS4 _DROME	26S proteasome regulatory subunit 4 OS=Drosophila melanogaster OX=7227 GN=Rpt2 PE=1 SV=2	0	96.811	100
P_KWMTBO MO15159	tr|H9J5Z1|H9J5Z 1_BOMMO	Uncharacterized protein OS=Bombyx mori OX=7091 PE=4 SV=1	0	97.685	99.34282585	sp|Q99PL5|RRB P1_MOUSE	Ribosome-binding protein 1 OS=Mus musculus OX=10090 GN=Rrbp1 PE=1 SV=2	3.36E-17	27.524	67.25082147
P_KWMTBO MO15359	tr|H9J6H4|H9J6 H4_BOMMO	Glutamyl-tRNA synthetase OS=Bombyx mori OX=7091 PE=3 SV=1	0	100	97.09944751	sp|P28668|SYEP _DROME	Bifunctional glutamate/proline--tRNA ligase OS=Drosophila melanogaster OX=7227 GN=GluProRS PE=1 SV=2	0	60.526	99.72375691
P_KWMTBO MO15361	tr|O16143|O1614 3_BOMMO	Rab1 protein OS=Bombyx mori OX=7091 GN=rabB PE=2 SV=1	5.31E-153	100	100	sp|Q05974|RAB 1A_LYMST	Ras-related protein Rab-1A OS=Lymnaea stagnalis OX=6523 GN=RAB1A PE=2 SV=1	4.74E-124	81.188	100
P_KWMTBO MO15584	tr|D2Y4R2|D2Y 4R2_BOMMO	Coatomer subunit alpha OS=Bombyx mori OX=7091 GN=COPA PE=2 SV=1	0	99.729	99.72972973	sp|P53621|COPA _HUMAN	Coatomer subunit alpha OS=Homo sapiens OX=9606 GN=COPA PE=1 SV=2	0	78.729	97.83783784
P_KWMTBO MO15715	tr|H9IYH6|H9IY H6_BOMMO	Uncharacterized protein OS=Bombyx mori OX=7091 PE=4 SV=1	0	86.272	98.32817337	--	--	--	--	--
P_KWMTBO MO15758	tr|B3VBE3|B3V BE3_BOMMO	Vacuolar proton pump subunit B OS=Bombyx mori OX=7091 PE=2 SV=1	0	99.278	88.21656051	sp|P31401|VAT B_MANSE	V-type proton ATPase subunit B OS=Manduca sexta OX=7130 GN=VHA55 PE=2 SV=1	0	97.112	88.21656051
P_KWMTBO MO16040	tr|H9J4W7|H9J4 W7_BOMMO	Uncharacterized protein OS=Bombyx mori OX=7091 PE=3 SV=1	0	100	77.56410256	sp|P42669|PURA _MOUSE	Transcriptional activator protein Pur-alpha OS=Mus musculus OX=10090 GN=Pura PE=1 SV=1	1.27E-77	50.213	75.32051282
P_KWMTBO MO16309	tr|H9JW60|H9J W60_BOMMO	UBX domain-containing protein OS=Bombyx mori OX=7091 PE=4 SV=1	1.01E-106	92.353	43.58974359	sp|Q3SZC4|NSF 1C_BOVIN	NSFL1 cofactor p47 OS=Bos taurus OX=9913 GN=NSFL1C PE=2 SV=1	6.48E-85	40.852	100
P_KWMTBO MO16357	tr|Q5UAN3|Q5U AN3_BOMMO	40S ribosomal protein S11 OS=Bombyx mori OX=7091 GN=RpS11-1 PE=2 SV=1	6.00E-114	100	100	sp|P62280|RS11 _HUMAN	40S ribosomal protein S11 OS=Homo sapiens OX=9606 GN=RPS11 PE=1 SV=3	4.63E-88	77.848	100

Supplementary Table S3. Unique PIPs of each fibroin gene in the M4 and L5D5 PSGs.

unique PIPs of fibH at M4

Name	Bombyx-mori	Subject ID	Description	e-value	identity(%)	coverage(%)	Metazoa	Subject ID	Description	e-value	identity(%)	coverage(%)
P_KWMTBOMO00366		tr|H9ITD6|H9ITD6_BOMMO	Uncharacterized protein OS=Bombyx mori OX=7091 PE=4 SV=1	0	99.775	83.80414313		sp|O42254|IF2B1_CHICK	Insulin-like growth factor 2 mRNA-binding protein 1 OS=Gallus gallus OX=9031 GN=IGF2BP1 PE=1 SV=1	7.53E-128	44.578	93.78531073
P_KWMTBOMO00437		tr|H9IU13|H9IU13_BOMMO	PHB domain-containing protein OS=Bombyx mori OX=7091 PE=3 SV=1	0	100	98.82629108		sp|O61491|FLOT1_DROME	Flotillin-1 OS=Drosophila melanogaster OX=7227 GN=Flo1 PE=2 SV=1	0	83.059	99.76525822
P_KWMTBOMO00687		tr|H9J134|H9J134_BOMMO	Nucleolar GTP-binding protein 1 OS=Bombyx mori OX=7091 PE=4 SV=1	0	93.029	64.59627329		sp|Q9V411|NOG1_DROME	Nucleolar GTP-binding protein 1 OS=Drosophila melanogaster OX=7227 GN=Non1 PE=2 SV=1	0	68.666	100
P_KWMTBOMO00786		tr|H9JJK3|H9JJK3_BOMMO	HIT domain-containing protein OS=Bombyx mori OX=7091 GN=693069 PE=4 SV=1	6.16E-93	100	100		sp|P62958|HINT1_BOVIN	Histidine triad nucleotide-binding protein 1 OS=Bos taurus OX=9913 GN=HINT1 PE=1 SV=2	1.81E-62	68.75	100
P_KWMTBOMO00881		tr|D4QF47|D4QF47_BOMMO	Ced-6 protein OS=Bombyx mori OX=7091 GN=ced-6 PE=2 SV=1	0	100	100		sp|Q7JUY7|CED6_DROME	PTB domain-containing adapter protein ced-6 OS=Drosophila melanogaster OX=7227 GN=ced-6 PE=1 SV=1	3.82E-84	39.463	94.34697856
P_KWMTBOMO01030		tr|H9JCZ3|H9JCZ3_BOMMO	Vitellogenin domain-containing protein OS=Bombyx mori OX=7091 PE=4 SV=1	0	88.516	89.49191686		sp|Q865F1|MTP_PIG	Microsomal triglyceride transfer protein large subunit OS=Sus scrofa OX=9823 GN=MTTP PE=2 SV=1	4.29E-24	22.451	100
P_KWMTBOMO01320		tr|H9JH84|H9JH84_BOMMO	Uncharacterized protein OS=Bombyx mori OX=7091 PE=3 SV=1	3.35E-146	100	100		sp|P46222|RL11_DROME	60S ribosomal protein L11 OS=Drosophila melanogaster OX=7227 GN=RpL11 PE=1 SV=2	4.44E-121	91.011	91.28205128
P_KWMTBOMO01790		tr|H9J907|H9J907_BOMMO	TPR_REGION domain-containing protein OS=Bombyx mori OX=7091 PE=4 SV=1	0	99.758	78.05108798		sp|Q9VGU5|TTC14_DROME	Tetratricopeptide repeat protein 14 homolog OS=Drosophila melanogaster OX=7227 GN=CG6621 PE=1 SV=2	1.20E-82	48.657	31.69347209
P_KWMTBOMO01927		sp|P21894|SYAC_BOMMO	Alanine--tRNA ligase, cytoplasmic OS=Bombyx mori OX=7091 PE=1 SV=1	0	99.276	100		sp|P21894|SYAC_BOMMO	Alanine--tRNA ligase, cytoplasmic OS=Bombyx mori OX=7091 PE=1 SV=1	0	99.276	100
P_KWMTBOMO02059		tr|Q5UAM4|Q5UAM4_BOMMO	40S ribosomal protein S18 OS=Bombyx mori OX=7091 GN=RpS18 PE=2 SV=1	1.29E-111	100	100		sp|Q962R1|RS18_SPOFR	40S ribosomal protein S18 OS=Spodoptera frugiperda OX=7108 GN=RpS18 PE=2 SV=1	1.23E-110	99.342	100
P_KWMTBOMO02211		tr|E3VQ36|E3VQ36_BOMMO	Non-specific serine/threonine protein kinase OS=Bombyx mori OX=7091 GN=100529202 PE=2 SV=1	0	100	96.63496708		sp|M3TYT0|ROCK2_PIG	Rho-associated protein kinase 2 OS=Sus scrofa OX=9823 GN=ROCK2 PE=1 SV=1	0	44.876	99.92684711
P_KWMTBOMO02432		tr|H9JVD5|H9JVD5_BOMMO	Dolichyl-diphosphooligosaccharide--protein glycosyltransferase subunit 2 OS=Bombyx mori OX=7091 PE=3 SV=1	0	100	100		sp|Q3SZI6|RPN2_BOVIN	Dolichyl-diphosphooligosaccharide--protein glycosyltransferase subunit 2 OS=Bos taurus OX=9913 GN=RPN2 PE=2 SV=1	1.44E-58	28.155	99.35691318
P_KWMTBOMO02748		tr|H9J2I1|H9J2I1_BOMMO	TLC domain-containing protein OS=Bombyx mori OX=7091 GN=101745731 PE=3 SV=1	0	100	100		sp|Q6DED0|TR1L1_XENLA	Translocating chain-associated membrane protein 1-like 1 OS=Xenopus laevis OX=8355 GN=tram1l1 PE=2 SV=1	1.09E-74	35.979	100
P_KWMTBOMO02894		tr|H9J2B5|H9J2B5_BOMMO	Uncharacterized protein OS=Bombyx mori OX=7091 GN=101743323 PE=3 SV=1	0	98.864	98.05013928		sp|Q03427|LAMC_DROME	Lamin-C OS=Drosophila melanogaster OX=7227 GN=LamC PE=1 SV=2	2.44E-127	61.491	89.69359331
P_KWMTBOMO02994		tr|H9J281|H9J281_BOMMO	DNA photolyase OS=Bombyx mori OX=7091 PE=3 SV=1	0	100	96.04519774		sp|Q28811|PHR_POTTR	Deoxyribodipyrimidine photo-lyase OS=Potorous tridactylus OX=9310 GN=PHR PE=2 SV=1	0	59.205	90.01883239
P_KWMTBOMO03173		tr|Q1HPV7|Q1HPV7_BOMMO	Histone H2A OS=Bombyx mori OX=7091 GN=h2a.z PE=2 SV=1	1.20E-88	100	100		sp|Q6Y237|H2AV_PAGMA	Histone H2A.V OS=Pagrus major OX=143350 GN=h2az2 PE=2 SV=3	3.37E-81	97.561	95.34883721
P_KWMTBOMO03601		tr|S5RN30|S5RN30_BOMMO	Farnesoic acid O-methyltransferase OS=Bombyx mori OX=7091 GN=FaMeT6-1 PE=2 SV=1	6.39E-110	100	100		sp|Q66S13|NATT4_THANI	Natterin-4 OS=Thalassophryne nattereri OX=289382 PE=2 SV=1	5.91E-07	31.061	89.79591837
P_KWMTBOMO03748		tr|Q2F645|Q2F645_BOMMO	Transketolase OS=Bombyx mori OX=7091 PE=2 SV=1	0	100	100		sp|Q9D4D4|TKTL2_MOUSE	Transketolase-like protein 2 OS=Mus musculus OX=10090 GN=Tktl2 PE=1 SV=1	0	62.153	98.55305466
P_KWMTBOMO03860		tr|H9JKX7|H9JKX7_BOMMO	Uncharacterized protein OS=Bombyx mori OX=7091 PE=3 SV=1	0	97.603	100		sp|P26686|SRR55_DROME	Serine-arginine protein 55 OS=Drosophila melanogaster OX=7227 GN=B52 PE=1 SV=4	6.44E-97	77.487	66.78321678
P_KWMTBOMO04504		tr|H9J7F7|H9J7F7_BOMMO	Zinc-hook domain-containing protein OS=Bombyx mori OX=7091 PE=3 SV=1	2.04E-100	100	98		sp|Q9W252|RAD50_DROME	DNA repair protein RAD50 OS=Drosophila melanogaster OX=7227 GN=rad50 PE=2 SV=4	9.46E-37	43.791	100
P_KWMTBOMO04505		tr|H9J7F7|H9J7F7_BOMMO	Zinc-hook domain-containing protein OS=Bombyx mori OX=7091 PE=3 SV=1	0	99.3	85.54319932		sp|Q9W252|RAD50_DROME	DNA repair protein RAD50 OS=Drosophila melanogaster OX=7227 GN=rad50 PE=2 SV=4	1.59E-155	31.037	100
P_KWMTBOMO04691		tr|Q5TLD1|Q5TLD1_BOMMO	Replication protein A small subunit OS=Bombyx mori OX=7091 GN=BmRPA3 PE=2 SV=1	2.98E-98	100	100		--	--	--	--	--
P_KWMTBOMO04742		tr|Q5UAR2|Q5UAR2_BOMMO	60S ribosomal protein L27 OS=Bombyx mori OX=7091 GN=RpL27 PE=2 SV=1	1.18E-95	100	100		sp|P61354|RL27_RAT	60S ribosomal protein L27 OS=Rattus norvegicus OX=10116 GN=Rpl27 PE=2 SV=2	2.09E-64	71.324	100
P_KWMTBOMO05068		tr|Q5UAR4|Q5UAR4_BOMMO	Ribosomal protein L24 OS=Bombyx mori OX=7091 GN=RpL24 PE=2 SV=1	2.43E-111	100	100		sp|Q6F444|RL24_PLUXY	60S ribosomal protein L24 OS=Plutella xylostella OX=51655 GN=RpL24 PE=2 SV=1	1.72E-90	95.484	100
P_KWMTBOMO05179		tr|H9JSH7|H9JSH7_BOMMO	Uncharacterized protein OS=Bombyx mori OX=7091 PE=3 SV=1	0	97.051	100		sp|P10079|FBP1_STRPU	Fibropellin-1 OS=Strongylocentrotus purpuratus OX=7668 GN=EGF1 PE=1 SV=2	2.23E-127	42.751	15.75402635

P_KWMTBOMO05675	tr|Q5UAR5|Q5UAR5_BOMMO	Ribosomal protein L23A OS=Bombyx mori OX=7091 GN=RpL23A PE=2 SV=1	0	100	100	sp|P62752|RL23A_RAT	60S ribosomal protein L23a OS=Rattus norvegicus OX=10116 GN=Rpl23a PE=2 SV=1	8.65E-62	76.19	35.79545455
P_KWMTBOMO05680	tr|H9JBF1|H9JBF1_BOMMO	VWFC domain-containing protein OS=Bombyx mori OX=7091 PE=4 SV=1	0	97.473	98.92405063	--	--	--	--	--
P_KWMTBOMO05728	tr|H9JBC1|H9JBC1_BOMMO	Aspartate carbamoyltransferase OS=Bombyx mori OX=7091 PE=3 SV=1	0	98.881	100	sp|P05990|PYR1_DROME	CAD protein OS=Drosophila melanogaster OX=7227 GN=r PE=1 SV=3	0	68.238	100
P_KWMTBOMO06056	tr|H9J011|H9J011_BOMMO	DUF1907 domain-containing protein OS=Bombyx mori OX=7091 GN=101745847 PE=4 SV=1	0	100	100	sp|Q91V76|CK054_MOUSE	Ester hydrolase C11orf54 homolog OS=Mus musculus OX=10090 PE=1 SV=1	1.39E-89	44.72	98.47094801
P_KWMTBOMO06082	tr|H9IWS5|H9IWS5_BOMMO	Uncharacterized protein OS=Bombyx mori OX=7091 GN=101739385 PE=4 SV=1	0	100	100	--	--	--	--	--
P_KWMTBOMO06231	tr|H9IX18|H9IX18_BOMMO	RuvB-like helicase OS=Bombyx mori OX=7091 PE=3 SV=1	1.12E-166	98.765	65.14745308	sp|Q9DE27|RUVB2_XENLA	RuvB-like 2 OS=Xenopus laevis OX=8355 GN=ruvbl2 PE=2 SV=1	0	81.501	100
P_KWMTBOMO06482	tr|H9JQQ1|H9JQQ1_BOMMO	Uncharacterized protein OS=Bombyx mori OX=7091 PE=3 SV=1	0	98.577	100	sp|P25823|TUD_DROME	Maternal protein tudor OS=Drosophila melanogaster OX=7227 GN=tud PE=1 SV=2	5.70E-67	30.841	33.54231975
P_KWMTBOMO06632	tr|H9JQ89|H9JQ89_BOMMO	Nucleoprotein TPR OS=Bombyx mori OX=7091 PE=3 SV=1	0	87.867	58.41986456	sp|A1Z8P9|TPR_DROME	Nucleoprotein TPR OS=Drosophila melanogaster OX=7227 GN=Mtor PE=1 SV=1	0	31.858	100
P_KWMTBOMO06731	tr|H9JQE1|H9JQE1_BOMMO	Histone deacetylase OS=Bombyx mori OX=7091 PE=3 SV=1	0	91.686	89.64803313	sp|Q94517|HDAC1_DROME	Histone deacetylase HDAC1 OS=Drosophila melanogaster OX=7227 GN=HDAC1 PE=1 SV=2	0	92.634	92.75362319
P_KWMTBOMO06770	tr|H9JRG2|H9JRG2_BOMMO	Carboxylic ester hydrolase OS=Bombyx mori OX=7091 PE=3 SV=1	0	100	89.4934334	sp|P35502|ESTF_MYZPE	Esterase FE4 OS=Myzus persicae OX=13164 PE=1 SV=1	8.36E-86	35.577	97.56097561
P_KWMTBOMO06787	tr|H9JRF6|H9JRF6_BOMMO	SUMO-activating enzyme subunit OS=Bombyx mori OX=7091 PE=3 SV=1	0	96.875	100	sp|Q28GH3|SAE2_XENTR	SUMO-activating enzyme subunit 2 OS=Xenopus tropicalis OX=8364 GN=uba2 PE=2 SV=1	0	55	95.39473684
P_KWMTBOMO06803	tr|H9JR86|H9JR86_BOMMO	CP-type G domain-containing protein OS=Bombyx mori OX=7091 GN=101742911 PE=4 SV=1	0	100	100	sp|Q9W590|LSG1_DROME	Large subunit GTPase 1 homolog OS=Drosophila melanogaster OX=7227 GN=Ns3 PE=1 SV=1	5.32E-148	39.605	100
P_KWMTBOMO07076	tr|H9JLN4|H9JLN4_BOMMO	Uncharacterized protein OS=Bombyx mori OX=7091 PE=4 SV=1	0	99.76	80.03838772	sp|Q28EW0|TM87A_XENTR	Transmembrane protein 87A OS=Xenopus tropicalis OX=8364 GN=tmem87a PE=2 SV=1	5.77E-124	39.051	100
P_KWMTBOMO07274	tr|A0A4P9D450|A0A4P9D450_BOMMO	Piwi OS=Bombyx mori OX=7091 PE=2 SV=1	0	100	100	sp|A8D8P8|SIWI_BOMMO	Piwi-like protein Siwi OS=Bombyx mori OX=7091 GN=Siwi PE=1 SV=1	0	100	100
P_KWMTBOMO07294	tr|H9JM82|H9JM82_BOMMO	Uncharacterized protein OS=Bombyx mori OX=7091 GN=101739074 PE=4 SV=1	0	99.634	100	sp|O94826|TOM70_HUMAN	Mitochondrial import receptor subunit TOM70 OS=Homo sapiens OX=9606 GN=TOMM70 PE=1 SV=1	1.26E-176	44.718	100
P_KWMTBOMO07373	tr|H9J6N5|H9J6N5_BOMMO	Uncharacterized protein OS=Bombyx mori OX=7091 PE=4 SV=1	4.18E-97	100	100	sp|Q9U1N0|HRP65_CHITE	Hrp65 protein OS=Chironomus tentans OX=7153 GN=HRP65 PE=1 SV=1	6.32E-24	60.227	61.97183099
P_KWMTBOMO07380	--	--	--	--	--	--	--	--	--	--
P_KWMTBOMO07780	tr|H9IUH3|H9IUH3_BOMMO	Eukaryotic translation initiation factor 3 subunit M OS=Bombyx mori OX=7091 GN=101737255 PE=3 SV=1	0	100	100	sp|Q17D30|EIF3M_AEDAE	Eukaryotic translation initiation factor 3 subunit M OS=Aedes aegypti OX=7159 GN=AAEL004347 PE=3 SV=1	0	72.798	100
P_KWMTBOMO08044	tr|H9IV44|H9IV44_BOMMO	DNA-(apurinic or apyrimidinic site) endonuclease OS=Bombyx mori OX=7091 GN=101739740 PE=3 SV=1	0	100	100	sp|P27864|RRP1_DROME	Recombination repair protein 1 OS=Drosophila melanogaster OX=7227 GN=Rrp1 PE=1 SV=2	6.85E-131	44.487	83.2278481
P_KWMTBOMO08193	tr|H9JIX2|H9JIX2_BOMMO	Uncharacterized protein OS=Bombyx mori OX=7091 PE=3 SV=1	2.11E-91	98.571	100	sp|O95831|AIFM1_HUMAN	Apoptosis-inducing factor 1, mitochondrial OS=Homo sapiens OX=9606 GN=AIFM1 PE=1 SV=1	4.02E-30	42.958	100
P_KWMTBOMO08194	tr|H9JIX2|H9JIX2_BOMMO	Uncharacterized protein OS=Bombyx mori OX=7091 PE=3 SV=1	1.35E-72	66.522	52.15419501	sp|Q9VQ79|AIFM1_DROME	Putative apoptosis-inducing factor 1, mitochondrial OS=Drosophila melanogaster OX=7227 GN=AIF PE=2 SV=2	5.12E-60	35.423	72.33560091
P_KWMTBOMO08213	tr|H9JIW2|H9JIW2_BOMMO	Uncharacterized protein OS=Bombyx mori OX=7091 GN=101746972 PE=4 SV=1	0	100	100	sp|P51610|HCFC1_HUMAN	Host cell factor 1 OS=Homo sapiens OX=9606 GN=HCFC1 PE=1 SV=2	0	71.99	45.58472554
P_KWMTBOMO08404	tr|H9JI59|H9JI59_BOMMO	RNA cytidine acetyltransferase OS=Bombyx mori OX=7091 PE=3 SV=1	0	96.934	100	sp|Q9W3C1|NAT10_DROME	RNA cytidine acetyltransferase OS=Drosophila melanogaster OX=7227 GN=l(1)G0020 PE=1 SV=2	0	60.513	99.1861648
P_KWMTBOMO08652	tr|H9JDP0|H9JDP0_BOMMO	Diadenosine tetraphosphate synthetase OS=Bombyx mori OX=7091 PE=3 SV=1	0	100	100	sp|Q04451|SYG_BOMMO	Glycine--tRNA ligase OS=Bombyx mori OX=7091 PE=1 SV=2	0	99.118	100
P_KWMTBOMO08740	tr|H9JEF8|H9JEF8_BOMMO	UBR-type domain-containing protein OS=Bombyx mori OX=7091 PE=4 SV=1	0	99.168	55.82430341	sp|Q9VLT5|POE_DROME	Protein purity of essence OS=Drosophila melanogaster OX=7227 GN=poe PE=1 SV=1	0	40.577	100
P_KWMTBOMO08865	tr|H9JE95|H9JE95_BOMMO	Protein arginine N-methyltransferase OS=Bombyx mori OX=7091 PE=3 SV=1	0	99.674	91.2202381	sp|O14744|ANM5_HUMAN	Protein arginine N-methyltransferase 5 OS=Homo sapiens OX=9606 GN=PRMT5 PE=1 SV=4	7.08E-170	40.413	100
P_KWMTBOMO08889	tr|H9JE82|H9JE82_BOMMO	ZP domain-containing protein OS=Bombyx mori OX=7091 PE=4 SV=1	0	100	51.98863636	sp|Q19053|CUT4_CAEEL	Cuticlin-4 OS=Caenorhabditis elegans OX=6239 GN=cut-4 PE=2 SV=2	2.15E-08	24	31.96022727
P_KWMTBOMO08926	tr|Q9NGS0|Q9NGS0_BOMMO	Serpin-2 OS=Bombyx mori OX=7091 PE=2 SV=1	0	92.042	94.25	sp|Q5MGH0|SPI3_LONON	Serine protease inhibitor 3/4 (Fragment) OS=Lonomia obliqua OX=304329 PE=1 SV=1	3.16E-87	41.176	85
P_KWMTBOMO08983	tr|Q5UAT2|Q5UAT2_BOMMO	60S ribosomal protein L8 OS=Bombyx mori OX=7091 GN=RpL8 PE=2 SV=1	0	100	100	sp|Q95V39|RL8_SPOFR	60S ribosomal protein L8 OS=Spodoptera frugiperda OX=7108 GN=RpL8 PE=2 SV=1	0	100	100
P_KWMTBOMO09034	tr|H9JDJ7|H9JDJ7_BOMMO	RNA helicase OS=Bombyx mori OX=7091 PE=3 SV=1	0	96.038	74.22969188	sp|P16381|DDX3L_MOUSE	Putative ATP-dependent RNA helicase Pl10 OS=Mus musculus OX=10090 GN=D1Pas1 PE=1 SV=1	0	56.444	94.53781513

P_KWMTBOMO09057	tr|H9JDA6|H9JDA6_BOMMO	C2 domain-containing protein OS=Bombyx mori OX=7091 PE=4 SV=1	0	92	57.71567436	sp|A0FGR8|ESYT2_HUMAN	Extended synaptotagmin-2 OS=Homo sapiens OX=9606 GN=ESYT2 PE=1 SV=1	1.37E-145	32.94	100
P_KWMTBOMO09300	tr|Q5UAU1|Q5UAU1_BOMMO	60S acidic ribosomal protein P0 OS=Bombyx mori OX=7091 GN=RpP0 PE=1 SV=1	0	100	100	sp|Q9U3U0|RLA0_CERCA	60S acidic ribosomal protein P0 OS=Ceratitis capitata OX=7213 GN=RpLP0 PE=3 SV=1	1.73E-177	81.388	100
P_KWMTBOMO09390	tr|H9JU84|H9JU84_BOMMO	Splicing factor, arginine/serine-rich 1 OS=Bombyx mori OX=7091 PE=3 SV=1	6.24E-155	100	100	sp|Q6NYA0|SRS1B_DANRE	Serine/arginine-rich splicing factor 1B OS=Danio rerio OX=7955 GN=srsf1b PE=2 SV=1	3.58E-87	71.939	91.1627907
P_KWMTBOMO09591	tr|H9JTH6|H9JTH6_BOMMO	Uncharacterized protein OS=Bombyx mori OX=7091 PE=3 SV=1	9.34E-169	100	55.27522936	sp|O61492|FLOT2_DROME	Flotillin-2 OS=Drosophila melanogaster OX=7227 GN=Flo2 PE=2 SV=3	0	76.923	95.41284404
P_KWMTBOMO09980	tr|Q1HPT4|Q1HPT4_BOMMO	14-3-3 epsilon OS=Bombyx mori OX=7091 PE=2 SV=1	0	100	100	sp|P92177|1433E_DROME	14-3-3 protein epsilon OS=Drosophila melanogaster OX=7227 GN=14-3-3epsilon PE=1 SV=2	4.71E-163	84.906	100
P_KWMTBOMO10493	tr|H9JC69|H9JC69_BOMMO	Eukaryotic translation initiation factor 3 subunit B OS=Bombyx mori OX=7091 PE=3 SV=1	0	99.568	100	sp|Q1HDZ5|EIF3B_BOMMO	Eukaryotic translation initiation factor 3 subunit B OS=Bombyx mori OX=7091 GN=eIF3-S9 PE=2 SV=1	0	99.568	100
P_KWMTBOMO10509	tr|Q5UAR7|Q5UAR7_BOMMO	Ribosomal protein L22 OS=Bombyx mori OX=7091 GN=RpL22 PE=2 SV=1	1.07E-106	100	100	sp|P52819|RL22_CAEEL	60S ribosomal protein L22 OS=Caenorhabditis elegans OX=6239 GN=rpl-22 PE=1 SV=3	4.50E-38	49.254	91.15646259
P_KWMTBOMO11045	tr|H9JRL1|H9JRL1_BOMMO	Uncharacterized protein OS=Bombyx mori OX=7091 PE=3 SV=1	0	28.363	91.51958674	sp|Q00963|SPTCB_DROME	Spectrin beta chain OS=Drosophila melanogaster OX=7227 GN=beta-Spec PE=1 SV=2	0	73.055	100
P_KWMTBOMO11098	tr|H9JGC5|H9JGC5_BOMMO	RNA-binding motif protein 21 OS=Bombyx mori OX=7091 GN=101736670 PE=4 SV=1	0	100	100	sp|Q1JPD6|STPAP_BOVIN	Speckle targeted PIP5K1A-regulated poly(A) polymerase OS=Bos taurus OX=9913 GN=TUT1 PE=2 SV=1	3.32E-28	31.646	29.4044665
P_KWMTBOMO11276	tr|H9IZS8|H9IZS8_BOMMO	MCM domain-containing protein OS=Bombyx mori OX=7091 GN=101746848 PE=3 SV=1	0	100	54.65346535	sp|Q26454|MCM4_DROME	DNA replication licensing factor MCM4 OS=Drosophila melanogaster OX=7227 GN=dpa PE=1 SV=2	0	68.515	100
P_KWMTBOMO11517	tr|H9J3H3|H9J3H3_BOMMO	Uncharacterized protein OS=Bombyx mori OX=7091 PE=4 SV=1	0	96.387	98.84169884	--	--	--	--	--
P_KWMTBOMO11519	tr|H9J3H4|H9J3H4_BOMMO	Uncharacterized protein OS=Bombyx mori OX=7091 PE=3 SV=1	0	68.669	56.41431739	sp|Q86VQ3|TXND2_HUMAN	Thioredoxin domain-containing protein 2 OS=Homo sapiens OX=9606 GN=TXNDC2 PE=1 SV=4	2.10E-08	25.68	10.48463731
P_KWMTBOMO11731	sp|Q1HPK6|EF2_BOMMO	Translation elongation factor 2 OS=Bombyx mori OX=7091 GN=tef2 PE=1 SV=1	0	100	100	sp|Q1HPK6|EF2_BOMMO	Translation elongation factor 2 OS=Bombyx mori OX=7091 GN=tef2 PE=1 SV=1	0	100	100
P_KWMTBOMO11868	tr|H9J4H5|H9J4H5_BOMMO	Uncharacterized protein OS=Bombyx mori OX=7091 PE=4 SV=1	0	97.426	40.3960396	sp|Q03001|DYST_HUMAN	Dystonin OS=Homo sapiens OX=9606 GN=DST PE=1 SV=4	1.78E-166	24.498	100
P_KWMTBOMO12037	tr|H9J470|H9J470_BOMMO	DEK_C domain-containing protein OS=Bombyx mori OX=7091 PE=4 SV=1	0	91.221	94.15322581	sp|Q6AXS3|DEK_RAT	Protein DEK OS=Rattus norvegicus OX=10116 GN=Dek PE=1 SV=1	5.05E-20	47.368	19.15322581
P_KWMTBOMO12057	tr|H9J463|H9J463_BOMMO	Eukaryotic translation initiation factor 2 subunit 1 OS=Bombyx mori OX=7091 GN=693063 PE=3 SV=1	0	100	100	sp|P41374|IF2A_DROME	Eukaryotic translation initiation factor 2 subunit 1 OS=Drosophila melanogaster OX=7227 GN=eIF2alpha PE=2 SV=1	2.35E-165	74.832	89.75903614
P_KWMTBOMO12165	tr|H9JWZ8|H9JWZ8_BOMMO	Glutathione synthase OS=Bombyx mori OX=7091 PE=3 SV=1	1.95E-169	99.565	52.99539171	sp|P35668|GSHB_XENLA	Glutathione synthetase OS=Xenopus laevis OX=8355 GN=gss PE=2 SV=1	2.49E-108	43.564	93.0875576
P_KWMTBOMO12205	tr|H9JXB6|H9JXB6_BOMMO	Uncharacterized protein OS=Bombyx mori OX=7091 PE=3 SV=1	0	99.788	99.78858351	sp|Q5R1W7|ECHB_PANTR	Trifunctional enzyme subunit beta, mitochondrial OS=Pan troglodytes OX=9598 GN=HADHB PE=2 SV=1	0	66.667	96.40591966
P_KWMTBOMO12264	tr|H9JHZ0|H9JHZ0_BOMMO	CYTOSOL_AP domain-containing protein OS=Bombyx mori OX=7091 PE=3 SV=1	0	93.99	79.54110899	sp|Q5R7G6|PEPL1_PONAB	Probable aminopeptidase NPEPL1 OS=Pongo abelii OX=9601 GN=NPEPL1 PE=3 SV=2	0	54.981	99.80879541
P_KWMTBOMO12350	tr|H9JS78|H9JS78_BOMMO	DZF domain-containing protein OS=Bombyx mori OX=7091 PE=4 SV=1	0	95.423	86.70634921	sp|Q5REX3|ZFR_PONAB	Zinc finger RNA-binding protein OS=Pongo abelii OX=9601 GN=ZFR PE=2 SV=1	0	38.139	100
P_KWMTBOMO12497	tr|Q5UAN8|Q5UAN8_BOMMO	40S ribosomal protein S6 OS=Bombyx mori OX=7091 GN=RpS6 PE=2 SV=1	0	99.605	100	sp|Q94624|RS6_MANSE	40S ribosomal protein S6 OS=Manduca sexta OX=7130 GN=RpS6 PE=2 SV=1	2.56E-178	96.443	100
P_KWMTBOMO13567	tr|H9JN68|H9JN68_BOMMO	Uncharacterized protein OS=Bombyx mori OX=7091 PE=4 SV=1	0	100	61.18326118	--	--	--	--	--
P_KWMTBOMO13721	tr|A1YM11|A1YM11_BOMMO	CCT-theta OS=Bombyx mori OX=7091 PE=2 SV=1	0	99.45	100	sp|Q6EE31|TCPQ_CHICK	T-complex protein 1 subunit theta OS=Gallus gallus OX=9031 GN=CCT8 PE=1 SV=3	0	63.72	99.63302752
P_KWMTBOMO13979	tr|Q2F646|Q2F646_BOMMO	Hairy cell leukemia protein 1 OS=Bombyx mori OX=7091 GN=693008 PE=2 SV=1	0	99.614	62.40963855	sp|O95478|NSA2_HUMAN	Ribosome biogenesis protein NSA2 homolog OS=Homo sapiens OX=9606 GN=NSA2 PE=1 SV=1	9.59E-148	76.923	62.65060241
P_KWMTBOMO13997	tr|Q6T9Z7|Q6T9Z7_BOMMO	Fibroinase OS=Bombyx mori OX=7091 GN=693031 PE=2 SV=1	0	100	100	sp|Q26636|CATL_SARPE	Cathepsin L OS=Sarcophaga peregrina OX=7386 PE=1 SV=1	6.18E-177	68.902	96.18768328
P_KWMTBOMO14420	tr|H9IS77|H9IS77_BOMMO	Uncharacterized protein OS=Bombyx mori OX=7091 GN=101744691 PE=4 SV=1	0	99.786	99.78609626	sp|Q9BUJ2|HNRL1_HUMAN	Heterogeneous nuclear ribonucleoprotein U-like protein 1 OS=Homo sapiens OX=9606 GN=HNRNPUL1 PE=1 SV=2	7.54E-52	41.87	26.31016043
P_KWMTBOMO14568	tr|H9JH04|H9JH04_BOMMO	Importin N-terminal domain-containing protein OS=Bombyx mori OX=7091 PE=3 SV=1	4.68E-93	98.649	15.44885177	sp|Q5ZLT0|XPO7_CHICK	Exportin-7 OS=Gallus gallus OX=9031 GN=XPO7 PE=2 SV=1	0	67.216	100
P_KWMTBOMO14752	tr|H9J2U6|H9J2U6_BOMMO	PUA domain-containing protein OS=Bombyx mori OX=7091 GN=101744752 PE=3 SV=1	0	100	100	sp|O44081|DKC1_DROME	H/ACA ribonucleoprotein complex subunit 4 OS=Drosophila melanogaster OX=7227 GN=Nop60B PE=1 SV=1	0	76.69	91.47121535
P_KWMTBOMO14773	tr|H9J2R9|H9J2R9_BOMMO	Dynactin subunit 1 OS=Bombyx mori OX=7091 PE=3 SV=1	0	93.296	83.98096749	sp|P13496|DCTN1_DROME	Dynactin subunit 1 OS=Drosophila melanogaster OX=7227 GN=DCTN1-p150 PE=1 SV=2	0	41.095	100
P_KWMTBOMO14858	tr|H9JRJ9|H9JRJ9_BOMMO	Uncharacterized protein OS=Bombyx mori OX=7091 PE=4 SV=2	0	89.299	80.63814358	sp|Q02645|HTS_DROME	Protein hu-li tai shao OS=Drosophila melanogaster OX=7227 GN=hts PE=1 SV=2	0	56.932	49.16606236

P_KWMTBOMO15867		tr|H9JV01|H9JV01_BOMMO	Uncharacterized protein OS=Bombyx mori OX=7091 GN=101745818 PE=3 SV=1	0	100	100		sp|P48758|CBR1_MOUSE	Carbonyl reductase [NADPH] 1 OS=Mus musculus OX=10090 GN=Cbr1 PE=1 SV=3	2.70E-81	46.739	100
P_KWMTBOMO16130		tr|H9J4U4|H9J4U4_BOMMO	Calcium-transporting ATPase OS=Bombyx mori OX=7091 PE=3 SV=1	0	100	66.44385027		sp|Q16720|AT2B3_HUMAN	Plasma membrane calcium-transporting ATPase 3 OS=Homo sapiens OX=9606 GN=ATP2B3 PE=1 SV=3	0	62.683	100
P_KWMTBOMO16524		tr|H9J7Z3|H9J7Z3_BOMMO	RRM domain-containing protein OS=Bombyx mori OX=7091 PE=3 SV=1	1.00E-90	76.064	100		sp|Q5R1W5|SRSF2_PANTR	Serine/arginine-rich splicing factor 2 OS=Pan troglodytes OX=9598 GN=SRSF2 PE=2 SV=3	1.89E-49	77.083	62.33766234
P_KWMTBOMO16530		tr|H9J7S2|H9J7S2_BOMMO	Ribosome biogenesis regulatory protein OS=Bombyx mori OX=7091 GN=101743869 PE=3 SV=1	0	99.716	100		sp|Q9CYH6|RRS1_MOUSE	Ribosome biogenesis regulatory protein homolog OS=Mus musculus OX=10090 GN=Rrs1 PE=1 SV=1	1.15E-85	46.726	95.45454545

unique PIPs of fibH at L5D5

Name	Bombyx-mori	Subject ID	Description	e-value	identity(%)	coverage(%)	Metazoa	Subject ID	Description	e-value	identity(%)	coverage(%)
P_KWMTBOMO00200		tr|Q8T0Z6|Q8T0Z6_BOMMO	E2 ubiquitin-conjugating enzyme OS=Bombyx mori OX=7091 GN=BmubcD4 PE=3 SV=1	8.54E-151	100	100		sp|P52486|UBCD4_DROME	Ubiquitin-conjugating enzyme E2-22 kDa OS=Drosophila melanogaster OX=7227 GN=Ubc4 PE=1 SV=2	3.69E-104	72.362	100
P_KWMTBOMO00265		tr|H9ITT4|H9ITT4_BOMMO	Transmembrane protein 208 OS=Bombyx mori OX=7091 PE=3 SV=1	2.59E-130	100	100		sp|Q6NYP0|TM208_DANRE	Transmembrane protein 208 OS=Danio rerio OX=7955 GN=tmem208 PE=2 SV=1	1.92E-39	44.444	92.04545455
P_KWMTBOMO00303		tr|H9ITG0|H9ITG0_BOMMO	Rab-GAP TBC domain-containing protein OS=Bombyx mori OX=7091 PE=4 SV=1	0	98.655	99.87789988		sp|Q9VYY9|EVI5_DROME	Ecotropic viral integration site 5 ortholog OS=Drosophila melanogaster OX=7227 GN=Evi5 PE=1 SV=3	0	59.115	93.77289377
P_KWMTBOMO00362		tr|H9ITY0|H9ITY0_BOMMO	MSP domain-containing protein OS=Bombyx mori OX=7091 PE=3 SV=1	0	100	86.17021277		sp|Q16943|VP33_APLCA	Vesicle-associated membrane protein/synaptobrevin-binding protein OS=Aplysia californica OX=6500 PE=2 SV=1	1.32E-48	36.232	97.87234043
P_KWMTBOMO00373		tr|H9ITY4|H9ITY4_BOMMO	Aa_trans domain-containing protein OS=Bombyx mori OX=7091 GN=101746014 PE=4 SV=1	0	100	100		sp|Q9VT04|PATH_DROME	Proton-coupled amino acid transporter-like protein pathetic OS=Drosophila melanogaster OX=7227 GN=path PE=1 SV=1	6.88E-175	56.182	99.35344828
P_KWMTBOMO00375		tr|H9ITY5|H9ITY5_BOMMO	Uncharacterized protein OS=Bombyx mori OX=7091 PE=3 SV=1	0	87.972	100		sp|Q9VSA3|ACADM_DROME	Probable medium-chain specific acyl-CoA dehydrogenase, mitochondrial OS=Drosophila melanogaster OX=7227 GN=CG12262 PE=2 SV=1	0	73.558	98.34515366
P_KWMTBOMO00390		tr|H9ITC4|H9ITC4_BOMMO	Uncharacterized protein OS=Bombyx mori OX=7091 PE=4 SV=1	0	100	79.5620438		--	--	--	--	--
P_KWMTBOMO00427		tr|Q9NKX3|Q9NKX3_BOMMO	Coatomer subunit gamma OS=Bombyx mori OX=7091 GN=copg1 PE=2 SV=1	0	99.884	100		sp|Q8I0G5|COPG_DROME	Coatomer subunit gamma OS=Drosophila melanogaster OX=7227 GN=gammaCOP PE=2 SV=1	0	57.555	100
P_KWMTBOMO00473		tr|Q1HPX8|Q1HPX8_BOMMO	6-phosphogluconate dehydrogenase, decarboxylating OS=Bombyx mori OX=7091 PE=2 SV=1	0	100	100		sp|P52209|6PGD_HUMAN	6-phosphogluconate dehydrogenase, decarboxylating OS=Homo sapiens OX=9606 GN=PGD PE=1 SV=3	0	73.542	99.37888199
P_KWMTBOMO00722		tr|A0A0D6A6H2|A0A0D6A6H2_BOMMO	Methionine--tRNA ligase, cytoplasmic OS=Bombyx mori OX=7091 GN=BmMRS PE=2 SV=1	0	100	100		sp|Q6PF21|SYMC_XENLA	Methionine--tRNA ligase, cytoplasmic OS=Xenopus laevis OX=8355 GN=mars1 PE=2 SV=1	0	46.816	89.41058941
P_KWMTBOMO00794		tr|Q1HQ81|Q1HQ81_BOMMO	Nascent polypeptide associated complex protein alpha subunit OS=Bombyx mori OX=7091 PE=2 SV=1	1.91E-153	100	100		sp|Q94518|NACA_DROME	Nascent polypeptide-associated complex subunit alpha OS=Drosophila melanogaster OX=7227 GN=Nacalpha PE=1 SV=2	1.16E-78	65.455	100
P_KWMTBOMO00837		tr|H9JJI3|H9JJI3_BOMMO	Proteasome subunit beta OS=Bombyx mori OX=7091 PE=3 SV=1	0	100	97.32824427		sp|P99026|PSB4_MOUSE	Proteasome subunit beta type-4 OS=Mus musculus OX=10090 GN=Psmb4 PE=1 SV=1	1.89E-88	49.799	95.03816794
P_KWMTBOMO00838		tr|Q5UAR6|Q5UAR6_BOMMO	60S ribosomal protein L23 OS=Bombyx mori OX=7091 GN=RpL23 PE=2 SV=1	5.38E-99	100	100		sp|P48159|RL23_DROME	60S ribosomal protein L23 OS=Drosophila melanogaster OX=7227 GN=RpL23 PE=1 SV=2	5.37E-94	95	100
P_KWMTBOMO00860		tr|D2Y4R7|D2Y4R7_BOMMO	Coatomer subunit zeta OS=Bombyx mori OX=7091 GN=COPZ PE=2 SV=1	1.18E-132	100	100		sp|P35604|COPZ1_BOVIN	Coatomer subunit zeta-1 OS=Bos taurus OX=9913 GN=COPZ1 PE=1 SV=2	2.88E-83	62.921	100
P_KWMTBOMO00982		tr|H9IVS2|H9IVS2_BOMMO	Uncharacterized protein OS=Bombyx mori OX=7091 PE=4 SV=1	9.80E-180	100	48.71287129		sp|Q9D0F3|LMAN1_MOUSE	Protein ERGIC-53 OS=Mus musculus OX=10090 GN=Lman1 PE=1 SV=1	1.46E-138	44.353	96.43564356
P_KWMTBOMO01055		tr|Q5UAL7|Q5UAL7_BOMMO	40S ribosomal protein S26 OS=Bombyx mori OX=7091 GN=RpS26 PE=2 SV=1	1.02E-81	100	87.12121212		sp|Q9GT45|RS26_ANOGA	40S ribosomal protein S26 OS=Anopheles gambiae OX=7165 GN=RpS26 PE=3 SV=2	5.07E-67	84.348	87.12121212
P_KWMTBOMO01072		tr|H9JD17|H9JD17_BOMMO	Uncharacterized protein OS=Bombyx mori OX=7091 PE=4 SV=1	0	99.688	40.02493766		sp|Q9W3N6|USO1_DROME	General vesicular transport factor p115 OS=Drosophila melanogaster OX=7227 GN=p115 PE=1 SV=2	0	50.619	100
P_KWMTBOMO01076		tr|Q2F635|Q2F635_BOMMO	Adenosine kinase OS=Bombyx mori OX=7091 GN=692855 PE=2 SV=1	0	100	100		sp|Q64640|ADK_RAT	Adenosine kinase OS=Rattus norvegicus OX=10116 GN=Adk PE=1 SV=3	1.17E-116	48.949	95.41547278
P_KWMTBOMO01129		tr|H9JIS9|H9JIS9_BOMMO	F-actin-capping protein subunit beta OS=Bombyx mori OX=7091 PE=3 SV=1	0	100	100		sp|Q5XI32|CAPZB_RAT	F-actin-capping protein subunit beta OS=Rattus norvegicus OX=10116 GN=Capzb PE=1 SV=1	7.35E-168	81.481	98.18181818
P_KWMTBOMO01182		tr|Q1EPM0|Q1EPM0_BOMMO	Glyceraldehyde-3-phosphate dehydrogenase OS=Bombyx mori OX=7091 GN=BmGAPDH PE=2 SV=1	0	100	100		sp|P07487|G3P2_DROME	Glyceraldehyde-3-phosphate dehydrogenase 2 OS=Drosophila melanogaster OX=7227 GN=Gapdh2 PE=1 SV=2	0	84.036	100
P_KWMTBOMO01315		tr|Q1HPJ9|Q1HPJ9_BOMMO	V-type proton ATPase subunit H OS=Bombyx mori OX=7091 PE=2 SV=1	0	99.789	100		sp|Q9U5N0|VATH_MANSE	V-type proton ATPase subunit H OS=Manduca sexta OX=7130 PE=2 SV=1	0	96.421	100
P_KWMTBOMO01380		tr|H9JHI5|H9JHI5_BOMMO	Lipoyl-binding domain-containing protein OS=Bombyx mori OX=7091 PE=4 SV=1	5.01E-120	98.78	92.65536723		sp|Q19749|ODP2_CAEEL	Dihydrolipoyllysine-residue acetyltransferase component of pyruvate dehydrogenase complex, mitochondrial OS=Caenorhabditis elegans OX=6239 GN=dlat-1 PE=1 SV=1	2.61E-52	72.414	65.53672316

P_KWMTBOMO01423	tr|H9JHG0|H9JHG0_BOMMO	NADH-ubiquinone oxidoreductase 75 kDa subunit, mitochondrial OS=Bombyx mori OX=7091 PE=3 SV=1	0	98.518	100	sp|Q94511|NDUS1_DROME	NADH-ubiquinone oxidoreductase 75 kDa subunit, mitochondrial OS=Drosophila melanogaster OX=7227 GN=ND-75 PE=2 SV=3	0	64.393	100
P_KWMTBOMO01424	tr|H9JHC4|H9JHC4_BOMMO	Elongation factor 1-beta' OS=Bombyx mori OX=7091 PE=3 SV=1	5.51E-165	100	100	sp|P29522|EF1B2_BOMMO	Elongation factor 1-beta' OS=Bombyx mori OX=7091 PE=1 SV=2	3.22E-163	99.55	100
P_KWMTBOMO01431	tr|H9JHF8|H9JHF8_BOMMO	Voltage-dependent anion-selective channel protein 3 OS=Bombyx mori OX=7091 GN=101739703 PE=3 SV=1	5.89E-131	100	100	sp|Q94920|VDAC_DROME	Voltage-dependent anion-selective channel OS=Drosophila melanogaster OX=7227 GN=porin PE=1 SV=3	8.71E-82	60.656	100
P_KWMTBOMO01452	tr|H9JHD3|H9JHD3_BOMMO	Arginyl-tRNA synthetase OS=Bombyx mori OX=7091 GN=101737924 PE=3 SV=1	0	100	100	sp|Q6P1S4|SYRC_XENTR	Arginine--tRNA ligase, cytoplasmic OS=Xenopus tropicalis OX=8364 GN=rars1 PE=2 SV=1	0	58.85	99.26793558
P_KWMTBOMO01579	tr|H9JH15|H9JH15_BOMMO	Uncharacterized protein OS=Bombyx mori OX=7091 PE=3 SV=1	2.14E-165	100	21.03939648	sp|Q5F3X8|SC31A_CHICK	Protein transport protein Sec31A OS=Gallus gallus OX=9031 GN=SEC31A PE=2 SV=1	0	32.3	100
P_KWMTBOMO01662	tr|Q19N36|Q19N36_BOMMO	Small GTP binding protein RAB5 OS=Bombyx mori OX=7091 GN=733058 PE=2 SV=1	1.91E-161	100	100	sp|P61021|RAB5B_MOUSE	Ras-related protein Rab-5B OS=Mus musculus OX=10090 GN=Rab5b PE=1 SV=1	1.06E-122	80.374	100
P_KWMTBOMO01708	tr|H9J9C9|H9J9C9_BOMMO	Dynein light intermediate chain OS=Bombyx mori OX=7091 PE=3 SV=1	2.61E-111	99.367	34.05172414	sp|Q6PDL0|DC1L2_MOUSE	Cytoplasmic dynein 1 light intermediate chain 2 OS=Mus musculus OX=10090 GN=Dync1li2 PE=1 SV=2	2.12E-142	50.33	98.06034483
P_KWMTBOMO01796	tr|Q2F5J2|Q2F5J2_BOMMO	Prohibitin OS=Bombyx mori OX=7091 GN=692987 PE=2 SV=1	0	100	100	sp|P24156|L2CC_DROME	Protein l(2)37Cc OS=Drosophila melanogaster OX=7227 GN=l(2)37Cc PE=2 SV=2	6.28E-169	83.088	99.27007299
P_KWMTBOMO01799	tr|Q1HPL3|Q1HPL3_BOMMO	Proteasome 26S non-ATPase subunit 7 OS=Bombyx mori OX=7091 GN=733131 PE=2 SV=1	0	100	100	sp|P26270|PSMD7_DROME	26S proteasome non-ATPase regulatory subunit 7 OS=Drosophila melanogaster OX=7227 GN=Rpn8 PE=1 SV=6	0	80.967	100
P_KWMTBOMO01802	tr|Q3HR36|Q3HR36_BOMMO	L-threonine dehydrogenase OS=Bombyx mori OX=7091 PE=2 SV=1	0	99.706	93.66391185	sp|Q2KIR8|TDH_BOVIN	L-threonine 3-dehydrogenase, mitochondrial OS=Bos taurus OX=9913 GN=TDH PE=2 SV=1	4.54E-138	60.062	88.98071625
P_KWMTBOMO01806	tr|H9J9G9|H9J9G9_BOMMO	Cation-transporting ATPase OS=Bombyx mori OX=7091 GN=101743411 PE=3 SV=1	0	99.914	100	sp|Q9EPE9|AT131_MOUSE	Manganese-transporting ATPase 13A1 OS=Mus musculus OX=10090 GN=Atp13a1 PE=1 SV=2	0	57.252	100
P_KWMTBOMO01817	tr|H9IVQ5|H9IVQ5_BOMMO	RNA helicase OS=Bombyx mori OX=7091 PE=3 SV=1	0	100	50.33200531	sp|Q501J6|DDX17_MOUSE	Probable ATP-dependent RNA helicase DDX17 OS=Mus musculus OX=10090 GN=Ddx17 PE=1 SV=1	0	61.331	73.83798141
P_KWMTBOMO01941	tr|Q5UAQ4|Q5UAQ4_BOMMO	60S ribosomal protein L35 OS=Bombyx mori OX=7091 GN=RpL35 PE=2 SV=1	1.64E-84	100	100	sp|Q69CJ9|RL35_OPHHA	60S ribosomal protein L35 OS=Ophiophagus hannah OX=8665 GN=RPL35 PE=2 SV=3	1.41E-50	67.48	100
P_KWMTBOMO02088	tr|H9J0E7|H9J0E7_BOMMO	Lysine--tRNA ligase OS=Bombyx mori OX=7091 GN=101740883 PE=3 SV=1	0	100	100	sp|Q99MN1|SYK_MOUSE	Lysine--tRNA ligase OS=Mus musculus OX=10090 GN=Kars1 PE=1 SV=1	0	68.206	97.06896552
P_KWMTBOMO02223	tr|Q1HQC7|Q1HQC7_BOMMO	Aspartate aminotransferase OS=Bombyx mori OX=7091 PE=2 SV=1	0	99.768	100	sp|P00507|AATM_RAT	Aspartate aminotransferase, mitochondrial OS=Rattus norvegicus OX=10116 GN=Got2 PE=1 SV=2	0	62.06	92.34338747
P_KWMTBOMO02278	sp|Q75VN3|TCTP_BOMMO	Translationally-controlled tumor protein homolog OS=Bombyx mori OX=7091 GN=Tctp PE=2 SV=1	8.85E-126	100	100	sp|Q75VN3|TCTP_BOMMO	Translationally-controlled tumor protein homolog OS=Bombyx mori OX=7091 GN=Tctp PE=2 SV=1	5.77E-125	100	100
P_KWMTBOMO02450	tr|Q1HQ36|Q1HQ36_BOMMO	Ubiquitin conjugating enzyme E2 OS=Bombyx mori OX=7091 GN=Ubc13 PE=2 SV=1	4.04E-111	100	100	sp|P35128|UBE2N_DROME	Ubiquitin-conjugating enzyme E2 N OS=Drosophila melanogaster OX=7227 GN=ben PE=1 SV=1	3.20E-101	88.742	100
P_KWMTBOMO02477	tr|Q5UAR9|Q5UAR9_BOMMO	Ribosomal protein L19 OS=Bombyx mori OX=7091 GN=RpL19 PE=2 SV=1	1.82E-145	100	100	sp|P36241|RL19_DROME	60S ribosomal protein L19 OS=Drosophila melanogaster OX=7227 GN=RpL19 PE=1 SV=2	4.16E-113	84.343	99
P_KWMTBOMO02500	tr|H9IZ97|H9IZ97_BOMMO	Uncharacterized protein OS=Bombyx mori OX=7091 PE=4 SV=1	0	90.773	100	sp|O75439|MPPB_HUMAN	Mitochondrial-processing peptidase subunit beta OS=Homo sapiens OX=9606 GN=PMPCB PE=1 SV=2	0	68	96.56652361
P_KWMTBOMO02535	tr|H9IZB1|H9IZB1_BOMMO	Uncharacterized protein OS=Bombyx mori OX=7091 PE=4 SV=1	0	96.875	70.12687428	sp|O08730|GLYG_RAT	Glycogenin-1 OS=Rattus norvegicus OX=10116 GN=Gyg1 PE=2 SV=4	3.47E-117	52.395	38.52364475
P_KWMTBOMO02542	tr|Q8T7L8|Q8T7L8_BOMMO	Seroin 1 OS=Bombyx mori OX=7091 PE=2 SV=1	7.09E-73	97.222	100	--	--	--	--	--
P_KWMTBOMO02734	tr|Q9BPS1|Q9BPS1_BOMMO	Elongation factor 1 delta OS=Bombyx mori OX=7091 GN=ef-1d PE=2 SV=1	9.74E-145	100	100	sp|P32192|EF1D_ARTSA	Elongation factor 1-delta OS=Artemia salina OX=85549 PE=1 SV=2	5.90E-72	62.632	93.1372549
P_KWMTBOMO02775	tr|H9J1V6|H9J1V6_BOMMO	Very-long-chain enoyl-CoA reductase OS=Bombyx mori OX=7091 GN=101742899 PE=3 SV=1	0	100	100	sp|Q9N5Y2|TECR_CAEEL	Probable very-long-chain enoyl-CoA reductase art-1 OS=Caenorhabditis elegans OX=6239 GN=art-1 PE=3 SV=1	6.15E-119	56.209	100
P_KWMTBOMO02840	sp|Q2F5R8|EIF3E_BOMMO	Eukaryotic translation initiation factor 3 subunit E OS=Bombyx mori OX=7091 GN=eIF3-S6 PE=2 SV=1	0	100	100	sp|Q2F5R8|EIF3E_BOMMO	Eukaryotic translation initiation factor 3 subunit E OS=Bombyx mori OX=7091 GN=eIF3-S6 PE=2 SV=1	0	100	100
P_KWMTBOMO02914	--	--	--	--	--	sp|P13804|ETFA_HUMAN	Electron transfer flavoprotein subunit alpha, mitochondrial OS=Homo sapiens OX=9606 GN=ETFA PE=1 SV=1	4.08E-159	69.136	98.18181818
P_KWMTBOMO03003	tr|H9J272|H9J272_BOMMO	Adenylate kinase isoenzyme 6 homolog OS=Bombyx mori OX=7091 GN=101735934 PE=3 SV=1	2.60E-133	100	100	sp|Q7JYV7|KAD6_DROME	Adenylate kinase isoenzyme 6 homolog OS=Drosophila melanogaster OX=7227 GN=Ak6 PE=1 SV=1	3.13E-69	57.317	93.71428571
P_KWMTBOMO03027	tr|Q2F5M6|Q2F5M6_BOMMO	Ras small monomeric GTPase Rab6 OS=Bombyx mori OX=7091 PE=2 SV=1	1.13E-158	100	100	sp|O18334|RAB6_DROME	Ras-related protein Rab6 OS=Drosophila melanogaster OX=7227 GN=Rab6 PE=1 SV=1	2.75E-141	90.338	99.0430622
P_KWMTBOMO03092	tr|H9J8H0|H9J8H0_BOMMO	Arginine kinase OS=Bombyx mori OX=7091 PE=3 SV=1	0	100	98.44789357	sp|Q95PM9|KARG_PLOIN	Arginine kinase OS=Plodia interpunctella OX=58824 GN=ARGK PE=1 SV=1	0	92.394	78.71396896
P_KWMTBOMO03113	tr|Q2F5V9|Q2F5V9_BOMMO	Translocon-associated protein subunit beta OS=Bombyx mori OX=7091 PE=2 SV=1	1.01E-139	100	100	sp|P43308|SSRB_HUMAN	Translocon-associated protein subunit beta OS=Homo sapiens OX=9606 GN=SSR2 PE=1 SV=1	2.35E-60	52.809	93.68421053
P_KWMTBOMO03301	tr|E0D4V7|E0D4V7_BOMMO	Phenylalanyl-tRNA synthetase beta subunit OS=Bombyx mori OX=7091 GN=FRSB PE=2 SV=1	0	100	100	sp|Q9VCA5|SYFB_DROME	Phenylalanine--tRNA ligase beta subunit OS=Drosophila melanogaster OX=7227 GN=beta-PheRS PE=1 SV=1	0	66.893	100

P_KWMTBOMO03430	tr|Q2F5R4|Q2F5R4_BOMMO	Glutamate dehydrogenase (NAD(P)(+)) OS=Bombyx mori OX=7091 GN=692939 PE=2 SV=1	0	100	74.38692098	sp|P54385|DHE3_DROME	Glutamate dehydrogenase, mitochondrial OS=Drosophila melanogaster OX=7227 GN=Gdh PE=1 SV=2	0	79.821	76.29427793
P_KWMTBOMO03431	tr|H9JA24|H9JA24_BOMMO	Uncharacterized protein OS=Bombyx mori OX=7091 GN=101744716 PE=4 SV=1	0	100	100	sp|Q6P5I8|YIPF5_DANRE	Protein YIPF5 OS=Danio rerio OX=7955 GN=yipf5 PE=2 SV=1	6.66E-50	40.293	100
P_KWMTBOMO03622	tr|H9J9Y4|H9J9Y4_BOMMO	ADP-ribosylation factor-like protein 6-interacting protein 4 OS=Bombyx mori OX=7091 GN=101742090 PE=3 SV=1	8.34E-108	100	98.14814815	sp|Q5F4A9|AR6P4_CHICK	ADP-ribosylation factor-like protein 6-interacting protein 4 OS=Gallus gallus OX=9031 GN=ARL6IP4 PE=2 SV=1	4.30E-31	76.119	41.35802469
P_KWMTBOMO03630	tr|H9J9R7|H9J9R7_BOMMO	Uncharacterized protein OS=Bombyx mori OX=7091 PE=4 SV=1	2.53E-122	74.359	44.17475728	sp|Q7TMK9|HNRPQ_MOUSE	Heterogeneous nuclear ribonucleoprotein Q OS=Mus musculus OX=10090 GN=Syncrip PE=1 SV=2	1.08E-179	55.031	78.802589
P_KWMTBOMO03631	tr|H9J9Y8|H9J9Y8_BOMMO	ER membrane protein complex subunit 1 OS=Bombyx mori OX=7091 PE=3 SV=1	0	100	93.14845024	sp|Q5ZL00|EMC1_CHICK	ER membrane protein complex subunit 1 OS=Gallus gallus OX=9031 GN=EMC1 PE=2 SV=1	5.23E-32	32.143	45.67699837
P_KWMTBOMO03721	tr|H9JKE9|H9JKE9_BOMMO	TPR_REGION domain-containing protein OS=Bombyx mori OX=7091 PE=3 SV=2	0	97.586	100	sp|O43765|SGTA_HUMAN	Small glutamine-rich tetratricopeptide repeat-containing protein alpha OS=Homo sapiens OX=9606 GN=SGTA PE=1 SV=1	4.07E-71	42.997	100
P_KWMTBOMO03722	tr|H9JKE1|H9JKE1_BOMMO	Dynamin-type G domain-containing protein OS=Bombyx mori OX=7091 PE=4 SV=1	0	100	76.69172932	sp|Q9H4M9|EHD1_HUMAN	EH domain-containing protein 1 OS=Homo sapiens OX=9606 GN=EHD1 PE=1 SV=2	0	69.101	100
P_KWMTBOMO03951	tr|Q5UAL6|Q5UAL6_BOMMO	40S ribosomal protein S27 OS=Bombyx mori OX=7091 GN=RpS27 PE=2 SV=1	1.35E-59	100	100	sp|P55833|RS27_HOMAM	40S ribosomal protein S27 OS=Homarus americanus OX=6706 GN=RPS27 PE=3 SV=2	1.42E-47	78.571	100
P_KWMTBOMO04051	tr|H9JL88|H9JL88_BOMMO	UDPGT domain-containing protein OS=Bombyx mori OX=7091 PE=3 SV=1	0	95.709	99.9002991	sp|A0A291PQH4|UGT2_DACCO	UDP-glucosyltransferase 2 OS=Dactylopius coccus OX=765876 GN=UTG2 PE=1 SV=1	6.72E-74	31.836	51.04685942
P_KWMTBOMO04132	tr|H9JGQ9|H9JGQ9_BOMMO	WD_REPEATS_REGION domain-containing protein OS=Bombyx mori OX=7091 PE=4 SV=1	0	99.782	75.8677686	sp|Q9VU68|WDR1_DROME	Actin-interacting protein 1 OS=Drosophila melanogaster OX=7227 GN=flr PE=2 SV=1	0	66.998	99.66942149
P_KWMTBOMO04168	tr|H9JGM3|H9JGM3_BOMMO	Uncharacterized protein OS=Bombyx mori OX=7091 PE=3 SV=1	0	100	100	sp|Q94516|AT5F1_DROME	ATP synthase subunit b, mitochondrial OS=Drosophila melanogaster OX=7227 GN=ATPsynB PE=2 SV=2	1.44E-101	69.036	81.06995885
P_KWMTBOMO04170	tr|H9JGS8|H9JGS8_BOMMO	UTP--glucose-1-phosphate uridylyltransferase OS=Bombyx mori OX=7091 PE=3 SV=1	0	100	97.85575049	sp|Q07130|UGPA_BOVIN	UTP--glucose-1-phosphate uridylyltransferase OS=Bos taurus OX=9913 GN=UGP2 PE=1 SV=2	0	65.737	97.85575049
P_KWMTBOMO04330	tr|H9JX71|H9JX71_BOMMO	PCI domain-containing protein OS=Bombyx mori OX=7091 GN=101735320 PE=3 SV=1	0	100	100	sp|Q7KLV9|PSD11_DROME	26S proteasome non-ATPase regulatory subunit 11 OS=Drosophila melanogaster OX=7227 GN=Rpn6 PE=1 SV=1	0	76.54	100
P_KWMTBOMO04427	tr|H9J7B7|H9J7B7_BOMMO	Signal recognition particle receptor subunit beta OS=Bombyx mori OX=7091 PE=3 SV=1	0	100	100	sp|Q9Y5M8|SRPRB_HUMAN	Signal recognition particle receptor subunit beta OS=Homo sapiens OX=9606 GN=SRPRB PE=1 SV=3	2.92E-59	46.575	87.6
P_KWMTBOMO04529	tr|Q2F6C7|Q2F6C7_BOMMO	ADP-ribosylation factor OS=Bombyx mori OX=7091 GN=778456 PE=2 SV=1	9.95E-136	100	100	sp|P61210|ARF1_LOCMI	ADP-ribosylation factor 1 OS=Locusta migratoria OX=7004 GN=ARF1 PE=2 SV=2	7.09E-134	98.901	100
P_KWMTBOMO04584	tr|Q2F5K1|Q2F5K1_BOMMO	Signal sequence receptor subunit gamma OS=Bombyx mori OX=7091 PE=2 SV=1	1.07E-132	100	100	sp|Q9UNL2|SSRG_HUMAN	Translocon-associated protein subunit gamma OS=Homo sapiens OX=9606 GN=SSR3 PE=1 SV=1	8.00E-87	64.865	100
P_KWMTBOMO04594	tr|Q1HE02|Q1HE02_BOMMO	ALY OS=Bombyx mori OX=7091 GN=733049 PE=2 SV=1	0	100	100	sp|Q58EA2|THO4A_XENLA	THO complex subunit 4-A OS=Xenopus laevis OX=8355 GN=alyref-a PE=2 SV=1	3.73E-51	49.237	100
P_KWMTBOMO04740	tr|H9JK13|H9JK13_BOMMO	Endoplasmic reticulum transmembrane protein OS=Bombyx mori OX=7091 GN=101735495 PE=3 SV=1	8.35E-166	100	100	sp|Q5R8H3|BAP31_PONAB	B-cell receptor-associated protein 31 OS=Pongo abelii OX=9601 GN=BCAP31 PE=2 SV=3	1.47E-45	42.742	100
P_KWMTBOMO04768	tr|H9JK00|H9JK00_BOMMO	Valyl-tRNA synthetase OS=Bombyx mori OX=7091 PE=3 SV=1	0	93.973	100	sp|Q04462|SYVC_RAT	Valine--tRNA ligase OS=Rattus norvegicus OX=10116 GN=Vars1 PE=2 SV=2	0	59.277	100
P_KWMTBOMO04789	tr|Q2F5P7|Q2F5P7_BOMMO	Mitochondrial matrix protein p32 OS=Bombyx mori OX=7091 PE=2 SV=1	0	100	100	sp|Q3T0B6|C1QBP_BOVIN	Complement component 1 Q subcomponent-binding protein, mitochondrial OS=Bos taurus OX=9913 GN=C1QBP PE=2 SV=1	7.30E-40	32.042	100
P_KWMTBOMO04906	tr|H9JEW9|H9JEW9_BOMMO	Aminopeptidase N-8 OS=Bombyx mori OX=7091 GN=101737565 PE=2 SV=1	0	98.024	92.42009132	sp|P15145|AMPN_PIG	Aminopeptidase N OS=Sus scrofa OX=9823 GN=ANPEP PE=1 SV=4	9.30E-128	32.057	76.34703196
P_KWMTBOMO04966	tr|E5EVW5|E5EVW5_BOMMO	Inorganic diphosphatase OS=Bombyx mori OX=7091 PE=3 SV=1	0	100	86.22754491	sp|O77460|IPYR_DROME	Inorganic pyrophosphatase OS=Drosophila melanogaster OX=7227 GN=Nurf-38 PE=1 SV=3	1.04E-129	58.389	89.22155689
P_KWMTBOMO04992	tr|H9JEZ8|H9JEZ8_BOMMO	Uncharacterized protein OS=Bombyx mori OX=7091 PE=3 SV=1	0	99.614	100	sp|Q21929|DCXR_CAEEL	L-xylulose reductase OS=Caenorhabditis elegans OX=6239 GN=dhs-21 PE=1 SV=2	5.68E-38	33.597	97.68339768
P_KWMTBOMO05078	tr|Q2F5L1|Q2F5L1_BOMMO	Signal peptidase complex catalytic subunit SEC11 OS=Bombyx mori OX=7091 PE=2 SV=1	3.67E-131	99.438	100	sp|Q9D8V7|SC11C_MOUSE	Signal peptidase complex catalytic subunit SEC11C OS=Mus musculus OX=10090 GN=Sec11c PE=1 SV=3	1.20E-103	79.429	98.31460674
P_KWMTBOMO05178	tr|Q2F5M8|Q2F5M8_BOMMO	Phosphoserine aminotransferase OS=Bombyx mori OX=7091 PE=2 SV=1	0	100	100	sp|Q9VAN0|SERC_DROME	Probable phosphoserine aminotransferase OS=Drosophila melanogaster OX=7227 GN=CG11899 PE=2 SV=1	9.90E-147	64.935	96.85534591
P_KWMTBOMO05258	tr|H9IZ08|H9IZ08_BOMMO	Peptidase_M24 domain-containing protein OS=Bombyx mori OX=7091 GN=101746307 PE=3 SV=1	0	100	100	sp|Q6AYD3|PA2G4_RAT	Proliferation-associated protein 2G4 OS=Rattus norvegicus OX=10116 GN=Pa2g4 PE=1 SV=1	1.26E-149	57.778	94.98680739
P_KWMTBOMO05281	tr|H9IZ19|H9IZ19_BOMMO	Uncharacterized protein OS=Bombyx mori OX=7091 GN=101735890 PE=3 SV=1	4.73E-144	100	100	sp|Q9CQC9|SAR1B_MOUSE	GTP-binding protein SAR1b OS=Mus musculus OX=10090 GN=Sar1b PE=1 SV=1	9.93E-107	75	100
P_KWMTBOMO05299	tr|H9IYR7|H9IYR7_BOMMO	Uncharacterized protein OS=Bombyx mori OX=7091 PE=3 SV=1	0	96.091	98.08306709	sp|O95749|GGPPS_HUMAN	Geranylgeranyl pyrophosphate synthase OS=Homo sapiens OX=9606 GN=GGPS1 PE=1 SV=1	5.33E-131	60.357	89.45686901
P_KWMTBOMO05321	tr|H9IYQ9|H9IYQ9_BOMMO	Uncharacterized protein OS=Bombyx mori OX=7091 PE=3 SV=1	0	99.85	97.65395894	sp|Q9VA73|CMC_DROME	Calcium-binding mitochondrial carrier protein Aralar1 OS=Drosophila melanogaster OX=7227 GN=aralar1 PE=2 SV=1	0	69.001	98.38709677
P_KWMTBOMO05464	tr|H9JB09|H9JB09_BOMMO	Vasa intronic protein OS=Bombyx mori OX=7091 GN=101741313 PE=2 SV=1	0	100	100	sp|Q6AXS5|PAIRB_RAT	Plasminogen activator inhibitor 1 RNA-binding protein OS=Rattus norvegicus OX=10116 GN=Serbp1 PE=1 SV=2	1.23E-20	40.625	41.1311054

P_KWMTBOMO05651	tr|H9JB94|H9JB94_BOMMO	Malectin domain-containing protein OS=Bombyx mori OX=7091 GN=101744143 PE=3 SV=1	0	99.621	100	sp|Q14165|MLEC_HUMAN	Malectin OS=Homo sapiens OX=9606 GN=MLEC PE=1 SV=1	8.20E-90	53.226	93.93939394
P_KWMTBOMO05687	tr|A8R081|A8R081_BOMMO	Bombyx homolog of P-element somatic inhibitor OS=Bombyx mori OX=7091 GN=BmPSI PE=2 SV=1	1.37E-12	23.754	27.52219532	sp|Q00341|VIGLN_HUMAN	Vigilin OS=Homo sapiens OX=9606 GN=HDLBP PE=1 SV=2	0	49.197	100
P_KWMTBOMO05723	tr|Q5UAM7|Q5UAM7_BOMMO	Ribosomal protein S15A OS=Bombyx mori OX=7091 GN=RpS15A PE=2 SV=1	5.26E-94	100	100	sp|Q6XIM8|RS15A_DROYA	40S ribosomal protein S15a OS=Drosophila yakuba OX=7245 GN=RpS15Aa PE=2 SV=3	3.84E-87	93.077	100
P_KWMTBOMO05784	tr|H9JBN7|H9JBN7_BOMMO	DUF1681 domain-containing protein OS=Bombyx mori OX=7091 PE=3 SV=1	3.74E-118	100	66.10878661	sp|Q9VXB0|NECAP_DROME	NECAP-like protein CG9132 OS=Drosophila melanogaster OX=7227 GN=CG9132 PE=2 SV=1	5.98E-101	59.216	100
P_KWMTBOMO05806	tr|H9JBP6|H9JBP6_BOMMO	Thioredoxin domain-containing protein OS=Bombyx mori OX=7091 PE=4 SV=1	0	100	93.97260274	sp|Q9D1Q6|ERP44_MOUSE	Endoplasmic reticulum resident protein 44 OS=Mus musculus OX=10090 GN=Erp44 PE=1 SV=1	3.26E-135	51.781	100
P_KWMTBOMO05810	tr|H9JBQ0|H9JBQ0_BOMMO	Uncharacterized protein OS=Bombyx mori OX=7091 PE=4 SV=1	0	99.381	50.35650624	sp|Q8SWU7|OLA1_DROME	Obg-like ATPase 1 OS=Drosophila melanogaster OX=7227 GN=CG1354 PE=1 SV=1	4.43E-180	78.274	14.97326203
P_KWMTBOMO06045	tr|H9J005|H9J005_BOMMO	TOG domain-containing protein OS=Bombyx mori OX=7091 PE=3 SV=2	0	93.348	100	sp|E9PVA8|GCN1_MOUSE	eIF-2-alpha kinase activator GCN1 OS=Mus musculus OX=10090 GN=Gcn1 PE=1 SV=1	0	45.751	100
P_KWMTBOMO06291	tr|H9IX45|H9IX45_BOMMO	C2H2-type domain-containing protein OS=Bombyx mori OX=7091 GN=101746228 PE=4 SV=1	0	100	100	--	--	--	--	--
P_KWMTBOMO06299	tr|H9IX47|H9IX47_BOMMO	RNA exonuclease 4 OS=Bombyx mori OX=7091 GN=101746969 PE=3 SV=1	6.14E-62	100	81.65137615	sp|Q6PAQ4|REXO4_MOUSE	RNA exonuclease 4 OS=Mus musculus OX=10090 GN=Rexo4 PE=2 SV=2	2.97E-07	35.526	69.72477064
P_KWMTBOMO06354	tr|H9JR23|H9JR23_BOMMO	Aminotran_1_2 domain-containing protein OS=Bombyx mori OX=7091 PE=4 SV=1	0	94.624	90.11627907	sp|Q28DB5|ALAT2_XENTR	Alanine aminotransferase 2 OS=Xenopus tropicalis OX=8364 GN=gpt2 PE=2 SV=1	0	58.078	94.76744186
P_KWMTBOMO06373	tr|Q2F5L8|Q2F5L8_BOMMO	Ribonucloprotein OS=Bombyx mori OX=7091 PE=2 SV=1	8.53E-93	100	100	sp|Q5XH16|NH2L1_XENLA	NHP2-like protein 1 OS=Xenopus laevis OX=8355 GN=snu13 PE=2 SV=1	4.36E-77	81.89	97.69230769
P_KWMTBOMO06427	tr|Q2F5L0|Q2F5L0_BOMMO	Signal sequence receptor subunit alpha OS=Bombyx mori OX=7091 GN=778461 PE=2 SV=1	0	100	100	sp|P43307|SSRA_HUMAN	Translocon-associated protein subunit alpha OS=Homo sapiens OX=9606 GN=SSR1 PE=1 SV=3	6.17E-70	50.45	79.56989247
P_KWMTBOMO06595	tr|I3VR81|I3VR81_BOMMO	Aminopeptidase OS=Bombyx mori OX=7091 PE=2 SV=1	0	100	100	sp|P55786|PSA_HUMAN	Puromycin-sensitive aminopeptidase OS=Homo sapiens OX=9606 GN=NPEPPS PE=1 SV=2	0	57.324	100
P_KWMTBOMO06639	tr|Q2F5Q1|Q2F5Q1_BOMMO	Lysophospholipase OS=Bombyx mori OX=7091 PE=2 SV=1	6.85E-166	100	100	sp|Q3MHR0|LYPA1_BOVIN	Acyl-protein thioesterase 1 OS=Bos taurus OX=9913 GN=LYPLA1 PE=2 SV=1	6.45E-78	53.425	99.54545455
P_KWMTBOMO06660	tr|Q2F640|Q2F640_BOMMO	Ubiquinol-cytochrome c reductase core protein II OS=Bombyx mori OX=7091 GN=100127121 PE=2 SV=1	0	100	100	sp|P22695|QCR2_HUMAN	Cytochrome b-c1 complex subunit 2, mitochondrial OS=Homo sapiens OX=9606 GN=UQCRC2 PE=1 SV=3	1.22E-72	34.615	95.19450801
P_KWMTBOMO06763	tr|H9JR70|H9JR70_BOMMO	Uncharacterized protein OS=Bombyx mori OX=7091 PE=4 SV=1	0	99.694	100	--	--	--	--	--
P_KWMTBOMO06822	tr|H9JR93|H9JR93_BOMMO	Aa_trans domain-containing protein OS=Bombyx mori OX=7091 PE=4 SV=1	0	79.582	95.77777778	sp|Q5RC98|S38AA_PONAB	Putative sodium-coupled neutral amino acid transporter 10 OS=Pongo abelii OX=9601 GN=SLC38A10 PE=2 SV=1	1.98E-84	35.204	43.55555556
P_KWMTBOMO06927	tr|Q0N2S2|Q0N2S2_BOMMO	Small GTP-binding protein Rab10 OS=Bombyx mori OX=7091 GN=778528 PE=2 SV=1	2.84E-152	100	100	sp|P22127|RAB10_DIPOM	Ras-related protein Rab-10 OS=Diplobatis ommata OX=1870830 PE=2 SV=1	9.80E-110	75.862	100
P_KWMTBOMO06999	tr|H9JLS3|H9JLS3_BOMMO	TPR_REGION domain-containing protein OS=Bombyx mori OX=7091 PE=3 SV=1	0	100	100	sp|P31948|STIP1_HUMAN	Stress-induced-phosphoprotein 1 OS=Homo sapiens OX=9606 GN=STIP1 PE=1 SV=1	0	52.007	100
P_KWMTBOMO07061	tr|H9JLP3|H9JLP3_BOMMO	Uncharacterized protein OS=Bombyx mori OX=7091 PE=3 SV=1	2.05E-51	100	50.31055901	sp|Q5R979|PGM2L_PONAB	Glucose 1,6-bisphosphate synthase OS=Pongo abelii OX=9601 GN=PGM2L1 PE=2 SV=1	4.81E-32	37.5	99.37888199
P_KWMTBOMO07103	tr|Q2F5P8|Q2F5P8_BOMMO	Malate dehydrogenase OS=Bombyx mori OX=7091 PE=2 SV=1	0	100	100	sp|O88989|MDHC_RAT	Malate dehydrogenase, cytoplasmic OS=Rattus norvegicus OX=10116 GN=Mdh1 PE=1 SV=3	7.95E-157	65.653	99.39577039
P_KWMTBOMO07214	tr|D5MTP2|D5MTP2_BOMMO	Bm8 interacting protein 2-11 OS=Bombyx mori OX=7091 PE=2 SV=1	0	94.898	78.82037534	sp|Q9VMA7|TGO1_DROME	Transport and Golgi organization protein 1 OS=Drosophila melanogaster OX=7227 GN=Tango1 PE=1 SV=2	6.97E-56	30.097	48.32439678
P_KWMTBOMO07278	tr|H9JM89|H9JM89_BOMMO	PNP_UDP_1 domain-containing protein OS=Bombyx mori OX=7091 GN=101736970 PE=3 SV=1	0	98.214	99.11504425	sp|Q16831|UPP1_HUMAN	Uridine phosphorylase 1 OS=Homo sapiens OX=9606 GN=UPP1 PE=1 SV=1	1.13E-111	53.135	89.38053097
P_KWMTBOMO07323	tr|Q2F6C4|Q2F6C4_BOMMO	T-complex protein 1 subunit delta OS=Bombyx mori OX=7091 PE=2 SV=1	0	99.814	100	sp|Q9NB32|TCPD_OCHTR	T-complex protein 1 subunit delta OS=Ochlerotatus triseriatus OX=7162 PE=2 SV=1	0	69.088	100
P_KWMTBOMO07368	tr|H9JMD1|H9JMD1_BOMMO	Uncharacterized protein OS=Bombyx mori OX=7091 GN=101744608 PE=3 SV=1	8.23E-94	100	100	sp|P24310|CX7A1_HUMAN	Cytochrome c oxidase subunit 7A1, mitochondrial OS=Homo sapiens OX=9606 GN=COX7A1 PE=1 SV=2	3.33E-12	47.458	45.38461538
P_KWMTBOMO07554	tr|H9J8L8|H9J8L8_BOMMO	Uncharacterized protein OS=Bombyx mori OX=7091 PE=4 SV=1	8.48E-153	100	53.18627451	sp|Q9NQC3|RTN4_HUMAN	Reticulon-4 OS=Homo sapiens OX=9606 GN=RTN4 PE=1 SV=2	2.84E-61	45.494	57.10784314
P_KWMTBOMO07600	tr|Q1HPU6|Q1HPU6_BOMMO	Sec61p gamma subunit OS=Bombyx mori OX=7091 GN=733141 PE=2 SV=1	5.06E-45	100	100	sp|Q7Z1B8|S61G1_GRYOR	Protein transport protein Sec61 subunit gamma OS=Gryllotalpa orientalis OX=213494 GN=SEC61G PE=3 SV=1	3.67E-42	91.176	100
P_KWMTBOMO07761	tr|H9IUG4|H9IUG4_BOMMO	Uncharacterized protein OS=Bombyx mori OX=7091 PE=4 SV=1	0	94.605	26.40722724	sp|Q8MSS1|LVA_DROME	Protein lava lamp OS=Drosophila melanogaster OX=7227 GN=lva PE=1 SV=2	5.58E-15	35.644	7.01876303
P_KWMTBOMO07772	tr|Q5UAN4|Q5UAN4_BOMMO	Ribosomal protein S10 OS=Bombyx mori OX=7091 GN=RpS10 PE=2 SV=1	4.40E-118	100	100	sp|Q962R9|RS10_SPOFR	40S ribosomal protein S10 OS=Spodoptera frugiperda OX=7108 GN=RpS10 PE=2 SV=1	1.19E-105	92.453	99.375
P_KWMTBOMO07857	tr|Q5R1P5|Q5R1P5_BOMMO	Heat shock protein hsp21.4 OS=Bombyx mori OX=7091 GN=Hsp21.4 PE=2 SV=1	4.58E-139	100	100	sp|Q00649|HSPB1_CHICK	Heat shock protein beta-1 OS=Gallus gallus OX=9031 GN=HSPB1 PE=2 SV=1	5.62E-19	47.727	47.05882353

P_KWMTBOMO08041	tr|H9IV46|H9IV46_BOMMO	Rab proteins geranylgeranyltransferase component A OS=Bombyx mori OX=7091 GN=101740100 PE=3 SV=1	0	100	100	sp|Q9V8W3|RABEP_DROME	Rab proteins geranylgeranyltransferase component A OS=Drosophila melanogaster OX=7227 GN=Rep PE=1 SV=1	6.05E-105	36.542	97.32313576
P_KWMTBOMO08056	tr|H9IV39|H9IV39_BOMMO	Rab GDP dissociation inhibitor OS=Bombyx mori OX=7091 GN=101738804 PE=3 SV=1	0	100	100	sp|P21856|GDIA_BOVIN	Rab GDP dissociation inhibitor alpha OS=Bos taurus OX=9913 GN=GDI1 PE=1 SV=1	0	68.75	100
P_KWMTBOMO08064	tr|Q5UAR1|Q5UAR1_BOMMO	60S ribosomal protein L27a OS=Bombyx mori OX=7091 GN=RpL27A PE=2 SV=1	3.15E-107	100	100	sp|P47830|RL27A_XENLA	60S ribosomal protein L27a OS=Xenopus laevis OX=8355 GN=rpl27a PE=2 SV=2	1.19E-82	77.027	100
P_KWMTBOMO08098	tr|G9FL14|G9FL14_BOMMO	DNA supercoiling factor OS=Bombyx mori OX=7091 GN=LOC692759 PE=2 SV=1	1.98E-48	100	100	sp|B5X4E0|CALUB_SALSA	Calumenin-B OS=Salmo salar OX=8030 GN=calub PE=2 SV=1	7.32E-13	49.333	100
P_KWMTBOMO08204	tr|H9JIZ5|H9JIZ5_BOMMO	Arf-GAP domain-containing protein OS=Bombyx mori OX=7091 GN=101746692 PE=4 SV=1	0	100	100	sp|Q28CM8|ARFG2_XENTR	ADP-ribosylation factor GTPase-activating protein 2 OS=Xenopus tropicalis OX=8364 GN=arfgap2 PE=2 SV=1	1.27E-99	39.455	100
P_KWMTBOMO08323	tr|H9JI91|H9JI91_BOMMO	Uncharacterized protein OS=Bombyx mori OX=7091 PE=4 SV=1	0	99.643	98.24561404	sp|Q5R4J9|NAA15_PONAB	N-alpha-acetyltransferase 15, NatA auxiliary subunit OS=Pongo abelii OX=9601 GN=NAA15 PE=2 SV=1	4.44E-47	41.985	91.92982456
P_KWMTBOMO08440	tr|Q5UAM6|Q5UAM6_BOMMO	Ribosomal protein S16 OS=Bombyx mori OX=7091 GN=RpS16 PE=2 SV=1	8.57E-109	100	100	sp|Q95V31|RS16_SPOFR	40S ribosomal protein S16 OS=Spodoptera frugiperda OX=7108 GN=RpS16 PE=2 SV=1	1.88E-104	98.013	100
P_KWMTBOMO08464	sp|P21828|FIBL_BOMMO	Fibroin light chain OS=Bombyx mori OX=7091 GN=FIBL PE=1 SV=1	0	100	100	sp|P21828|FIBL_BOMMO	Fibroin light chain OS=Bombyx mori OX=7091 GN=FIBL PE=1 SV=1	0	100	100
P_KWMTBOMO08578	tr|H9JDK4|H9JDK4_BOMMO	Multifunctional fusion protein OS=Bombyx mori OX=7091 GN=101741966 PE=3 SV=1	0	100	100	sp|Q7SY23|AL4A1_DANRE	Delta-1-pyrroline-5-carboxylate dehydrogenase, mitochondrial OS=Danio rerio OX=7955 GN=aldh4a1 PE=2 SV=1	0	55.159	94.33628319
P_KWMTBOMO08596	tr|H9JEM4|H9JEM4_BOMMO	Uncharacterized protein OS=Bombyx mori OX=7091 PE=3 SV=1	1.43E-162	100	97.69585253	sp|Q7SXP0|S22BB_DANRE	Vesicle-trafficking protein SEC22b-B OS=Danio rerio OX=7955 GN=sec22bb PE=2 SV=1	4.44E-87	56.621	100
P_KWMTBOMO08655	tr|Q2F649|Q2F649_BOMMO	Surfeit 4-like protein OS=Bombyx mori OX=7091 PE=2 SV=1	0	100	100	sp|O18405|SURF4_DROME	Surfeit locus protein 4 homolog OS=Drosophila melanogaster OX=7227 GN=Surf4 PE=2 SV=1	2.43E-168	81.852	100
P_KWMTBOMO08707	tr|H9JDR8|H9JDR8_BOMMO	Elongator complex protein 3 OS=Bombyx mori OX=7091 PE=3 SV=1	0	99.66	82.4684432	sp|Q5ZHS1|ELP3_CHICK	Elongator complex protein 3 OS=Gallus gallus OX=9031 GN=ELP3 PE=2 SV=1	0	84.962	74.61430575
P_KWMTBOMO08894	tr|Q5UAN9|Q5UAN9_BOMMO	Ribosomal protein S5 OS=Bombyx mori OX=7091 GN=RpS5 PE=2 SV=1	3.62E-166	100	100	sp|P46782|RS5_HUMAN	40S ribosomal protein S5 OS=Homo sapiens OX=9606 GN=RPS5 PE=1 SV=4	1.84E-134	91.327	89.49771689
P_KWMTBOMO08961	tr|Q5UAQ8|Q5UAQ8_BOMMO	60S ribosomal protein L30 OS=Bombyx mori OX=7091 GN=RpL30 PE=2 SV=1	2.87E-80	100	100	sp|P58375|RL30_SPOFR	60S ribosomal protein L30 OS=Spodoptera frugiperda OX=7108 GN=RpL30 PE=3 SV=1	9.75E-78	99.099	98.2300885
P_KWMTBOMO09055	tr|H9JDA5|H9JDA5_BOMMO	Proton-translocating NAD(P)(+) transhydrogenase OS=Bombyx mori OX=7091 PE=3 SV=1	0	93.082	100	sp|P11024|NNTM_BOVIN	NAD(P) transhydrogenase, mitochondrial OS=Bos taurus OX=9913 GN=NNT PE=1 SV=3	0	59.925	100
P_KWMTBOMO09183	tr|H9J1H4|H9J1H4_BOMMO	Uncharacterized protein OS=Bombyx mori OX=7091 PE=4 SV=1	0	99.804	46.6179159	sp|O00410|IPO5_HUMAN	Importin-5 OS=Homo sapiens OX=9606 GN=IPO5 PE=1 SV=4	0	52.516	99.90859232
P_KWMTBOMO09232	tr|Q5UAS9|Q5UAS9_BOMMO	Ribosomal protein L10A OS=Bombyx mori OX=7091 GN=RpL10A PE=2 SV=1	4.16E-162	100	100	sp|Q963B6|RL10A_SPOFR	60S ribosomal protein L10a OS=Spodoptera frugiperda OX=7108 GN=RpL10A PE=2 SV=1	1.14E-156	96.774	100
P_KWMTBOMO09250	tr|H9J1E3|H9J1E3_BOMMO	IU_nuc_hydro domain-containing protein OS=Bombyx mori OX=7091 PE=4 SV=1	0	98.442	96.3963964	--	--	--	--	--
P_KWMTBOMO09355	tr|Q6T3A7|Q6T3A7_BOMMO	Thiol peroxiredoxin OS=Bombyx mori OX=7091 GN=692638 PE=2 SV=1	5.60E-148	100	100	sp|Q9V3P0|PRDX1_DROME	Peroxiredoxin 1 OS=Drosophila melanogaster OX=7227 GN=Jafrac1 PE=1 SV=1	8.32E-117	81.152	97.94871795
P_KWMTBOMO09553	tr|Q1HPN7|Q1HPN7_BOMMO	Fructose-bisphosphate aldolase OS=Bombyx mori OX=7091 GN=778467 PE=2 SV=1	0	100	100	sp|P07764|ALF_DROME	Fructose-bisphosphate aldolase OS=Drosophila melanogaster OX=7227 GN=Ald1 PE=1 SV=5	0	82.143	100
P_KWMTBOMO09597	tr|H9JXD6|H9JXD6_BOMMO	Carrier domain-containing protein OS=Bombyx mori OX=7091 PE=3 SV=1	0	99.659	63.28293737	sp|Q63ZT8|AL1L1_XENTR	Cytosolic 10-formyltetrahydrofolate dehydrogenase OS=Xenopus tropicalis OX=8364 GN=aldh1l1 PE=2 SV=1	0	63.865	98.92008639
P_KWMTBOMO09631	tr|H9JTW9|H9JTW9_BOMMO	GOLD domain-containing protein OS=Bombyx mori OX=7091 GN=101737070 PE=3 SV=1	5.12E-155	100	100	sp|P49020|TMED2_CRIGR	Transmembrane emp24 domain-containing protein 2 (Fragment) OS=Cricetulus griseus OX=10029 GN=TMED2 PE=1 SV=1	4.26E-87	63.587	90.19607843
P_KWMTBOMO09672	tr|H9JTU8|H9JTU8_BOMMO	Uncharacterized protein OS=Bombyx mori OX=7091 PE=4 SV=1	7.15E-83	98.276	42.64705882	sp|P48738|PIPNA_RABIT	Phosphatidylinositol transfer protein alpha isoform OS=Oryctolagus cuniculus OX=9986 GN=PITPNA PE=3 SV=2	1.85E-115	59.928	100
P_KWMTBOMO09709	tr|H9JKR5|H9JKR5_BOMMO	CRAL-TRIO domain-containing protein OS=Bombyx mori OX=7091 GN=101736546 PE=4 SV=1	4.60E-53	36.226	100	sp|Q9D3D0|TTPAL_MOUSE	Alpha-tocopherol transfer protein-like OS=Mus musculus OX=10090 GN=Ttpal PE=1 SV=3	1.73E-10	26.111	68.44106464
P_KWMTBOMO09838	tr|H9JUC0|H9JUC0_BOMMO	Asparagine--tRNA ligase OS=Bombyx mori OX=7091 PE=3 SV=1	0	99.104	100	sp|Q2KJG3|SYNC_BOVIN	Asparagine--tRNA ligase, cytoplasmic OS=Bos taurus OX=9913 GN=NARS PE=2 SV=3	0	70.599	99.45848375
P_KWMTBOMO09876	tr|H9JUE4|H9JUE4_BOMMO	Uncharacterized protein OS=Bombyx mori OX=7091 PE=3 SV=1	9.18E-74	100	100	sp|O01725|RLA2_BRAFL	60S acidic ribosomal protein P2 OS=Branchiostoma floridae OX=7739 PE=3 SV=1	2.58E-28	56.034	100
P_KWMTBOMO10049	sp|Q5UAP8|RL38_BOMMO	60S ribosomal protein L38 OS=Bombyx mori OX=7091 GN=RpL38 PE=3 SV=1	8.84E-45	100	100	sp|Q5UAP8|RL38_BOMMO	60S ribosomal protein L38 OS=Bombyx mori OX=7091 GN=RpL38 PE=3 SV=1	5.76E-44	100	100
P_KWMTBOMO10128	tr|H9J806|H9J806_BOMMO	Uncharacterized protein OS=Bombyx mori OX=7091 PE=3 SV=1	1.66E-70	100	91.58878505	sp|Q9W141|ATPK_DROME	Putative ATP synthase subunit f, mitochondrial OS=Drosophila melanogaster OX=7227 GN=ATPsynF PE=3 SV=1	7.40E-60	75.701	100
P_KWMTBOMO10129	tr|Q2F5X3|Q2F5X3_BOMMO	26S proteasome non-ATPase regulatory subunit 4 OS=Bombyx mori OX=7091 GN=778515 PE=2 SV=1	0	100	100	sp|P55035|PSMD4_DROME	26S proteasome non-ATPase regulatory subunit 4 OS=Drosophila melanogaster OX=7227 GN=Rpn10 PE=1 SV=2	1.63E-145	60.677	100
P_KWMTBOMO10243	tr|H9JCR8|H9JCR8_BOMMO	Ubiquitin carboxyl-terminal hydrolase 7 OS=Bombyx mori OX=7091 PE=3 SV=1	0	95.544	100	sp|Q4VSI4|UBP7_RAT	Ubiquitin carboxyl-terminal hydrolase 7 OS=Rattus norvegicus OX=10116 GN=Usp7 PE=1 SV=1	0	60.572	96.69642857

P_KWMTBOMO10337	tr|H9JCI5|H9JCI5_BOMMO	ERF-3 OS=Bombyx mori OX=7091 PE=4 SV=1	0	100	96.32352941	sp|Q8R050|ERF3A_MOUSE	Eukaryotic peptide chain release factor GTP-binding subunit ERF3A OS=Mus musculus OX=10090 GN=Gspt1 PE=1 SV=2	0	66.599	90.25735294
P_KWMTBOMO10591	tr|Q6PUF7|Q6PUF7_BOMMO	40S ribosomal protein S24 OS=Bombyx mori OX=7091 GN=100101150 PE=2 SV=1	4.69E-94	100	100	sp|Q962Q6|RS24_SPOFR	40S ribosomal protein S24 OS=Spodoptera frugiperda OX=7108 GN=RpS24 PE=2 SV=1	2.80E-92	98.485	100
P_KWMTBOMO10779	tr|Q0N2R5|Q0N2R5_BOMMO	Signal recognition particle receptor alpha subunit OS=Bombyx mori OX=7091 PE=2 SV=1	0	99.51	100	sp|Q9U5L1|SRPRA_DROME	Signal recognition particle receptor subunit alpha homolog OS=Drosophila melanogaster OX=7227 GN=SrpRalpha PE=1 SV=2	0	59.486	100
P_KWMTBOMO10816	tr|H9JFZ5|H9JFZ5_BOMMO	Pyruvate carboxylase OS=Bombyx mori OX=7091 PE=4 SV=1	0	97.263	98.9001692	sp|P11498|PYC_HUMAN	Pyruvate carboxylase, mitochondrial OS=Homo sapiens OX=9606 GN=PC PE=1 SV=2	0	68.512	97.80033841
P_KWMTBOMO10916	tr|Q1HQ44|Q1HQ44_BOMMO	Proteasome subunit alpha type OS=Bombyx mori OX=7091 PE=2 SV=1	0	100	100	sp|O70435|PSA3_MOUSE	Proteasome subunit alpha type-3 OS=Mus musculus OX=10090 GN=Psma3 PE=1 SV=3	2.47E-130	65.49	100
P_KWMTBOMO10965	tr|Q1HQC6|Q1HQC6_BOMMO	26S protease regulatory subunit 6B OS=Bombyx mori OX=7091 PE=2 SV=1	0	100	100	sp|P46507|PRS6B_MANSE	26S proteasome regulatory subunit 6B OS=Manduca sexta OX=7130 PE=2 SV=1	0	98.313	100
P_KWMTBOMO11021	tr|Q5UAM3|Q5UAM3_BOMMO	Ribosomal protein S19 OS=Bombyx mori OX=7091 GN=RpS19 PE=2 SV=1	6.33E-111	100	100	sp|P39018|RS19A_DROME	40S ribosomal protein S19a OS=Drosophila melanogaster OX=7227 GN=RpS19a PE=1 SV=3	5.94E-71	66.892	96.73202614
P_KWMTBOMO11110	tr|Q0ZB74|Q0ZB74_BOMMO	Eukaryotic translation initiation factor 5 OS=Bombyx mori OX=7091 PE=2 SV=1	0	100	100	sp|Q9VXK6|IF5_DROME	Eukaryotic translation initiation factor 5 OS=Drosophila melanogaster OX=7227 GN=eIF5 PE=1 SV=1	3.65E-164	54.699	89.63282937
P_KWMTBOMO11161	tr|H9JGE8|H9JGE8_BOMMO	26S proteasome non-ATPase regulatory subunit 8 OS=Bombyx mori OX=7091 GN=101737946 PE=3 SV=1	0	100	100	sp|Q9CX56|PSMD8_MOUSE	26S proteasome non-ATPase regulatory subunit 8 OS=Mus musculus OX=10090 GN=Psmd8 PE=1 SV=2	3.67E-110	60.075	100
P_KWMTBOMO11202	tr|H9JT03|H9JT03_BOMMO	F-actin-capping protein subunit alpha OS=Bombyx mori OX=7091 GN=101738938 PE=3 SV=1	0	100	100	sp|Q9W2N0|CAPZA_DROME	F-actin-capping protein subunit alpha OS=Drosophila melanogaster OX=7227 GN=cpa PE=2 SV=1	1.31E-147	68.44	97.91666667
P_KWMTBOMO11416	tr|H9IXG0|H9IXG0_BOMMO	ATPase ASNA1 homolog OS=Bombyx mori OX=7091 GN=101735717 PE=3 SV=1	0	100	100	sp|B0WEV5|ASNA_CULQU	ATPase ASNA1 homolog OS=Culex quinquefasciatus OX=7176 GN=CPIJ005690 PE=3 SV=1	0	80.428	97.6119403
P_KWMTBOMO11583	tr|H9J3D6|H9J3D6_BOMMO	Cyclin-Q OS=Bombyx mori OX=7091 GN=101735856 PE=3 SV=1	0	100	100	sp|Q4QQW5|CCNQ_RAT	Cyclin-Q OS=Rattus norvegicus OX=10116 GN=Ccnq PE=2 SV=1	4.51E-56	41.441	88.8
P_KWMTBOMO11590	tr|H9J3D2|H9J3D2_BOMMO	Ku domain-containing protein OS=Bombyx mori OX=7091 GN=101736121 PE=4 SV=1	0	98.077	80.68965517	sp|O93257|XRCC6_CHICK	X-ray repair cross-complementing protein 5 OS=Gallus gallus OX=9031 GN=XRCC6 PE=2 SV=1	5.59E-42	26.126	95.68965517
P_KWMTBOMO11666	tr|Q2V0H5|Q2V0H5_BOMMO	Vesicle-fusing ATPase OS=Bombyx mori OX=7091 GN=ter94 PE=2 SV=1	0	100	100	sp|Q7KN62|TERA_DROME	Transitional endoplasmic reticulum ATPase TER94 OS=Drosophila melanogaster OX=7227 GN=TER94 PE=1 SV=1	0	88.025	100
P_KWMTBOMO11678	tr|Q5UAS7|Q5UAS7_BOMMO	60S ribosomal protein L12 OS=Bombyx mori OX=7091 GN=RpL12 PE=2 SV=1	1.46E-118	100	100	sp|P23358|RL12_RAT	60S ribosomal protein L12 OS=Rattus norvegicus OX=10116 GN=Rpl12 PE=2 SV=1	3.08E-96	81.25	97.56097561
P_KWMTBOMO11680	tr|H9J390|H9J390_BOMMO	Seryl-tRNA synthetase OS=Bombyx mori OX=7091 GN=101743384 PE=3 SV=1	0	100	100	sp|Q9GMB8|SYSC_BOVIN	Serine--tRNA ligase, cytoplasmic OS=Bos taurus OX=9913 GN=SARS1 PE=2 SV=3	0	69.495	100
P_KWMTBOMO11689	tr|H9J385|H9J385_BOMMO	Uncharacterized protein OS=Bombyx mori OX=7091 PE=4 SV=1	0	100	100	sp|O18373|SPS1_DROME	Inactive selenide, water dikinase-like protein OS=Drosophila melanogaster OX=7227 GN=SelD PE=2 SV=1	0	87.94	99.00497512
P_KWMTBOMO11987	tr|Q5UAN1|Q5UAN1_BOMMO	40S ribosomal protein S12 OS=Bombyx mori OX=7091 GN=RpS12 PE=2 SV=1	4.19E-99	100	100	sp|P80455|RS12_DROME	40S ribosomal protein S12 OS=Drosophila melanogaster OX=7227 GN=RpS12 PE=1 SV=2	1.09E-68	72.143	100
P_KWMTBOMO12018	tr|Q5UAN7|Q5UAN7_BOMMO	40S ribosomal protein S7 OS=Bombyx mori OX=7091 GN=RpS7 PE=2 SV=1	1.24E-137	99.474	100	sp|P48155|RS7_MANSE	40S ribosomal protein S7 OS=Manduca sexta OX=7130 GN=RpS7 PE=2 SV=1	1.42E-134	97.368	100
P_KWMTBOMO12094	tr|Q1HPM2|Q1HPM2_BOMMO	Phosphoenolpyruvate carboxykinase (GTP) OS=Bombyx mori OX=7091 PE=2 SV=1	0	99.365	99.84152139	sp|P20007|PCKG_DROME	Phosphoenolpyruvate carboxykinase [GTP] OS=Drosophila melanogaster OX=7227 GN=Pepck PE=2 SV=2	0	65.645	98.25673534
P_KWMTBOMO12189	tr|Q1HPX0|Q1HPX0_BOMMO	Cytochrome c oxidase polypeptide Vb OS=Bombyx mori OX=7091 PE=2 SV=1	1.68E-90	100	100	sp|Q710D6|COX5B_VULVU	Cytochrome c oxidase subunit 5B, mitochondrial OS=Vulpes vulpes OX=9627 GN=COX5B PE=1 SV=1	1.33E-27	44.248	92.62295082
P_KWMTBOMO12193	tr|H9JXB0|H9JXB0_BOMMO	COX6C domain-containing protein OS=Bombyx mori OX=7091 GN=101746562 PE=3 SV=1	1.23E-53	100	100	sp|Q7YRK7|COX6C_TARSY	Cytochrome c oxidase subunit 6C OS=Tarsius syrichta OX=1868482 GN=COX6C PE=3 SV=1	1.86E-18	53.623	88.46153846
P_KWMTBOMO12208	tr|H9JE23|H9JE23_BOMMO	SAC domain-containing protein OS=Bombyx mori OX=7091 PE=4 SV=1	3.33E-156	100	35.95317726	sp|Q9W0I6|SAC1_DROME	Phosphatidylinositol-3-phosphatase SAC1 OS=Drosophila melanogaster OX=7227 GN=Sac1 PE=2 SV=1	0	49.144	97.65886288
P_KWMTBOMO12285	tr|Q93137|Q93137_BOMMO	Reverse transcriptase OS=Bombyx mori OX=7091 PE=4 SV=1	0	71.082	49.23913043	sp|O17449|TBB1_MANSE	Tubulin beta-1 chain OS=Manduca sexta OX=7130 PE=2 SV=1	0	99.769	23.47826087
P_KWMTBOMO12400	tr|H9JSB8|H9JSB8_BOMMO	RRM domain-containing protein OS=Bombyx mori OX=7091 PE=4 SV=1	7.88E-54	100	70	--	--	--	--	--
P_KWMTBOMO12428	sp|Q5UAS2|RL17_BOMMO	60S ribosomal protein L17 OS=Bombyx mori OX=7091 GN=RpL17 PE=2 SV=1	5.32E-141	100	100	sp|Q5UAS2|RL17_BOMMO	60S ribosomal protein L17 OS=Bombyx mori OX=7091 GN=RpL17 PE=2 SV=1	3.47E-140	100	100
P_KWMTBOMO12448	tr|H9IYM5|H9IYM5_BOMMO	Secretory carrier-associated membrane protein OS=Bombyx mori OX=7091 PE=3 SV=1	1.69E-99	87.5	73.01587302	sp|Q8K021|SCAM1_MOUSE	Secretory carrier-associated membrane protein 1 OS=Mus musculus OX=10090 GN=Scamp1 PE=1 SV=1	8.88E-77	52.838	90.87301587
P_KWMTBOMO12461	tr|Q4JI71|Q4JI71_BOMMO	FK506-binding protein OS=Bombyx mori OX=7091 GN=FKBP45 PE=2 SV=1	0	100	100	sp|Q26486|FKBP4_SPOFR	46 kDa FK506-binding nuclear protein OS=Spodoptera frugiperda OX=7108 GN=FKBP46 PE=2 SV=1	0	71.838	100
P_KWMTBOMO12483	tr|Q66SV9|Q66SV9_BOMMO	40S ribosomal protein S15 OS=Bombyx mori OX=7091 GN=S15 PE=2 SV=1	9.24E-107	100	100	sp|P62844|RS15_PIG	40S ribosomal protein S15 OS=Sus scrofa OX=9823 GN=RPS15 PE=1 SV=2	2.23E-77	81.633	100
P_KWMTBOMO12602	tr|H9IWB5|H9IWB5_BOMMO	Uncharacterized protein OS=Bombyx mori OX=7091 PE=3 SV=1	0	95.142	67.58824981	sp|P37276|DYHC_DROME	Dynein heavy chain, cytoplasmic OS=Drosophila melanogaster OX=7227 GN=Dhc64C PE=2 SV=2	0	79.96	99.77783263

P_KWMTBOMO12628	tr|H9IWC7|H9IWC7_BOMMO	Isoleucyl-tRNA synthetase OS=Bombyx mori OX=7091 GN=101742308 PE=3 SV=1	0	99.835	100	sp|Q8BU30|SYIC_MOUSE	Isoleucine--tRNA ligase, cytoplasmic OS=Mus musculus OX=10090 GN=Iars1 PE=1 SV=2	0	60.246	100
P_KWMTBOMO12706	tr|Q5UAQ2|Q5UAQ2_BOMMO	60S ribosomal protein L36 OS=Bombyx mori OX=7091 GN=RpL36 PE=2 SV=1	1.02E-81	100	100	sp|P49630|RL36_DROME	60S ribosomal protein L36 OS=Drosophila melanogaster OX=7227 GN=RpL36 PE=1 SV=1	4.53E-50	71.304	96.63865546
P_KWMTBOMO12878	tr|Q0KIX8|Q0KIX8_BOMMO	Glutamate synthase OS=Bombyx mori OX=7091 GN=BmGOGAT PE=2 SV=1	0	99.658	100	sp|Q18164|DPYD_CAEEL	Dihydropyrimidine dehydrogenase [NADP(+)] OS=Caenorhabditis elegans OX=6239 GN=dpyd-1 PE=3 SV=2	2.57E-20	29.206	15.39589443
P_KWMTBOMO12965	tr|H9IT68|H9IT68_BOMMO	Vesicle-fusing ATPase OS=Bombyx mori OX=7091 PE=3 SV=1	0	99.828	55.48141087	sp|P46461|NSF1_DROME	Vesicle-fusing ATPase 1 OS=Drosophila melanogaster OX=7227 GN=comt PE=2 SV=1	0	72.851	69.87607245
P_KWMTBOMO13370	tr|H9JN38|H9JN38_BOMMO	Signal recognition particle subunit SRP72 OS=Bombyx mori OX=7091 PE=3 SV=1	0	100	66.30602782	sp|O76094|SRP72_HUMAN	Signal recognition particle subunit SRP72 OS=Homo sapiens OX=9606 GN=SRP72 PE=1 SV=3	7.11E-121	37.721	96.29057187
P_KWMTBOMO13520	tr|Q2F5T2|Q2F5T2_BOMMO	Calmodulin OS=Bombyx mori OX=7091 PE=2 SV=1	5.64E-106	100	100	sp|P62153|CALMA_HALRO	Calmodulin-A OS=Halocynthia roretzi OX=7729 PE=1 SV=2	3.68E-105	100	100
P_KWMTBOMO13597	tr|H9JN56|H9JN56_BOMMO	GOLD domain-containing protein OS=Bombyx mori OX=7091 PE=3 SV=1	8.08E-165	100	100	sp|B4NKL0|TMEDE_DROWI	Transmembrane emp24 domain-containing protein eca OS=Drosophila willistoni OX=7260 GN=eca PE=3 SV=1	1.07E-127	80.952	96.77419355
P_KWMTBOMO13722	tr|H9JPQ0|H9JPQ0_BOMMO	Uncharacterized protein OS=Bombyx mori OX=7091 PE=3 SV=1	2.24E-39	100	73.56321839	sp|Q99417|MYCBP_HUMAN	c-Myc-binding protein OS=Homo sapiens OX=9606 GN=MYCBP PE=1 SV=3	2.31E-23	56.944	82.75862069
P_KWMTBOMO13723	tr|Q1HPX3|Q1HPX3_BOMMO	ATP synthase subunit O, mitochondrial OS=Bombyx mori OX=7091 PE=2 SV=1	1.17E-152	100	100	sp|Q24439|ATPO_DROME	ATP synthase subunit O, mitochondrial OS=Drosophila melanogaster OX=7227 GN=ATPsynO PE=2 SV=2	7.56E-89	61.692	96.1722488
P_KWMTBOMO13729	tr|Q684K3|Q684K3_BOMMO	Protein-synthesizing GTPase OS=Bombyx mori OX=7091 GN=eIF2g PE=2 SV=1	0	100	100	sp|Q24208|IF2G_DROME	Eukaryotic translation initiation factor 2 subunit 3 OS=Drosophila melanogaster OX=7227 GN=eIF2gamma PE=2 SV=1	0	84.12	99.57264957
P_KWMTBOMO13774	tr|Q2F5W7|Q2F5W7_BOMMO	Ras-related GTP-binding protein 4b OS=Bombyx mori OX=7091 GN=692901 PE=2 SV=1	6.54E-160	100	100	sp|Q68EK7|RAB4B_DANRE	Ras-related protein Rab-4B OS=Danio rerio OX=7955 GN=rab4b PE=2 SV=1	5.07E-117	74.648	99.53271028
P_KWMTBOMO13800	tr|H9JPL2|H9JPL2_BOMMO	60S ribosomal protein L18a OS=Bombyx mori OX=7091 PE=3 SV=1	4.32E-132	100	100	sp|Q8WQI7|RL18A_SPOFR	60S ribosomal protein L18a OS=Spodoptera frugiperda OX=7108 GN=RpL18A PE=2 SV=1	1.32E-126	95.48	100
P_KWMTBOMO14041	tr|Q2F5J1|Q2F5J1_BOMMO	26S proteasome non-ATPase regulatory subunit 13 OS=Bombyx mori OX=7091 PE=2 SV=1	0	100	100	sp|B0BN93|PSD13_RAT	26S proteasome non-ATPase regulatory subunit 13 OS=Rattus norvegicus OX=10116 GN=Psmd13 PE=1 SV=1	1.26E-102	46.921	88.57142857
P_KWMTBOMO14157	tr|H9JNS6|H9JNS6_BOMMO	Coatomer subunit gamma OS=Bombyx mori OX=7091 GN=692402 PE=3 SV=1	0	99.768	100	sp|Q29AE5|COPG_DROPS	Coatomer subunit gamma OS=Drosophila pseudoobscura pseudoobscura OX=46245 GN=gammaCop PE=3 SV=1	0	58.75	100
P_KWMTBOMO14160	tr|H9JPF8|H9JPF8_BOMMO	Iso_dh domain-containing protein OS=Bombyx mori OX=7091 PE=3 SV=1	0	100	100	sp|Q58CP0|IDH3G_BOVIN	Isocitrate dehydrogenase [NAD] subunit gamma, mitochondrial OS=Bos taurus OX=9913 GN=IDH3G PE=2 SV=1	5.17E-132	50.653	98.71134021
P_KWMTBOMO14478	tr|H9IS48|H9IS48_BOMMO	Uncharacterized protein OS=Bombyx mori OX=7091 GN=101742289 PE=3 SV=1	1.04E-66	100	100	sp|Q9W1V3|FBRL_DROME	rRNA 2'-O-methyltransferase fibrillarin OS=Drosophila melanogaster OX=7227 GN=Fib PE=2 SV=1	4.31E-61	94.737	100
P_KWMTBOMO14530	tr|Q2F5R7|Q2F5R7_BOMMO	Eukaryotic peptide chain release factor subunit 1 OS=Bombyx mori OX=7091 PE=2 SV=1	0	99.314	100	sp|Q9VPH7|ERF1_DROME	Eukaryotic peptide chain release factor subunit 1 OS=Drosophila melanogaster OX=7227 GN=eRF1 PE=1 SV=2	0	93.822	100
P_KWMTBOMO14639	tr|Q5UAQ6|Q5UAQ6_BOMMO	60S ribosomal protein L32 OS=Bombyx mori OX=7091 GN=RpL32 PE=2 SV=1	1.84E-97	100	100	sp|Q962T1|RL32_SPOFR	60S ribosomal protein L32 OS=Spodoptera frugiperda OX=7108 GN=RpL32 PE=2 SV=1	6.75E-95	97.761	100
P_KWMTBOMO14715	tr|Q1HPL2|Q1HPL2_BOMMO	Phosphate carrier 1 OS=Bombyx mori OX=7091 GN=PiC PE=2 SV=1	0	100	100	sp|O61703|MPCP_CHOFU	Phosphate carrier protein, mitochondrial OS=Choristoneura fumiferana OX=7141 PE=2 SV=1	0	78.771	100
P_KWMTBOMO14761	tr|H9J2S8|H9J2S8_BOMMO	Dihydrolipoyllysine-residue succinyltransferase component of 2-oxoglutarate dehydrogenase complex, mitochondrial OS=Bombyx mori OX=7091 PE=3 SV=1	0	78.71	100	sp|P11179|ODO2_BOVIN	Dihydrolipoyllysine-residue succinyltransferase component of 2-oxoglutarate dehydrogenase complex, mitochondrial OS=Bos taurus OX=9913 GN=DLST PE=1 SV=2	8.56E-150	56.542	92.04301075
P_KWMTBOMO14845	tr|Q1HQC8|Q1HQC8_BOMMO	ARP1 actin-related protein 1-like protein A OS=Bombyx mori OX=7091 GN=732868 PE=2 SV=1	0	100	100	sp|P61162|ACTZ_CANLF	Alpha-centractin OS=Canis lupus familiaris OX=9615 GN=ACTR1A PE=2 SV=1	0	81.649	100
P_KWMTBOMO14887	tr|H9JRM2|H9JRM2_BOMMO	T-complex protein 1 subunit eta OS=Bombyx mori OX=7091 PE=3 SV=1	0	99.631	100	sp|Q5ZJK8|TCPH_CHICK	T-complex protein 1 subunit eta OS=Gallus gallus OX=9031 GN=CCT7 PE=1 SV=1	0	69.376	97.60147601
P_KWMTBOMO14942	tr|H9IU76|H9IU76_BOMMO	AMP deaminase OS=Bombyx mori OX=7091 PE=3 SV=1	0	98.229	97.60638298	sp|Q02356|AMPD2_RAT	AMP deaminase 2 OS=Rattus norvegicus OX=10116 GN=Ampd2 PE=1 SV=2	0	59.916	95.21276596
P_KWMTBOMO15060	tr|H9J5R2|H9J5R2_BOMMO	Protein transport protein SEC23 OS=Bombyx mori OX=7091 PE=3 SV=1	0	100	100	sp|Q01405|SC23A_MOUSE	Protein transport protein Sec23A OS=Mus musculus OX=10090 GN=Sec23a PE=1 SV=2	0	74.583	100
P_KWMTBOMO15121	tr|Q0N2R8|Q0N2R8_BOMMO	Signal recognition particle 54 kDa protein OS=Bombyx mori OX=7091 GN=SRP54 PE=2 SV=1	0	99.601	100	sp|Q7ZVN5|SRP54_DANRE	Signal recognition particle 54 kDa protein OS=Danio rerio OX=7955 GN=srp54 PE=2 SV=1	0	80.2	99.8003992
P_KWMTBOMO15125	tr|H9J651|H9J651_BOMMO	Adipocyte plasma membrane-associated protein OS=Bombyx mori OX=7091 PE=3 SV=1	0	99.398	94.32624113	sp|Q5ZIF1|APMAP_CHICK	Adipocyte plasma membrane-associated protein OS=Gallus gallus OX=9031 GN=APMAP PE=2 SV=1	2.85E-45	32.203	50.21276596
P_KWMTBOMO15179	tr|G9I6Y0|G9I6Y0_BOMMO	RING-type E3 ubiquitin transferase OS=Bombyx mori OX=7091 GN=100862840 PE=2 SV=1	0	85.632	100	sp|Q803I8|SYVN1_DANRE	E3 ubiquitin-protein ligase synoviolin OS=Danio rerio OX=7955 GN=syvn1 PE=2 SV=2	3.34E-177	67.761	64.17624521
P_KWMTBOMO15198	tr|B5AU20|B5AU20_BOMMO	UDP-glucuronosyltransferase OS=Bombyx mori OX=7091 GN=UGT2 PE=2 SV=1	0	100	100	sp|A0A291PQH4|UGT2_DACCO	UDP-glucosyltransferase 2 OS=Dactylopius coccus OX=765876 GN=UTG2 PE=1 SV=1	2.31E-59	29.352	94.0952381
P_KWMTBOMO15243	tr|H9J673|H9J673_BOMMO	Uncharacterized protein OS=Bombyx mori OX=7091 PE=3 SV=1	0	94.172	100	sp|Q6NRP2|PSME4_XENLA	Proteasome activator complex subunit 4 OS=Xenopus laevis OX=8355 GN=psme4 PE=2 SV=1	0	34.078	81.69968717

P_KWMTBOMO15357		tr|H9J6H5|H9J6H5_BOMMO	Prolyl-tRNA synthetase OS=Bombyx mori OX=7091 PE=3 SV=1	0	98.315	99.64028777		sp|P28668|SYEP_DROME	Bifunctional glutamate/proline--tRNA ligase OS=Drosophila melanogaster OX=7227 GN=GluProRS PE=1 SV=2	0	61.306	99.16067146
P_KWMTBOMO15361		tr|O16143|O16143_BOMMO	Rab1 protein OS=Bombyx mori OX=7091 GN=rabB PE=2 SV=1	5.31E-153	100	100		sp|Q05974|RAB1A_LYMST	Ras-related protein Rab-1A OS=Lymnaea stagnalis OX=6523 GN=RAB1A PE=2 SV=1	4.74E-124	81.188	100
P_KWMTBOMO15365		tr|B0FRJ4|B0FRJ4_BOMMO	Fibroin heavy chain Fib-h (Fragment) OS=Bombyx mori OX=7091 PE=4 SV=1	4.87E-120	99.351	2.943987765		sp|Q99050|FIBH_BOMMA	Fibroin heavy chain (Fragment) OS=Bombyx mandarina OX=7092 GN=FIBH PE=2 SV=2	5.97E-138	97.765	3.421907857
P_KWMTBOMO15407		tr|Q1HQA9|Q1HQA9_BOMMO	Transmembrane trafficking protein OS=Bombyx mori OX=7091 GN=733098 PE=2 SV=1	1.47E-154	100	100		sp|B3LVB0|TMEDA_DROAN	Transmembrane emp24 domain-containing protein bai OS=Drosophila ananassae OX=7217 GN=bai PE=3 SV=1	6.57E-105	69.5	97.56097561
P_KWMTBOMO15419		tr|H9J5I6|H9J5I6_BOMMO	Methyltransf_25 domain-containing protein OS=Bombyx mori OX=7091 PE=4 SV=1	0	100	99.15730337		sp|Q6PAK3|ANM8_MOUSE	Protein arginine N-methyltransferase 8 OS=Mus musculus OX=10090 GN=Prmt8 PE=1 SV=2	0	71.988	93.25842697
P_KWMTBOMO15518		tr|Q1HPL0|Q1HPL0_BOMMO	26S proteasome regulatory ATPase subunit 10B OS=Bombyx mori OX=7091 PE=2 SV=1	0	100	100		sp|P62334|PRS10_MOUSE	26S proteasome regulatory subunit 10B OS=Mus musculus OX=10090 GN=Psmc6 PE=1 SV=1	0	88.571	97.22222222
P_KWMTBOMO15547		tr|H9JMI6|H9JMI6_BOMMO	Nop domain-containing protein OS=Bombyx mori OX=7091 PE=3 SV=1	0	91.939	99.35064935		sp|O00567|NOP56_HUMAN	Nucleolar protein 56 OS=Homo sapiens OX=9606 GN=NOP56 PE=1 SV=4	0	66.59	93.93939394
P_KWMTBOMO15665		tr|H9IYF4|H9IYF4_BOMMO	Uncharacterized protein OS=Bombyx mori OX=7091 GN=101736745 PE=4 SV=1	0	100	100		sp|P26305|LPSBP_PERAM	Hemolymph lipopolysaccharide-binding protein OS=Periplaneta americana OX=6978 PE=1 SV=1	3.37E-26	39.716	45.19230769
P_KWMTBOMO15675		tr|H9IYG1|H9IYG1_BOMMO	Uncharacterized protein OS=Bombyx mori OX=7091 PE=4 SV=1	0	100	56.60377358		sp|Q2M296|MTHSD_HUMAN	Methenyltetrahydrofolate synthase domain-containing protein OS=Homo sapiens OX=9606 GN=MTHFSD PE=1 SV=2	5.55E-81	40.212	79.24528302
P_KWMTBOMO15712		tr|Q2F5N9|Q2F5N9_BOMMO	Nucleoplasmin isoform 2 OS=Bombyx mori OX=7091 GN=692956 PE=2 SV=1	7.05E-137	100	100		sp|Q27415|NLP_DROME	Nucleoplasmin-like protein OS=Drosophila melanogaster OX=7227 GN=Nlp PE=1 SV=1	2.34E-29	48.148	57.7540107
P_KWMTBOMO15757		tr|B3VBE3|B3VBE3_BOMMO	Vacuolar proton pump subunit B OS=Bombyx mori OX=7091 PE=2 SV=1	5.34E-157	100	100		sp|P31401|VATB_MANSE	V-type proton ATPase subunit B OS=Manduca sexta OX=7130 GN=VHA55 PE=2 SV=1	2.90E-155	99.526	100
P_KWMTBOMO15781		tr|H9IYL2|H9IYL2_BOMMO	Uncharacterized protein OS=Bombyx mori OX=7091 PE=3 SV=2	0	87.5	100		sp|O46036|CTBP_DROME	C-terminal-binding protein OS=Drosophila melanogaster OX=7227 GN=CtBP PE=1 SV=3	0	91.643	78.9010989
P_KWMTBOMO16000		tr|A0A1Q0AL85|A0A1Q0AL85_BOMMO	Homeostasis protein OS=Bombyx mori OX=7091 GN=IHoP PE=2 SV=1	0	100	100		sp|Q61233|PLSL_MOUSE	Plastin-2 OS=Mus musculus OX=10090 GN=Lcp1 PE=1 SV=4	0	51.823	91.05339105
P_KWMTBOMO16197		tr|Q1HPX4|Q1HPX4_BOMMO	ATP synthase subunit gamma OS=Bombyx mori OX=7091 GN=732965 PE=2 SV=1	0	100	100		sp|O01666|ATPG_DROME	ATP synthase subunit gamma, mitochondrial OS=Drosophila melanogaster OX=7227 GN=ATPsyngamma PE=2 SV=2	2.51E-151	73.05	95.27027027
P_KWMTBOMO16204		tr|H9JWQ7|H9JWQ7_BOMMO	CHK domain-containing protein OS=Bombyx mori OX=7091 GN=101744771 PE=4 SV=1	0	100	100		--	--	--	--	--
P_KWMTBOMO16261		tr|Q2F6A0|Q2F6A0_BOMMO	Exuperantia OS=Bombyx mori OX=7091 PE=2 SV=1	0	100	100		sp|Q24747|EXU_DROVI	Maternal protein exuperantia OS=Drosophila virilis OX=7244 GN=exu PE=3 SV=1	5.40E-82	39.231	94.43099274
P_KWMTBOMO16319		tr|Q2F5K7|Q2F5K7_BOMMO	Receptor expression-enhancing protein OS=Bombyx mori OX=7091 PE=2 SV=1	3.16E-130	99.429	100		sp|Q29RM3|REEP5_BOVIN	Receptor expression-enhancing protein 5 OS=Bos taurus OX=9913 GN=REEP5 PE=2 SV=1	3.07E-59	50.595	96
P_KWMTBOMO16375		tr|H9JW87|H9JW87_BOMMO	Uncharacterized protein OS=Bombyx mori OX=7091 PE=3 SV=1	3.73E-150	100	93.69369369		sp|Q5XGW6|CHM4B_XENLA	Charged multivesicular body protein 4b OS=Xenopus laevis OX=8355 GN=chmp4b PE=2 SV=1	3.62E-77	62.054	100
P_KWMTBOMO16481		tr|Q19KB8|Q19KB8_BOMMO	Ras-related GTP-binding protein Rab11 OS=Bombyx mori OX=7091 GN=733063 PE=2 SV=1	1.33E-160	100	100		sp|P62492|RB11A_MOUSE	Ras-related protein Rab-11A OS=Mus musculus OX=10090 GN=Rab11a PE=1 SV=3	7.02E-129	82.326	100
P_KWMTBOMO16600		tr|H9J1F3|H9J1F3_BOMMO	acidPPc domain-containing protein OS=Bombyx mori OX=7091 PE=4 SV=1	1.70E-144	99.502	93.05555556		sp|Q66H88|PLPP6_RAT	Phospholipid phosphatase 6 OS=Rattus norvegicus OX=10116 GN=Plpp6 PE=2 SV=1	1.53E-18	35.754	82.87037037

unique PIPs of fibL at M4

Name	Bombyx-mori	Subject ID	Description	e-value	identity(%)	coverage(%)	Metazoa	Subject ID	Description	e-value	identity(%)	coverage(%)
P_KWMTBOMO00339		tr|H9ITE6|H9ITE6_BOMMO	CSD_1 domain-containing protein OS=Bombyx mori OX=7091 PE=4 SV=1	0	100	100		sp|P41824|YBOXH_APLCA	Y-box factor homolog OS=Aplysia californica OX=6500 PE=2 SV=1	1.24E-50	69.106	47.49034749
P_KWMTBOMO00437		tr|H9IU13|H9IU13_BOMMO	PHB domain-containing protein OS=Bombyx mori OX=7091 PE=3 SV=1	0	100	98.82629108		sp|O61491|FLOT1_DROME	Flotillin-1 OS=Drosophila melanogaster OX=7227 GN=Flo1 PE=2 SV=1	0	83.059	99.76525822
P_KWMTBOMO00497		tr|H9JRZ9|H9JRZ9_BOMMO	Uncharacterized protein OS=Bombyx mori OX=7091 PE=3 SV=1	0	100	100		sp|Q60HD7|SERA_MACFA	D-3-phosphoglycerate dehydrogenase OS=Macaca fascicularis OX=9541 GN=PHGDH PE=2 SV=4	2.42E-109	50.814	93.88379205
P_KWMTBOMO00545		tr|A7LBQ0|A7LBQ0_BOMMO	L-lactate dehydrogenase OS=Bombyx mori OX=7091 GN=LDH PE=2 SV=1	0	100	95.66473988		sp|Q95028|LDH_DROME	L-lactate dehydrogenase OS=Drosophila melanogaster OX=7227 GN=Ldh PE=2 SV=1	1.96E-164	65.964	95.95375723
P_KWMTBOMO00638		tr|D2Y4R4|D2Y4R4_BOMMO	Coatomer subunit beta' OS=Bombyx mori OX=7091 GN=COPB2 PE=2 SV=1	0	99.877	99.26918392		sp|O62621|COPB2_DROME	Coatomer subunit beta' OS=Drosophila melanogaster OX=7227 GN=beta'COP PE=2 SV=2	0	71.059	98.90377588
P_KWMTBOMO00687		tr|H9J134|H9J134_BOMMO	Nucleolar GTP-binding protein 1 OS=Bombyx mori OX=7091 PE=4 SV=1	0	93.029	64.59627329		sp|Q9V411|NOG1_DROME	Nucleolar GTP-binding protein 1 OS=Drosophila melanogaster OX=7227 GN=Non1 PE=2 SV=1	0	68.666	100
P_KWMTBOMO00834		tr|H9JJI4|H9JJI4_BOMMO	RRM domain-containing protein OS=Bombyx mori OX=7091 GN=101746268 PE=4 SV=1	0	100	100		--	--	--	--	--

P_KWMTBOMO00881	tr|D4QF47|D4QF47_BOMMO	Ced-6 protein OS=Bombyx mori OX=7091 GN=ced-6 PE=2 SV=1	0	100	100	sp|Q7JUY7|CED6_DROME	PTB domain-containing adapter protein ced-6 OS=Drosophila melanogaster OX=7227 GN=ced-6 PE=1 SV=1	3.82E-84	39.463	94.34697856
P_KWMTBOMO01320	tr|H9JH84|H9JH84_BOMMO	Uncharacterized protein OS=Bombyx mori OX=7091 PE=3 SV=1	3.35E-146	100	100	sp|P46222|RL11_DROME	60S ribosomal protein L11 OS=Drosophila melanogaster OX=7227 GN=RpL11 PE=1 SV=2	4.44E-121	91.011	91.28205128
P_KWMTBOMO01502	tr|Q5CCJ4|Q5CCJ4_BOMMO	Glutathione S-transferase sigma OS=Bombyx mori OX=7091 GN=GST sigma PE=1 SV=1	3.95E-152	100	100	sp|P46429|GST2_MANSE	Glutathione S-transferase 2 OS=Manduca sexta OX=7130 GN=GST2 PE=2 SV=1	8.62E-95	66.176	100
P_KWMTBOMO01918	tr|Q2F5L5|Q2F5L5_BOMMO	Protein transport protein Sec61 subunit beta OS=Bombyx mori OX=7091 GN=733070 PE=2 SV=1	1.66E-65	100	100	sp|P60467|SC61B_CANLF	Protein transport protein Sec61 subunit beta OS=Canis lupus familiaris OX=9615 GN=SEC61B PE=1 SV=2	2.67E-41	71.277	96.90721649
P_KWMTBOMO01927	sp|P21894|SYAC_BOMMO	Alanine--tRNA ligase, cytoplasmic OS=Bombyx mori OX=7091 PE=1 SV=1	0	99.276	100	sp|P21894|SYAC_BOMMO	Alanine--tRNA ligase, cytoplasmic OS=Bombyx mori OX=7091 PE=1 SV=1	0	99.276	100
P_KWMTBOMO02059	tr|Q5UAM4|Q5UAM4_BOMMO	40S ribosomal protein S18 OS=Bombyx mori OX=7091 GN=RpS18 PE=2 SV=1	1.29E-111	100	100	sp|Q962R1|RS18_SPOFR	40S ribosomal protein S18 OS=Spodoptera frugiperda OX=7108 GN=RpS18 PE=2 SV=1	1.23E-110	99.342	100
P_KWMTBOMO02061	tr|Q1HPU7|Q1HPU7_BOMMO	Adenosylhomocysteinase OS=Bombyx mori OX=7091 PE=2 SV=1	0	100	100	sp|O76757|SAHH_ANOGA	Adenosylhomocysteinase OS=Anopheles gambiae OX=7165 GN=Ahcy13 PE=2 SV=2	0	80.233	100
P_KWMTBOMO02431	tr|Q1HQ25|Q1HQ25_BOMMO	ATP synthase subunit OS=Bombyx mori OX=7091 GN=732934 PE=2 SV=1	2.04E-70	100	100	sp|Q5RFH0|ATP5L_PONAB	ATP synthase subunit g, mitochondrial OS=Pongo abelii OX=9601 GN=ATP5MG PE=3 SV=1	1.02E-32	52.632	95.95959596
P_KWMTBOMO02748	tr|H9J2I1|H9J2I1_BOMMO	TLC domain-containing protein OS=Bombyx mori OX=7091 GN=101745731 PE=3 SV=1	0	100	100	sp|Q6DED0|TR1L1_XENLA	Translocating chain-associated membrane protein 1-like 1 OS=Xenopus laevis OX=8355 GN=tram1l1 PE=2 SV=1	1.09E-74	35.979	100
P_KWMTBOMO02840	sp|Q2F5R8|EIF3E_BOMMO	Eukaryotic translation initiation factor 3 subunit E OS=Bombyx mori OX=7091 GN=eIF3-S6 PE=2 SV=1	0	100	100	sp|Q2F5R8|EIF3E_BOMMO	Eukaryotic translation initiation factor 3 subunit E OS=Bombyx mori OX=7091 GN=eIF3-S6 PE=2 SV=1	0	100	100
P_KWMTBOMO02857	tr|O17508|O17508_BOMMO	Cdc2-related kinase OS=Bombyx mori OX=7091 PE=2 SV=1	0	100	100	sp|Q3UMM4|CDK10_MOUSE	Cyclin-dependent kinase 10 OS=Mus musculus OX=10090 GN=Cdk10 PE=1 SV=1	1.53E-171	69.486	81.93069307
P_KWMTBOMO03005	tr|H9J271|H9J271_BOMMO	Elongation factor 1-alpha OS=Bombyx mori OX=7091 GN=EF1a PE=2 SV=1	0	100	100	sp|P29520|EF1A_BOMMO	Elongation factor 1-alpha OS=Bombyx mori OX=7091 PE=2 SV=1	0	99.784	100
P_KWMTBOMO03122	tr|L8B3Q2|L8B3Q2_BOMMO	Kinesin-like protein OS=Bombyx mori OX=7091 GN=MCAK PE=2 SV=1	0	100	100	sp|Q960Z0|KI10A_DROME	Kinesin-like protein Klp10A OS=Drosophila melanogaster OX=7227 GN=Klp10A PE=1 SV=1	0	62.086	82.17687075
P_KWMTBOMO03169	tr|Q17201|Q17201_BOMMO	Bmsqd-2 OS=Bombyx mori OX=7091 PE=2 SV=1	0	99.34	100	sp|Q08473|SQD_DROME	RNA-binding protein squid OS=Drosophila melanogaster OX=7227 GN=sqd PE=1 SV=3	4.44E-100	72.683	67.65676568
P_KWMTBOMO03173	tr|Q1HPV7|Q1HPV7_BOMMO	Histone H2A OS=Bombyx mori OX=7091 GN=h2a.z PE=2 SV=1	1.20E-88	100	100	sp|Q6Y237|H2AV_PAGMA	Histone H2A.V OS=Pagrus major OX=143350 GN=h2az2 PE=2 SV=3	3.37E-81	97.561	95.34883721
P_KWMTBOMO03314	tr|Q5UAS3|Q5UAS3_BOMMO	Ribosomal protein L15 OS=Bombyx mori OX=7091 GN=RpL15 PE=2 SV=1	2.87E-148	100	100	sp|P30736|RL15_CHITE	60S ribosomal protein L15 OS=Chironomus tentans OX=7153 GN=RpL15 PE=3 SV=3	3.13E-122	80.392	100
P_KWMTBOMO03337	tr|H9JA60|H9JA60_BOMMO	Uncharacterized protein OS=Bombyx mori OX=7091 PE=4 SV=1	0	98.697	96.84542587	sp|P21522|ROA1_SCHAM	Heterogeneous nuclear ribonucleoprotein A1, A2/B1 homolog OS=Schistocerca americana OX=7009 GN=HNRNP PE=2 SV=1	1.83E-91	74.857	55.20504732
P_KWMTBOMO03550	tr|H9J9U5|H9J9U5_BOMMO	Chromo domain-containing protein OS=Bombyx mori OX=7091 PE=4 SV=1	1.41E-72	82.09	51.14503817	sp|Q13185|CBX3_HUMAN	Chromobox protein homolog 3 OS=Homo sapiens OX=9606 GN=CBX3 PE=1 SV=4	2.25E-36	46.309	56.87022901
P_KWMTBOMO03601	tr|S5RN30|S5RN30_BOMMO	Farnesoic acid O-methyltransferase OS=Bombyx mori OX=7091 GN=FaMeT6-1 PE=2 SV=1	6.39E-110	100	100	sp|Q66S13|NATT4_THANI	Natterin-4 OS=Thalassophryne nattereri OX=289382 PE=2 SV=1	5.91E-07	31.061	89.79591837
P_KWMTBOMO03630	tr|H9J9R7|H9J9R7_BOMMO	Uncharacterized protein OS=Bombyx mori OX=7091 PE=4 SV=1	2.53E-122	74.359	44.17475728	sp|Q7TMK9|HNRPQ_MOUSE	Heterogeneous nuclear ribonucleoprotein Q OS=Mus musculus OX=10090 GN=Syncrip PE=1 SV=2	1.08E-179	55.031	78.802589
P_KWMTBOMO03631	tr|H9J9Y8|H9J9Y8_BOMMO	ER membrane protein complex subunit 1 OS=Bombyx mori OX=7091 PE=3 SV=1	0	100	93.14845024	sp|Q5ZL00|EMC1_CHICK	ER membrane protein complex subunit 1 OS=Gallus gallus OX=9031 GN=EMC1 PE=2 SV=1	5.23E-32	32.143	45.67699837
P_KWMTBOMO03748	tr|Q2F645|Q2F645_BOMMO	Transketolase OS=Bombyx mori OX=7091 PE=2 SV=1	0	100	100	sp|Q9D4D4|TKTL2_MOUSE	Transketolase-like protein 2 OS=Mus musculus OX=10090 GN=Tktl2 PE=1 SV=1	0	62.153	98.55305466
P_KWMTBOMO04505	tr|H9J7F7|H9J7F7_BOMMO	Zinc-hook domain-containing protein OS=Bombyx mori OX=7091 PE=3 SV=1	0	99.3	85.54319932	sp|Q9W252|RAD50_DROME	DNA repair protein RAD50 OS=Drosophila melanogaster OX=7227 GN=rad50 PE=2 SV=4	1.59E-155	31.037	100
P_KWMTBOMO04691	tr|Q5TLD1|Q5TLD1_BOMMO	Replication protein A small subunit OS=Bombyx mori OX=7091 GN=BmRPA3 PE=2 SV=1	2.98E-98	100	100	--	--	--	--	--
P_KWMTBOMO05068	tr|Q5UAR4|Q5UAR4_BOMMO	Ribosomal protein L24 OS=Bombyx mori OX=7091 GN=RpL24 PE=2 SV=1	2.43E-111	100	100	sp|Q6F444|RL24_PLUXY	60S ribosomal protein L24 OS=Plutella xylostella OX=51655 GN=RpL24 PE=2 SV=1	1.72E-90	95.484	100
P_KWMTBOMO05572	tr|H9JAP3|H9JAP3_BOMMO	Uncharacterized protein OS=Bombyx mori OX=7091 PE=4 SV=1	6.08E-168	76.435	54.17348609	sp|B4KID9|PESC_DROMO	Pescadillo homolog OS=Drosophila mojavensis OX=7230 GN=GI18209 PE=3 SV=1	0	52.674	100
P_KWMTBOMO05631	tr|H9JAK7|H9JAK7_BOMMO	Protein kinase domain-containing protein OS=Bombyx mori OX=7091 PE=4 SV=1	0	94.696	90.02808989	sp|Q1ECX4|TLK2_DANRE	Serine/threonine-protein kinase tousled-like 2 OS=Danio rerio OX=7955 GN=tlk2 PE=2 SV=2	0	52.639	100
P_KWMTBOMO05675	tr|Q5UAR5|Q5UAR5_BOMMO	Ribosomal protein L23A OS=Bombyx mori OX=7091 GN=RpL23A PE=2 SV=1	0	100	100	sp|P62752|RL23A_RAT	60S ribosomal protein L23a OS=Rattus norvegicus OX=10116 GN=Rpl23a PE=2 SV=1	8.65E-62	76.19	35.79545455
P_KWMTBOMO05680	tr|H9JBF1|H9JBF1_BOMMO	VWFC domain-containing protein OS=Bombyx mori OX=7091 PE=4 SV=1	0	97.473	98.92405063	--	--	--	--	--
P_KWMTBOMO05687	tr|A8R081|A8R081_BOMMO	Bombyx homolog of P-element somatic inhibitor OS=Bombyx mori OX=7091 GN=BmPSI PE=2 SV=1	1.37E-12	23.754	27.52219532	sp|Q00341|VIGLN_HUMAN	Vigilin OS=Homo sapiens OX=9606 GN=HDLBP PE=1 SV=2	0	49.197	100

P_KWMTBO MO05700	tr|H9JBG4|H9JBG4_B OMMO	Complex I-ESSS OS=Bombyx mori OX=7091 GN=101739775 PE=3 SV=1	8.39E-108	100	100	sp|O09111|NDUBB _MOUSE	NADH dehydrogenase [ubiquinone] 1 beta subcomplex subunit 11, mitochondrial OS=Mus musculus OX=10090 GN=Ndufb11 PE=1 SV=2	1.76E-13	30.216	97.2027972
P_KWMTBO MO05723	tr|Q5UAM7|Q5UAM7_ BOMMO	Ribosomal protein S15A OS=Bombyx mori OX=7091 GN=RpS15A PE=2 SV=1	5.26E-94	100	100	sp|Q6XIM8|RS15A_ DROYA	40S ribosomal protein S15a OS=Drosophila yakuba OX=7245 GN=RpS15Aa PE=2 SV=3	3.84E-87	93.077	100
P_KWMTBO MO05979	tr|Q5UAT0|Q5UAT0_B OMMO	Ribosomal protein L10 OS=Bombyx mori OX=7091 GN=RpL10 PE=2 SV=1	7.89E-166	100	100	sp|O96647|RL10_B OMMA	60S ribosomal protein L10 OS=Bombyx mandarina OX=7092 GN=RpL10 PE=2 SV=1	2.91E-164	99.543	100
P_KWMTBO MO06076	tr|H9IWS8|H9IWS8_B OMMO	Thioredoxin domain-containing protein OS=Bombyx mori OX=7091 GN=101738690 PE=4 SV=1	8.04E-161	100	100	sp|Q9CQ79|TXND9 _MOUSE	Thioredoxin domain-containing protein 9 OS=Mus musculus OX=10090 GN=Txndc9 PE=1 SV=1	8.67E-76	52.709	94.41860465
P_KWMTBO MO06334	tr|D2WL76|D2WL76_B OMMO	Sericin 2 OS=Bombyx mori OX=7091 GN=Ser2 PE=4 SV=1	0	96.132	100	sp|Q25460|FP1_MY TED	Adhesive plaque matrix protein (Fragment) OS=Mytilus edulis OX=6550 GN=FP1 PE=1 SV=1	1.11E-56	36.364	41.02122777
P_KWMTBO MO06373	tr|Q2F5L8|Q2F5L8_BO MMO	Ribonucloprotein OS=Bombyx mori OX=7091 PE=2 SV=1	8.53E-93	100	100	sp|Q5XH16|NH2L1 _XENLA	NHP2-like protein 1 OS=Xenopus laevis OX=8355 GN=snu13 PE=2 SV=1	4.36E-77	81.89	97.69230769
P_KWMTBO MO06621	tr|U3U9V0|U3U9V0_B OMMO	Uncharacterized protein OS=Bombyx mori OX=7091 PE=2 SV=1	0	100	100	sp|Q61686|CBX5_M OUSE	Chromobox protein homolog 5 OS=Mus musculus OX=10090 GN=Cbx5 PE=1 SV=1	1.39E-17	35.882	61.5942029
P_KWMTBO MO06632	tr|H9JQ89|H9JQ89_BO MMO	Nucleoprotein TPR OS=Bombyx mori OX=7091 PE=3 SV=1	0	87.867	58.41986456	sp|A1Z8P9|TPR_DR OME	Nucleoprotein TPR OS=Drosophila melanogaster OX=7227 GN=Mtor PE=1 SV=1	0	31.858	100
P_KWMTBO MO06731	tr|H9JQE1|H9JQE1_B OMMO	Histone deacetylase OS=Bombyx mori OX=7091 PE=3 SV=1	0	91.686	89.64803313	sp|Q94517|HDAC1_ DROME	Histone deacetylase HDAC1 OS=Drosophila melanogaster OX=7227 GN=HDAC1 PE=1 SV=2	0	92.634	92.75362319
P_KWMTBO MO06760	tr|H9JRG6|H9JRG6_B OMMO	Eukaryotic translation initiation factor 3 subunit L OS=Bombyx mori OX=7091 PE=3 SV=1	0	100	98.33641405	sp|Q16FL6|EIF3L_ AEDAE	Eukaryotic translation initiation factor 3 subunit L OS=Aedes aegypti OX=7159 GN=AAEL009617 PE=3 SV=1	0	70.534	100
P_KWMTBO MO06803	tr|H9JR86|H9JR86_BO MMO	CP-type G domain-containing protein OS=Bombyx mori OX=7091 GN=101742911 PE=4 SV=1	0	100	100	sp|Q9W590|LSG1_ DROME	Large subunit GTPase 1 homolog OS=Drosophila melanogaster OX=7227 GN=Ns3 PE=1 SV=1	5.32E-148	39.605	100
P_KWMTBO MO07076	tr|H9JLN4|H9JLN4_B OMMO	Uncharacterized protein OS=Bombyx mori OX=7091 PE=4 SV=1	0	99.76	80.03838772	sp|Q28EW0|TM87A _XENTR	Transmembrane protein 87A OS=Xenopus tropicalis OX=8364 GN=tmem87a PE=2 SV=1	5.77E-124	39.051	100
P_KWMTBO MO07274	tr|A0A4P9D450|A0A4P 9D450_BOMMO	Piwi OS=Bombyx mori OX=7091 PE=2 SV=1	0	100	100	sp|A8D8P8|SIWI_B OMMO	Piwi-like protein Siwi OS=Bombyx mori OX=7091 GN=Siwi PE=1 SV=1	0	100	100
P_KWMTBO MO07373	tr|H9J6N5|H9J6N5_BO MMO	Uncharacterized protein OS=Bombyx mori OX=7091 PE=4 SV=1	4.18E-97	100	100	sp|Q9U1N0|HRP65_ CHITE	Hrp65 protein OS=Chironomus tentans OX=7153 GN=HRP65 PE=1 SV=1	6.32E-24	60.227	61.97183099
P_KWMTBO MO07600	tr|Q1HPU6|Q1HPU6_B OMMO	Sec61p gamma subunit OS=Bombyx mori OX=7091 GN=733141 PE=2 SV=1	5.06E-45	100	100	sp|Q7Z1B8|S61G1_ GRYOR	Protein transport protein Sec61 subunit gamma OS=Gryllotalpa orientalis OX=213494 GN=SEC61G PE=3 SV=1	3.67E-42	91.176	100
P_KWMTBO MO07679	tr|H9IVK1|H9IVK1_B OMMO	Uncharacterized protein OS=Bombyx mori OX=7091 PE=4 SV=1	0	99.634	39.73799127	sp|Q8TEQ6|GEMI5 _HUMAN	Gem-associated protein 5 OS=Homo sapiens OX=9606 GN=GEMIN5 PE=1 SV=3	3.95E-26	26.185	58.36972344
P_KWMTBO MO07688	tr|Q5UAP3|Q5UAP3_B OMMO	40S ribosomal protein S2 OS=Bombyx mori OX=7091 GN=RpS2 PE=2 SV=1	0	100	100	sp|P31009|RS2_DR OME	40S ribosomal protein S2 OS=Drosophila melanogaster OX=7227 GN=RpS2 PE=1 SV=2	4.18E-144	90.278	82.75862069
P_KWMTBO MO07780	tr|H9IUH3|H9IUH3_B OMMO	Eukaryotic translation initiation factor 3 subunit M OS=Bombyx mori OX=7091 GN=101737255 PE=3 SV=1	0	100	100	sp|Q17D30|EIF3M_ AEDAE	Eukaryotic translation initiation factor 3 subunit M OS=Aedes aegypti OX=7159 GN=AAEL004347 PE=3 SV=1	0	72.798	100
P_KWMTBO MO07811	tr|H9IVE3|H9IVE3_BO MMO	RNA helicase OS=Bombyx mori OX=7091 GN=101746250 PE=4 SV=1	0	100	100	sp|O61305|DDX19_ DROME	DEAD-box helicase Dbp80 OS=Drosophila melanogaster OX=7227 GN=Dbp80 PE=1 SV=1	0	70.652	98.92473118
P_KWMTBO MO07930	tr|Q1HQ48|Q1HQ48_B OMMO	Mitochondrial single-stranded DNA-binding protein OS=Bombyx mori OX=7091 PE=2 SV=1	1.39E-108	99.324	100	sp|P54622|SSBP_D ROME	Single-stranded DNA-binding protein, mitochondrial OS=Drosophila melanogaster OX=7227 GN=mtSSB PE=1 SV=2	2.56E-50	64.545	74.32432432
P_KWMTBO MO07952	tr|H9IUR1|H9IUR1_B OMMO	Histidine--tRNA ligase OS=Bombyx mori OX=7091 PE=3 SV=1	0	66.062	100	sp|Q61035|HARS1_ MOUSE	Histidine--tRNA ligase, cytoplasmic OS=Mus musculus OX=10090 GN=Hars1 PE=1 SV=2	0	67.683	95.53398058
P_KWMTBO MO08022	tr|H9IV52|H9IV52_BO MMO	Dsp protein OS=Bombyx mori OX=7091 GN=dsp PE=2 SV=1	0	100	71.39423077	sp|Q24537|HMG2_ DROME	High mobility group protein DSP1 OS=Drosophila melanogaster OX=7227 GN=Dsp1 PE=2 SV=1	1.07E-75	48.182	79.32692308
P_KWMTBO MO08044	tr|H9IV44|H9IV44_BO MMO	DNA-(apurinic or apyrimidinic site) endonuclease OS=Bombyx mori OX=7091 GN=101739740 PE=3 SV=1	0	100	100	sp|P27864|RRP1_D ROME	Recombination repair protein 1 OS=Drosophila melanogaster OX=7227 GN=Rrp1 PE=1 SV=2	6.85E-131	44.487	83.2278481
P_KWMTBO MO08100	tr|Q5UAR8|Q5UAR8_ BOMMO	60S ribosomal protein L21 OS=Bombyx mori OX=7091 GN=RpL21 PE=2 SV=1	1.42E-115	100	100	sp|P46778|RL21_H UMAN	60S ribosomal protein L21 OS=Homo sapiens OX=9606 GN=RPL21 PE=1 SV=2	2.69E-77	66.875	100
P_KWMTBO MO08194	tr|H9JIX2|H9JIX2_BO MMO	Uncharacterized protein OS=Bombyx mori OX=7091 PE=3 SV=1	1.35E-72	66.522	52.15419501	sp|Q9VQ79|AIFM1_ DROME	Putative apoptosis-inducing factor 1, mitochondrial OS=Drosophila melanogaster OX=7227 GN=AIF PE=2 SV=2	5.12E-60	35.423	72.33560091
P_KWMTBO MO08201	tr|H9JIW6|H9JIW6_BO MMO	Glucosamine-6-phosphate isomerase OS=Bombyx mori OX=7091 GN=101746460 PE=3 SV=1	0	98.12	98.51851852	sp|A4IHW6|GNPI2_ XENTR	Glucosamine-6-phosphate isomerase 2 OS=Xenopus tropicalis OX=8364 GN=gnpda2 PE=2 SV=1	1.21E-159	75.281	98.88888889
P_KWMTBO MO08213	tr|H9JIW2|H9JIW2_BO MMO	Uncharacterized protein OS=Bombyx mori OX=7091 GN=101746972 PE=4 SV=1	0	100	100	sp|P51610|HCFC1_ HUMAN	Host cell factor 1 OS=Homo sapiens OX=9606 GN=HCFC1 PE=1 SV=2	0	71.99	45.58472554
P_KWMTBO MO08535	tr|B3GQU6|B3GQU6_ BOMMO	3-hydroxyisobutyrate dehydrogenase OS=Bombyx mori OX=7091 GN=Hibadh PE=2 SV=1	5.84E-10	25.126	78.34645669	sp|Q7Q161|GLYR1_ ANOGA	Putative oxidoreductase GLYR1 homolog OS=Anopheles gambiae OX=7165 GN=AGAP009949 PE=3 SV=5	2.16E-119	64.427	99.60629921
P_KWMTBO MO08542	tr|H9JIM5|H9JIM5_BO MMO	Uncharacterized protein OS=Bombyx mori OX=7091 PE=3 SV=1	1.95E-83	99.167	46.692607	sp|Q9V447|KRH2_ DROME	Krueppel homolog 2 OS=Drosophila melanogaster OX=7227 GN=Kr-h2 PE=1 SV=1	4.91E-83	50.628	92.99610895

P_KWMTBOMO08642	tr|F8UN44|F8UN44_BOMMO	Heat shock protein 70-3 OS=Bombyx mori OX=7091 PE=2 SV=1	0	100	100	sp|P29844|BIP_DROME	Endoplasmic reticulum chaperone BiP OS=Drosophila melanogaster OX=7227 GN=Hsc70-3 PE=1 SV=2	0	89.708	99.38931298
P_KWMTBOMO08652	tr|H9JDP0|H9JDP0_BOMMO	Diadenosine tetraphosphate synthetase OS=Bombyx mori OX=7091 PE=3 SV=1	0	100	100	sp|Q04451|SYG_BOMMO	Glycine--tRNA ligase OS=Bombyx mori OX=7091 PE=1 SV=2	0	99.118	100
P_KWMTBOMO08740	tr|H9JEF8|H9JEF8_BOMMO	UBR-type domain-containing protein OS=Bombyx mori OX=7091 PE=4 SV=1	0	99.168	55.82430341	sp|Q9VLT5|POE_DROME	Protein purity of essence OS=Drosophila melanogaster OX=7227 GN=poe PE=1 SV=1	0	40.577	100
P_KWMTBOMO08776	sp|Q1HPW4|EIF3I_BOMMO	Eukaryotic translation initiation factor 3 subunit I OS=Bombyx mori OX=7091 PE=2 SV=1	0	100	100	sp|Q1HPW4|EIF3I_BOMMO	Eukaryotic translation initiation factor 3 subunit I OS=Bombyx mori OX=7091 PE=2 SV=1	0	100	100
P_KWMTBOMO08930	tr|H9JDX6|H9JDX6_BOMMO	S-formylglutathione hydrolase OS=Bombyx mori OX=7091 PE=3 SV=1	0	100	89.54703833	sp|Q9GJT2|ESTD_PIG	S-formylglutathione hydrolase OS=Sus scrofa OX=9823 GN=ESD PE=2 SV=1	1.95E-129	61.754	99.30313589
P_KWMTBOMO08983	tr|Q5UAT2|Q5UAT2_BOMMO	60S ribosomal protein L8 OS=Bombyx mori OX=7091 GN=RpL8 PE=2 SV=1	0	100	100	sp|Q95V39|RL8_SPOFR	60S ribosomal protein L8 OS=Spodoptera frugiperda OX=7108 GN=RpL8 PE=2 SV=1	0	100	100
P_KWMTBOMO09000	tr|H9JE32|H9JE32_BOMMO	Uncharacterized protein OS=Bombyx mori OX=7091 PE=3 SV=1	2.27E-105	100	100	sp|P79382|MGST1_PIG	Microsomal glutathione S-transferase 1 OS=Sus scrofa OX=9823 GN=MGST1 PE=2 SV=3	3.61E-32	43.333	99.33774834
P_KWMTBOMO09034	tr|H9JDJ7|H9JDJ7_BOMMO	RNA helicase OS=Bombyx mori OX=7091 PE=3 SV=1	0	96.038	74.22969188	sp|P16381|DDX3L_MOUSE	Putative ATP-dependent RNA helicase Pl10 OS=Mus musculus OX=10090 GN=D1Pas1 PE=1 SV=1	0	56.444	94.53781513
P_KWMTBOMO09050	tr|H9JDA0|H9JDA0_BOMMO	Uncharacterized protein OS=Bombyx mori OX=7091 PE=4 SV=1	0	100	92.79661017	sp|Q96RQ3|MCCA_HUMAN	Methylcrotonoyl-CoA carboxylase subunit alpha, mitochondrial OS=Homo sapiens OX=9606 GN=MCCC1 PE=1 SV=3	0	56.278	95.62146893
P_KWMTBOMO09054	tr|Q1HQ47|Q1HQ47_BOMMO	Isocitrate dehydrogenase [NADP] OS=Bombyx mori OX=7091 GN=100101164 PE=2 SV=1	0	100	100	sp|P33198|IDHP_PIG	Isocitrate dehydrogenase [NADP], mitochondrial (Fragment) OS=Sus scrofa OX=9823 GN=IDH2 PE=1 SV=1	0	71.325	95.40229885
P_KWMTBOMO09300	tr|Q5UAU1|Q5UAU1_BOMMO	60S acidic ribosomal protein P0 OS=Bombyx mori OX=7091 GN=RpP0 PE=1 SV=1	0	100	100	sp|Q9U3U0|RLA0_CERCA	60S acidic ribosomal protein P0 OS=Ceratitis capitata OX=7213 GN=RpLP0 PE=3 SV=1	1.73E-177	81.388	100
P_KWMTBOMO09385	tr|H9JT97|H9JT97_BOMMO	TLDc domain-containing protein OS=Bombyx mori OX=7091 PE=4 SV=1	6.24E-121	98.235	34.97942387	--	--	--	--	--
P_KWMTBOMO09390	tr|H9JU84|H9JU84_BOMMO	Splicing factor, arginine/serine-rich 1 OS=Bombyx mori OX=7091 PE=3 SV=1	6.24E-155	100	100	sp|Q6NYA0|SRS1B_DANRE	Serine/arginine-rich splicing factor 1B OS=Danio rerio OX=7955 GN=srsf1b PE=2 SV=1	3.58E-87	71.939	91.1627907
P_KWMTBOMO09433	tr|H9JU67|H9JU67_BOMMO	Uncharacterized protein OS=Bombyx mori OX=7091 PE=3 SV=1	0	89.433	100	sp|A8JUV0|SBNO_DROME	Protein strawberry notch OS=Drosophila melanogaster OX=7227 GN=sno PE=1 SV=2	0	65.705	92.03539823
P_KWMTBOMO09591	tr|H9JTH6|H9JTH6_BOMMO	Uncharacterized protein OS=Bombyx mori OX=7091 PE=3 SV=1	9.34E-169	100	55.27522936	sp|O61492|FLOT2_DROME	Flotillin-2 OS=Drosophila melanogaster OX=7227 GN=Flo2 PE=2 SV=3	0	76.923	95.41284404
P_KWMTBOMO09788	tr|Q2QEH2|Q2QEH2_BOMMO	Cellular retinoic acid binding protein OS=Bombyx mori OX=7091 GN=BmFABP1 PE=2 SV=1	1.36E-94	100	100	sp|C4N147|FABP1_DORPE	Sodium/calcium exchanger regulatory protein 1 OS=Doryteuthis pealeii OX=1051067 PE=1 SV=1	2.61E-37	46.4	94.6969697
P_KWMTBOMO09954	tr|H9JUM2|H9JUM2_BOMMO	Proline dehydrogenase OS=Bombyx mori OX=7091 PE=3 SV=1	0	100	58.63636364	sp|Q04499|PROD_DROME	Proline dehydrogenase 1, mitochondrial OS=Drosophila melanogaster OX=7227 GN=slgA PE=2 SV=2	0	60.081	74.39393939
P_KWMTBOMO09980	tr|Q1HPT4|Q1HPT4_BOMMO	14-3-3 epsilon OS=Bombyx mori OX=7091 PE=2 SV=1	0	100	100	sp|P92177|1433E_DROME	14-3-3 protein epsilon OS=Drosophila melanogaster OX=7227 GN=14-3-3epsilon PE=1 SV=2	4.71E-163	84.906	100
P_KWMTBOMO10056	tr|B5M9A0|B5M9A0_BOMMO	Myosin heavy chain (Fragment) OS=Bombyx mori OX=7091 PE=2 SV=1	0	55.179	26.95431472	sp|Q99323|MYSN_DROME	Myosin heavy chain, non-muscle OS=Drosophila melanogaster OX=7227 GN=zip PE=1 SV=2	0	74.987	100
P_KWMTBOMO10128	tr|H9J806|H9J806_BOMMO	Uncharacterized protein OS=Bombyx mori OX=7091 PE=3 SV=1	1.66E-70	100	91.58878505	sp|Q9W141|ATPK_DROME	Putative ATP synthase subunit f, mitochondrial OS=Drosophila melanogaster OX=7227 GN=ATPsynF PE=3 SV=1	7.40E-60	75.701	100
P_KWMTBOMO10169	tr|H9J7Y6|H9J7Y6_BOMMO	OMPdecase OS=Bombyx mori OX=7091 PE=3 SV=1	0	100	100	sp|Q01637|UMPS_DROME	Uridine 5'-monophosphate synthase OS=Drosophila melanogaster OX=7227 GN=r-l PE=2 SV=2	6.51E-172	50.924	100
P_KWMTBOMO10299	tr|H9JCL8|H9JCL8_BOMMO	Uncharacterized protein OS=Bombyx mori OX=7091 PE=4 SV=1	0	96.75	100	sp|Q9VLK2|Y3096_DROME	Ribosomal L1 domain-containing protein CG13096 OS=Drosophila melanogaster OX=7227 GN=CG13096 PE=1 SV=1	1.41E-35	33.197	62.88659794
P_KWMTBOMO10376	tr|H9JC16|H9JC16_BOMMO	Pept_C1 domain-containing protein OS=Bombyx mori OX=7091 PE=3 SV=1	0	100	100	sp|P00787|CATB_RAT	Cathepsin B OS=Rattus norvegicus OX=10116 GN=Ctsb PE=1 SV=2	8.05E-140	58.333	99.70326409
P_KWMTBOMO10509	tr|Q5UAR7|Q5UAR7_BOMMO	Ribosomal protein L22 OS=Bombyx mori OX=7091 GN=RpL22 PE=2 SV=1	1.07E-106	100	100	sp|P52819|RL22_CAEEL	60S ribosomal protein L22 OS=Caenorhabditis elegans OX=6239 GN=rpl-22 PE=1 SV=3	4.50E-38	49.254	91.15646259
P_KWMTBOMO11083	tr|Q9BPS3|Q9BPS3_BOMMO	Elongation factor 1 gamma OS=Bombyx mori OX=7091 GN=ef-1g PE=2 SV=1	0	100	100	sp|P12261|EF1G_ARTSA	Elongation factor 1-gamma OS=Artemia salina OX=85549 PE=1 SV=3	0	65.741	100
P_KWMTBOMO11192	tr|C6L8Q2|C6L8Q2_BOMMO	Putative acetyl transferase OS=Bombyx mori OX=7091 GN=Bmat1 PE=2 SV=1	0	100	100	sp|P42765|THIM_HUMAN	3-ketoacyl-CoA thiolase, mitochondrial OS=Homo sapiens OX=9606 GN=ACAA2 PE=1 SV=2	5.87E-173	58.987	99.74747475
P_KWMTBOMO11558	tr|Q2F5S1|Q2F5S1_BOMMO	Microtubule-associated protein RP/EB family member 3 OS=Bombyx mori OX=7091 PE=2 SV=1	0	100	100	sp|Q3ZBD9|MARE1_BOVIN	Microtubule-associated protein RP/EB family member 1 OS=Bos taurus OX=9913 GN=MAPRE1 PE=2 SV=3	2.38E-98	58.333	94.48818898
P_KWMTBOMO11678	tr|Q5UAS7|Q5UAS7_BOMMO	60S ribosomal protein L12 OS=Bombyx mori OX=7091 GN=RpL12 PE=2 SV=1	1.46E-118	100	100	sp|P23358|RL12_RAT	60S ribosomal protein L12 OS=Rattus norvegicus OX=10116 GN=Rpl12 PE=2 SV=1	3.08E-96	81.25	97.56097561
P_KWMTBOMO11689	tr|H9J385|H9J385_BOMMO	Uncharacterized protein OS=Bombyx mori OX=7091 PE=4 SV=1	0	100	100	sp|O18373|SPS1_DROME	Inactive selenide, water dikinase-like protein OS=Drosophila melanogaster OX=7227 GN=SelD PE=2 SV=1	0	87.94	99.00497512
P_KWMTBOMO11731	sp|Q1HPK6|EF2_BOMMO	Translation elongation factor 2 OS=Bombyx mori OX=7091 GN=tef2 PE=1 SV=1	0	100	100	sp|Q1HPK6|EF2_BOMMO	Translation elongation factor 2 OS=Bombyx mori OX=7091 GN=tef2 PE=1 SV=1	0	100	100

P_KWMTBOMO11985	tr|Q5UAT4|Q5UAT4_BOMMO	Ribosomal protein L7 OS=Bombyx mori OX=7091 GN=RpL7 PE=2 SV=1	0	100	100	sp|P32100|RL7_DROME	60S ribosomal protein L7 OS=Drosophila melanogaster OX=7227 GN=RpL7 PE=1 SV=2	4.63E-117	66.667	92.56505576
P_KWMTBOMO12037	tr|H9J470|H9J470_BOMMO	DEK_C domain-containing protein OS=Bombyx mori OX=7091 PE=4 SV=1	0	91.221	94.15322581	sp|Q6AXS3|DEK_RAT	Protein DEK OS=Rattus norvegicus OX=10116 GN=Dek PE=1 SV=1	5.05E-20	47.368	19.15322581
P_KWMTBOMO12057	tr|H9J463|H9J463_BOMMO	Eukaryotic translation initiation factor 2 subunit 1 OS=Bombyx mori OX=7091 GN=693063 PE=3 SV=1	0	100	100	sp|P41374|IF2A_DROME	Eukaryotic translation initiation factor 2 subunit 1 OS=Drosophila melanogaster OX=7227 GN=eIF2alpha PE=2 SV=1	2.35E-165	74.832	89.75903614
P_KWMTBOMO12165	tr|H9JWZ8|H9JWZ8_BOMMO	Glutathione synthase OS=Bombyx mori OX=7091 PE=3 SV=1	1.95E-169	99.565	52.99539171	sp|P35668|GSHB_XENLA	Glutathione synthetase OS=Xenopus laevis OX=8355 GN=gss PE=2 SV=1	2.49E-108	43.564	93.0875576
P_KWMTBOMO12208	tr|H9JE23|H9JE23_BOMMO	SAC domain-containing protein OS=Bombyx mori OX=7091 PE=4 SV=1	3.33E-156	100	35.95317726	sp|Q9W0I6|SAC1_DROME	Phosphatidylinositol-3-phosphatase SAC1 OS=Drosophila melanogaster OX=7227 GN=Sac1 PE=2 SV=1	0	49.144	97.65886288
P_KWMTBOMO12287	tr|Q5UAM2|Q5UAM2_BOMMO	40S ribosomal protein S20 OS=Bombyx mori OX=7091 GN=RpS20 PE=2 SV=1	3.74E-87	100	100	sp|P55828|RS20_DROME	40S ribosomal protein S20 OS=Drosophila melanogaster OX=7227 GN=RpS20 PE=1 SV=1	1.92E-68	83.74	100
P_KWMTBOMO12350	tr|H9JS78|H9JS78_BOMMO	DZF domain-containing protein OS=Bombyx mori OX=7091 PE=4 SV=1	0	95.423	86.70634921	sp|Q5REX3|ZFR_PONAB	Zinc finger RNA-binding protein OS=Pongo abelii OX=9601 GN=ZFR PE=2 SV=1	0	38.139	100
P_KWMTBOMO12400	tr|H9JSB8|H9JSB8_BOMMO	RRM domain-containing protein OS=Bombyx mori OX=7091 PE=4 SV=1	7.88E-54	100	70	--	--	--	--	--
P_KWMTBOMO12483	tr|Q66SV9|Q66SV9_BOMMO	40S ribosomal protein S15 OS=Bombyx mori OX=7091 GN=S15 PE=2 SV=1	9.24E-107	100	100	sp|P62844|RS15_PIG	40S ribosomal protein S15 OS=Sus scrofa OX=9823 GN=RPS15 PE=1 SV=2	2.23E-77	81.633	100
P_KWMTBOMO12659	tr|H9IVX5|H9IVX5_BOMMO	T-complex protein 1 subunit gamma OS=Bombyx mori OX=7091 GN=101744569 PE=3 SV=1	0	100	100	sp|P48605|TCPG_DROME	T-complex protein 1 subunit gamma OS=Drosophila melanogaster OX=7227 GN=CCT3 PE=2 SV=2	0	81.041	99.62962963
P_KWMTBOMO12895	tr|Q19AA9|Q19AA9_BOMMO	Transport protein Sec61 alpha subunit OS=Bombyx mori OX=7091 GN=733068 PE=2 SV=1	0	100	100	sp|Q9JLR1|S61A2_MOUSE	Protein transport protein Sec61 subunit alpha isoform 2 OS=Mus musculus OX=10090 GN=Sec61a2 PE=2 SV=3	0	91.597	100
P_KWMTBOMO12902	tr|H9IT95|H9IT95_BOMMO	Calreticulin OS=Bombyx mori OX=7091 PE=3 SV=1	0	100	100	sp|Q7Z1E6|CALR_BOMMO	Calreticulin OS=Bombyx mori OX=7091 GN=crt PE=1 SV=1	0	99.749	100
P_KWMTBOMO12903	tr|H9IT94|H9IT94_BOMMO	CP-type G domain-containing protein OS=Bombyx mori OX=7091 GN=101736688 PE=4 SV=1	0	100	100	sp|Q8MT06|GNL3_DROME	Guanine nucleotide-binding protein-like 3 homolog OS=Drosophila melanogaster OX=7227 GN=Ns1 PE=1 SV=2	4.41E-100	48.179	100
P_KWMTBOMO12968	tr|H9IT65|H9IT65_BOMMO	Uncharacterized protein OS=Bombyx mori OX=7091 PE=4 SV=1	0	89.312	35.04361211	sp|A2BGM5|FOXN4_DANRE	Forkhead box protein N4 OS=Danio rerio OX=7955 GN=foxn4 PE=1 SV=1	4.74E-53	62	7.696254489
P_KWMTBOMO13230	tr|H9JKC7|H9JKC7_BOMMO	LisH domain-containing protein OS=Bombyx mori OX=7091 PE=4 SV=2	4.71E-171	74.845	98.02130898	sp|A4II09|EIF3A_XENTR	Eukaryotic translation initiation factor 3 subunit A OS=Xenopus tropicalis OX=8364 GN=eif3a PE=2 SV=1	1.65E-06	44.242	25.11415525
P_KWMTBOMO13287	tr|H9J5C1|H9J5C1_BOMMO	RuvB-like helicase OS=Bombyx mori OX=7091 GN=101739823 PE=3 SV=1	0	100	100	sp|Q0IFL2|RUVB1_AEDAE	RuvB-like helicase 1 OS=Aedes aegypti OX=7159 GN=pont PE=3 SV=1	0	83.736	99.78070175
P_KWMTBOMO13455	sp|Q5UAP0|RS4_BOMMO	40S ribosomal protein S4 OS=Bombyx mori OX=7091 GN=RpS4 PE=2 SV=1	0	100	100	sp|Q5UAP0|RS4_BOMMO	40S ribosomal protein S4 OS=Bombyx mori OX=7091 GN=RpS4 PE=2 SV=1	0	100	100
P_KWMTBOMO13567	tr|H9JN68|H9JN68_BOMMO	Uncharacterized protein OS=Bombyx mori OX=7091 PE=4 SV=1	0	100	61.18326118	--	--	--	--	--
P_KWMTBOMO13569	tr|Q5UAR3|Q5UAR3_BOMMO	Ribosomal protein L26 OS=Bombyx mori OX=7091 GN=RpL26 PE=2 SV=1	7.52E-72	100	100	sp|Q95WA0|RL26_LITLI	60S ribosomal protein L26 OS=Littorina littorea OX=31216 GN=RPL26 PE=2 SV=1	1.90E-51	80.392	100
P_KWMTBOMO13641	tr|H9JND7|H9JND7_BOMMO	SMC_N domain-containing protein OS=Bombyx mori OX=7091 PE=4 SV=1	4.13E-107	100	69.04761905	sp|O93309|SMC3_XENLA	Structural maintenance of chromosomes protein 3 OS=Xenopus laevis OX=8355 GN=smc3 PE=1 SV=2	5.93E-87	68.317	96.19047619
P_KWMTBOMO13800	tr|H9JPL2|H9JPL2_BOMMO	60S ribosomal protein L18a OS=Bombyx mori OX=7091 PE=3 SV=1	4.32E-132	100	100	sp|Q8WQI7|RL18A_SPOFR	60S ribosomal protein L18a OS=Spodoptera frugiperda OX=7108 GN=RpL18A PE=2 SV=1	1.32E-126	95.48	100
P_KWMTBOMO13979	tr|Q2F646|Q2F646_BOMMO	Hairy cell leukemia protein 1 OS=Bombyx mori OX=7091 GN=693008 PE=2 SV=1	0	99.614	62.40963855	sp|O95478|NSA2_HUMAN	Ribosome biogenesis protein NSA2 homolog OS=Homo sapiens OX=9606 GN=NSA2 PE=1 SV=1	9.59E-148	76.923	62.65060241
P_KWMTBOMO13997	tr|Q6T9Z7|Q6T9Z7_BOMMO	Fibroinase OS=Bombyx mori OX=7091 GN=693031 PE=2 SV=1	0	100	100	sp|Q26636|CATL_SARPE	Cathepsin L OS=Sarcophaga peregrina OX=7386 PE=1 SV=1	6.18E-177	68.902	96.18768328
P_KWMTBOMO14024	tr|H9JPA3|H9JPA3_BOMMO	DJ-1 beta OS=Bombyx mori OX=7091 GN=100422789 PE=2 SV=1	2.25E-138	100	100	sp|Q9VA37|DJ1B_DROME	Protein dj-1beta OS=Drosophila melanogaster OX=7227 GN=dj-1beta PE=1 SV=3	5.03E-67	54.787	98.94736842
P_KWMTBOMO14420	tr|H9IS77|H9IS77_BOMMO	Uncharacterized protein OS=Bombyx mori OX=7091 GN=101744691 PE=4 SV=1	0	99.786	99.78609626	sp|Q9BUJ2|HNRL1_HUMAN	Heterogeneous nuclear ribonucleoprotein U-like protein 1 OS=Homo sapiens OX=9606 GN=HNRNPUL1 PE=1 SV=2	7.54E-52	41.87	26.31016043
P_KWMTBOMO14483	tr|H9IS46|H9IS46_BOMMO	Uncharacterized protein OS=Bombyx mori OX=7091 PE=4 SV=1	0	99.603	91.30434783	sp|P25171|RCC1_DROME	Regulator of chromosome condensation OS=Drosophila melanogaster OX=7227 GN=Rcc1 PE=1 SV=2	7.43E-112	40.272	93.11594203
P_KWMTBOMO14536	tr|H9JXM7|H9JXM7_BOMMO	Citrate synthase OS=Bombyx mori OX=7091 GN=101740166 PE=3 SV=1	0	99.425	32.01471941	sp|Q91V92|ACLY_MOUSE	ATP-citrate synthase OS=Mus musculus OX=10090 GN=Acly PE=1 SV=1	0	61.187	100
P_KWMTBOMO14633	tr|H9JJ62|H9JJ62_BOMMO	Uncharacterized protein OS=Bombyx mori OX=7091 PE=3 SV=1	0	100	94.42508711	sp|Q9H8M5|CNNM2_HUMAN	Metal transporter CNNM2 OS=Homo sapiens OX=9606 GN=CNNM2 PE=1 SV=2	2.48E-179	49.733	97.73519164
P_KWMTBOMO14752	tr|H9J2U6|H9J2U6_BOMMO	PUA domain-containing protein OS=Bombyx mori OX=7091 GN=101744752 PE=3 SV=1	0	100	100	sp|O44081|DKC1_DROME	H/ACA ribonucleoprotein complex subunit 4 OS=Drosophila melanogaster OX=7227 GN=Nop60B PE=1 SV=1	0	76.69	91.47121535
P_KWMTBOMO14804	tr|H9JRQ1|H9JRQ1_BOMMO	Programmed cell death protein 4 OS=Bombyx mori OX=7091 GN=101743674 PE=3 SV=1	0	100	59.14221219	sp|Q98TX3|PDCD4_CHICK	Programmed cell death protein 4 OS=Gallus gallus OX=9031 GN=PDCD4 PE=2 SV=1	3.96E-92	43.371	100

P_KWMTBOMO14858		tr|H9JRJ9|H9JRJ9_BOMMO	Uncharacterized protein OS=Bombyx mori OX=7091 PE=4 SV=2	0	89.299	80.63814358		sp|Q02645|HTS_DROME	Protein hu-li tai shao OS=Drosophila melanogaster OX=7227 GN=hts PE=1 SV=2	0	56.932	49.16606236
P_KWMTBOMO14877		tr|Q0ZB80|Q0ZB80_BOMMO	eIF2B-beta protein OS=Bombyx mori OX=7091 PE=2 SV=1	0	99.717	100		sp|Q5E9B4|EI2BB_BOVIN	Translation initiation factor eIF-2B subunit beta OS=Bos taurus OX=9913 GN=EIF2B2 PE=2 SV=1	1.50E-63	36.676	98.86685552
P_KWMTBOMO15159		tr|H9J5Z1|H9J5Z1_BOMMO	Uncharacterized protein OS=Bombyx mori OX=7091 PE=4 SV=1	0	97.685	99.34282585		sp|Q99PL5|RRBP1_MOUSE	Ribosome-binding protein 1 OS=Mus musculus OX=10090 GN=Rrbp1 PE=1 SV=2	3.36E-17	27.524	67.25082147
P_KWMTBOMO15176		tr|H9J5Z8|H9J5Z8_BOMMO	Uncharacterized protein OS=Bombyx mori OX=7091 GN=101745895 PE=4 SV=1	0	100	100		sp|Q86UA1|PRP39_HUMAN	Pre-mRNA-processing factor 39 OS=Homo sapiens OX=9606 GN=PRPF39 PE=1 SV=3	3.41E-142	39.835	65.26429342
P_KWMTBOMO15419		tr|H9J5I6|H9J5I6_BOMMO	Methyltransf_25 domain-containing protein OS=Bombyx mori OX=7091 PE=4 SV=1	0	100	99.15730337		sp|Q6PAK3|ANM8_MOUSE	Protein arginine N-methyltransferase 8 OS=Mus musculus OX=10090 GN=Prmt8 PE=1 SV=2	0	71.988	93.25842697
P_KWMTBOMO15583		tr|D2Y4R2|D2Y4R2_BOMMO	Coatomer subunit alpha OS=Bombyx mori OX=7091 GN=COPA PE=2 SV=1	0	99.767	100		sp|Q27954|COPA_BOVIN	Coatomer subunit alpha OS=Bos taurus OX=9913 GN=COPA PE=1 SV=1	0	59.127	100
P_KWMTBOMO15592		tr|A3QVV0|A3QVV0_BOMMO	Small nuclear ribonucleoprotein Sm D2 OS=Bombyx mori OX=7091 PE=2 SV=1	1.84E-77	100	100		sp|Q9VI10|SMD2_DROME	Probable small nuclear ribonucleoprotein Sm D2 OS=Drosophila melanogaster OX=7227 GN=SmD2 PE=1 SV=1	9.05E-54	90.722	88.99082569
P_KWMTBOMO15928		tr|Q0N2S4|Q0N2S4_BOMMO	Polyadenylate-binding protein OS=Bombyx mori OX=7091 GN=PAbp PE=2 SV=1	0	99.502	98.52941176		sp|P11940|PABP1_HUMAN	Polyadenylate-binding protein 1 OS=Homo sapiens OX=9606 GN=PABPC1 PE=1 SV=2	0	68.454	100
P_KWMTBOMO16040		tr|H9J4W7|H9J4W7_BOMMO	Uncharacterized protein OS=Bombyx mori OX=7091 PE=3 SV=1	0	100	77.56410256		sp|P42669|PURA_MOUSE	Transcriptional activator protein Pur-alpha OS=Mus musculus OX=10090 GN=Pura PE=1 SV=1	1.27E-77	50.213	75.32051282
P_KWMTBOMO16637		tr|Q9GSB6|Q9GSB6_BOMMO	Heat shock protein hsp20.4 OS=Bombyx mori OX=7091 GN=Hsp20.4 PE=2 SV=2	5.98E-133	100	100		sp|P82147|L2EFL_DROME	Protein lethal(2)essential for life OS=Drosophila melanogaster OX=7227 GN=l(2)efl PE=1 SV=1	8.44E-55	50.568	97.23756906

unique PIPs of fibL at L5D5

Name	Bombyx-mori	Subject ID	Description	e-value	identity(%)	coverage(%)	Metazoa	Subject ID	Description	e-value	identity(%)	coverage(%)
P_KWMTBOMO00279		tr|H9ITU2|H9ITU2_BOMMO	Citrate synthase OS=Bombyx mori OX=7091 GN=101737646 PE=3 SV=1	0	100	100		sp|Q16P20|CISY2_AEDAE	Probable citrate synthase 2, mitochondrial OS=Aedes aegypti OX=7159 GN=AAEL011789 PE=3 SV=1	0	81.075	100
P_KWMTBOMO00373		tr|H9ITY4|H9ITY4_BOMMO	Aa_trans domain-containing protein OS=Bombyx mori OX=7091 GN=101746014 PE=4 SV=1	0	100	100		sp|Q9VT04|PATH_DROME	Proton-coupled amino acid transporter-like protein pathetic OS=Drosophila melanogaster OX=7227 GN=path PE=1 SV=1	6.88E-175	56.182	99.35344828
P_KWMTBOMO00375		tr|H9ITY5|H9ITY5_BOMMO	Uncharacterized protein OS=Bombyx mori OX=7091 PE=3 SV=1	0	87.972	100		sp|Q9VSA3|ACADM_DROME	Probable medium-chain specific acyl-CoA dehydrogenase, mitochondrial OS=Drosophila melanogaster OX=7227 GN=CG12262 PE=2 SV=1	0	73.558	98.34515366
P_KWMTBOMO00390		tr|H9ITC4|H9ITC4_BOMMO	Uncharacterized protein OS=Bombyx mori OX=7091 PE=4 SV=1	0	100	79.5620438		--	--	--	--	--
P_KWMTBOMO00473		tr|Q1HPX8|Q1HPX8_BOMMO	6-phosphogluconate dehydrogenase, decarboxylating OS=Bombyx mori OX=7091 PE=2 SV=1	0	100	100		sp|P52209|6PGD_HUMAN	6-phosphogluconate dehydrogenase, decarboxylating OS=Homo sapiens OX=9606 GN=PGD PE=1 SV=3	0	73.542	99.37888199
P_KWMTBOMO00722		tr|A0A0D6A6H2|A0A0D6A6H2_BOMMO	Methionine--tRNA ligase, cytoplasmic OS=Bombyx mori OX=7091 GN=BmMRS PE=2 SV=1	0	100	100		sp|Q6PF21|SYMC_XENLA	Methionine--tRNA ligase, cytoplasmic OS=Xenopus laevis OX=8355 GN=mars1 PE=2 SV=1	0	46.816	89.41058941
P_KWMTBOMO00837		tr|H9JJI3|H9JJI3_BOMMO	Proteasome subunit beta OS=Bombyx mori OX=7091 PE=3 SV=1	0	100	97.32824427		sp|P99026|PSB4_MOUSE	Proteasome subunit beta type-4 OS=Mus musculus OX=10090 GN=Psmb4 PE=1 SV=1	1.89E-88	49.799	95.03816794
P_KWMTBOMO00973		--	--	--	--	--		--	--	--	--	--
P_KWMTBOMO00982		tr|H9IVS2|H9IVS2_BOMMO	Uncharacterized protein OS=Bombyx mori OX=7091 PE=4 SV=1	9.80E-180	100	48.71287129		sp|Q9D0F3|LMAN1_MOUSE	Protein ERGIC-53 OS=Mus musculus OX=10090 GN=Lman1 PE=1 SV=1	1.46E-138	44.353	96.43564356
P_KWMTBOMO01420		tr|H9JHG2|H9JHG2_BOMMO	Complex I-SGDH OS=Bombyx mori OX=7091 PE=3 SV=1	4.71E-146	100	100		sp|Q0MQD8|NDUB5_PANTR	NADH dehydrogenase [ubiquinone] 1 beta subcomplex subunit 5, mitochondrial OS=Pan troglodytes OX=9598 GN=NDUFB5 PE=2 SV=1	5.40E-31	42.857	66.31578947
P_KWMTBOMO01423		tr|H9JHG0|H9JHG0_BOMMO	NADH-ubiquinone oxidoreductase 75 kDa subunit, mitochondrial OS=Bombyx mori OX=7091 PE=3 SV=1	0	98.518	100		sp|Q94511|NDUS1_DROME	NADH-ubiquinone oxidoreductase 75 kDa subunit, mitochondrial OS=Drosophila melanogaster OX=7227 GN=ND-75 PE=2 SV=3	0	64.393	100
P_KWMTBOMO01431		tr|H9JHF8|H9JHF8_BOMMO	Voltage-dependent anion-selective channel protein 3 OS=Bombyx mori OX=7091 GN=101739703 PE=3 SV=1	5.89E-131	100	100		sp|Q94920|VDAC_DROME	Voltage-dependent anion-selective channel OS=Drosophila melanogaster OX=7227 GN=porin PE=1 SV=3	8.71E-82	60.656	100
P_KWMTBOMO01438		tr|H9JHC9|H9JHC9_BOMMO	U-box domain-containing protein OS=Bombyx mori OX=7091 PE=3 SV=1	0	99.029	97.93977813		sp|Q9ES00|UBE4B_MOUSE	Ubiquitin conjugation factor E4 B OS=Mus musculus OX=10090 GN=Ube4b PE=1 SV=3	1.88E-46	30.886	73.37559429
P_KWMTBOMO01452		tr|H9JHD3|H9JHD3_BOMMO	Arginyl-tRNA synthetase OS=Bombyx mori OX=7091 GN=101737924 PE=3 SV=1	0	100	100		sp|Q6P1S4|SYRC_XENTR	Arginine--tRNA ligase, cytoplasmic OS=Xenopus tropicalis OX=8364 GN=rars1 PE=2 SV=1	0	58.85	99.26793558
P_KWMTBOMO01658		tr|H9J955|H9J955_BOMMO	Calcium channel flower OS=Bombyx mori OX=7091 PE=3 SV=1	2.66E-135	100	95.81151832		sp|B3M9W1|FLOWR_DROAN	Calcium channel flower OS=Drosophila ananassae OX=7217 GN=flower PE=3 SV=2	4.16E-59	49.735	98.95287958
P_KWMTBOMO01708		tr|H9J9C9|H9J9C9_BOMMO	Dynein light intermediate chain OS=Bombyx mori OX=7091 PE=3 SV=1	2.61E-111	99.367	34.05172414		sp|Q6PDL0|DC1L2_MOUSE	Cytoplasmic dynein 1 light intermediate chain 2 OS=Mus musculus OX=10090 GN=Dync1li2 PE=1 SV=2	2.12E-142	50.33	98.06034483

P_KWMTBOMO01806	tr|H9J9G9|H9J9G9_BOMMO	Cation-transporting ATPase OS=Bombyx mori OX=7091 GN=101743411 PE=3 SV=1	0	99.914	100	sp|Q9EPE9|AT131_MOUSE	Manganese-transporting ATPase 13A1 OS=Mus musculus OX=10090 GN=Atp13a1 PE=1 SV=2	0	57.252	100
P_KWMTBOMO01821	tr|H9J9H8|H9J9H8_BOMMO	Uncharacterized protein OS=Bombyx mori OX=7091 GN=101744329 PE=4 SV=1	0	100	89.2635315	sp|Q5ZJP5|FND3A_CHICK	Fibronectin type-III domain-containing protein 3a OS=Gallus gallus OX=9031 GN=FNDC3A PE=2 SV=2	0	33.302	95.65217391
P_KWMTBOMO02013	tr|H9J125|H9J125_BOMMO	26S proteasome non-ATPase regulatory subunit 6 OS=Bombyx mori OX=7091 GN=101735567 PE=3 SV=1	0	100	100	sp|Q15008|PSMD6_HUMAN	26S proteasome non-ATPase regulatory subunit 6 OS=Homo sapiens OX=9606 GN=PSMD6 PE=1 SV=1	5.45E-102	43.112	100
P_KWMTBOMO02088	tr|H9J0E7|H9J0E7_BOMMO	Lysine--tRNA ligase OS=Bombyx mori OX=7091 GN=101740883 PE=3 SV=1	0	100	100	sp|Q99MN1|SYK_MOUSE	Lysine--tRNA ligase OS=Mus musculus OX=10090 GN=Kars1 PE=1 SV=1	0	68.206	97.06896552
P_KWMTBOMO02223	tr|Q1HQC7|Q1HQC7_BOMMO	Aspartate aminotransferase OS=Bombyx mori OX=7091 PE=2 SV=1	0	99.768	100	sp|P00507|AATM_RAT	Aspartate aminotransferase, mitochondrial OS=Rattus norvegicus OX=10116 GN=Got2 PE=1 SV=2	0	62.06	92.34338747
P_KWMTBOMO02278	sp|Q75VN3|TCTP_BOMMO	Translationally-controlled tumor protein homolog OS=Bombyx mori OX=7091 GN=Tctp PE=2 SV=1	8.85E-126	100	100	sp|Q75VN3|TCTP_BOMMO	Translationally-controlled tumor protein homolog OS=Bombyx mori OX=7091 GN=Tctp PE=2 SV=1	5.77E-125	100	100
P_KWMTBOMO02354	tr|H9JXZ9|H9JXZ9_BOMMO	Uncharacterized protein OS=Bombyx mori OX=7091 PE=4 SV=1	0	97.674	41.28	sp|P45953|ACADV_RAT	Very long-chain specific acyl-CoA dehydrogenase, mitochondrial OS=Rattus norvegicus OX=10116 GN=Acadvl PE=1 SV=1	0	56.689	95.68
P_KWMTBOMO02392	sp|B9VJ80|UBA5_BOMMO	Ubiquitin-like modifier-activating enzyme 5 OS=Bombyx mori OX=7091 PE=2 SV=1	0	99.237	100	sp|B9VJ80|UBA5_BOMMO	Ubiquitin-like modifier-activating enzyme 5 OS=Bombyx mori OX=7091 PE=2 SV=1	0	99.237	100
P_KWMTBOMO02535	tr|H9IZB1|H9IZB1_BOMMO	Uncharacterized protein OS=Bombyx mori OX=7091 PE=4 SV=1	0	96.875	70.12687428	sp|O08730|GLYG_RAT	Glycogenin-1 OS=Rattus norvegicus OX=10116 GN=Gyg1 PE=2 SV=4	3.47E-117	52.395	38.52364475
P_KWMTBOMO02542	tr|Q8T7L8|Q8T7L8_BOMMO	Seroin 1 OS=Bombyx mori OX=7091 PE=2 SV=1	7.09E-73	97.222	100	--	--	--	--	--
P_KWMTBOMO02836	tr|H9J1Y2|H9J1Y2_BOMMO	Uncharacterized protein OS=Bombyx mori OX=7091 PE=3 SV=1	7.22E-124	90	100	sp|P55326|YZG1_CAEEL	Uncharacterized protein F13E6.1 OS=Caenorhabditis elegans OX=6239 GN=F13E6.1 PE=3 SV=2	1.51E-19	35.032	84.40860215
P_KWMTBOMO03002	tr|Q587N3|Q587N3_BOMMO	Protein disulfide-isomerase OS=Bombyx mori OX=7091 GN=erp57 PE=2 SV=1	0	100	100	sp|Q8JG64|PDIA3_CHICK	Protein disulfide-isomerase A3 OS=Gallus gallus OX=9031 GN=PDIA3 PE=2 SV=1	4.56E-149	50.644	94.90835031
P_KWMTBOMO03003	tr|H9J272|H9J272_BOMMO	Adenylate kinase isoenzyme 6 homolog OS=Bombyx mori OX=7091 GN=101735934 PE=3 SV=1	2.60E-133	100	100	sp|Q7JYV7|KAD6_DROME	Adenylate kinase isoenzyme 6 homolog OS=Drosophila melanogaster OX=7227 GN=Ak6 PE=1 SV=1	3.13E-69	57.317	93.71428571
P_KWMTBOMO03027	tr|Q2F5M6|Q2F5M6_BOMMO	Ras small monomeric GTPase Rab6 OS=Bombyx mori OX=7091 PE=2 SV=1	1.13E-158	100	100	sp|O18334|RAB6_DROME	Ras-related protein Rab6 OS=Drosophila melanogaster OX=7227 GN=Rab6 PE=1 SV=1	2.75E-141	90.338	99.0430622
P_KWMTBOMO03301	tr|E0D4V7|E0D4V7_BOMMO	Phenylalanyl-tRNA synthetase beta subunit OS=Bombyx mori OX=7091 GN=FRSB PE=2 SV=1	0	100	100	sp|Q9VCA5|SYFB_DROME	Phenylalanine--tRNA ligase beta subunit OS=Drosophila melanogaster OX=7227 GN=beta-PheRS PE=1 SV=1	0	66.893	100
P_KWMTBOMO03311	tr|H9JAB8|H9JAB8_BOMMO	RRM domain-containing protein OS=Bombyx mori OX=7091 PE=4 SV=1	0	100	100	sp|P23588|IF4B_HUMAN	Eukaryotic translation initiation factor 4B OS=Homo sapiens OX=9606 GN=EIF4B PE=1 SV=2	2.09E-33	35.971	77.79850746
P_KWMTBOMO03622	tr|H9J9Y4|H9J9Y4_BOMMO	ADP-ribosylation factor-like protein 6-interacting protein 4 OS=Bombyx mori OX=7091 GN=101742090 PE=3 SV=1	8.34E-108	100	98.14814815	sp|Q5F4A9|AR6P4_CHICK	ADP-ribosylation factor-like protein 6-interacting protein 4 OS=Gallus gallus OX=9031 GN=ARL6IP4 PE=2 SV=1	4.30E-31	76.119	41.35802469
P_KWMTBOMO04051	tr|H9JL88|H9JL88_BOMMO	UDPGT domain-containing protein OS=Bombyx mori OX=7091 PE=3 SV=1	0	95.709	99.9002991	sp|A0A291PQH4|UGT2_DACCO	UDP-glucosyltransferase 2 OS=Dactylopius coccus OX=765876 GN=UTG2 PE=1 SV=1	6.72E-74	31.836	51.04685942
P_KWMTBOMO04057	tr|G9LPU0|G9LPU0_BOMMO	UDP-glucuronosyltransferase OS=Bombyx mori OX=7091 GN=UGT40A1 PE=2 SV=1	0	100	100	sp|A0A291PQH4|UGT2_DACCO	UDP-glucosyltransferase 2 OS=Dactylopius coccus OX=765876 GN=UTG2 PE=1 SV=1	1.37E-77	32.544	97.5
P_KWMTBOMO04170	tr|H9JGS8|H9JGS8_BOMMO	UTP--glucose-1-phosphate uridylyltransferase OS=Bombyx mori OX=7091 PE=3 SV=1	0	100	97.85575049	sp|Q07130|UGPA_BOVIN	UTP--glucose-1-phosphate uridylyltransferase OS=Bos taurus OX=9913 GN=UGP2 PE=1 SV=2	0	65.737	97.85575049
P_KWMTBOMO04330	tr|H9JX71|H9JX71_BOMMO	PCI domain-containing protein OS=Bombyx mori OX=7091 GN=101735320 PE=3 SV=1	0	100	100	sp|Q7KLV9|PSD11_DROME	26S proteasome non-ATPase regulatory subunit 11 OS=Drosophila melanogaster OX=7227 GN=Rpn6 PE=1 SV=1	0	76.54	100
P_KWMTBOMO04424	tr|H9J7A0|H9J7A0_BOMMO	PA28_alpha domain-containing protein OS=Bombyx mori OX=7091 PE=4 SV=1	3.58E-90	99.225	52.43902439	sp|Q5F3J5|PSME3_CHICK	Proteasome activator complex subunit 3 OS=Gallus gallus OX=9031 GN=PSME3 PE=1 SV=1	5.95E-92	50.201	100
P_KWMTBOMO04427	tr|H9J7B7|H9J7B7_BOMMO	Signal recognition particle receptor subunit beta OS=Bombyx mori OX=7091 PE=3 SV=1	0	100	100	sp|Q9Y5M8|SRPRB_HUMAN	Signal recognition particle receptor subunit beta OS=Homo sapiens OX=9606 GN=SRPRB PE=1 SV=3	2.92E-59	46.575	87.6
P_KWMTBOMO04469	tr|Q5TLD2|Q5TLD2_BOMMO	Replication protein A middle subunit OS=Bombyx mori OX=7091 GN=BmRPA2 PE=2 SV=1	0	100	100	sp|Q63528|RFA2_RAT	Replication protein A 32 kDa subunit OS=Rattus norvegicus OX=10116 GN=Rpa2 PE=2 SV=2	1.69E-31	29.963	100
P_KWMTBOMO04584	tr|Q2F5K1|Q2F5K1_BOMMO	Signal sequence receptor subunit gamma OS=Bombyx mori OX=7091 PE=2 SV=1	1.07E-132	100	100	sp|Q9UNL2|SSRG_HUMAN	Translocon-associated protein subunit gamma OS=Homo sapiens OX=9606 GN=SSR3 PE=1 SV=1	8.00E-87	64.865	100
P_KWMTBOMO04594	tr|Q1HE02|Q1HE02_BOMMO	ALY OS=Bombyx mori OX=7091 GN=733049 PE=2 SV=1	0	100	100	sp|Q58EA2|THO4A_XENLA	THO complex subunit 4-A OS=Xenopus laevis OX=8355 GN=alyref-a PE=2 SV=1	3.73E-51	49.237	100
P_KWMTBOMO04674	tr|Q1HQ86|Q1HQ86_BOMMO	Mobility group protein 1B OS=Bombyx mori OX=7091 GN=100101161 PE=2 SV=1	3.17E-78	100	100	sp|Q06943|HMGZ_DROME	High mobility group protein Z OS=Drosophila melanogaster OX=7227 GN=HmgZ PE=1 SV=1	5.24E-33	66.234	64.70588235
P_KWMTBOMO04737	tr|H9JK23|H9JK23_BOMMO	Carboxylic ester hydrolase OS=Bombyx mori OX=7091 PE=3 SV=1	0	99.538	95.58498896	sp|B2D0J5|EST6_APIME	Venom carboxylesterase-6 OS=Apis mellifera OX=7460 PE=2 SV=1	1.76E-118	41.256	98.45474614
P_KWMTBOMO04906	tr|H9JEW9|H9JEW9_BOMMO	Aminopeptidase N-8 OS=Bombyx mori OX=7091 GN=101737565 PE=2 SV=1	0	98.024	92.42009132	sp|P15145|AMPN_PIG	Aminopeptidase N OS=Sus scrofa OX=9823 GN=ANPEP PE=1 SV=4	9.30E-128	32.057	76.34703196
P_KWMTBOMO04966	tr|E5EVW5|E5EVW5_BOMMO	Inorganic diphosphatase OS=Bombyx mori OX=7091 PE=3 SV=1	0	100	86.22754491	sp|O77460|IPYR_DROME	Inorganic pyrophosphatase OS=Drosophila melanogaster OX=7227 GN=Nurf-38 PE=1 SV=3	1.04E-129	58.389	89.22155689

P_KWMTBOMO04992	tr|H9JEZ8|H9JEZ8_BOMMO	Uncharacterized protein OS=Bombyx mori OX=7091 PE=3 SV=1	0	99.614	100	sp|Q21929|DCXR_CAEEL	L-xylulose reductase OS=Caenorhabditis elegans OX=6239 GN=dhs-21 PE=1 SV=2	5.68E-38	33.597	97.68339768
P_KWMTBOMO05078	tr|Q2F5L1|Q2F5L1_BOMMO	Signal peptidase complex catalytic subunit SEC11 OS=Bombyx mori OX=7091 PE=2 SV=1	3.67E-131	99.438	100	sp|Q9D8V7|SC11C_MOUSE	Signal peptidase complex catalytic subunit SEC11C OS=Mus musculus OX=10090 GN=Sec11c PE=1 SV=3	1.20E-103	79.429	98.31460674
P_KWMTBOMO05113	tr|H9JSJ4|H9JSJ4_BOMMO	Uncharacterized protein OS=Bombyx mori OX=7091 PE=4 SV=1	0	100	100	sp|P38659|PDIA4_RAT	Protein disulfide-isomerase A4 OS=Rattus norvegicus OX=10116 GN=Pdia4 PE=1 SV=2	2.64E-09	23.81	66.66666667
P_KWMTBOMO05178	tr|Q2F5M8|Q2F5M8_BOMMO	Phosphoserine aminotransferase OS=Bombyx mori OX=7091 PE=2 SV=1	0	100	100	sp|Q9VAN0|SERC_DROME	Probable phosphoserine aminotransferase OS=Drosophila melanogaster OX=7227 GN=CG11899 PE=2 SV=1	9.90E-147	64.935	96.85534591
P_KWMTBOMO05205	tr|H9IYX7|H9IYX7_BOMMO	5-aminoimidazole-4-carboxamide ribonucleotide formyltransferase OS=Bombyx mori OX=7091 PE=3 SV=1	0	100	98.81556684	sp|P31335|PUR9_CHICK	Bifunctional purine biosynthesis protein ATIC OS=Gallus gallus OX=9031 GN=ATIC PE=1 SV=1	0	68.855	100
P_KWMTBOMO05281	tr|H9IZ19|H9IZ19_BOMMO	Uncharacterized protein OS=Bombyx mori OX=7091 GN=101735890 PE=3 SV=1	4.73E-144	100	100	sp|Q9CQC9|SAR1B_MOUSE	GTP-binding protein SAR1b OS=Mus musculus OX=10090 GN=Sar1b PE=1 SV=1	9.93E-107	75	100
P_KWMTBOMO05299	tr|H9IYR7|H9IYR7_BOMMO	Uncharacterized protein OS=Bombyx mori OX=7091 PE=3 SV=1	0	96.091	98.08306709	sp|O95749|GGPPS_HUMAN	Geranylgeranyl pyrophosphate synthase OS=Homo sapiens OX=9606 GN=GGPS1 PE=1 SV=1	5.33E-131	60.357	89.45686901
P_KWMTBOMO05464	tr|H9JB09|H9JB09_BOMMO	Vasa intronic protein OS=Bombyx mori OX=7091 GN=101741313 PE=2 SV=1	0	100	100	sp|Q6AXS5|PAIRB_RAT	Plasminogen activator inhibitor 1 RNA-binding protein OS=Rattus norvegicus OX=10116 GN=Serbp1 PE=1 SV=2	1.23E-20	40.625	41.1311054
P_KWMTBOMO05699	tr|H9JBD3|H9JBD3_BOMMO	RNA-splicing ligase RtcB homolog OS=Bombyx mori OX=7091 GN=101739631 PE=3 SV=1	0	100	100	sp|Q9VIW7|RTCB_DROME	RNA-splicing ligase RtcB homolog OS=Drosophila melanogaster OX=7227 GN=RtcB PE=2 SV=1	0	88.34	100
P_KWMTBOMO05810	tr|H9JBQ0|H9JBQ0_BOMMO	Uncharacterized protein OS=Bombyx mori OX=7091 PE=4 SV=1	0	99.381	50.35650624	sp|Q8SWU7|OLA1_DROME	Obg-like ATPase 1 OS=Drosophila melanogaster OX=7227 GN=CG1354 PE=1 SV=1	4.43E-180	78.274	14.97326203
P_KWMTBOMO05987	tr|A0FDQ5|A0FDQ5_BOMMO	Dolichyl-diphosphooligosaccharide--protein glycosyltransferase 48 kDa subunit OS=Bombyx mori OX=7091 PE=2 SV=1	0	99.541	100	sp|Q24319|OST48_DROME	Dolichyl-diphosphooligosaccharide--protein glycosyltransferase 48 kDa subunit OS=Drosophila melanogaster OX=7227 GN=Ost48 PE=2 SV=2	0	65.707	95.64220183
P_KWMTBOMO05993	tr|H9IZY3|H9IZY3_BOMMO	Uncharacterized protein OS=Bombyx mori OX=7091 PE=3 SV=1	0	99.777	90.89068826	sp|P91938|TRXR1_DROME	Thioredoxin reductase 1, mitochondrial OS=Drosophila melanogaster OX=7227 GN=Trxr-1 PE=1 SV=2	0	67.677	100
P_KWMTBOMO06045	tr|H9J005|H9J005_BOMMO	TOG domain-containing protein OS=Bombyx mori OX=7091 PE=3 SV=2	0	93.348	100	sp|E9PVA8|GCN1_MOUSE	eIF-2-alpha kinase activator GCN1 OS=Mus musculus OX=10090 GN=Gcn1 PE=1 SV=1	0	45.751	100
P_KWMTBOMO06228	tr|H9IWL1|H9IWL1_BOMMO	LITAF domain-containing protein OS=Bombyx mori OX=7091 PE=3 SV=1	1.49E-80	99.153	65.55555556	--	--	--	--	--
P_KWMTBOMO06354	tr|H9JR23|H9JR23_BOMMO	Aminotran_1_2 domain-containing protein OS=Bombyx mori OX=7091 PE=4 SV=1	0	94.624	90.11627907	sp|Q28DB5|ALAT2_XENTR	Alanine aminotransferase 2 OS=Xenopus tropicalis OX=8364 GN=gpt2 PE=2 SV=1	0	58.078	94.76744186
P_KWMTBOMO06427	tr|Q2F5L0|Q2F5L0_BOMMO	Signal sequence receptor subunit alpha OS=Bombyx mori OX=7091 GN=778461 PE=2 SV=1	0	100	100	sp|P43307|SSRA_HUMAN	Translocon-associated protein subunit alpha OS=Homo sapiens OX=9606 GN=SSR1 PE=1 SV=3	6.17E-70	50.45	79.56989247
P_KWMTBOMO06639	tr|Q2F5Q1|Q2F5Q1_BOMMO	Lysophospholipase OS=Bombyx mori OX=7091 PE=2 SV=1	6.85E-166	100	100	sp|Q3MHR0|LYPA1_BOVIN	Acyl-protein thioesterase 1 OS=Bos taurus OX=9913 GN=LYPLA1 PE=2 SV=1	6.45E-78	53.425	99.54545455
P_KWMTBOMO06654	tr|A0FDQ0|A0FDQ0_BOMMO	Programmed cell death protein 5-like protein OS=Bombyx mori OX=7091 GN=778501 PE=2 SV=1	6.72E-92	100	100	sp|P56812|PDCD5_MOUSE	Programmed cell death protein 5 OS=Mus musculus OX=10090 GN=Pdcd5 PE=1 SV=3	6.66E-31	46.512	99.23076923
P_KWMTBOMO06762	tr|H9JRG5|H9JRG5_BOMMO	Uncharacterized protein OS=Bombyx mori OX=7091 PE=4 SV=1	1.50E-169	100	100	sp|Q9BRJ6|CG050_HUMAN	Uncharacterized protein C7orf50 OS=Homo sapiens OX=9606 GN=C7orf50 PE=1 SV=1	5.26E-14	35.417	41.55844156
P_KWMTBOMO07061	tr|H9JLP3|H9JLP3_BOMMO	Uncharacterized protein OS=Bombyx mori OX=7091 PE=3 SV=1	2.05E-51	100	50.31055901	sp|Q5R979|PGM2L_PONAB	Glucose 1,6-bisphosphate synthase OS=Pongo abelii OX=9601 GN=PGM2L1 PE=2 SV=1	4.81E-32	37.5	99.37888199
P_KWMTBOMO07103	tr|Q2F5P8|Q2F5P8_BOMMO	Malate dehydrogenase OS=Bombyx mori OX=7091 PE=2 SV=1	0	100	100	sp|O88989|MDHC_RAT	Malate dehydrogenase, cytoplasmic OS=Rattus norvegicus OX=10116 GN=Mdh1 PE=1 SV=3	7.95E-157	65.653	99.39577039
P_KWMTBOMO07205	tr|H9JLI0|H9JLI0_BOMMO	Uncharacterized protein OS=Bombyx mori OX=7091 PE=3 SV=1	0	98.254	100	sp|Q24179|SLY1_DROME	Protein sly1 homolog OS=Drosophila melanogaster OX=7227 GN=Slh PE=2 SV=4	0	62.813	100
P_KWMTBOMO07214	tr|D5MTP2|D5MTP2_BOMMO	Bm8 interacting protein 2-11 OS=Bombyx mori OX=7091 PE=2 SV=1	0	94.898	78.82037534	sp|Q9VMA7|TGO1_DROME	Transport and Golgi organization protein 1 OS=Drosophila melanogaster OX=7227 GN=Tango1 PE=1 SV=2	6.97E-56	30.097	48.32439678
P_KWMTBOMO07368	tr|H9JMD1|H9JMD1_BOMMO	Uncharacterized protein OS=Bombyx mori OX=7091 GN=101744608 PE=3 SV=1	8.23E-94	100	100	sp|P24310|CX7A1_HUMAN	Cytochrome c oxidase subunit 7A1, mitochondrial OS=Homo sapiens OX=9606 GN=COX7A1 PE=1 SV=2	3.33E-12	47.458	45.38461538
P_KWMTBOMO07722	tr|H9IVI5|H9IVI5_BOMMO	Eukaryotic translation initiation factor 5B OS=Bombyx mori OX=7091 PE=4 SV=1	2.43E-114	98.802	82.67326733	sp|Q05D44|IF2P_MOUSE	Eukaryotic translation initiation factor 5B OS=Mus musculus OX=10090 GN=Eif5b PE=1 SV=2	4.35E-111	76.238	100
P_KWMTBOMO07723	tr|H9IVI5|H9IVI5_BOMMO	Eukaryotic translation initiation factor 5B OS=Bombyx mori OX=7091 PE=4 SV=1	0	96.413	100	sp|O60841|IF2P_HUMAN	Eukaryotic translation initiation factor 5B OS=Homo sapiens OX=9606 GN=EIF5B PE=1 SV=4	1.08E-159	65.746	39.34782609
P_KWMTBOMO07761	tr|H9IUG4|H9IUG4_BOMMO	Uncharacterized protein OS=Bombyx mori OX=7091 PE=4 SV=1	0	94.605	26.40722724	sp|Q8MSS1|LVA_DROME	Protein lava lamp OS=Drosophila melanogaster OX=7227 GN=lva PE=1 SV=2	5.58E-15	35.644	7.01876303
P_KWMTBOMO07821	tr|H9IUJ6|H9IUJ6_BOMMO	Transaldolase OS=Bombyx mori OX=7091 PE=3 SV=1	0	100	100	sp|Q2TBL6|TALDO_BOVIN	Transaldolase OS=Bos taurus OX=9913 GN=TALDO1 PE=2 SV=1	4.86E-159	68.598	98.79518072
P_KWMTBOMO08040	tr|H9IUV5|H9IUV5_BOMMO	Inosine-5'-monophosphate dehydrogenase OS=Bombyx mori OX=7091 GN=101740233 PE=3 SV=1	0	100	100	sp|Q07152|IMDH_DROME	Inosine-5'-monophosphate dehydrogenase OS=Drosophila melanogaster OX=7227 GN=ras PE=1 SV=1	0	79.093	99.0234375

P_KWMTBOMO08045	tr|H9IUV7|H9IUV7_BOMMO	Leucyl-tRNA synthetase OS=Bombyx mori OX=7091 PE=3 SV=1	0	99.658	99.20724802	sp|Q9P2J5|SYLC_HUMAN	Leucine--tRNA ligase, cytoplasmic OS=Homo sapiens OX=9606 GN=LARS1 PE=1 SV=2	0	65.984	99.54699887
P_KWMTBOMO08056	tr|H9IV39|H9IV39_BOMMO	Rab GDP dissociation inhibitor OS=Bombyx mori OX=7091 GN=101738804 PE=3 SV=1	0	100	100	sp|P21856|GDIA_BOVIN	Rab GDP dissociation inhibitor alpha OS=Bos taurus OX=9913 GN=GDI1 PE=1 SV=1	0	68.75	100
P_KWMTBOMO08098	tr|G9FL14|G9FL14_BOMMO	DNA supercoiling factor OS=Bombyx mori OX=7091 GN=LOC692759 PE=2 SV=1	1.98E-48	100	100	sp|B5X4E0|CALUB_SALSA	Calumenin-B OS=Salmo salar OX=8030 GN=calub PE=2 SV=1	7.32E-13	49.333	100
P_KWMTBOMO08204	tr|H9JIZ5|H9JIZ5_BOMMO	Arf-GAP domain-containing protein OS=Bombyx mori OX=7091 GN=101746692 PE=4 SV=1	0	100	100	sp|Q28CM8|ARFG2_XENTR	ADP-ribosylation factor GTPase-activating protein 2 OS=Xenopus tropicalis OX=8364 GN=arfgap2 PE=2 SV=1	1.27E-99	39.455	100
P_KWMTBOMO08343	tr|H9JI86|H9JI86_BOMMO	Uncharacterized protein OS=Bombyx mori OX=7091 PE=4 SV=1	0	100	71.51898734	sp|P54802|ANAG_HUMAN	Alpha-N-acetylglucosaminidase OS=Homo sapiens OX=9606 GN=NAGLU PE=1 SV=2	2.63E-165	41.81	67.61603376
P_KWMTBOMO08503	tr|H9JIK9|H9JIK9_BOMMO	Mitochondrial DNA polymerase catalytic subunit OS=Bombyx mori OX=7091 PE=3 SV=1	0	100	96.68246445	sp|Q9BXS5|AP1M1_HUMAN	AP-1 complex subunit mu-1 OS=Homo sapiens OX=9606 GN=AP1M1 PE=1 SV=3	0	84.123	100
P_KWMTBOMO08584	tr|H9JDK7|H9JDK7_BOMMO	Uncharacterized protein OS=Bombyx mori OX=7091 PE=4 SV=1	0	99.913	57.49373434	sp|O15027|SC16A_HUMAN	Protein transport protein Sec16A OS=Homo sapiens OX=9606 GN=SEC16A PE=1 SV=4	3.13E-60	34.081	22.35588972
P_KWMTBOMO08596	tr|H9JEM4|H9JEM4_BOMMO	Uncharacterized protein OS=Bombyx mori OX=7091 PE=3 SV=1	1.43E-162	100	97.69585253	sp|Q7SXP0|S22BB_DANRE	Vesicle-trafficking protein SEC22b-B OS=Danio rerio OX=7955 GN=sec22bb PE=2 SV=1	4.44E-87	56.621	100
P_KWMTBOMO08655	tr|Q2F649|Q2F649_BOMMO	Surfeit 4-like protein OS=Bombyx mori OX=7091 PE=2 SV=1	0	100	100	sp|O18405|SURF4_DROME	Surfeit locus protein 4 homolog OS=Drosophila melanogaster OX=7227 GN=Surf4 PE=2 SV=1	2.43E-168	81.852	100
P_KWMTBOMO08707	tr|H9JDR8|H9JDR8_BOMMO	Elongator complex protein 3 OS=Bombyx mori OX=7091 PE=3 SV=1	0	99.66	82.4684432	sp|Q5ZHS1|ELP3_CHICK	Elongator complex protein 3 OS=Gallus gallus OX=9031 GN=ELP3 PE=2 SV=1	0	84.962	74.61430575
P_KWMTBOMO08773	tr|H9JDU2|H9JDU2_BOMMO	TROVE domain-containing protein OS=Bombyx mori OX=7091 PE=3 SV=1	0	96.22	100	sp|P42700|RO60_XENLA	60 kDa SS-A/Ro ribonucleoprotein OS=Xenopus laevis OX=8355 GN=ro60 PE=1 SV=1	6.33E-37	24.324	99.05511811
P_KWMTBOMO08894	tr|Q5UAN9|Q5UAN9_BOMMO	Ribosomal protein S5 OS=Bombyx mori OX=7091 GN=RpS5 PE=2 SV=1	3.62E-166	100	100	sp|P46782|RS5_HUMAN	40S ribosomal protein S5 OS=Homo sapiens OX=9606 GN=RPS5 PE=1 SV=4	1.84E-134	91.327	89.49771689
P_KWMTBOMO09183	tr|H9J1H4|H9J1H4_BOMMO	Uncharacterized protein OS=Bombyx mori OX=7091 PE=4 SV=1	0	99.804	46.6179159	sp|O00410|IPO5_HUMAN	Importin-5 OS=Homo sapiens OX=9606 GN=IPO5 PE=1 SV=4	0	52.516	99.90859232
P_KWMTBOMO09232	tr|Q5UAS9|Q5UAS9_BOMMO	Ribosomal protein L10A OS=Bombyx mori OX=7091 GN=RpL10A PE=2 SV=1	4.16E-162	100	100	sp|Q963B6|RL10A_SPOFR	60S ribosomal protein L10a OS=Spodoptera frugiperda OX=7108 GN=RpL10A PE=2 SV=1	1.14E-156	96.774	100
P_KWMTBOMO09474	tr|H9JU51|H9JU51_BOMMO	Malate dehydrogenase OS=Bombyx mori OX=7091 GN=101736985 PE=3 SV=1	0	100	100	sp|P40926|MDHM_HUMAN	Malate dehydrogenase, mitochondrial OS=Homo sapiens OX=9606 GN=MDH2 PE=1 SV=3	4.35E-146	60.294	99.70674487
P_KWMTBOMO09553	tr|Q1HPN7|Q1HPN7_BOMMO	Fructose-bisphosphate aldolase OS=Bombyx mori OX=7091 GN=778467 PE=2 SV=1	0	100	100	sp|P07764|ALF_DROME	Fructose-bisphosphate aldolase OS=Drosophila melanogaster OX=7227 GN=Ald1 PE=1 SV=5	0	82.143	100
P_KWMTBOMO09631	tr|H9JTW9|H9JTW9_BOMMO	GOLD domain-containing protein OS=Bombyx mori OX=7091 GN=101737070 PE=3 SV=1	5.12E-155	100	100	sp|P49020|TMED2_CRIGR	Transmembrane emp24 domain-containing protein 2 (Fragment) OS=Cricetulus griseus OX=10029 GN=TMED2 PE=1 SV=1	4.26E-87	63.587	90.19607843
P_KWMTBOMO09641	tr|Q2F671|Q2F671_BOMMO	NIPSNAP protein OS=Bombyx mori OX=7091 PE=2 SV=1	0	100	97.8021978	sp|Q9VXK0|NIPSN_DROME	Protein NipSnap OS=Drosophila melanogaster OX=7227 GN=Nipsnap PE=2 SV=2	9.40E-141	68.498	100
P_KWMTBOMO09657	tr|H9JTV5|H9JTV5_BOMMO	Uncharacterized protein OS=Bombyx mori OX=7091 PE=4 SV=1	0	100	94.05829596	sp|Q15020|SART3_HUMAN	Squamous cell carcinoma antigen recognized by T-cells 3 OS=Homo sapiens OX=9606 GN=SART3 PE=1 SV=1	3.10E-76	27.373	100
P_KWMTBOMO09672	tr|H9JTU8|H9JTU8_BOMMO	Uncharacterized protein OS=Bombyx mori OX=7091 PE=4 SV=1	7.15E-83	98.276	42.64705882	sp|P48738|PIPNA_RABIT	Phosphatidylinositol transfer protein alpha isoform OS=Oryctolagus cuniculus OX=9986 GN=PITPNA PE=3 SV=2	1.85E-115	59.928	100
P_KWMTBOMO09811	tr|H9JUC9|H9JUC9_BOMMO	PH domain-containing protein OS=Bombyx mori OX=7091 PE=4 SV=1	0	92.813	66.07869742	sp|P22059|OSBP1_HUMAN	Oxysterol-binding protein 1 OS=Homo sapiens OX=9606 GN=OSBP PE=1 SV=1	0	49.396	100
P_KWMTBOMO09838	tr|H9JUC0|H9JUC0_BOMMO	Asparagine--tRNA ligase OS=Bombyx mori OX=7091 PE=3 SV=1	0	99.104	100	sp|Q2KJG3|SYNC_BOVIN	Asparagine--tRNA ligase, cytoplasmic OS=Bos taurus OX=9913 GN=NARS PE=2 SV=3	0	70.599	99.45848375
P_KWMTBOMO10129	tr|Q2F5X3|Q2F5X3_BOMMO	26S proteasome non-ATPase regulatory subunit 4 OS=Bombyx mori OX=7091 GN=778515 PE=2 SV=1	0	100	100	sp|P55035|PSMD4_DROME	26S proteasome non-ATPase regulatory subunit 4 OS=Drosophila melanogaster OX=7227 GN=Rpn10 PE=1 SV=2	1.63E-145	60.677	100
P_KWMTBOMO10211	tr|Q5UAQ9|Q5UAQ9_BOMMO	60S ribosomal protein L29 OS=Bombyx mori OX=7091 GN=RpL29 PE=2 SV=1	7.51E-48	100	100	sp|Q24154|RL29_DROME	60S ribosomal protein L29 OS=Drosophila melanogaster OX=7227 GN=RpL29 PE=1 SV=1	3.02E-27	65.278	98.63013699
P_KWMTBOMO10212	tr|Q0N2R7|Q0N2R7_BOMMO	Signal recognition particle subunit SRP68 OS=Bombyx mori OX=7091 GN=SRP68 PE=2 SV=1	0	100	100	sp|Q9VSS2|SRP68_DROME	Signal recognition particle subunit SRP68 OS=Drosophila melanogaster OX=7227 GN=Srp68 PE=2 SV=1	0	50.413	100
P_KWMTBOMO10310	tr|H9JCL3|H9JCL3_BOMMO	Uncharacterized protein OS=Bombyx mori OX=7091 PE=3 SV=1	1.43E-167	100	92.62295082	sp|Q91XV4|DCXR_MESAU	L-xylulose reductase OS=Mesocricetus auratus OX=10036 GN=DCXR PE=1 SV=1	3.99E-99	55.328	100
P_KWMTBOMO10337	tr|H9JCI5|H9JCI5_BOMMO	ERF-3 OS=Bombyx mori OX=7091 PE=4 SV=1	0	100	96.32352941	sp|Q8R050|ERF3A_MOUSE	Eukaryotic peptide chain release factor GTP-binding subunit ERF3A OS=Mus musculus OX=10090 GN=Gspt1 PE=1 SV=2	0	66.599	90.25735294
P_KWMTBOMO10397	tr|H9JBY9|H9JBY9_BOMMO	Uncharacterized protein OS=Bombyx mori OX=7091 PE=3 SV=1	0	82.394	90.59011164	sp|P52186|KCNJ2_CHICK	Inward rectifier potassium channel 2 OS=Gallus gallus OX=9031 GN=KCNJ2 PE=2 SV=1	1.92E-127	49.596	59.17065391
P_KWMTBOMO10661	tr|H9JFF9|H9JFF9_BOMMO	Uncharacterized protein OS=Bombyx mori OX=7091 PE=4 SV=1	0	99.829	69.84505364	sp|B4J9K1|BOP1_DROGR	Ribosome biogenesis protein BOP1 homolog OS=Drosophila grimshawi OX=7222 GN=GH22027 PE=3 SV=1	0	57.698	76.63885578
P_KWMTBOMO10779	tr|Q0N2R5|Q0N2R5_BOMMO	Signal recognition particle receptor alpha subunit OS=Bombyx mori OX=7091 PE=2 SV=1	0	99.51	100	sp|Q9U5L1|SRPRA_DROME	Signal recognition particle receptor subunit alpha homolog OS=Drosophila melanogaster OX=7227 GN=SrpRalpha PE=1 SV=2	0	59.486	100

P_KWMTBOMO10965	tr|Q1HQC6|Q1HQC6_BOMMO	26S protease regulatory subunit 6B OS=Bombyx mori OX=7091 PE=2 SV=1	0	100	100	sp|P46507|PRS6B_MANSE	26S proteasome regulatory subunit 6B OS=Manduca sexta OX=7130 PE=2 SV=1	0	98.313	100
P_KWMTBOMO11021	tr|Q5UAM3|Q5UAM3_BOMMO	Ribosomal protein S19 OS=Bombyx mori OX=7091 GN=RpS19 PE=2 SV=1	6.33E-111	100	100	sp|P39018|RS19A_DROME	40S ribosomal protein S19a OS=Drosophila melanogaster OX=7227 GN=RpS19a PE=1 SV=3	5.94E-71	66.892	96.73202614
P_KWMTBOMO11110	tr|Q0ZB74|Q0ZB74_BOMMO	Eukaryotic translation initiation factor 5 OS=Bombyx mori OX=7091 PE=2 SV=1	0	100	100	sp|Q9VXK6|IF5_DROME	Eukaryotic translation initiation factor 5 OS=Drosophila melanogaster OX=7227 GN=eIF5 PE=1 SV=1	3.65E-164	54.699	89.63282937
P_KWMTBOMO11316	tr|H9IX59|H9IX59_BOMMO	Uncharacterized protein OS=Bombyx mori OX=7091 PE=3 SV=1	0	98.19	100	sp|A4FV45|TM214_BOVIN	Transmembrane protein 214 OS=Bos taurus OX=9913 GN=TMEM214 PE=2 SV=1	1.34E-63	29.485	84.91704374
P_KWMTBOMO11336	tr|H9IX66|H9IX66_BOMMO	Threonyl-tRNA synthetase OS=Bombyx mori OX=7091 PE=3 SV=1	0	98.151	98.59747546	sp|Q3ZBV8|SYTC_BOVIN	Threonine--tRNA ligase 1, cytoplasmic OS=Bos taurus OX=9913 GN=TARS1 PE=2 SV=1	0	69.371	100
P_KWMTBOMO11416	tr|H9IXG0|H9IXG0_BOMMO	ATPase ASNA1 homolog OS=Bombyx mori OX=7091 GN=101735717 PE=3 SV=1	0	100	100	sp|B0WEV5|ASNA_CULQU	ATPase ASNA1 homolog OS=Culex quinquefasciatus OX=7176 GN=CPIJ005690 PE=3 SV=1	0	80.428	97.6119403
P_KWMTBOMO11570	tr|H9J3K3|H9J3K3_BOMMO	Midasin OS=Bombyx mori OX=7091 PE=3 SV=1	0	99.922	95.86361096	sp|Q9NU22|MDN1_HUMAN	Midasin OS=Homo sapiens OX=9606 GN=MDN1 PE=1 SV=2	0	45.946	44.12148314
P_KWMTBOMO11741	tr|H9J3V7|H9J3V7_BOMMO	Uncharacterized protein OS=Bombyx mori OX=7091 PE=3 SV=1	1.81E-180	99.19	98.4063745	sp|Q8TCT9|HM13_HUMAN	Minor histocompatibility antigen H13 OS=Homo sapiens OX=9606 GN=HM13 PE=1 SV=1	2.27E-101	62.832	90.03984064
P_KWMTBOMO11742	tr|H9J3V7|H9J3V7_BOMMO	Uncharacterized protein OS=Bombyx mori OX=7091 PE=3 SV=1	2.40E-68	100	100	sp|Q8TCT9|HM13_HUMAN	Minor histocompatibility antigen H13 OS=Homo sapiens OX=9606 GN=HM13 PE=1 SV=1	2.24E-37	59.804	99.02912621
P_KWMTBOMO11772	tr|H9ISG1|H9ISG1_BOMMO	Dolichyl-diphosphooligosaccharide--protein glycosyltransferase subunit DAD1 OS=Bombyx mori OX=7091 GN=101737303 PE=3 SV=1	2.45E-79	100	100	sp|Q9VLM5|DAD1_DROME	Dolichyl-diphosphooligosaccharide--protein glycosyltransferase subunit DAD1 OS=Drosophila melanogaster OX=7227 GN=Dad1 PE=1 SV=1	3.97E-57	72.321	100
P_KWMTBOMO12285	tr|Q93137|Q93137_BOMMO	Reverse transcriptase OS=Bombyx mori OX=7091 PE=4 SV=1	0	71.082	49.23913043	sp|O17449|TBB1_MANSE	Tubulin beta-1 chain OS=Manduca sexta OX=7130 PE=2 SV=1	0	99.769	23.47826087
P_KWMTBOMO12448	tr|H9IYM5|H9IYM5_BOMMO	Secretory carrier-associated membrane protein OS=Bombyx mori OX=7091 PE=3 SV=1	1.69E-99	87.5	73.01587302	sp|Q8K021|SCAM1_MOUSE	Secretory carrier-associated membrane protein 1 OS=Mus musculus OX=10090 GN=Scamp1 PE=1 SV=1	8.88E-77	52.838	90.87301587
P_KWMTBOMO12560	tr|A0A1Q1NKL1|A0A1Q1NKL1_BOMMO	Tyrosine--tRNA ligase OS=Bombyx mori OX=7091 PE=2 SV=1	0	99.809	100	sp|Q5ZJ08|SYYC_CHICK	Tyrosine--tRNA ligase, cytoplasmic OS=Gallus gallus OX=9031 GN=YARS1 PE=2 SV=1	0	71.374	100
P_KWMTBOMO12602	tr|H9IWB5|H9IWB5_BOMMO	Uncharacterized protein OS=Bombyx mori OX=7091 PE=3 SV=1	0	95.142	67.58824981	sp|P37276|DYHC_DROME	Dynein heavy chain, cytoplasmic OS=Drosophila melanogaster OX=7227 GN=Dhc64C PE=2 SV=2	0	79.96	99.77783263
P_KWMTBOMO12628	tr|H9IWC7|H9IWC7_BOMMO	Isoleucyl-tRNA synthetase OS=Bombyx mori OX=7091 GN=101742308 PE=3 SV=1	0	99.835	100	sp|Q8BU30|SYIC_MOUSE	Isoleucine--tRNA ligase, cytoplasmic OS=Mus musculus OX=10090 GN=Iars1 PE=1 SV=2	0	60.246	100
P_KWMTBOMO12706	tr|Q5UAQ2|Q5UAQ2_BOMMO	60S ribosomal protein L36 OS=Bombyx mori OX=7091 GN=RpL36 PE=2 SV=1	1.02E-81	100	100	sp|P49630|RL36_DROME	60S ribosomal protein L36 OS=Drosophila melanogaster OX=7227 GN=RpL36 PE=1 SV=1	4.53E-50	71.304	96.63865546
P_KWMTBOMO12878	tr|Q0KIX8|Q0KIX8_BOMMO	Glutamate synthase OS=Bombyx mori OX=7091 GN=BmGOGAT PE=2 SV=1	0	99.658	100	sp|Q18164|DPYD_CAEEL	Dihydropyrimidine dehydrogenase [NADP(+)] OS=Caenorhabditis elegans OX=6239 GN=dpyd-1 PE=3 SV=2	2.57E-20	29.206	15.39589443
P_KWMTBOMO13041	tr|Q8MUB0|Q8MUB0_BOMMO	Dihydrolipoyl dehydrogenase OS=Bombyx mori OX=7091 GN=692608 PE=2 SV=1	0	100	100	sp|O18480|DLDH_MANSE	Dihydrolipoyl dehydrogenase OS=Manduca sexta OX=7130 PE=2 SV=1	0	89.579	100
P_KWMTBOMO13226	tr|H6VTQ9|H6VTQ9_BOMMO	DnaJ-19 OS=Bombyx mori OX=7091 PE=2 SV=1	0	100	99.29775281	sp|Q9UGP8|SEC63_HUMAN	Translocation protein SEC63 homolog OS=Homo sapiens OX=9606 GN=SEC63 PE=1 SV=2	0	44.537	100
P_KWMTBOMO13722	tr|H9JPQ0|H9JPQ0_BOMMO	Uncharacterized protein OS=Bombyx mori OX=7091 PE=3 SV=1	2.24E-39	100	73.56321839	sp|Q99417|MYCBP_HUMAN	c-Myc-binding protein OS=Homo sapiens OX=9606 GN=MYCBP PE=1 SV=3	2.31E-23	56.944	82.75862069
P_KWMTBOMO13723	tr|Q1HPX3|Q1HPX3_BOMMO	ATP synthase subunit O, mitochondrial OS=Bombyx mori OX=7091 PE=2 SV=1	1.17E-152	100	100	sp|Q24439|ATPO_DROME	ATP synthase subunit O, mitochondrial OS=Drosophila melanogaster OX=7227 GN=ATPsynO PE=2 SV=2	7.56E-89	61.692	96.1722488
P_KWMTBOMO13774	tr|Q2F5W7|Q2F5W7_BOMMO	Ras-related GTP-binding protein 4b OS=Bombyx mori OX=7091 GN=692901 PE=2 SV=1	6.54E-160	100	100	sp|Q68EK7|RAB4B_DANRE	Ras-related protein Rab-4B OS=Danio rerio OX=7955 GN=rab4b PE=2 SV=1	5.07E-117	74.648	99.53271028
P_KWMTBOMO13859	tr|H9JPJ1|H9JPJ1_BOMMO	40S ribosomal protein S30 OS=Bombyx mori OX=7091 PE=3 SV=1	5.15E-93	100	100	sp|Q9W6Y0|RS30_ORYLA	40S ribosomal protein S30 OS=Oryzias latipes OX=8090 GN=fau PE=3 SV=2	1.58E-23	72.881	45.38461538
P_KWMTBOMO14041	tr|Q2F5J1|Q2F5J1_BOMMO	26S proteasome non-ATPase regulatory subunit 13 OS=Bombyx mori OX=7091 PE=2 SV=1	0	100	100	sp|B0BN93|PSD13_RAT	26S proteasome non-ATPase regulatory subunit 13 OS=Rattus norvegicus OX=10116 GN=Psmd13 PE=1 SV=1	1.26E-102	46.921	88.57142857
P_KWMTBOMO14157	tr|H9JNS6|H9JNS6_BOMMO	Coatomer subunit gamma OS=Bombyx mori OX=7091 GN=692402 PE=3 SV=1	0	99.768	100	sp|Q29AE5|COPG_DROPS	Coatomer subunit gamma OS=Drosophila pseudoobscura pseudoobscura OX=46245 GN=gammaCop PE=3 SV=1	0	58.75	100
P_KWMTBOMO14240	tr|H9JNK0|H9JNK0_BOMMO	Uncharacterized protein OS=Bombyx mori OX=7091 PE=4 SV=1	0	100	74.05159332	sp|Q9VJZ7|RRP1L_DROME	Ribosomal RNA processing protein 1 homolog OS=Drosophila melanogaster OX=7227 GN=Nnp-1 PE=1 SV=1	2.18E-72	29.222	100
P_KWMTBOMO14478	tr|H9IS48|H9IS48_BOMMO	Uncharacterized protein OS=Bombyx mori OX=7091 GN=101742289 PE=3 SV=1	1.04E-66	100	100	sp|Q9W1V3|FBRL_DROME	rRNA 2'-O-methyltransferase fibrillarin OS=Drosophila melanogaster OX=7227 GN=Fib PE=2 SV=1	4.31E-61	94.737	100
P_KWMTBOMO14530	tr|Q2F5R7|Q2F5R7_BOMMO	Eukaryotic peptide chain release factor subunit 1 OS=Bombyx mori OX=7091 PE=2 SV=1	0	99.314	100	sp|Q9VPH7|ERF1_DROME	Eukaryotic peptide chain release factor subunit 1 OS=Drosophila melanogaster OX=7227 GN=eRF1 PE=1 SV=2	0	93.822	100
P_KWMTBOMO14639	tr|Q5UAQ6|Q5UAQ6_BOMMO	60S ribosomal protein L32 OS=Bombyx mori OX=7091 GN=RpL32 PE=2 SV=1	1.84E-97	100	100	sp|Q962T1|RL32_SPOFR	60S ribosomal protein L32 OS=Spodoptera frugiperda OX=7108 GN=RpL32 PE=2 SV=1	6.75E-95	97.761	100

P_KWMTBOMO14715	tr|Q1HPL2|Q1HPL2_BOMMO	Phosphate carrier 1 OS=Bombyx mori OX=7091 GN=PiC PE=2 SV=1	0	100	100	sp|Q61703|MPCP_CHOFU	Phosphate carrier protein, mitochondrial OS=Choristoneura fumiferana OX=7141 PE=2 SV=1	0	78.771	100
P_KWMTBOMO14936	tr|H9IU37|H9IU37_BOMMO	GB1/RHD3-type G domain-containing protein OS=Bombyx mori OX=7091 PE=3 SV=1	0	97.695	100	sp|Q9VC57|ATLAS_DROME	Atlastin OS=Drosophila melanogaster OX=7227 GN=atl PE=1 SV=1	0	68.275	97.82214156
P_KWMTBOMO14942	tr|H9IU76|H9IU76_BOMMO	AMP deaminase OS=Bombyx mori OX=7091 PE=3 SV=1	0	98.229	97.60638298	sp|Q02356|AMPD2_RAT	AMP deaminase 2 OS=Rattus norvegicus OX=10116 GN=Ampd2 PE=1 SV=2	0	59.916	95.21276596
P_KWMTBOMO14962	tr|H9IU27|H9IU27_BOMMO	Uncharacterized protein OS=Bombyx mori OX=7091 PE=4 SV=1	0	100	99.40652819	sp|Q14690|RRP5_HUMAN	Protein RRP5 homolog OS=Homo sapiens OX=9606 GN=PDCD11 PE=1 SV=3	2.60E-77	28.588	86.84470821
P_KWMTBOMO15101	tr|H9J5V8|H9J5V8_BOMMO	Dolichyl-diphosphooligosaccharide--protein glycotransferase OS=Bombyx mori OX=7091 PE=3 SV=1	0	95.652	98.69960988	sp|Q3TDQ1|STT3B_MOUSE	Dolichyl-diphosphooligosaccharide--protein glycosyltransferase subunit STT3B OS=Mus musculus OX=10090 GN=Stt3b PE=1 SV=2	0	71.108	98.56957087
P_KWMTBOMO15121	tr|Q0N2R8|Q0N2R8_BOMMO	Signal recognition particle 54 kDa protein OS=Bombyx mori OX=7091 GN=SRP54 PE=2 SV=1	0	99.601	100	sp|Q7ZVN5|SRP54_DANRE	Signal recognition particle 54 kDa protein OS=Danio rerio OX=7955 GN=srp54 PE=2 SV=1	0	80.2	99.8003992
P_KWMTBOMO15547	tr|H9JMI6|H9JMI6_BOMMO	Nop domain-containing protein OS=Bombyx mori OX=7091 PE=3 SV=1	0	91.939	99.35064935	sp|O00567|NOP56_HUMAN	Nucleolar protein 56 OS=Homo sapiens OX=9606 GN=NOP56 PE=1 SV=4	0	66.59	93.93939394
P_KWMTBOMO15584	tr|D2Y4R2|D2Y4R2_BOMMO	Coatomer subunit alpha OS=Bombyx mori OX=7091 GN=COPA PE=2 SV=1	0	99.729	99.72972973	sp|P53621|COPA_HUMAN	Coatomer subunit alpha OS=Homo sapiens OX=9606 GN=COPA PE=1 SV=2	0	78.729	97.83783784
P_KWMTBOMO15665	tr|H9IYF4|H9IYF4_BOMMO	Uncharacterized protein OS=Bombyx mori OX=7091 GN=101736745 PE=4 SV=1	0	100	100	sp|P26305|LPSBP_PERAM	Hemolymph lipopolysaccharide-binding protein OS=Periplaneta americana OX=6978 PE=1 SV=1	3.37E-26	39.716	45.19230769
P_KWMTBOMO16130	tr|H9J4U4|H9J4U4_BOMMO	Calcium-transporting ATPase OS=Bombyx mori OX=7091 PE=3 SV=1	0	100	66.44385027	sp|Q16720|AT2B3_HUMAN	Plasma membrane calcium-transporting ATPase 3 OS=Homo sapiens OX=9606 GN=ATP2B3 PE=1 SV=3	0	62.683	100
P_KWMTBOMO16204	tr|H9JWQ7|H9JWQ7_BOMMO	CHK domain-containing protein OS=Bombyx mori OX=7091 GN=101744771 PE=4 SV=1	0	100	100	--	--	--	--	--
P_KWMTBOMO16309	tr|H9JW60|H9JW60_BOMMO	UBX domain-containing protein OS=Bombyx mori OX=7091 PE=4 SV=1	1.01E-106	92.353	43.58974359	sp|Q3SZC4|NSF1C_BOVIN	NSFL1 cofactor p47 OS=Bos taurus OX=9913 GN=NSFL1C PE=2 SV=1	6.48E-85	40.852	100
P_KWMTBOMO16319	tr|Q2F5K7|Q2F5K7_BOMMO	Receptor expression-enhancing protein OS=Bombyx mori OX=7091 PE=2 SV=1	3.16E-130	99.429	100	sp|Q29RM3|REEP5_BOVIN	Receptor expression-enhancing protein 5 OS=Bos taurus OX=9913 GN=REEP5 PE=2 SV=1	3.07E-59	50.595	96
P_KWMTBOMO16481	tr|Q19KB8|Q19KB8_BOMMO	Ras-related GTP-binding protein Rab11 OS=Bombyx mori OX=7091 GN=733063 PE=2 SV=1	1.33E-160	100	100	sp|P62492|RB11A_MOUSE	Ras-related protein Rab-11A OS=Mus musculus OX=10090 GN=Rab11a PE=1 SV=3	7.02E-129	82.326	100
P_KWMTBOMO16600	tr|H9J1F3|H9J1F3_BOMMO	acidPPc domain-containing protein OS=Bombyx mori OX=7091 PE=4 SV=1	1.70E-144	99.502	93.05555556	sp|Q66H88|PLPP6_RAT	Phospholipid phosphatase 6 OS=Rattus norvegicus OX=10116 GN=Plpp6 PE=2 SV=1	1.53E-18	35.754	82.87037037

unique PIPs of P25 at M4

Name	Bombyx-mori	Subject ID	Description	e-value	identity(%)	coverage(%)	Metazoa	Subject ID	Description	e-value	identity(%)	coverage(%)
P_KWMTBOMO00437		tr|H9IU13|H9IU13_BOMMO	PHB domain-containing protein OS=Bombyx mori OX=7091 PE=3 SV=1	0	100	98.82629108		sp|O61491|FLOT1_DROME	Flotillin-1 OS=Drosophila melanogaster OX=7227 GN=Flo1 PE=2 SV=1	0	83.059	99.76525822
P_KWMTBOMO00574		tr|H9JRT0|H9JRT0_BOMMO	Uncharacterized protein OS=Bombyx mori OX=7091 PE=4 SV=1	0	64.645	80.95238095		sp|P98160|PGBM_HUMAN	Basement membrane-specific heparan sulfate proteoglycan core protein OS=Homo sapiens OX=9606 GN=HSPG2 PE=1 SV=4	6.49E-141	26.794	84.83063328
P_KWMTBOMO00687		tr|H9J134|H9J134_BOMMO	Nucleolar GTP-binding protein 1 OS=Bombyx mori OX=7091 PE=4 SV=1	0	93.029	64.59627329		sp|Q9V411|NOG1_DROME	Nucleolar GTP-binding protein 1 OS=Drosophila melanogaster OX=7227 GN=Non1 PE=2 SV=1	0	68.666	100
P_KWMTBOMO00786		tr|H9JJK3|H9JJK3_BOMMO	HIT domain-containing protein OS=Bombyx mori OX=7091 GN=693069 PE=4 SV=1	6.16E-93	100	100		sp|P62958|HINT1_BOVIN	Histidine triad nucleotide-binding protein 1 OS=Bos taurus OX=9913 GN=HINT1 PE=1 SV=2	1.81E-62	68.75	100
P_KWMTBOMO00834		tr|H9JJI4|H9JJI4_BOMMO	RRM domain-containing protein OS=Bombyx mori OX=7091 GN=101746268 PE=4 SV=1	0	100	100		--	--	--	--	--
P_KWMTBOMO00881		tr|D4QF47|D4QF47_BOMMO	Ced-6 protein OS=Bombyx mori OX=7091 GN=ced-6 PE=2 SV=1	0	100	100		sp|Q7JUY7|CED6_DROME	PTB domain-containing adapter protein ced-6 OS=Drosophila melanogaster OX=7227 GN=ced-6 PE=1 SV=1	3.82E-84	39.463	94.34697856
P_KWMTBOMO01790		tr|H9J907|H9J907_BOMMO	TPR_REGION domain-containing protein OS=Bombyx mori OX=7091 PE=4 SV=1	0	99.758	78.05108798		sp|Q9VGU5|TTC14_DROME	Tetratricopeptide repeat protein 14 homolog OS=Drosophila melanogaster OX=7227 GN=CG6621 PE=1 SV=2	1.20E-82	48.657	31.69347209
P_KWMTBOMO01817		tr|H9IVQ5|H9IVQ5_BOMMO	RNA helicase OS=Bombyx mori OX=7091 PE=3 SV=1	0	100	50.33200531		sp|Q501J6|DDX17_MOUSE	Probable ATP-dependent RNA helicase DDX17 OS=Mus musculus OX=10090 GN=Ddx17 PE=1 SV=1	0	61.331	73.83798141
P_KWMTBOMO02059		tr|Q5UAM4|Q5UAM4_BOMMO	40S ribosomal protein S18 OS=Bombyx mori OX=7091 GN=RpS18 PE=2 SV=1	1.29E-111	100	100		sp|Q962R1|RS18_SPOFR	40S ribosomal protein S18 OS=Spodoptera frugiperda OX=7108 GN=RpS18 PE=2 SV=1	1.23E-110	99.342	100
P_KWMTBOMO02211		tr|E3VQ36|E3VQ36_BOMMO	Non-specific serine/threonine protein kinase OS=Bombyx mori OX=7091 GN=100529202 PE=2 SV=1	0	100	96.63496708		sp|M3TYT0|ROCK2_PIG	Rho-associated protein kinase 2 OS=Sus scrofa OX=9823 GN=ROCK2 PE=1 SV=1	0	44.876	99.92684711
P_KWMTBOMO02432		tr|H9JVD5|H9JVD5_BOMMO	Dolichyl-diphosphooligosaccharide--protein glycosyltransferase subunit 2 OS=Bombyx mori OX=7091 PE=3 SV=1	0	100	100		sp|Q3SZI6|RPN2_BOVIN	Dolichyl-diphosphooligosaccharide--protein glycosyltransferase subunit 2 OS=Bos taurus OX=9913 GN=RPN2 PE=2 SV=1	1.44E-58	28.155	99.35691318

P_KWMTBOMO02460	tr|H9IZ80|H9IZ80_BOMMO	Uncharacterized protein OS=Bombyx mori OX=7091 GN=101746215 PE=4 SV=1	0	100	100	--	--	--	--	--
P_KWMTBOMO02748	tr|H9J2I1|H9J2I1_BOMMO	TLC domain-containing protein OS=Bombyx mori OX=7091 GN=101745731 PE=3 SV=1	0	100	100	sp|Q6DED0|TR1L1_XENLA	Translocating chain-associated membrane protein 1-like 1 OS=Xenopus laevis OX=8355 GN=tram1l1 PE=2 SV=1	1.09E-74	35.979	100
P_KWMTBOMO02894	tr|H9J2B5|H9J2B5_BOMMO	Uncharacterized protein OS=Bombyx mori OX=7091 GN=101743323 PE=3 SV=1	0	98.864	98.05013928	sp|Q03427|LAMC_DROME	Lamin-C OS=Drosophila melanogaster OX=7227 GN=LamC PE=1 SV=2	2.44E-127	61.491	89.69359331
P_KWMTBOMO02994	tr|H9J281|H9J281_BOMMO	DNA photolyase OS=Bombyx mori OX=7091 PE=3 SV=1	0	100	96.04519774	sp|Q28811|PHR_POTTR	Deoxyribodipyrimidine photo-lyase OS=Potorous tridactylus OX=9310 GN=PHR PE=2 SV=1	0	59.205	90.01883239
P_KWMTBOMO03016	tr|H9J265|H9J265_BOMMO	Uncharacterized protein OS=Bombyx mori OX=7091 PE=4 SV=1	0	88.902	100	sp|Q6NV83|SR140_MOUSE	U2 snRNP-associated SURP motif-containing protein OS=Mus musculus OX=10090 GN=U2surp PE=1 SV=3	0	44.39	94.74299065
P_KWMTBOMO03146	tr|H9JVB0|H9JVB0_BOMMO	RRM domain-containing protein OS=Bombyx mori OX=7091 PE=4 SV=1	1.87E-131	98.404	53.71428571	sp|P31943|HNRH1_HUMAN	Heterogeneous nuclear ribonucleoprotein H OS=Homo sapiens OX=9606 GN=HNRNPH1 PE=1 SV=4	4.35E-92	43.733	100
P_KWMTBOMO03550	tr|H9J9U5|H9J9U5_BOMMO	Chromo domain-containing protein OS=Bombyx mori OX=7091 PE=4 SV=1	1.41E-72	82.09	51.14503817	sp|Q13185|CBX3_HUMAN	Chromobox protein homolog 3 OS=Homo sapiens OX=9606 GN=CBX3 PE=1 SV=4	2.25E-36	46.309	56.87022901
P_KWMTBOMO03748	tr|Q2F645|Q2F645_BOMMO	Transketolase OS=Bombyx mori OX=7091 PE=2 SV=1	0	100	100	sp|Q9D4D4|TKTL2_MOUSE	Transketolase-like protein 2 OS=Mus musculus OX=10090 GN=Tktl2 PE=1 SV=1	0	62.153	98.55305466
P_KWMTBOMO04413	tr|H9J7A3|H9J7A3_BOMMO	Uncharacterized protein OS=Bombyx mori OX=7091 PE=4 SV=1	0	99.719	93.93139842	sp|Q9WTX2|PRKRA_MOUSE	Interferon-inducible double-stranded RNA-dependent protein kinase activator A OS=Mus musculus OX=10090 GN=Prkra PE=1 SV=1	5.42E-56	36.774	81.79419525
P_KWMTBOMO04504	tr|H9J7F7|H9J7F7_BOMMO	Zinc-hook domain-containing protein OS=Bombyx mori OX=7091 PE=3 SV=1	2.04E-100	100	98	sp|Q9W252|RAD50_DROME	DNA repair protein RAD50 OS=Drosophila melanogaster OX=7227 GN=rad50 PE=2 SV=4	9.46E-37	43.791	100
P_KWMTBOMO04505	tr|H9J7F7|H9J7F7_BOMMO	Zinc-hook domain-containing protein OS=Bombyx mori OX=7091 PE=3 SV=1	0	99.3	85.54319932	sp|Q9W252|RAD50_DROME	DNA repair protein RAD50 OS=Drosophila melanogaster OX=7227 GN=rad50 PE=2 SV=4	1.59E-155	31.037	100
P_KWMTBOMO04742	tr|Q5UAR2|Q5UAR2_BOMMO	60S ribosomal protein L27 OS=Bombyx mori OX=7091 GN=RpL27 PE=2 SV=1	1.18E-95	100	100	sp|P61354|RL27_RAT	60S ribosomal protein L27 OS=Rattus norvegicus OX=10116 GN=Rpl27 PE=2 SV=2	2.09E-64	71.324	100
P_KWMTBOMO04769	tr|Q6ITV1|Q6ITV1_BOMMO	Transformer-2 protein A OS=Bombyx mori OX=7091 GN=TRA-2 PE=4 SV=2	0	99.648	100	sp|P62996|TRA2B_MOUSE	Transformer-2 protein homolog beta OS=Mus musculus OX=10090 GN=Tra2b PE=1 SV=1	3.65E-57	60.123	57.3943662
P_KWMTBOMO05013	tr|H9JF20|H9JF20_BOMMO	Uncharacterized protein OS=Bombyx mori OX=7091 PE=3 SV=1	0	98.512	93.82141346	sp|A1Z713|VPS13_DROME	Vacuolar protein sorting-associated protein 13 OS=Drosophila melanogaster OX=7227 GN=Vps13 PE=1 SV=2	0	37.121	100
P_KWMTBOMO05056	tr|Q2F5I7|Q2F5I7_BOMMO	DnaJ homolog subfamily A member 1 OS=Bombyx mori OX=7091 PE=2 SV=1	0	100	100	sp|P31689|DNJA1_HUMAN	DnaJ homolog subfamily A member 1 OS=Homo sapiens OX=9606 GN=DNAJA1 PE=1 SV=2	0	67.246	100
P_KWMTBOMO05673	tr|H9JBE6|H9JBE6_BOMMO	Tudor domain-containing protein OS=Bombyx mori OX=7091 GN=101736520 PE=4 SV=1	0	99.667	100	sp|O88884|AKAP1_RAT	A-kinase anchor protein 1, mitochondrial OS=Rattus norvegicus OX=10116 GN=Akap1 PE=1 SV=1	1.80E-64	39.329	54.75792988
P_KWMTBOMO05675	tr|Q5UAR5|Q5UAR5_BOMMO	Ribosomal protein L23A OS=Bombyx mori OX=7091 GN=RpL23A PE=2 SV=1	0	100	100	sp|P62752|RL23A_RAT	60S ribosomal protein L23a OS=Rattus norvegicus OX=10116 GN=Rpl23a PE=2 SV=1	8.65E-62	76.19	35.79545455
P_KWMTBOMO05688	tr|H9JBF6|H9JBF6_BOMMO	TOG domain-containing protein OS=Bombyx mori OX=7091 GN=101738436 PE=3 SV=1	0	100	100	sp|O57683|SF3B1_XENLA	Splicing factor 3B subunit 1 OS=Xenopus laevis OX=8355 GN=sf3b1 PE=2 SV=1	0	77.31	100
P_KWMTBOMO05728	tr|H9JBC1|H9JBC1_BOMMO	Aspartate carbamoyltransferase OS=Bombyx mori OX=7091 PE=3 SV=1	0	98.881	100	sp|P05990|PYR1_DROME	CAD protein OS=Drosophila melanogaster OX=7227 GN=r PE=1 SV=3	0	68.238	100
P_KWMTBOMO06056	tr|H9J011|H9J011_BOMMO	DUF1907 domain-containing protein OS=Bombyx mori OX=7091 GN=101745847 PE=4 SV=1	0	100	100	sp|Q91V76|CK054_MOUSE	Ester hydrolase C11orf54 homolog OS=Mus musculus OX=10090 PE=1 SV=1	1.39E-89	44.72	98.47094801
P_KWMTBOMO06229	tr|H9IX17|H9IX17_BOMMO	Ribos_L4_asso_C domain-containing protein OS=Bombyx mori OX=7091 GN=692659 PE=3 SV=1	0	100	100	sp|P09180|RL4_DROME	60S ribosomal protein L4 OS=Drosophila melanogaster OX=7227 GN=RpL4 PE=1 SV=2	0	68.407	88.24884793
P_KWMTBOMO06334	tr|D2WL76|D2WL76_BOMMO	Sericin 2 OS=Bombyx mori OX=7091 GN=Ser2 PE=4 SV=1	0	96.132	100	sp|Q25460|FP1_MYTED	Adhesive plaque matrix protein (Fragment) OS=Mytilus edulis OX=6550 GN=FP1 PE=1 SV=1	1.11E-56	36.364	41.02122777
P_KWMTBOMO06603	tr|H9JQ71|H9JQ71_BOMMO	Uncharacterized protein OS=Bombyx mori OX=7091 PE=4 SV=1	0	100	95.18201285	sp|Q6AYK9|CDYL_RAT	Chromodomain Y-like protein OS=Rattus norvegicus OX=10116 GN=Cdyl PE=1 SV=1	5.06E-19	25.287	27.94432548
P_KWMTBOMO06621	tr|U3U9V0|U3U9V0_BOMMO	Uncharacterized protein OS=Bombyx mori OX=7091 PE=2 SV=1	0	100	100	sp|Q61686|CBX5_MOUSE	Chromobox protein homolog 5 OS=Mus musculus OX=10090 GN=Cbx5 PE=1 SV=1	1.39E-17	35.882	61.5942029
P_KWMTBOMO06639	tr|Q2F5Q1|Q2F5Q1_BOMMO	Lysophospholipase OS=Bombyx mori OX=7091 PE=2 SV=1	6.85E-166	100	100	sp|Q3MHR0|LYPA1_BOVIN	Acyl-protein thioesterase 1 OS=Bos taurus OX=9913 GN=LYPLA1 PE=2 SV=1	6.45E-78	53.425	99.54545455
P_KWMTBOMO06731	tr|H9JQE1|H9JQE1_BOMMO	Histone deacetylase OS=Bombyx mori OX=7091 PE=3 SV=1	0	91.686	89.64803313	sp|Q94517|HDAC1_DROME	Histone deacetylase HDAC1 OS=Drosophila melanogaster OX=7227 GN=HDAC1 PE=1 SV=2	0	92.634	92.75362319
P_KWMTBOMO06766	tr|H9IU99|H9IU99_BOMMO	CPSF_A domain-containing protein OS=Bombyx mori OX=7091 GN=101738508 PE=4 SV=1	0	99.918	100	sp|Q921M3|SF3B3_MOUSE	Splicing factor 3B subunit 3 OS=Mus musculus OX=10090 GN=Sf3b3 PE=1 SV=1	0	76.346	100
P_KWMTBOMO06787	tr|H9JRF6|H9JRF6_BOMMO	SUMO-activating enzyme subunit OS=Bombyx mori OX=7091 PE=3 SV=1	0	96.875	100	sp|Q28GH3|SAE2_XENTR	SUMO-activating enzyme subunit 2 OS=Xenopus tropicalis OX=8364 GN=uba2 PE=2 SV=1	0	55	95.39473684
P_KWMTBOMO06803	tr|H9JR86|H9JR86_BOMMO	CP-type G domain-containing protein OS=Bombyx mori OX=7091 GN=101742911 PE=4 SV=1	0	100	100	sp|Q9W590|LSG1_DROME	Large subunit GTPase 1 homolog OS=Drosophila melanogaster OX=7227 GN=Ns3 PE=1 SV=1	5.32E-148	39.605	100
P_KWMTBOMO07076	tr|H9JLN4|H9JLN4_BOMMO	Uncharacterized protein OS=Bombyx mori OX=7091 PE=4 SV=1	0	99.76	80.03838772	sp|Q28EW0|TM87A_XENTR	Transmembrane protein 87A OS=Xenopus tropicalis OX=8364 GN=tmem87a PE=2 SV=1	5.77E-124	39.051	100

P_KWMTBOMO07331	tr|H9JMB7|H9JMB7_BOMMO	Uncharacterized protein OS=Bombyx mori OX=7091 PE=3 SV=1	0	99.752	100	sp|Q80W14|PR40B_MOUSE	Pre-mRNA-processing factor 40 homolog B OS=Mus musculus OX=10090 GN=Prpf40b PE=1 SV=2	6.02E-159	42.751	83.66336634
P_KWMTBOMO07380	--	--	--	--	--	--	--	--	--	--
P_KWMTBOMO07506	tr|H9J8Q6|H9J8Q6_BOMMO	BTB domain-containing protein OS=Bombyx mori OX=7091 PE=4 SV=1	0	100	90.33232628	sp|Q7KRI2|LOLAL_DROME	Longitudinals lacking protein-like OS=Drosophila melanogaster OX=7227 GN=lolal PE=1 SV=1	1.34E-20	34.862	32.9305136
P_KWMTBOMO07780	tr|H9IUH3|H9IUH3_BOMMO	Eukaryotic translation initiation factor 3 subunit M OS=Bombyx mori OX=7091 GN=101737255 PE=3 SV=1	0	100	100	sp|Q17D30|EIF3M_AEDAE	Eukaryotic translation initiation factor 3 subunit M OS=Aedes aegypti OX=7159 GN=AAEL004347 PE=3 SV=1	0	72.798	100
P_KWMTBOMO08022	tr|H9IV52|H9IV52_BOMMO	Dsp protein OS=Bombyx mori OX=7091 GN=dsp PE=2 SV=1	0	100	71.39423077	sp|Q24537|HMG2_DROME	High mobility group protein DSP1 OS=Drosophila melanogaster OX=7227 GN=Dsp1 PE=2 SV=1	1.07E-75	48.182	79.32692308
P_KWMTBOMO08044	tr|H9IV44|H9IV44_BOMMO	DNA-(apurinic or apyrimidinic site) endonuclease OS=Bombyx mori OX=7091 GN=101739740 PE=3 SV=1	0	100	100	sp|P27864|RRP1_DROME	Recombination repair protein 1 OS=Drosophila melanogaster OX=7227 GN=Rrp1 PE=1 SV=2	6.85E-131	44.487	83.2278481
P_KWMTBOMO08194	tr|H9JIX2|H9JIX2_BOMMO	Uncharacterized protein OS=Bombyx mori OX=7091 PE=3 SV=1	1.35E-72	66.522	52.15419501	sp|Q9VQ79|AIFM1_DROME	Putative apoptosis-inducing factor 1, mitochondrial OS=Drosophila melanogaster OX=7227 GN=AIF PE=2 SV=2	5.12E-60	35.423	72.33560091
P_KWMTBOMO08201	tr|H9JIW6|H9JIW6_BOMMO	Glucosamine-6-phosphate isomerase OS=Bombyx mori OX=7091 GN=101746460 PE=3 SV=1	0	98.12	98.51851852	sp|A4IHW6|GNPI2_XENTR	Glucosamine-6-phosphate isomerase 2 OS=Xenopus tropicalis OX=8364 GN=gnpda2 PE=2 SV=1	1.21E-159	75.281	98.88888889
P_KWMTBOMO08213	tr|H9JIW2|H9JIW2_BOMMO	Uncharacterized protein OS=Bombyx mori OX=7091 GN=101746972 PE=4 SV=1	0	100	100	sp|P51610|HCFC1_HUMAN	Host cell factor 1 OS=Homo sapiens OX=9606 GN=HCFC1 PE=1 SV=2	0	71.99	45.58472554
P_KWMTBOMO08404	tr|H9JI59|H9JI59_BOMMO	RNA cytidine acetyltransferase OS=Bombyx mori OX=7091 PE=3 SV=1	0	96.934	100	sp|Q9W3C1|NAT10_DROME	RNA cytidine acetyltransferase OS=Drosophila melanogaster OX=7227 GN=l(1)G0020 PE=1 SV=2	0	60.513	99.1861648
P_KWMTBOMO08509	tr|H9JIL2|H9JIL2_BOMMO	Coronin OS=Bombyx mori OX=7091 PE=3 SV=1	0	88.396	100	sp|Q0V8F1|CORO7_BOVIN	Coronin-7 OS=Bos taurus OX=9913 GN=CORO7 PE=2 SV=1	1.76E-106	43.373	35.40955631
P_KWMTBOMO08535	tr|B3GQU6|B3GQU6_BOMMO	3-hydroxyisobutyrate dehydrogenase OS=Bombyx mori OX=7091 GN=Hibadh PE=2 SV=1	5.84E-10	25.126	78.34645669	sp|Q7Q161|GLYR1_ANOGA	Putative oxidoreductase GLYR1 homolog OS=Anopheles gambiae OX=7165 GN=AGAP009949 PE=3 SV=5	2.16E-119	64.427	99.60629921
P_KWMTBOMO08652	tr|H9JDP0|H9JDP0_BOMMO	Diadenosine tetraphosphate synthetase OS=Bombyx mori OX=7091 PE=3 SV=1	0	100	100	sp|Q04451|SYG_BOMMO	Glycine--tRNA ligase OS=Bombyx mori OX=7091 PE=1 SV=2	0	99.118	100
P_KWMTBOMO08862	tr|H9JE97|H9JE97_BOMMO	SURF6 domain-containing protein OS=Bombyx mori OX=7091 PE=3 SV=1	8.16E-55	97.03	42.9787234	sp|Q9VDS6|SURF6_DROME	Surfeit locus protein 6 homolog OS=Drosophila melanogaster OX=7227 GN=Surf6 PE=1 SV=1	8.28E-14	43.011	39.57446809
P_KWMTBOMO08865	tr|H9JE95|H9JE95_BOMMO	Protein arginine N-methyltransferase OS=Bombyx mori OX=7091 PE=3 SV=1	0	99.674	91.2202381	sp|O14744|ANM5_HUMAN	Protein arginine N-methyltransferase 5 OS=Homo sapiens OX=9606 GN=PRMT5 PE=1 SV=4	7.08E-170	40.413	100
P_KWMTBOMO09000	tr|H9JE32|H9JE32_BOMMO	Uncharacterized protein OS=Bombyx mori OX=7091 PE=3 SV=1	2.27E-105	100	100	sp|P79382|MGST1_PIG	Microsomal glutathione S-transferase 1 OS=Sus scrofa OX=9823 GN=MGST1 PE=2 SV=3	3.61E-32	43.333	99.33774834
P_KWMTBOMO09385	tr|H9JT97|H9JT97_BOMMO	TLDc domain-containing protein OS=Bombyx mori OX=7091 PE=4 SV=1	6.24E-121	98.235	34.97942387	--	--	--	--	--
P_KWMTBOMO09591	tr|H9JTH6|H9JTH6_BOMMO	Uncharacterized protein OS=Bombyx mori OX=7091 PE=3 SV=1	9.34E-169	100	55.27522936	sp|O61492|FLOT2_DROME	Flotillin-2 OS=Drosophila melanogaster OX=7227 GN=Flo2 PE=2 SV=3	0	76.923	95.41284404
P_KWMTBOMO10056	tr|B5M9A0|B5M9A0_BOMMO	Myosin heavy chain (Fragment) OS=Bombyx mori OX=7091 PE=2 SV=1	0	55.179	26.95431472	sp|Q99323|MYSN_DROME	Myosin heavy chain, non-muscle OS=Drosophila melanogaster OX=7227 GN=zip PE=1 SV=2	0	74.987	100
P_KWMTBOMO10208	tr|H9J7V0|H9J7V0_BOMMO	Eukaryotic translation initiation factor 3 subunit D OS=Bombyx mori OX=7091 PE=3 SV=1	0	100	100	sp|Q0ZB77|EIF3D_BOMMO	Eukaryotic translation initiation factor 3 subunit D OS=Bombyx mori OX=7091 GN=eIF3-S7 PE=2 SV=1	0	99.636	100
P_KWMTBOMO10376	tr|H9JC16|H9JC16_BOMMO	Pept_C1 domain-containing protein OS=Bombyx mori OX=7091 PE=3 SV=1	0	100	100	sp|P00787|CATB_RAT	Cathepsin B OS=Rattus norvegicus OX=10116 GN=Ctsb PE=1 SV=2	8.05E-140	58.333	99.70326409
P_KWMTBOMO10493	tr|H9JC69|H9JC69_BOMMO	Eukaryotic translation initiation factor 3 subunit B OS=Bombyx mori OX=7091 PE=3 SV=1	0	99.568	100	sp|Q1HDZ5|EIF3B_BOMMO	Eukaryotic translation initiation factor 3 subunit B OS=Bombyx mori OX=7091 GN=eIF3-S9 PE=2 SV=1	0	99.568	100
P_KWMTBOMO11098	tr|H9JGC5|H9JGC5_BOMMO	RNA-binding motif protein 21 OS=Bombyx mori OX=7091 GN=101736670 PE=4 SV=1	0	100	100	sp|Q1JPD6|STPAP_BOVIN	Speckle targeted PIP5K1A-regulated poly(A) polymerase OS=Bos taurus OX=9913 GN=TUT1 PE=2 SV=1	3.32E-28	31.646	29.4044665
P_KWMTBOMO11160	tr|H9JGE9|H9JGE9_BOMMO	Uncharacterized protein OS=Bombyx mori OX=7091 PE=4 SV=1	0	96.247	100	sp|O15381|NVL_HUMAN	Nuclear valosin-containing protein-like OS=Homo sapiens OX=9606 GN=NVL PE=1 SV=1	0	41.724	100
P_KWMTBOMO11394	tr|H9IXH1|H9IXH1_BOMMO	KH domain-containing protein OS=Bombyx mori OX=7091 PE=4 SV=1	3.62E-180	94.928	71.31782946	sp|Q5ZIQ3|HNRPK_CHICK	Heterogeneous nuclear ribonucleoprotein K OS=Gallus gallus OX=9031 GN=HNRNPK PE=2 SV=1	3.38E-82	42.344	100
P_KWMTBOMO11517	tr|H9J3H3|H9J3H3_BOMMO	Uncharacterized protein OS=Bombyx mori OX=7091 PE=4 SV=1	0	96.387	98.84169884	--	--	--	--	--
P_KWMTBOMO11519	tr|H9J3H4|H9J3H4_BOMMO	Uncharacterized protein OS=Bombyx mori OX=7091 PE=3 SV=1	0	68.669	56.41431739	sp|Q86VQ3|TXND2_HUMAN	Thioredoxin domain-containing protein 2 OS=Homo sapiens OX=9606 GN=TXNDC2 PE=1 SV=4	2.10E-08	25.68	10.48463731
P_KWMTBOMO11731	sp|Q1HPK6|EF2_BOMMO	Translation elongation factor 2 OS=Bombyx mori OX=7091 GN=tef2 PE=1 SV=1	0	100	100	sp|Q1HPK6|EF2_BOMMO	Translation elongation factor 2 OS=Bombyx mori OX=7091 GN=tef2 PE=1 SV=1	0	100	100
P_KWMTBOMO12165	tr|H9JWZ8|H9JWZ8_BOMMO	Glutathione synthase OS=Bombyx mori OX=7091 PE=3 SV=1	1.95E-169	99.565	52.99539171	sp|P35668|GSHB_XENLA	Glutathione synthetase OS=Xenopus laevis OX=8355 GN=gss PE=2 SV=1	2.49E-108	43.564	93.0875576
P_KWMTBOMO12205	tr|H9JXB6|H9JXB6_BOMMO	Uncharacterized protein OS=Bombyx mori OX=7091 PE=3 SV=1	0	99.788	99.78858351	sp|Q5R1W7|ECHB_PANTR	Trifunctional enzyme subunit beta, mitochondrial OS=Pan troglodytes OX=9598 GN=HADHB PE=2 SV=1	0	66.667	96.40591966

P_KWMTBOMO12497		tr|Q5UAN8|Q5UAN8_BOMMO	40S ribosomal protein S6 OS=Bombyx mori OX=7091 GN=RpS6 PE=2 SV=1	0	99.605	100		sp|Q94624|RS6_MANSE	40S ribosomal protein S6 OS=Manduca sexta OX=7130 GN=RpS6 PE=2 SV=1	2.56E-178	96.443	100
P_KWMTBOMO12607		tr|H9IVY4|H9IVY4_BOMMO	Uncharacterized protein OS=Bombyx mori OX=7091 GN=101742698 PE=4 SV=1	0	100	57.16969317		sp|P0CG71|UBIQ1_CAEEL	Polyubiquitin-A OS=Caenorhabditis elegans OX=6239 GN=ubq-1 PE=3 SV=1	0	99.88	52.34815279
P_KWMTBOMO13405		tr|H9JN03|H9JN03_BOMMO	Proliferating cell nuclear antigen OS=Bombyx mori OX=7091 GN=692356 PE=3 SV=1	0	100	100		sp|O01377|PCNA_BOMMO	Proliferating cell nuclear antigen OS=Bombyx mori OX=7091 GN=PCNA PE=2 SV=1	0	98.846	100
P_KWMTBOMO13641		tr|H9JND7|H9JND7_BOMMO	SMC_N domain-containing protein OS=Bombyx mori OX=7091 PE=4 SV=1	4.13E-107	100	69.04761905		sp|O93309|SMC3_XENLA	Structural maintenance of chromosomes protein 3 OS=Xenopus laevis OX=8355 GN=smc3 PE=1 SV=2	5.93E-87	68.317	96.19047619
P_KWMTBOMO13997		tr|Q6T9Z7|Q6T9Z7_BOMMO	Fibroinase OS=Bombyx mori OX=7091 GN=693031 PE=2 SV=1	0	100	100		sp|Q26636|CATL_SARPE	Cathepsin L OS=Sarcophaga peregrina OX=7386 PE=1 SV=1	6.18E-177	68.902	96.18768328
P_KWMTBOMO14346		tr|H9JFA2|H9JFA2_BOMMO	Thioredoxin domain-containing protein OS=Bombyx mori OX=7091 PE=4 SV=1	0	100	84.31372549		sp|Q9H3N1|TMX1_HUMAN	Thioredoxin-related transmembrane protein 1 OS=Homo sapiens OX=9606 GN=TMX1 PE=1 SV=1	1.79E-63	47.802	59.47712418
P_KWMTBOMO14417		tr|D2Y4R5|D2Y4R5_BOMMO	Coatomer subunit delta OS=Bombyx mori OX=7091 GN=COPD PE=2 SV=1	1.83E-156	100	100		sp|P53619|COPD_BOVIN	Coatomer subunit delta OS=Bos taurus OX=9913 GN=ARCN1 PE=1 SV=1	4.75E-99	69.712	96.2962963
P_KWMTBOMO14483		tr|H9IS46|H9IS46_BOMMO	Uncharacterized protein OS=Bombyx mori OX=7091 PE=4 SV=1	0	99.603	91.30434783		sp|P25171|RCC1_DROME	Regulator of chromosome condensation OS=Drosophila melanogaster OX=7227 GN=Rcc1 PE=1 SV=2	7.43E-112	40.272	93.11594203
P_KWMTBOMO14568		tr|H9JH04|H9JH04_BOMMO	Importin N-terminal domain-containing protein OS=Bombyx mori OX=7091 PE=3 SV=1	4.68E-93	98.649	15.44885177		sp|Q5ZLT0|XPO7_CHICK	Exportin-7 OS=Gallus gallus OX=9031 GN=XPO7 PE=2 SV=1	0	67.216	100
P_KWMTBOMO14858		tr|H9JRJ9|H9JRJ9_BOMMO	Uncharacterized protein OS=Bombyx mori OX=7091 PE=4 SV=2	0	89.299	80.63814358		sp|Q02645|HTS_DROME	Protein hu-li tai shao OS=Drosophila melanogaster OX=7227 GN=hts PE=1 SV=2	0	56.932	49.16606236
P_KWMTBOMO15314		tr|A0A0K2S4Y7|A0A0K2S4Y7_BOMMO	Sodium/potassium-transporting ATPase subunit alpha OS=Bombyx mori OX=7091 PE=2 SV=1	0	99.901	100		sp|P13607|ATNA_DROME	Sodium/potassium-transporting ATPase subunit alpha OS=Drosophila melanogaster OX=7227 GN=Atpalpha PE=1 SV=3	0	92.254	99.80178394
P_KWMTBOMO15583		tr|D2Y4R2|D2Y4R2_BOMMO	Coatomer subunit alpha OS=Bombyx mori OX=7091 GN=COPA PE=2 SV=1	0	99.767	100		sp|Q27954|COPA_BOVIN	Coatomer subunit alpha OS=Bos taurus OX=9913 GN=COPA PE=1 SV=1	0	59.127	100
P_KWMTBOMO15617		tr|A5JPM0|A5JPM0_BOMMO	MLE protein OS=Bombyx mori OX=7091 PE=2 SV=1	0	100	100		sp|P24785|MLE_DROME	Dosage compensation regulator OS=Drosophila melanogaster OX=7227 GN=mle PE=1 SV=2	0	56.49	93.65443425
P_KWMTBOMO15867		tr|H9JV01|H9JV01_BOMMO	Uncharacterized protein OS=Bombyx mori OX=7091 GN=101745818 PE=3 SV=1	0	100	100		sp|P48758|CBR1_MOUSE	Carbonyl reductase [NADPH] 1 OS=Mus musculus OX=10090 GN=Cbr1 PE=1 SV=3	2.70E-81	46.739	100
P_KWMTBOMO16130		tr|H9J4U4|H9J4U4_BOMMO	Calcium-transporting ATPase OS=Bombyx mori OX=7091 PE=3 SV=1	0	100	66.44385027		sp|Q16720|AT2B3_HUMAN	Plasma membrane calcium-transporting ATPase 3 OS=Homo sapiens OX=9606 GN=ATP2B3 PE=1 SV=3	0	62.683	100
P_KWMTBOMO16524		tr|H9J7Z3|H9J7Z3_BOMMO	RRM domain-containing protein OS=Bombyx mori OX=7091 PE=3 SV=1	1.00E-90	76.064	100		sp|Q5R1W5|SRSF2_PANTR	Serine/arginine-rich splicing factor 2 OS=Pan troglodytes OX=9598 GN=SRSF2 PE=2 SV=3	1.89E-49	77.083	62.33766234
P_KWMTBOMO16530		tr|H9J7S2|H9J7S2_BOMMO	Ribosome biogenesis regulatory protein OS=Bombyx mori OX=7091 GN=101743869 PE=3 SV=1	0	99.716	100		sp|Q9CYH6|RRS1_MOUSE	Ribosome biogenesis regulatory protein homolog OS=Mus musculus OX=10090 GN=Rrs1 PE=1 SV=1	1.15E-85	46.726	95.45454545
P_KWMTBOMO16637		tr|Q9GSB6|Q9GSB6_BOMMO	Heat shock protein hsp20.4 OS=Bombyx mori OX=7091 GN=Hsp20.4 PE=2 SV=2	5.98E-133	100	100		sp|P82147|L2EFL_DROME	Protein lethal(2)essential for life OS=Drosophila melanogaster OX=7227 GN=l(2)efl PE=1 SV=1	8.44E-55	50.568	97.23756906

unique PIPs of P25 at L5D5

Name	Bombyx-mori	Subject ID	Description	e-value	identity(%)	coverage(%)	Metazoa	Subject ID	Description	e-value	identity(%)	coverage(%)
P_KWMTBOMO00106		tr|H9IXN0|H9IXN0_BOMMO	Uncharacterized protein OS=Bombyx mori OX=7091 PE=4 SV=1	0	92.621	66.19537275		sp|O95373|IPO7_HUMAN	Importin-7 OS=Homo sapiens OX=9606 GN=IPO7 PE=1 SV=1	0	50	100
P_KWMTBOMO00223		tr|B0M0V1|B0M0V1_BOMMO	Glycerol kinase OS=Bombyx mori OX=7091 GN=BmGK PE=2 SV=1	0	100	100		sp|Q63060|GLPK_RAT	Glycerol kinase OS=Rattus norvegicus OX=10116 GN=Gk PE=2 SV=1	3.17E-159	57.641	98.41688654
P_KWMTBOMO00254		sp|P82204|TPIS_BOMMO	Triosephosphate isomerase OS=Bombyx mori OX=7091 GN=Tpi PE=1 SV=2	0	100	100		sp|P82204|TPIS_BOMMO	Triosephosphate isomerase OS=Bombyx mori OX=7091 GN=Tpi PE=1 SV=2	0	100	100
P_KWMTBOMO00279		tr|H9ITU2|H9ITU2_BOMMO	Citrate synthase OS=Bombyx mori OX=7091 GN=101737646 PE=3 SV=1	0	100	100		sp|Q16P20|CISY2_AEDAE	Probable citrate synthase 2, mitochondrial OS=Aedes aegypti OX=7159 GN=AAEL011789 PE=3 SV=1	0	81.075	100
P_KWMTBOMO00303		tr|H9ITG0|H9ITG0_BOMMO	Rab-GAP TBC domain-containing protein OS=Bombyx mori OX=7091 PE=4 SV=1	0	98.655	99.87789988		sp|Q9VYY9|EVI5_DROME	Ecotropic viral integration site 5 ortholog OS=Drosophila melanogaster OX=7227 GN=Evi5 PE=1 SV=3	0	59.115	93.77289377
P_KWMTBOMO00362		tr|H9ITY0|H9ITY0_BOMMO	MSP domain-containing protein OS=Bombyx mori OX=7091 PE=3 SV=1	0	100	86.17021277		sp|Q16943|VP33_APLCA	Vesicle-associated membrane protein/synaptobrevin-binding protein OS=Aplysia californica OX=6500 PE=2 SV=1	1.32E-48	36.232	97.87234043
P_KWMTBOMO00390		tr|H9ITC4|H9ITC4_BOMMO	Uncharacterized protein OS=Bombyx mori OX=7091 PE=4 SV=1	0	100	79.5620438		--	--	--	--	--
P_KWMTBOMO00473		tr|Q1HPX8|Q1HPX8_BOMMO	6-phosphogluconate dehydrogenase, decarboxylating OS=Bombyx mori OX=7091 PE=2 SV=1	0	100	100		sp|P52209|6PGD_HUMAN	6-phosphogluconate dehydrogenase, decarboxylating OS=Homo sapiens OX=9606 GN=PGD PE=1 SV=3	0	73.542	99.37888199
P_KWMTBOMO00637		tr|H9J305|H9J305_BOMMO	Coatomer subunit beta' OS=Bombyx mori OX=7091 GN=100379187 PE=3 SV=1	2.13E-64	100	100		--	--	--	--	--

P_KWMTBOMO00722	tr|A0A0D6A6H2|A0A0D6A6H2_BOMMO	Methionine--tRNA ligase, cytoplasmic OS=Bombyx mori OX=7091 GN=BmMRS PE=2 SV=1	0	100	100	sp|Q6PF21|SYMC_XENLA	Methionine--tRNA ligase, cytoplasmic OS=Xenopus laevis OX=8355 GN=mars1 PE=2 SV=1	0	46.816	89.41058941
P_KWMTBOMO00793	tr|H9JJJ8|H9JJJ8_BOMMO	Uncharacterized protein OS=Bombyx mori OX=7091 GN=101743807 PE=3 SV=1	0	100	100	sp|Q68FQ0|TCPE_RAT	T-complex protein 1 subunit epsilon OS=Rattus norvegicus OX=10116 GN=Cct5 PE=1 SV=1	0	70.391	99.07749077
P_KWMTBOMO00837	tr|H9JJI3|H9JJI3_BOMMO	Proteasome subunit beta OS=Bombyx mori OX=7091 PE=3 SV=1	0	100	97.32824427	sp|P99026|PSB4_MOUSE	Proteasome subunit beta type-4 OS=Mus musculus OX=10090 GN=Psmb4 PE=1 SV=1	1.89E-88	49.799	95.03816794
P_KWMTBOMO00860	tr|D2Y4R7|D2Y4R7_BOMMO	Coatomer subunit zeta OS=Bombyx mori OX=7091 GN=COPZ PE=2 SV=1	1.18E-132	100	100	sp|P35604|COPZ1_BOVIN	Coatomer subunit zeta-1 OS=Bos taurus OX=9913 GN=COPZ1 PE=1 SV=2	2.88E-83	62.921	100
P_KWMTBOMO00973	--	--	--	--	--	--	--	--	--	--
P_KWMTBOMO00974	sp|Q6PUF8|RS21_BOMMO	40S ribosomal protein S21 OS=Bombyx mori OX=7091 GN=RpS21 PE=3 SV=1	2.43E-58	100	100	sp|Q962Q8|RS21_SPOFR	40S ribosomal protein S21 OS=Spodoptera frugiperda OX=7108 GN=RpS21 PE=3 SV=1	1.59E-57	100	100
P_KWMTBOMO00982	tr|H9IVS2|H9IVS2_BOMMO	Uncharacterized protein OS=Bombyx mori OX=7091 PE=4 SV=1	9.80E-180	100	48.71287129	sp|Q9D0F3|LMAN1_MOUSE	Protein ERGIC-53 OS=Mus musculus OX=10090 GN=Lman1 PE=1 SV=1	1.46E-138	44.353	96.43564356
P_KWMTBOMO01055	tr|Q5UAL7|Q5UAL7_BOMMO	40S ribosomal protein S26 OS=Bombyx mori OX=7091 GN=RpS26 PE=2 SV=1	1.02E-81	100	87.12121212	sp|Q9GT45|RS26_ANOGA	40S ribosomal protein S26 OS=Anopheles gambiae OX=7165 GN=RpS26 PE=3 SV=2	5.07E-67	84.348	87.12121212
P_KWMTBOMO01068	tr|H9JD15|H9JD15_BOMMO	60S ribosomal protein L34 OS=Bombyx mori OX=7091 PE=3 SV=1	1.19E-81	100	100	sp|P45842|RL34_AEDAL	60S ribosomal protein L34 OS=Aedes albopictus OX=7160 GN=RpL34 PE=2 SV=2	1.72E-56	74.074	90.75630252
P_KWMTBOMO01114	tr|H9JCT5|H9JCT5_BOMMO	26S proteasome regulatory subunit 7 OS=Bombyx mori OX=7091 GN=101744256 PE=3 SV=1	0	100	100	sp|P35998|PRS7_HUMAN	26S proteasome regulatory subunit 7 OS=Homo sapiens OX=9606 GN=PSMC2 PE=1 SV=3	0	91.014	100
P_KWMTBOMO01129	tr|H9JIS9|H9JIS9_BOMMO	F-actin-capping protein subunit beta OS=Bombyx mori OX=7091 PE=3 SV=1	0	100	100	sp|Q5XI32|CAPZB_RAT	F-actin-capping protein subunit beta OS=Rattus norvegicus OX=10116 GN=Capzb PE=1 SV=1	7.35E-168	81.481	98.18181818
P_KWMTBOMO01131	tr|Q4F863|Q4F863_BOMMO	Eukaryotic translation initiation factor 5A OS=Bombyx mori OX=7091 GN=eIF5A PE=2 SV=1	2.30E-118	100	100	sp|P62924|IF5A_SPOEX	Eukaryotic translation initiation factor 5A OS=Spodoptera exigua OX=7107 GN=eIF-5A PE=2 SV=1	1.61E-116	98.75	100
P_KWMTBOMO01148	tr|Q2F5M4|Q2F5M4_BOMMO	Receptor for activated protein kinase C RACK isoform 1 OS=Bombyx mori OX=7091 PE=2 SV=1	0	100	100	sp|O18640|GBLP_DROME	Guanine nucleotide-binding protein subunit beta-like protein OS=Drosophila melanogaster OX=7227 GN=Rack1 PE=1 SV=2	0	86.52	100
P_KWMTBOMO01182	tr|Q1EPM0|Q1EPM0_BOMMO	Glyceraldehyde-3-phosphate dehydrogenase OS=Bombyx mori OX=7091 GN=BmGAPDH PE=2 SV=1	0	100	100	sp|P07487|G3P2_DROME	Glyceraldehyde-3-phosphate dehydrogenase 2 OS=Drosophila melanogaster OX=7227 GN=Gapdh2 PE=1 SV=2	0	84.036	100
P_KWMTBOMO01300	tr|H9JH77|H9JH77_BOMMO	UBX domain-containing protein OS=Bombyx mori OX=7091 GN=101739090 PE=4 SV=1	0	100	100	sp|Q6GQ69|FAF2B_XENLA	FAS-associated factor 2-B OS=Xenopus laevis OX=8355 GN=faf2-b PE=2 SV=1	7.44E-108	40.858	100
P_KWMTBOMO01312	tr|Q2F5N7|Q2F5N7_BOMMO	26S proteasome regulatory subunit RPN11 OS=Bombyx mori OX=7091 PE=2 SV=1	0	100	100	sp|Q9V3H2|PSDE_DROME	26S proteasome non-ATPase regulatory subunit 14 OS=Drosophila melanogaster OX=7227 GN=Rpn11 PE=1 SV=1	0	92.926	100
P_KWMTBOMO01315	tr|Q1HPJ9|Q1HPJ9_BOMMO	V-type proton ATPase subunit H OS=Bombyx mori OX=7091 PE=2 SV=1	0	99.789	100	sp|Q9U5N0|VATH_MANSE	V-type proton ATPase subunit H OS=Manduca sexta OX=7130 PE=2 SV=1	0	96.421	100
P_KWMTBOMO01379	tr|H9JHI6|H9JHI6_BOMMO	2-oxoacid_dh domain-containing protein OS=Bombyx mori OX=7091 PE=4 SV=1	1.12E-103	99.306	49.14675768	sp|Q19749|ODP2_CAEEL	Dihydrolipoyllysine-residue acetyltransferase component of pyruvate dehydrogenase complex, mitochondrial OS=Caenorhabditis elegans OX=6239 GN=dlat-1 PE=1 SV=1	2.91E-85	50.593	86.34812287
P_KWMTBOMO01380	tr|H9JHI5|H9JHI5_BOMMO	Lipoyl-binding domain-containing protein OS=Bombyx mori OX=7091 PE=4 SV=1	5.01E-120	98.78	92.65536723	sp|Q19749|ODP2_CAEEL	Dihydrolipoyllysine-residue acetyltransferase component of pyruvate dehydrogenase complex, mitochondrial OS=Caenorhabditis elegans OX=6239 GN=dlat-1 PE=1 SV=1	2.61E-52	72.414	65.53672316
P_KWMTBOMO01423	tr|H9JHG0|H9JHG0_BOMMO	NADH-ubiquinone oxidoreductase 75 kDa subunit, mitochondrial OS=Bombyx mori OX=7091 PE=3 SV=1	0	98.518	100	sp|Q94511|NDUS1_DROME	NADH-ubiquinone oxidoreductase 75 kDa subunit, mitochondrial OS=Drosophila melanogaster OX=7227 GN=ND-75 PE=2 SV=3	0	64.393	100
P_KWMTBOMO01431	tr|H9JHF8|H9JHF8_BOMMO	Voltage-dependent anion-selective channel protein 3 OS=Bombyx mori OX=7091 GN=101739703 PE=3 SV=1	5.89E-131	100	100	sp|Q94920|VDAC_DROME	Voltage-dependent anion-selective channel OS=Drosophila melanogaster OX=7227 GN=porin PE=1 SV=3	8.71E-82	60.656	100
P_KWMTBOMO01579	tr|H9JH15|H9JH15_BOMMO	Uncharacterized protein OS=Bombyx mori OX=7091 PE=3 SV=1	2.14E-165	100	21.03939648	sp|Q5F3X8|SC31A_CHICK	Protein transport protein Sec31A OS=Gallus gallus OX=9031 GN=SEC31A PE=2 SV=1	0	32.3	100
P_KWMTBOMO01662	tr|Q19N36|Q19N36_BOMMO	Small GTP binding protein RAB5 OS=Bombyx mori OX=7091 GN=733058 PE=2 SV=1	1.91E-161	100	100	sp|P61021|RAB5B_MOUSE	Ras-related protein Rab-5B OS=Mus musculus OX=10090 GN=Rab5b PE=1 SV=1	1.06E-122	80.374	100
P_KWMTBOMO01757	sp|Q9GV27|EIF3H_BOMMO	Eukaryotic translation initiation factor 3 subunit H OS=Bombyx mori OX=7091 PE=2 SV=1	0	100	100	sp|Q9GV27|EIF3H_BOMMO	Eukaryotic translation initiation factor 3 subunit H OS=Bombyx mori OX=7091 PE=2 SV=1	0	100	100
P_KWMTBOMO01796	tr|Q2F5J2|Q2F5J2_BOMMO	Prohibitin OS=Bombyx mori OX=7091 GN=692987 PE=2 SV=1	0	100	100	sp|P24156|L2CC_DROME	Protein l(2)37Cc OS=Drosophila melanogaster OX=7227 GN=l(2)37Cc PE=2 SV=2	6.28E-169	83.088	99.27007299
P_KWMTBOMO01799	tr|Q1HPL3|Q1HPL3_BOMMO	Proteasome 26S non-ATPase subunit 7 OS=Bombyx mori OX=7091 GN=733131 PE=2 SV=1	0	100	100	sp|P26270|PSMD7_DROME	26S proteasome non-ATPase regulatory subunit 7 OS=Drosophila melanogaster OX=7227 GN=Rpn8 PE=1 SV=6	0	80.967	100
P_KWMTBOMO01802	tr|Q3HR36|Q3HR36_BOMMO	L-threonine dehydrogenase OS=Bombyx mori OX=7091 PE=2 SV=1	0	99.706	93.66391185	sp|Q2KIR8|TDH_BOVIN	L-threonine 3-dehydrogenase, mitochondrial OS=Bos taurus OX=9913 GN=TDH PE=2 SV=1	4.54E-138	60.062	88.98071625
P_KWMTBOMO01806	tr|H9J9G9|H9J9G9_BOMMO	Cation-transporting ATPase OS=Bombyx mori OX=7091 GN=101743411 PE=3 SV=1	0	99.914	100	sp|Q9EPE9|AT131_MOUSE	Manganese-transporting ATPase 13A1 OS=Mus musculus OX=10090 GN=Atp13a1 PE=1 SV=2	0	57.252	100
P_KWMTBOMO01821	tr|H9J9H8|H9J9H8_BOMMO	Uncharacterized protein OS=Bombyx mori OX=7091 GN=101744329 PE=4 SV=1	0	100	89.2635315	sp|Q5ZJP5|FND3A_CHICK	Fibronectin type-III domain-containing protein 3a OS=Gallus gallus OX=9031 GN=FNDC3A PE=2 SV=2	0	33.302	95.65217391

P_KWMTBOMO01941	tr|Q5UAQ4|Q5UAQ4_BOMMO	60S ribosomal protein L35 OS=Bombyx mori OX=7091 GN=RpL35 PE=2 SV=1	1.64E-84	100	100	sp|Q69CJ9|RL35_OPHHA	60S ribosomal protein L35 OS=Ophiophagus hannah OX=8665 GN=RPL35 PE=2 SV=3	1.41E-50	67.48	100
P_KWMTBOMO02013	tr|H9J125|H9J125_BOMMO	26S proteasome non-ATPase regulatory subunit 6 OS=Bombyx mori OX=7091 GN=101735567 PE=3 SV=1	0	100	100	sp|Q15008|PSMD6_HUMAN	26S proteasome non-ATPase regulatory subunit 6 OS=Homo sapiens OX=9606 GN=PSMD6 PE=1 SV=1	5.45E-102	43.112	100
P_KWMTBOMO02082	sp|Q68HB4|PROF_BOMMO	Profilin OS=Bombyx mori OX=7091 PE=2 SV=1	9.31E-92	100	100	sp|Q68HB4|PROF_BOMMO	Profilin OS=Bombyx mori OX=7091 PE=2 SV=1	6.07E-91	100	100
P_KWMTBOMO02088	tr|H9J0E7|H9J0E7_BOMMO	Lysine--tRNA ligase OS=Bombyx mori OX=7091 GN=101740883 PE=3 SV=1	0	100	100	sp|Q99MN1|SYK_MOUSE	Lysine--tRNA ligase OS=Mus musculus OX=10090 GN=Kars1 PE=1 SV=1	0	68.206	97.06896552
P_KWMTBOMO02278	sp|Q75VN3|TCTP_BOMMO	Translationally-controlled tumor protein homolog OS=Bombyx mori OX=7091 GN=Tctp PE=2 SV=1	8.85E-126	100	100	sp|Q75VN3|TCTP_BOMMO	Translationally-controlled tumor protein homolog OS=Bombyx mori OX=7091 GN=Tctp PE=2 SV=1	5.77E-125	100	100
P_KWMTBOMO02354	tr|H9JXZ9|H9JXZ9_BOMMO	Uncharacterized protein OS=Bombyx mori OX=7091 PE=4 SV=1	0	97.674	41.28	sp|P45953|ACADV_RAT	Very long-chain specific acyl-CoA dehydrogenase, mitochondrial OS=Rattus norvegicus OX=10116 GN=Acadvl PE=1 SV=1	0	56.689	95.68
P_KWMTBOMO02392	sp|B9VJ80|UBA5_BOMMO	Ubiquitin-like modifier-activating enzyme 5 OS=Bombyx mori OX=7091 PE=2 SV=1	0	99.237	100	sp|B9VJ80|UBA5_BOMMO	Ubiquitin-like modifier-activating enzyme 5 OS=Bombyx mori OX=7091 PE=2 SV=1	0	99.237	100
P_KWMTBOMO02468	tr|H9IZ84|H9IZ84_BOMMO	Uncharacterized protein OS=Bombyx mori OX=7091 PE=3 SV=1	0	99.523	98.82075472	sp|Q63081|PDIA6_RAT	Protein disulfide-isomerase A6 OS=Rattus norvegicus OX=10116 GN=Pdia6 PE=1 SV=2	0	59.155	100
P_KWMTBOMO02477	tr|Q5UAR9|Q5UAR9_BOMMO	Ribosomal protein L19 OS=Bombyx mori OX=7091 GN=RpL19 PE=2 SV=1	1.82E-145	100	100	sp|P36241|RL19_DROME	60S ribosomal protein L19 OS=Drosophila melanogaster OX=7227 GN=RpL19 PE=1 SV=2	4.16E-113	84.343	99
P_KWMTBOMO02500	tr|H9IZ97|H9IZ97_BOMMO	Uncharacterized protein OS=Bombyx mori OX=7091 PE=4 SV=1	0	90.773	100	sp|O75439|MPPB_HUMAN	Mitochondrial-processing peptidase subunit beta OS=Homo sapiens OX=9606 GN=PMPCB PE=1 SV=2	0	68	96.56652361
P_KWMTBOMO02535	tr|H9IZB1|H9IZB1_BOMMO	Uncharacterized protein OS=Bombyx mori OX=7091 PE=4 SV=1	0	96.875	70.12687428	sp|O08730|GLYG_RAT	Glycogenin-1 OS=Rattus norvegicus OX=10116 GN=Gyg1 PE=2 SV=4	3.47E-117	52.395	38.52364475
P_KWMTBOMO02542	tr|Q8T7L8|Q8T7L8_BOMMO	Seroin 1 OS=Bombyx mori OX=7091 PE=2 SV=1	7.09E-73	97.222	100	--	--	--	--	--
P_KWMTBOMO02577	tr|H9IZJ0|H9IZJ0_BOMMO	Uncharacterized protein OS=Bombyx mori OX=7091 PE=4 SV=1	0	100	99.85795455	sp|Q9W266|WDP_DROME	Protein windpipe OS=Drosophila melanogaster OX=7227 GN=wdp PE=1 SV=1	2.32E-16	32.8	35.51136364
P_KWMTBOMO02734	tr|Q9BPS1|Q9BPS1_BOMMO	Elongation factor 1 delta OS=Bombyx mori OX=7091 GN=ef-1d PE=2 SV=1	9.74E-145	100	100	sp|P32192|EF1D_ARTSA	Elongation factor 1-delta OS=Artemia salina OX=85549 PE=1 SV=2	5.90E-72	62.632	93.1372549
P_KWMTBOMO02775	tr|H9J1V6|H9J1V6_BOMMO	Very-long-chain enoyl-CoA reductase OS=Bombyx mori OX=7091 GN=101742899 PE=3 SV=1	0	100	100	sp|Q9N5Y2|TECR_CAEEL	Probable very-long-chain enoyl-CoA reductase art-1 OS=Caenorhabditis elegans OX=6239 GN=art-1 PE=3 SV=1	6.15E-119	56.209	100
P_KWMTBOMO02836	tr|H9J1Y2|H9J1Y2_BOMMO	Uncharacterized protein OS=Bombyx mori OX=7091 PE=3 SV=1	7.22E-124	90	100	sp|P55326|YZG1_CAEEL	Uncharacterized protein F13E6.1 OS=Caenorhabditis elegans OX=6239 GN=F13E6.1 PE=3 SV=2	1.51E-19	35.032	84.40860215
P_KWMTBOMO02840	sp|Q2F5R8|EIF3E_BOMMO	Eukaryotic translation initiation factor 3 subunit E OS=Bombyx mori OX=7091 GN=eIF3-S6 PE=2 SV=1	0	100	100	sp|Q2F5R8|EIF3E_BOMMO	Eukaryotic translation initiation factor 3 subunit E OS=Bombyx mori OX=7091 GN=eIF3-S6 PE=2 SV=1	0	100	100
P_KWMTBOMO02914	--	--	--	--	--	sp|P13804|ETFA_HUMAN	Electron transfer flavoprotein subunit alpha, mitochondrial OS=Homo sapiens OX=9606 GN=ETFA PE=1 SV=1	4.08E-159	69.136	98.18181818
P_KWMTBOMO03002	tr|Q587N3|Q587N3_BOMMO	Protein disulfide-isomerase OS=Bombyx mori OX=7091 GN=erp57 PE=2 SV=1	0	100	100	sp|Q8JG64|PDIA3_CHICK	Protein disulfide-isomerase A3 OS=Gallus gallus OX=9031 GN=PDIA3 PE=2 SV=1	4.56E-149	50.644	94.90835031
P_KWMTBOMO03003	tr|H9J272|H9J272_BOMMO	Adenylate kinase isoenzyme 6 homolog OS=Bombyx mori OX=7091 GN=101735934 PE=3 SV=1	2.60E-133	100	100	sp|Q7JYV7|KAD6_DROME	Adenylate kinase isoenzyme 6 homolog OS=Drosophila melanogaster OX=7227 GN=Ak6 PE=1 SV=1	3.13E-69	57.317	93.71428571
P_KWMTBOMO03027	tr|Q2F5M6|Q2F5M6_BOMMO	Ras small monomeric GTPase Rab6 OS=Bombyx mori OX=7091 PE=2 SV=1	1.13E-158	100	100	sp|O18334|RAB6_DROME	Ras-related protein Rab6 OS=Drosophila melanogaster OX=7227 GN=Rab6 PE=1 SV=1	2.75E-141	90.338	99.0430622
P_KWMTBOMO03092	tr|H9J8H0|H9J8H0_BOMMO	Arginine kinase OS=Bombyx mori OX=7091 PE=3 SV=1	0	100	98.44789357	sp|Q95PM9|KARG_PLOIN	Arginine kinase OS=Plodia interpunctella OX=58824 GN=ARGK PE=1 SV=1	0	92.394	78.71396896
P_KWMTBOMO03113	tr|Q2F5V9|Q2F5V9_BOMMO	Translocon-associated protein subunit beta OS=Bombyx mori OX=7091 PE=2 SV=1	1.01E-139	100	100	sp|P43308|SSRB_HUMAN	Translocon-associated protein subunit beta OS=Homo sapiens OX=9606 GN=SSR2 PE=1 SV=1	2.35E-60	52.809	93.68421053
P_KWMTBOMO03120	tr|H9J8F1|H9J8F1_BOMMO	Cytochrome c oxidase polypeptide Va OS=Bombyx mori OX=7091 PE=3 SV=1	6.12E-112	100	100	sp|Q94514|COX5A_DROME	Cytochrome c oxidase subunit 5A, mitochondrial OS=Drosophila melanogaster OX=7227 GN=COX5A PE=2 SV=2	2.89E-60	61.745	98.67549669
P_KWMTBOMO03169	tr|Q17201|Q17201_BOMMO	Bmsqd-2 OS=Bombyx mori OX=7091 PE=2 SV=1	0	99.34	100	sp|Q08473|SQD_DROME	RNA-binding protein squid OS=Drosophila melanogaster OX=7227 GN=sqd PE=1 SV=3	4.44E-100	72.683	67.65676568
P_KWMTBOMO03271	tr|H9JYD8|H9JYD8_BOMMO	Uncharacterized protein OS=Bombyx mori OX=7091 PE=3 SV=1	0	99.727	71.76470588	sp|Q9JLH7|CK5P3_RAT	CDK5 regulatory subunit-associated protein 3 OS=Rattus norvegicus OX=10116 GN=Cdk5rap3 PE=1 SV=1	5.35E-137	42.994	100
P_KWMTBOMO03301	tr|E0D4V7|E0D4V7_BOMMO	Phenylalanyl-tRNA synthetase beta subunit OS=Bombyx mori OX=7091 GN=FRSB PE=2 SV=1	0	100	100	sp|Q9VCA5|SYFB_DROME	Phenylalanine--tRNA ligase beta subunit OS=Drosophila melanogaster OX=7227 GN=beta-PheRS PE=1 SV=1	0	66.893	100
P_KWMTBOMO03311	tr|H9JAB8|H9JAB8_BOMMO	RRM domain-containing protein OS=Bombyx mori OX=7091 PE=4 SV=1	0	100	100	sp|P23588|IF4B_HUMAN	Eukaryotic translation initiation factor 4B OS=Homo sapiens OX=9606 GN=EIF4B PE=1 SV=2	2.09E-33	35.971	77.79850746
P_KWMTBOMO03337	tr|H9JA60|H9JA60_BOMMO	Uncharacterized protein OS=Bombyx mori OX=7091 PE=4 SV=1	0	98.697	96.84542587	sp|P21522|ROA1_SCHAM	Heterogeneous nuclear ribonucleoprotein A1, A2/B1 homolog OS=Schistocerca americana OX=7009 GN=HNRNP PE=2 SV=1	1.83E-91	74.857	55.20504732
P_KWMTBOMO03473	tr|L8B6C5|L8B6C5_BOMMO	Glucosidase 2 subunit beta OS=Bombyx mori OX=7091 PE=2 SV=1	0	100	100	sp|P14314|GLU2B_HUMAN	Glucosidase 2 subunit beta OS=Homo sapiens OX=9606 GN=PRKCSH PE=1 SV=2	7.96E-116	40.808	94.64627151

P_KWMTBOMO03656	tr|H9IY70|H9IY70_BOMMO	Uncharacterized protein OS=Bombyx mori OX=7091 PE=4 SV=1	3.38E-113	79.902	100	sp|P35293|RAB18_MOUSE	Ras-related protein Rab-18 OS=Mus musculus OX=10090 GN=Rab18 PE=1 SV=2	3.29E-73	54.082	96.07843137
P_KWMTBOMO03721	tr|H9JKE9|H9JKE9_BOMMO	TPR_REGION domain-containing protein OS=Bombyx mori OX=7091 PE=3 SV=2	0	97.586	100	sp|O43765|SGTA_HUMAN	Small glutamine-rich tetratricopeptide repeat-containing protein alpha OS=Homo sapiens OX=9606 GN=SGTA PE=1 SV=1	4.07E-71	42.997	100
P_KWMTBOMO03722	tr|H9JKE1|H9JKE1_BOMMO	Dynamin-type G domain-containing protein OS=Bombyx mori OX=7091 PE=4 SV=1	0	100	76.69172932	sp|Q9H4M9|EHD1_HUMAN	EH domain-containing protein 1 OS=Homo sapiens OX=9606 GN=EHD1 PE=1 SV=2	0	69.101	100
P_KWMTBOMO03951	tr|Q5UAL6|Q5UAL6_BOMMO	40S ribosomal protein S27 OS=Bombyx mori OX=7091 GN=RpS27 PE=2 SV=1	1.35E-59	100	100	sp|P55833|RS27_HOMAM	40S ribosomal protein S27 OS=Homarus americanus OX=6706 GN=RPS27 PE=3 SV=2	1.42E-47	78.571	100
P_KWMTBOMO03975	tr|Q1HPS9|Q1HPS9_BOMMO	Vacuolar ATP synthase subunit E OS=Bombyx mori OX=7091 PE=2 SV=1	2.68E-164	99.558	100	sp|P31402|VATE_MANSE	V-type proton ATPase subunit E OS=Manduca sexta OX=7130 GN=VHA26 PE=2 SV=1	3.29E-156	94.69	100
P_KWMTBOMO03986	tr|Q9NL60|Q9NL60_BOMMO	Annexin OS=Bombyx mori OX=7091 PE=2 SV=1	0	99.69	100	sp|P22464|ANXB9_DROME	Annexin B9 OS=Drosophila melanogaster OX=7227 GN=AnxB9 PE=2 SV=2	9.67E-180	76.012	99.38080495
P_KWMTBOMO04003	tr|H9JTB8|H9JTB8_BOMMO	Succinate--CoA ligase [ADP/GDP-forming] subunit alpha, mitochondrial OS=Bombyx mori OX=7091 GN=101735810 PE=3 SV=1	0	100	100	sp|Q94522|SUCA_DROME	Succinate--CoA ligase [ADP/GDP-forming] subunit alpha, mitochondrial OS=Drosophila melanogaster OX=7227 GN=Scsalpha1 PE=2 SV=3	1.01E-166	78.248	100
P_KWMTBOMO04051	tr|H9JL88|H9JL88_BOMMO	UDPGT domain-containing protein OS=Bombyx mori OX=7091 PE=3 SV=1	0	95.709	99.9002991	sp|A0A291PQH4|UGT2_DACCO	UDP-glucosyltransferase 2 OS=Dactylopius coccus OX=765876 GN=UTG2 PE=1 SV=1	6.72E-74	31.836	51.04685942
P_KWMTBOMO04132	tr|H9JGQ9|H9JGQ9_BOMMO	WD_REPEATS_REGION domain-containing protein OS=Bombyx mori OX=7091 PE=4 SV=1	0	99.782	75.8677686	sp|Q9VU68|WDR1_DROME	Actin-interacting protein 1 OS=Drosophila melanogaster OX=7227 GN=flr PE=2 SV=1	0	66.998	99.66942149
P_KWMTBOMO04167	tr|Q5CCJ5|Q5CCJ5_BOMMO	Ras oncogene OS=Bombyx mori OX=7091 GN=Bras2 PE=2 SV=1	6.42E-151	100	100	sp|P04388|RAS2_DROME	Ras-like protein 2 OS=Drosophila melanogaster OX=7227 GN=Ras64B PE=1 SV=2	4.74E-100	78.01	95.5
P_KWMTBOMO04170	tr|H9JGS8|H9JGS8_BOMMO	UTP--glucose-1-phosphate uridylyltransferase OS=Bombyx mori OX=7091 PE=3 SV=1	0	100	97.85575049	sp|Q07130|UGPA_BOVIN	UTP--glucose-1-phosphate uridylyltransferase OS=Bos taurus OX=9913 GN=UGP2 PE=1 SV=2	0	65.737	97.85575049
P_KWMTBOMO04330	tr|H9JX71|H9JX71_BOMMO	PCI domain-containing protein OS=Bombyx mori OX=7091 GN=101735320 PE=3 SV=1	0	100	100	sp|Q7KLV9|PSD11_DROME	26S proteasome non-ATPase regulatory subunit 11 OS=Drosophila melanogaster OX=7227 GN=Rpn6 PE=1 SV=1	0	76.54	100
P_KWMTBOMO04424	tr|H9J7A0|H9J7A0_BOMMO	PA28_alpha domain-containing protein OS=Bombyx mori OX=7091 PE=4 SV=1	3.58E-90	99.225	52.43902439	sp|Q5F3J5|PSME3_CHICK	Proteasome activator complex subunit 3 OS=Gallus gallus OX=9031 GN=PSME3 PE=1 SV=1	5.95E-92	50.201	100
P_KWMTBOMO04427	tr|H9J7B7|H9J7B7_BOMMO	Signal recognition particle receptor subunit beta OS=Bombyx mori OX=7091 PE=3 SV=1	0	100	100	sp|Q9Y5M8|SRPRB_HUMAN	Signal recognition particle receptor subunit beta OS=Homo sapiens OX=9606 GN=SRPRB PE=1 SV=3	2.92E-59	46.575	87.6
P_KWMTBOMO04529	tr|Q2F6C7|Q2F6C7_BOMMO	ADP-ribosylation factor OS=Bombyx mori OX=7091 GN=778456 PE=2 SV=1	9.95E-136	100	100	sp|P61210|ARF1_LOCMI	ADP-ribosylation factor 1 OS=Locusta migratoria OX=7004 GN=ARF1 PE=2 SV=2	7.09E-134	98.901	100
P_KWMTBOMO04543	tr|H9J7H4|H9J7H4_BOMMO	AP-2 complex subunit alpha OS=Bombyx mori OX=7091 GN=101739513 PE=3 SV=1	0	100	100	sp|Q29N38|AP2A_DROPS	AP-2 complex subunit alpha OS=Drosophila pseudoobscura pseudoobscura OX=46245 GN=alpha-Adaptin PE=3 SV=1	0	82.696	100
P_KWMTBOMO04568	tr|H9J7I1|H9J7I1_BOMMO	Uncharacterized protein OS=Bombyx mori OX=7091 GN=100862833 PE=4 SV=1	0	100	100	sp|Q08D64|ABCB6_XENTR	ATP-binding cassette sub-family B member 6, mitochondrial OS=Xenopus tropicalis OX=8364 GN=abcb6 PE=2 SV=1	0	55.201	95.75033201
P_KWMTBOMO04584	tr|Q2F5K1|Q2F5K1_BOMMO	Signal sequence receptor subunit gamma OS=Bombyx mori OX=7091 PE=2 SV=1	1.07E-132	100	100	sp|Q9UNL2|SSRG_HUMAN	Translocon-associated protein subunit gamma OS=Homo sapiens OX=9606 GN=SSR3 PE=1 SV=1	8.00E-87	64.865	100
P_KWMTBOMO04674	tr|Q1HQ86|Q1HQ86_BOMMO	Mobility group protein 1B OS=Bombyx mori OX=7091 GN=100101161 PE=2 SV=1	3.17E-78	100	100	sp|Q06943|HMGZ_DROME	High mobility group protein Z OS=Drosophila melanogaster OX=7227 GN=HmgZ PE=1 SV=1	5.24E-33	66.234	64.70588235
P_KWMTBOMO04737	tr|H9JK23|H9JK23_BOMMO	Carboxylic ester hydrolase OS=Bombyx mori OX=7091 PE=3 SV=1	0	99.538	95.58498896	sp|B2D0J5|EST6_APIME	Venom carboxylesterase-6 OS=Apis mellifera OX=7460 PE=2 SV=1	1.76E-118	41.256	98.45474614
P_KWMTBOMO04740	tr|H9JK13|H9JK13_BOMMO	Endoplasmic reticulum transmembrane protein OS=Bombyx mori OX=7091 GN=101735495 PE=3 SV=1	8.35E-166	100	100	sp|Q5R8H3|BAP31_PONAB	B-cell receptor-associated protein 31 OS=Pongo abelii OX=9601 GN=BCAP31 PE=2 SV=3	1.47E-45	42.742	100
P_KWMTBOMO04757	tr|H9JK06|H9JK06_BOMMO	Uncharacterized protein OS=Bombyx mori OX=7091 PE=3 SV=1	0	95.892	89.63178295	sp|Q23979|MY61F_DROME	Unconventional myosin IC OS=Drosophila melanogaster OX=7227 GN=Myo61F PE=1 SV=3	0	60.155	100
P_KWMTBOMO04762	tr|H9JK34|H9JK34_BOMMO	RNA helicase OS=Bombyx mori OX=7091 PE=3 SV=1	0	95.957	100	sp|Q9CWX9|DDX47_MOUSE	Probable ATP-dependent RNA helicase DDX47 OS=Mus musculus OX=10090 GN=Ddx47 PE=2 SV=2	0	71.8	98.08510638
P_KWMTBOMO04768	tr|H9JK00|H9JK00_BOMMO	Valyl-tRNA synthetase OS=Bombyx mori OX=7091 PE=3 SV=1	0	93.973	100	sp|Q04462|SYVC_RAT	Valine--tRNA ligase OS=Rattus norvegicus OX=10116 GN=Vars1 PE=2 SV=2	0	59.277	100
P_KWMTBOMO04789	tr|Q2F5P7|Q2F5P7_BOMMO	Mitochondrial matrix protein p32 OS=Bombyx mori OX=7091 PE=2 SV=1	0	100	100	sp|Q3T0B6|C1QBP_BOVIN	Complement component 1 Q subcomponent-binding protein, mitochondrial OS=Bos taurus OX=9913 GN=C1QBP PE=2 SV=1	7.30E-40	32.042	100
P_KWMTBOMO04877	tr|H9JEV4|H9JEV4_BOMMO	Myosin motor domain-containing protein OS=Bombyx mori OX=7091 PE=3 SV=1	0	99.662	24.30213465	sp|Q01989|MYS9_DROME	Myosin heavy chain 95F OS=Drosophila melanogaster OX=7227 GN=jar PE=2 SV=4	0	58.481	100
P_KWMTBOMO04906	tr|H9JEW9|H9JEW9_BOMMO	Aminopeptidase N-8 OS=Bombyx mori OX=7091 GN=101737565 PE=2 SV=1	0	98.024	92.42009132	sp|P15145|AMPN_PIG	Aminopeptidase N OS=Sus scrofa OX=9823 GN=ANPEP PE=1 SV=4	9.30E-128	32.057	76.34703196
P_KWMTBOMO04992	tr|H9JEZ8|H9JEZ8_BOMMO	Uncharacterized protein OS=Bombyx mori OX=7091 PE=3 SV=1	0	99.614	100	sp|Q21929|DCXR_CAEEL	L-xylulose reductase OS=Caenorhabditis elegans OX=6239 GN=dhs-21 PE=1 SV=2	5.68E-38	33.597	97.68339768
P_KWMTBOMO05032	tr|Q1HQ41|Q1HQ41_BOMMO	Signal recognition particle 19 kDa protein OS=Bombyx mori OX=7091 PE=2 SV=1	6.19E-109	99.324	100	sp|P49963|SRP19_DROME	Signal recognition particle 19 kDa protein OS=Drosophila melanogaster OX=7227 GN=Srp19 PE=2 SV=2	1.63E-51	56.934	92.56756757

P_KWMTBOMO05178	tr|Q2F5M8|Q2F5M8_BOMMO	Phosphoserine aminotransferase OS=Bombyx mori OX=7091 PE=2 SV=1	0	100	100	sp|Q9VAN0|SERC_DROME	Probable phosphoserine aminotransferase OS=Drosophila melanogaster OX=7227 GN=CG11899 PE=2 SV=1	9.90E-147	64.935	96.85534591
P_KWMTBOMO05228	tr|Q5CCL3|Q5CCL3_BOMMO	Peptidyl-prolyl cis-trans isomerase OS=Bombyx mori OX=7091 PE=2 SV=1	7.28E-122	100	100	sp|P25007|PPIA_DROME	Peptidyl-prolyl cis-trans isomerase OS=Drosophila melanogaster OX=7227 GN=Cyp1 PE=1 SV=2	1.23E-98	79.755	98.78787879
P_KWMTBOMO05254	tr|H9IZ07|H9IZ07_BOMMO	RWD domain-containing protein OS=Bombyx mori OX=7091 GN=101745778 PE=4 SV=1	2.39E-176	100	100	sp|Q9CQK7|RWDD1_MOUSE	RWD domain-containing protein 1 OS=Mus musculus OX=10090 GN=Rwdd1 PE=1 SV=1	1.44E-57	50.826	100
P_KWMTBOMO05258	tr|H9IZ08|H9IZ08_BOMMO	Peptidase_M24 domain-containing protein OS=Bombyx mori OX=7091 GN=101746307 PE=3 SV=1	0	100	100	sp|Q6AYD3|PA2G4_RAT	Proliferation-associated protein 2G4 OS=Rattus norvegicus OX=10116 GN=Pa2g4 PE=1 SV=1	1.26E-149	57.778	94.98680739
P_KWMTBOMO05281	tr|H9IZ19|H9IZ19_BOMMO	Uncharacterized protein OS=Bombyx mori OX=7091 GN=101735890 PE=3 SV=1	4.73E-144	100	100	sp|Q9CQC9|SAR1B_MOUSE	GTP-binding protein SAR1b OS=Mus musculus OX=10090 GN=Sar1b PE=1 SV=1	9.93E-107	75	100
P_KWMTBOMO05295	tr|H9IZ23|H9IZ23_BOMMO	Pyruvate kinase OS=Bombyx mori OX=7091 PE=3 SV=1	0	99.132	51.2455516	sp|O62619|KPYK_DROME	Pyruvate kinase OS=Drosophila melanogaster OX=7227 GN=PyK PE=2 SV=2	0	72.659	47.5088968
P_KWMTBOMO05299	tr|H9IYR7|H9IYR7_BOMMO	Uncharacterized protein OS=Bombyx mori OX=7091 PE=3 SV=1	0	96.091	98.08306709	sp|O95749|GGPPS_HUMAN	Geranylgeranyl pyrophosphate synthase OS=Homo sapiens OX=9606 GN=GGPS1 PE=1 SV=1	5.33E-131	60.357	89.45686901
P_KWMTBOMO05321	tr|H9IYQ9|H9IYQ9_BOMMO	Uncharacterized protein OS=Bombyx mori OX=7091 PE=3 SV=1	0	99.85	97.65395894	sp|Q9VA73|CMC_DROME	Calcium-binding mitochondrial carrier protein Aralar1 OS=Drosophila melanogaster OX=7227 GN=aralar1 PE=2 SV=1	0	69.001	98.38709677
P_KWMTBOMO05357	tr|H9IZ54|H9IZ54_BOMMO	V-type proton ATPase subunit a OS=Bombyx mori OX=7091 PE=3 SV=1	0	93.548	77.6849642	sp|Q8AVM5|VPP1_XENLA	V-type proton ATPase 116 kDa subunit a1 OS=Xenopus laevis OX=8355 GN=atp6v0a1 PE=2 SV=1	0	59.645	100
P_KWMTBOMO05364	tr|H9IZ59|H9IZ59_BOMMO	Moesin/ezrin/radixin homolog 1 OS=Bombyx mori OX=7091 PE=4 SV=1	0	100	89.41798942	sp|B0WYY2|MOEH_CULQU	Moesin/ezrin/radixin homolog 1 OS=Culex quinquefasciatus OX=7176 GN=Moe PE=3 SV=1	0	84.441	100
P_KWMTBOMO05464	tr|H9JB09|H9JB09_BOMMO	Vasa intronic protein OS=Bombyx mori OX=7091 GN=101741313 PE=2 SV=1	0	100	100	sp|Q6AXS5|PAIRB_RAT	Plasminogen activator inhibitor 1 RNA-binding protein OS=Rattus norvegicus OX=10116 GN=Serbp1 PE=1 SV=2	1.23E-20	40.625	41.1311054
P_KWMTBOMO05509	tr|H9JB27|H9JB27_BOMMO	Uncharacterized protein OS=Bombyx mori OX=7091 PE=4 SV=1	0	100	89.41798942	sp|Q9CXW2|RT22_MOUSE	28S ribosomal protein S22, mitochondrial OS=Mus musculus OX=10090 GN=Mrps22 PE=1 SV=1	7.68E-52	34.783	79.1005291
P_KWMTBOMO05651	tr|H9JB94|H9JB94_BOMMO	Malectin domain-containing protein OS=Bombyx mori OX=7091 GN=101744143 PE=3 SV=1	0	99.621	100	sp|Q14165|MLEC_HUMAN	Malectin OS=Homo sapiens OX=9606 GN=MLEC PE=1 SV=1	8.20E-90	53.226	93.93939394
P_KWMTBOMO05719	tr|D2CZY1|D2CZY1_BOMMO	UV excision repair protein RAD23 OS=Bombyx mori OX=7091 GN=100328606 PE=2 SV=1	0	100	100	sp|P54725|RD23A_HUMAN	UV excision repair protein RAD23 homolog A OS=Homo sapiens OX=9606 GN=RAD23A PE=1 SV=1	1.40E-101	52.909	100
P_KWMTBOMO05784	tr|H9JBN7|H9JBN7_BOMMO	DUF1681 domain-containing protein OS=Bombyx mori OX=7091 PE=3 SV=1	3.74E-118	100	66.10878661	sp|Q9VXB0|NECAP_DROME	NECAP-like protein CG9132 OS=Drosophila melanogaster OX=7227 GN=CG9132 PE=2 SV=1	5.98E-101	59.216	100
P_KWMTBOMO05806	tr|H9JBP6|H9JBP6_BOMMO	Thioredoxin domain-containing protein OS=Bombyx mori OX=7091 PE=4 SV=1	0	100	93.97260274	sp|Q9D1Q6|ERP44_MOUSE	Endoplasmic reticulum resident protein 44 OS=Mus musculus OX=10090 GN=Erp44 PE=1 SV=1	3.26E-135	51.781	100
P_KWMTBOMO05810	tr|H9JBQ0|H9JBQ0_BOMMO	Uncharacterized protein OS=Bombyx mori OX=7091 PE=4 SV=1	0	99.381	50.35650624	sp|Q8SWU7|OLA1_DROME	Obg-like ATPase 1 OS=Drosophila melanogaster OX=7227 GN=CG1354 PE=1 SV=1	4.43E-180	78.274	14.97326203
P_KWMTBOMO05820	tr|Q2F681|Q2F681_BOMMO	Isocitrate dehydrogenase [NADP] OS=Bombyx mori OX=7091 PE=2 SV=1	0	100	100	sp|Q9Z2K9|IDHC_MICME	Isocitrate dehydrogenase [NADP] cytoplasmic OS=Microtus mexicanus OX=79689 GN=IDH1 PE=2 SV=1	0	77.451	100
P_KWMTBOMO05976	tr|H9J059|H9J059_BOMMO	Tr-type G domain-containing protein OS=Bombyx mori OX=7091 PE=4 SV=1	6.18E-175	93.103	95.25547445	sp|D2XV59|GTPB1_RAT	GTP-binding protein 1 OS=Rattus norvegicus OX=10116 GN=Gtpbp1 PE=1 SV=1	1.81E-93	64.317	82.84671533
P_KWMTBOMO05993	tr|H9IZY3|H9IZY3_BOMMO	Uncharacterized protein OS=Bombyx mori OX=7091 PE=3 SV=1	0	99.777	90.89068826	sp|P91938|TRXR1_DROME	Thioredoxin reductase 1, mitochondrial OS=Drosophila melanogaster OX=7227 GN=Trxr-1 PE=1 SV=2	0	67.677	100
P_KWMTBOMO06045	tr|H9J005|H9J005_BOMMO	TOG domain-containing protein OS=Bombyx mori OX=7091 PE=3 SV=2	0	93.348	100	sp|E9PVA8|GCN1_MOUSE	eIF-2-alpha kinase activator GCN1 OS=Mus musculus OX=10090 GN=Gcn1 PE=1 SV=1	0	45.751	100
P_KWMTBOMO06100	tr|H9IWU2|H9IWU2_BOMMO	Aconitate hydratase OS=Bombyx mori OX=7091 PE=4 SV=1	0	100	44.7309417	sp|P21399|ACOC_HUMAN	Cytoplasmic aconitate hydratase OS=Homo sapiens OX=9606 GN=ACO1 PE=1 SV=3	0	66.367	99.66367713
P_KWMTBOMO06227	tr|H9IWL2|H9IWL2_BOMMO	HTH La-type RNA-binding domain-containing protein OS=Bombyx mori OX=7091 PE=4 SV=1	0	90.363	59.54844779	sp|Q9VAW5|LARP_DROME	La-related protein 1 OS=Drosophila melanogaster OX=7227 GN=larp PE=1 SV=5	4.79E-144	59.113	38.19379116
P_KWMTBOMO06228	tr|H9IWL1|H9IWL1_BOMMO	LITAF domain-containing protein OS=Bombyx mori OX=7091 PE=3 SV=1	1.49E-80	99.153	65.55555556	--	--	--	--	--
P_KWMTBOMO06291	tr|H9IX45|H9IX45_BOMMO	C2H2-type domain-containing protein OS=Bombyx mori OX=7091 GN=101746228 PE=4 SV=1	0	100	100	--	--	--	--	--
P_KWMTBOMO06299	tr|H9IX47|H9IX47_BOMMO	RNA exonuclease 4 OS=Bombyx mori OX=7091 GN=101746969 PE=3 SV=1	6.14E-62	100	81.65137615	sp|Q6PAQ4|REXO4_MOUSE	RNA exonuclease 4 OS=Mus musculus OX=10090 GN=Rexo4 PE=2 SV=2	2.97E-07	35.526	69.72477064
P_KWMTBOMO06354	tr|H9JR23|H9JR23_BOMMO	Aminotran_1_2 domain-containing protein OS=Bombyx mori OX=7091 PE=4 SV=1	0	94.624	90.11627907	sp|Q28DB5|ALAT2_XENTR	Alanine aminotransferase 2 OS=Xenopus tropicalis OX=8364 GN=gpt2 PE=2 SV=1	0	58.078	94.76744186
P_KWMTBOMO06355	tr|H9JR22|H9JR22_BOMMO	Uncharacterized protein OS=Bombyx mori OX=7091 PE=4 SV=1	0	82.688	100	sp|P98158|LRP2_RAT	Low-density lipoprotein receptor-related protein 2 OS=Rattus norvegicus OX=10116 GN=Lrp2 PE=1 SV=1	0	41.512	100
P_KWMTBOMO06373	tr|Q2F5L8|Q2F5L8_BOMMO	Ribonucleoprotein OS=Bombyx mori OX=7091 PE=2 SV=1	8.53E-93	100	100	sp|Q5XH16|NH2L1_XENLA	NHP2-like protein 1 OS=Xenopus laevis OX=8355 GN=snu13 PE=2 SV=1	4.36E-77	81.89	97.69230769
P_KWMTBOMO06401	tr|Q1HPP3|Q1HPP3_BOMMO	ATP synthase subunit d, mitochondrial OS=Bombyx mori OX=7091 GN=100101181 PE=2 SV=1	1.07E-130	100	100	sp|Q24251|ATP5H_DROME	ATP synthase subunit d, mitochondrial OS=Drosophila melanogaster OX=7227 GN=ATPsynD PE=2 SV=2	2.01E-70	59.88	93.29608939

P_KWMTBOMO06427	tr|Q2F5L0|Q2F5L0_BOMMO	Signal sequence receptor subunit alpha OS=Bombyx mori OX=7091 GN=778461 PE=2 SV=1	0	100	100	sp|P43307|SSRA_HUMAN	Translocon-associated protein subunit alpha OS=Homo sapiens OX=9606 GN=SSR1 PE=1 SV=3	6.17E-70	50.45	79.56989247
P_KWMTBOMO06474	tr|Q1HQ62|Q1HQ62_BOMMO	Cytochrome c1 OS=Bombyx mori OX=7091 PE=2 SV=1	0	100	100	sp|P00125|CY1_BOVIN	Cytochrome c1, heme protein, mitochondrial OS=Bos taurus OX=9913 GN=CYC1 PE=1 SV=2	1.23E-115	58.746	98.37662338
P_KWMTBOMO06558	tr|H9JQL8|H9JQL8_BOMMO	N-acetyltransferase domain-containing protein OS=Bombyx mori OX=7091 GN=101746540 PE=4 SV=1	5.25E-137	100	100	sp|Q9QY36|NAA10_MOUSE	N-alpha-acetyltransferase 10 OS=Mus musculus OX=10090 GN=Naa10 PE=1 SV=1	3.50E-95	83.226	86.11111111
P_KWMTBOMO06595	tr|I3VR81|I3VR81_BOMMO	Aminopeptidase OS=Bombyx mori OX=7091 PE=2 SV=1	0	100	100	sp|P55786|PSA_HUMAN	Puromycin-sensitive aminopeptidase OS=Homo sapiens OX=9606 GN=NPEPPS PE=1 SV=2	0	57.324	100
P_KWMTBOMO06660	tr|Q2F640|Q2F640_BOMMO	Ubiquinol-cytochrome c reductase core protein II OS=Bombyx mori OX=7091 GN=100127121 PE=2 SV=1	0	100	100	sp|P22695|QCR2_HUMAN	Cytochrome b-c1 complex subunit 2, mitochondrial OS=Homo sapiens OX=9606 GN=UQCRC2 PE=1 SV=3	1.22E-72	34.615	95.19450801
P_KWMTBOMO06763	tr|H9JR70|H9JR70_BOMMO	Uncharacterized protein OS=Bombyx mori OX=7091 PE=4 SV=1	0	99.694	100	--	--	--	--	--
P_KWMTBOMO06772	tr|H9JR72|H9JR72_BOMMO	10 kDa heat shock protein, mitochondrial OS=Bombyx mori OX=7091 GN=101745352 PE=3 SV=1	6.79E-71	100	100	sp|Q5DC69|CH10_SCHJA	10 kDa heat shock protein, mitochondrial OS=Schistosoma japonicum OX=6182 GN=SJCHGC01960 PE=3 SV=2	1.03E-38	61.765	99.02912621
P_KWMTBOMO06927	tr|Q0N2S2|Q0N2S2_BOMMO	Small GTP-binding protein Rab10 OS=Bombyx mori OX=7091 GN=778528 PE=2 SV=1	2.84E-152	100	100	sp|P22127|RAB10_DIPOM	Ras-related protein Rab-10 OS=Diplobatis ommata OX=1870830 PE=2 SV=1	9.80E-110	75.862	100
P_KWMTBOMO06932	tr|O01678|O01678_BOMMO	3-oxoacyl-[acyl-carrier-protein] reductase OS=Bombyx mori OX=7091 PE=2 SV=1	0	43.121	100	sp|P12276|FAS_CHICK	Fatty acid synthase OS=Gallus gallus OX=9031 GN=FASN PE=1 SV=5	0	47.841	47.50941817
P_KWMTBOMO06999	tr|H9JLS3|H9JLS3_BOMMO	TPR_REGION domain-containing protein OS=Bombyx mori OX=7091 PE=3 SV=1	0	100	100	sp|P31948|STIP1_HUMAN	Stress-induced-phosphoprotein 1 OS=Homo sapiens OX=9606 GN=STIP1 PE=1 SV=1	0	52.007	100
P_KWMTBOMO07103	tr|Q2F5P8|Q2F5P8_BOMMO	Malate dehydrogenase OS=Bombyx mori OX=7091 PE=2 SV=1	0	100	100	sp|O88989|MDHC_RAT	Malate dehydrogenase, cytoplasmic OS=Rattus norvegicus OX=10116 GN=Mdh1 PE=1 SV=3	7.95E-157	65.653	99.39577039
P_KWMTBOMO07205	tr|H9JLI0|H9JLI0_BOMMO	Uncharacterized protein OS=Bombyx mori OX=7091 PE=3 SV=1	0	98.254	100	sp|Q24179|SLY1_DROME	Protein sly1 homolog OS=Drosophila melanogaster OX=7227 GN=Slh PE=2 SV=4	0	62.813	100
P_KWMTBOMO07214	tr|D5MTP2|D5MTP2_BOMMO	Bm8 interacting protein 2-11 OS=Bombyx mori OX=7091 PE=2 SV=1	0	94.898	78.82037534	sp|Q9VMA7|TGO1_DROME	Transport and Golgi organization protein 1 OS=Drosophila melanogaster OX=7227 GN=Tango1 PE=1 SV=2	6.97E-56	30.097	48.32439678
P_KWMTBOMO07247	tr|Q1HQ34|Q1HQ34_BOMMO	H+ transporting ATP synthase delta subunit OS=Bombyx mori OX=7091 GN=100101165 PE=2 SV=1	2.12E-115	100	100	sp|Q9D3D9|ATPD_MOUSE	ATP synthase subunit delta, mitochondrial OS=Mus musculus OX=10090 GN=Atp5f1d PE=1 SV=1	3.61E-44	55.385	80.74534161
P_KWMTBOMO07278	tr|H9JM89|H9JM89_BOMMO	PNP_UDP_1 domain-containing protein OS=Bombyx mori OX=7091 GN=101736970 PE=3 SV=1	0	98.214	99.11504425	sp|Q16831|UPP1_HUMAN	Uridine phosphorylase 1 OS=Homo sapiens OX=9606 GN=UPP1 PE=1 SV=1	1.13E-111	53.135	89.38053097
P_KWMTBOMO07368	tr|H9JMD1|H9JMD1_BOMMO	Uncharacterized protein OS=Bombyx mori OX=7091 GN=101744608 PE=3 SV=1	8.23E-94	100	100	sp|P24310|CX7A1_HUMAN	Cytochrome c oxidase subunit 7A1, mitochondrial OS=Homo sapiens OX=9606 GN=COX7A1 PE=1 SV=2	3.33E-12	47.458	45.38461538
P_KWMTBOMO07617	tr|H9IVM3|H9IVM3_BOMMO	Uncharacterized protein OS=Bombyx mori OX=7091 PE=3 SV=1	0	100	92.1686747	sp|Q5ZJ06|MAGT1_CHICK	Magnesium transporter protein 1 OS=Gallus gallus OX=9031 GN=MAGT1 PE=2 SV=1	3.74E-114	50.943	95.78313253
P_KWMTBOMO07685	tr|Q2F5V6|Q2F5V6_BOMMO	Mitochondrial import inner membrane translocase subunit OS=Bombyx mori OX=7091 GN=692908 PE=2 SV=1	3.63E-64	100	100	sp|Q9Y1A3|TIM8_DROME	Mitochondrial import inner membrane translocase subunit Tim8 OS=Drosophila melanogaster OX=7227 GN=Tim8 PE=3 SV=1	2.13E-38	69.88	93.25842697
P_KWMTBOMO07723	tr|H9IVI5|H9IVI5_BOMMO	Eukaryotic translation initiation factor 5B OS=Bombyx mori OX=7091 PE=4 SV=1	0	96.413	100	sp|O60841|IF2P_HUMAN	Eukaryotic translation initiation factor 5B OS=Homo sapiens OX=9606 GN=EIF5B PE=1 SV=4	1.08E-159	65.746	39.34782609
P_KWMTBOMO07761	tr|H9IUG4|H9IUG4_BOMMO	Uncharacterized protein OS=Bombyx mori OX=7091 PE=4 SV=1	0	94.605	26.40722724	sp|Q8MSS1|LVA_DROME	Protein lava lamp OS=Drosophila melanogaster OX=7227 GN=lva PE=1 SV=2	5.58E-15	35.644	7.01876303
P_KWMTBOMO07820	tr|H9IVD8|H9IVD8_BOMMO	Uncharacterized protein OS=Bombyx mori OX=7091 GN=101745384 PE=3 SV=1	0	96.905	100	sp|O95757|HS74L_HUMAN	Heat shock 70 kDa protein 4L OS=Homo sapiens OX=9606 GN=HSPA4L PE=1 SV=3	0	48.157	100
P_KWMTBOMO07821	tr|H9IUJ6|H9IUJ6_BOMMO	Transaldolase OS=Bombyx mori OX=7091 PE=3 SV=1	0	100	100	sp|Q2TBL6|TALDO_BOVIN	Transaldolase OS=Bos taurus OX=9913 GN=TALDO1 PE=2 SV=1	4.86E-159	68.598	98.79518072
P_KWMTBOMO07855	tr|Q2F6C2|Q2F6C2_BOMMO	CCT-beta OS=Bombyx mori OX=7091 GN=692797 PE=2 SV=1	0	100	100	sp|Q3ZBH0|TCPB_BOVIN	T-complex protein 1 subunit beta OS=Bos taurus OX=9913 GN=CCT2 PE=1 SV=3	0	72.15	99.81343284
P_KWMTBOMO07952	tr|H9IUR1|H9IUR1_BOMMO	Histidine--tRNA ligase OS=Bombyx mori OX=7091 PE=3 SV=1	0	66.062	100	sp|Q61035|HARS1_MOUSE	Histidine--tRNA ligase, cytoplasmic OS=Mus musculus OX=10090 GN=Hars1 PE=1 SV=2	0	67.683	95.53398058
P_KWMTBOMO08040	tr|H9IUV5|H9IUV5_BOMMO	Inosine-5'-monophosphate dehydrogenase OS=Bombyx mori OX=7091 GN=101740233 PE=3 SV=1	0	100	100	sp|Q07152|IMDH_DROME	Inosine-5'-monophosphate dehydrogenase OS=Drosophila melanogaster OX=7227 GN=ras PE=1 SV=1	0	79.093	99.0234375
P_KWMTBOMO08045	tr|H9IUV7|H9IUV7_BOMMO	Leucyl-tRNA synthetase OS=Bombyx mori OX=7091 PE=3 SV=1	0	99.658	99.20724802	sp|Q9P2J5|SYLC_HUMAN	Leucine--tRNA ligase, cytoplasmic OS=Homo sapiens OX=9606 GN=LARS1 PE=1 SV=2	0	65.984	99.54699887
P_KWMTBOMO08046	tr|H9IUV7|H9IUV7_BOMMO	Leucyl-tRNA synthetase OS=Bombyx mori OX=7091 PE=3 SV=1	0	99.254	97.10144928	sp|Q8BMJ2|SYLC_MOUSE	Leucine--tRNA ligase, cytoplasmic OS=Mus musculus OX=10090 GN=Lars1 PE=1 SV=2	1.02E-65	41.971	99.27536232
P_KWMTBOMO08056	tr|H9IV39|H9IV39_BOMMO	Rab GDP dissociation inhibitor OS=Bombyx mori OX=7091 GN=101738804 PE=3 SV=1	0	100	100	sp|P21856|GDIA_BOVIN	Rab GDP dissociation inhibitor alpha OS=Bos taurus OX=9913 GN=GDI1 PE=1 SV=1	0	68.75	100
P_KWMTBOMO08064	tr|Q5UAR1|Q5UAR1_BOMMO	60S ribosomal protein L27a OS=Bombyx mori OX=7091 GN=RpL27A PE=2 SV=1	3.15E-107	100	100	sp|P47830|RL27A_XENLA	60S ribosomal protein L27a OS=Xenopus laevis OX=8355 GN=rpl27a PE=2 SV=2	1.19E-82	77.027	100
P_KWMTBOMO08098	tr|G9FL14|G9FL14_BOMMO	DNA supercoiling factor OS=Bombyx mori OX=7091 GN=LOC692759 PE=2 SV=1	1.98E-48	100	100	sp|B5X4E0|CALUB_SALSA	Calumenin-B OS=Salmo salar OX=8030 GN=calub PE=2 SV=1	7.32E-13	49.333	100

P_KWMTBOMO08204	tr|H9JIZ5|H9JIZ5_BOMMO	Arf-GAP domain-containing protein OS=Bombyx mori OX=7091 GN=101746692 PE=4 SV=1	0	100	100	sp|Q28CM8|ARFG2_XENTR	ADP-ribosylation factor GTPase-activating protein 2 OS=Xenopus tropicalis OX=8364 GN=arfgap2 PE=2 SV=1	1.27E-99	39.455	100
P_KWMTBOMO08241	tr|A5JTL8|A5JTL8_BOMMO	Pyruvate dehydrogenase E1 component subunit alpha OS=Bombyx mori OX=7091 GN=l(b002) PE=2 SV=1	0	100	100	sp|P52899|ODPA_CAEEL	Probable pyruvate dehydrogenase E1 component subunit alpha, mitochondrial OS=Caenorhabditis elegans OX=6239 GN=T05H10.6 PE=3 SV=1	0	63.636	96.49122807
P_KWMTBOMO08343	tr|H9JI86|H9JI86_BOMMO	Uncharacterized protein OS=Bombyx mori OX=7091 PE=4 SV=1	0	100	71.51898734	sp|P54802|ANAG_HUMAN	Alpha-N-acetylglucosaminidase OS=Homo sapiens OX=9606 GN=NAGLU PE=1 SV=2	2.63E-165	41.81	67.61603376
P_KWMTBOMO08464	sp|P21828|FIBL_BOMMO	Fibroin light chain OS=Bombyx mori OX=7091 GN=FIBL PE=1 SV=1	0	100	100	sp|P21828|FIBL_BOMMO	Fibroin light chain OS=Bombyx mori OX=7091 GN=FIBL PE=1 SV=1	0	100	100
P_KWMTBOMO08552	tr|H9JIG6|H9JIG6_BOMMO	BEACH-type PH domain-containing protein OS=Bombyx mori OX=7091 PE=4 SV=1	0	99.482	84.02710552	sp|Q9W4E2|NBEA_DROME	Neurobeachin OS=Drosophila melanogaster OX=7227 GN=rg PE=1 SV=4	0	83.709	27.92836399
P_KWMTBOMO08578	tr|H9JDK4|H9JDK4_BOMMO	Multifunctional fusion protein OS=Bombyx mori OX=7091 GN=101741966 PE=3 SV=1	0	100	100	sp|Q7SY23|AL4A1_DANRE	Delta-1-pyrroline-5-carboxylate dehydrogenase, mitochondrial OS=Danio rerio OX=7955 GN=aldh4a1 PE=2 SV=1	0	55.159	94.33628319
P_KWMTBOMO08584	tr|H9JDK7|H9JDK7_BOMMO	Uncharacterized protein OS=Bombyx mori OX=7091 PE=4 SV=1	0	99.913	57.49373434	sp|O15027|SC16A_HUMAN	Protein transport protein Sec16A OS=Homo sapiens OX=9606 GN=SEC16A PE=1 SV=4	3.13E-60	34.081	22.35588972
P_KWMTBOMO08596	tr|H9JEM4|H9JEM4_BOMMO	Uncharacterized protein OS=Bombyx mori OX=7091 PE=3 SV=1	1.43E-162	100	97.69585253	sp|Q7SXP0|S22BB_DANRE	Vesicle-trafficking protein SEC22b-B OS=Danio rerio OX=7955 GN=sec22bb PE=2 SV=1	4.44E-87	56.621	100
P_KWMTBOMO08672	tr|Q1HQ66|Q1HQ66_BOMMO	AIR carboxylase OS=Bombyx mori OX=7091 GN=732910 PE=1 SV=1	0	100	100	sp|Q9I7S8|PUR6_DROME	Multifunctional protein ADE2 OS=Drosophila melanogaster OX=7227 GN=Paics PE=2 SV=2	0	69.007	97.17647059
P_KWMTBOMO08699	tr|Q5UAT3|Q5UAT3_BOMMO	60S ribosomal protein L7a OS=Bombyx mori OX=7091 GN=RpL7A PE=2 SV=1	0	100	100	sp|P46223|RL7A_DROME	60S ribosomal protein L7a OS=Drosophila melanogaster OX=7227 GN=RpL7A PE=1 SV=2	1.06E-136	75.984	94.7761194
P_KWMTBOMO08707	tr|H9JDR8|H9JDR8_BOMMO	Elongator complex protein 3 OS=Bombyx mori OX=7091 PE=3 SV=1	0	99.66	82.4684432	sp|Q5ZHS1|ELP3_CHICK	Elongator complex protein 3 OS=Gallus gallus OX=9031 GN=ELP3 PE=2 SV=1	0	84.962	74.61430575
P_KWMTBOMO08734	tr|H9JEG2|H9JEG2_BOMMO	Uncharacterized protein OS=Bombyx mori OX=7091 PE=4 SV=1	0	96.168	71.40335393	sp|A2AWA9|RBGP1_MOUSE	Rab GTPase-activating protein 1 OS=Mus musculus OX=10090 GN=Rabgap1 PE=1 SV=1	0	39.326	86.40776699
P_KWMTBOMO08760	tr|H9JDT4|H9JDT4_BOMMO	Phosphoglycerate kinase OS=Bombyx mori OX=7091 PE=3 SV=1	0	100	94.93975904	sp|Q01604|PGK_DROME	Phosphoglycerate kinase OS=Drosophila melanogaster OX=7227 GN=Pgk PE=2 SV=2	0	77.349	100
P_KWMTBOMO08815	tr|H9JEC2|H9JEC2_BOMMO	Uncharacterized protein OS=Bombyx mori OX=7091 GN=101746856 PE=4 SV=1	0	95.364	100	sp|Q767L0|ABCF1_PIG	ATP-binding cassette sub-family F member 1 OS=Sus scrofa OX=9823 GN=ABCF1 PE=3 SV=1	0	63.973	68.75
P_KWMTBOMO08894	tr|Q5UAN9|Q5UAN9_BOMMO	Ribosomal protein S5 OS=Bombyx mori OX=7091 GN=RpS5 PE=2 SV=1	3.62E-166	100	100	sp|P46782|RS5_HUMAN	40S ribosomal protein S5 OS=Homo sapiens OX=9606 GN=RPS5 PE=1 SV=4	1.84E-134	91.327	89.49771689
P_KWMTBOMO08930	tr|H9JDX6|H9JDX6_BOMMO	S-formylglutathione hydrolase OS=Bombyx mori OX=7091 PE=3 SV=1	0	100	89.54703833	sp|Q9GJT2|ESTD_PIG	S-formylglutathione hydrolase OS=Sus scrofa OX=9823 GN=ESD PE=2 SV=1	1.95E-129	61.754	99.30313589
P_KWMTBOMO08961	tr|Q5UAQ8|Q5UAQ8_BOMMO	60S ribosomal protein L30 OS=Bombyx mori OX=7091 GN=RpL30 PE=2 SV=1	2.87E-80	100	100	sp|P58375|RL30_SPOFR	60S ribosomal protein L30 OS=Spodoptera frugiperda OX=7108 GN=RpL30 PE=3 SV=1	9.75E-78	99.099	98.2300885
P_KWMTBOMO08991	tr|H9JE36|H9JE36_BOMMO	Succinate-CoA ligase subunit beta OS=Bombyx mori OX=7091 PE=3 SV=1	0	99.729	80.92105263	sp|Q9Z2I9|SUCB1_MOUSE	Succinate--CoA ligase [ADP-forming] subunit beta, mitochondrial OS=Mus musculus OX=10090 GN=Sucla2 PE=1 SV=2	0	62.319	90.78947368
P_KWMTBOMO09050	tr|H9JDA0|H9JDA0_BOMMO	Uncharacterized protein OS=Bombyx mori OX=7091 PE=4 SV=1	0	100	92.79661017	sp|Q96RQ3|MCCA_HUMAN	Methylcrotonoyl-CoA carboxylase subunit alpha, mitochondrial OS=Homo sapiens OX=9606 GN=MCCC1 PE=1 SV=3	0	56.278	95.62146893
P_KWMTBOMO09183	tr|H9J1H4|H9J1H4_BOMMO	Uncharacterized protein OS=Bombyx mori OX=7091 PE=4 SV=1	0	99.804	46.6179159	sp|O00410|IPO5_HUMAN	Importin-5 OS=Homo sapiens OX=9606 GN=IPO5 PE=1 SV=4	0	52.516	99.90859232
P_KWMTBOMO09232	tr|Q5UAS9|Q5UAS9_BOMMO	Ribosomal protein L10A OS=Bombyx mori OX=7091 GN=RpL10A PE=2 SV=1	4.16E-162	100	100	sp|Q963B6|RL10A_SPOFR	60S ribosomal protein L10a OS=Spodoptera frugiperda OX=7108 GN=RpL10A PE=2 SV=1	1.14E-156	96.774	100
P_KWMTBOMO09236	tr|H9J1E8|H9J1E8_BOMMO	Proteasome endopeptidase complex OS=Bombyx mori OX=7091 GN=101745852 PE=4 SV=1	4.69E-170	100	100	sp|P28072|PSB6_HUMAN	Proteasome subunit beta type-6 OS=Homo sapiens OX=9606 GN=PSMB6 PE=1 SV=4	7.04E-108	67.308	92.44444444
P_KWMTBOMO09250	tr|H9J1E3|H9J1E3_BOMMO	IU_nuc_hydro domain-containing protein OS=Bombyx mori OX=7091 PE=4 SV=1	0	98.442	96.3963964	--	--	--	--	--
P_KWMTBOMO09355	tr|Q6T3A7|Q6T3A7_BOMMO	Thiol peroxiredoxin OS=Bombyx mori OX=7091 GN=692638 PE=2 SV=1	5.60E-148	100	100	sp|Q9V3P0|PRDX1_DROME	Peroxiredoxin 1 OS=Drosophila melanogaster OX=7227 GN=Jafrac1 PE=1 SV=1	8.32E-117	81.152	97.94871795
P_KWMTBOMO09387	sp|H9JU86|POXIN_BOMMO	Poxin OS=Bombyx mori OX=7091 PE=1 SV=1	4.15E-180	100	100	sp|H9JU86|POXIN_BOMMO	Poxin OS=Bombyx mori OX=7091 PE=1 SV=1	2.70E-179	100	100
P_KWMTBOMO09390	tr|H9JU84|H9JU84_BOMMO	Splicing factor, arginine/serine-rich 1 OS=Bombyx mori OX=7091 PE=3 SV=1	6.24E-155	100	100	sp|Q6NYA0|SRS1B_DANRE	Serine/arginine-rich splicing factor 1B OS=Danio rerio OX=7955 GN=srsf1b PE=2 SV=1	3.58E-87	71.939	91.1627907
P_KWMTBOMO09474	tr|H9JU51|H9JU51_BOMMO	Malate dehydrogenase OS=Bombyx mori OX=7091 GN=101736985 PE=3 SV=1	0	100	100	sp|P40926|MDHM_HUMAN	Malate dehydrogenase, mitochondrial OS=Homo sapiens OX=9606 GN=MDH2 PE=1 SV=3	4.35E-146	60.294	99.70674487
P_KWMTBOMO09553	tr|Q1HPN7|Q1HPN7_BOMMO	Fructose-bisphosphate aldolase OS=Bombyx mori OX=7091 GN=778467 PE=2 SV=1	0	100	100	sp|P07764|ALF_DROME	Fructose-bisphosphate aldolase OS=Drosophila melanogaster OX=7227 GN=Ald1 PE=1 SV=5	0	82.143	100
P_KWMTBOMO09597	tr|H9JXD6|H9JXD6_BOMMO	Carrier domain-containing protein OS=Bombyx mori OX=7091 PE=3 SV=1	0	99.659	63.28293737	sp|Q63ZT8|AL1L1_XENTR	Cytosolic 10-formyltetrahydrofolate dehydrogenase OS=Xenopus tropicalis OX=8364 GN=aldh1l1 PE=2 SV=1	0	63.865	98.92008639

P_KWMTBOMO09631	tr|H9JTW9|H9JTW9_BOMMO	GOLD domain-containing protein OS=Bombyx mori OX=7091 GN=101737070 PE=3 SV=1	5.12E-155	100	100	sp|P49020|TMED2_CRIGR	Transmembrane emp24 domain-containing protein 2 (Fragment) OS=Cricetulus griseus OX=10029 GN=TMED2 PE=1 SV=1	4.26E-87	63.587	90.19607843
P_KWMTBOMO09641	tr|Q2F671|Q2F671_BOMMO	NIPSNAP protein OS=Bombyx mori OX=7091 PE=2 SV=1	0	100	97.8021978	sp|Q9VXK0|NIPSN_DROME	Protein NipSnap OS=Drosophila melanogaster OX=7227 GN=Nipsnap PE=2 SV=2	9.40E-141	68.498	100
P_KWMTBOMO09672	tr|H9JTU8|H9JTU8_BOMMO	Uncharacterized protein OS=Bombyx mori OX=7091 PE=4 SV=1	7.15E-83	98.276	42.64705882	sp|P48738|PIPNA_RABIT	Phosphatidylinositol transfer protein alpha isoform OS=Oryctolagus cuniculus OX=9986 GN=PITPNA PE=3 SV=2	1.85E-115	59.928	100
P_KWMTBOMO09788	tr|Q2QEH2|Q2QEH2_BOMMO	Cellular retinoic acid binding protein OS=Bombyx mori OX=7091 GN=BmFABP1 PE=2 SV=1	1.36E-94	100	100	sp|C4N147|FABP1_DORPE	Sodium/calcium exchanger regulatory protein 1 OS=Doryteuthis pealeii OX=1051067 PE=1 SV=1	2.61E-37	46.4	94.6969697
P_KWMTBOMO09811	tr|H9JUC9|H9JUC9_BOMMO	PH domain-containing protein OS=Bombyx mori OX=7091 PE=4 SV=1	0	92.813	66.07869742	sp|P22059|OSBP1_HUMAN	Oxysterol-binding protein 1 OS=Homo sapiens OX=9606 GN=OSBP PE=1 SV=1	0	49.396	100
P_KWMTBOMO09838	tr|H9JUC0|H9JUC0_BOMMO	Asparagine--tRNA ligase OS=Bombyx mori OX=7091 PE=3 SV=1	0	99.104	100	sp|Q2KJG3|SYNC_BOVIN	Asparagine--tRNA ligase, cytoplasmic OS=Bos taurus OX=9913 GN=NARS PE=2 SV=3	0	70.599	99.45848375
P_KWMTBOMO09900	tr|Q1HPY7|Q1HPY7_BOMMO	Phosphatidylethanolamine binding protein isoform 2 OS=Bombyx mori OX=7091 PE=2 SV=1	2.65E-142	99.487	43.72197309	sp|O16264|PEBPH_CAEEL	Phosphatidylethanolamine-binding protein homolog F40A3.3 OS=Caenorhabditis elegans OX=6239 GN=F40A3.3 PE=3 SV=1	8.92E-76	60	42.60089686
P_KWMTBOMO09934	tr|H9JUN1|H9JUN1_BOMMO	Phosphoglycerate mutase OS=Bombyx mori OX=7091 PE=3 SV=1	0	100	100	sp|Q5ZLN1|PGAM1_CHICK	Phosphoglycerate mutase 1 OS=Gallus gallus OX=9031 GN=PGAM1 PE=1 SV=3	4.89E-125	67.589	99.21568627
P_KWMTBOMO09954	tr|H9JUM2|H9JUM2_BOMMO	Proline dehydrogenase OS=Bombyx mori OX=7091 PE=3 SV=1	0	100	58.63636364	sp|Q04499|PROD_DROME	Proline dehydrogenase 1, mitochondrial OS=Drosophila melanogaster OX=7227 GN=slgA PE=2 SV=2	0	60.081	74.39393939
P_KWMTBOMO10049	sp|Q5UAP8|RL38_BOMMO	60S ribosomal protein L38 OS=Bombyx mori OX=7091 GN=RpL38 PE=3 SV=1	8.84E-45	100	100	sp|Q5UAP8|RL38_BOMMO	60S ribosomal protein L38 OS=Bombyx mori OX=7091 GN=RpL38 PE=3 SV=1	5.76E-44	100	100
P_KWMTBOMO10128	tr|H9J806|H9J806_BOMMO	Uncharacterized protein OS=Bombyx mori OX=7091 PE=3 SV=1	1.66E-70	100	91.58878505	sp|Q9W141|ATPK_DROME	Putative ATP synthase subunit f, mitochondrial OS=Drosophila melanogaster OX=7227 GN=ATPsynF PE=3 SV=1	7.40E-60	75.701	100
P_KWMTBOMO10129	tr|Q2F5X3|Q2F5X3_BOMMO	26S proteasome non-ATPase regulatory subunit 4 OS=Bombyx mori OX=7091 GN=778515 PE=2 SV=1	0	100	100	sp|P55035|PSMD4_DROME	26S proteasome non-ATPase regulatory subunit 4 OS=Drosophila melanogaster OX=7227 GN=Rpn10 PE=1 SV=2	1.63E-145	60.677	100
P_KWMTBOMO10147	tr|H9J7Z3|H9J7Z3_BOMMO	RRM domain-containing protein OS=Bombyx mori OX=7091 PE=3 SV=1	9.38E-80	85.106	100	sp|P30352|SRSF2_CHICK	Serine/arginine-rich splicing factor 2 OS=Gallus gallus OX=9031 GN=SRSF2 PE=2 SV=1	6.66E-51	75.49	57.62711864
P_KWMTBOMO10169	tr|H9J7Y6|H9J7Y6_BOMMO	OMPdecase OS=Bombyx mori OX=7091 PE=3 SV=1	0	100	100	sp|Q01637|UMPS_DROME	Uridine 5'-monophosphate synthase OS=Drosophila melanogaster OX=7227 GN=r-l PE=2 SV=2	6.51E-172	50.924	100
P_KWMTBOMO10212	tr|Q0N2R7|Q0N2R7_BOMMO	Signal recognition particle subunit SRP68 OS=Bombyx mori OX=7091 GN=SRP68 PE=2 SV=1	0	100	100	sp|Q9VSS2|SRP68_DROME	Signal recognition particle subunit SRP68 OS=Drosophila melanogaster OX=7227 GN=Srp68 PE=2 SV=1	0	50.413	100
P_KWMTBOMO10243	tr|H9JCR8|H9JCR8_BOMMO	Ubiquitin carboxyl-terminal hydrolase 7 OS=Bombyx mori OX=7091 PE=3 SV=1	0	95.544	100	sp|Q4VSI4|UBP7_RAT	Ubiquitin carboxyl-terminal hydrolase 7 OS=Rattus norvegicus OX=10116 GN=Usp7 PE=1 SV=1	0	60.572	96.69642857
P_KWMTBOMO10283	tr|H9JCM2|H9JCM2_BOMMO	PCI domain-containing protein OS=Bombyx mori OX=7091 GN=692900 PE=3 SV=1	0	100	100	sp|Q9D8W5|PSD12_MOUSE	26S proteasome non-ATPase regulatory subunit 12 OS=Mus musculus OX=10090 GN=Psmd12 PE=1 SV=4	0	59.071	100
P_KWMTBOMO10310	tr|H9JCL3|H9JCL3_BOMMO	Uncharacterized protein OS=Bombyx mori OX=7091 PE=3 SV=1	1.43E-167	100	92.62295082	sp|Q91XV4|DCXR_MESAU	L-xylulose reductase OS=Mesocricetus auratus OX=10036 GN=DCXR PE=1 SV=1	3.99E-99	55.328	100
P_KWMTBOMO10337	tr|H9JCI5|H9JCI5_BOMMO	ERF-3 OS=Bombyx mori OX=7091 PE=4 SV=1	0	100	96.32352941	sp|Q8R050|ERF3A_MOUSE	Eukaryotic peptide chain release factor GTP-binding subunit ERF3A OS=Mus musculus OX=10090 GN=Gspt1 PE=1 SV=2	0	66.599	90.25735294
P_KWMTBOMO10354	tr|H9JCH9|H9JCH9_BOMMO	PP28 domain-containing protein OS=Bombyx mori OX=7091 GN=101735383 PE=4 SV=1	1.27E-121	100	100	sp|Q13442|HAP28_HUMAN	28 kDa heat- and acid-stable phosphoprotein OS=Homo sapiens OX=9606 GN=PDAP1 PE=1 SV=1	2.80E-30	50	91.86046512
P_KWMTBOMO10453	tr|Q1HQ70|Q1HQ70_BOMMO	Transgelin OS=Bombyx mori OX=7091 PE=2 SV=1	5.19E-142	100	100	sp|Q24799|MYPH_ECHGR	Myophilin OS=Echinococcus granulosus OX=6210 PE=2 SV=1	1.03E-54	48.168	100
P_KWMTBOMO10489	tr|H9JC66|H9JC66_BOMMO	Dihydropyrimidine dehydrogenase [NADP(+)] OS=Bombyx mori OX=7091 PE=3 SV=1	0	96.884	100	sp|Q6NYG8|DPYD_DANRE	Dihydropyrimidine dehydrogenase [NADP(+)] OS=Danio rerio OX=7955 GN=dpyd PE=2 SV=1	0	66.246	100
P_KWMTBOMO10508	tr|H9JC76|H9JC76_BOMMO	Citrate hydro-lyase OS=Bombyx mori OX=7091 PE=3 SV=1	0	99.636	69.67005076	sp|P34455|ACON_CAEEL	Probable aconitate hydratase, mitochondrial OS=Caenorhabditis elegans OX=6239 GN=aco-2 PE=3 SV=2	0	78.238	95.05076142
P_KWMTBOMO10558	tr|H9JC99|H9JC99_BOMMO	3-hydroxyisobutyrate dehydrogenase OS=Bombyx mori OX=7091 PE=3 SV=1	0	100	100	sp|P29266|3HIDH_RAT	3-hydroxyisobutyrate dehydrogenase, mitochondrial OS=Rattus norvegicus OX=10116 GN=Hibadh PE=1 SV=3	3.18E-89	46.602	95.96273292
P_KWMTBOMO10591	tr|Q6PUF7|Q6PUF7_BOMMO	40S ribosomal protein S24 OS=Bombyx mori OX=7091 GN=100101150 PE=2 SV=1	4.69E-94	100	100	sp|Q962Q6|RS24_SPOFR	40S ribosomal protein S24 OS=Spodoptera frugiperda OX=7108 GN=RpS24 PE=2 SV=1	2.80E-92	98.485	100
P_KWMTBOMO10779	tr|Q0N2R5|Q0N2R5_BOMMO	Signal recognition particle receptor alpha subunit OS=Bombyx mori OX=7091 PE=2 SV=1	0	99.51	100	sp|Q9U5L1|SRPRA_DROME	Signal recognition particle receptor subunit alpha homolog OS=Drosophila melanogaster OX=7227 GN=SrpRalpha PE=1 SV=2	0	59.486	100
P_KWMTBOMO10816	tr|H9JFZ5|H9JFZ5_BOMMO	Pyruvate carboxylase OS=Bombyx mori OX=7091 PE=4 SV=1	0	97.263	98.9001692	sp|P11498|PYC_HUMAN	Pyruvate carboxylase, mitochondrial OS=Homo sapiens OX=9606 GN=PC PE=1 SV=2	0	68.512	97.80033841
P_KWMTBOMO10916	tr|Q1HQ44|Q1HQ44_BOMMO	Proteasome subunit alpha type OS=Bombyx mori OX=7091 PE=2 SV=1	0	100	100	sp|O70435|PSA3_MOUSE	Proteasome subunit alpha type-3 OS=Mus musculus OX=10090 GN=Psma3 PE=1 SV=3	2.47E-130	65.49	100
P_KWMTBOMO10965	tr|Q1HQC6|Q1HQC6_BOMMO	26S protease regulatory subunit 6B OS=Bombyx mori OX=7091 PE=2 SV=1	0	100	100	sp|P46507|PRS6B_MANSE	26S proteasome regulatory subunit 6B OS=Manduca sexta OX=7130 PE=2 SV=1	0	98.313	100
P_KWMTBOMO11021	tr|Q5UAM3|Q5UAM3_BOMMO	Ribosomal protein S19 OS=Bombyx mori OX=7091 GN=RpS19 PE=2 SV=1	6.33E-111	100	100	sp|P39018|RS19A_DROME	40S ribosomal protein S19a OS=Drosophila melanogaster OX=7227 GN=RpS19a PE=1 SV=3	5.94E-71	66.892	96.73202614

P_KWMTBOMO11064	tr|A0A1L6UW57|A0A1L6UW57_BOMMO	Hypoxia up-regulated-like protein OS=Bombyx mori OX=7091 PE=2 SV=1	0	100	100	sp|Q7ZUW2|HYOU1_DANRE	Hypoxia up-regulated protein 1 OS=Danio rerio OX=7955 GN=hyou1 PE=2 SV=1	0	44.531	98.6784141
P_KWMTBOMO11161	tr|H9JGE8|H9JGE8_BOMMO	26S proteasome non-ATPase regulatory subunit 8 OS=Bombyx mori OX=7091 GN=101737946 PE=3 SV=1	0	100	100	sp|Q9CX56|PSMD8_MOUSE	26S proteasome non-ATPase regulatory subunit 8 OS=Mus musculus OX=10090 GN=Psmd8 PE=1 SV=2	3.67E-110	60.075	100
P_KWMTBOMO11192	tr|C6L8Q2|C6L8Q2_BOMMO	Putative acetyl transferase OS=Bombyx mori OX=7091 GN=Bmat1 PE=2 SV=1	0	100	100	sp|P42765|THIM_HUMAN	3-ketoacyl-CoA thiolase, mitochondrial OS=Homo sapiens OX=9606 GN=ACAA2 PE=1 SV=2	5.87E-173	58.987	99.74747475
P_KWMTBOMO11413	tr|H9IX93|H9IX93_BOMMO	Alpha-amylase OS=Bombyx mori OX=7091 PE=3 SV=1	0	93.421	100	sp|Q0P5L8|KBL_BOVIN	2-amino-3-ketobutyrate coenzyme A ligase, mitochondrial OS=Bos taurus OX=9913 GN=GCAT PE=1 SV=1	1.85E-172	58.561	94.60093897
P_KWMTBOMO11486	tr|H9IXD2|H9IXD2_BOMMO	Proteasome endopeptidase complex OS=Bombyx mori OX=7091 PE=3 SV=1	1.39E-142	91.324	85.21400778	sp|P25789|PSA4_HUMAN	Proteasome subunit alpha type-4 OS=Homo sapiens OX=9606 GN=PSMA4 PE=1 SV=1	2.07E-149	82.278	92.21789883
P_KWMTBOMO11514	tr|H9IT60|H9IT60_BOMMO	PHB domain-containing protein OS=Bombyx mori OX=7091 PE=3 SV=1	6.72E-21	34.132	41.2345679	sp|Q9UJZ1|STML2_HUMAN	Stomatin-like protein 2, mitochondrial OS=Homo sapiens OX=9606 GN=STOML2 PE=1 SV=1	2.52E-149	68.687	73.33333333
P_KWMTBOMO11680	tr|H9J390|H9J390_BOMMO	Seryl-tRNA synthetase OS=Bombyx mori OX=7091 GN=101743384 PE=3 SV=1	0	100	100	sp|Q9GMB8|SYSC_BOVIN	Serine--tRNA ligase, cytoplasmic OS=Bos taurus OX=9913 GN=SARS1 PE=2 SV=3	0	69.495	100
P_KWMTBOMO11689	tr|H9J385|H9J385_BOMMO	Uncharacterized protein OS=Bombyx mori OX=7091 PE=4 SV=1	0	100	100	sp|O18373|SPS1_DROME	Inactive selenide, water dikinase-like protein OS=Drosophila melanogaster OX=7227 GN=SelD PE=2 SV=1	0	87.94	99.00497512
P_KWMTBOMO11710	sp|Q0ZB73|EIF3J_BOMMO	Eukaryotic translation initiation factor 3 subunit J OS=Bombyx mori OX=7091 PE=2 SV=1	9.13E-177	100	100	sp|Q0ZB73|EIF3J_BOMMO	Eukaryotic translation initiation factor 3 subunit J OS=Bombyx mori OX=7091 PE=2 SV=1	5.95E-176	100	100
P_KWMTBOMO11771	tr|Q1HQ18|Q1HQ18_BOMMO	Proteasome endopeptidase complex OS=Bombyx mori OX=7091 GN=100101166 PE=2 SV=1	4.64E-178	100	100	sp|P40304|PSB1_DROME	Proteasome subunit beta type-1 OS=Drosophila melanogaster OX=7227 GN=Prosbeta6 PE=2 SV=2	3.74E-105	62.009	98.70689655
P_KWMTBOMO11812	tr|Q1HQ98|Q1HQ98_BOMMO	Cytochrome c oxidase polypeptide IV OS=Bombyx mori OX=7091 GN=780848 PE=2 SV=1	3.90E-133	100	100	sp|O46578|COX41_GORGO	Cytochrome c oxidase subunit 4 isoform 1, mitochondrial (Fragment) OS=Gorilla gorilla gorilla OX=9595 GN=COX4I1 PE=3 SV=1	1.73E-38	48.12	74.71910112
P_KWMTBOMO11977	tr|H9J4D7|H9J4D7_BOMMO	Proteasome subunit beta OS=Bombyx mori OX=7091 GN=101740220 PE=3 SV=1	0	100	100	sp|P28075|PSB5_RAT	Proteasome subunit beta type-5 OS=Rattus norvegicus OX=10116 GN=Psmb5 PE=1 SV=3	2.21E-102	61.233	80.21201413
P_KWMTBOMO11987	tr|Q5UAN1|Q5UAN1_BOMMO	40S ribosomal protein S12 OS=Bombyx mori OX=7091 GN=RpS12 PE=2 SV=1	4.19E-99	100	100	sp|P80455|RS12_DROME	40S ribosomal protein S12 OS=Drosophila melanogaster OX=7227 GN=RpS12 PE=1 SV=2	1.09E-68	72.143	100
P_KWMTBOMO12094	tr|Q1HPM2|Q1HPM2_BOMMO	Phosphoenolpyruvate carboxykinase (GTP) OS=Bombyx mori OX=7091 PE=2 SV=1	0	99.365	99.84152139	sp|P20007|PCKG_DROME	Phosphoenolpyruvate carboxykinase [GTP] OS=Drosophila melanogaster OX=7227 GN=Pepck PE=2 SV=2	0	65.645	98.25673534
P_KWMTBOMO12193	tr|H9JXB0|H9JXB0_BOMMO	COX6C domain-containing protein OS=Bombyx mori OX=7091 GN=101746562 PE=3 SV=1	1.23E-53	100	100	sp|Q7YRK7|COX6C_TARSY	Cytochrome c oxidase subunit 6C OS=Tarsius syrichta OX=1868482 GN=COX6C PE=3 SV=1	1.86E-18	53.623	88.46153846
P_KWMTBOMO12199	tr|H9JXB2|H9JXB2_BOMMO	AAA domain-containing protein OS=Bombyx mori OX=7091 GN=101747034 PE=3 SV=1	0	100	100	sp|P54814|PRS8_MANSE	26S proteasome regulatory subunit 8 OS=Manduca sexta OX=7130 PE=2 SV=1	0	99.256	100
P_KWMTBOMO12208	tr|H9JE23|H9JE23_BOMMO	SAC domain-containing protein OS=Bombyx mori OX=7091 PE=4 SV=1	3.33E-156	100	35.95317726	sp|Q9W0I6|SAC1_DROME	Phosphatidylinositol-3-phosphatase SAC1 OS=Drosophila melanogaster OX=7227 GN=Sac1 PE=2 SV=1	0	49.144	97.65886288
P_KWMTBOMO12285	tr|Q93137|Q93137_BOMMO	Reverse transcriptase OS=Bombyx mori OX=7091 PE=4 SV=1	0	71.082	49.23913043	sp|O17449|TBB1_MANSE	Tubulin beta-1 chain OS=Manduca sexta OX=7130 PE=2 SV=1	0	99.769	23.47826087
P_KWMTBOMO12287	tr|Q5UAM2|Q5UAM2_BOMMO	40S ribosomal protein S20 OS=Bombyx mori OX=7091 GN=RpS20 PE=2 SV=1	3.74E-87	100	100	sp|P55828|RS20_DROME	40S ribosomal protein S20 OS=Drosophila melanogaster OX=7227 GN=RpS20 PE=1 SV=1	1.92E-68	83.74	100
P_KWMTBOMO12304	tr|H9JHX3|H9JHX3_BOMMO	4_1_CTD domain-containing protein OS=Bombyx mori OX=7091 PE=4 SV=1	0	98.443	72.63157895	sp|Q9V8R9|EPB41_DROME	Protein 4.1 homolog OS=Drosophila melanogaster OX=7227 GN=cora PE=1 SV=1	0	52.778	36.49122807
P_KWMTBOMO12428	sp|Q5UAS2|RL17_BOMMO	60S ribosomal protein L17 OS=Bombyx mori OX=7091 GN=RpL17 PE=2 SV=1	5.32E-141	100	100	sp|Q5UAS2|RL17_BOMMO	60S ribosomal protein L17 OS=Bombyx mori OX=7091 GN=RpL17 PE=2 SV=1	3.47E-140	100	100
P_KWMTBOMO12442	tr|H9IYM1|H9IYM1_BOMMO	Methenyltetrahydrofolate cyclohydrolase OS=Bombyx mori OX=7091 GN=101741628 PE=2 SV=1	0	100	95.25316456	sp|Q9H903|MTD2L_HUMAN	Probable bifunctional methylenetetrahydrofolate dehydrogenase/cyclohydrolase 2 OS=Homo sapiens OX=9606 GN=MTHFD2L PE=1 SV=3	1.20E-122	60.204	93.03797468
P_KWMTBOMO12448	tr|H9IYM5|H9IYM5_BOMMO	Secretory carrier-associated membrane protein OS=Bombyx mori OX=7091 PE=3 SV=1	1.69E-99	87.5	73.01587302	sp|Q8K021|SCAM1_MOUSE	Secretory carrier-associated membrane protein 1 OS=Mus musculus OX=10090 GN=Scamp1 PE=1 SV=1	8.88E-77	52.838	90.87301587
P_KWMTBOMO12461	tr|Q4JI71|Q4JI71_BOMMO	FK506-binding protein OS=Bombyx mori OX=7091 GN=FKBP45 PE=2 SV=1	0	100	100	sp|Q26486|FKBP4_SPOFR	46 kDa FK506-binding nuclear protein OS=Spodoptera frugiperda OX=7108 GN=FKBP46 PE=2 SV=1	0	71.838	100
P_KWMTBOMO12628	tr|H9IWC7|H9IWC7_BOMMO	Isoleucyl-tRNA synthetase OS=Bombyx mori OX=7091 GN=101742308 PE=3 SV=1	0	99.835	100	sp|Q8BU30|SYIC_MOUSE	Isoleucine--tRNA ligase, cytoplasmic OS=Mus musculus OX=10090 GN=Iars1 PE=1 SV=2	0	60.246	100
P_KWMTBOMO12682	tr|H9IWG1|H9IWG1_BOMMO	Complex I-B16.6 OS=Bombyx mori OX=7091 PE=3 SV=1	5.53E-112	100	100	sp|Q95KV7|NDUAD_BOVIN	NADH dehydrogenase [ubiquinone] 1 alpha subcomplex subunit 13 OS=Bos taurus OX=9913 GN=NDUFA13 PE=1 SV=3	3.49E-21	40.769	84.96732026
P_KWMTBOMO12706	tr|Q5UAQ2|Q5UAQ2_BOMMO	60S ribosomal protein L36 OS=Bombyx mori OX=7091 GN=RpL36 PE=2 SV=1	1.02E-81	100	100	sp|P49630|RL36_DROME	60S ribosomal protein L36 OS=Drosophila melanogaster OX=7227 GN=RpL36 PE=1 SV=1	4.53E-50	71.304	96.63865546
P_KWMTBOMO12878	tr|Q0KIX8|Q0KIX8_BOMMO	Glutamate synthase OS=Bombyx mori OX=7091 GN=BmGOGAT PE=2 SV=1	0	99.658	100	sp|Q18164|DPYD_CAEEL	Dihydropyrimidine dehydrogenase [NADP(+)] OS=Caenorhabditis elegans OX=6239 GN=dpyd-1 PE=3 SV=2	2.57E-20	29.206	15.39589443
P_KWMTBOMO12902	tr|H9IT95|H9IT95_BOMMO	Calreticulin OS=Bombyx mori OX=7091 PE=3 SV=1	0	100	100	sp|Q7Z1E6|CALR_BOMMO	Calreticulin OS=Bombyx mori OX=7091 GN=crt PE=1 SV=1	0	99.749	100

P_KWMTBOMO12903	tr|H9IT94|H9IT94_BOMMO	CP-type G domain-containing protein OS=Bombyx mori OX=7091 GN=101736688 PE=4 SV=1	0	100	100	sp|Q8MT06|GNL3_DROME	Guanine nucleotide-binding protein-like 3 homolog OS=Drosophila melanogaster OX=7227 GN=Ns1 PE=1 SV=2	4.41E-100	48.179	100
P_KWMTBOMO13301	tr|Q1HQ07|Q1HQ07_BOMMO	1110059p08rik-like protein OS=Bombyx mori OX=7091 GN=732946 PE=2 SV=1	0	100	100	sp|Q32L63|VTA1_BOVIN	Vacuolar protein sorting-associated protein VTA1 homolog OS=Bos taurus OX=9913 GN=VTA1 PE=2 SV=1	8.62E-93	47.352	100
P_KWMTBOMO13520	tr|Q2F5T2|Q2F5T2_BOMMO	Calmodulin OS=Bombyx mori OX=7091 PE=2 SV=1	5.64E-106	100	100	sp|P62153|CALMA_HALRO	Calmodulin-A OS=Halocynthia roretzi OX=7729 PE=1 SV=2	3.68E-105	100	100
P_KWMTBOMO13530	tr|Q5TLU4|Q5TLU4_BOMMO	Superoxide dismutase [Cu-Zn] OS=Bombyx mori OX=7091 GN=Cu/Zn SOD PE=2 SV=2	5.49E-110	100	100	sp|P82205|SODC_BOMMO	Superoxide dismutase [Cu-Zn] OS=Bombyx mori OX=7091 PE=1 SV=3	3.58E-109	100	100
P_KWMTBOMO13597	tr|H9JN56|H9JN56_BOMMO	GOLD domain-containing protein OS=Bombyx mori OX=7091 PE=3 SV=1	8.08E-165	100	100	sp|B4NKL0|TMEDE_DROWI	Transmembrane emp24 domain-containing protein eca OS=Drosophila willistoni OX=7260 GN=eca PE=3 SV=1	1.07E-127	80.952	96.77419355
P_KWMTBOMO13712	tr|H9JNA6|H9JNA6_BOMMO	E3 UFM1-protein ligase 1 homolog OS=Bombyx mori OX=7091 PE=3 SV=1	0	99.323	77.25490196	sp|Q0IG18|UFL1_AEDAE	E3 UFM1-protein ligase 1 homolog OS=Aedes aegypti OX=7159 GN=AAEL003536 PE=3 SV=1	0	47.135	100
P_KWMTBOMO13713	tr|Q1HPR2|Q1HPR2_BOMMO	Prefoldin subunit 3 OS=Bombyx mori OX=7091 PE=2 SV=1	1.83E-137	98.396	100	sp|Q9VGP6|PFD3_DROME	Prefoldin subunit 3 OS=Drosophila melanogaster OX=7227 GN=mgr PE=1 SV=3	6.30E-70	60.234	91.44385027
P_KWMTBOMO13721	tr|A1YM11|A1YM11_BOMMO	CCT-theta OS=Bombyx mori OX=7091 PE=2 SV=1	0	99.45	100	sp|Q6EE31|TCPQ_CHICK	T-complex protein 1 subunit theta OS=Gallus gallus OX=9031 GN=CCT8 PE=1 SV=3	0	63.72	99.63302752
P_KWMTBOMO13722	tr|H9JPQ0|H9JPQ0_BOMMO	Uncharacterized protein OS=Bombyx mori OX=7091 PE=3 SV=1	2.24E-39	100	73.56321839	sp|Q99417|MYCBP_HUMAN	c-Myc-binding protein OS=Homo sapiens OX=9606 GN=MYCBP PE=1 SV=3	2.31E-23	56.944	82.75862069
P_KWMTBOMO13723	tr|Q1HPX3|Q1HPX3_BOMMO	ATP synthase subunit O, mitochondrial OS=Bombyx mori OX=7091 PE=2 SV=1	1.17E-152	100	100	sp|Q24439|ATPO_DROME	ATP synthase subunit O, mitochondrial OS=Drosophila melanogaster OX=7227 GN=ATPsynO PE=2 SV=2	7.56E-89	61.692	96.1722488
P_KWMTBOMO13729	tr|Q684K3|Q684K3_BOMMO	Protein-synthesizing GTPase OS=Bombyx mori OX=7091 GN=eIF2g PE=2 SV=1	0	100	100	sp|Q24208|IF2G_DROME	Eukaryotic translation initiation factor 2 subunit 3 OS=Drosophila melanogaster OX=7227 GN=eIF2gamma PE=2 SV=1	0	84.12	99.57264957
P_KWMTBOMO13800	tr|H9JPL2|H9JPL2_BOMMO	60S ribosomal protein L18a OS=Bombyx mori OX=7091 PE=3 SV=1	4.32E-132	100	100	sp|Q8WQI7|RL18A_SPOFR	60S ribosomal protein L18a OS=Spodoptera frugiperda OX=7108 GN=RpL18A PE=2 SV=1	1.32E-126	95.48	100
P_KWMTBOMO13899	tr|H9JPY4|H9JPY4_BOMMO	Uncharacterized protein OS=Bombyx mori OX=7091 GN=101740613 PE=4 SV=1	1.40E-71	100	100	--	--	--	--	--
P_KWMTBOMO13923	tr|Q19P04|Q19P04_BOMMO	Acyl-coenzyme A dehydrogenase OS=Bombyx mori OX=7091 GN=acade PE=2 SV=1	0	100	100	sp|P45954|ACDSB_HUMAN	Short/branched chain specific acyl-CoA dehydrogenase, mitochondrial OS=Homo sapiens OX=9606 GN=ACADSB PE=1 SV=1	4.38E-162	64.848	99.39759036
P_KWMTBOMO13925	tr|H9JP55|H9JP55_BOMMO	Uncharacterized protein OS=Bombyx mori OX=7091 PE=4 SV=1	0	99.927	100	--	--	--	--	--
P_KWMTBOMO14024	tr|H9JPA3|H9JPA3_BOMMO	DJ-1 beta OS=Bombyx mori OX=7091 GN=100422789 PE=2 SV=1	2.25E-138	100	100	sp|Q9VA37|DJ1B_DROME	Protein dj-1beta OS=Drosophila melanogaster OX=7227 GN=dj-1beta PE=1 SV=3	5.03E-67	54.787	98.94736842
P_KWMTBOMO14160	tr|H9JPF8|H9JPF8_BOMMO	Iso_dh domain-containing protein OS=Bombyx mori OX=7091 PE=3 SV=1	0	100	100	sp|Q58CP0|IDH3G_BOVIN	Isocitrate dehydrogenase [NAD] subunit gamma, mitochondrial OS=Bos taurus OX=9913 GN=IDH3G PE=2 SV=1	5.17E-132	50.653	98.71134021
P_KWMTBOMO14478	tr|H9IS48|H9IS48_BOMMO	Uncharacterized protein OS=Bombyx mori OX=7091 GN=101742289 PE=3 SV=1	1.04E-66	100	100	sp|Q9W1V3|FBRL_DROME	rRNA 2'-O-methyltransferase fibrillarin OS=Drosophila melanogaster OX=7227 GN=Fib PE=2 SV=1	4.31E-61	94.737	100
P_KWMTBOMO14639	tr|Q5UAQ6|Q5UAQ6_BOMMO	60S ribosomal protein L32 OS=Bombyx mori OX=7091 GN=RpL32 PE=2 SV=1	1.84E-97	100	100	sp|Q962T1|RL32_SPOFR	60S ribosomal protein L32 OS=Spodoptera frugiperda OX=7108 GN=RpL32 PE=2 SV=1	6.75E-95	97.761	100
P_KWMTBOMO14715	tr|Q1HPL2|Q1HPL2_BOMMO	Phosphate carrier 1 OS=Bombyx mori OX=7091 GN=PiC PE=2 SV=1	0	100	100	sp|O61703|MPCP_CHOFU	Phosphate carrier protein, mitochondrial OS=Choristoneura fumiferana OX=7141 PE=2 SV=1	0	78.771	100
P_KWMTBOMO14761	tr|H9J2S8|H9J2S8_BOMMO	Dihydrolipoyllysine-residue succinyltransferase component of 2-oxoglutarate dehydrogenase complex, mitochondrial OS=Bombyx mori OX=7091 PE=3 SV=1	0	78.71	100	sp|P11179|ODO2_BOVIN	Dihydrolipoyllysine-residue succinyltransferase component of 2-oxoglutarate dehydrogenase complex, mitochondrial OS=Bos taurus OX=9913 GN=DLST PE=1 SV=2	8.56E-150	56.542	92.04301075
P_KWMTBOMO14845	tr|Q1HQC8|Q1HQC8_BOMMO	ARP1 actin-related protein 1-like protein A OS=Bombyx mori OX=7091 GN=732868 PE=2 SV=1	0	100	100	sp|P61162|ACTZ_CANLF	Alpha-centractin OS=Canis lupus familiaris OX=9615 GN=ACTR1A PE=2 SV=1	0	81.649	100
P_KWMTBOMO14850	tr|Q1HQ06|Q1HQ06_BOMMO	ATP synthase subunit e, mitochondrial OS=Bombyx mori OX=7091 PE=2 SV=1	2.78E-34	98.305	59.5959596	--	--	--	--	--
P_KWMTBOMO14877	tr|Q0ZB80|Q0ZB80_BOMMO	eIF2B-beta protein OS=Bombyx mori OX=7091 PE=2 SV=1	0	99.717	100	sp|Q5E9B4|EI2BB_BOVIN	Translation initiation factor eIF-2B subunit beta OS=Bos taurus OX=9913 GN=EIF2B2 PE=2 SV=1	1.50E-63	36.676	98.86685552
P_KWMTBOMO14936	tr|H9IU37|H9IU37_BOMMO	GB1/RHD3-type G domain-containing protein OS=Bombyx mori OX=7091 PE=3 SV=1	0	97.695	100	sp|Q9VC57|ATLAS_DROME	Atlastin OS=Drosophila melanogaster OX=7227 GN=atl PE=1 SV=1	0	68.275	97.82214156
P_KWMTBOMO14942	tr|H9IU76|H9IU76_BOMMO	AMP deaminase OS=Bombyx mori OX=7091 PE=3 SV=1	0	98.229	97.60638298	sp|Q02356|AMPD2_RAT	AMP deaminase 2 OS=Rattus norvegicus OX=10116 GN=Ampd2 PE=1 SV=2	0	59.916	95.21276596
P_KWMTBOMO14962	tr|H9IU27|H9IU27_BOMMO	Uncharacterized protein OS=Bombyx mori OX=7091 PE=4 SV=1	0	100	99.40652819	sp|Q14690|RRP5_HUMAN	Protein RRP5 homolog OS=Homo sapiens OX=9606 GN=PDCD11 PE=1 SV=3	2.60E-77	28.588	86.84470821
P_KWMTBOMO15035	tr|Q2WBZ0|Q2WBZ0_BOMMO	Chlorophyllide A binding protein OS=Bombyx mori OX=7091 GN=chbp PE=2 SV=1	0	94.715	71.02828443	sp|P53041|PPP5_HUMAN	Serine/threonine-protein phosphatase 5 OS=Homo sapiens OX=9606 GN=PPP5C PE=1 SV=1	0	63.389	12.63547449
P_KWMTBOMO15066	tr|Q2F6A1|Q2F6A1_BOMMO	Electron transfer flavoprotein subunit beta OS=Bombyx mori OX=7091 GN=692811 PE=2 SV=1	0	100	100	sp|Q9DCW4|ETFB_MOUSE	Electron transfer flavoprotein subunit beta OS=Mus musculus OX=10090 GN=Etfb PE=1 SV=3	2.31E-126	69.323	99.20948617

P_KWMTBOMO15121	tr|Q0N2R8|Q0N2R8_BOMMO	Signal recognition particle 54 kDa protein OS=Bombyx mori OX=7091 GN=SRP54 PE=2 SV=1	0	99.601	100	sp|Q7ZVN5|SRP54_DANRE	Signal recognition particle 54 kDa protein OS=Danio rerio OX=7955 GN=srp54 PE=2 SV=1	0	80.2	99.8003992
P_KWMTBOMO15125	tr|H9J651|H9J651_BOMMO	Adipocyte plasma membrane-associated protein OS=Bombyx mori OX=7091 PE=3 SV=1	0	99.398	94.32624113	sp|Q5ZIF1|APMAP_CHICK	Adipocyte plasma membrane-associated protein OS=Gallus gallus OX=9031 GN=APMAP PE=2 SV=1	2.85E-45	32.203	50.21276596
P_KWMTBOMO15179	tr|G9I6Y0|G9I6Y0_BOMMO	RING-type E3 ubiquitin transferase OS=Bombyx mori OX=7091 GN=100862840 PE=2 SV=1	0	85.632	100	sp|Q803I8|SYVN1_DANRE	E3 ubiquitin-protein ligase synoviolin OS=Danio rerio OX=7955 GN=syvn1 PE=2 SV=2	3.34E-177	67.761	64.17624521
P_KWMTBOMO15198	tr|B5AU20|B5AU20_BOMMO	UDP-glucuronosyltransferase OS=Bombyx mori OX=7091 GN=UGT2 PE=2 SV=1	0	100	100	sp|A0A291PQH4|UGT2_DACCO	UDP-glucosyltransferase 2 OS=Dactylopius coccus OX=765876 GN=UTG2 PE=1 SV=1	2.31E-59	29.352	94.0952381
P_KWMTBOMO15341	tr|H9J6I4|H9J6I4_BOMMO	Tryptophanyl-tRNA synthetase OS=Bombyx mori OX=7091 PE=3 SV=1	0	100	74.75	sp|P23381|SYWC_HUMAN	Tryptophan--tRNA ligase, cytoplasmic OS=Homo sapiens OX=9606 GN=WARS1 PE=1 SV=2	0	65.128	97.5
P_KWMTBOMO15357	tr|H9J6H5|H9J6H5_BOMMO	Prolyl-tRNA synthetase OS=Bombyx mori OX=7091 PE=3 SV=1	0	98.315	99.64028777	sp|P28668|SYEP_DROME	Bifunctional glutamate/proline--tRNA ligase OS=Drosophila melanogaster OX=7227 GN=GluProRS PE=1 SV=2	0	61.306	99.16067146
P_KWMTBOMO15365	tr|B0FRJ4|B0FRJ4_BOMMO	Fibroin heavy chain Fib-h (Fragment) OS=Bombyx mori OX=7091 PE=4 SV=1	4.87E-120	99.351	2.943987765	sp|Q99050|FIBH_BOMMA	Fibroin heavy chain (Fragment) OS=Bombyx mandarina OX=7092 GN=FIBH PE=2 SV=2	5.97E-138	97.765	3.421907857
P_KWMTBOMO15419	tr|H9J5I6|H9J5I6_BOMMO	Methyltransf_25 domain-containing protein OS=Bombyx mori OX=7091 PE=4 SV=1	0	100	99.15730337	sp|Q6PAK3|ANM8_MOUSE	Protein arginine N-methyltransferase 8 OS=Mus musculus OX=10090 GN=Prmt8 PE=1 SV=2	0	71.988	93.25842697
P_KWMTBOMO15485	tr|H9JMN2|H9JMN2_BOMMO	Telomere length regulation protein TEL2 homolog OS=Bombyx mori OX=7091 PE=3 SV=1	0	99.857	52.8831563	sp|Q14677|EPN4_HUMAN	Clathrin interactor 1 OS=Homo sapiens OX=9606 GN=CLINT1 PE=1 SV=1	9.68E-82	42.982	34.59787557
P_KWMTBOMO15518	tr|Q1HPL0|Q1HPL0_BOMMO	26S proteasome regulatory ATPase subunit 10B OS=Bombyx mori OX=7091 PE=2 SV=1	0	100	100	sp|P62334|PRS10_MOUSE	26S proteasome regulatory subunit 10B OS=Mus musculus OX=10090 GN=Psmc6 PE=1 SV=1	0	88.571	97.22222222
P_KWMTBOMO15520	tr|H9JMJ8|H9JMJ8_BOMMO	Transcription factor BTF3 OS=Bombyx mori OX=7091 GN=101747148 PE=3 SV=1	7.93E-125	100	100	sp|Q5M8V0|BT3L4_XENTR	Transcription factor BTF3 homolog 4 OS=Xenopus tropicalis OX=8364 GN=btf3l4 PE=2 SV=1	3.16E-70	73.248	90.22988506
P_KWMTBOMO15547	tr|H9JMI6|H9JMI6_BOMMO	Nop domain-containing protein OS=Bombyx mori OX=7091 PE=3 SV=1	0	91.939	99.35064935	sp|O00567|NOP56_HUMAN	Nucleolar protein 56 OS=Homo sapiens OX=9606 GN=NOP56 PE=1 SV=4	0	66.59	93.93939394
P_KWMTBOMO15629	tr|A0A1C9CW41|A0A1C9CW41_BOMMO	Phosphoacetylglucosamine mutase OS=Bombyx mori OX=7091 GN=pgm PE=2 SV=1	0	100	100	sp|O95394|AGM1_HUMAN	Phosphoacetylglucosamine mutase OS=Homo sapiens OX=9606 GN=PGM3 PE=1 SV=1	0	52.354	96.89781022
P_KWMTBOMO15665	tr|H9IYF4|H9IYF4_BOMMO	Uncharacterized protein OS=Bombyx mori OX=7091 GN=101736745 PE=4 SV=1	0	100	100	sp|P26305|LPSBP_PERAM	Hemolymph lipopolysaccharide-binding protein OS=Periplaneta americana OX=6978 PE=1 SV=1	3.37E-26	39.716	45.19230769
P_KWMTBOMO15675	tr|H9IYG1|H9IYG1_BOMMO	Uncharacterized protein OS=Bombyx mori OX=7091 PE=4 SV=1	0	100	56.60377358	sp|Q2M296|MTHSD_HUMAN	Methenyltetrahydrofolate synthase domain-containing protein OS=Homo sapiens OX=9606 GN=MTHFSD PE=1 SV=2	5.55E-81	40.212	79.24528302
P_KWMTBOMO15712	tr|Q2F5N9|Q2F5N9_BOMMO	Nucleoplasmin isoform 2 OS=Bombyx mori OX=7091 GN=692956 PE=2 SV=1	7.05E-137	100	100	sp|Q27415|NLP_DROME	Nucleoplasmin-like protein OS=Drosophila melanogaster OX=7227 GN=Nlp PE=1 SV=1	2.34E-29	48.148	57.7540107
P_KWMTBOMO15757	tr|B3VBE3|B3VBE3_BOMMO	Vacuolar proton pump subunit B OS=Bombyx mori OX=7091 PE=2 SV=1	5.34E-157	100	100	sp|P31401|VATB_MANSE	V-type proton ATPase subunit B OS=Manduca sexta OX=7130 GN=VHA55 PE=2 SV=1	2.90E-155	99.526	100
P_KWMTBOMO15769	tr|H9IYK6|H9IYK6_BOMMO	Uncharacterized protein OS=Bombyx mori OX=7091 GN=101741848 PE=4 SV=1	0	100	100	sp|Q76MZ3|2AAA_MOUSE	Serine/threonine-protein phosphatase 2A 65 kDa regulatory subunit A alpha isoform OS=Mus musculus OX=10090 GN=Ppp2r1a PE=1 SV=3	0	76.581	99.15254237
P_KWMTBOMO15781	tr|H9IYL2|H9IYL2_BOMMO	Uncharacterized protein OS=Bombyx mori OX=7091 PE=3 SV=2	0	87.5	100	sp|O46036|CTBP_DROME	C-terminal-binding protein OS=Drosophila melanogaster OX=7227 GN=CtBP PE=1 SV=3	0	91.643	78.9010989
P_KWMTBOMO15875	tr|H9JUY0|H9JUY0_BOMMO	Uncharacterized protein OS=Bombyx mori OX=7091 PE=4 SV=1	2.28E-159	100	87.85714286	--	--	--	--	--
P_KWMTBOMO15928	tr|Q0N2S4|Q0N2S4_BOMMO	Polyadenylate-binding protein OS=Bombyx mori OX=7091 GN=PAbp PE=2 SV=1	0	99.502	98.52941176	sp|P11940|PABP1_HUMAN	Polyadenylate-binding protein 1 OS=Homo sapiens OX=9606 GN=PABPC1 PE=1 SV=2	0	68.454	100
P_KWMTBOMO16000	tr|A0A1Q0AL85|A0A1Q0AL85_BOMMO	Homeostasis protein OS=Bombyx mori OX=7091 GN=IHoP PE=2 SV=1	0	100	100	sp|Q61233|PLSL_MOUSE	Plastin-2 OS=Mus musculus OX=10090 GN=Lcp1 PE=1 SV=4	0	51.823	91.05339105
P_KWMTBOMO16126	tr|Q5R1P6|Q5R1P6_BOMMO	Heat shock protein hsp20.1 OS=Bombyx mori OX=7091 GN=Hsp20.1 PE=2 SV=1	3.12E-134	100	100	sp|P82147|L2EFL_DROME	Protein lethal(2)essential for life OS=Drosophila melanogaster OX=7227 GN=l(2)efl PE=1 SV=1	1.62E-53	56.129	87.07865169
P_KWMTBOMO16197	tr|Q1HPX4|Q1HPX4_BOMMO	ATP synthase subunit gamma OS=Bombyx mori OX=7091 GN=732965 PE=2 SV=1	0	100	100	sp|O01666|ATPG_DROME	ATP synthase subunit gamma, mitochondrial OS=Drosophila melanogaster OX=7227 GN=ATPsyngamma PE=2 SV=2	2.51E-151	73.05	95.27027027
P_KWMTBOMO16204	tr|H9JWQ7|H9JWQ7_BOMMO	CHK domain-containing protein OS=Bombyx mori OX=7091 GN=101744771 PE=4 SV=1	0	100	100	--	--	--	--	--
P_KWMTBOMO16261	tr|Q2F6A0|Q2F6A0_BOMMO	Exuperantia OS=Bombyx mori OX=7091 PE=2 SV=1	0	100	100	sp|Q24747|EXU_DROVI	Maternal protein exuperantia OS=Drosophila virilis OX=7244 GN=exu PE=3 SV=1	5.40E-82	39.231	94.43099274
P_KWMTBOMO16319	tr|Q2F5K7|Q2F5K7_BOMMO	Receptor expression-enhancing protein OS=Bombyx mori OX=7091 PE=2 SV=1	3.16E-130	99.429	100	sp|Q29RM3|REEP5_BOVIN	Receptor expression-enhancing protein 5 OS=Bos taurus OX=9913 GN=REEP5 PE=2 SV=1	3.07E-59	50.595	96
P_KWMTBOMO16481	tr|Q19KB8|Q19KB8_BOMMO	Ras-related GTP-binding protein Rab11 OS=Bombyx mori OX=7091 GN=733063 PE=2 SV=1	1.33E-160	100	100	sp|P62492|RB11A_MOUSE	Ras-related protein Rab-11A OS=Mus musculus OX=10090 GN=Rab11a PE=1 SV=3	7.02E-129	82.326	100

Supplementary Table S4. Common PIPs shared by the three fibroin genes.

common PIPs at M4

Name	Bombyx-mori	Subject ID	Description	e-value	identity(%)	coverage(%)	Metazoa	Subject ID	Description	e-value	identity(%)	coverage(%)
P_KWMTBOMO00339		tr|H9ITE6|H9ITE6_BOMMO	CSD_1 domain-containing protein OS=Bombyx mori OX=7091 PE=4 SV=1	0	100	100		sp|P41824|YBOXH_APLCA	Y-box factor homolog OS=Aplysia californica OX=6500 PE=2 SV=1	1.24E-50	69.106	47.49034749
P_KWMTBOMO00366		tr|H9ITD6|H9ITD6_BOMMO	Uncharacterized protein OS=Bombyx mori OX=7091 PE=4 SV=1	0	99.775	83.80414313		sp|O42254|IF2B1_CHICK	Insulin-like growth factor 2 mRNA-binding protein 1 OS=Gallus gallus OX=9031 GN=IGF2BP1 PE=1 SV=1	7.53E-128	44.578	93.78531073
P_KWMTBOMO00437		tr|H9IU13|H9IU13_BOMMO	PHB domain-containing protein OS=Bombyx mori OX=7091 PE=3 SV=1	0	100	98.82629108		sp|O61491|FLOT1_DROME	Flotillin-1 OS=Drosophila melanogaster OX=7227 GN=Flo1 PE=2 SV=1	0	83.059	99.76525822
P_KWMTBOMO00462		tr|D9N4J4|D9N4J4_BOMMO	Staphylococcal nuclease domain-containing protein OS=Bombyx mori OX=7091 GN=Tudor-SN PE=2 SV=1	0	99.887	100		sp|Q9W0S7|SND1_DROME	Staphylococcal nuclease domain-containing protein 1 OS=Drosophila melanogaster OX=7227 GN=Tudor-SN PE=1 SV=1	0	52.412	100
P_KWMTBOMO00497		tr|H9JRZ9|H9JRZ9_BOMMO	Uncharacterized protein OS=Bombyx mori OX=7091 PE=3 SV=1	0	100	100		sp|Q60HD7|SERA_MACFA	D-3-phosphoglycerate dehydrogenase OS=Macaca fascicularis OX=9541 GN=PHGDH PE=2 SV=4	2.42E-109	50.814	93.88379205
P_KWMTBOMO00638		tr|D2Y4R4|D2Y4R4_BOMMO	Coatomer subunit beta' OS=Bombyx mori OX=7091 GN=COPB2 PE=2 SV=1	0	99.877	99.26918392		sp|O62621|COPB2_DROME	Coatomer subunit beta' OS=Drosophila melanogaster OX=7227 GN=beta'COP PE=2 SV=2	0	71.059	98.90377588
P_KWMTBOMO00687		tr|H9J134|H9J134_BOMMO	Nucleolar GTP-binding protein 1 OS=Bombyx mori OX=7091 PE=4 SV=1	0	93.029	64.59627329		sp|Q9V411|NOG1_DROME	Nucleolar GTP-binding protein 1 OS=Drosophila melanogaster OX=7227 GN=Non1 PE=2 SV=1	0	68.666	100
P_KWMTBOMO00786		tr|H9JJK3|H9JJK3_BOMMO	HIT domain-containing protein OS=Bombyx mori OX=7091 GN=693069 PE=4 SV=1	6.16E-93	100	100		sp|P62958|HINT1_BOVIN	Histidine triad nucleotide-binding protein 1 OS=Bos taurus OX=9913 GN=HINT1 PE=1 SV=2	1.81E-62	68.75	100
P_KWMTBOMO00881		tr|D4QF47|D4QF47_BOMMO	Ced-6 protein OS=Bombyx mori OX=7091 GN=ced-6 PE=2 SV=1	0	100	100		sp|Q7JUY7|CED6_DROME	PTB domain-containing adapter protein ced-6 OS=Drosophila melanogaster OX=7227 GN=ced-6 PE=1 SV=1	3.82E-84	39.463	94.34697856
P_KWMTBOMO00927		tr|H9JJU3|H9JJU3_BOMMO	Aspartate--tRNA ligase, cytoplasmic OS=Bombyx mori OX=7091 PE=3 SV=1	0	100	71.22040073		sp|Q3SYZ4|SYDC_BOVIN	Aspartate--tRNA ligase, cytoplasmic OS=Bos taurus OX=9913 GN=DARS1 PE=2 SV=1	0	64.329	90.89253188
P_KWMTBOMO01075		tr|H9JCV2|H9JCV2_BOMMO	Uncharacterized protein OS=Bombyx mori OX=7091 GN=101741707 PE=3 SV=1	0	99.629	100		sp|O02649|CH60A_DROME	Heat shock protein 60A OS=Drosophila melanogaster OX=7227 GN=Hsp60A PE=1 SV=3	0	84.72	95.91836735
P_KWMTBOMO01273		tr|Q5UAS5|Q5UAS5_BOMMO	60S ribosomal protein L13a OS=Bombyx mori OX=7091 GN=RpL13A PE=2 SV=1	3.08E-151	100	100		sp|Q8MUR4|RL13A_CHOPR	60S ribosomal protein L13a OS=Choristoneura parallela OX=106495 GN=RpL13A PE=2 SV=1	3.13E-140	91.667	100
P_KWMTBOMO01606		tr|H9J993|H9J993_BOMMO	Nop domain-containing protein OS=Bombyx mori OX=7091 GN=101742458 PE=3 SV=1	0	99.436	100		sp|Q9Y2X3|NOP58_HUMAN	Nucleolar protein 58 OS=Homo sapiens OX=9606 GN=NOP58 PE=1 SV=1	0	63.377	86.20037807
P_KWMTBOMO01720		tr|H9J930|H9J930_BOMMO	Uncharacterized protein OS=Bombyx mori OX=7091 PE=4 SV=1	0	98.191	100		sp|Q26457|LA_AEDAL	La protein homolog OS=Aedes albopictus OX=7160 PE=1 SV=1	2.92E-91	44.225	93.42105263
P_KWMTBOMO01790		tr|H9J907|H9J907_BOMMO	TPR_REGION domain-containing protein OS=Bombyx mori OX=7091 PE=4 SV=1	0	99.758	78.05108798		sp|Q9VGU5|TTC14_DROME	Tetratricopeptide repeat protein 14 homolog OS=Drosophila melanogaster OX=7227 GN=CG6621 PE=1 SV=2	1.20E-82	48.657	31.69347209
P_KWMTBOMO01918		tr|Q2F5L5|Q2F5L5_BOMMO	Protein transport protein Sec61 subunit beta OS=Bombyx mori OX=7091 GN=733070 PE=2 SV=1	1.66E-65	100	100		sp|P60467|SC61B_CANLF	Protein transport protein Sec61 subunit beta OS=Canis lupus familiaris OX=9615 GN=SEC61B PE=1 SV=2	2.67E-41	71.277	96.90721649
P_KWMTBOMO01927		sp|P21894|SYAC_BOMMO	Alanine--tRNA ligase, cytoplasmic OS=Bombyx mori OX=7091 PE=1 SV=1	0	99.276	100		sp|P21894|SYAC_BOMMO	Alanine--tRNA ligase, cytoplasmic OS=Bombyx mori OX=7091 PE=1 SV=1	0	99.276	100
P_KWMTBOMO02059		tr|Q5UAM4|Q5UAM4_BOMMO	40S ribosomal protein S18 OS=Bombyx mori OX=7091 GN=RpS18 PE=2 SV=1	1.29E-111	100	100		sp|Q962R1|RS18_SPOFR	40S ribosomal protein S18 OS=Spodoptera frugiperda OX=7108 GN=RpS18 PE=2 SV=1	1.23E-110	99.342	100
P_KWMTBOMO02081		tr|Q1HPW2|Q1HPW2_BOMMO	RNA helicase OS=Bombyx mori OX=7091 GN=692781 PE=2 SV=1	0	100	100		sp|Q02748|IF4A_DROME	Eukaryotic initiation factor 4A OS=Drosophila melanogaster OX=7227 GN=eIF4A PE=1 SV=3	0	79.949	93.80952381
P_KWMTBOMO02431		tr|Q1HQ25|Q1HQ25_BOMMO	ATP synthase subunit OS=Bombyx mori OX=7091 GN=732934 PE=2 SV=1	2.04E-70	100	100		sp|Q5RFH0|ATP5L_PONAB	ATP synthase subunit g, mitochondrial OS=Pongo abelii OX=9601 GN=ATP5MG PE=3 SV=1	1.02E-32	52.632	95.95959596
P_KWMTBOMO02432		tr|H9JVD5|H9JVD5_BOMMO	Dolichyl-diphosphooligosaccharide--protein glycosyltransferase subunit 2 OS=Bombyx mori OX=7091 PE=3 SV=1	0	100	100		sp|Q3SZI6|RPN2_BOVIN	Dolichyl-diphosphooligosaccharide--protein glycosyltransferase subunit 2 OS=Bos taurus OX=9913 GN=RPN2 PE=2 SV=1	1.44E-58	28.155	99.35691318
P_KWMTBOMO02470		tr|H9IZ85|H9IZ85_BOMMO	Dolichyl-diphosphooligosaccharide--protein glycosyltransferase subunit 1 OS=Bombyx mori OX=7091 PE=3 SV=1	0	100	98.93162393		sp|E2RQ08|RPN1_CANLF	Dolichyl-diphosphooligosaccharide--protein glycosyltransferase subunit 1 OS=Canis lupus familiaris OX=9615 GN=RPN1 PE=1 SV=1	6.16E-166	53.811	92.52136752
P_KWMTBOMO02748		tr|H9J2I1|H9J2I1_BOMMO	TLC domain-containing protein OS=Bombyx mori OX=7091 GN=101745731 PE=3 SV=1	0	100	100		sp|Q6DED0|TR1L1_XENLA	Translocating chain-associated membrane protein 1-like 1 OS=Xenopus laevis OX=8355 GN=tram1l1 PE=2 SV=1	1.09E-74	35.979	100

P_KWMTBOMO02835	tr|H9J2D4|H9J2D4_BOMMO	RPN13_C domain-containing protein OS=Bombyx mori OX=7091 PE=3 SV=1	0	97.136	100	sp|Q6NZ09|ADRM1_DANRE	Proteasomal ubiquitin receptor ADRM1 OS=Danio rerio OX=7955 GN=adrm1b PE=1 SV=1	1.84E-92	45.524	96.06879607
P_KWMTBOMO02994	tr|H9J281|H9J281_BOMMO	DNA photolyase OS=Bombyx mori OX=7091 PE=3 SV=1	0	100	96.04519774	sp|Q28811|PHR_POTTR	Deoxyribodipyrimidine photo-lyase OS=Potorous tridactylus OX=9310 GN=PHR PE=2 SV=1	0	59.205	90.01883239
P_KWMTBOMO03067	tr|H9J8B0|H9J8B0_BOMMO	Tripeptidyl-peptidase 2 OS=Bombyx mori OX=7091 GN=101742969 PE=4 SV=1	0	100	100	sp|P29144|TPP2_HUMAN	Tripeptidyl-peptidase 2 OS=Homo sapiens OX=9606 GN=TPP2 PE=1 SV=4	0	41.24	100
P_KWMTBOMO03173	tr|Q1HPV7|Q1HPV7_BOMMO	Histone H2A OS=Bombyx mori OX=7091 GN=h2a.z PE=2 SV=1	1.20E-88	100	100	sp|Q6Y237|H2AV_PAGMA	Histone H2A.V OS=Pagrus major OX=143350 GN=h2az2 PE=2 SV=3	3.37E-81	97.561	95.34883721
P_KWMTBOMO03177	tr|H9JV99|H9JV99_BOMMO	BRO1 domain-containing protein OS=Bombyx mori OX=7091 PE=4 SV=1	0	97.718	100	sp|Q9QZA2|PDC6I_RAT	Programmed cell death 6-interacting protein OS=Rattus norvegicus OX=10116 GN=Pdcd6ip PE=1 SV=2	0	43.273	98.36289222
P_KWMTBOMO03314	tr|Q5UAS3|Q5UAS3_BOMMO	Ribosomal protein L15 OS=Bombyx mori OX=7091 GN=RpL15 PE=2 SV=1	2.87E-148	100	100	sp|P30736|RL15_CHITE	60S ribosomal protein L15 OS=Chironomus tentans OX=7153 GN=RpL15 PE=3 SV=3	3.13E-122	80.392	100
P_KWMTBOMO03626	tr|H9J9S0|H9J9S0_BOMMO	CCT-alpha OS=Bombyx mori OX=7091 GN=101742382 PE=3 SV=1	0	100	100	sp|P12613|TCPA_DROME	T-complex protein 1 subunit alpha OS=Drosophila melanogaster OX=7227 GN=CCT1 PE=2 SV=2	0	80.576	100
P_KWMTBOMO03748	tr|Q2F645|Q2F645_BOMMO	Transketolase OS=Bombyx mori OX=7091 PE=2 SV=1	0	100	100	sp|Q9D4D4|TKTL2_MOUSE	Transketolase-like protein 2 OS=Mus musculus OX=10090 GN=Tktl2 PE=1 SV=1	0	62.153	98.55305466
P_KWMTBOMO03860	tr|H9JKX7|H9JKX7_BOMMO	Uncharacterized protein OS=Bombyx mori OX=7091 PE=3 SV=1	0	97.603	100	sp|P26686|SRR55_DROME	Serine-arginine protein 55 OS=Drosophila melanogaster OX=7227 GN=B52 PE=1 SV=4	6.44E-97	77.487	66.78321678
P_KWMTBOMO04370	tr|H9J6V7|H9J6V7_BOMMO	OBG-type G domain-containing protein OS=Bombyx mori OX=7091 PE=4 SV=1	2.07E-172	99.156	64.57765668	sp|P32234|128UP_DROME	GTP-binding protein 128up OS=Drosophila melanogaster OX=7227 GN=128up PE=2 SV=2	0	89.946	100
P_KWMTBOMO04502	tr|Q5TLD3|Q5TLD3_BOMMO	Replication protein A subunit OS=Bombyx mori OX=7091 GN=BmRPA1 PE=2 SV=1	0	99.498	100	sp|Q24492|RFA1_DROME	Replication protein A 70 kDa DNA-binding subunit OS=Drosophila melanogaster OX=7227 GN=RpA-70 PE=1 SV=1	0	47.421	100
P_KWMTBOMO04505	tr|H9J7F7|H9J7F7_BOMMO	Zinc-hook domain-containing protein OS=Bombyx mori OX=7091 PE=3 SV=1	0	99.3	85.54319932	sp|Q9W252|RAD50_DROME	DNA repair protein RAD50 OS=Drosophila melanogaster OX=7227 GN=rad50 PE=2 SV=4	1.59E-155	31.037	100
P_KWMTBOMO04605	tr|H9J7K1|H9J7K1_BOMMO	2-phospho-D-glycerate hydro-lyase OS=Bombyx mori OX=7091 PE=3 SV=1	0	100	100	sp|P15007|ENO_DROME	Enolase OS=Drosophila melanogaster OX=7227 GN=Eno PE=1 SV=2	0	81.481	99.76905312
P_KWMTBOMO04610	tr|H9J723|H9J723_BOMMO	26S proteasome non-ATPase regulatory subunit 2 OS=Bombyx mori OX=7091 PE=3 SV=1	0	97.303	100	sp|Q5R9I6|PSMD2_PONAB	26S proteasome non-ATPase regulatory subunit 2 OS=Pongo abelii OX=9601 GN=PSMD2 PE=2 SV=1	0	55.556	98.05825243
P_KWMTBOMO04806	tr|H9JK60|H9JK60_BOMMO	Ubiquitin-activating enzyme E1 OS=Bombyx mori OX=7091 GN=101739389 PE=3 SV=1	0	99.904	100	sp|Q5U300|UBA1_RAT	Ubiquitin-like modifier-activating enzyme 1 OS=Rattus norvegicus OX=10116 GN=Uba1 PE=1 SV=1	0	65.034	97.8906999
P_KWMTBOMO05541	tr|C7AQP4|C7AQP4_BOMMO	Calcium-transporting ATPase OS=Bombyx mori OX=7091 PE=2 SV=1	0	99.301	100	sp|Q7PPA5|ATC1_ANOGA	Calcium-transporting ATPase sarcoplasmic/endoplasmic reticulum type OS=Anopheles gambiae OX=7165 GN=SERCA PE=3 SV=5	0	89.279	99.6007984
P_KWMTBOMO05564	tr|Q2F6A6|Q2F6A6_BOMMO	Elongation factor Tu OS=Bombyx mori OX=7091 PE=2 SV=1	0	100	100	sp|P49411|EFTU_HUMAN	Elongation factor Tu, mitochondrial OS=Homo sapiens OX=9606 GN=TUFM PE=1 SV=2	0	65.672	86.4516129
P_KWMTBOMO05566	tr|Q2F5M1|Q2F5M1_BOMMO	GTP-binding nuclear protein OS=Bombyx mori OX=7091 GN=692970 PE=2 SV=1	1.62E-162	100	100	sp|Q9VZ23|RAN_DROME	GTP-binding nuclear protein Ran OS=Drosophila melanogaster OX=7227 GN=Ran PE=1 SV=1	2.67E-147	91.429	98.5915493
P_KWMTBOMO05612	tr|H9JB77|H9JB77_BOMMO	SAM_MT_RSMB_NOP domain-containing protein OS=Bombyx mori OX=7091 GN=101741314 PE=3 SV=1	0	99.874	100	sp|Q922K7|NOP2_MOUSE	Probable 28S rRNA (cytosine-C(5))-methyltransferase OS=Mus musculus OX=10090 GN=Nop2 PE=1 SV=1	0	53.565	72.69279393
P_KWMTBOMO05675	tr|Q5UAR5|Q5UAR5_BOMMO	Ribosomal protein L23A OS=Bombyx mori OX=7091 GN=RpL23A PE=2 SV=1	0	100	100	sp|P62752|RL23A_RAT	60S ribosomal protein L23a OS=Rattus norvegicus OX=10116 GN=Rpl23a PE=2 SV=1	8.65E-62	76.19	35.79545455
P_KWMTBOMO05741	tr|A1E129|A1E129_BOMMO	Kinesin-like protein OS=Bombyx mori OX=7091 PE=3 SV=2	0	99.17	100	sp|P17210|KINH_DROME	Kinesin heavy chain OS=Drosophila melanogaster OX=7227 GN=Khc PE=1 SV=2	0	74.871	100
P_KWMTBOMO05975	tr|H9J059|H9J059_BOMMO	Tr-type G domain-containing protein OS=Bombyx mori OX=7091 PE=4 SV=1	0	100	100	sp|Q5R8Q7|GTPB1_PONAB	GTP-binding protein 1 (Fragment) OS=Pongo abelii OX=9601 GN=GTPBP1 PE=2 SV=2	2.41E-105	74.641	75.72463768
P_KWMTBOMO05979	tr|Q5UAT0|Q5UAT0_BOMMO	Ribosomal protein L10 OS=Bombyx mori OX=7091 GN=RpL10 PE=2 SV=1	7.89E-166	100	100	sp|O96647|RL10_BOMMA	60S ribosomal protein L10 OS=Bombyx mandarina OX=7092 GN=RpL10 PE=2 SV=1	2.91E-164	99.543	100
P_KWMTBOMO06082	tr|H9IWS5|H9IWS5_BOMMO	Uncharacterized protein OS=Bombyx mori OX=7091 GN=101739385 PE=4 SV=1	0	100	100	--	--	--	--	--
P_KWMTBOMO06229	tr|H9IX17|H9IX17_BOMMO	Ribos_L4_asso_C domain-containing protein OS=Bombyx mori OX=7091 GN=692659 PE=3 SV=1	0	100	100	sp|P09180|RL4_DROME	60S ribosomal protein L4 OS=Drosophila melanogaster OX=7227 GN=RpL4 PE=1 SV=2	0	68.407	88.24884793
P_KWMTBOMO06231	tr|H9IX18|H9IX18_BOMMO	RuvB-like helicase OS=Bombyx mori OX=7091 PE=3 SV=1	1.12E-166	98.765	65.14745308	sp|Q9DE27|RUVB2_XENLA	RuvB-like 2 OS=Xenopus laevis OX=8355 GN=ruvbl2 PE=2 SV=1	0	81.501	100
P_KWMTBOMO06480	sp|Q5UAS1|RL18_BOMMO	60S ribosomal protein L18 OS=Bombyx mori OX=7091 GN=RpL18 PE=2 SV=1	1.41E-132	100	100	sp|Q5UAS1|RL18_BOMMO	60S ribosomal protein L18 OS=Bombyx mori OX=7091 GN=RpL18 PE=2 SV=1	9.16E-132	100	100
P_KWMTBOMO06632	tr|H9JQ89|H9JQ89_BOMMO	Nucleoprotein TPR OS=Bombyx mori OX=7091 PE=3 SV=1	0	87.867	58.41986456	sp|A1Z8P9|TPR_DROME	Nucleoprotein TPR OS=Drosophila melanogaster OX=7227 GN=Mtor PE=1 SV=1	0	31.858	100

P_KWMTBOMO06731	tr|H9JQE1|H9JQE1_BOMMO	Histone deacetylase OS=Bombyx mori OX=7091 PE=3 SV=1	0	91.686	89.64803313	sp|Q94517|HDAC1_DROME	Histone deacetylase HDAC1 OS=Drosophila melanogaster OX=7227 GN=HDAC1 PE=1 SV=2	0	92.634	92.75362319
P_KWMTBOMO06770	tr|H9JRG2|H9JRG2_BOMMO	Carboxylic ester hydrolase OS=Bombyx mori OX=7091 PE=3 SV=1	0	100	89.4934334	sp|P35502|ESTF_MYZPE	Esterase FE4 OS=Myzus persicae OX=13164 PE=1 SV=1	8.36E-86	35.577	97.56097561
P_KWMTBOMO06803	tr|H9JR86|H9JR86_BOMMO	CP-type G domain-containing protein OS=Bombyx mori OX=7091 GN=101742911 PE=4 SV=1	0	100	100	sp|Q9W590|LSG1_DROME	Large subunit GTPase 1 homolog OS=Drosophila melanogaster OX=7227 GN=Ns3 PE=1 SV=1	5.32E-148	39.605	100
P_KWMTBOMO06974	tr|Q5UAQ1|Q5UAQ1_BOMMO	Ribosomal protein L36A OS=Bombyx mori OX=7091 GN=RpL36A PE=2 SV=1	2.04E-71	100	100	sp|Q9NB33|RL44_OCHTR	60S ribosomal protein L44 OS=Ochlerotatus triseriatus OX=7162 GN=RpL44 PE=3 SV=3	5.04E-61	85.577	100
P_KWMTBOMO07076	tr|H9JLN4|H9JLN4_BOMMO	Uncharacterized protein OS=Bombyx mori OX=7091 PE=4 SV=1	0	99.76	80.03838772	sp|Q28EW0|TM87A_XENTR	Transmembrane protein 87A OS=Xenopus tropicalis OX=8364 GN=tmem87a PE=2 SV=1	5.77E-124	39.051	100
P_KWMTBOMO07274	tr|A0A4P9D450|A0A4P9D450_BOMMO	Piwi OS=Bombyx mori OX=7091 PE=2 SV=1	0	100	100	sp|A8D8P8|SIWI_BOMMO	Piwi-like protein Siwi OS=Bombyx mori OX=7091 GN=Siwi PE=1 SV=1	0	100	100
P_KWMTBOMO07373	tr|H9J6N5|H9J6N5_BOMMO	Uncharacterized protein OS=Bombyx mori OX=7091 PE=4 SV=1	4.18E-97	100	100	sp|Q9U1N0|HRP65_CHITE	Hrp65 protein OS=Chironomus tentans OX=7153 GN=HRP65 PE=1 SV=1	6.32E-24	60.227	61.97183099
P_KWMTBOMO07374	tr|Q0ZAL3|Q0ZAL3_BOMMO	Splicing factor proline-and glutamine-rich OS=Bombyx mori OX=7091 GN=SFPQ PE=2 SV=1	0	99.802	100	sp|Q9GRW7|NONA_DROVI	Protein no-on-transient A OS=Drosophila virilis OX=7244 GN=nonA PE=2 SV=1	5.73E-114	61.132	52.47524752
P_KWMTBOMO07589	tr|A0A023SG69|A0A023SG69_BOMMO	Coatomer subunit beta OS=Bombyx mori OX=7091 GN=beta-COP PE=4 SV=1	0	93.705	100	sp|Q5ZIA5|COPB_CHICK	Coatomer subunit beta OS=Gallus gallus OX=9031 GN=COPB1 PE=2 SV=1	0	57.39	100
P_KWMTBOMO07688	tr|Q5UAP3|Q5UAP3_BOMMO	40S ribosomal protein S2 OS=Bombyx mori OX=7091 GN=RpS2 PE=2 SV=1	0	100	100	sp|P31009|RS2_DROME	40S ribosomal protein S2 OS=Drosophila melanogaster OX=7227 GN=RpS2 PE=1 SV=2	4.18E-144	90.278	82.75862069
P_KWMTBOMO07780	tr|H9IUH3|H9IUH3_BOMMO	Eukaryotic translation initiation factor 3 subunit M OS=Bombyx mori OX=7091 GN=101737255 PE=3 SV=1	0	100	100	sp|Q17D30|EIF3M_AEDAE	Eukaryotic translation initiation factor 3 subunit M OS=Aedes aegypti OX=7159 GN=AAEL004347 PE=3 SV=1	0	72.798	100
P_KWMTBOMO07930	tr|Q1HQ48|Q1HQ48_BOMMO	Mitochondrial single-stranded DNA-binding protein OS=Bombyx mori OX=7091 PE=2 SV=1	1.39E-108	99.324	100	sp|P54622|SSBP_DROME	Single-stranded DNA-binding protein, mitochondrial OS=Drosophila melanogaster OX=7227 GN=mtSSB PE=1 SV=2	2.56E-50	64.545	74.32432432
P_KWMTBOMO08044	tr|H9IV44|H9IV44_BOMMO	DNA-(apurinic or apyrimidinic site) endonuclease OS=Bombyx mori OX=7091 GN=101739740 PE=3 SV=1	0	100	100	sp|P27864|RRP1_DROME	Recombination repair protein 1 OS=Drosophila melanogaster OX=7227 GN=Rrp1 PE=1 SV=2	6.85E-131	44.487	83.2278481
P_KWMTBOMO08100	tr|Q5UAR8|Q5UAR8_BOMMO	60S ribosomal protein L21 OS=Bombyx mori OX=7091 GN=RpL21 PE=2 SV=1	1.42E-115	100	100	sp|P46778|RL21_HUMAN	60S ribosomal protein L21 OS=Homo sapiens OX=9606 GN=RPL21 PE=1 SV=2	2.69E-77	66.875	100
P_KWMTBOMO08106	tr|H9IUY0|H9IUY0_BOMMO	Clathrin light chain OS=Bombyx mori OX=7091 PE=3 SV=1	2.92E-149	98.095	100	sp|Q9VWA1|CLC_DROME	Clathrin light chain OS=Drosophila melanogaster OX=7227 GN=Clc PE=2 SV=1	5.76E-40	46.948	100
P_KWMTBOMO08193	tr|H9JIX2|H9JIX2_BOMMO	Uncharacterized protein OS=Bombyx mori OX=7091 PE=3 SV=1	2.11E-91	98.571	100	sp|O95831|AIFM1_HUMAN	Apoptosis-inducing factor 1, mitochondrial OS=Homo sapiens OX=9606 GN=AIFM1 PE=1 SV=1	4.02E-30	42.958	100
P_KWMTBOMO08194	tr|H9JIX2|H9JIX2_BOMMO	Uncharacterized protein OS=Bombyx mori OX=7091 PE=3 SV=1	1.35E-72	66.522	52.15419501	sp|Q9VQ79|AIFM1_DROME	Putative apoptosis-inducing factor 1, mitochondrial OS=Drosophila melanogaster OX=7227 GN=AIF PE=2 SV=2	5.12E-60	35.423	72.33560091
P_KWMTBOMO08213	tr|H9JIW2|H9JIW2_BOMMO	Uncharacterized protein OS=Bombyx mori OX=7091 GN=101746972 PE=4 SV=1	0	100	100	sp|P51610|HCFC1_HUMAN	Host cell factor 1 OS=Homo sapiens OX=9606 GN=HCFC1 PE=1 SV=2	0	71.99	45.58472554
P_KWMTBOMO08310	tr|Q1HQB9|Q1HQB9_BOMMO	Nucleosome assembly protein isoform 1 OS=Bombyx mori OX=7091 PE=2 SV=1	0	100	100	sp|Q5U2Z3|NP1L4_RAT	Nucleosome assembly protein 1-like 4 OS=Rattus norvegicus OX=10116 GN=Nap1l4 PE=1 SV=1	4.23E-97	49.462	94.17721519
P_KWMTBOMO08607	tr|H9JEL8|H9JEL8_BOMMO	Uncharacterized protein OS=Bombyx mori OX=7091 PE=4 SV=1	2.44E-142	98.974	54.31754875	sp|Q5M9F1|RBM34_RAT	RNA-binding protein 34 OS=Rattus norvegicus OX=10116 GN=Rbm34 PE=1 SV=1	8.14E-07	26.087	64.06685237
P_KWMTBOMO08642	tr|F8UN44|F8UN44_BOMMO	Heat shock protein 70-3 OS=Bombyx mori OX=7091 PE=2 SV=1	0	100	100	sp|P29844|BIP_DROME	Endoplasmic reticulum chaperone BiP OS=Drosophila melanogaster OX=7227 GN=Hsc70-3 PE=1 SV=2	0	89.708	99.38931298
P_KWMTBOMO08648	tr|H9JEK1|H9JEK1_BOMMO	Uncharacterized protein OS=Bombyx mori OX=7091 PE=3 SV=1	0	91.125	100	sp|P13395|SPTCA_DROME	Spectrin alpha chain OS=Drosophila melanogaster OX=7227 GN=alpha-Spec PE=1 SV=2	0	76.124	100
P_KWMTBOMO08652	tr|H9JDP0|H9JDP0_BOMMO	Diadenosine tetraphosphate synthetase OS=Bombyx mori OX=7091 PE=3 SV=1	0	100	100	sp|Q04451|SYG_BOMMO	Glycine--tRNA ligase OS=Bombyx mori OX=7091 PE=1 SV=2	0	99.118	100
P_KWMTBOMO08666	tr|Q66SV4|Q66SV4_BOMMO	40S ribosomal protein S23 OS=Bombyx mori OX=7091 GN=S23 PE=2 SV=1	5.48E-103	100	100	sp|Q6EV23|RS23_PAPDA	40S ribosomal protein S23 OS=Papilio dardanus OX=77259 GN=RpS23 PE=2 SV=1	6.27E-101	97.902	100
P_KWMTBOMO08776	sp|Q1HPW4|EIF3I_BOMMO	Eukaryotic translation initiation factor 3 subunit I OS=Bombyx mori OX=7091 PE=2 SV=1	0	100	100	sp|Q1HPW4|EIF3I_BOMMO	Eukaryotic translation initiation factor 3 subunit I OS=Bombyx mori OX=7091 PE=2 SV=1	0	100	100
P_KWMTBOMO08820	tr|Q1HPI5|Q1HPI5_BOMMO	Nucleoside diphosphate kinase OS=Bombyx mori OX=7091 PE=2 SV=1	4.74E-113	100	100	sp|P08879|NDKA_DROME	Nucleoside diphosphate kinase OS=Drosophila melanogaster OX=7227 GN=awd PE=1 SV=3	5.17E-94	81.046	100

P_KWMTBOMO08956	tr|H9JDY1|H9JDY1_BOMMO	M20_dimer domain-containing protein OS=Bombyx mori OX=7091 PE=3 SV=1	0	99.784	96.86847599	sp|Q3ZC84|CNDP2_BOVIN	Cytosolic non-specific dipeptidase OS=Bos taurus OX=9913 GN=CNDP2 PE=2 SV=1	0	58.65	98.95615866
P_KWMTBOMO09054	tr|Q1HQ47|Q1HQ47_BOMMO	Isocitrate dehydrogenase [NADP] OS=Bombyx mori OX=7091 GN=100101164 PE=2 SV=1	0	100	100	sp|P33198|IDHP_PIG	Isocitrate dehydrogenase [NADP], mitochondrial (Fragment) OS=Sus scrofa OX=9823 GN=IDH2 PE=1 SV=1	0	71.325	95.40229885
P_KWMTBOMO09230	tr|H9J1M5|H9J1M5_BOMMO	Uncharacterized protein OS=Bombyx mori OX=7091 PE=3 SV=1	3.45E-75	100	100	sp|P08570|RLA1_DROME	60S acidic ribosomal protein P1 OS=Drosophila melanogaster OX=7227 GN=RpLP1 PE=1 SV=2	1.01E-42	81.25	100
P_KWMTBOMO09300	tr|Q5UAU1|Q5UAU1_BOMMO	60S acidic ribosomal protein P0 OS=Bombyx mori OX=7091 GN=RpP0 PE=1 SV=1	0	100	100	sp|Q9U3U0|RLA0_CERCA	60S acidic ribosomal protein P0 OS=Ceratitis capitata OX=7213 GN=RpLP0 PE=3 SV=1	1.73E-177	81.388	100
P_KWMTBOMO09415	tr|H9JTB0|H9JTB0_BOMMO	RNA helicase OS=Bombyx mori OX=7091 GN=101741115 PE=4 SV=1	0	99.765	100	sp|Q27268|DX39B_DROME	ATP-dependent RNA helicase WM6 OS=Drosophila melanogaster OX=7227 GN=Hel25E PE=1 SV=1	0	88.498	100
P_KWMTBOMO09442	tr|Q5UAT5|Q5UAT5_BOMMO	60S ribosomal protein L6 OS=Bombyx mori OX=7091 GN=RpL6 PE=2 SV=1	0	99.634	100	sp|Q6QMZ4|RL6_CHILA	60S ribosomal protein L6 OS=Chinchilla lanigera OX=34839 GN=RPL6 PE=2 SV=3	4.24E-65	48.8	91.57509158
P_KWMTBOMO09542	tr|H9JU13|H9JU13_BOMMO	Eukaryotic translation initiation factor 3 subunit A OS=Bombyx mori OX=7091 PE=3 SV=1	0	96.154	90.33898305	sp|Q173M7|EIF3A_AEDAE	Eukaryotic translation initiation factor 3 subunit A OS=Aedes aegypti OX=7159 GN=eIF3-S10 PE=3 SV=1	0	66.215	68.72881356
P_KWMTBOMO09591	tr|H9JTH6|H9JTH6_BOMMO	Uncharacterized protein OS=Bombyx mori OX=7091 PE=3 SV=1	9.34E-169	100	55.27522936	sp|O61492|FLOT2_DROME	Flotillin-2 OS=Drosophila melanogaster OX=7227 GN=Flo2 PE=2 SV=3	0	76.923	95.41284404
P_KWMTBOMO09621	tr|H9JTI9|H9JTI9_BOMMO	Eukaryotic translation initiation factor 3 subunit C OS=Bombyx mori OX=7091 GN=eIF3c PE=3 SV=1	0	100	100	sp|Q0ZB76|EIF3C_BOMMO	Eukaryotic translation initiation factor 3 subunit C OS=Bombyx mori OX=7091 GN=eIF3-S8 PE=2 SV=1	0	99.886	100
P_KWMTBOMO09747	tr|B7XFU6|B7XFU6_BOMMO	Clathrin heavy chain OS=Bombyx mori OX=7091 GN=100233163 PE=2 SV=1	0	99.941	100	sp|P29742|CLH_DROME	Clathrin heavy chain OS=Drosophila melanogaster OX=7227 GN=Chc PE=1 SV=1	0	85.196	100
P_KWMTBOMO09808	tr|Q2F6C3|Q2F6C3_BOMMO	Chaperonin subunit 6a zeta OS=Bombyx mori OX=7091 GN=692796 PE=2 SV=1	0	100	100	sp|P80317|TCPZ_MOUSE	T-complex protein 1 subunit zeta OS=Mus musculus OX=10090 GN=Cct6a PE=1 SV=3	0	72.505	100
P_KWMTBOMO10056	tr|B5M9A0|B5M9A0_BOMMO	Myosin heavy chain (Fragment) OS=Bombyx mori OX=7091 PE=2 SV=1	0	55.179	26.95431472	sp|Q99323|MYSN_DROME	Myosin heavy chain, non-muscle OS=Drosophila melanogaster OX=7227 GN=zip PE=1 SV=2	0	74.987	100
P_KWMTBOMO10248	tr|H9JCR4|H9JCR4_BOMMO	40S ribosomal protein SA OS=Bombyx mori OX=7091 PE=3 SV=1	0	100	100	sp|Q5UAP4|RSSA_BOMMO	40S ribosomal protein SA OS=Bombyx mori OX=7091 PE=2 SV=1	0	99.673	100
P_KWMTBOMO10358	tr|H9JX26|H9JX26_BOMMO	DUF4781 domain-containing protein OS=Bombyx mori OX=7091 PE=4 SV=1	0	79.827	48.32869081	--	--	--	--	--
P_KWMTBOMO10493	tr|H9JC69|H9JC69_BOMMO	Eukaryotic translation initiation factor 3 subunit B OS=Bombyx mori OX=7091 PE=3 SV=1	0	99.568	100	sp|Q1HDZ5|EIF3B_BOMMO	Eukaryotic translation initiation factor 3 subunit B OS=Bombyx mori OX=7091 GN=eIF3-S9 PE=2 SV=1	0	99.568	100
P_KWMTBOMO10780	tr|H9JFX9|H9JFX9_BOMMO	Uncharacterized protein OS=Bombyx mori OX=7091 PE=4 SV=1	0	90.138	43.81909548	sp|Q9V3P6|PSMD1_DROME	26S proteasome non-ATPase regulatory subunit 1 OS=Drosophila melanogaster OX=7227 GN=Rpn2 PE=1 SV=1	0	71.218	100
P_KWMTBOMO11045	tr|H9JRL1|H9JRL1_BOMMO	Uncharacterized protein OS=Bombyx mori OX=7091 PE=3 SV=1	0	28.363	91.51958674	sp|Q00963|SPTCB_DROME	Spectrin beta chain OS=Drosophila melanogaster OX=7227 GN=beta-Spec PE=1 SV=2	0	73.055	100
P_KWMTBOMO11061	tr|Q2F5N1|Q2F5N1_BOMMO	Prohibitin OS=Bombyx mori OX=7091 GN=693108 PE=2 SV=1	0	100	100	sp|Q5XIH7|PHB2_RAT	Prohibitin-2 OS=Rattus norvegicus OX=10116 GN=Phb2 PE=1 SV=1	5.66E-158	72.667	100
P_KWMTBOMO11083	tr|Q9BPS3|Q9BPS3_BOMMO	Elongation factor 1 gamma OS=Bombyx mori OX=7091 GN=ef-1g PE=2 SV=1	0	100	100	sp|P12261|EF1G_ARTSA	Elongation factor 1-gamma OS=Artemia salina OX=85549 PE=1 SV=3	0	65.741	100
P_KWMTBOMO11096	tr|A1E9B3|A1E9B3_BOMMO	H(+)-transporting two-sector ATPase OS=Bombyx mori OX=7091 PE=2 SV=1	0	99.676	100	sp|P31400|VATA_MANSE	V-type proton ATPase catalytic subunit A OS=Manduca sexta OX=7130 GN=VHAA PE=2 SV=1	0	98.217	100
P_KWMTBOMO11255	tr|Q5UAN5|Q5UAN5_BOMMO	40S ribosomal protein S9 OS=Bombyx mori OX=7091 GN=RpS9 PE=2 SV=1	5.62E-140	100	100	sp|B4PEU8|RS9_DROYA	40S ribosomal protein S9 OS=Drosophila yakuba OX=7245 GN=RpS9 PE=2 SV=1	2.83E-117	89.231	100
P_KWMTBOMO11448	tr|H9IXE5|H9IXE5_BOMMO	Uncharacterized protein OS=Bombyx mori OX=7091 GN=733057 PE=4 SV=1	1.36E-160	100	100	sp|P53994|RAB2A_MOUSE	Ras-related protein Rab-2A OS=Mus musculus OX=10090 GN=Rab2a PE=1 SV=1	2.20E-141	90.654	100
P_KWMTBOMO11517	tr|H9J3H3|H9J3H3_BOMMO	Uncharacterized protein OS=Bombyx mori OX=7091 PE=4 SV=1	0	96.387	98.84169884	--	--	--	--	--
P_KWMTBOMO11731	sp|Q1HPK6|EF2_BOMMO	Translation elongation factor 2 OS=Bombyx mori OX=7091 GN=tef2 PE=1 SV=1	0	100	100	sp|Q1HPK6|EF2_BOMMO	Translation elongation factor 2 OS=Bombyx mori OX=7091 GN=tef2 PE=1 SV=1	0	100	100
P_KWMTBOMO11985	tr|Q5UAT4|Q5UAT4_BOMMO	Ribosomal protein L7 OS=Bombyx mori OX=7091 GN=RpL7 PE=2 SV=1	0	100	100	sp|P32100|RL7_DROME	60S ribosomal protein L7 OS=Drosophila melanogaster OX=7227 GN=RpL7 PE=1 SV=2	4.63E-117	66.667	92.56505576
P_KWMTBOMO12037	tr|H9J470|H9J470_BOMMO	DEK_C domain-containing protein OS=Bombyx mori OX=7091 PE=4 SV=1	0	91.221	94.15322581	sp|Q6AXS3|DEK_RAT	Protein DEK OS=Rattus norvegicus OX=10116 GN=Dek PE=1 SV=1	5.05E-20	47.368	19.15322581
P_KWMTBOMO12165	tr|H9JWZ8|H9JWZ8_BOMMO	Glutathione synthase OS=Bombyx mori OX=7091 PE=3 SV=1	1.95E-169	99.565	52.99539171	sp|P35668|GSHB_XENLA	Glutathione synthetase OS=Xenopus laevis OX=8355 GN=gss PE=2 SV=1	2.49E-108	43.564	93.0875576
P_KWMTBOMO12166	tr|H9JWZ4|H9JWZ4_BOMMO	NADH dehydrogenase [ubiquinone] 1 alpha subcomplex subunit 10, mitochondrial	0	100	100	sp|P91929|NDUAA_DROME	NADH dehydrogenase [ubiquinone] 1 alpha subcomplex subunit 10, mitochondrial OS=Drosophila melanogaster OX=7227 GN=ND-42 PE=1 SV=2	1.80E-134	49.485	97.48743719

		OS=Bombyx mori OX=7091 GN=101745480 PE=3 SV=1								
P_KWMTBOMO12264	tr|H9JHZ0|H9JHZ0_BOMMO	CYTOSOL_AP domain-containing protein OS=Bombyx mori OX=7091 PE=3 SV=1	0	93.99	79.54110899	sp|Q5R7G6|PEPL1_PONAB	Probable aminopeptidase NPEPL1 OS=Pongo abelii OX=9601 GN=NPEPL1 PE=3 SV=2	0	54.981	99.80879541
P_KWMTBOMO12389	tr|H9JSC6|H9JSC6_BOMMO	Coatomer subunit epsilon OS=Bombyx mori OX=7091 PE=3 SV=1	0	100	100	sp|Q5ZIK9|COPE_CHICK	Coatomer subunit epsilon OS=Gallus gallus OX=9031 GN=COPE PE=2 SV=1	6.42E-96	48.475	97.68211921
P_KWMTBOMO12659	tr|H9IVX5|H9IVX5_BOMMO	T-complex protein 1 subunit gamma OS=Bombyx mori OX=7091 GN=101744569 PE=3 SV=1	0	100	100	sp|P48605|TCPG_DROME	T-complex protein 1 subunit gamma OS=Drosophila melanogaster OX=7227 GN=CCT3 PE=2 SV=2	0	81.041	99.62962963
P_KWMTBOMO12667	tr|Q1HPM7|Q1HPM7_BOMMO	Mitochondrial aldehyde dehydrogenase OS=Bombyx mori OX=7091 PE=2 SV=1	0	97.291	47.22814499	sp|P81178|ALDH2_MESAU	Aldehyde dehydrogenase, mitochondrial OS=Mesocricetus auratus OX=10036 GN=ALDH2 PE=1 SV=1	0	70	45.84221748
P_KWMTBOMO12844	tr|H9U396|H9U396_BOMMO	Endoplasmin-like protein OS=Bombyx mori OX=7091 PE=2 SV=1	0	100	100	sp|Q66HD0|ENPL_RAT	Endoplasmin OS=Rattus norvegicus OX=10116 GN=Hsp90b1 PE=1 SV=2	0	59.264	93.95061728
P_KWMTBOMO12895	tr|Q19AA9|Q19AA9_BOMMO	Transport protein Sec61 alpha subunit OS=Bombyx mori OX=7091 GN=733068 PE=2 SV=1	0	100	100	sp|Q9JLR1|S61A2_MOUSE	Protein transport protein Sec61 subunit alpha isoform 2 OS=Mus musculus OX=10090 GN=Sec61a2 PE=2 SV=3	0	91.597	100
P_KWMTBOMO13293	tr|E9JEI6|E9JEI6_BOMMO	Poly [ADP-ribose] polymerase (Fragment) OS=Bombyx mori OX=7091 PE=2 SV=1	0	99.894	94.5	sp|Q11208|PARP_SARPE	Poly [ADP-ribose] polymerase OS=Sarcophaga peregrina OX=7386 PE=1 SV=1	0	48.473	100
P_KWMTBOMO13405	tr|H9JN03|H9JN03_BOMMO	Proliferating cell nuclear antigen OS=Bombyx mori OX=7091 GN=692356 PE=3 SV=1	0	100	100	sp|O01377|PCNA_BOMMO	Proliferating cell nuclear antigen OS=Bombyx mori OX=7091 GN=PCNA PE=2 SV=1	0	98.846	100
P_KWMTBOMO13455	sp|Q5UAP0|RS4_BOMMO	40S ribosomal protein S4 OS=Bombyx mori OX=7091 GN=RpS4 PE=2 SV=1	0	100	100	sp|Q5UAP0|RS4_BOMMO	40S ribosomal protein S4 OS=Bombyx mori OX=7091 GN=RpS4 PE=2 SV=1	0	100	100
P_KWMTBOMO13567	tr|H9JN68|H9JN68_BOMMO	Uncharacterized protein OS=Bombyx mori OX=7091 PE=4 SV=1	0	100	61.18326118	--	--	--	--	--
P_KWMTBOMO13569	tr|Q5UAR3|Q5UAR3_BOMMO	Ribosomal protein L26 OS=Bombyx mori OX=7091 GN=RpL26 PE=2 SV=1	7.52E-72	100	100	sp|Q95WA0|RL26_LITLI	60S ribosomal protein L26 OS=Littorina littorea OX=31216 GN=RPL26 PE=2 SV=1	1.90E-51	80.392	100
P_KWMTBOMO13960	tr|Q5UAN0|Q5UAN0_BOMMO	40S ribosomal protein S13 OS=Bombyx mori OX=7091 GN=RpS13 PE=2 SV=1	8.93E-110	100	100	sp|Q962R6|RS13_SPOFR	40S ribosomal protein S13 OS=Spodoptera frugiperda OX=7108 GN=RpS13 PE=2 SV=3	1.63E-108	99.338	100
P_KWMTBOMO13997	tr|Q6T9Z7|Q6T9Z7_BOMMO	Fibroinase OS=Bombyx mori OX=7091 GN=693031 PE=2 SV=1	0	100	100	sp|Q26636|CATL_SARPE	Cathepsin L OS=Sarcophaga peregrina OX=7386 PE=1 SV=1	6.18E-177	68.902	96.18768328
P_KWMTBOMO14420	tr|H9IS77|H9IS77_BOMMO	Uncharacterized protein OS=Bombyx mori OX=7091 GN=101744691 PE=4 SV=1	0	99.786	99.78609626	sp|Q9BUJ2|HNRL1_HUMAN	Heterogeneous nuclear ribonucleoprotein U-like protein 1 OS=Homo sapiens OX=9606 GN=HNRNPUL1 PE=1 SV=2	7.54E-52	41.87	26.31016043
P_KWMTBOMO14440	tr|Q1HPP5|Q1HPP5_BOMMO	Actin-depolymerizing factor 1 OS=Bombyx mori OX=7091 PE=2 SV=1	1.70E-108	100	100	sp|P45594|CADF_DROME	Cofilin/actin-depolymerizing factor homolog OS=Drosophila melanogaster OX=7227 GN=tsr PE=1 SV=1	2.22E-100	92.568	100
P_KWMTBOMO14479	tr|H9IS48|H9IS48_BOMMO	Uncharacterized protein OS=Bombyx mori OX=7091 GN=101742289 PE=3 SV=1	7.91E-100	100	100	sp|Q8I1F4|FBRL_DROER	rRNA 2'-O-methyltransferase fibrillarin OS=Drosophila erecta OX=7220 GN=Fib PE=3 SV=1	2.11E-41	82.278	50
P_KWMTBOMO14549	tr|I6XKQ0|I6XKQ0_BOMMO	Heat shock protein 70-5 OS=Bombyx mori OX=7091 PE=2 SV=1	0	99.565	100	sp|P29845|HSP7E_DROME	Heat shock 70 kDa protein cognate 5 OS=Drosophila melanogaster OX=7227 GN=Hsc70-5 PE=1 SV=2	0	81.86	93.47826087
P_KWMTBOMO14732	tr|Q86PG2|Q86PG2_BOMMO	ADP/ATP translocase OS=Bombyx mori OX=7091 GN=ANT PE=2 SV=1	0	100	100	sp|Q26365|ADT_DROME	ADP,ATP carrier protein OS=Drosophila melanogaster OX=7227 GN=sesB PE=2 SV=4	0	87.458	98.33333333
P_KWMTBOMO14752	tr|H9J2U6|H9J2U6_BOMMO	PUA domain-containing protein OS=Bombyx mori OX=7091 GN=101744752 PE=3 SV=1	0	100	100	sp|O44081|DKC1_DROME	H/ACA ribonucleoprotein complex subunit 4 OS=Drosophila melanogaster OX=7227 GN=Nop60B PE=1 SV=1	0	76.69	91.47121535
P_KWMTBOMO14804	tr|H9JRQ1|H9JRQ1_BOMMO	Programmed cell death protein 4 OS=Bombyx mori OX=7091 GN=101743674 PE=3 SV=1	0	100	59.14221219	sp|Q98TX3|PDCD4_CHICK	Programmed cell death protein 4 OS=Gallus gallus OX=9031 GN=PDCD4 PE=2 SV=1	3.96E-92	43.371	100
P_KWMTBOMO14858	tr|H9JRJ9|H9JRJ9_BOMMO	Uncharacterized protein OS=Bombyx mori OX=7091 PE=4 SV=2	0	89.299	80.63814358	sp|Q02645|HTS_DROME	Protein hu-li tai shao OS=Drosophila melanogaster OX=7227 GN=hts PE=1 SV=2	0	56.932	49.16606236
P_KWMTBOMO14984	tr|H9J5J2|H9J5J2_BOMMO	AAA domain-containing protein OS=Bombyx mori OX=7091 PE=3 SV=1	0	100	100	sp|O42587|PR6AA_XENLA	26S proteasome regulatory subunit 6A-A OS=Xenopus laevis OX=8355 GN=psmc3-a PE=2 SV=2	0	88.124	98.36448598
P_KWMTBOMO15114	tr|Q5UAL8|Q5UAL8_BOMMO	40S ribosomal protein S25 OS=Bombyx mori OX=7091 GN=RpS25 PE=2 SV=1	7.13E-83	100	100	sp|Q962Q5|RS25_SPOFR	40S ribosomal protein S25 OS=Spodoptera frugiperda OX=7108 GN=RpS25 PE=3 SV=1	1.98E-81	99.16	100
P_KWMTBOMO15123	tr|H9J5W8|H9J5W8_BOMMO	AAA domain-containing protein OS=Bombyx mori OX=7091 PE=3 SV=2	0	99.241	90.38901602	sp|P48601|PRS4_DROME	26S proteasome regulatory subunit 4 OS=Drosophila melanogaster OX=7227 GN=Rpt2 PE=1 SV=2	0	96.811	100
P_KWMTBOMO15159	tr|H9J5Z1|H9J5Z1_BOMMO	Uncharacterized protein OS=Bombyx mori OX=7091 PE=4 SV=1	0	97.685	99.34282585	sp|Q99PL5|RRBP1_MOUSE	Ribosome-binding protein 1 OS=Mus musculus OX=10090 GN=Rrbp1 PE=1 SV=2	3.36E-17	27.524	67.25082147
P_KWMTBOMO15359	tr|H9J6H4|H9J6H4_BOMMO	Glutamyl-tRNA synthetase OS=Bombyx mori OX=7091 PE=3 SV=1	0	100	97.09944751	sp|P28668|SYEP_DROME	Bifunctional glutamate/proline--tRNA ligase OS=Drosophila melanogaster OX=7227 GN=GluProRS PE=1 SV=2	0	60.526	99.72375691

P_KWMTBOMO15758		tr|B3VBE3|B3VBE3_BOMMO	Vacuolar proton pump subunit B OS=Bombyx mori OX=7091 PE=2 SV=1	0	99.278	88.21656051		sp|P31401|VATB_MANSE	V-type proton ATPase subunit B OS=Manduca sexta OX=7130 GN=VHA55 PE=2 SV=1	0	97.112	88.21656051
P_KWMTBOMO16357		tr|Q5UAN3|Q5UAN3_BOMMO	40S ribosomal protein S11 OS=Bombyx mori OX=7091 GN=RpS11-1 PE=2 SV=1	6.00E-114	100	100		sp|P62280|RS11_HUMAN	40S ribosomal protein S11 OS=Homo sapiens OX=9606 GN=RPS11 PE=1 SV=3	4.63E-88	77.848	100
P_KWMTBOMO16524		tr|H9J7Z3|H9J7Z3_BOMMO	RRM domain-containing protein OS=Bombyx mori OX=7091 PE=3 SV=1	1.00E-90	76.064	100		sp|Q5R1W5|SRSF2_PANTR	Serine/arginine-rich splicing factor 2 OS=Pan troglodytes OX=9598 GN=SRSF2 PE=2 SV=3	1.89E-49	77.083	62.33766234
P_KWMTBOMO16637		tr|Q9GSB6|Q9GSB6_BOMMO	Heat shock protein hsp20.4 OS=Bombyx mori OX=7091 GN=Hsp20.4 PE=2 SV=2	5.98E-133	100	100		sp|P82147|L2EFL_DROME	Protein lethal(2)essential for life OS=Drosophila melanogaster OX=7227 GN=l(2)efl PE=1 SV=1	8.44E-55	50.568	97.23756906

common PIPs at L5D5

Name	Bombyx-mori	Subject ID	Description	e-value	identity(%)	coverage(%)	Metazoa	Subject ID	Description	e-value	identity(%)	coverage(%)
P_KWMTBOMO00303		tr|H9ITG0|H9ITG0_BOMMO	Rab-GAP TBC domain-containing protein OS=Bombyx mori OX=7091 PE=4 SV=1	0	98.655	99.87789988		sp|Q9VYY9|EVI5_DROME	Ecotropic viral integration site 5 ortholog OS=Drosophila melanogaster OX=7227 GN=Evi5 PE=1 SV=3	0	59.115	93.77289377
P_KWMTBOMO00375		tr|H9ITY5|H9ITY5_BOMMO	Uncharacterized protein OS=Bombyx mori OX=7091 PE=3 SV=1	0	87.972	100		sp|Q9VSA3|ACADM_DROME	Probable medium-chain specific acyl-CoA dehydrogenase, mitochondrial OS=Drosophila melanogaster OX=7227 GN=CG12262 PE=2 SV=1	0	73.558	98.34515366
P_KWMTBOMO00390		tr|H9ITC4|H9ITC4_BOMMO	Uncharacterized protein OS=Bombyx mori OX=7091 PE=4 SV=1	0	100	79.5620438		--	--	--	--	--
P_KWMTBOMO00427		tr|Q9NKX3|Q9NKX3_BOMMO	Coatomer subunit gamma OS=Bombyx mori OX=7091 GN=copg1 PE=2 SV=1	0	99.884	100		sp|Q8I0G5|COPG_DROME	Coatomer subunit gamma OS=Drosophila melanogaster OX=7227 GN=gammaCOP PE=2 SV=1	0	57.555	100
P_KWMTBOMO00462		tr|D9N4J4|D9N4J4_BOMMO	Staphylococcal nuclease domain-containing protein OS=Bombyx mori OX=7091 GN=Tudor-SN PE=2 SV=1	0	99.887	100		sp|Q9W0S7|SND1_DROME	Staphylococcal nuclease domain-containing protein 1 OS=Drosophila melanogaster OX=7227 GN=Tudor-SN PE=1 SV=1	0	52.412	100
P_KWMTBOMO00473		tr|Q1HPX8|Q1HPX8_BOMMO	6-phosphogluconate dehydrogenase, decarboxylating OS=Bombyx mori OX=7091 PE=2 SV=1	0	100	100		sp|P52209|6PGD_HUMAN	6-phosphogluconate dehydrogenase, decarboxylating OS=Homo sapiens OX=9606 GN=PGD PE=1 SV=3	0	73.542	99.37888199
P_KWMTBOMO00722		tr|A0A0D6A6H2|A0A0D6A6H2_BOMMO	Methionine--tRNA ligase, cytoplasmic OS=Bombyx mori OX=7091 GN=BmMRS PE=2 SV=1	0	100	100		sp|Q6PF21|SYMC_XENLA	Methionine--tRNA ligase, cytoplasmic OS=Xenopus laevis OX=8355 GN=mars1 PE=2 SV=1	0	46.816	89.41058941
P_KWMTBOMO00793		tr|H9JJJ8|H9JJJ8_BOMMO	Uncharacterized protein OS=Bombyx mori OX=7091 GN=101743807 PE=3 SV=1	0	100	100		sp|Q68FQ0|TCPE_RAT	T-complex protein 1 subunit epsilon OS=Rattus norvegicus OX=10116 GN=Cct5 PE=1 SV=1	0	70.391	99.07749077
P_KWMTBOMO00794		tr|Q1HQ81|Q1HQ81_BOMMO	Nascent polypeptide associated complex protein alpha subunit OS=Bombyx mori OX=7091 PE=2 SV=1	1.91E-153	100	100		sp|Q94518|NACA_DROME	Nascent polypeptide-associated complex subunit alpha OS=Drosophila melanogaster OX=7227 GN=Nacalpha PE=1 SV=2	1.16E-78	65.455	100
P_KWMTBOMO00837		tr|H9JJI3|H9JJI3_BOMMO	Proteasome subunit beta OS=Bombyx mori OX=7091 PE=3 SV=1	0	100	97.32824427		sp|P99026|PSB4_MOUSE	Proteasome subunit beta type-4 OS=Mus musculus OX=10090 GN=Psmb4 PE=1 SV=1	1.89E-88	49.799	95.03816794
P_KWMTBOMO00838		tr|Q5UAR6|Q5UAR6_BOMMO	60S ribosomal protein L23 OS=Bombyx mori OX=7091 GN=RpL23 PE=2 SV=1	5.38E-99	100	100		sp|P48159|RL23_DROME	60S ribosomal protein L23 OS=Drosophila melanogaster OX=7227 GN=RpL23 PE=1 SV=2	5.37E-94	95	100
P_KWMTBOMO00860		tr|D2Y4R7|D2Y4R7_BOMMO	Coatomer subunit zeta OS=Bombyx mori OX=7091 GN=COPZ PE=2 SV=1	1.18E-132	100	100		sp|P35604|COPZ1_BOVIN	Coatomer subunit zeta-1 OS=Bos taurus OX=9913 GN=COPZ1 PE=1 SV=2	2.88E-83	62.921	100
P_KWMTBOMO00927		tr|H9JJU3|H9JJU3_BOMMO	Aspartate--tRNA ligase, cytoplasmic OS=Bombyx mori OX=7091 PE=3 SV=1	0	100	71.22040073		sp|Q3SYZ4|SYDC_BOVIN	Aspartate--tRNA ligase, cytoplasmic OS=Bos taurus OX=9913 GN=DARS1 PE=2 SV=1	0	64.329	90.89253188
P_KWMTBOMO00982		tr|H9IVS2|H9IVS2_BOMMO	Uncharacterized protein OS=Bombyx mori OX=7091 PE=4 SV=1	9.80E-180	100	48.71287129		sp|Q9D0F3|LMAN1_MOUSE	Protein ERGIC-53 OS=Mus musculus OX=10090 GN=Lman1 PE=1 SV=1	1.46E-138	44.353	96.43564356
P_KWMTBOMO01055		tr|Q5UAL7|Q5UAL7_BOMMO	40S ribosomal protein S26 OS=Bombyx mori OX=7091 GN=RpS26 PE=2 SV=1	1.02E-81	100	87.12121212		sp|Q9GT45|RS26_ANOGA	40S ribosomal protein S26 OS=Anopheles gambiae OX=7165 GN=RpS26 PE=3 SV=2	5.07E-67	84.348	87.12121212
P_KWMTBOMO01072		tr|H9JD17|H9JD17_BOMMO	Uncharacterized protein OS=Bombyx mori OX=7091 PE=4 SV=1	0	99.688	40.02493766		sp|Q9W3N6|USO1_DROME	General vesicular transport factor p115 OS=Drosophila melanogaster OX=7227 GN=p115 PE=1 SV=2	0	50.619	100
P_KWMTBOMO01075		tr|H9JCV2|H9JCV2_BOMMO	Uncharacterized protein OS=Bombyx mori OX=7091 GN=101741707 PE=3 SV=1	0	99.629	100		sp|O02649|CH60A_DROME	Heat shock protein 60A OS=Drosophila melanogaster OX=7227 GN=Hsp60A PE=1 SV=3	0	84.72	95.91836735
P_KWMTBOMO01114		tr|H9JCT5|H9JCT5_BOMMO	26S proteasome regulatory subunit 7 OS=Bombyx mori OX=7091 GN=101744256 PE=3 SV=1	0	100	100		sp|P35998|PRS7_HUMAN	26S proteasome regulatory subunit 7 OS=Homo sapiens OX=9606 GN=PSMC2 PE=1 SV=3	0	91.014	100
P_KWMTBOMO01148		tr|Q2F5M4|Q2F5M4_BOMMO	Receptor for activated protein kinase C RACK isoform 1 OS=Bombyx mori OX=7091 PE=2 SV=1	0	100	100		sp|O18640|GBLP_DROME	Guanine nucleotide-binding protein subunit beta-like protein OS=Drosophila melanogaster OX=7227 GN=Rack1 PE=1 SV=2	0	86.52	100
P_KWMTBOMO01273		tr|Q5UAS5|Q5UAS5_BOMMO	60S ribosomal protein L13a OS=Bombyx mori OX=7091 GN=RpL13A PE=2 SV=1	3.08E-151	100	100		sp|Q8MUR4|RL13A_CHOPR	60S ribosomal protein L13a OS=Choristoneura parallela OX=106495 GN=RpL13A PE=2 SV=1	3.13E-140	91.667	100

P_KWMTBOMO01312	tr|Q2F5N7|Q2F5N7_BOMMO	26S proteasome regulatory subunit RPN11 OS=Bombyx mori OX=7091 PE=2 SV=1	0	100	100	sp|Q9V3H2|PSDE_DROME	26S proteasome non-ATPase regulatory subunit 14 OS=Drosophila melanogaster OX=7227 GN=Rpn11 PE=1 SV=1	0	92.926	100
P_KWMTBOMO01315	tr|Q1HPJ9|Q1HPJ9_BOMMO	V-type proton ATPase subunit H OS=Bombyx mori OX=7091 PE=2 SV=1	0	99.789	100	sp|Q9U5N0|VATH_MANSE	V-type proton ATPase subunit H OS=Manduca sexta OX=7130 PE=2 SV=1	0	96.421	100
P_KWMTBOMO01423	tr|H9JHG0|H9JHG0_BOMMO	NADH-ubiquinone oxidoreductase 75 kDa subunit, mitochondrial OS=Bombyx mori OX=7091 PE=3 SV=1	0	98.518	100	sp|Q94511|NDUS1_DROME	NADH-ubiquinone oxidoreductase 75 kDa subunit, mitochondrial OS=Drosophila melanogaster OX=7227 GN=ND-75 PE=2 SV=3	0	64.393	100
P_KWMTBOMO01424	tr|H9JHC4|H9JHC4_BOMMO	Elongation factor 1-beta' OS=Bombyx mori OX=7091 PE=3 SV=1	5.51E-165	100	100	sp|P29522|EF1B2_BOMMO	Elongation factor 1-beta' OS=Bombyx mori OX=7091 PE=1 SV=2	3.22E-163	99.55	100
P_KWMTBOMO01431	tr|H9JHF8|H9JHF8_BOMMO	Voltage-dependent anion-selective channel protein 3 OS=Bombyx mori OX=7091 GN=101739703 PE=3 SV=1	5.89E-131	100	100	sp|Q94920|VDAC_DROME	Voltage-dependent anion-selective channel OS=Drosophila melanogaster OX=7227 GN=porin PE=1 SV=3	8.71E-82	60.656	100
P_KWMTBOMO01452	tr|H9JHD3|H9JHD3_BOMMO	Arginyl-tRNA synthetase OS=Bombyx mori OX=7091 GN=101737924 PE=3 SV=1	0	100	100	sp|Q6P1S4|SYRC_XENTR	Arginine--tRNA ligase, cytoplasmic OS=Xenopus tropicalis OX=8364 GN=rars1 PE=2 SV=1	0	58.85	99.26793558
P_KWMTBOMO01606	tr|H9J993|H9J993_BOMMO	Nop domain-containing protein OS=Bombyx mori OX=7091 GN=101742458 PE=3 SV=1	0	99.436	100	sp|Q9Y2X3|NOP58_HUMAN	Nucleolar protein 58 OS=Homo sapiens OX=9606 GN=NOP58 PE=1 SV=1	0	63.377	86.20037807
P_KWMTBOMO01708	tr|H9J9C9|H9J9C9_BOMMO	Dynein light intermediate chain OS=Bombyx mori OX=7091 PE=3 SV=1	2.61E-111	99.367	34.05172414	sp|Q6PDL0|DC1L2_MOUSE	Cytoplasmic dynein 1 light intermediate chain 2 OS=Mus musculus OX=10090 GN=Dync1li2 PE=1 SV=2	2.12E-142	50.33	98.06034483
P_KWMTBOMO01720	tr|H9J930|H9J930_BOMMO	Uncharacterized protein OS=Bombyx mori OX=7091 PE=4 SV=1	0	98.191	100	sp|Q26457|LA_AEDAL	La protein homolog OS=Aedes albopictus OX=7160 PE=1 SV=1	2.92E-91	44.225	93.42105263
P_KWMTBOMO01796	tr|Q2F5J2|Q2F5J2_BOMMO	Prohibitin OS=Bombyx mori OX=7091 GN=692987 PE=2 SV=1	0	100	100	sp|P24156|L2CC_DROME	Protein l(2)37Cc OS=Drosophila melanogaster OX=7227 GN=l(2)37Cc PE=2 SV=2	6.28E-169	83.088	99.27007299
P_KWMTBOMO01806	tr|H9J9G9|H9J9G9_BOMMO	Cation-transporting ATPase OS=Bombyx mori OX=7091 GN=101743411 PE=3 SV=1	0	99.914	100	sp|Q9EPE9|AT131_MOUSE	Manganese-transporting ATPase 13A1 OS=Mus musculus OX=10090 GN=Atp13a1 PE=1 SV=2	0	57.252	100
P_KWMTBOMO02081	tr|Q1HPW2|Q1HPW2_BOMMO	RNA helicase OS=Bombyx mori OX=7091 GN=692781 PE=2 SV=1	0	100	100	sp|Q02748|IF4A_DROME	Eukaryotic initiation factor 4A OS=Drosophila melanogaster OX=7227 GN=eIF4A PE=1 SV=3	0	79.949	93.80952381
P_KWMTBOMO02088	tr|H9J0E7|H9J0E7_BOMMO	Lysine--tRNA ligase OS=Bombyx mori OX=7091 GN=101740883 PE=3 SV=1	0	100	100	sp|Q99MN1|SYK_MOUSE	Lysine--tRNA ligase OS=Mus musculus OX=10090 GN=Kars1 PE=1 SV=1	0	68.206	97.06896552
P_KWMTBOMO02223	tr|Q1HQC7|Q1HQC7_BOMMO	Aspartate aminotransferase OS=Bombyx mori OX=7091 PE=2 SV=1	0	99.768	100	sp|P00507|AATM_RAT	Aspartate aminotransferase, mitochondrial OS=Rattus norvegicus OX=10116 GN=Got2 PE=1 SV=2	0	62.06	92.34338747
P_KWMTBOMO02278	sp|Q75VN3|TCTP_BOMMO	Translationally-controlled tumor protein homolog OS=Bombyx mori OX=7091 GN=Tctp PE=2 SV=1	8.85E-126	100	100	sp|Q75VN3|TCTP_BOMMO	Translationally-controlled tumor protein homolog OS=Bombyx mori OX=7091 GN=Tctp PE=2 SV=1	5.77E-125	100	100
P_KWMTBOMO02470	tr|H9IZ85|H9IZ85_BOMMO	Dolichyl-diphosphooligosaccharide--protein glycosyltransferase subunit 1 OS=Bombyx mori OX=7091 PE=3 SV=1	0	100	98.93162393	sp|E2RQ08|RPN1_CANLF	Dolichyl-diphosphooligosaccharide--protein glycosyltransferase subunit 1 OS=Canis lupus familiaris OX=9615 GN=RPN1 PE=1 SV=1	6.16E-166	53.811	92.52136752
P_KWMTBOMO02477	tr|Q5UAR9|Q5UAR9_BOMMO	Ribosomal protein L19 OS=Bombyx mori OX=7091 GN=RpL19 PE=2 SV=1	1.82E-145	100	100	sp|P36241|RL19_DROME	60S ribosomal protein L19 OS=Drosophila melanogaster OX=7227 GN=RpL19 PE=1 SV=2	4.16E-113	84.343	99
P_KWMTBOMO02500	tr|H9IZ97|H9IZ97_BOMMO	Uncharacterized protein OS=Bombyx mori OX=7091 PE=4 SV=1	0	90.773	100	sp|O75439|MPPB_HUMAN	Mitochondrial-processing peptidase subunit beta OS=Homo sapiens OX=9606 GN=PMPCB PE=1 SV=2	0	68	96.56652361
P_KWMTBOMO02535	tr|H9IZB1|H9IZB1_BOMMO	Uncharacterized protein OS=Bombyx mori OX=7091 PE=4 SV=1	0	96.875	70.12687428	sp|O08730|GLYG_RAT	Glycogenin-1 OS=Rattus norvegicus OX=10116 GN=Gyg1 PE=2 SV=4	3.47E-117	52.395	38.52364475
P_KWMTBOMO02542	tr|Q8T7L8|Q8T7L8_BOMMO	Seroin 1 OS=Bombyx mori OX=7091 PE=2 SV=1	7.09E-73	97.222	100	--	--	--	--	--
P_KWMTBOMO02734	tr|Q9BPS1|Q9BPS1_BOMMO	Elongation factor 1 delta OS=Bombyx mori OX=7091 GN=ef-1d PE=2 SV=1	9.74E-145	100	100	sp|P32192|EF1D_ARTSA	Elongation factor 1-delta OS=Artemia salina OX=85549 PE=1 SV=2	5.90E-72	62.632	93.1372549
P_KWMTBOMO02775	tr|H9J1V6|H9J1V6_BOMMO	Very-long-chain enoyl-CoA reductase OS=Bombyx mori OX=7091 GN=101742899 PE=3 SV=1	0	100	100	sp|Q9N5Y2|TECR_CAEEL	Probable very-long-chain enoyl-CoA reductase art-1 OS=Caenorhabditis elegans OX=6239 GN=art-1 PE=3 SV=1	6.15E-119	56.209	100
P_KWMTBOMO02835	tr|H9J2D4|H9J2D4_BOMMO	RPN13_C domain-containing protein OS=Bombyx mori OX=7091 PE=3 SV=1	0	97.136	100	sp|Q6NZ09|ADRM1_DANRE	Proteasomal ubiquitin receptor ADRM1 OS=Danio rerio OX=7955 GN=adrm1b PE=1 SV=1	1.84E-92	45.524	96.06879607
P_KWMTBOMO03003	tr|H9J272|H9J272_BOMMO	Adenylate kinase isoenzyme 6 homolog OS=Bombyx mori OX=7091 GN=101735934 PE=3 SV=1	2.60E-133	100	100	sp|Q7JYV7|KAD6_DROME	Adenylate kinase isoenzyme 6 homolog OS=Drosophila melanogaster OX=7227 GN=Ak6 PE=1 SV=1	3.13E-69	57.317	93.71428571
P_KWMTBOMO03027	tr|Q2F5M6|Q2F5M6_BOMMO	Ras small monomeric GTPase Rab6 OS=Bombyx mori OX=7091 PE=2 SV=1	1.13E-158	100	100	sp|O18334|RAB6_DROME	Ras-related protein Rab6 OS=Drosophila melanogaster OX=7227 GN=Rab6 PE=1 SV=1	2.75E-141	90.338	99.0430622
P_KWMTBOMO03067	tr|H9J8B0|H9J8B0_BOMMO	Tripeptidyl-peptidase 2 OS=Bombyx mori OX=7091 GN=101742969 PE=4 SV=1	0	100	100	sp|P29144|TPP2_HUMAN	Tripeptidyl-peptidase 2 OS=Homo sapiens OX=9606 GN=TPP2 PE=1 SV=4	0	41.24	100

P_KWMTBOMO03113	tr|Q2F5V9|Q2F5V9_BOMMO	Translocon-associated protein subunit beta OS=Bombyx mori OX=7091 PE=2 SV=1	1.01E-139	100	100	sp|P43308|SSRB_HUMAN	Translocon-associated protein subunit beta OS=Homo sapiens OX=9606 GN=SSR2 PE=1 SV=1	2.35E-60	52.809	93.68421053
P_KWMTBOMO03177	tr|H9JV99|H9JV99_BOMMO	BRO1 domain-containing protein OS=Bombyx mori OX=7091 PE=4 SV=1	0	97.718	100	sp|Q9QZA2|PDC6I_RAT	Programmed cell death 6-interacting protein OS=Rattus norvegicus OX=10116 GN=Pdcd6ip PE=1 SV=2	0	43.273	98.36289222
P_KWMTBOMO03301	tr|E0D4V7|E0D4V7_BOMMO	Phenylalanyl-tRNA synthetase beta subunit OS=Bombyx mori OX=7091 GN=FRSB PE=2 SV=1	0	100	100	sp|Q9VCA5|SYFB_DROME	Phenylalanine--tRNA ligase beta subunit OS=Drosophila melanogaster OX=7227 GN=beta-PheRS PE=1 SV=1	0	66.893	100
P_KWMTBOMO03430	tr|Q2F5R4|Q2F5R4_BOMMO	Glutamate dehydrogenase (NAD(P)(+)) OS=Bombyx mori OX=7091 GN=692939 PE=2 SV=1	0	100	74.38692098	sp|P54385|DHE3_DROME	Glutamate dehydrogenase, mitochondrial OS=Drosophila melanogaster OX=7227 GN=Gdh PE=1 SV=2	0	79.821	76.29427793
P_KWMTBOMO03626	tr|H9J9S0|H9J9S0_BOMMO	CCT-alpha OS=Bombyx mori OX=7091 GN=101742382 PE=3 SV=1	0	100	100	sp|P12613|TCPA_DROME	T-complex protein 1 subunit alpha OS=Drosophila melanogaster OX=7227 GN=CCT1 PE=2 SV=2	0	80.576	100
P_KWMTBOMO03722	tr|H9JKE1|H9JKE1_BOMMO	Dynamin-type G domain-containing protein OS=Bombyx mori OX=7091 PE=4 SV=1	0	100	76.69172932	sp|Q9H4M9|EHD1_HUMAN	EH domain-containing protein 1 OS=Homo sapiens OX=9606 GN=EHD1 PE=1 SV=2	0	69.101	100
P_KWMTBOMO03951	tr|Q5UAL6|Q5UAL6_BOMMO	40S ribosomal protein S27 OS=Bombyx mori OX=7091 GN=RpS27 PE=2 SV=1	1.35E-59	100	100	sp|P55833|RS27_HOMAM	40S ribosomal protein S27 OS=Homarus americanus OX=6706 GN=RPS27 PE=3 SV=2	1.42E-47	78.571	100
P_KWMTBOMO03986	tr|Q9NL60|Q9NL60_BOMMO	Annexin OS=Bombyx mori OX=7091 PE=2 SV=1	0	99.69	100	sp|P22464|ANXB9_DROME	Annexin B9 OS=Drosophila melanogaster OX=7227 GN=AnxB9 PE=2 SV=2	9.67E-180	76.012	99.38080495
P_KWMTBOMO04051	tr|H9JL88|H9JL88_BOMMO	UDPGT domain-containing protein OS=Bombyx mori OX=7091 PE=3 SV=1	0	95.709	99.9002991	sp|A0A291PQH4|UGT2_DACCO	UDP-glucosyltransferase 2 OS=Dactylopius coccus OX=765876 GN=UTG2 PE=1 SV=1	6.72E-74	31.836	51.04685942
P_KWMTBOMO04168	tr|H9JGM3|H9JGM3_BOMMO	Uncharacterized protein OS=Bombyx mori OX=7091 PE=3 SV=1	0	100	100	sp|Q94516|AT5F1_DROME	ATP synthase subunit b, mitochondrial OS=Drosophila melanogaster OX=7227 GN=ATPsynB PE=2 SV=2	1.44E-101	69.036	81.06995885
P_KWMTBOMO04170	tr|H9JGS8|H9JGS8_BOMMO	UTP--glucose-1-phosphate uridylyltransferase OS=Bombyx mori OX=7091 PE=3 SV=1	0	100	97.85575049	sp|Q07130|UGPA_BOVIN	UTP--glucose-1-phosphate uridylyltransferase OS=Bos taurus OX=9913 GN=UGP2 PE=1 SV=2	0	65.737	97.85575049
P_KWMTBOMO04330	tr|H9JX71|H9JX71_BOMMO	PCI domain-containing protein OS=Bombyx mori OX=7091 GN=101735320 PE=3 SV=1	0	100	100	sp|Q7KLV9|PSD11_DROME	26S proteasome non-ATPase regulatory subunit 11 OS=Drosophila melanogaster OX=7227 GN=Rpn6 PE=1 SV=1	0	76.54	100
P_KWMTBOMO04370	tr|H9J6V7|H9J6V7_BOMMO	OBG-type G domain-containing protein OS=Bombyx mori OX=7091 PE=4 SV=1	2.07E-172	99.156	64.57765668	sp|P32234|128UP_DROME	GTP-binding protein 128up OS=Drosophila melanogaster OX=7227 GN=128up PE=2 SV=2	0	89.946	100
P_KWMTBOMO04427	tr|H9J7B7|H9J7B7_BOMMO	Signal recognition particle receptor subunit beta OS=Bombyx mori OX=7091 PE=3 SV=1	0	100	100	sp|Q9Y5M8|SRPRB_HUMAN	Signal recognition particle receptor subunit beta OS=Homo sapiens OX=9606 GN=SRPRB PE=1 SV=3	2.92E-59	46.575	87.6
P_KWMTBOMO04502	tr|Q5TLD3|Q5TLD3_BOMMO	Replication protein A subunit OS=Bombyx mori OX=7091 GN=BmRPA1 PE=2 SV=1	0	99.498	100	sp|Q24492|RFA1_DROME	Replication protein A 70 kDa DNA-binding subunit OS=Drosophila melanogaster OX=7227 GN=RpA-70 PE=1 SV=1	0	47.421	100
P_KWMTBOMO04529	tr|Q2F6C7|Q2F6C7_BOMMO	ADP-ribosylation factor OS=Bombyx mori OX=7091 GN=778456 PE=2 SV=1	9.95E-136	100	100	sp|P61210|ARF1_LOCMI	ADP-ribosylation factor 1 OS=Locusta migratoria OX=7004 GN=ARF1 PE=2 SV=2	7.09E-134	98.901	100
P_KWMTBOMO04584	tr|Q2F5K1|Q2F5K1_BOMMO	Signal sequence receptor subunit gamma OS=Bombyx mori OX=7091 PE=2 SV=1	1.07E-132	100	100	sp|Q9UNL2|SSRG_HUMAN	Translocon-associated protein subunit gamma OS=Homo sapiens OX=9606 GN=SSR3 PE=1 SV=1	8.00E-87	64.865	100
P_KWMTBOMO04605	tr|H9J7K1|H9J7K1_BOMMO	2-phospho-D-glycerate hydro-lyase OS=Bombyx mori OX=7091 PE=3 SV=1	0	100	100	sp|P15007|ENO_DROME	Enolase OS=Drosophila melanogaster OX=7227 GN=Eno PE=1 SV=2	0	81.481	99.76905312
P_KWMTBOMO04610	tr|H9J723|H9J723_BOMMO	26S proteasome non-ATPase regulatory subunit 2 OS=Bombyx mori OX=7091 PE=3 SV=1	0	97.303	100	sp|Q5R9I6|PSMD2_PONAB	26S proteasome non-ATPase regulatory subunit 2 OS=Pongo abelii OX=9601 GN=PSMD2 PE=2 SV=1	0	55.556	98.05825243
P_KWMTBOMO04768	tr|H9JK00|H9JK00_BOMMO	Valyl-tRNA synthetase OS=Bombyx mori OX=7091 PE=3 SV=1	0	93.973	100	sp|Q04462|SYVC_RAT	Valine--tRNA ligase OS=Rattus norvegicus OX=10116 GN=Vars1 PE=2 SV=2	0	59.277	100
P_KWMTBOMO04789	tr|Q2F5P7|Q2F5P7_BOMMO	Mitochondrial matrix protein p32 OS=Bombyx mori OX=7091 PE=2 SV=1	0	100	100	sp|Q3T0B6|C1QBP_BOVIN	Complement component 1 Q subcomponent-binding protein, mitochondrial OS=Bos taurus OX=9913 GN=C1QBP PE=2 SV=1	7.30E-40	32.042	100
P_KWMTBOMO04806	tr|H9JK60|H9JK60_BOMMO	Ubiquitin-activating enzyme E1 OS=Bombyx mori OX=7091 GN=101739389 PE=3 SV=1	0	99.904	100	sp|Q5U300|UBA1_RAT	Ubiquitin-like modifier-activating enzyme 1 OS=Rattus norvegicus OX=10116 GN=Uba1 PE=1 SV=1	0	65.034	97.8906999
P_KWMTBOMO04906	tr|H9JEW9|H9JEW9_BOMMO	Aminopeptidase N-8 OS=Bombyx mori OX=7091 GN=101737565 PE=2 SV=1	0	98.024	92.42009132	sp|P15145|AMPN_PIG	Aminopeptidase N OS=Sus scrofa OX=9823 GN=ANPEP PE=1 SV=4	9.30E-128	32.057	76.34703196
P_KWMTBOMO04992	tr|H9JEZ8|H9JEZ8_BOMMO	Uncharacterized protein OS=Bombyx mori OX=7091 PE=3 SV=1	0	99.614	100	sp|Q21929|DCXR_CAEEL	L-xylulose reductase OS=Caenorhabditis elegans OX=6239 GN=dhs-21 PE=1 SV=2	5.68E-38	33.597	97.68339768
P_KWMTBOMO05178	tr|Q2F5M8|Q2F5M8_BOMMO	Phosphoserine aminotransferase OS=Bombyx mori OX=7091 PE=2 SV=1	0	100	100	sp|Q9VAN0|SERC_DROME	Probable phosphoserine aminotransferase OS=Drosophila melanogaster OX=7227 GN=CG11899 PE=2 SV=1	9.90E-147	64.935	96.85534591
P_KWMTBOMO05205	tr|H9IYX7|H9IYX7_BOMMO	5-aminoimidazole-4-carboxamide ribonucleotide formyltransferase OS=Bombyx mori OX=7091 PE=3 SV=1	0	100	98.81556684	sp|P31335|PUR9_CHICK	Bifunctional purine biosynthesis protein ATIC OS=Gallus gallus OX=9031 GN=ATIC PE=1 SV=1	0	68.855	100

P_KWMTBOMO05258	tr|H9IZ08|H9IZ08_BOMMO	Peptidase_M24 domain-containing protein OS=Bombyx mori OX=7091 GN=101746307 PE=3 SV=1	0	100	100	sp|Q6AYD3|PA2G4_RAT	Proliferation-associated protein 2G4 OS=Rattus norvegicus OX=10116 GN=Pa2g4 PE=1 SV=1	1.26E-149	57.778	94.98680739
P_KWMTBOMO05281	tr|H9IZ19|H9IZ19_BOMMO	Uncharacterized protein OS=Bombyx mori OX=7091 GN=101735890 PE=3 SV=1	4.73E-144	100	100	sp|Q9CQC9|SAR1B_MOUSE	GTP-binding protein SAR1b OS=Mus musculus OX=10090 GN=Sar1b PE=1 SV=1	9.93E-107	75	100
P_KWMTBOMO05299	tr|H9IYR7|H9IYR7_BOMMO	Uncharacterized protein OS=Bombyx mori OX=7091 PE=3 SV=1	0	96.091	98.08306709	sp|O95749|GGPPS_HUMAN	Geranylgeranyl pyrophosphate synthase OS=Homo sapiens OX=9606 GN=GGPS1 PE=1 SV=1	5.33E-131	60.357	89.45686901
P_KWMTBOMO05464	tr|H9JB09|H9JB09_BOMMO	Vasa intronic protein OS=Bombyx mori OX=7091 GN=101741313 PE=2 SV=1	0	100	100	sp|Q6AXS5|PAIRB_RAT	Plasminogen activator inhibitor 1 RNA-binding protein OS=Rattus norvegicus OX=10116 GN=Serbp1 PE=1 SV=2	1.23E-20	40.625	41.1311054
P_KWMTBOMO05541	tr|C7AQP4|C7AQP4_BOMMO	Calcium-transporting ATPase OS=Bombyx mori OX=7091 PE=2 SV=1	0	99.301	100	sp|Q7PPA5|ATC1_ANOGA	Calcium-transporting ATPase sarcoplasmic/endoplasmic reticulum type OS=Anopheles gambiae OX=7165 GN=SERCA PE=3 SV=5	0	89.279	99.6007984
P_KWMTBOMO05564	tr|Q2F6A6|Q2F6A6_BOMMO	Elongation factor Tu OS=Bombyx mori OX=7091 PE=2 SV=1	0	100	100	sp|P49411|EFTU_HUMAN	Elongation factor Tu, mitochondrial OS=Homo sapiens OX=9606 GN=TUFM PE=1 SV=2	0	65.672	86.4516129
P_KWMTBOMO05566	tr|Q2F5M1|Q2F5M1_BOMMO	GTP-binding nuclear protein OS=Bombyx mori OX=7091 GN=692970 PE=2 SV=1	1.62E-162	100	100	sp|Q9VZ23|RAN_DROME	GTP-binding nuclear protein Ran OS=Drosophila melanogaster OX=7227 GN=Ran PE=1 SV=1	2.67E-147	91.429	98.5915493
P_KWMTBOMO05612	tr|H9JB77|H9JB77_BOMMO	SAM_MT_RSMB_NOP domain-containing protein OS=Bombyx mori OX=7091 GN=101741314 PE=3 SV=1	0	99.874	100	sp|Q922K7|NOP2_MOUSE	Probable 28S rRNA (cytosine-C(5))-methyltransferase OS=Mus musculus OX=10090 GN=Nop2 PE=1 SV=1	0	53.565	72.69279393
P_KWMTBOMO05741	tr|A1E129|A1E129_BOMMO	Kinesin-like protein OS=Bombyx mori OX=7091 PE=3 SV=2	0	99.17	100	sp|P17210|KINH_DROME	Kinesin heavy chain OS=Drosophila melanogaster OX=7227 GN=Khc PE=1 SV=2	0	74.871	100
P_KWMTBOMO05810	tr|H9JBQ0|H9JBQ0_BOMMO	Uncharacterized protein OS=Bombyx mori OX=7091 PE=4 SV=1	0	99.381	50.35650624	sp|Q8SWU7|OLA1_DROME	Obg-like ATPase 1 OS=Drosophila melanogaster OX=7227 GN=CG1354 PE=1 SV=1	4.43E-180	78.274	14.97326203
P_KWMTBOMO05975	tr|H9J059|H9J059_BOMMO	Tr-type G domain-containing protein OS=Bombyx mori OX=7091 PE=4 SV=1	0	100	100	sp|Q5R8Q7|GTPB1_PONAB	GTP-binding protein 1 (Fragment) OS=Pongo abelii OX=9601 GN=GTPBP1 PE=2 SV=2	2.41E-105	74.641	75.72463768
P_KWMTBOMO06045	tr|H9J005|H9J005_BOMMO	TOG domain-containing protein OS=Bombyx mori OX=7091 PE=3 SV=2	0	93.348	100	sp|E9PVA8|GCN1_MOUSE	eIF-2-alpha kinase activator GCN1 OS=Mus musculus OX=10090 GN=Gcn1 PE=1 SV=1	0	45.751	100
P_KWMTBOMO06354	tr|H9JR23|H9JR23_BOMMO	Aminotran_1_2 domain-containing protein OS=Bombyx mori OX=7091 PE=4 SV=1	0	94.624	90.11627907	sp|Q28DB5|ALAT2_XENTR	Alanine aminotransferase 2 OS=Xenopus tropicalis OX=8364 GN=gpt2 PE=2 SV=1	0	58.078	94.76744186
P_KWMTBOMO06427	tr|Q2F5L0|Q2F5L0_BOMMO	Signal sequence receptor subunit alpha OS=Bombyx mori OX=7091 GN=778461 PE=2 SV=1	0	100	100	sp|P43307|SSRA_HUMAN	Translocon-associated protein subunit alpha OS=Homo sapiens OX=9606 GN=SSR1 PE=1 SV=3	6.17E-70	50.45	79.56989247
P_KWMTBOMO06480	sp|Q5UAS1|RL18_BOMMO	60S ribosomal protein L18 OS=Bombyx mori OX=7091 GN=RpL18 PE=2 SV=1	1.41E-132	100	100	sp|Q5UAS1|RL18_BOMMO	60S ribosomal protein L18 OS=Bombyx mori OX=7091 GN=RpL18 PE=2 SV=1	9.16E-132	100	100
P_KWMTBOMO06595	tr|I3VR81|I3VR81_BOMMO	Aminopeptidase OS=Bombyx mori OX=7091 PE=2 SV=1	0	100	100	sp|P55786|PSA_HUMAN	Puromycin-sensitive aminopeptidase OS=Homo sapiens OX=9606 GN=NPEPPS PE=1 SV=2	0	57.324	100
P_KWMTBOMO06660	tr|Q2F640|Q2F640_BOMMO	Ubiquinol-cytochrome c reductase core protein II OS=Bombyx mori OX=7091 GN=100127121 PE=2 SV=1	0	100	100	sp|P22695|QCR2_HUMAN	Cytochrome b-c1 complex subunit 2, mitochondrial OS=Homo sapiens OX=9606 GN=UQCRC2 PE=1 SV=3	1.22E-72	34.615	95.19450801
P_KWMTBOMO06763	tr|H9JR70|H9JR70_BOMMO	Uncharacterized protein OS=Bombyx mori OX=7091 PE=4 SV=1	0	99.694	100	--	--	--	--	--
P_KWMTBOMO06974	tr|Q5UAQ1|Q5UAQ1_BOMMO	Ribosomal protein L36A OS=Bombyx mori OX=7091 GN=RpL36A PE=2 SV=1	2.04E-71	100	100	sp|Q9NB33|RL44_OCHTR	60S ribosomal protein L44 OS=Ochlerotatus triseriatus OX=7162 GN=RpL44 PE=3 SV=3	5.04E-61	85.577	100
P_KWMTBOMO06999	tr|H9JLS3|H9JLS3_BOMMO	TPR_REGION domain-containing protein OS=Bombyx mori OX=7091 PE=3 SV=1	0	100	100	sp|P31948|STIP1_HUMAN	Stress-induced-phosphoprotein 1 OS=Homo sapiens OX=9606 GN=STIP1 PE=1 SV=1	0	52.007	100
P_KWMTBOMO07103	tr|Q2F5P8|Q2F5P8_BOMMO	Malate dehydrogenase OS=Bombyx mori OX=7091 PE=2 SV=1	0	100	100	sp|O88989|MDHC_RAT	Malate dehydrogenase, cytoplasmic OS=Rattus norvegicus OX=10116 GN=Mdh1 PE=1 SV=3	7.95E-157	65.653	99.39577039
P_KWMTBOMO07214	tr|D5MTP2|D5MTP2_BOMMO	Bm8 interacting protein 2-11 OS=Bombyx mori OX=7091 PE=2 SV=1	0	94.898	78.82037534	sp|Q9VMA7|TGO1_DROME	Transport and Golgi organization protein 1 OS=Drosophila melanogaster OX=7227 GN=Tango1 PE=1 SV=2	6.97E-56	30.097	48.32439678
P_KWMTBOMO07323	tr|Q2F6C4|Q2F6C4_BOMMO	T-complex protein 1 subunit delta OS=Bombyx mori OX=7091 PE=2 SV=1	0	99.814	100	sp|Q9NB32|TCPD_OCHTR	T-complex protein 1 subunit delta OS=Ochlerotatus triseriatus OX=7162 PE=2 SV=1	0	69.088	100
P_KWMTBOMO07368	tr|H9JMD1|H9JMD1_BOMMO	Uncharacterized protein OS=Bombyx mori OX=7091 GN=101744608 PE=3 SV=1	8.23E-94	100	100	sp|P24310|CX7A1_HUMAN	Cytochrome c oxidase subunit 7A1, mitochondrial OS=Homo sapiens OX=9606 GN=COX7A1 PE=1 SV=2	3.33E-12	47.458	45.38461538
P_KWMTBOMO07374	tr|Q0ZAL3|Q0ZAL3_BOMMO	Splicing factor proline-and glutamine-rich OS=Bombyx mori OX=7091 GN=SFPQ PE=2 SV=1	0	99.802	100	sp|Q9GRW7|NONA_DROVI	Protein no-on-transient A OS=Drosophila virilis OX=7244 GN=nonA PE=2 SV=1	5.73E-114	61.132	52.47524752

P_KWMTBOMO07589	tr|A0A023SG69|A0A023SG69_BOMMO	Coatomer subunit beta OS=Bombyx mori OX=7091 GN=beta-COP PE=4 SV=1	0	93.705	100	sp|Q5ZIA5|COPB_CHICK	Coatomer subunit beta OS=Gallus gallus OX=9031 GN=COPB1 PE=2 SV=1	0	57.39	100
P_KWMTBOMO07761	tr|H9IUG4|H9IUG4_BOMMO	Uncharacterized protein OS=Bombyx mori OX=7091 PE=4 SV=1	0	94.605	26.40722724	sp|Q8MSS1|LVA_DROME	Protein lava lamp OS=Drosophila melanogaster OX=7227 GN=lva PE=1 SV=2	5.58E-15	35.644	7.01876303
P_KWMTBOMO07772	tr|Q5UAN4|Q5UAN4_BOMMO	Ribosomal protein S10 OS=Bombyx mori OX=7091 GN=RpS10 PE=2 SV=1	4.40E-118	100	100	sp|Q962R9|RS10_SPOFR	40S ribosomal protein S10 OS=Spodoptera frugiperda OX=7108 GN=RpS10 PE=2 SV=1	1.19E-105	92.453	99.375
P_KWMTBOMO07855	tr|Q2F6C2|Q2F6C2_BOMMO	CCT-beta OS=Bombyx mori OX=7091 GN=692797 PE=2 SV=1	0	100	100	sp|Q3ZBH0|TCPB_BOVIN	T-complex protein 1 subunit beta OS=Bos taurus OX=9913 GN=CCT2 PE=1 SV=3	0	72.15	99.81343284
P_KWMTBOMO07857	tr|Q5R1P5|Q5R1P5_BOMMO	Heat shock protein hsp21.4 OS=Bombyx mori OX=7091 GN=Hsp21.4 PE=2 SV=1	4.58E-139	100	100	sp|Q00649|HSPB1_CHICK	Heat shock protein beta-1 OS=Gallus gallus OX=9031 GN=HSPB1 PE=2 SV=1	5.62E-19	47.727	47.05882353
P_KWMTBOMO08045	tr|H9IUV7|H9IUV7_BOMMO	Leucyl-tRNA synthetase OS=Bombyx mori OX=7091 PE=3 SV=1	0	99.658	99.20724802	sp|Q9P2J5|SYLC_HUMAN	Leucine--tRNA ligase, cytoplasmic OS=Homo sapiens OX=9606 GN=LARS1 PE=1 SV=2	0	65.984	99.54699887
P_KWMTBOMO08056	tr|H9IV39|H9IV39_BOMMO	Rab GDP dissociation inhibitor OS=Bombyx mori OX=7091 GN=101738804 PE=3 SV=1	0	100	100	sp|P21856|GDIA_BOVIN	Rab GDP dissociation inhibitor alpha OS=Bos taurus OX=9913 GN=GDI1 PE=1 SV=1	0	68.75	100
P_KWMTBOMO08064	tr|Q5UAR1|Q5UAR1_BOMMO	60S ribosomal protein L27a OS=Bombyx mori OX=7091 GN=RpL27A PE=2 SV=1	3.15E-107	100	100	sp|P47830|RL27A_XENLA	60S ribosomal protein L27a OS=Xenopus laevis OX=8355 GN=rpl27a PE=2 SV=2	1.19E-82	77.027	100
P_KWMTBOMO08098	tr|G9FL14|G9FL14_BOMMO	DNA supercoiling factor OS=Bombyx mori OX=7091 GN=LOC692759 PE=2 SV=1	1.98E-48	100	100	sp|B5X4E0|CALUB_SALSA	Calumenin-B OS=Salmo salar OX=8030 GN=calub PE=2 SV=1	7.32E-13	49.333	100
P_KWMTBOMO08106	tr|H9IUY0|H9IUY0_BOMMO	Clathrin light chain OS=Bombyx mori OX=7091 PE=3 SV=1	2.92E-149	98.095	100	sp|Q9VWA1|CLC_DROME	Clathrin light chain OS=Drosophila melanogaster OX=7227 GN=Clc PE=2 SV=1	5.76E-40	46.948	100
P_KWMTBOMO08204	tr|H9JIZ5|H9JIZ5_BOMMO	Arf-GAP domain-containing protein OS=Bombyx mori OX=7091 GN=101746692 PE=4 SV=1	0	100	100	sp|Q28CM8|ARFG2_XENTR	ADP-ribosylation factor GTPase-activating protein 2 OS=Xenopus tropicalis OX=8364 GN=arfgap2 PE=2 SV=1	1.27E-99	39.455	100
P_KWMTBOMO08310	tr|Q1HQB9|Q1HQB9_BOMMO	Nucleosome assembly protein isoform 1 OS=Bombyx mori OX=7091 PE=2 SV=1	0	100	100	sp|Q5U2Z3|NP1L4_RAT	Nucleosome assembly protein 1-like 4 OS=Rattus norvegicus OX=10116 GN=Nap1l4 PE=1 SV=1	4.23E-97	49.462	94.17721519
P_KWMTBOMO08323	tr|H9JI91|H9JI91_BOMMO	Uncharacterized protein OS=Bombyx mori OX=7091 PE=4 SV=1	0	99.643	98.24561404	sp|Q5R4J9|NAA15_PONAB	N-alpha-acetyltransferase 15, NatA auxiliary subunit OS=Pongo abelii OX=9601 GN=NAA15 PE=2 SV=1	4.44E-47	41.985	91.92982456
P_KWMTBOMO08343	tr|H9JI86|H9JI86_BOMMO	Uncharacterized protein OS=Bombyx mori OX=7091 PE=4 SV=1	0	100	71.51898734	sp|P54802|ANAG_HUMAN	Alpha-N-acetylglucosaminidase OS=Homo sapiens OX=9606 GN=NAGLU PE=1 SV=2	2.63E-165	41.81	67.61603376
P_KWMTBOMO08440	tr|Q5UAM6|Q5UAM6_BOMMO	Ribosomal protein S16 OS=Bombyx mori OX=7091 GN=RpS16 PE=2 SV=1	8.57E-109	100	100	sp|Q95V31|RS16_SPOFR	40S ribosomal protein S16 OS=Spodoptera frugiperda OX=7108 GN=RpS16 PE=2 SV=1	1.88E-104	98.013	100
P_KWMTBOMO08578	tr|H9JDK4|H9JDK4_BOMMO	Multifunctional fusion protein OS=Bombyx mori OX=7091 GN=101741966 PE=3 SV=1	0	100	100	sp|Q7SY23|AL4A1_DANRE	Delta-1-pyrroline-5-carboxylate dehydrogenase, mitochondrial OS=Danio rerio OX=7955 GN=aldh4a1 PE=2 SV=1	0	55.159	94.33628319
P_KWMTBOMO08596	tr|H9JEM4|H9JEM4_BOMMO	Uncharacterized protein OS=Bombyx mori OX=7091 PE=3 SV=1	1.43E-162	100	97.69585253	sp|Q7SXP0|S22BB_DANRE	Vesicle-trafficking protein SEC22b-B OS=Danio rerio OX=7955 GN=sec22bb PE=2 SV=1	4.44E-87	56.621	100
P_KWMTBOMO08607	tr|H9JEL8|H9JEL8_BOMMO	Uncharacterized protein OS=Bombyx mori OX=7091 PE=4 SV=1	2.44E-142	98.974	54.31754875	sp|Q5M9F1|RBM34_RAT	RNA-binding protein 34 OS=Rattus norvegicus OX=10116 GN=Rbm34 PE=1 SV=1	8.14E-07	26.087	64.06685237
P_KWMTBOMO08648	tr|H9JEK1|H9JEK1_BOMMO	Uncharacterized protein OS=Bombyx mori OX=7091 PE=3 SV=1	0	91.125	100	sp|P13395|SPTCA_DROME	Spectrin alpha chain OS=Drosophila melanogaster OX=7227 GN=alpha-Spec PE=1 SV=2	0	76.124	100
P_KWMTBOMO08666	tr|Q66SV4|Q66SV4_BOMMO	40S ribosomal protein S23 OS=Bombyx mori OX=7091 GN=S23 PE=2 SV=1	5.48E-103	100	100	sp|Q6EV23|RS23_PAPDA	40S ribosomal protein S23 OS=Papilio dardanus OX=77259 GN=RpS23 PE=2 SV=1	6.27E-101	97.902	100
P_KWMTBOMO08707	tr|H9JDR8|H9JDR8_BOMMO	Elongator complex protein 3 OS=Bombyx mori OX=7091 PE=3 SV=1	0	99.66	82.4684432	sp|Q5ZHS1|ELP3_CHICK	Elongator complex protein 3 OS=Gallus gallus OX=9031 GN=ELP3 PE=2 SV=1	0	84.962	74.61430575
P_KWMTBOMO08820	tr|Q1HPI5|Q1HPI5_BOMMO	Nucleoside diphosphate kinase OS=Bombyx mori OX=7091 PE=2 SV=1	4.74E-113	100	100	sp|P08879|NDKA_DROME	Nucleoside diphosphate kinase OS=Drosophila melanogaster OX=7227 GN=awd PE=1 SV=3	5.17E-94	81.046	100
P_KWMTBOMO08894	tr|Q5UAN9|Q5UAN9_BOMMO	Ribosomal protein S5 OS=Bombyx mori OX=7091 GN=RpS5 PE=2 SV=1	3.62E-166	100	100	sp|P46782|RS5_HUMAN	40S ribosomal protein S5 OS=Homo sapiens OX=9606 GN=RPS5 PE=1 SV=4	1.84E-134	91.327	89.49771689
P_KWMTBOMO08956	tr|H9JDY1|H9JDY1_BOMMO	M20_dimer domain-containing protein OS=Bombyx mori OX=7091 PE=3 SV=1	0	99.784	96.86847599	sp|Q3ZC84|CNDP2_BOVIN	Cytosolic non-specific dipeptidase OS=Bos taurus OX=9913 GN=CNDP2 PE=2 SV=1	0	58.65	98.95615866
P_KWMTBOMO08961	tr|Q5UAQ8|Q5UAQ8_BOMMO	60S ribosomal protein L30 OS=Bombyx mori OX=7091 GN=RpL30 PE=2 SV=1	2.87E-80	100	100	sp|P58375|RL30_SPOFR	60S ribosomal protein L30 OS=Spodoptera frugiperda OX=7108 GN=RpL30 PE=3 SV=1	9.75E-78	99.099	98.2300885
P_KWMTBOMO09055	tr|H9JDA5|H9JDA5_BOMMO	Proton-translocating NAD(P)(+) transhydrogenase OS=Bombyx mori OX=7091 PE=3 SV=1	0	93.082	100	sp|P11024|NNTM_BOVIN	NAD(P) transhydrogenase, mitochondrial OS=Bos taurus OX=9913 GN=NNT PE=1 SV=3	0	59.925	100
P_KWMTBOMO09183	tr|H9J1H4|H9J1H4_BOMMO	Uncharacterized protein OS=Bombyx mori OX=7091 PE=4 SV=1	0	99.804	46.6179159	sp|O00410|IPO5_HUMAN	Importin-5 OS=Homo sapiens OX=9606 GN=IPO5 PE=1 SV=4	0	52.516	99.90859232
P_KWMTBOMO09230	tr|H9J1M5|H9J1M5_BOMMO	Uncharacterized protein OS=Bombyx mori OX=7091 PE=3 SV=1	3.45E-75	100	100	sp|P08570|RLA1_DROME	60S acidic ribosomal protein P1 OS=Drosophila melanogaster OX=7227 GN=RpLP1 PE=1 SV=2	1.01E-42	81.25	100

P_KWMTBOMO09232	tr|Q5UAS9|Q5UAS9_BOMMO	Ribosomal protein L10A OS=Bombyx mori OX=7091 GN=RpL10A PE=2 SV=1	4.16E-162	100	100	sp|Q963B6|RL10A_SPOFR	60S ribosomal protein L10a OS=Spodoptera frugiperda OX=7108 GN=RpL10A PE=2 SV=1	1.14E-156	96.774	100
P_KWMTBOMO09355	tr|Q6T3A7|Q6T3A7_BOMMO	Thiol peroxiredoxin OS=Bombyx mori OX=7091 GN=692638 PE=2 SV=1	5.60E-148	100	100	sp|Q9V3P0|PRDX1_DROME	Peroxiredoxin 1 OS=Drosophila melanogaster OX=7227 GN=Jafrac1 PE=1 SV=1	8.32E-117	81.152	97.94871795
P_KWMTBOMO09415	tr|H9JTB0|H9JTB0_BOMMO	RNA helicase OS=Bombyx mori OX=7091 GN=101741115 PE=4 SV=1	0	99.765	100	sp|Q27268|DX39B_DROME	ATP-dependent RNA helicase WM6 OS=Drosophila melanogaster OX=7227 GN=Hel25E PE=1 SV=1	0	88.498	100
P_KWMTBOMO09442	tr|Q5UAT5|Q5UAT5_BOMMO	60S ribosomal protein L6 OS=Bombyx mori OX=7091 GN=RpL6 PE=2 SV=1	0	99.634	100	sp|Q6QMZ4|RL6_CHILA	60S ribosomal protein L6 OS=Chinchilla lanigera OX=34839 GN=RPL6 PE=2 SV=3	4.24E-65	48.8	91.57509158
P_KWMTBOMO09542	tr|H9JU13|H9JU13_BOMMO	Eukaryotic translation initiation factor 3 subunit A OS=Bombyx mori OX=7091 PE=3 SV=1	0	96.154	90.33898305	sp|Q173M7|EIF3A_AEDAE	Eukaryotic translation initiation factor 3 subunit A OS=Aedes aegypti OX=7159 GN=eIF3-S10 PE=3 SV=1	0	66.215	68.72881356
P_KWMTBOMO09553	tr|Q1HPN7|Q1HPN7_BOMMO	Fructose-bisphosphate aldolase OS=Bombyx mori OX=7091 GN=778467 PE=2 SV=1	0	100	100	sp|P07764|ALF_DROME	Fructose-bisphosphate aldolase OS=Drosophila melanogaster OX=7227 GN=Ald1 PE=1 SV=5	0	82.143	100
P_KWMTBOMO09621	tr|H9JTI9|H9JTI9_BOMMO	Eukaryotic translation initiation factor 3 subunit C OS=Bombyx mori OX=7091 GN=eIF3c PE=3 SV=1	0	100	100	sp|Q0ZB76|EIF3C_BOMMO	Eukaryotic translation initiation factor 3 subunit C OS=Bombyx mori OX=7091 GN=eIF3-S8 PE=2 SV=1	0	99.886	100
P_KWMTBOMO09631	tr|H9JTW9|H9JTW9_BOMMO	GOLD domain-containing protein OS=Bombyx mori OX=7091 GN=101737070 PE=3 SV=1	5.12E-155	100	100	sp|P49020|TMED2_CRIGR	Transmembrane emp24 domain-containing protein 2 (Fragment) OS=Cricetulus griseus OX=10029 GN=TMED2 PE=1 SV=1	4.26E-87	63.587	90.19607843
P_KWMTBOMO09672	tr|H9JTU8|H9JTU8_BOMMO	Uncharacterized protein OS=Bombyx mori OX=7091 PE=4 SV=1	7.15E-83	98.276	42.64705882	sp|P48738|PIPNA_RABIT	Phosphatidylinositol transfer protein alpha isoform OS=Oryctolagus cuniculus OX=9986 GN=PITPNA PE=3 SV=2	1.85E-115	59.928	100
P_KWMTBOMO09747	tr|B7XFU6|B7XFU6_BOMMO	Clathrin heavy chain OS=Bombyx mori OX=7091 GN=100233163 PE=2 SV=1	0	99.941	100	sp|P29742|CLH_DROME	Clathrin heavy chain OS=Drosophila melanogaster OX=7227 GN=Chc PE=1 SV=1	0	85.196	100
P_KWMTBOMO09808	tr|Q2F6C3|Q2F6C3_BOMMO	Chaperonin subunit 6a zeta OS=Bombyx mori OX=7091 GN=692796 PE=2 SV=1	0	100	100	sp|P80317|TCPZ_MOUSE	T-complex protein 1 subunit zeta OS=Mus musculus OX=10090 GN=Cct6a PE=1 SV=3	0	72.505	100
P_KWMTBOMO09838	tr|H9JUC0|H9JUC0_BOMMO	Asparagine--tRNA ligase OS=Bombyx mori OX=7091 PE=3 SV=1	0	99.104	100	sp|Q2KJG3|SYNC_BOVIN	Asparagine--tRNA ligase, cytoplasmic OS=Bos taurus OX=9913 GN=NARS PE=2 SV=3	0	70.599	99.45848375
P_KWMTBOMO10129	tr|Q2F5X3|Q2F5X3_BOMMO	26S proteasome non-ATPase regulatory subunit 4 OS=Bombyx mori OX=7091 GN=778515 PE=2 SV=1	0	100	100	sp|P55035|PSMD4_DROME	26S proteasome non-ATPase regulatory subunit 4 OS=Drosophila melanogaster OX=7227 GN=Rpn10 PE=1 SV=2	1.63E-145	60.677	100
P_KWMTBOMO10248	tr|H9JCR4|H9JCR4_BOMMO	40S ribosomal protein SA OS=Bombyx mori OX=7091 PE=3 SV=1	0	100	100	sp|Q5UAP4|RSSA_BOMMO	40S ribosomal protein SA OS=Bombyx mori OX=7091 PE=2 SV=1	0	99.673	100
P_KWMTBOMO10283	tr|H9JCM2|H9JCM2_BOMMO	PCI domain-containing protein OS=Bombyx mori OX=7091 GN=692900 PE=3 SV=1	0	100	100	sp|Q9D8W5|PSD12_MOUSE	26S proteasome non-ATPase regulatory subunit 12 OS=Mus musculus OX=10090 GN=Psmd12 PE=1 SV=4	0	59.071	100
P_KWMTBOMO10337	tr|H9JCI5|H9JCI5_BOMMO	ERF-3 OS=Bombyx mori OX=7091 PE=4 SV=1	0	100	96.32352941	sp|Q8R050|ERF3A_MOUSE	Eukaryotic peptide chain release factor GTP-binding subunit ERF3A OS=Mus musculus OX=10090 GN=Gspt1 PE=1 SV=2	0	66.599	90.25735294
P_KWMTBOMO10358	tr|H9JX26|H9JX26_BOMMO	DUF4781 domain-containing protein OS=Bombyx mori OX=7091 PE=4 SV=1	0	79.827	48.32869081	--	--	--	--	--
P_KWMTBOMO10779	tr|Q0N2R5|Q0N2R5_BOMMO	Signal recognition particle receptor alpha subunit OS=Bombyx mori OX=7091 PE=2 SV=1	0	99.51	100	sp|Q9U5L1|SRPRA_DROME	Signal recognition particle receptor subunit alpha homolog OS=Drosophila melanogaster OX=7227 GN=SrpRalpha PE=1 SV=2	0	59.486	100
P_KWMTBOMO10780	tr|H9JFX9|H9JFX9_BOMMO	Uncharacterized protein OS=Bombyx mori OX=7091 PE=4 SV=1	0	90.138	43.81909548	sp|Q9V3P6|PSMD1_DROME	26S proteasome non-ATPase regulatory subunit 1 OS=Drosophila melanogaster OX=7227 GN=Rpn2 PE=1 SV=1	0	71.218	100
P_KWMTBOMO10916	tr|Q1HQ44|Q1HQ44_BOMMO	Proteasome subunit alpha type OS=Bombyx mori OX=7091 PE=2 SV=1	0	100	100	sp|O70435|PSA3_MOUSE	Proteasome subunit alpha type-3 OS=Mus musculus OX=10090 GN=Psma3 PE=1 SV=3	2.47E-130	65.49	100
P_KWMTBOMO10965	tr|Q1HQC6|Q1HQC6_BOMMO	26S protease regulatory subunit 6B OS=Bombyx mori OX=7091 PE=2 SV=1	0	100	100	sp|P46507|PRS6B_MANSE	26S proteasome regulatory subunit 6B OS=Manduca sexta OX=7130 PE=2 SV=1	0	98.313	100
P_KWMTBOMO11021	tr|Q5UAM3|Q5UAM3_BOMMO	Ribosomal protein S19 OS=Bombyx mori OX=7091 GN=RpS19 PE=2 SV=1	6.33E-111	100	100	sp|P39018|RS19A_DROME	40S ribosomal protein S19a OS=Drosophila melanogaster OX=7227 GN=RpS19a PE=1 SV=3	5.94E-71	66.892	96.73202614
P_KWMTBOMO11061	tr|Q2F5N1|Q2F5N1_BOMMO	Prohibitin OS=Bombyx mori OX=7091 GN=693108 PE=2 SV=1	0	100	100	sp|Q5XIH7|PHB2_RAT	Prohibitin-2 OS=Rattus norvegicus OX=10116 GN=Phb2 PE=1 SV=1	5.66E-158	72.667	100
P_KWMTBOMO11096	tr|A1E9B3|A1E9B3_BOMMO	H(+)-transporting two-sector ATPase OS=Bombyx mori OX=7091 PE=2 SV=1	0	99.676	100	sp|P31400|VATA_MANSE	V-type proton ATPase catalytic subunit A OS=Manduca sexta OX=7130 GN=VHAA PE=2 SV=1	0	98.217	100
P_KWMTBOMO11202	tr|H9JT03|H9JT03_BOMMO	F-actin-capping protein subunit alpha OS=Bombyx mori OX=7091 GN=101738938 PE=3 SV=1	0	100	100	sp|Q9W2N0|CAPZA_DROME	F-actin-capping protein subunit alpha OS=Drosophila melanogaster OX=7227 GN=cpa PE=2 SV=1	1.31E-147	68.44	97.91666667
P_KWMTBOMO11255	tr|Q5UAN5|Q5UAN5_BOMMO	40S ribosomal protein S9 OS=Bombyx mori OX=7091 GN=RpS9 PE=2 SV=1	5.62E-140	100	100	sp|B4PEU8|RS9_DROYA	40S ribosomal protein S9 OS=Drosophila yakuba OX=7245 GN=RpS9 PE=2 SV=1	2.83E-117	89.231	100

P_KWMTBOMO11336	tr|H9IX66|H9IX66_BOMMO	Threonyl-tRNA synthetase OS=Bombyx mori OX=7091 PE=3 SV=1	0	98.151	98.59747546	sp|Q3ZBV8|SYTC_BOVIN	Threonine--tRNA ligase 1, cytoplasmic OS=Bos taurus OX=9913 GN=TARS1 PE=2 SV=1	0	69.371	100
P_KWMTBOMO11448	tr|H9IXE5|H9IXE5_BOMMO	Uncharacterized protein OS=Bombyx mori OX=7091 GN=733057 PE=4 SV=1	1.36E-160	100	100	sp|P53994|RAB2A_MOUSE	Ras-related protein Rab-2A OS=Mus musculus OX=10090 GN=Rab2a PE=1 SV=1	2.20E-141	90.654	100
P_KWMTBOMO11590	tr|H9J3D2|H9J3D2_BOMMO	Ku domain-containing protein OS=Bombyx mori OX=7091 GN=101736121 PE=4 SV=1	0	98.077	80.68965517	sp|O93257|XRCC6_CHICK	X-ray repair cross-complementing protein 5 OS=Gallus gallus OX=9031 GN=XRCC6 PE=2 SV=1	5.59E-42	26.126	95.68965517
P_KWMTBOMO11666	tr|Q2V0H5|Q2V0H5_BOMMO	Vesicle-fusing ATPase OS=Bombyx mori OX=7091 GN=ter94 PE=2 SV=1	0	100	100	sp|Q7KN62|TERA_DROME	Transitional endoplasmic reticulum ATPase TER94 OS=Drosophila melanogaster OX=7227 GN=TER94 PE=1 SV=1	0	88.025	100
P_KWMTBOMO11680	tr|H9J390|H9J390_BOMMO	Seryl-tRNA synthetase OS=Bombyx mori OX=7091 GN=101743384 PE=3 SV=1	0	100	100	sp|Q9GMB8|SYSC_BOVIN	Serine--tRNA ligase, cytoplasmic OS=Bos taurus OX=9913 GN=SARS1 PE=2 SV=3	0	69.495	100
P_KWMTBOMO12018	tr|Q5UAN7|Q5UAN7_BOMMO	40S ribosomal protein S7 OS=Bombyx mori OX=7091 GN=RpS7 PE=2 SV=1	1.24E-137	99.474	100	sp|P48155|RS7_MANSE	40S ribosomal protein S7 OS=Manduca sexta OX=7130 GN=RpS7 PE=2 SV=1	1.42E-134	97.368	100
P_KWMTBOMO12166	tr|H9JWZ4|H9JWZ4_BOMMO	NADH dehydrogenase [ubiquinone] 1 alpha subcomplex subunit 10, mitochondrial OS=Bombyx mori OX=7091 GN=101745480 PE=3 SV=1	0	100	100	sp|P91929|NDUAA_DROME	NADH dehydrogenase [ubiquinone] 1 alpha subcomplex subunit 10, mitochondrial OS=Drosophila melanogaster OX=7227 GN=ND-42 PE=1 SV=2	1.80E-134	49.485	97.48743719
P_KWMTBOMO12285	tr|Q93137|Q93137_BOMMO	Reverse transcriptase OS=Bombyx mori OX=7091 PE=4 SV=1	0	71.082	49.23913043	sp|O17449|TBB1_MANSE	Tubulin beta-1 chain OS=Manduca sexta OX=7130 PE=2 SV=1	0	99.769	23.47826087
P_KWMTBOMO12389	tr|H9JSC6|H9JSC6_BOMMO	Coatomer subunit epsilon OS=Bombyx mori OX=7091 PE=3 SV=1	0	100	100	sp|Q5ZIK9|COPE_CHICK	Coatomer subunit epsilon OS=Gallus gallus OX=9031 GN=COPE PE=2 SV=1	6.42E-96	48.475	97.68211921
P_KWMTBOMO12428	sp|Q5UAS2|RL17_BOMMO	60S ribosomal protein L17 OS=Bombyx mori OX=7091 GN=RpL17 PE=2 SV=1	5.32E-141	100	100	sp|Q5UAS2|RL17_BOMMO	60S ribosomal protein L17 OS=Bombyx mori OX=7091 GN=RpL17 PE=2 SV=1	3.47E-140	100	100
P_KWMTBOMO12448	tr|H9IYM5|H9IYM5_BOMMO	Secretory carrier-associated membrane protein OS=Bombyx mori OX=7091 PE=3 SV=1	1.69E-99	87.5	73.01587302	sp|Q8K021|SCAM1_MOUSE	Secretory carrier-associated membrane protein 1 OS=Mus musculus OX=10090 GN=Scamp1 PE=1 SV=1	8.88E-77	52.838	90.87301587
P_KWMTBOMO12560	tr|A0A1Q1NKL1|A0A1Q1NKL1_BOMMO	Tyrosine--tRNA ligase OS=Bombyx mori OX=7091 PE=2 SV=1	0	99.809	100	sp|Q5ZJ08|SYYC_CHICK	Tyrosine--tRNA ligase, cytoplasmic OS=Gallus gallus OX=9031 GN=YARS1 PE=2 SV=1	0	71.374	100
P_KWMTBOMO12602	tr|H9IWB5|H9IWB5_BOMMO	Uncharacterized protein OS=Bombyx mori OX=7091 PE=3 SV=1	0	95.142	67.58824981	sp|P37276|DYHC_DROME	Dynein heavy chain, cytoplasmic OS=Drosophila melanogaster OX=7227 GN=Dhc64C PE=2 SV=2	0	79.96	99.77783263
P_KWMTBOMO12628	tr|H9IWC7|H9IWC7_BOMMO	Isoleucyl-tRNA synthetase OS=Bombyx mori OX=7091 GN=101742308 PE=3 SV=1	0	99.835	100	sp|Q8BU30|SYIC_MOUSE	Isoleucine--tRNA ligase, cytoplasmic OS=Mus musculus OX=10090 GN=Iars1 PE=1 SV=2	0	60.246	100
P_KWMTBOMO12667	tr|Q1HPM7|Q1HPM7_BOMMO	Mitochondrial aldehyde dehydrogenase OS=Bombyx mori OX=7091 PE=2 SV=1	0	97.291	47.22814499	sp|P81178|ALDH2_MESAU	Aldehyde dehydrogenase, mitochondrial OS=Mesocricetus auratus OX=10036 GN=ALDH2 PE=1 SV=1	0	70	45.84221748
P_KWMTBOMO12706	tr|Q5UAQ2|Q5UAQ2_BOMMO	60S ribosomal protein L36 OS=Bombyx mori OX=7091 GN=RpL36 PE=2 SV=1	1.02E-81	100	100	sp|P49630|RL36_DROME	60S ribosomal protein L36 OS=Drosophila melanogaster OX=7227 GN=RpL36 PE=1 SV=1	4.53E-50	71.304	96.63865546
P_KWMTBOMO12844	tr|H9U396|H9U396_BOMMO	Endoplasmin-like protein OS=Bombyx mori OX=7091 PE=2 SV=1	0	100	100	sp|Q66HD0|ENPL_RAT	Endoplasmin OS=Rattus norvegicus OX=10116 GN=Hsp90b1 PE=1 SV=2	0	59.264	93.95061728
P_KWMTBOMO12878	tr|Q0KIX8|Q0KIX8_BOMMO	Glutamate synthase OS=Bombyx mori OX=7091 GN=BmGOGAT PE=2 SV=1	0	99.658	100	sp|Q18164|DPYD_CAEEL	Dihydropyrimidine dehydrogenase [NADP(+)] OS=Caenorhabditis elegans OX=6239 GN=dpyd-1 PE=3 SV=2	2.57E-20	29.206	15.39589443
P_KWMTBOMO13226	tr|H6VTQ9|H6VTQ9_BOMMO	DnaJ-19 OS=Bombyx mori OX=7091 PE=2 SV=1	0	100	99.29775281	sp|Q9UGP8|SEC63_HUMAN	Translocation protein SEC63 homolog OS=Homo sapiens OX=9606 GN=SEC63 PE=1 SV=2	0	44.537	100
P_KWMTBOMO13293	tr|E9JEI6|E9JEI6_BOMMO	Poly [ADP-ribose] polymerase (Fragment) OS=Bombyx mori OX=7091 PE=2 SV=1	0	99.894	94.5	sp|Q11208|PARP_SARPE	Poly [ADP-ribose] polymerase OS=Sarcophaga peregrina OX=7386 PE=1 SV=1	0	48.473	100
P_KWMTBOMO13370	tr|H9JN38|H9JN38_BOMMO	Signal recognition particle subunit SRP72 OS=Bombyx mori OX=7091 PE=3 SV=1	0	100	66.30602782	sp|O76094|SRP72_HUMAN	Signal recognition particle subunit SRP72 OS=Homo sapiens OX=9606 GN=SRP72 PE=1 SV=3	7.11E-121	37.721	96.29057187
P_KWMTBOMO13722	tr|H9JPQ0|H9JPQ0_BOMMO	Uncharacterized protein OS=Bombyx mori OX=7091 PE=3 SV=1	2.24E-39	100	73.56321839	sp|Q99417|MYCBP_HUMAN	c-Myc-binding protein OS=Homo sapiens OX=9606 GN=MYCBP PE=1 SV=3	2.31E-23	56.944	82.75862069
P_KWMTBOMO13723	tr|Q1HPX3|Q1HPX3_BOMMO	ATP synthase subunit O, mitochondrial OS=Bombyx mori OX=7091 PE=2 SV=1	1.17E-152	100	100	sp|Q24439|ATPO_DROME	ATP synthase subunit O, mitochondrial OS=Drosophila melanogaster OX=7227 GN=ATPsynO PE=2 SV=2	7.56E-89	61.692	96.1722488
P_KWMTBOMO13729	tr|Q684K3|Q684K3_BOMMO	Protein-synthesizing GTPase OS=Bombyx mori OX=7091 GN=eIF2g PE=2 SV=1	0	100	100	sp|Q24208|IF2G_DROME	Eukaryotic translation initiation factor 2 subunit 3 OS=Drosophila melanogaster OX=7227 GN=eIF2gamma PE=2 SV=1	0	84.12	99.57264957
P_KWMTBOMO13960	tr|Q5UAN0|Q5UAN0_BOMMO	40S ribosomal protein S13 OS=Bombyx mori OX=7091 GN=RpS13 PE=2 SV=1	8.93E-110	100	100	sp|Q962R6|RS13_SPOFR	40S ribosomal protein S13 OS=Spodoptera frugiperda OX=7108 GN=RpS13 PE=2 SV=3	1.63E-108	99.338	100
P_KWMTBOMO14041	tr|Q2F5J1|Q2F5J1_BOMMO	26S proteasome non-ATPase regulatory subunit 13 OS=Bombyx mori OX=7091 PE=2 SV=1	0	100	100	sp|B0BN93|PSD13_RAT	26S proteasome non-ATPase regulatory subunit 13 OS=Rattus norvegicus OX=10116 GN=Psmd13 PE=1 SV=1	1.26E-102	46.921	88.57142857

P_KWMTBOMO14157	tr|H9JNS6|H9JNS6_BOMMO	Coatomer subunit gamma OS=Bombyx mori OX=7091 GN=692402 PE=3 SV=1	0	99.768	100	sp|Q29AE5|COPG_DROPS	Coatomer subunit gamma OS=Drosophila pseudoobscura pseudoobscura OX=46245 GN=gammaCop PE=3 SV=1	0	58.75	100
P_KWMTBOMO14440	tr|Q1HPP5|Q1HPP5_BOMMO	Actin-depolymerizing factor 1 OS=Bombyx mori OX=7091 PE=2 SV=1	1.70E-108	100	100	sp|P45594|CADF_DROME	Cofilin/actin-depolymerizing factor homolog OS=Drosophila melanogaster OX=7227 GN=tsr PE=1 SV=1	2.22E-100	92.568	100
P_KWMTBOMO14478	tr|H9IS48|H9IS48_BOMMO	Uncharacterized protein OS=Bombyx mori OX=7091 GN=101742289 PE=3 SV=1	1.04E-66	100	100	sp|Q9W1V3|FBRL_DROME	rRNA 2'-O-methyltransferase fibrillarin OS=Drosophila melanogaster OX=7227 GN=Fib PE=2 SV=1	4.31E-61	94.737	100
P_KWMTBOMO14479	tr|H9IS48|H9IS48_BOMMO	Uncharacterized protein OS=Bombyx mori OX=7091 GN=101742289 PE=3 SV=1	7.91E-100	100	100	sp|Q8I1F4|FBRL_DROER	rRNA 2'-O-methyltransferase fibrillarin OS=Drosophila erecta OX=7220 GN=Fib PE=3 SV=1	2.11E-41	82.278	50
P_KWMTBOMO14530	tr|Q2F5R7|Q2F5R7_BOMMO	Eukaryotic peptide chain release factor subunit 1 OS=Bombyx mori OX=7091 PE=2 SV=1	0	99.314	100	sp|Q9VPH7|ERF1_DROME	Eukaryotic peptide chain release factor subunit 1 OS=Drosophila melanogaster OX=7227 GN=eRF1 PE=1 SV=2	0	93.822	100
P_KWMTBOMO14549	tr|I6XKQ0|I6XKQ0_BOMMO	Heat shock protein 70-5 OS=Bombyx mori OX=7091 PE=2 SV=1	0	99.565	100	sp|P29845|HSP7E_DROME	Heat shock 70 kDa protein cognate 5 OS=Drosophila melanogaster OX=7227 GN=Hsc70-5 PE=1 SV=2	0	81.86	93.47826087
P_KWMTBOMO14639	tr|Q5UAQ6|Q5UAQ6_BOMMO	60S ribosomal protein L32 OS=Bombyx mori OX=7091 GN=RpL32 PE=2 SV=1	1.84E-97	100	100	sp|Q962T1|RL32_SPOFR	60S ribosomal protein L32 OS=Spodoptera frugiperda OX=7108 GN=RpL32 PE=2 SV=1	6.75E-95	97.761	100
P_KWMTBOMO14715	tr|Q1HPL2|Q1HPL2_BOMMO	Phosphate carrier 1 OS=Bombyx mori OX=7091 GN=PiC PE=2 SV=1	0	100	100	sp|O61703|MPCP_CHOFU	Phosphate carrier protein, mitochondrial OS=Choristoneura fumiferana OX=7141 PE=2 SV=1	0	78.771	100
P_KWMTBOMO14732	tr|Q86PG2|Q86PG2_BOMMO	ADP/ATP translocase OS=Bombyx mori OX=7091 GN=ANT PE=2 SV=1	0	100	100	sp|Q26365|ADT_DROME	ADP,ATP carrier protein OS=Drosophila melanogaster OX=7227 GN=sesB PE=2 SV=4	0	87.458	98.33333333
P_KWMTBOMO14761	tr|H9J2S8|H9J2S8_BOMMO	Dihydrolipoyllysine-residue succinyltransferase component of 2-oxoglutarate dehydrogenase complex, mitochondrial OS=Bombyx mori OX=7091 PE=3 SV=1	0	78.71	100	sp|P11179|ODO2_BOVIN	Dihydrolipoyllysine-residue succinyltransferase component of 2-oxoglutarate dehydrogenase complex, mitochondrial OS=Bos taurus OX=9913 GN=DLST PE=1 SV=2	8.56E-150	56.542	92.04301075
P_KWMTBOMO14887	tr|H9JRM2|H9JRM2_BOMMO	T-complex protein 1 subunit eta OS=Bombyx mori OX=7091 PE=3 SV=1	0	99.631	100	sp|Q5ZJK8|TCPH_CHICK	T-complex protein 1 subunit eta OS=Gallus gallus OX=9031 GN=CCT7 PE=1 SV=1	0	69.376	97.60147601
P_KWMTBOMO14936	tr|H9IU37|H9IU37_BOMMO	GB1/RHD3-type G domain-containing protein OS=Bombyx mori OX=7091 PE=3 SV=1	0	97.695	100	sp|Q9VC57|ATLAS_DROME	Atlastin OS=Drosophila melanogaster OX=7227 GN=atl PE=1 SV=1	0	68.275	97.82214156
P_KWMTBOMO14942	tr|H9IU76|H9IU76_BOMMO	AMP deaminase OS=Bombyx mori OX=7091 PE=3 SV=1	0	98.229	97.60638298	sp|Q02356|AMPD2_RAT	AMP deaminase 2 OS=Rattus norvegicus OX=10116 GN=Ampd2 PE=1 SV=2	0	59.916	95.21276596
P_KWMTBOMO14984	tr|H9J5J2|H9J5J2_BOMMO	AAA domain-containing protein OS=Bombyx mori OX=7091 PE=3 SV=1	0	100	100	sp|O42587|PR6AA_XENLA	26S proteasome regulatory subunit 6A-A OS=Xenopus laevis OX=8355 GN=psmc3-a PE=2 SV=2	0	88.124	98.36448598
P_KWMTBOMO15060	tr|H9J5R2|H9J5R2_BOMMO	Protein transport protein SEC23 OS=Bombyx mori OX=7091 PE=3 SV=1	0	100	100	sp|Q01405|SC23A_MOUSE	Protein transport protein Sec23A OS=Mus musculus OX=10090 GN=Sec23a PE=1 SV=2	0	74.583	100
P_KWMTBOMO15114	tr|Q5UAL8|Q5UAL8_BOMMO	40S ribosomal protein S25 OS=Bombyx mori OX=7091 GN=RpS25 PE=2 SV=1	7.13E-83	100	100	sp|Q962Q5|RS25_SPOFR	40S ribosomal protein S25 OS=Spodoptera frugiperda OX=7108 GN=RpS25 PE=3 SV=1	1.98E-81	99.16	100
P_KWMTBOMO15121	tr|Q0N2R8|Q0N2R8_BOMMO	Signal recognition particle 54 kDa protein OS=Bombyx mori OX=7091 GN=SRP54 PE=2 SV=1	0	99.601	100	sp|Q7ZVN5|SRP54_DANRE	Signal recognition particle 54 kDa protein OS=Danio rerio OX=7955 GN=srp54 PE=2 SV=1	0	80.2	99.8003992
P_KWMTBOMO15123	tr|H9J5W8|H9J5W8_BOMMO	AAA domain-containing protein OS=Bombyx mori OX=7091 PE=3 SV=2	0	99.241	90.38901602	sp|P48601|PRS4_DROME	26S proteasome regulatory subunit 4 OS=Drosophila melanogaster OX=7227 GN=Rpt2 PE=1 SV=2	0	96.811	100
P_KWMTBOMO15357	tr|H9J6H5|H9J6H5_BOMMO	Prolyl-tRNA synthetase OS=Bombyx mori OX=7091 PE=3 SV=1	0	98.315	99.64028777	sp|P28668|SYEP_DROME	Bifunctional glutamate/proline--tRNA ligase OS=Drosophila melanogaster OX=7227 GN=GluProRS PE=1 SV=2	0	61.306	99.16067146
P_KWMTBOMO15359	tr|H9J6H4|H9J6H4_BOMMO	Glutamyl-tRNA synthetase OS=Bombyx mori OX=7091 PE=3 SV=1	0	100	97.09944751	sp|P28668|SYEP_DROME	Bifunctional glutamate/proline--tRNA ligase OS=Drosophila melanogaster OX=7227 GN=GluProRS PE=1 SV=2	0	60.526	99.72375691
P_KWMTBOMO15361	tr|O16143|O16143_BOMMO	Rab1 protein OS=Bombyx mori OX=7091 GN=rabB PE=2 SV=1	5.31E-153	100	100	sp|Q05974|RAB1A_LYMST	Ras-related protein Rab-1A OS=Lymnaea stagnalis OX=6523 GN=RAB1A PE=2 SV=1	4.74E-124	81.188	100
P_KWMTBOMO15518	tr|Q1HPL0|Q1HPL0_BOMMO	26S proteasome regulatory ATPase subunit 10B OS=Bombyx mori OX=7091 PE=2 SV=1	0	100	100	sp|P62334|PRS10_MOUSE	26S proteasome regulatory subunit 10B OS=Mus musculus OX=10090 GN=Psmc6 PE=1 SV=1	0	88.571	97.22222222
P_KWMTBOMO15547	tr|H9JMI6|H9JMI6_BOMMO	Nop domain-containing protein OS=Bombyx mori OX=7091 PE=3 SV=1	0	91.939	99.35064935	sp|O00567|NOP56_HUMAN	Nucleolar protein 56 OS=Homo sapiens OX=9606 GN=NOP56 PE=1 SV=4	0	66.59	93.93939394
P_KWMTBOMO15584	tr|D2Y4R2|D2Y4R2_BOMMO	Coatomer subunit alpha OS=Bombyx mori OX=7091 GN=COPA PE=2 SV=1	0	99.729	99.72972973	sp|P53621|COPA_HUMAN	Coatomer subunit alpha OS=Homo sapiens OX=9606 GN=COPA PE=1 SV=2	0	78.729	97.83783784
P_KWMTBOMO15665	tr|H9IYF4|H9IYF4_BOMMO	Uncharacterized protein OS=Bombyx mori OX=7091 GN=101736745 PE=4 SV=1	0	100	100	sp|P26305|LPSBP_PERAM	Hemolymph lipopolysaccharide-binding protein OS=Periplaneta americana OX=6978 PE=1 SV=1	3.37E-26	39.716	45.19230769
P_KWMTBOMO15712	tr|Q2F5N9|Q2F5N9_BOMMO	Nucleoplasmin isoform 2 OS=Bombyx mori OX=7091 GN=692956 PE=2 SV=1	7.05E-137	100	100	sp|Q27415|NLP_DROME	Nucleoplasmin-like protein OS=Drosophila melanogaster OX=7227 GN=Nlp PE=1 SV=1	2.34E-29	48.148	57.7540107

P_KWMTBOMO15758	tr|B3VBE3|B3VBE3_BOMMO	Vacuolar proton pump subunit B OS=Bombyx mori OX=7091 PE=2 SV=1	0	99.278	88.21656051	sp|P31401|VATB_MANSE	V-type proton ATPase subunit B OS=Manduca sexta OX=7130 GN=VHA55 PE=2 SV=1	0	97.112	88.21656051
P_KWMTBOMO15781	tr|H9IYL2|H9IYL2_BOMMO	Uncharacterized protein OS=Bombyx mori OX=7091 PE=3 SV=2	0	87.5	100	sp|O46036|CTBP_DROME	C-terminal-binding protein OS=Drosophila melanogaster OX=7227 GN=CtBP PE=1 SV=3	0	91.643	78.9010989
P_KWMTBOMO16197	tr|Q1HPX4|Q1HPX4_BOMMO	ATP synthase subunit gamma OS=Bombyx mori OX=7091 GN=732965 PE=2 SV=1	0	100	100	sp|O01666|ATPG_DROME	ATP synthase subunit gamma, mitochondrial OS=Drosophila melanogaster OX=7227 GN=ATPsyngamma PE=2 SV=2	2.51E-151	73.05	95.27027027
P_KWMTBOMO16204	tr|H9JWQ7|H9JWQ7_BOMMO	CHK domain-containing protein OS=Bombyx mori OX=7091 GN=101744771 PE=4 SV=1	0	100	100	--	--	--	--	--
P_KWMTBOMO16261	tr|Q2F6A0|Q2F6A0_BOMMO	Exuperantia OS=Bombyx mori OX=7091 PE=2 SV=1	0	100	100	sp|Q24747|EXU_DROVI	Maternal protein exuperantia OS=Drosophila virilis OX=7244 GN=exu PE=3 SV=1	5.40E-82	39.231	94.43099274
P_KWMTBOMO16309	tr|H9JW60|H9JW60_BOMMO	UBX domain-containing protein OS=Bombyx mori OX=7091 PE=4 SV=1	1.01E-106	92.353	43.58974359	sp|Q3SZC4|NSF1C_BOVIN	NSFL1 cofactor p47 OS=Bos taurus OX=9913 GN=NSFL1C PE=2 SV=1	6.48E-85	40.852	100
P_KWMTBOMO16319	tr|Q2F5K7|Q2F5K7_BOMMO	Receptor expression-enhancing protein OS=Bombyx mori OX=7091 PE=2 SV=1	3.16E-130	99.429	100	sp|Q29RM3|REEP5_BOVIN	Receptor expression-enhancing protein 5 OS=Bos taurus OX=9913 GN=REEP5 PE=2 SV=1	3.07E-59	50.595	96
P_KWMTBOMO16357	tr|Q5UAN3|Q5UAN3_BOMMO	40S ribosomal protein S11 OS=Bombyx mori OX=7091 GN=RpS11-1 PE=2 SV=1	6.00E-114	100	100	sp|P62280|RS11_HUMAN	40S ribosomal protein S11 OS=Homo sapiens OX=9606 GN=RPS11 PE=1 SV=3	4.63E-88	77.848	100
P_KWMTBOMO16481	tr|Q19KB8|Q19KB8_BOMMO	Ras-related GTP-binding protein Rab11 OS=Bombyx mori OX=7091 GN=733063 PE=2 SV=1	1.33E-160	100	100	sp|P62492|RB11A_MOUSE	Ras-related protein Rab-11A OS=Mus musculus OX=10090 GN=Rab11a PE=1 SV=3	7.02E-129	82.326	100

Supplementary Table S5. List of PIPs associated with Figure 6.

PIPs associated with Figure 6a

Query Item	String Id	Identity	Bit score	Preferred Name	Annotation
P_KWMTBOMO01918	7091.BGIBMGA006209-TA	100	100.5	LOC733070	Protein transport protein Sec61 subunit beta; Necessary for protein translocation in the endoplasmic reticulum
P_KWMTBOMO02470	7091.BGIBMGA002570-TA	100	912.9	BGIBMGA002570-TA	Dolichyl-diphosphooligosaccharide--protein glycosyltransferase subunit 1; Essential subunit of the N-oligosaccharyl transferase (OST) complex which catalyzes the transfer of a high mannose oligosaccharide from a lipid-linked oligosaccharide donor to an asparagine residue within an Asn-X-Ser/Thr consensus motif in nascent polypeptide chains
P_KWMTBOMO02432	7091.BGIBMGA013498-TA	100	1187.6	BGIBMGA013498-TA	annotation not available
P_KWMTBOMO12895	7091.BGIBMGA012687-TA	100	926	Sec61alpha	Transport protein Sec61 alpha subunit
P_KWMTBOMO08100	7091.BGIBMGA001106-TA	100	316.2	RpL21	Ribosomal protein L21
P_KWMTBOMO11255	7091.BGIBMGA012626-TA	100	375.2	RpS9	Ribosomal protein S9 ; Belongs to the universal ribosomal protein uS4 family
P_KWMTBOMO01273	7091.BGIBMGA008865-TA	100	416.8	RpL13A	Ribosomal protein L13A
P_KWMTBOMO06229	7091.BGIBMGA001800-TA	100	729.9	RpL4	annotation not available
P_KWMTBOMO02059	7091.BGIBMGA003197-TA	100	273.9	RpS18	annotation not available
P_KWMTBOMO16357	7091.BGIBMGA013792-TA	79	293.9	RpS11	annotation not available
P_KWMTBOMO05979	7091.BGIBMGA002811-TA	100	434.9	RpL10	Ribosomal protein L10
P_KWMTBOMO09300	7091.BGIBMGA003309-TA	100	544.7	RpP0	60S acidic ribosomal protein P0; Ribosomal protein P0 is the functional equivalent of E.coli protein L10
P_KWMTBOMO09442	7091.BGIBMGA013076-TA	95.2	499.6	RpL6	annotation not available
P_KWMTBOMO08666	7091.BGIBMGA007645-TA	100	285.8	RpS23	annotation not available
P_KWMTBOMO11985	7091.BGIBMGA004375-TA	100	490.7	RpL7	Ribosomal protein L7
P_KWMTBOMO05675	7091.BGIBMGA006835-TA	100	250	RpL23A	annotation not available
P_KWMTBOMO06974	7091.BGIBMGA010487-TA	100	179.1	RpL36A	Ribosomal protein L36A ; Belongs to the eukaryotic ribosomal protein eL42 family
P_KWMTBOMO10248	7091.BGIBMGA007311-TA	100	547	RpSA	40S ribosomal protein SA; Required for the assembly and/or stability of the 40S ribosomal subunit. Required for the processing of the 20S rRNA- precursor to mature 18S rRNA in a late step of the maturation of 40S ribosomal subunits
P_KWMTBOMO13569	7091.BGIBMGA010970-TA	100	200.3	RpL26	Ribosomal protein L26
P_KWMTBOMO07688	7091.BGIBMGA000867-TA	100	447.2	RpS2e	Ribosomal protein S2 ; Belongs to the universal ribosomal protein uS5 family
P_KWMTBOMO06480	7091.BGIBMGA011620-TA	100	351.3	RpL18	60S ribosomal protein L18
P_KWMTBOMO13455	7091.BGIBMGA010867-TA	100	495.4	RpS4	40S ribosomal protein S4 ; Belongs to the eukaryotic ribosomal protein eS4 family
P_KWMTBOMO15114	7091.BGIBMGA004905-TA	98.3	172.6	RpS25	annotation not available
P_KWMTBOMO03314	7091.BGIBMGA006414-TA	100	403.7	RpL15	Ribosomal protein L15 ; Belongs to the eukaryotic ribosomal protein eL15 family
P_KWMTBOMO13960	7091.BGIBMGA011282-TA	100	301.6	RpS13	Ribosomal protein S13 ; Belongs to the universal ribosomal protein uS15 family
P_KWMTBOMO09230	7091.BGIBMGA003412-TA	100	135.6	RpP1	annotation not available
P_KWMTBOMO11731	7091.BGIBMGA004165-TA	100	1686.8	tef2	Translation elongation factor 2; Catalyzes the GTP-dependent ribosomal translocation step during translation elongation. During this step, the ribosome changes from the pre-translocational (PRE) to the post- translocational (POST) state as the newly formed A-site-bound peptidyl-tRNA and P-site-bound deacylated tRNA move to the P and E sites, respectively. Catalyzes the coordinated movement of the two tRNA molecules, the mRNA and conformational changes in the ribosome
P_KWMTBOMO11083	7091.BGIBMGA008302-TA	100	800.4	Ef-1g	Elongation factor 1 gamma
P_KWMTBOMO02081	7091.BGIBMGA003186-TA	100	833.9	Eif-4a	Eukaryotic translation initiation factor 4A ; Belongs to the DEAD box helicase family
P_KWMTBOMO14804	7091.BGIBMGA012211-TA	100	511.9	BGIBMGA012211-TA	annotation not available
P_KWMTBOMO10493	7091.BGIBMGA007114-TA	99.6	1402.9	eIF3-S9	Eukaryotic translation initiation factor 3 subunit B; RNA-binding component of the eukaryotic translation initiation factor 3 (eIF-3) complex, which is involved in protein synthesis of a specialized repertoire of mRNAs and, together with other initiation factors, stimulates binding of mRNA and methionyl-tRNAi to the 40S ribosome. The eIF-3 complex specifically targets and initiates translation of a subset of mRNAs involved in cell proliferation
P_KWMTBOMO08776	7091.BGIBMGA007889-TA	100	681	eIF3i	Eukaryotic translation initiation factor 3 subunit I; Component of the eukaryotic translation initiation factor 3 (eIF-3) complex, which is involved in protein synthesis of a specialized repertoire of mRNAs and, together with other initiation factors, stimulates binding of mRNA and methionyl-tRNAi to the 40S ribosome. The eIF-3 complex specifically targets and initiates translation of a subset of mRNAs involved in cell proliferation

P_KWMTBOMO07780	7091.BGIBMGA000903-TA	100	745	BGIBMGA000903-TA	Eukaryotic translation initiation factor 3 subunit M; Component of the eukaryotic translation initiation factor 3 (eIF-3) complex, which is involved in protein synthesis of a specialized repertoire of mRNAs and, together with other initiation factors, stimulates binding of mRNA and methionyl-tRNAi to the 40S ribosome. The eIF-3 complex specifically targets and initiates translation of a subset of mRNAs involved in cell proliferation
P_KWMTBOMO09542	7091.BGIBMGA013025-TA	99.9	1437.2	BGIBMGA013025-TA	Eukaryotic translation initiation factor 3 subunit A; RNA-binding component of the eukaryotic translation initiation factor 3 (eIF-3) complex, which is involved in protein synthesis of a specialized repertoire of mRNAs and, together with other initiation factors, stimulates binding of mRNA and methionyl-tRNAi to the 40S ribosome. The eIF-3 complex specifically targets and initiates translation of a subset of mRNAs involved in cell proliferation
P_KWMTBOMO09621	7091.BGIBMGA012851-TA	100	1412.9	eIF3c	Eukaryotic translation initiation factor 3 subunit C; Component of the eukaryotic translation initiation factor 3 (eIF-3) complex, which is involved in protein synthesis of a specialized repertoire of mRNAs and, together with other initiation factors, stimulates binding of mRNA and methionyl-tRNAi to the 40S ribosome. The eIF-3 complex specifically targets and initiates translation of a subset of mRNAs involved in cell proliferation
P_KWMTBOMO01606	7091.BGIBMGA006085-TA	100	862.4	BGIBMGA006085-TA	annotation not available
P_KWMTBOMO06231	7091.BGIBMGA001801-TA	82	555.8	BGIBMGA001801-TA	RuvB-like helicase; Proposed core component of the chromatin remodeling Ino80 complex which is involved in transcriptional regulation, DNA replication and probably DNA repair
P_KWMTBOMO14752	7091.BGIBMGA003833-TA	100	876.7	BGIBMGA003833-TA	annotation not available
P_KWMTBOMO14479	7091.BGIBMGA000074-TA	100	177.6	BGIBMGA000074-TA	annotation not available
P_KWMTBOMO05612	7091.BGIBMGA006772-TA	96.3	997.3	BGIBMGA006772-TA	annotation not available
P_KWMTBOMO00687	7091.BGIBMGA003221-TA	87.4	908.3	BGIBMGA003221-TA	annotation not available
P_KWMTBOMO15123	7091.BGIBMGA004908-TA	99.7	750.4	732870	annotation not available
P_KWMTBOMO10780	7091.BGIBMGA008426-TA	83.5	636.7	BGIBMGA008426-TA	annotation not available
P_KWMTBOMO14984	7091.BGIBMGA004782-TA	100	775.8	BGIBMGA004782-TA	annotation not available
P_KWMTBOMO04610	7091.BGIBMGA005315-TA	97.2	1604.7	BGIBMGA005315-TA	annotation not available
P_KWMTBOMO02835	7091.BGIBMGA003671-TA	96.9	452.2	BGIBMGA003671-TA	annotation not available

PIPs associated with Figure 6b

Query Item	String Id	Identity	Bit score	Preferred Name	Annotation
P_KWMTBOMO12389	7091.BGIBMGA012437-TA	100	585.1	COPE	Coatomer subunit epsilon; The coatomer is a cytosolic protein complex that binds to dilysine motifs and reversibly associates with Golgi non- clathrin-coated vesicles, which further mediate biosynthetic protein transport from the ER, via the Golgi up to the trans Golgi network. The coatomer complex is required for budding from Golgi membranes, and is essential for the retrograde Golgi-to-ER transport of dilysine-tagged proteins
P_KWMTBOMO15584	7091.BGIBMGA010725-TA	87.5	252.3	100379186	Coatomer subunit alpha; The coatomer is a cytosolic protein complex that binds to dilysine motifs and reversibly associates with Golgi non- clathrin-coated vesicles, which further mediate biosynthetic protein transport from the ER, via the Golgi up to the trans Golgi network
P_KWMTBOMO07589	7091.BGIBMGA013717-TA	75.1	1440.2	100379188	Coatomer subunit beta; The coatomer is a cytosolic protein complex that binds to dilysine motifs and reversibly associates with Golgi non- clathrin-coated vesicles, which further mediate biosynthetic protein transport from the ER, via the Golgi up to the trans Golgi network. Coatomer complex is required for budding from Golgi membranes, and is essential for the retrograde Golgi-to-ER transport of dilysine-tagged proteins
P_KWMTBOMO00860	7091.BGIBMGA009760-TA	95.4	322.4	692893	annotation not available
P_KWMTBOMO14157	7091.BGIBMGA011180-TA	99.8	1650.2	Copg2	Coatomer subunit gamma; The coatomer is a cytosolic protein complex that binds to dilysine motifs and reversibly associates with Golgi non- clathrin-coated vesicles, which further mediate biosynthetic protein transport from the ER, via the Golgi up to the trans Golgi network. Coatomer complex is required for budding from Golgi membranes, and is essential for the retrograde Golgi-to-ER transport of dilysine-tagged proteins
P_KWMTBOMO00427	7091.BGIBMGA000484-TA	100	1452.2	Copg1	Coatomer subunit gamma; The coatomer is a cytosolic protein complex that binds to dilysine motifs and reversibly associates with Golgi non- clathrin-coated vesicles, which further mediate biosynthetic protein transport from the ER, via the Golgi up to the trans Golgi network. Coatomer complex is required for budding from Golgi membranes, and is essential for the retrograde Golgi-to-ER transport of dilysine-tagged proteins
P_KWMTBOMO03027	7091.BGIBMGA003597-TA	100	359.4	LOC692966	annotation not available
P_KWMTBOMO08056	7091.BGIBMGA001119-TA	100	900.6	BGIBMGA001119-TA	Rab GDP dissociation inhibitor; Regulates the GDP/GTP exchange reaction of most RAB proteins by inhibiting the dissociation of GDP from them, and the subsequent binding of GTP
P_KWMTBOMO16481	7091.BGIBMGA002209-TA	100	421.8	Rab11	Ras-related GTP-binding protein Rab11
P_KWMTBOMO15361	7091.BGIBMGA005115-TA	100	414.5	Rabb	Rab1 protein; Small GTP-binding protein
P_KWMTBOMO01072	7091.BGIBMGA007414-TA	99.7	610.1	BGIBMGA007414-TA	annotation not available
P_KWMTBOMO08596	7091.BGIBMGA007971-TA	100	414.8	BGIBMGA007971-TA	annotation not available
P_KWMTBOMO15060	7091.BGIBMGA004852-TA	100	1466.1	BGIBMGA004852-TA	annotation not available
P_KWMTBOMO07214	7091.BGIBMGA010574-TA	72.1	1424.1	100463492	annotation not available
P_KWMTBOMO05281	7091.BGIBMGA002504-TA	100	404.8	BGIBMGA002504-TA	annotation not available
P_KWMTBOMO10283	7091.BGIBMGA007268-TA	100	874.8	LOC692900	annotation not available

P_KWMTBOMO01114	7091.BGIBMGA007332-TA	100	867.5	BGIBMGA007332-TA	annotation not available
P_KWMTBOMO01312	7091.BGIBMGA009010-TA	69.8	387.1	692958	annotation not available
P_KWMTBOMO10780	7091.BGIBMGA008426-TA	83.5	636.7	BGIBMGA008426-TA	annotation not available
P_KWMTBOMO00837	7091.BGIBMGA009684-TA	100	530.8	BGIBMGA009684-TA	Proteasome subunit beta type
P_KWMTBOMO10916	7091.BGIBMGA004657-TA	33.6	132.1	LOC732998	Proteasome subunit alpha type; The proteasome is a multicatalytic proteinase complex which is characterized by its ability to cleave peptides with Arg, Phe, Tyr, Leu, and Glu adjacent to the leaving group at neutral or slightly basic pH; Belongs to the peptidase T1A family
P_KWMTBOMO10965	7091.BGIBMGA004908-TA	53.2	388.7	732870	annotation not available
P_KWMTBOMO04610	7091.BGIBMGA005315-TA	97.2	1604.7	BGIBMGA005315-TA	annotation not available
P_KWMTBOMO02835	7091.BGIBMGA003671-TA	96.9	452.2	BGIBMGA003671-TA	annotation not available
P_KWMTBOMO14984	7091.BGIBMGA004782-TA	100	775.8	BGIBMGA004782-TA	annotation not available
P_KWMTBOMO15518	7091.BGIBMGA010794-TA	100	711.8	LOC733024	annotation not available
P_KWMTBOMO04330	7091.BGIBMGA014136-TA	100	792.3	BGIBMGA014136-TA	annotation not available
P_KWMTBOMO10129	7091.BGIBMGA005560-TA	100	530.4	LOC778515	Proteasome 26S non-ATPase subunit 4
P_KWMTBOMO14041	7091.BGIBMGA011237-TA	95.2	648.3	693113	annotation not available
P_KWMTBOMO12628	7091.BGIBMGA001558-TA	99.8	2481.8	BGIBMGA001558-TA	annotation not available
P_KWMTBOMO16357	7091.BGIBMGA013792-TA	79	293.9	RpS11	annotation not available
P_KWMTBOMO15359	7091.BGIBMGA005116-TA	100	1431	BGIBMGA005116-TA	annotation not available
P_KWMTBOMO09232	7091.BGIBMGA003337-TA	100	429.9	RpL10A	annotation not available
P_KWMTBOMO00838	7091.BGIBMGA009751-TA	100	276.2	RpL23	Ribosomal protein L23 ; Belongs to the universal ribosomal protein uL14 family
P_KWMTBOMO08440	7091.BGIBMGA009411-TA	99.3	293.1	RpS16	annotation not available
P_KWMTBOMO09838	7091.BGIBMGA013133-TA	99.1	1125.5	BGIBMGA013133-TA	annotation not available
P_KWMTBOMO02477	7091.BGIBMGA002572-TA	99	348.2	RpL19	Ribosomal protein L19 ; Belongs to the eukaryotic ribosomal protein eL19 family
P_KWMTBOMO08961	7091.BGIBMGA007795-TA	100	223.4	RpL30	Ribosomal protein L30
P_KWMTBOMO15114	7091.BGIBMGA004905-TA	98.3	172.6	RpS25	annotation not available
P_KWMTBOMO02088	7091.BGIBMGA002984-TA	100	1116.7	BGIBMGA002984-TA	Lysine--tRNA ligase
P_KWMTBOMO14530	7091.BGIBMGA014398-TA	100	818.1	LOC692937	annotation not available
P_KWMTBOMO11336	7091.BGIBMGA001849-TA	98.1	1407.1	BGIBMGA001849-TA	annotation not available
P_KWMTBOMO15357	7091.BGIBMGA005117-TA	98.3	1544.3	BGIBMGA005117-TA	annotation not available
P_KWMTBOMO01273	7091.BGIBMGA008865-TA	100	416.8	RpL13A	Ribosomal protein L13A
P_KWMTBOMO01452	7091.BGIBMGA008930-TA	100	1336.6	BGIBMGA008930-TA	annotation not available
P_KWMTBOMO11255	7091.BGIBMGA012626-TA	100	375.2	RpS9	Ribosomal protein S9 ; Belongs to the universal ribosomal protein uS4 family
P_KWMTBOMO12706	7091.BGIBMGA001991-TA	100	203.4	RpL36	Ribosomal protein L36
P_KWMTBOMO09442	7091.BGIBMGA013076-TA	95.2	499.6	RpL6	annotation not available
P_KWMTBOMO08045	7091.BGIBMGA001037-TA	99.7	1771.5	BGIBMGA001037-TA	annotation not available
P_KWMTBOMO09230	7091.BGIBMGA003412-TA	100	135.6	RpP1	annotation not available
P_KWMTBOMO01055	7091.BGIBMGA007363-TA	100	231.5	RpS26	Ribosomal protein S26 ; Belongs to the eukaryotic ribosomal protein eS26 family
P_KWMTBOMO10337	7091.BGIBMGA007230-TA	90	995.3	Elf	annotation not available
P_KWMTBOMO06974	7091.BGIBMGA010487-TA	100	179.1	RpL36A	Ribosomal protein L36A ; Belongs to the eukaryotic ribosomal protein eL42 family
P_KWMTBOMO15121	7091.BGIBMGA004993-TA	100	960.3	Srp54	annotation not available
P_KWMTBOMO12560	7091.BGIBMGA001525-TA	64.4	591.7	BGIBMGA001525-TA	annotation not available
P_KWMTBOMO01148	7091.BGIBMGA007477-TA	99.4	587	Rack1	annotation not available
P_KWMTBOMO00722	7091.BGIBMGA003259-TA	98	1843.9	101739633	annotation not available
P_KWMTBOMO13960	7091.BGIBMGA011282-TA	100	301.6	RpS13	Ribosomal protein S13 ; Belongs to the universal ribosomal protein uS15 family

P_KWMTBOMO14639	7091.BGIBMGA000066-TA	100	262.7	RpL32	Ribosomal protein L32
P_KWMTBOMO13370	7091.BGIBMGA010941-TA	88.9	1065.4	BGIBMGA010941-TA	annotation not available
P_KWMTBOMO10248	7091.BGIBMGA007311-TA	100	547	RpSA	40S ribosomal protein SA; Required for the assembly and/or stability of the 40S ribosomal subunit. Required for the processing of the 20S rRNA- precursor to mature 18S rRNA in a late step of the maturation of 40S ribosomal subunits
P_KWMTBOMO06480	7091.BGIBMGA011620-TA	100	351.3	RpL18	60S ribosomal protein L18
P_KWMTBOMO12428	7091.BGIBMGA012414-TA	100	357.5	RpL17	60S ribosomal protein L17 ; Belongs to the universal ribosomal protein uL22 family
P_KWMTBOMO04768	7091.BGIBMGA009851-TA	94.7	2035.8	BGIBMGA009851-TA	annotation not available
P_KWMTBOMO00927	7091.BGIBMGA009794-TA	100	688	BGIBMGA009794-TA	annotation not available
P_KWMTBOMO08894	7091.BGIBMGA007710-TA	100	433	RpS5	Ribosomal protein S5 ; Belongs to the universal ribosomal protein uS7 family
P_KWMTBOMO11680	7091.BGIBMGA003977-TA	100	989.6	BGIBMGA003977-TA	annotation not available
P_KWMTBOMO08666	7091.BGIBMGA007645-TA	100	285.8	RpS23	annotation not available
P_KWMTBOMO03951	7091.BGIBMGA010139-TA	100	181.4	RpS27	40S ribosomal protein S27
P_KWMTBOMO12018	7091.BGIBMGA004356-TA	98.4	343.6	RpS7	annotation not available
P_KWMTBOMO07772	7091.BGIBMGA001243-TA	100	301.6	RpS10	annotation not available

Supplementary Table S6. List of TFs associated with Figure S5.

Query Item	String Id	Identity	Bit score	Preferred Name	Annotation
P_KWMTBOMO04742	7091.BGIBMGA009863-TA	100	270.8	RpL27	60S ribosomal protein L27 ; Belongs to the eukaryotic ribosomal protein eL27 family
P_KWMTBOMO05675	7091.BGIBMGA006835-TA	100	250	RpL23A	annotation not available
P_KWMTBOMO05810	7091.BGIBMGA006945-TA	68.5	2686	732871	annotation not available
P_KWMTBOMO07323	7091.BGIBMGA010666-TA	95.3	968.8	LOC692795	T-complex protein 1 subunit delta; Molecular chaperone; assists the folding of proteins upon ATP hydrolysis
P_KWMTBOMO09808	7091.BGIBMGA013116-TA	100	1023.5	LOC692796	Chaperonin subunit 6a zeta; Molecular chaperone; assists the folding of proteins upon ATP hydrolysis
P_KWMTBOMO11731	7091.BGIBMGA004165-TA	100	1686.8	tef2	Translation elongation factor 2; Catalyzes the GTP-dependent ribosomal translocation step during translation elongation. During this step, the ribosome changes from the pre-translocational (PRE) to the post- translocational (POST) state as the newly formed A-site-bound peptidyl-tRNA and P-site-bound deacylated tRNA move to the P and E sites, respectively. Catalyzes the coordinated movement of the two tRNA molecules, the mRNA and conformational changes in the ribosome
P_KWMTBOMO12659	7091.BGIBMGA001406-TA	100	1041.6	BGIBMGA001406-TA	T-complex protein 1 subunit gamma; Molecular chaperone; assists the folding of proteins upon ATP hydrolysis
P_KWMTBOMO14887	7091.BGIBMGA012182-TA	99.6	1052	BGIBMGA012182-TA	Uncharacterized protein; Molecular chaperone; assists the folding of proteins upon ATP hydrolysis
P_KWMTBOMO01796	7091.BGIBMGA006158-TA	100	519.2	LOC692987	Prohibitin protein WPH
P_KWMTBOMO11061	7091.BGIBMGA008312-TA	100	565.1	LOC693108	Mitochondrial prohibitin complex protein 2

Supplementary Table S7. Primer sequences used in this study.

Name	Primer sequences (5′ → 3′)
fibH-BF	bio-GGTACCAGATAACGGATATAAATG
fibH-F	GGTACCAGATAACGGATATAAATG
fibH-R	GCTAGCCTTGAGAGTTGG
fibL-BF	bio-TAACAAAGTGGTGCCTATCC
fibL-F	TAACAAAGTGGTGCCTATCC
fibL-R	TTTAGTGGTCTGTTATGTGACCAATC
P25-BF	bio-CGCCACTGAGTCGCATTA
P25-F	CGCCACTGAGTCGCATTA
P25-R	GTTGCGCGAATAATAAACAACTG
GFP-F	ATGGAGAGCGACGAGAGC
GFP-R	GCGAGATCCGGTGGAGCC
